# rboAnalyzer: A Software to Improve Characterization of Non-coding RNAs From Sequence Database Search Output

**DOI:** 10.3389/fgene.2020.00675

**Published:** 2020-07-28

**Authors:** Marek Schwarz, Jiří Vohradský, Martin Modrák, Josef Pánek

**Affiliations:** Laboratory of Bioinformatics, Institute of Microbiology, Czech Academy of Sciences, Prague, Czechia

**Keywords:** RNA, sequence, database, search, secondary structure, RNA homology

## Abstract

Searching for similar sequences in a database via BLAST or a similar tool is one of the most common bioinformatics tasks applied in general, and to non-coding RNAs in particular. However, the results of the search might be difficult to interpret due to the presence of partial matches to the database subject sequences. Here, we present rboAnalyzer – a tool that helps with interpreting sequence search result by (1) extending partial matches into plausible full-length subject sequences, (2) predicting homology of RNAs represented by full-length subject sequences to the query RNA, (3) pooling information across homologous RNAs found in the search results and public databases such as Rfam to predict more reliable secondary structures for all matches, and (4) contextualizing the matches by providing the prediction results and other relevant information in a rich graphical output. Using predicted full-length matches improves secondary structure prediction and makes rboAnalyzer robust with regards to identification of homology. The output of the tool should help the user to reliably characterize non-coding RNAs in BLAST output. The usefulness of the rboAnalyzer and its ability to correctly extend partial matches to full-length is demonstrated on known homologous RNAs. To allow the user to use custom databases and search options, rboAnalyzer accepts any search results as a text file in the BLAST format. The main output is an interactive HTML page displaying the computed characteristics and other context of the matches. The output can also be exported in an appropriate sequence and/or secondary structure formats.

## Introduction

The output of a BLAST (Camacho et al., 2009) search is a list of hits of the query sequence in the search database that are called *high-scoring pairs* (HSPs). They are characterized by their statistically estimated quality and position within the sequences in the search database. A HSP contains the sequences of the matched RNA and the query RNA that are similar to each other. These sequences can be either full sequences or fragments of the full sequences, so called *partial matches*.

Since it is frequently impossible to reliably determine secondary structure, homology and function from a fragment of a non-coding RNA, the interpretation of results of a sequence search for non-coding RNAs requires full-length sequences of the matched RNAs. The full-length sequences of the partial matches are usually identified manually using external bioinformatics tools for individual RNAs which can be laborious and inefficient. The aim of the presented tool, rboAnalyzer, is to replace the manual work by an automated workflow and thereby to make the interpretation of the database sequence search results easier.

The rboAnalyzer pipeline extends partial or otherwise imperfect matches to the length of the query sequence and computes its secondary structure and a homology to the query RNA. These tasks are handled with available bioinformatics algorithms integrated into a framework that combines the information contained in the BLAST output with external sources such as Rfam (Nawrocki et al., 2015). rboAnalyzer runs from command line. Its input consist of a BLAST output text file, a FASTA file with the query RNA sequence and the database used in the search. The output is a HTML page that integrates the computed characteristics of the subject RNAs together with the subject RNAs data. Results are presented in a clear, interactive and exportable form.

While the presented version of rboAnalyzer takes BLAST results as an input, the algorithm is general and can be easily extended to accept matches obtained with other database sequence search tools.

## Implementation

**FIGURE 1:**
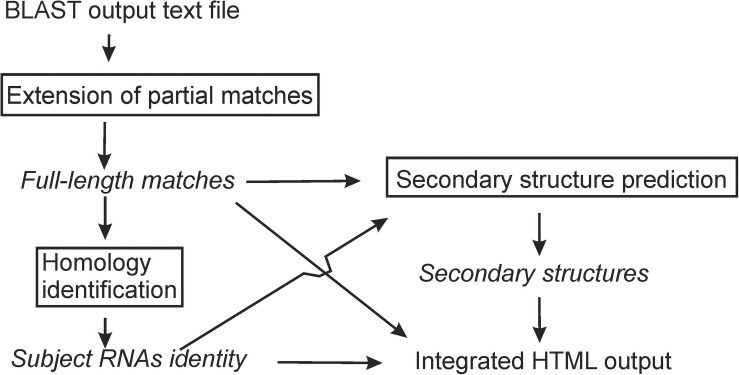
An rboAnalyzer flowchart. The names of analysis steps are in boxes. The names of data and information are in italics. The arrows indicate the data and information flow.

rboAnalyzer operates in three steps (Figure 1): (1) extension of partial matches to their probable full-length, (2) homology identification of the subject RNAs, and (3) secondary structure prediction. All the information is then integrated into a HTML output. rboAnalyzer runs on Linux and was implemented using Bash and Python 3 with the Biopython (Cock et al., 2009), NumPy (Oliphant, 2006), Pandas (McKinney, 2010), matplotlib (Hunter, 2007), and Jinja2^1^ libraries.

Step 1: Extending partial matches to full length

**FIGURE 2:**
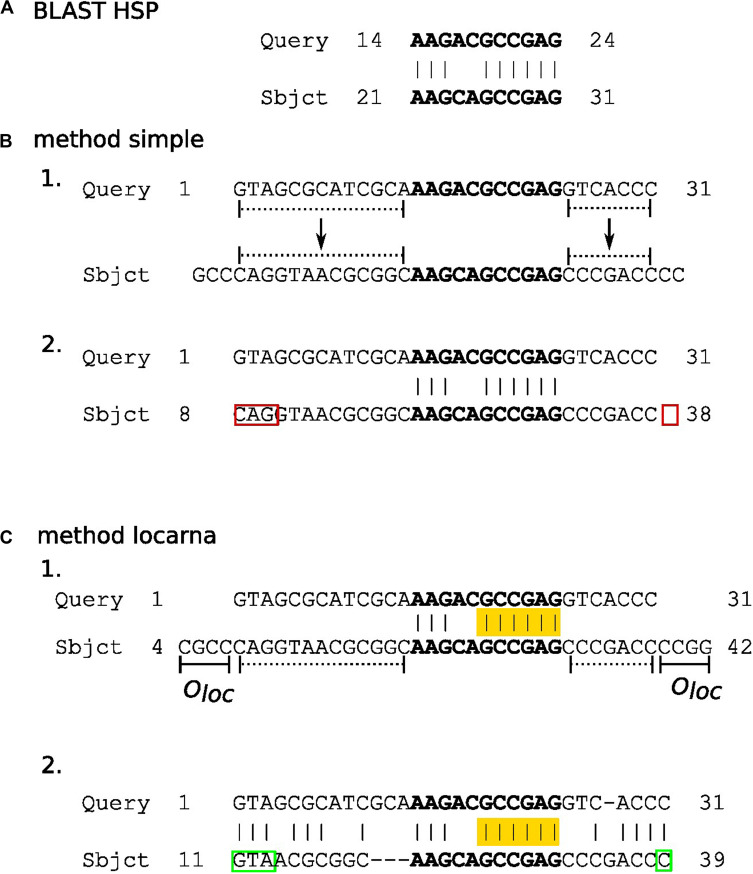
The diagram outlying “simple” and “locarna” methods for extension of partial matches. Panels **(A–C)** demonstrate BLAST HSP, “simple,” and “locarna” methods, respectively. Panel **(A)** shows an example of BLAST HSP. The HSP consists of a partial match between the query and subject sequences. The match (with nucleotides highlighted in bold) is located between nucleotides 14 and 24 on the query sequence, while between nucleotides 21 and 31 on the subject sequence. The partial match of the subject sequence is extended to its predicted full length using the “simple” and “locarna” methods, demonstrated in panels (B, C), respectively. Panel **(B)** depicts the “simple” method. The example query sequence with 31 nucleotides is shown and marked as “Query” on its 5′ end. In section B 1. the partial match of the subject sequence (with nucleotides in bold on “Sbjct” sequence) is extended to its predicted full length simply by adding the parts of the subject sequence according to the length of the query sequence to its both ends. These parts are marked by dotted lines. Section B 2 shows the result of the extension, i.e., the extended partial match for the subject sequence. It lies between nucleotides 8 and 38 on the subject sequence. The original HSP is highlighted in bold. The red rectangles mark errors that can be either mismatched or missing nucleotides, marked by 5′ and 3′ red rectangles, respectively, in the diagram. The errors may be produced due to direct simple assignment of unaligned 5′ and 3′ flanking sequences of partial match without nucleotide context. Panel **(C)** depicts the “locarna” method. In section C 1., the partial match of the subject sequence is extended so it can be aligned to the query sequence by “locarna,” while anchored at HSP. The anchor is highlighted in yellow. The extension sequences at the both ends of the partial match of the subject sequence consists each of the extension to the length of the query sequence (dotted lines) and an extra sequence of an empirically chosen length of *O*_loc_ nucleotides (solid lines denoted as *O*_loc_). For explanation, why this is used, please, refer to the main text. Section C 2. depicts the result of the “locarna” alignment. The extended partial match for subject sequence is between nucleotides 11 and 39 of the subject sequence and it is the final result, i.e., the extended partial match of the subject sequence. Note, that the “locarna” anchored alignment eliminated the errors produced by the “simple” method (B.2) due to taking into account nucleotide context of 5′ and 3′ flanking sequences of the partial match and therefore the correct nucleotides were assigned (marked by green rectangles).

In this step, partial matches of subject sequences in HSPs (Figure 2) are extended to their predicted full-length in sequences in the underlying database. We accomplish this extension by three methods termed here “simple,” “locarna,” and “meta.”

In the “simple” method (Figure 2B), the lengths of the missing 5′ and 3′ stretches of the subject sequences are computed by directly extending partial matches in HSPs by the number of unmatched bases of the query sequence on either end.

The “locarna” method (Figure 2C) starts with the sequence of extended match determined by the “simple” extension method plus *o*_loc_ number of nucleotides on its either end copied from the database sequence. Then, this extended sequence and the query sequence are aligned using LocaRNA P sequence alignment algorithm (Will et al., 2012). The ungapped stretches of the matched bases in the original HSP are used as anchors. “No gap cost for end gaps” parameter is used to ensure that the sequence is aligned with respect to the original HSP. Then, the final sequence of extended match is identified in the “locarna” alignment as the parts of the match sequence aligned with the query sequence.

We have set *o*_loc_ = *30* after optimization over a set of known RNAs (see the Supplementary Material “Optimization of rboAnalyzer step i) -Estimation of full-length subject sequences, methods and parameters” for details).

In the “meta” extension method, rboAnalyzer uses both of the above described approaches to obtain two versions of the full-length match. Then it chooses the better one of the two sequences according to a score obtained by comparison of the two full-length sequences to the covariance model for the query sequence. The covariance model is computed by RSEARCH (Klein and Eddy, 2003) from the query sequence and the predicted secondary structure of the query RNA. The structure is predicted by RNAFold (Tafer et al., 2011). The user can also choose to use either a covariance model from Rfam that best matches the query sequence, or can provide his own model directly.

### Evaluating Partial Matches Extension Methods

To evaluate the performance of the methods for extension of partial matches we prepared a dataset with known RNA sequences located at known positions in the database sequence. The database sequence was constructed artificially using sequences of RNAs families in CompaRNA dataset (Puton et al., 2013). CompaRNA contains those Rfam families that have at least one homolog with experimentally identified structure. Of the families in CompaRNA, we used only those whose Rfam seed alignments included at least 20 homologs. The homolog with experimentally identified structure was used as a template for evaluation of the accuracy of our secondary structure prediction.

The above mentioned criteria were fulfilled by the following RNA families: RF00001, RF00002, RF00005, RF00008, RF00015, RF00017, RF00020, RF00095, RF00100, RF00162, RF00167, RF00169, RF00175, RF00209, RF00230, RF00250, RF00374, RF00379, RF00380, RF00480, RF01051, RF01725, RF01739, RF01807, RF01831, RF01852, RF02095, RF02253, and RF02348. For each family, three RNA sequences were chosen randomly and set aside to be used as query sequences. The remaining sequences were used to construct an artificial subject sequence, in which they were placed one after another, separated from each other by their 1000 nucleotides long 5′ and 3′ flanking regions, forming a long single sequence. The sequences of the flanking regions were obtained from NCBI using Rfam accession numbers of appropriate RNAs. When flanking regions with 1000 nucleotides were not available, a random sequence was used to fill the missing section.

Furthermore, to create decoys in the artificial subject sequence, the same RNA sequences were shuffled 10 times each, and together with their flanking regions included into the artificial subject sequence in the same way as for the original RNAs. For this artificial subject sequence, a BLAST database was build using makeblastdb program (Camacho et al., 2009).

**FIGURE 3:**
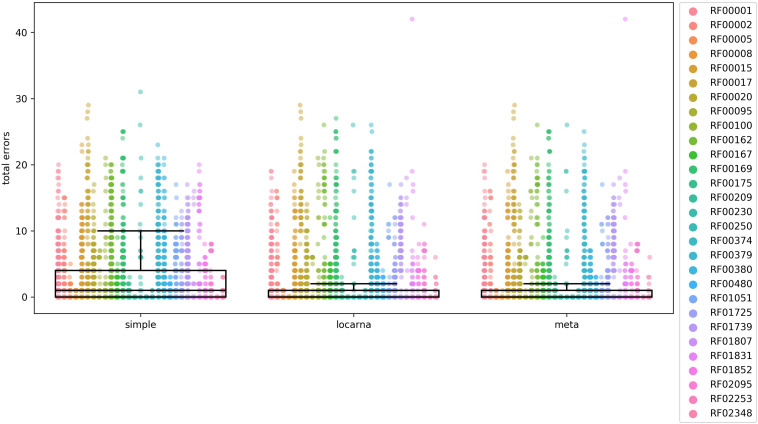
Comparison of accuracy of the three methods for extending partial matches. The comparison was carried out using 29 Rfam RNA families, listed on the right side of the figure and indicated by colors. The total error on y-axis is a number of nucleotides that are either missing or are in excess in comparison to reference Rfam sequences at both 5′ and 3′ ends of estimated full-length sequences. In the case of “locarna” and “meta” the median line is located at the very bottom of the box. To compare the accuracy of each of the three methods we used Mann–Whitney *U* test. The differences were significant between “simple” and “locarna” (*p* < 0.001) and between “simple” and “meta” (*p* < 0.001) methods, while the difference between “locarna” and “meta” was not significant (*p* = 0.34), saying that the “simple” method had the worst accuracy of the three methods, while accuracy of the “meta” and “locarna” was similar. We therefore chose the “locarna” method as the default method as its accuracy is better than that of “simple” method and it is faster than “meta” method.

This database was searched by the three query RNA sequences set aside previously for each RNA family to generate BLAST outputs using blastn program with parameters: -gapopen 2 -gapextend 1 -penalty -1 -reward 1 -word_size 7. The outputs were analyzed using rboAnalyzer and only the BLAST hits of the query sequences into homologous RNAs were considered for comparison. The partial matches were extended using the three extension methods and the result of the comparison are summarized in Figure 3.

The box plots in Figure 3 indicated that the “simple” extension method was least accurate. The accuracies of “meta” and “locarna” methods were comparable, but the “locarna” method was faster, as the “meta” method is a combination of “simple” and “locarna” methods. We therefore chose the “locarna” method as the default extension method (see Supplementary Material “Comparison of three methods for extending partial matches –Figure 3” for sum of errors).

Step 2: Identification of homology of subject RNAs

In this step, the homology of the subject RNAs to the query RNA was identified using sequences of extended matches of the subject RNAs. First, a covariance model of the query RNA was computed with RSEARCH, as described for the “meta” extension method in Step 1. Then, rboAnalyzer scored each of the extended matches by comparing it to the covariance model using cmalign (Nawrocki and Eddy, 2013). The score is a measure of the homology of the subject RNAs to the query RNA in terms of similarity of their sequences represented by their extended matches and their potential secondary structure. Based on the score, rboAnalyzer classified subject RNAs into three categories:

$$\left\{ \begin{array}{cc} \mathrm{homologous} & if\frac{s}{l}\geq 0.5\wedge s\geq 20\\ not\mathrm{homologous} & ifs<0\\ uncertain & otherwise \end{array}\right.,$$

where *s* is the score and *l* the length of the query sequence. The term *s/l* ≥ 0.5 for “homologous” is used to guarantee that the similarity between the subject sequence and CM model of the query sequence, from which the bit score is derived, is created by at least a half of a bit per one nucleotide of the query sequence. This way we want to filter out fragments with relatively high local similarity that are not homologous. The dependence of the threshold on the length of the query sequence reflects the fact that for longer sequences the length of the fragment with locally concentrated similarity can be longer.

The information about homology of subject RNA to the query RNA is included in the rboAnalyzer output.

Step 3: Prediction of secondary structure of subject RNAs

The subject RNAs are further characterized by a secondary structure predicted using their extended matches.

rboAnalyzer offers 15 methods for the secondary structure prediction implemented using available algorithms and their combinations in order to efficiently exploit the information in the BLAST output. The list and brief description of each method is outlined in Table 1 (for more details, see Supplementary Table S2 and online documentation).

**TABLE 1:** List of secondary structure prediction methods implemented in rboAnalyzer with brief description and advantages/disadvantages related to prediction of secondary structures for full matches.

Method name (category*)	Brief description	Advantages (+)/disadvantages (–)
rnafold (2)	*de novo* prediction by RNAfold	+ Fast + Universal − Single sequence prediction that do not employ any additional information from BLAST output
C-A-r-Rc (1)/ M-A-r-Rc (1)	Uses conserved base pairs in consensus secondary structure built from MSA of conserved full matches extended from HSPs in BLAST output to add information to RNAfold prediction	+ Information about conserved base pairs carried to individual structure prediction −Good alignment for consensus structure prediction may be hard to obtain especially when the BLAST output was from restricted search space
C-A-U-r-Rc (1)/ M-A-U-r-Rc (1)	The same as for C-A-r-Rc/M-A-r-Rc, but uses conserved unpaired bases	The same as for C-A-r-Rc/M-A-r-Rc, but applying to the conserved unpaired bases
rfam-Rc (2)	Uses consensus structure from covariance model to add information to RNAfold prediction	+ Based on curated information from either Rfam or user −Can’t be used when the covariance model with secondary structure is not available
rfam-centroid (2)	Uses sequence information from covariance model for CentroidHomfold prediction	+ Based on curated information from either Rfam or user −Can’t be used when the covariance model is not available
C-A-sub (1)/ M-A-sub (1)	Uses consensus secondary structure built from MSA of conserved full matches extended from HSPs in BLAST output to select the best of suboptimal secondary structures generated by hybrid-ss-min prediction	+ Suboptimal secondary structure may provide better prediction then minimum free energy (MFE) predictions −Good alignment for consensus structure prediction may be hard to obtain especially when the BLAST output was from restricted search space
rfam-sub (2)	Uses consensus secondary structure from covariance model to select the best of suboptimal secondary structures generated by hybrid-ss-min prediction	+ Suboptimal secondary structures may provide better prediction then MFE predictions −Can’t be used when the covariance model with secondary structure is not available
fq-sub (2)	Secondary structure of query sequence predicted by RNAfold is used to select the best of suboptimal secondary structures generated by hybrid-ss-min prediction	+ Useful when secondary structure predicted by RNAfold is correct + Suboptimal secondary structures may provide better prediction then MFE predictions −No additional information from BLAST output is used
centroid (1)/centroid-fast (1)	Uses sequence information from full-length matches (either conserved or first few) for CentroidHomfold secondary structure prediction	+ Design of CentroidHomfold well suited for this task
TurboFold (1)/Turbo-fast (1)	Uses sequence information from full-length matches (either conserved or first few) for TurboFold secondary structure prediction	+ Best performing without reference data^†^ − Slow − Memory demanding − Can’t be used when sequence contains ambiguous bases

*The categories are defined in Step 3: Prediction of secondary structure of subject RNAs section. ^†^See Supplementary Material “Comparison of methods for prediction of secondary structures from estimated full-length sequences”.

There are two broad categories of structure prediction methods: those using multiple subject sequences from the BLAST output (category 1) and those which do not use them (category 2).

The methods from the category 1 use the extended matches (Step 1) of those RNAs that were determined as homologous to the query RNA (Step 2). Among the sequences of the extended matches of these RNAs, each prediction method selects sequences which it will use as reference sequences for prediction. The selection is based on individually optimized threshold for similarity of the sequences to the query. rboAnalyzer also does not use sequences that are either identical or very similar to the query as they can distort prediction. The level of the sequence similarity is determined by the parameter optimized individually for each of the methods.

The sequences are used in two ways depending on the prediction methods:

(a) to build a reference consensus secondary structure using RNAalifold (Bernhart et al., 2008) with the multiple sequence alignment made by Clustal Omega (Sievers et al., 2011) or muscle (Edgar, 2004), followed by refold.pl (Tafer et al., 2011)/RNAfold –C (Lorenz et al., 2016), or UNAfold (Markham and Zuker, 2008);
(b) to serve as reference sequences by the methods based on TurboFold (Tan et al., 2017) or CentroidHomfold (Hamada et al., 2009).

The methods belonging to the category 2 include RNAfold, which is a *de novo* prediction method, then prediction methods that use covariance models identified in Rfam, and finally, a prediction method that uses RNAfold to predict secondary structure of the query RNA, which is then used as a structural template for finding a best matching structure among suboptimal structures of subject RNA predicted by UNAfold.

## Results

### Default Values of rboAnalyzer Parameters

The default set-up for rboAnalyzer includes “locarna” method for extension of partial matches and three methods for the secondary structure prediction, RNAfold, TurboFold, and rfam-Rc (which is a shortcut for RNAfold –C with a Rfam consensus structure as constraint). TurboFold was chosen as it performed best of all the prediction methods. RNAfold was chosen as a standard with minimum input providing an output under any conditions. RNAfold –C with a Rfam consensus structure as constraint was chosen as a representative of the methods using information from an external source. The three selected methods were chosen as they are based on different prediction principles and guarantee that the user gets most accurate prediction available. The secondary structure prediction methods performance test is summarized in Supplementary Table S3.

rboAnalyzer has numerous parameters that belong to the algorithms used for its construction. Their default values were optimized using RNAs with experimentally identified secondary structures. The list of the parameters and the algorithms used in rboAnalyzer and the details about the parameter optimization are described in the Supplementary Material “Optimization of rboAnalyzer parameters.”

### Test of rboAnalyzer Performance

Here, the performance of rboAnalyzer with respect to the quality of HSPs in a BLAST output was tested.

We first created a synthetic sequence database from the sequences of the RNA families selected using the criteria described in the “Evaluating partial matches extension methods” section above.

For each RNA family we downloaded sequences from CompaRNA dataset which were then used as queries for BLAST. Then we extracted sequences from Rfam seed alignment for each RNA family that were used as known homologs. For each of them we downloaded its parent sequence from NCBI that were placed between sequences of its randomly shuffled 500 nucleotide long 5′ and 3′ flanking regions. These sequences were used to construct a BLAST database, in which the query RNAs with experimentally identified structures were searched with blastn program (parameters: -gapopen 2 -gapextend 1 -penalty -1 -reward 1 -word_size 7) obtaining BLAST outputs. For the families that included more than one RNA with experimentally identified secondary structure, more than one BLAST output were obtained and of them the one with the largest number of matches to the sequences from Rfam seed alignments was used for further analysis.

Matches in each BLAST output were sorted into three groups according to their quality. The quality was defined as high, moderate and low and the thresholds were set so that each of the three groups contained equal number of matches to the sequences from Rfam seed alignments. Finally, the groups were used to form new synthetic BLAST outputs resulting into three synthetic outputs for each of the original BLAST outputs. These synthetic outputs were analyzed by rboAnalyzer to find out how its performance depends on the quality of BLAST outputs.

The performance was measured by structural similarity between the secondary structures of subject RNAs predicted using sequences of their extended partial matches and the experimentally identified structures of the query RNAs. In this test, the subject RNAs and the query RNAs were known to be homologous as they came from the same families, and therefore the predicted secondary structures and experimentally identified structures should be similar if the rboAnalyzer characterizing pipeline was correct and accurate. The higher the similarity, the higher the overall performance of rboAnalyzer, because the secondary structure prediction is the final step of rboAnalyzer and therefore depends on the performance of the previous steps.

**FIGURE 4:**
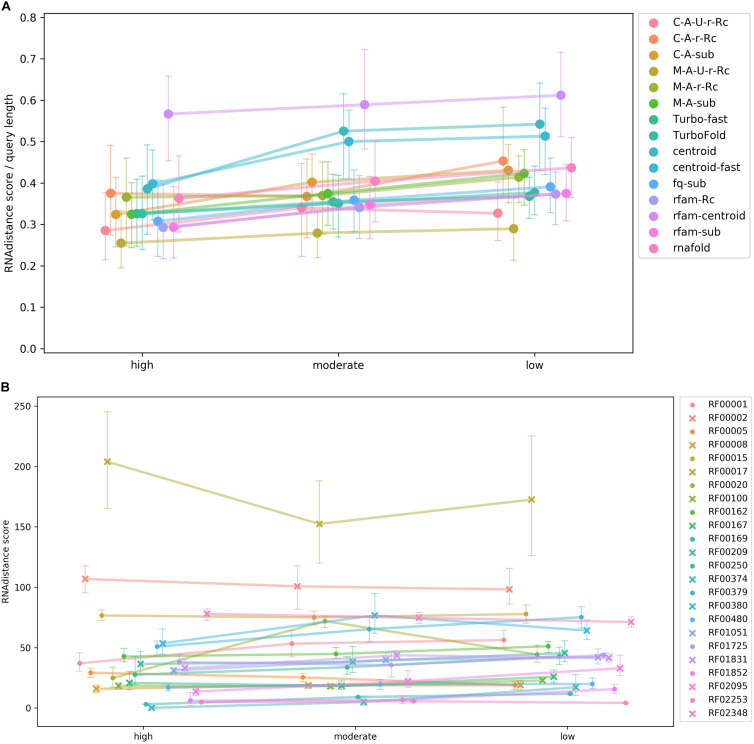
A test of rboAnalyzer performance depending on quality of BLAST HSPs. The performance was measured using quality of predicted secondary structures. The secondary structure prediction is the last step of the rboAnalyzer analysis and therefore depends on the quality of all the previous steps, thus reflecting an overall rboAnalyzer performance. The quality was computed by matching the predicted secondary structures to their experimentally identified templates. The test was performed using RNAs from selected Rfam families that were searched by BLAST in a database that we constructed using the Rfam RNAs. Full length sequences of the Rfam RNAs were extended by rboAnalyzer from their partial matches in BLAST HSPs. The HSPs were assigned one of three levels of quality based on BLAST bit score: high, moderate and low. For definition of the quality levels, refer to the *Test of rboAnalyzer performance* subsection of the Results section. The HSPs depending on their assigned quality level were copied from the original, native BLAST output into one of three artificially constructed BLAST outputs. The outputs were three, physically three files, each containing HSPs only of one quality level. From partial matches in HSPs in these three outputs, the RNAs were reconstructed by rboAnalyzer. The HSP quality levels referring to the three outputs are shown on the horizontal axis in panels **(A,B)**. The quality of predicted secondary structures shown on the vertical axis in panels **(A,B)** is measured as a mean of tree edit distances [RNAdistance (Tafer et al., 2011)] between the predicted secondary structures of subject RNAs and experimentally identified structures in the test Rfam families. Each of the families contained at least one experimentally identified structure used as a structure template. Note that the lower the mean (vertical axis), the higher the structure similarity, as the similarity is measured using the tree edit distance. Therefore, lower values on vertical axis indicate a higher quality and a better overall rboAnalyzer performance. The colors of the curves in both panels indicate either a secondary structure prediction method **(A)** or a test Rfam RNA family **(B)**. The colors are defined on the right hand side of the panels. Panel **(A)** shows rboAnalyzer performance depending on the secondary structure prediction methods. In the graph, the tree edit distances of individual RNAs were averaged for individual prediction methods across the Rfam families. The tree edit distances were normalized to the length of respective query sequences. Panel **(B)** shows performance depending on test Rfam families. In the graph, the tree edit distances were averaged for individual Rfam families across the prediction methods. The error bars on the curves in the both panels represent 95% intervals from 100 bootstrap iterations. The families RF00095, RF00175, RF00230, RF01739, and RF01807 did not have enough matches into the sequences from Rfam seed alignments to divide their BLAST outputs to the three parts according to the quality of HSPs and therefore were excluded from the analysis.

The results of the test were summarized in Figure 4. The rather flat curves showed steady rboAnalyzer performance regardless of the quality of HSPs in input BLAST outputs. This indicated that rboAnalyzer is robust and capable to produce accurate secondary structures even for short partial matches of subject RNAs.

## Example Usage of rboAnalyzer

**FIGURE 5:**
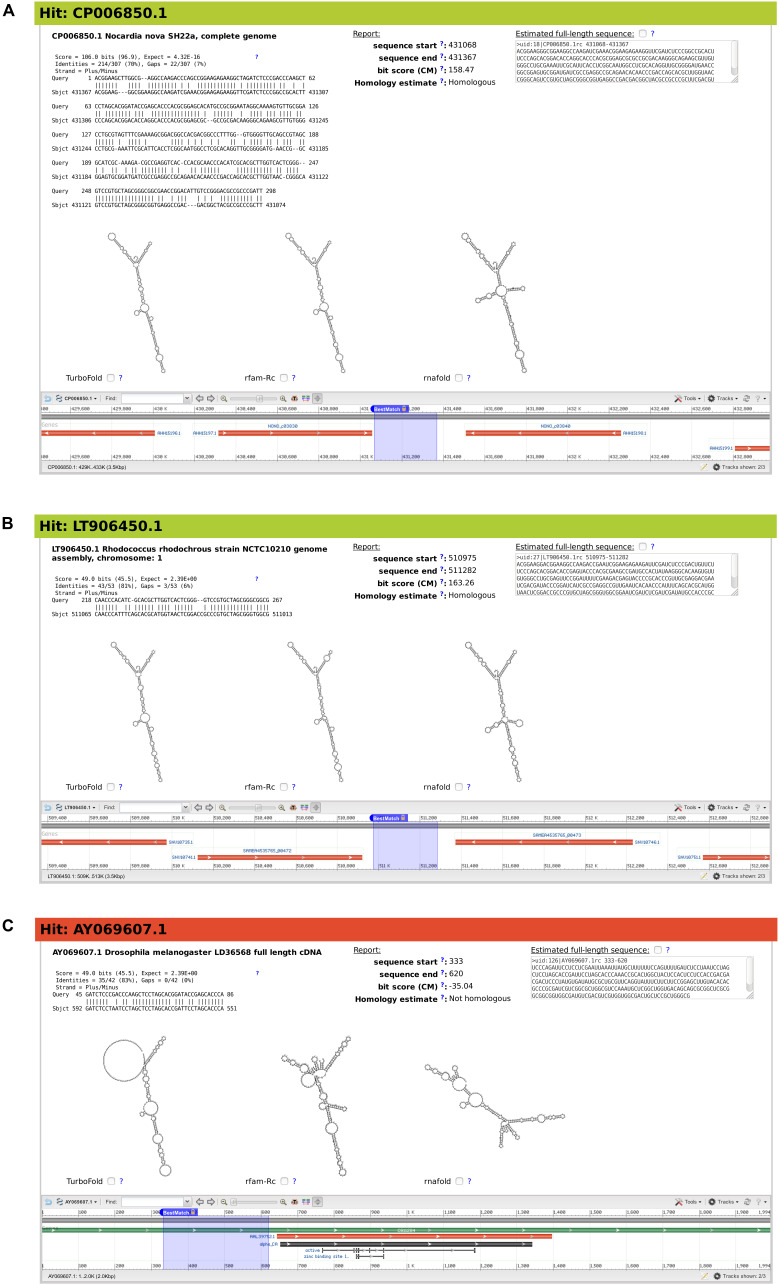
Snapshots of three example matches from output of BLAST search for *Mycobacterium smegmatis* ms1 RNA homologs, characterized by rboAnalyzer. Each of the three panels **(A–C)** contains from upper left corner to the right bottom corner following: ID and name of subject sequence, HSP with a partial match of the query sequence in the subject sequence, HSP characteristics, position of the full match of the subject RNA on a database subject sequence, homology of subject RNA identified by rboAnalyzer, full match sequence identified by rboAnalyzer by extension of HSP partial match, predicted secondary structures of subject RNA using its full match and depiction of the full match using NCBI genome browser.

**FIGURE 6:**
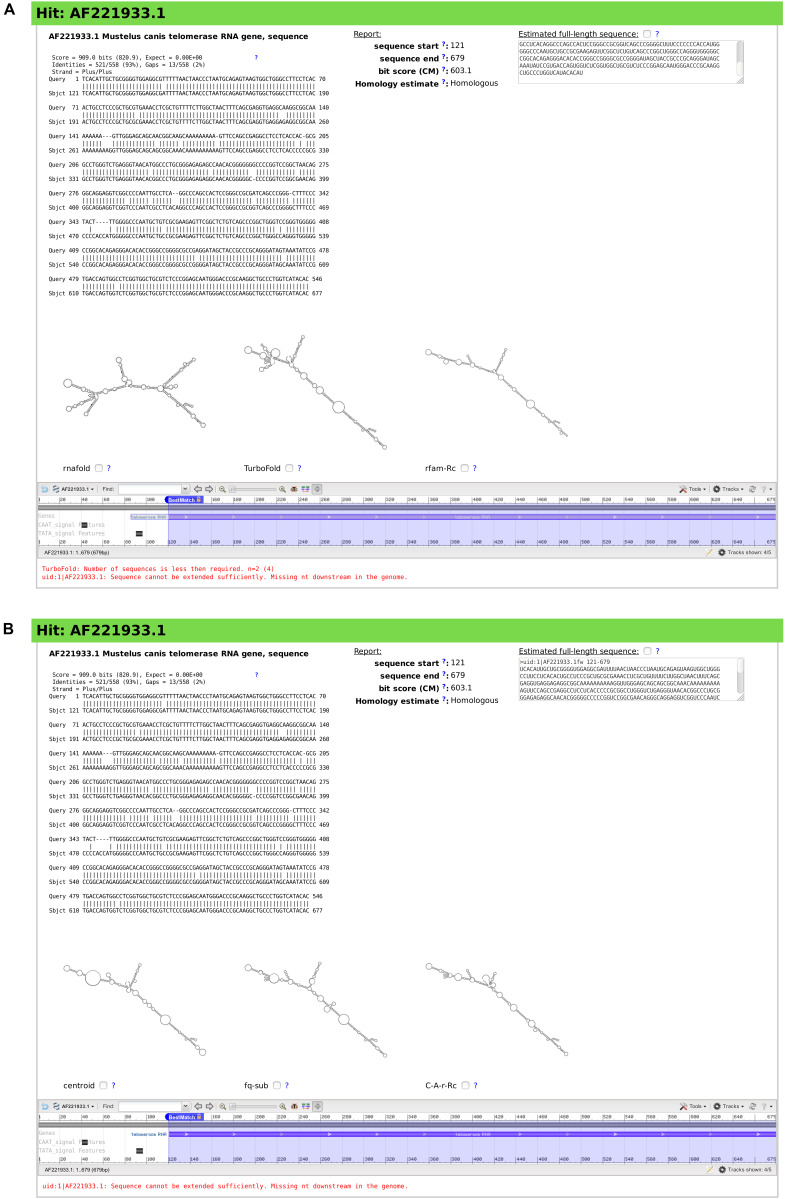
Snapshots of rboAnalyzer output for characterization of the output of BLAST search for *Rhizoprionodon porosus* TR homologs. For description of the items in the both panels **(A,B)**, see the legend of Figure 5.

**FIGURE 7:**
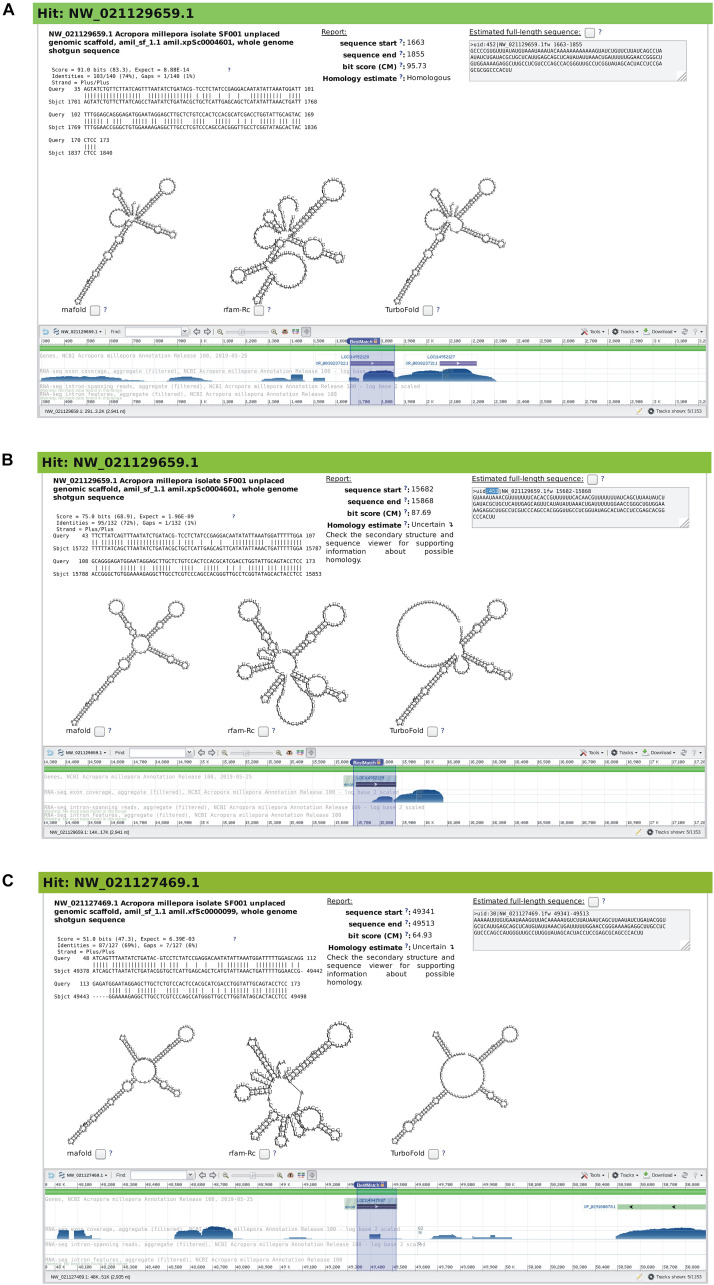
Snapshots of rboAnalyzer output for characterization of the output of BLAST output of a search for homologs of the human u2 RNA in *Cnidaria*. For description of the items in panels **(A–C)**, see the legend of Figure 5.

**FIGURE 8:**
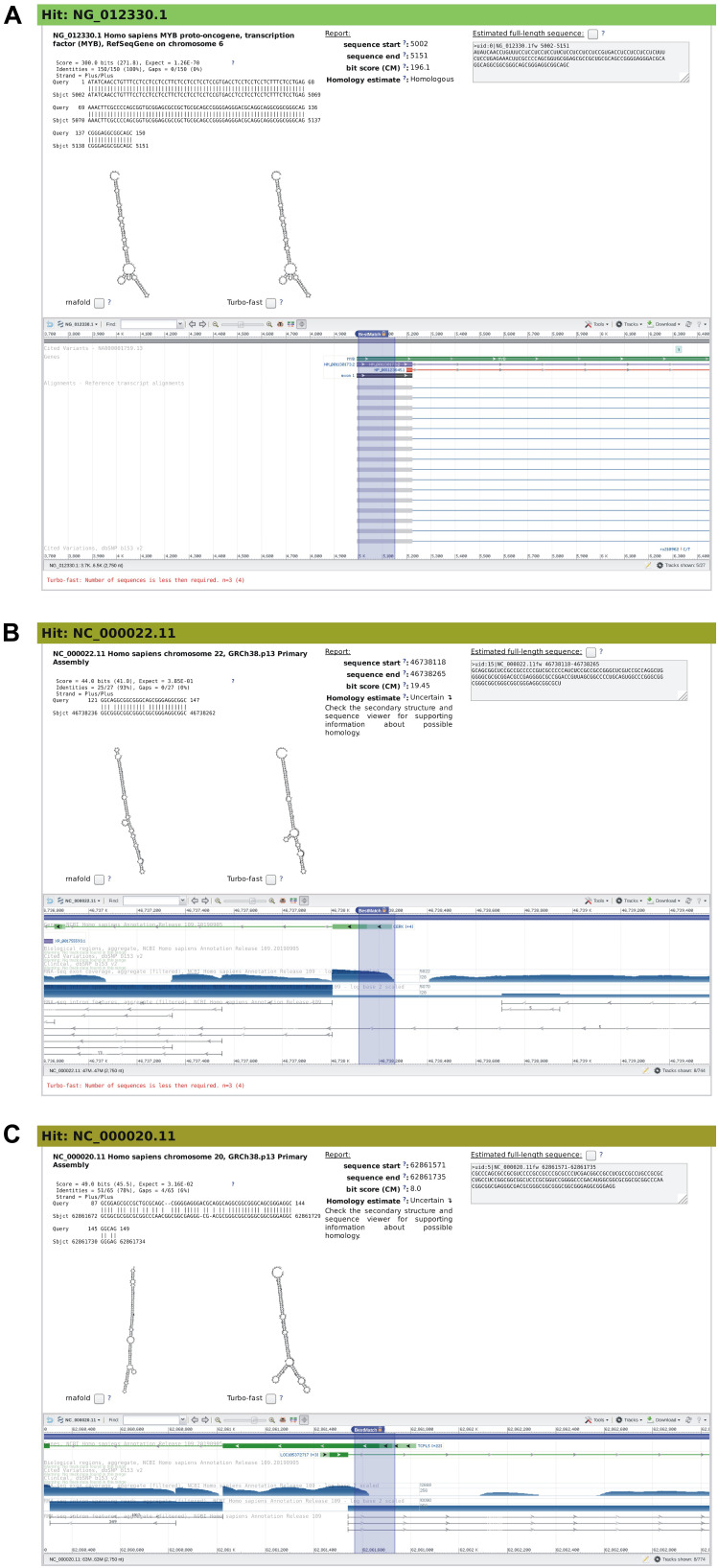
Snapshots of rboAnalyzer output for characterization of the output of BLAST search for the human MYB IRES homologs. For description of the items in panels **(A–C)**, see the legend of Figure 5.

We demonstrate the use of rboAnalyzer on four examples (Figures 5-8) for various scenarios of the rboAnalyzer use. The BLAST searches for rboAnalyzer demonstration were done at NCBI BLAST web server. The parameters of the searches are described in section “Results” of the Supplementary Material.

The number of HSPs in the BLAST outputs used for demonstration and time taken for the rboAnalyzer to run is shown in Table 2.

**TABLE 2:** Summary of the examples of rboAnalyzer usage.

RNA Family	HSPs	Time taken (min)	Time taken without TurboFold based method (min)	Supplementary file with full outputs
Ms1	146	23	2	MS1.zip
TR	89	38	11	TR.zip
U2	44	21	2	u2.zip
MYB IRES	41	2	0.5	MYB.zip

In the examples, most of the computation time was used by TurboFold or the Turbofold-based method TurboFast for prediction of secondary structures, so we also report running times without using TurboFast. The characterization of the TR BLAST output was relatively slow in spite of less number of characterized HSPs, as TR is relatively large, e.g., much larger than ms1 RNA from the first example. On the contrary, characterization of MYB IRES was relatively fast, as MYB IRES is a relatively short RNA.

### ms1 RNA

The first example demonstrates a common usage of rboAnalyzer; a characterization of HSPs in the output of BLAST search for the homologs of *Mycobacterium smegmatis* ms1 RNA in *Actinobacteria* (Figure 5). We chose three example HSPs for Figure 5, one with a high quality (BLAST *E*-value = 4.32 × 10^–16^; Figure 5A) and two with low qualities (both with BLAST *E*-values = 2.39; Figures 5B,C). The high quality HSP (Figure 5A) is an example of the ideal situation. The HSP covers a substantial part of the query sequence with relatively few gaps indicating strong similarity between sequences of query and subject RNAs and thus suggesting their homology, which indeed was identified by rboAnalyzer. The high sequence similarity led to an accurately extended full match that made it possible to predict accurate secondary structures that were best represented by Turbofold and rfam-Rc prediction in Figure 5A.

The remaining two HSPs, shown in Figures 5B,C, represented homologous and non-homologous RNAs, respectively. The homology was correctly identified by rboAnalyzer. Indeed, ms1 RNA is an exclusively bacterial RNA (Pánek et al., 2010) and the latter, non-homologous HSP (Figure 5C) was in eukaryotic species.

Further demonstrating capabilities of rboAnalyzer, the extended match for the HSP in Figure 5B was accurately estimated although the HSP was a short fragment that covered only 50 nucleotides out of ∼300 nucleotides of the query sequence with numerous gaps. Consequently, the secondary structure of the subject RNA was predicted correctly showing similarity to the ms1 RNA structure (here represented best by the predicted secondary structures in Figure 5A). The Turbofold prediction was more accurate than the RNAfold prediction (cf. predictions denoted Turbofold and rnafold in Figure 5B). It is because Turbofold uses multiple sequences, while RNAfold single sequences only. It is advantageous to use Turbofold here, as the multiple sequences of the extended matches of homologous RNAs found in a BLAST output can be used for its input.

The predicted secondary structures for the non-homologous HSP (Figure 5C) were inaccurate, i.e., dissimilar to the ms1 RNA secondary structure, as the subject sequence did not represent homologous RNA.

### TR (Telomerase RNA)

The second example (Figure 6) demonstrates the use of the alternative methods for secondary structure prediction implemented in rboAnalyzer. It uses BLAST search for *Rhizoprionodon porosus* TR homologs in vertebrates.

For the demonstration purposes we chose a hit into *Mustelus canis* TR evaluated by rboAnalyzer (Figure 6). The secondary structure of *Mustelus canis* TR predicted using default prediction methods in rboAnalyzer, i.e., RNAfold, TurboFold, and rfam-Rc, were dissimilar to each other (Figure 6A). The dissimilarity should give the user a clue that the result is not robust and needs further investigation.

In such an uncertain situation, the user can re-examine the HSP using alternative methods for secondary structure prediction and check if they produce similar secondary structures that will help to identify the RNA.

In this example, we chose centroid, fq-sub and C-A-r-Rc methods. They predicted similar secondary structures that were also similar to a reference, experimentally identified TR structure [found, e.g., in rPredictorDB database (Jelínek et al., 2019)].

The similarity obtained with the alternative methods suggested that the hit was a TR RNA homolog. If the structures were dissimilar again, it would be a clue that the hit most likely represented rather than a TR RNA homolog, a non-homologous RNA or non-physiological artifact RNA sequence.

### u2 RNA

The following two examples demonstrate the use of the information computed by rboAnalyzer to infer the homology of subject RNAs represented by their partial matches to the query RNA. The following example (Figure 7) is a characterization of BLAST output of a search for homologs of the human u2 RNA in *Cnidaria*. Figure 7 shows three HSPs of human u2 RNA in *Cnidaria* selected in the complete rboAnalyzer output with varying quality. The first HSP represented an u2 RNA homolog, while the homology to u2 RNA of the latter two HSPs (Figures 7B,C) was uncertain.

Here, to infer the homology, the user can use the predicted secondary structures obtained using full-length subject sequences reconstructed from the partial hits. In this case, the structures showed similarity to the predicted secondary structures of the u2 RNA homolog in Figure 7A, thus indicating that both HSPs may represent u2 RNA homologs. Also, the sequence viewer for the two uncertain HSPs, showed that the two HSPs belong to the RF00004 family (not shown) which is the u2 RNA Rfam family. All this evidence led to the conclusion that the two HSPs were indeed u2 homologs.

### MYB IRES

The fourth example (Figure 8) is a characterization of BLAST output of a sequence search for the human MYB IRES homologs. It is an example of a situation, when it is hard to determine the homology of subject RNAs represented by HSPs as the HSPs had a low quality and there were not enough good sequences in the BLAST output that would serve for secondary structure prediction, as indicated in red at the bottom of the HSPs sections in Figure 8.

Figure 8A shows a HSP of the query RNA, i.e., the human MYB IRES. The other two HPSs (Figures 8B,C) represented uncertain hits with low quality as indicated by BLAST *E*-values (0.385 and 0.0316) and CM bit scoring very low (8 and 19.45) compared to the *E*-value and CM bit score of the query RNA: 1.26 × 10^–70^ and 196.1, respectively. The two HSPs were short and strongly gapped. They also lacked their predicted secondary structure (predicted using the rfam-Rc prediction method), as MYB IRES is not a member of Rfam database and therefore lacks its Rfam CM model. Therefore the secondary structures were predicted only by the two remaining default prediction methods, RNAfold, and Turbofold. Finally, the sequence viewer did not provide any information that would be useful for determination of the homology of the two HSPs.

Nevertheless, there were two indications that these two HSPs might represent MYB IRES homologs: (1) the predicted secondary structures showed a partial similarity to the predicted secondary structure of the MYB IRES homolog shown in Figure 8A, (2) the genomic locus in the 5′ end of genes as shown by sequence viewer is in general the genomic locus of an IRES (for an example, see the sequence viewer in Figure 8A, which is annotated as “MYB”).

While it still remained questionable whether the two HSPs represented homologous RNAs, we see that even for such extremely bad HSPs rboAnalyzer could provide hints to possible homology of subject RNAs to the query RNA that otherwise, based merely on the information in the original BLAST output, could be missed.

## Discussion and Conclusion

We present rboAnalyzer, a tool for interpreting RNA sequence search outputs. It characterizes the hits in the outputs by prediction of their full-length sequences, homology to the query molecule and secondary structures. The tool is primarily aimed at non-coding RNA molecules, but can also be used with other RNAs that have defined structure (e.g., riboswitches).

In our opinion, the tool is needed, because only full-length sequences allow for effective analysis of RNAs in general. The prediction and analysis of secondary structure, homology and function identification is also essential, as RNAs function only with their full-length sequences. The partial and/or gapped matches in the output of sequence search usually come without any other information than their quality. The next step naturally is the identification of full-length sequences of the matched RNAs. So far, up to our knowledge, there is no other choice than to do it manually The presented tools facilitates this task by integrating appropriate tools to one framework with rich user control and results output.

By testing rboAnalyzer with BLAST outputs of varying quality we demonstrated that rboAnalyzer was able to give accurate secondary structure predictions even for HSPs that corresponded to short fragments and with low-quality HSPs.

Since running rboAnalyzer on typical BLAST results takes from several minutes to about an hour, depending on the number of HSPs, the length of the query RNA and chosen prediction methods, it is suitable for analyzing small number of BLAST outputs on a personal workstation, but requires cluster-scale computational resources for larger analyses.

rboAnalyzer is not suitable in situations, when subject RNAs include intronic RNA while the query RNA does not. It is because the difference between the length of the query sequence and the size of the genome locus containing the subject RNA with an intron makes it impossible to correctly extend the partial match of the subject RNA.

Currently, the rboAnalyzer webserver is being developed. Also a minimal version of rboAnalyzer fast enough to be able to analyze individual HSPs in real time is under preparation and will be included in the webserver.

rboAnalyzer is available under GPL 2.0 license.

## Data Availability Statement

The datasets generated for this study are available on request to the corresponding author.

## Author Contributions

JP: initiation and conception. MS: design, development, implementation, and testing. MS and JP: writing of the manuscript. MS, MM, JV, and JP: proofreading and discussion. All authors contributed to the article and approved the submitted version.

## Conflict of Interest

The authors declare that the research was conducted in the absence of any commercial or financial relationships that could be construed as a potential conflict of interest.

## Footnotes

1 https://palletsprojects.com/p/jinja/
