# Supplementary material for: rboAnalyzer: A Software to Improve Characterization of Non-coding RNAs From Sequence Database Search Output
Source: Front Genet. 2020 Jul 28;11:675. doi: 10.3389/fgene.2020.00675 (PMC7401326; doi:10.3389/fgene.2020.00675)
Supplement: Supplementary file 1 [file Data_Sheet_1.pdf]

Supplementary data
All experiments including timing of execution were done on Ubuntu 14.04 with CPU i7-5820K and 32 GB of RAM.

1. Optimization of rboAnalyzer parameters

The rboAnalyzer integrates several algorithms and uses few processing steps which depends on user chosen parameters. To determine the default values we did optimization for the parameters. The list of default parameters values with brief description follows.

1.1 Optimization of rboAnalyzer step i) - Estimation of full-length subject sequences, methods and parameters

Method "simple": no parameters are needed.

Method "locarna": In order to optimize the parameters for the extension of partial matches by locarna we've prepared artificial sequences constructed from known RNAs. These were sequences from Rfam (Nawrocki et al., 2015) database seed alignments of following families: RF00001, RF00002, RF00003, RF00013, RF00021, RF00022, RF00059, RF00169 and RF000622. The artificial sequence was constructed as follows: For each RNA sequence from the seed alignment we obtained up to 1000nt flanking regions (nucleotide sequences adjacent on 5' and 3' ends of the RNA) from NCBI (each sequence has unique accession number). If flanking region for the RNA was shorter then 1000nt, we used random sequence to fill the missing section up to 1000nts. The RNA sequence was then placed between obtained 1000nt sequences on 5' and 3' ends. Also the RNA sequence was shuffled 10 times and placed between the flanking regions to create decoys. This was done for all sequences from seed alignment for one RNA family. Finally all these sequences were joined to one sequence and BLAST database was build from it with `makeblastdb` program (Camacho et al., 2009). Then 3 RNA sequences from the seed alignment were chosen at random and were searched against the BLAST database (blastn program parameters: `-gapopen 2 -gapextend 1 -penalty -1 -reward 1 -word_size 7`) and 3 BLAST outputs were obtained for each RNA family. Then we executed the rboAnalyzer with varying parameter of interest.

For the evaluation of precision, we used only the full-length matches from the HSPs which localized to the known location of known RNAs. Because the location of RNA is known we can compute how precisely the extended full match is localized on the subject sequence from the BLAST database. As a measure of precision we take sum of number of bases by which the 5' and 3' end indexes differs from true RNA loci (defined as indexes for 5' and 3' ends), computed as follows: $err = |5'_{ext} - 5'_{ref}| + |3'_{ext} - 3'_{ref}|$, where $5'_{ext}$ and $3'_{ext}$ are indexes for the extended sequence and $5'_{ref}$ and $3'_{ref}$ are indexes for position of the reference sequence.

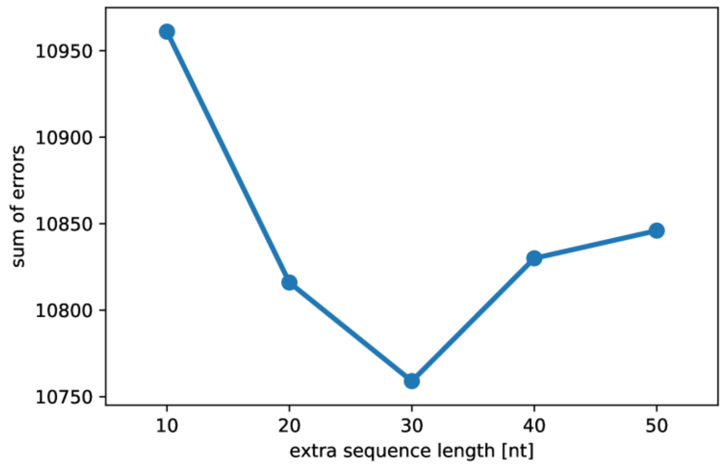

Supplementary figure 1: Sum of localization errors with changing the subseq_window_locarna parameter. The best setting was found to be 30 nt of extra sequence added to 5' and 3' ends when using Locarna alignment.

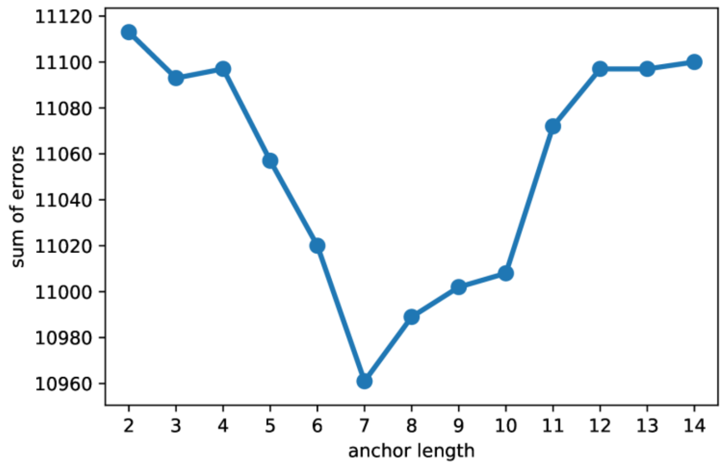

Supplementary figure 2: Sum of localization error with changing the locarna_anchor_lenght parameter. The best setting in our test was found to be 7.

Parameter `subseq_window_locarna 30` – controls the length of the extra flanking regions added on 5' and 3' ends of the full match obtained as with "simple" method.

Parameter `locarna_anchor_length 7` – controls minimal length of ungapped stretches of matches in HSP to be written as anchors for alignment by Locarna.

Locarna command-line parameter `--free_endgaps=++++` is used to set zero cost on gaps on 5' and 3' sides of the locarna alignment. Furthermore, the parameters `–struct-local=0` and `--sequ-local=0` are explicitly given to locarna while 0 (false) being their default value.

Mode "meta": no parameters other then the ones for "locarna" are needed.

1.2 Comparison of three methods for extending partial matches – Figure 3:

To evaluate the performance of methods for extending partial matches to full-length matches we've prepared dataset with RNA sequences at known positions in the chromosome. The dataset was prepared similary to the 1.1 – locarna section but with more RNA families from CompaRNA

database. We selected RNA families having at least one experimentally identified structure and at least 20 homologs in Rfam database (these were: RF00001, RF00002, RF00005, RF00008, RF00015, RF00017, RF00020, RF00095, RF00100, RF00162, RF00167, RF00169, RF00175, RF00209, RF00230, RF00250, RF00374, RF00379, RF00380, RF00480, RF01051, RF01725, RF01739, RF01807, RF01831, RF01852, RF02095, RF02253, RF02348). For each family 3 RNA sequences were chosen randomly to be used as query sequences, the rest of the sequences was used to construct artificial chromosome. The artificial sequence was constructed as follows: For each RNA sequence from the seed alignment we obtained up to 1000nt flanking regions (nucleotide sequences adjacent on 5’ and 3’ ends of the RNA) from NCBI (each sequence has unique accession number). If flanking region for the RNA was shorter then 1000nt, we used random sequence to fill the missing section up to 1000nts. The RNA sequence was then placed between obtained 1000nt sequences on 5’ and 3’ ends. Also the RNA sequence was shuffled 10 times and placed between the flanking regions to create decoys. This was done for remaining sequences from seed alignment (minus the sequences chosen as query sequences for given RNA family) for one RNA family. Finally all these sequences were joined to one sequence and BLAST database was build from it.

The 3 RNA sequences set aside previously were then used to search the BLAST database to generate BLAST outputs (blastn program parameters: `-gapopen 2 -gapextend 1 -penalty -1 -reward 1 -word_size 7`) for each RNA family.

Supplementary Table S1: The sums of errors for extension methods and number of HSP

Extension method	Sum of errors [nt]	Exact estimate (0-1] error	Small error (1-5] errors	Large error >5 errors
None (HSP indices)	110106	547 (11%)	1485 (30%)	2971 (59%)
simple	13232	2492 (50%)	1415 (28%)	1096 (22%)
locarna	8819	3138 (63%)	1247 (25%)	618 (12%)
meta	8656	3157 (63%)	1236 (25%)	610 (12%)

1.3 Optimization of rboAnalyzer step ii) – Identification of homology of subject RNAs

We used the BraliBase III dataset (Freyhult et al., 2007) for the evaluation of RIBOSUM matrix choice on RSEARCH performance. The results were evaluated as area under ROC curve. Results were very similar for all the RIBOSUM arrays and the RIBOSUM65 was chosen.

1.4 List of flowcharts of secondary structure prediction methods implemented in rboAnalyzer

Table S2. List and flowcharts of secondary structure prediction methods implemented in rboAnalyzer.

method (category‖) — description

rnafold (2)

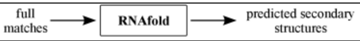

C-A-r-Rc (1) / M-A-r-Rc (1)

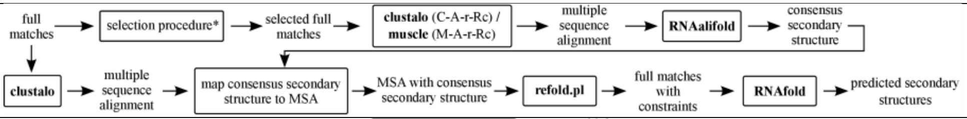

C-A-U-r-Rc (1) / M-A-U-r-Rc (1)

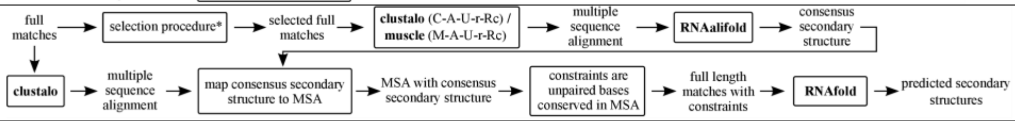

rfam-Rc (2)

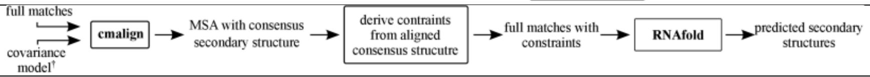

Method	Scheme
Rfam-centroid (2)	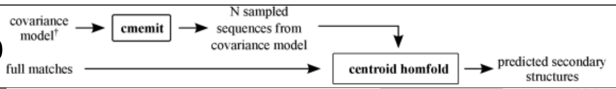
C-A-sub (1) / M-A-sub (1)	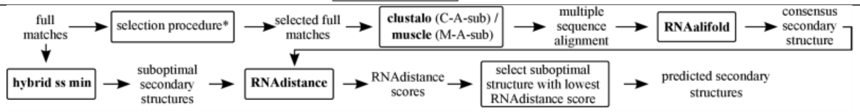
rfam-sub (2)	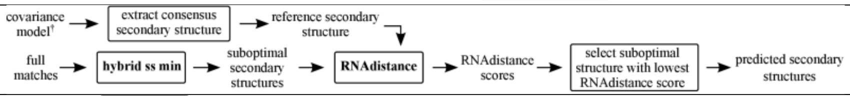
fq-sub (2)	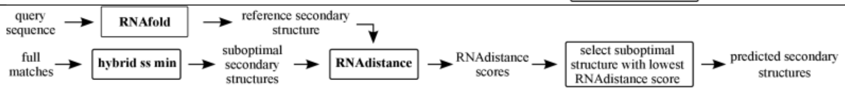
centroid (1) / centroid-fast (1)	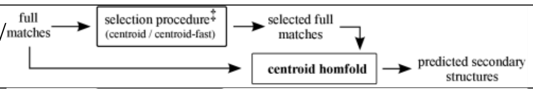
TurboFold (1) / Turbo-fast (1)	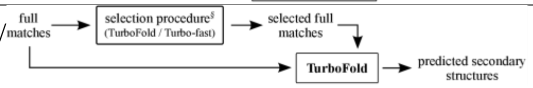

‖ The categories are defined in the *Step 3: Prediction of secondary structure of subject RNAs* section.

* full matches are filtered based on score from the rboAnalyzer step 2 (for description, see the “Step 2: Identification of homology of subject RNAs” subsection of this section) keeping only full matches scoring higher then threshold B, ratio between full match length and query sequence length keeping only full matches within ratio 1±R and full match sequence to sequence similarity to prevent populating selected full matches with too similar sequences keeping only those with similarity up to threshold S.

† covariance model obtained either from Rfam database or provided by the user.

‡ for “centroid” prediction method the selection procedure is same as in *, while for “centroid-fast” it is as follows: unique full matches are filtered based on ratio between full match length and query sequence length keeping only full matches within ratio 1±R, then first N full matches are selected keeping the original order from BLAST output.

§ for “TurboFold” prediction method the selection procedure starts same as in * and continues by taking up to N full matches keeping the original order from BLAST output, while for “Turbo-fast” it is as with “centroid-fast” prediction method.

The thresholds *B*, *R*, *S* and *N* were grid-searched for each prediction method separately and their values are listed in supplementary material section 1.5.

1.5 Optimization of rboAnalyzer step iii) – Prediction of secondary structure of subject RNAs, methods and parameters

Optimization of parameters for methods for prediction of secondary structures for full-length matches was done by comparing predicted secondary structures to reference RNA with known secondary structure. The RNAs with known secondary structures were obtained from CompaRNA dataset (Puton et al., 2013), the following families were used: RF00001, RF00002, RF00005, RF00008, RF00015, RF00017, RF00020, RF00162, RF00167, RF00169, RF00230, RF00379, RF00380, RF00480, RF01051, RF01725, RF01739, RF01831, RF01852, RF02253. For each RNA family from the CompaRNA dataset, up to 300 sequences from Rfam database seed alignment for the respective RNA family were obtained, 10 of these sequences were set aside to serve as query sequences and were not added to the database. For each sequence (from CompaRNA / Rfam) 10 randomized decoy sequences with preserved dinucleotide composition were computed using uShuffle (Jiang et al., 2008). The RNA sequences and the decoys were embedded in random sequence background and BLAST database was build for each family. For the optimization we used only 5 of the 10 prepared query sequences for each RNA family, the remaining 5 query sequences were used in the comparison of prediction methods. Each query sequence was searched with `blastn` program (parameters: -gapopen 2 -gapextend 1 -penalty -1 -reward 1 -word_size 7) in the database.

For the evaluation, only the predicted secondary structures for the full-length matches from the HSPs for the RNA with known secondary structure were used. Because of high number of optimized parameters, the figures were omitted from this supplementary material and are available upon request. The result of the optimization in form of default values for each prediction method is

listed below. For the description of the secondary structure prediction methods and their parameters please see the documentation available at https://github.com/cas-bioinf/rboAnalyzer.

List of default prediction parameters:

C-A-r-Rc:

- cmscore_percent: 30,
- pred_sim_threshold: 90,
- query_max_len_diff: 0.05

TurboFold:

- cmscore_percent: 20,
- query_max_len_diff: 0.15,
- max_seqs_in_prediction: 4

Turbo-fast:

- query_max_len_diff: 0.05,
- max_seqs_in_prediction: 4

rfam-sub:

- mfold: [10, 2, 80]

M-A-U-r-Rc:

- cmscore_percent: 40,
- pred_sim_threshold: 50,
- query_max_len_diff: 0.15,
- conseq_conserved: 3,
- repred_unpaired_tr: "*"

M-A-sub:

- cmscore_percent: 0,
- pred_sim_threshold: 50,
- query_max_len_diff: 0.1,
- mfold: [10, 2, 50]

fq-sub:

- mfold: [5, 2, 5]

C-A-sub:

- cmscore_percent: 0,
- pred_sim_threshold: 50,
- query_max_len_diff: 0.1,
- mfold: [5, 2, 50]

rfam-Rc:

- no parameters

M-A-r-Rc:

- cmscore_percent: 20,
- pred_sim_threshold: 90,
- query_max_len_diff: 0.05

centroid:

- centroid_homfold: "--engine_s CONTRAfold",
- cmscore_percent: 10,
- pred_sim_threshold: 70,
- query_max_len_diff: 0.15

C-A-U-r-Rc:

- cmscore_percent: 40,
- pred_sim_threshold: 50,
- query_max_len_diff: 0.1,
- conseq_conserved: 3,
- repred_unpaired_tr: "*"

rnafold:

- no parameters

centroid-fast:

- query_max_len_diff: 0.1,
- max_seqs_in_prediction: 3,

- centroid_homfold: "--engine_s CONTRAfold"

rfam-centroid:

- n_seqs: 9,
- centroid_homfold": "--engine_s McCaskill"

1.6 Comparison of methods for prediction of secondary structures from estimated full-length sequences:

The dataset was the same as it was for optimization of parameters for secondary structure prediction methods (supplementary section 1.4) with the remaining query sequences (the 5 prepared unused query sequences). Each query RNA was searched with `blastn` program (parameters: `-gapopen 2 -gapextend 1 -penalty -1 -reward 1 -word_size 7`) in the database. For the evaluation, only the predicted secondary structures for the full-length matches from the HSPs for the RNA with known secondary structure were used. As a measure of secondary structure similarity we used the RNAdistance score (Tafer et al., 2011) (tree edit distance) between the predicted secondary structure and known reference structure for the particular RNA. Then, for each extended full-length match, we did rank transform of the RNAdistance scores for all secondary structure prediction methods and then taken sum of achieved ranks for each secondary structure prediction method.

The best performing method was the rfam-Rc which uses consensus structure form Rfam covariance model as constraints for RNAFold. The next-best scoring methods is TurboFold, which is independent on the Rfam database and thus is more universal. As default we chose 3 prediction methods, the TurboFold and rfam-Rc methods for the prediction quality and RNAFold as universal reference.

Supplementary Table S3: Sum of ranks for prediction methods

prediction methods	Sum of ranks
rfam-Rc	2776.0
TurboFold	2968.0
Turbo-fast	2976.0
centroid	3266.0
rfam-sub	3334.5
C-A-r-Rc	3400.0
centroid-fast	3527.0
M-A-sub	3635.5
M-A-r-Rc	3721.0
C-A-sub	3820.0
M-A-U-r-Rc	3990.5
C-A-U-r-Rc	4275.5
fq-sub	4422.5
rfam-centroid	4441.5
rnafold	5351.0

2. Test of rboAnalyzer performance

The performance of rboAnalyzer was tested with BLAST outputs with different levels of quality. To test the influence of BLAST output quality we prepared dataset as follows: We obtained RNAs (query RNAs) with known reference structures from CompaRNA dataset for Rfam families having more then 20 homologs in the seed alignment (these were: RF00001, RF00002, RF00005, RF00008, RF00015, RF00017, RF00020, RF00095, RF00100, RF00162, RF00167, RF00169, RF00175, RF00209, RF00230, RF00250, RF00374, RF00379, RF00380, RF00480, RF01051, RF01725, RF01739, RF01807, RF01831, RF01852, RF02095, RF02253, RF02348). Then we obtained RNA sequences from Rfam seed alignment for each family, for each RNA sequence we downloaded whole parent sequence from NCBI and placed shuffled 500nt long sequence fragment on 5' and 3' sequence end. From these sequences we've build BLAST database. Each query RNA was searched with blastn program (parameters: `-gapopen 2 -gapextend 1 -penalty -1 -reward 1 -word_size 7`) in the database. Only the HSPs mapping to position of sequence from Rfam seed alignment was considered TP, all other HSPs are considered FP. The BLAST outputs were analyzed and the one with most true positive (TP) HSPs was used to produce synthetic BLAST outputs of different quality (high, moderate and low). The RNA families RF00095, RF00175, RF00230, RF01739 and RF01807 did not have enough TP HSPs to divide their BLAST outputs to the three parts and therefore were excluded from the analysis.

To make BLAST outputs with high, moderate and low quality HSPs we've computed two cut-off values C_{hm} and C_{ml} between high-moderate and moderate-low quality TP HSPs respectively. These cut-off values were computed as a mean of two adjacent HSP scores in first and second thirds of the ordered list of TP HSPs in each BLAST output (one for each RNA family, see Supplementary Table S3). Then we've divided each BLAST output to three parts using these cutoff values:

- low: HSPs $(0, C_{ml}]$
- moderate: HSPs $(C_{ml}, C_{hm}]$
- high: HSPs (C_{hm}, Inf).

Each prepared BLAST output was then analyzed by the rboAnalyzer (all prediction methods, default settings). Then RNAdistance score was computed for each predicted secondary structure for full-length match from TP HSP and respective reference structure. To compare between output qualities, RNA families and secondary structure prediction methods, we computed mean of RNAdistance scores for TP HSPs for each prediction method within each analyzed output. Results are summarized in Supplementary Figures 3 and 4.

Supplementary Table S3: List of cut-off score values C_{ml} and C_{hm} used to divide BLAST outputs used in the test. Each RNA family has their own cut-off values.

RNA family	low – moderate	moderate – high
RF00001	35	46
RF00002	47.5	60.5
RF00005	26	34
RF00008	24	26
RF00015	32	39.5
RF00017	65.5	262
RF00020	25	27.5
RF00100	30	34
RF00162	31	37
RF00167	22	29
RF00169	24	27
RF00209	178	211
RF00250	21	23
RF00374	38	58
RF00379	25	29
RF00380	40	65
RF00480	28	33
RF01051	29.5	35
RF01725	27	29
RF01831	24.5	37
RF01852	78.5	85.5
RF02095	35.5	51
RF02253	23	26
RF02348	40	48.5

Supplementary Figure 3: Influence of BLAST output quality on secondary structure prediction by prediction method for all methods for extension of partial matches

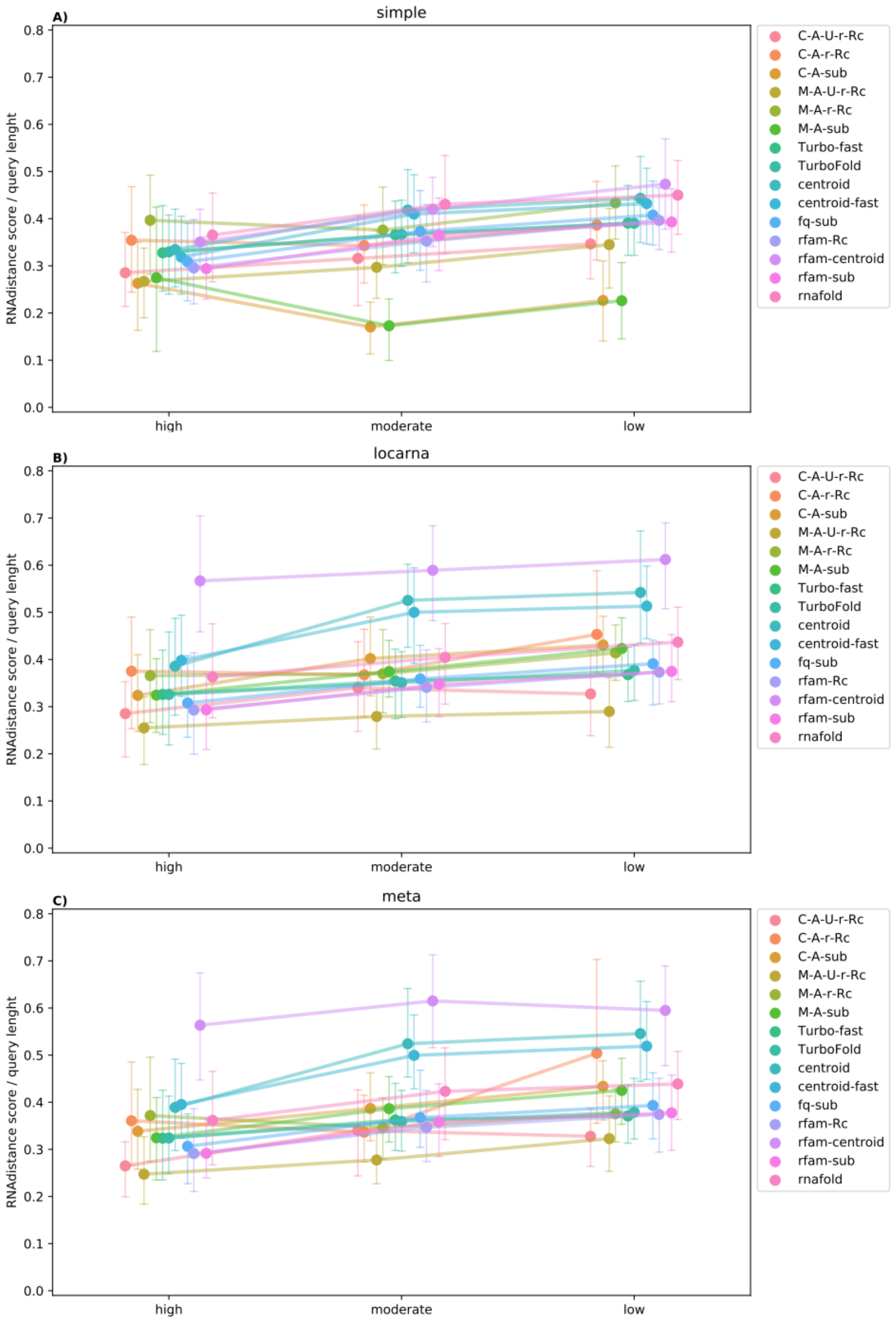
A)
simple
B)
locarna
C)
meta
RNAdistance score / query lenght
high
moderate
low
C-A-U-r-Rc
C-A-r-Rc
C-A-sub
M-A-U-r-Rc
M-A-r-Rc
M-A-sub
Turbo-fast
TurboFold
centroid
centroid-fast
fq-sub
rfam-Rc
rfam-centroid
rfam-sub
rnafold

Supplementary Figure 3: Influence of BLAST output quality on secondary structure prediction by for different secondary structure prediction methods and methods for extension of partial matches: A) extension method “simple”, B) extension method “locarna” and C) extension method “meta”. We show mean of normalized RNAdistance score RNA family means. The normalization of RNAdistance score was done by dividing the score by length of respective query sequences. The error bars represent 95% of 100 bootstrap iterations.

Supplementary Figure 4: Influence of BLAST output quality on prediction of secondary structure by RNA family for all methods for estimation of full-length sequence

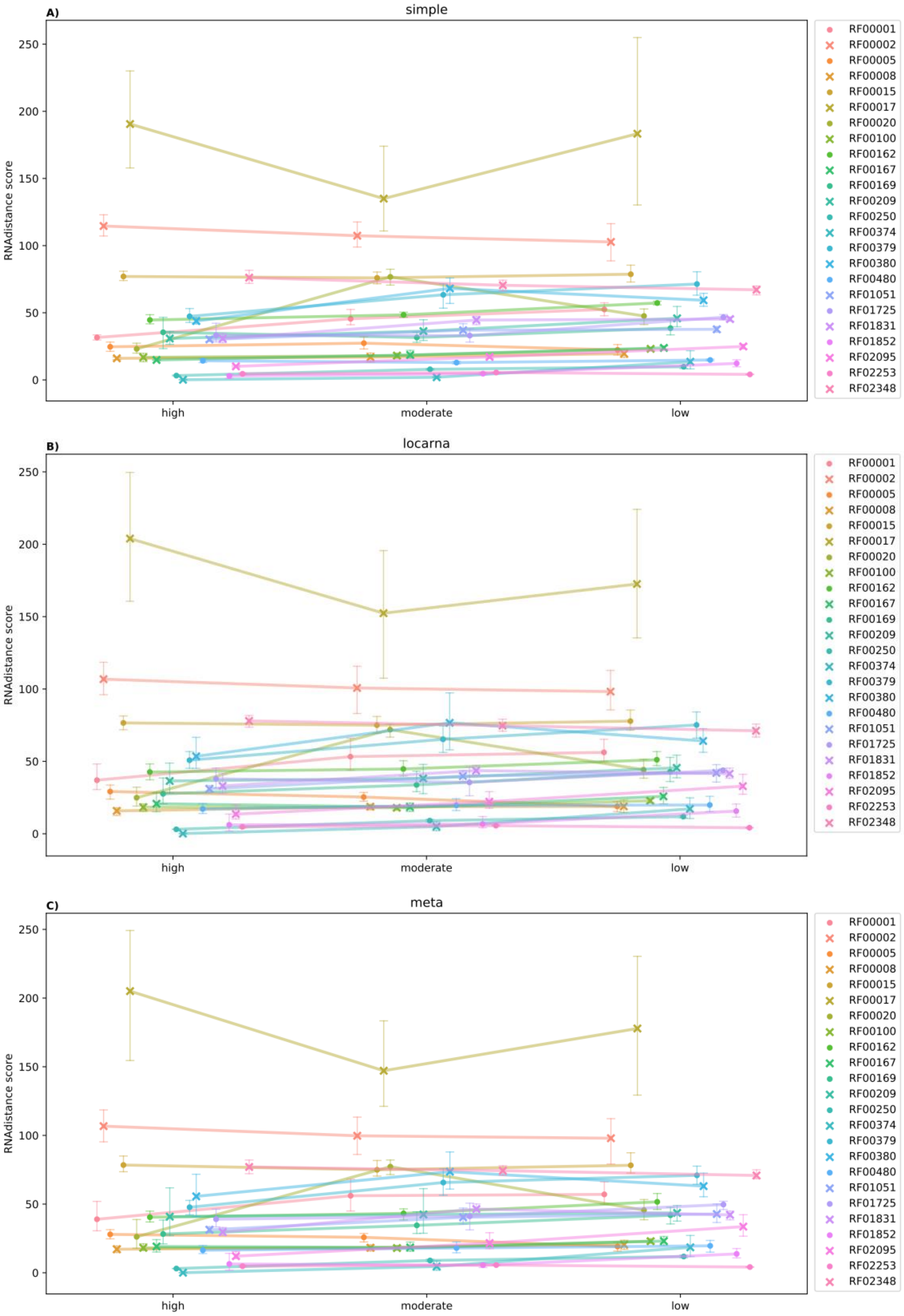

Supplementary Figure 4: Influence of BLAST output quality on secondary structure prediction for different RNA families and method for extension of partial mathches: A) extension method “simple”, B) extension method “locarna” and C) extension method “meta”. We show mean for RNAdistance score RNA family means. The error bars represent 95% of 100 bootstrap iterations.

3. Parameters of BLAST searches for examples in the “*Example usage of rboAnalyzer*” section

All searches were done via NCBI BLAST web-server with default parameters, if it is not described otherwise in the following.

3.1 ms1 RNA

Streptomycetaceae and *Mycobacteriaceae* classes were excluded from the search to avoid identical and very similar matches. The maximum number of matches was set to 10000. The searched database was “Nucleotide collection (nr/nt)”.

3.2 Telomerase RNA

The search was limited to *Vertebrae* and the maximum number of matches was set to 1000. The searched database was “Nucleotide collection (nr/nt)”.

3.3 u2 RNA and MYB IRES

The search was done using discontiguous megablast limited to *Eukarya* in “Whole-genome shotgun contigs (wgs).

References:

Camacho, C., Coulouris, G., Avagyan, V., Ma, N., Papadopoulos, J., Bealer, K., et al. (2009). BLAST plus: architecture and applications. *BMC Bioinformatics* 10, 1. doi:Artn 421\nDoi 10.1186/1471-2105-10-421.

Freyhult, E. K., Bollback, J. P., and Gardner, P. P. (2007). Exploring genomic dark matter: A critical assessment of the performance of homology search methods on noncoding RNA. *Genome Res.* 17, 117–125. doi:10.1101/gr.5890907.

Jiang, M., Anderson, J., Gillespie, J., and Mayne, M. (2008). uShuffle: A useful tool for shuffling biological sequences while preserving the k-let counts. *BMC Bioinformatics* 9, 192. doi:10.1186/1471-2105-9-192.

Nawrocki, E. P., Burge, S. W., Bateman, A., Daub, J., Eberhardt, R. Y., Eddy, S. R., et al. (2015). Rfam 12.0: updates to the RNA families database. *Nucleic Acids Res.* 43, D130-7. doi:10.1093/nar/gku1063.

Puton, T., Kozlowski, L. P., Rother, K. M., and Bujnicki, J. M. (2013). CompaRNA: a server for continuous benchmarking of automated methods for RNA secondary structure prediction. *Nucleic Acids Res.* 41, 4307–4323. doi:10.1093/nar/gkt101.

Tafer, H., Höner zu Siederdissen, C., Stadler, P. F., Bernhart, S. H., Hofacker, I. L., Lorenz, R., et al. (2011). ViennaRNA Package 2.0. *Algorithms Mol. Biol.* 6, 26. doi:10.1186/1748-7188-6-26.
