# Supplementary material for: rboAnalyzer: A Software to Improve Characterization of Non-coding RNAs From Sequence Database Search Output
Source: Front Genet. 2020 Jul 28;11:675. doi: 10.3389/fgene.2020.00675 (PMC7401326; doi:10.3389/fgene.2020.00675)
Supplement: Supplementary file 2 [file Data_Sheet_1.ZIP › MS1/MS1_rboAnalyzer_output.html]

 
 
 
     
     rboAnalyzer 
     
 
 

    
     
    

     
 
     
         Please wait while sequence viewer is loading... 
         
              
         
     
     
         
              -->
             rboAnalyzer report 
            
 
 BLAST output file:   /media/big-user/data-raid/clanek_rbo_analyzer/examples/MS1_def/PWWDWZTJ014-Alignment.xml
Query sequence file: /media/big-user/data-raid/clanek_rbo_analyzer/examples/MS1_def/MS1_query.fasta

RFAM model with best score to a query sequence     ?  Infered from query sequence by cmscan program.  
Family name: Ms1
E-value:     1.2e-42
 
 


         
         
            
    


    
     
         
            Hit: AF092842.1
         
         
             
                 
                     AF092842.1 Mycobacterium marinum G13 promoter construct 
                     
                         
 ?  
This is BLAST alignment as read from the input file 
                         
 Score = 185.0 bits (168.1), Expect = 2.91E-37
 Identities = 190/246 (77%), Gaps = 8/246 (3%)
 Strand = Plus/Plus
Query   1 ACGGAAGCTTGGCGAGGCCAAGACCCAGCCGGAAGAGAAGGCTAGATCTCCCGACCCAAGCTCCTAGCAC 70 
          |||| |||  || || ||||||  | | ||||||||||||| | |  ||||||||||  || || |||||    
Sbjct 213 ACGGCAGCCCGGTGAAGCCAAGGTCGAACCGGAAGAGAAGGTTCGTCCTCCCGACCCGGGCACCCAGCAC 282

Query  71 GGATACCGAGCACCCACGCGGAG-CACATGCCGCGGAATAGGCAAAAGTGTTGCGGACCTGCGTAGTTTC 139
          ||   ||| | |||||||||||| || | ||||||  |  |||| ||||||||||| |||||||| || |    
Sbjct 283 GGCCCCCG-GAACCCACGCGGAGTCATA-GCCGCGATAATGGCAGAAGTGTTGCGGGCCTGCGTAATTGC 350

Query 140 GAAAAGCGGACGGCCACGACGGCCCTTTGGGTGGGGTTGCAGCC-GTAGCGCATCGCAAAGACGCCGAGG 208
          ||||  | || ||   || ||||||||||||||||| ||||||| | || |  |||||||  ||||||||    
Sbjct 351 GAAATTCAGATGGTGCCGGCGGCCCTTTGGGTGGGGCTGCAGCCAGAAGGG--TCGCAAAAGCGCCGAGG 418

Query 209 TC-ACCCACGCAACCCAC-ATCGCACGCTTGGTCAC 242
           | ||||||||| ||||| |  ||||||||||| ||    
Sbjct 419 CCAACCCACGCAGCCCACAAATGCACGCTTGGTAAC 454

 
                 
                
                 
                      Report:  
                     
                         
                             sequence start
                                   ?  
                                     
Start position of the estimated full-length sequence in genome.
Start index  
                                 :
                             
                             213 
                         
                         
                             sequence end
                                   ?  
                                     
End position of the estimated full-length sequence in genome.
Start index  
                                 :
                             
                             514 
                         
                         
                             bit score (CM)
                                   ?  
                                     
The score for aligning estimated full-length sequence to CM model
  (computed by RSEARCH -> default,
  infered from Rfam or provided by user) 
                                 :
                             
                             160.08 
                         
                         
                             Homology estimate
                                   ?  
                                     
Quick homology estimate:
  Not homologous: bit score   20 and bit score > 0.5 * query length
  Uncertain otherwise 
                                 :
                             
                             Homologous 
                         
                     
                 
                
                
                 
                     
                          Estimated full-length sequence:  
                         
                          ? 
                             
Click checkbox to select multiple seuqences.
Fasta header format:
  UID|accession.versionSTRAND start-end 
                         
                     
                     >uid:0|AF092842.1fw 213-514
ACGGCAGCCCGGUGAAGCCAAGGUCGAACCGGAAGAGAAGGUUCGUCCUCCCGACCCGGG
CACCCAGCACGGCCCCCGGAACCCACGCGGAGUCAUAGCCGCGAUAAUGGCAGAAGUGUU
GCGGGCCUGCGUAAUUGCGAAAUUCAGAUGGUGCCGGCGGCCCUUUGGGUGGGGCUGCAG
CCAGAAGGGUCGCAAAAGCGCCGAGGCCAACCCACGCAGCCCACAAAUGCACGCUUGGUA
ACCGAGAUCCCCCGGGCUGCAGGAAUUCGAUAUCAAGCUUAUCGAUACCGUCGACCUCGA
GG
 
                 
                
             
            
             
                
                     
                         
                         
                             TurboFold 
                             
                              ? 
                                 
Visualisation of predicted secondary structure.
To save the image:
  Right click on the image -> Save Image as. 
                             
                         
                     
                
                     
                         
                         
                             rfam-Rc 
                             
                              ? 
                                 
Visualisation of predicted secondary structure.
To save the image:
  Right click on the image -> Save Image as. 
                             
                         
                     
                
                     
                         
                         
                             rnafold 
                             
                              ? 
                                 
Visualisation of predicted secondary structure.
To save the image:
  Right click on the image -> Save Image as. 
                             
                         
                     
                
             
            

        
             
                 Load Sequence viewer 
             
        
        
         
     
    

    
     
         
            Hit: CP038030.1
         
         
             
                 
                     CP038030.1 Rhodococcus ruber strain R1 chromosome, complete genome 
                     
                         
 ?  
This is BLAST alignment as read from the input file 
                         
 Score = 128.0 bits (116.7), Expect = 4.62E-22
 Identities = 209/295 (71%), Gaps = 18/295 (6%)
 Strand = Plus/Minus
Query     15 AGGCCAAGACCCAGCCGGAAGAGAAGGCTAGATCTCCCGACCCAAGCTCCTAGCACGGATACCG 78    
             ||||||||| | |  |||||||||||| | ||||||||||||  |  ||| |||||||| ||||       
Sbjct 539528 AGGCCAAGAGCGAATCGGAAGAGAAGGTTCGATCTCCCGACCGGACTTCCCAGCACGGACACCG 539465

Query     79 AGCACCCACGCGGAGCACATGCCGCGGAATAGGCAAAAGTGTTGCGGACCTGCGTAGTTTCGAA 142   
             ||||||||||||||||  ||||| |   |  |||||||| |||| || | |||| || ||||         
Sbjct 539464 AGCACCCACGCGGAGCCGATGCCACTATAAGGGCAAAAGCGTTGTGGGCTTGCG-AGATTCGGG 539402

Query    143 AAGCGGACGGC---CACGACG-GCCCTTTGGGTGG-GGTTGCAGCCGTAGCGCATCGCAAAGAC 201   
                  ||||||   |||  ||  ||| ||| | ||  |   ||||  || |    || |    |       
Sbjct 539401 TCTTCGACGGCGAGCACCCCGCACCCGTTGCGAGGACGAACCAGCGATATC----CGAACCCTC 539342

Query    202 GCCGAGGTCA--CCCACGCAACCCACATCGCACGCTTGGTCACTCGG-GGTCCGTGCTAGCGGG 262   
             ||||||| ||    ||| |||||||    ||||||||||| ||||||  ||||||||| |||||       
Sbjct 539341 GCCGAGGCCAGTGACACACAACCCATGCAGCACGCTTGGTAACTCGGCAGTCCGTGCTGGCGGG 539278

Query    263 CGGCG-AACCG--GACATTGTCCGGGACGCCGCCCGATT 298   
             || ||  ||||  | |||  || | |||| |||||| ||       
Sbjct 539277 CGACGTCACCGAAGTCAT--TCGGCGACGTCGCCCGCTT 539241

 
                 
                
                 
                      Report:  
                     
                         
                             sequence start
                                   ?  
                                     
Start position of the estimated full-length sequence in genome.
Start index  
                                 :
                             
                             539235 
                         
                         
                             sequence end
                                   ?  
                                     
End position of the estimated full-length sequence in genome.
Start index  
                                 :
                             
                             539542 
                         
                         
                             bit score (CM)
                                   ?  
                                     
The score for aligning estimated full-length sequence to CM model
  (computed by RSEARCH -> default,
  infered from Rfam or provided by user) 
                                 :
                             
                             165.94 
                         
                         
                             Homology estimate
                                   ?  
                                     
Quick homology estimate:
  Not homologous: bit score   20 and bit score > 0.5 * query length
  Uncertain otherwise 
                                 :
                             
                             Homologous 
                         
                     
                 
                
                
                 
                     
                          Estimated full-length sequence:  
                         
                          ? 
                             
Click checkbox to select multiple seuqences.
Fasta header format:
  UID|accession.versionSTRAND start-end 
                         
                     
                     >uid:1|CP038030.1rc 539235-539542
UACGGAAGGACGGAAGGCCAAGAGCGAAUCGGAAGAGAAGGUUCGAUCUCCCGACCGGAC
UUCCCAGCACGGACACCGAGCACCCACGCGGAGCCGAUGCCACUAUAAGGGCAAAAGCGU
UGUGGGCUUGCGAGAUUCGGGUCUUCGACGGCGAGCACCCCGCACCCGUUGCGAGGACGA
ACCAGCGAUAUCCGAACCCUCGCCGAGGCCAGUGACACACAACCCAUGCAGCACGCUUGG
UAACUCGGCAGUCCGUGCUGGCGGGCGACGUCACCGAAGUCAUUCGGCGACGUCGCCCGC
UUACAUGU
 
                 
                
             
            
             
                
                     
                         
                         
                             TurboFold 
                             
                              ? 
                                 
Visualisation of predicted secondary structure.
To save the image:
  Right click on the image -> Save Image as. 
                             
                         
                     
                
                     
                         
                         
                             rfam-Rc 
                             
                              ? 
                                 
Visualisation of predicted secondary structure.
To save the image:
  Right click on the image -> Save Image as. 
                             
                         
                     
                
                     
                         
                         
                             rnafold 
                             
                              ? 
                                 
Visualisation of predicted secondary structure.
To save the image:
  Right click on the image -> Save Image as. 
                             
                         
                     
                
             
            

        
             
                 Load Sequence viewer 
             
        
        
         
     
    

    
     
         
            Hit: CP023714.1
         
         
             
                 
                     CP023714.1 Rhodococcus ruber strain YC-YT1 chromosome, complete genome 
                     
                         
 ?  
This is BLAST alignment as read from the input file 
                         
 Score = 128.0 bits (116.7), Expect = 4.62E-22
 Identities = 209/295 (71%), Gaps = 18/295 (6%)
 Strand = Plus/Minus
Query     15 AGGCCAAGACCCAGCCGGAAGAGAAGGCTAGATCTCCCGACCCAAGCTCCTAGCACGGATACCG 78    
             ||||||||| | |  |||||||||||| | ||||||||||||  |  ||| |||||||| ||||       
Sbjct 760366 AGGCCAAGAGCGAATCGGAAGAGAAGGTTCGATCTCCCGACCGGACTTCCCAGCACGGACACCG 760303

Query     79 AGCACCCACGCGGAGCACATGCCGCGGAATAGGCAAAAGTGTTGCGGACCTGCGTAGTTTCGAA 142   
             ||||||||||||||||  ||||| |   |  |||||||| |||| || | |||| || ||||         
Sbjct 760302 AGCACCCACGCGGAGCCGATGCCACTATAAGGGCAAAAGCGTTGTGGGCTTGCG-AGATTCGGG 760240

Query    143 AAGCGGACGGC---CACGACG-GCCCTTTGGGTGG-GGTTGCAGCCGTAGCGCATCGCAAAGAC 201   
                  ||||||   |||  ||  ||| ||| | ||  |   ||||  || |    || |    |       
Sbjct 760239 TCTTCGACGGCGAGCACCCCGCACCCGTTGCGAGGACGAACCAGCGATATC----CGAACCCTC 760180

Query    202 GCCGAGGTCA--CCCACGCAACCCACATCGCACGCTTGGTCACTCGG-GGTCCGTGCTAGCGGG 262   
             ||||||| ||    ||| |||||||    ||||||||||| ||||||  ||||||||| |||||       
Sbjct 760179 GCCGAGGCCAGTGACACACAACCCATGCAGCACGCTTGGTAACTCGGCAGTCCGTGCTGGCGGG 760116

Query    263 CGGCG-AACCG--GACATTGTCCGGGACGCCGCCCGATT 298   
             || ||  ||||  | |||  || | |||| |||||| ||       
Sbjct 760115 CGACGCCACCGAAGTCAT--TCGGCGACGTCGCCCGCTT 760079

 
                 
                
                 
                      Report:  
                     
                         
                             sequence start
                                   ?  
                                     
Start position of the estimated full-length sequence in genome.
Start index  
                                 :
                             
                             760073 
                         
                         
                             sequence end
                                   ?  
                                     
End position of the estimated full-length sequence in genome.
Start index  
                                 :
                             
                             760380 
                         
                         
                             bit score (CM)
                                   ?  
                                     
The score for aligning estimated full-length sequence to CM model
  (computed by RSEARCH -> default,
  infered from Rfam or provided by user) 
                                 :
                             
                             165.64 
                         
                         
                             Homology estimate
                                   ?  
                                     
Quick homology estimate:
  Not homologous: bit score   20 and bit score > 0.5 * query length
  Uncertain otherwise 
                                 :
                             
                             Homologous 
                         
                     
                 
                
                
                 
                     
                          Estimated full-length sequence:  
                         
                          ? 
                             
Click checkbox to select multiple seuqences.
Fasta header format:
  UID|accession.versionSTRAND start-end 
                         
                     
                     >uid:2|CP023714.1rc 760073-760380
UACGGAAGGACGGAAGGCCAAGAGCGAAUCGGAAGAGAAGGUUCGAUCUCCCGACCGGAC
UUCCCAGCACGGACACCGAGCACCCACGCGGAGCCGAUGCCACUAUAAGGGCAAAAGCGU
UGUGGGCUUGCGAGAUUCGGGUCUUCGACGGCGAGCACCCCGCACCCGUUGCGAGGACGA
ACCAGCGAUAUCCGAACCCUCGCCGAGGCCAGUGACACACAACCCAUGCAGCACGCUUGG
UAACUCGGCAGUCCGUGCUGGCGGGCGACGCCACCGAAGUCAUUCGGCGACGUCGCCCGC
UUACAUGU
 
                 
                
             
            
             
                
                     
                         
                         
                             TurboFold 
                             
                              ? 
                                 
Visualisation of predicted secondary structure.
To save the image:
  Right click on the image -> Save Image as. 
                             
                         
                     
                
                     
                         
                         
                             rfam-Rc 
                             
                              ? 
                                 
Visualisation of predicted secondary structure.
To save the image:
  Right click on the image -> Save Image as. 
                             
                         
                     
                
                     
                         
                         
                             rnafold 
                             
                              ? 
                                 
Visualisation of predicted secondary structure.
To save the image:
  Right click on the image -> Save Image as. 
                             
                         
                     
                
             
            

        
             
                 Load Sequence viewer 
             
        
        
         
     
    

    
     
         
            Hit: CP029146.1
         
         
             
                 
                     CP029146.1 Rhodococcus ruber strain SD3 chromosome, complete genome 
                     
                         
 ?  
This is BLAST alignment as read from the input file 
                         
 Score = 128.0 bits (116.7), Expect = 4.62E-22
 Identities = 209/295 (71%), Gaps = 18/295 (6%)
 Strand = Plus/Minus
Query     15 AGGCCAAGACCCAGCCGGAAGAGAAGGCTAGATCTCCCGACCCAAGCTCCTAGCACGGATACCG 78    
             ||||||||| | |  |||||||||||| | ||||||||||||  |  ||| |||||||| ||||       
Sbjct 538220 AGGCCAAGAGCGAATCGGAAGAGAAGGTTCGATCTCCCGACCGGACTTCCCAGCACGGACACCG 538157

Query     79 AGCACCCACGCGGAGCACATGCCGCGGAATAGGCAAAAGTGTTGCGGACCTGCGTAGTTTCGAA 142   
             ||||||||||||||||  ||||| |   |  |||||||| |||| || | |||| || ||||         
Sbjct 538156 AGCACCCACGCGGAGCCGATGCCACTATAAGGGCAAAAGCGTTGTGGGCTTGCG-AGATTCGGG 538094

Query    143 AAGCGGACGGC---CACGACG-GCCCTTTGGGTGG-GGTTGCAGCCGTAGCGCATCGCAAAGAC 201   
                  ||||||   |||  ||  ||| ||| | ||  |   ||||  || |    || |    |       
Sbjct 538093 TCTTCGACGGCGAGCACCCCGCACCCGTTGCGAGGACGAACCAGCGATATC----CGAACCCTC 538034

Query    202 GCCGAGGTCA--CCCACGCAACCCACATCGCACGCTTGGTCACTCGG-GGTCCGTGCTAGCGGG 262   
             ||||||| ||    ||| |||||||    ||||||||||| ||||||  ||||||||| |||||       
Sbjct 538033 GCCGAGGCCAGTGACACACAACCCATGCAGCACGCTTGGTAACTCGGCAGTCCGTGCTGGCGGG 537970

Query    263 CGGCG-AACCG--GACATTGTCCGGGACGCCGCCCGATT 298   
             || ||  ||||  | |||  || | |||| |||||| ||       
Sbjct 537969 CGACGCCACCGAAGTCAT--TCGGCGACGTCGCCCGCTT 537933

 
                 
                
                 
                      Report:  
                     
                         
                             sequence start
                                   ?  
                                     
Start position of the estimated full-length sequence in genome.
Start index  
                                 :
                             
                             537927 
                         
                         
                             sequence end
                                   ?  
                                     
End position of the estimated full-length sequence in genome.
Start index  
                                 :
                             
                             538234 
                         
                         
                             bit score (CM)
                                   ?  
                                     
The score for aligning estimated full-length sequence to CM model
  (computed by RSEARCH -> default,
  infered from Rfam or provided by user) 
                                 :
                             
                             165.64 
                         
                         
                             Homology estimate
                                   ?  
                                     
Quick homology estimate:
  Not homologous: bit score   20 and bit score > 0.5 * query length
  Uncertain otherwise 
                                 :
                             
                             Homologous 
                         
                     
                 
                
                
                 
                     
                          Estimated full-length sequence:  
                         
                          ? 
                             
Click checkbox to select multiple seuqences.
Fasta header format:
  UID|accession.versionSTRAND start-end 
                         
                     
                     >uid:3|CP029146.1rc 537927-538234
UACGGAAGGACGGAAGGCCAAGAGCGAAUCGGAAGAGAAGGUUCGAUCUCCCGACCGGAC
UUCCCAGCACGGACACCGAGCACCCACGCGGAGCCGAUGCCACUAUAAGGGCAAAAGCGU
UGUGGGCUUGCGAGAUUCGGGUCUUCGACGGCGAGCACCCCGCACCCGUUGCGAGGACGA
ACCAGCGAUAUCCGAACCCUCGCCGAGGCCAGUGACACACAACCCAUGCAGCACGCUUGG
UAACUCGGCAGUCCGUGCUGGCGGGCGACGCCACCGAAGUCAUUCGGCGACGUCGCCCGC
UUACAUGU
 
                 
                
             
            
             
                
                     
                         
                         
                             TurboFold 
                             
                              ? 
                                 
Visualisation of predicted secondary structure.
To save the image:
  Right click on the image -> Save Image as. 
                             
                         
                     
                
                     
                         
                         
                             rfam-Rc 
                             
                              ? 
                                 
Visualisation of predicted secondary structure.
To save the image:
  Right click on the image -> Save Image as. 
                             
                         
                     
                
                     
                         
                         
                             rnafold 
                             
                              ? 
                                 
Visualisation of predicted secondary structure.
To save the image:
  Right click on the image -> Save Image as. 
                             
                         
                     
                
             
            

        
             
                 Load Sequence viewer 
             
        
        
         
     
    

    
     
         
            Hit: CP024890.1
         
         
             
                 
                     CP024890.1 Rhodococcus ruber strain YYL chromosome, complete genome 
                     
                         
 ?  
This is BLAST alignment as read from the input file 
                         
 Score = 128.0 bits (116.7), Expect = 4.62E-22
 Identities = 209/295 (71%), Gaps = 18/295 (6%)
 Strand = Plus/Minus
Query      15 AGGCCAAGACCCAGCCGGAAGAGAAGGCTAGATCTCCCGACCCAAGCTCCTAGCACGGATAC 76     
              ||||||||| | |  |||||||||||| | ||||||||||||  |  ||| |||||||| ||        
Sbjct 5572362 AGGCCAAGAGCGAATCGGAAGAGAAGGTTCGATCTCCCGACCGGACTTCCCAGCACGGACAC 5572301

Query      77 CGAGCACCCACGCGGAGCACATGCCGCGGAATAGGCAAAAGTGTTGCGGACCTGCGTAGTTT 138    
              ||||||||||||||||||  ||||| |   |  |||||||| |||| || | |||| || ||        
Sbjct 5572300 CGAGCACCCACGCGGAGCCGATGCCACTATAAGGGCAAAAGCGTTGTGGGCTTGCG-AGATT 5572240

Query     139 CGAAAAGCGGACGGC---CACGACG-GCCCTTTGGGTGG-GGTTGCAGCCGTAGCGCATCGC 195    
              ||       ||||||   |||  ||  ||| ||| | ||  |   ||||  || |    ||         
Sbjct 5572239 CGGGTCTTCGACGGCGAGCACCCCGCACCCGTTGCGAGGACGAACCAGCGATATC----CGA 5572182

Query     196 AAAGACGCCGAGGTCA--CCCACGCAACCCACATCGCACGCTTGGTCACTCGG-GGTCCGTG 254    
              |    |||||||| ||    ||| |||||||    ||||||||||| ||||||  |||||||        
Sbjct 5572181 ACCCTCGCCGAGGCCAGTGACACACAACCCATGCAGCACGCTTGGTAACTCGGCAGTCCGTG 5572120

Query     255 CTAGCGGGCGGCG-AACCG--GACATTGTCCGGGACGCCGCCCGATT 298    
              || ||||||| ||  ||||  | |||  || | |||| |||||| ||        
Sbjct 5572119 CTGGCGGGCGACGCCACCGAAGTCAT--TCGGCGACGTCGCCCGCTT 5572075

 
                 
                
                 
                      Report:  
                     
                         
                             sequence start
                                   ?  
                                     
Start position of the estimated full-length sequence in genome.
Start index  
                                 :
                             
                             5572069 
                         
                         
                             sequence end
                                   ?  
                                     
End position of the estimated full-length sequence in genome.
Start index  
                                 :
                             
                             5572376 
                         
                         
                             bit score (CM)
                                   ?  
                                     
The score for aligning estimated full-length sequence to CM model
  (computed by RSEARCH -> default,
  infered from Rfam or provided by user) 
                                 :
                             
                             165.64 
                         
                         
                             Homology estimate
                                   ?  
                                     
Quick homology estimate:
  Not homologous: bit score   20 and bit score > 0.5 * query length
  Uncertain otherwise 
                                 :
                             
                             Homologous 
                         
                     
                 
                
                
                 
                     
                          Estimated full-length sequence:  
                         
                          ? 
                             
Click checkbox to select multiple seuqences.
Fasta header format:
  UID|accession.versionSTRAND start-end 
                         
                     
                     >uid:4|CP024890.1rc 5572069-5572376
UACGGAAGGACGGAAGGCCAAGAGCGAAUCGGAAGAGAAGGUUCGAUCUCCCGACCGGAC
UUCCCAGCACGGACACCGAGCACCCACGCGGAGCCGAUGCCACUAUAAGGGCAAAAGCGU
UGUGGGCUUGCGAGAUUCGGGUCUUCGACGGCGAGCACCCCGCACCCGUUGCGAGGACGA
ACCAGCGAUAUCCGAACCCUCGCCGAGGCCAGUGACACACAACCCAUGCAGCACGCUUGG
UAACUCGGCAGUCCGUGCUGGCGGGCGACGCCACCGAAGUCAUUCGGCGACGUCGCCCGC
UUACAUGU
 
                 
                
             
            
             
                
                     
                         
                         
                             TurboFold 
                             
                              ? 
                                 
Visualisation of predicted secondary structure.
To save the image:
  Right click on the image -> Save Image as. 
                             
                         
                     
                
                     
                         
                         
                             rfam-Rc 
                             
                              ? 
                                 
Visualisation of predicted secondary structure.
To save the image:
  Right click on the image -> Save Image as. 
                             
                         
                     
                
                     
                         
                         
                             rnafold 
                             
                              ? 
                                 
Visualisation of predicted secondary structure.
To save the image:
  Right click on the image -> Save Image as. 
                             
                         
                     
                
             
            

        
             
                 Load Sequence viewer 
             
        
        
         
     
    

    
     
         
            Hit: CP024315.1
         
         
             
                 
                     CP024315.1 Rhodococcus ruber strain P14 chromosome, complete genome 
                     
                         
 ?  
This is BLAST alignment as read from the input file 
                         
 Score = 128.0 bits (116.7), Expect = 4.62E-22
 Identities = 209/295 (71%), Gaps = 18/295 (6%)
 Strand = Plus/Minus
Query      15 AGGCCAAGACCCAGCCGGAAGAGAAGGCTAGATCTCCCGACCCAAGCTCCTAGCACGGATAC 76     
              ||||||||| | |  |||||||||||| | ||||||||||||  |  ||| |||||||| ||        
Sbjct 4145853 AGGCCAAGAGCGAATCGGAAGAGAAGGTTCGATCTCCCGACCGGACTTCCCAGCACGGACAC 4145792

Query      77 CGAGCACCCACGCGGAGCACATGCCGCGGAATAGGCAAAAGTGTTGCGGACCTGCGTAGTTT 138    
              ||||||||||||||||||  ||||| |   |  |||||||| |||| || | |||| || ||        
Sbjct 4145791 CGAGCACCCACGCGGAGCCGATGCCACTATAAGGGCAAAAGCGTTGTGGGCTTGCG-AGATT 4145731

Query     139 CGAAAAGCGGACGGC---CACGACG-GCCCTTTGGGTGG-GGTTGCAGCCGTAGCGCATCGC 195    
              ||       ||||||   |||  ||  ||| ||| | ||  |   ||||  || |    ||         
Sbjct 4145730 CGGGTCTTCGACGGCGAGCACCCCGCACCCGTTGCGAGGACGAACCAGCGATATC----CGA 4145673

Query     196 AAAGACGCCGAGGTCA--CCCACGCAACCCACATCGCACGCTTGGTCACTCGG-GGTCCGTG 254    
              |    |||||||| ||    ||| |||||||    ||||||||||| ||||||  |||||||        
Sbjct 4145672 ACCCTCGCCGAGGCCAGTGACACACAACCCATGCAGCACGCTTGGTAACTCGGCAGTCCGTG 4145611

Query     255 CTAGCGGGCGGCG-AACCG--GACATTGTCCGGGACGCCGCCCGATT 298    
              || ||||||| ||  ||||  | |||  || | |||| |||||| ||        
Sbjct 4145610 CTGGCGGGCGACGCCACCGAAGTCAT--TCGGCGACGTCGCCCGCTT 4145566

 
                 
                
                 
                      Report:  
                     
                         
                             sequence start
                                   ?  
                                     
Start position of the estimated full-length sequence in genome.
Start index  
                                 :
                             
                             4145560 
                         
                         
                             sequence end
                                   ?  
                                     
End position of the estimated full-length sequence in genome.
Start index  
                                 :
                             
                             4145867 
                         
                         
                             bit score (CM)
                                   ?  
                                     
The score for aligning estimated full-length sequence to CM model
  (computed by RSEARCH -> default,
  infered from Rfam or provided by user) 
                                 :
                             
                             165.64 
                         
                         
                             Homology estimate
                                   ?  
                                     
Quick homology estimate:
  Not homologous: bit score   20 and bit score > 0.5 * query length
  Uncertain otherwise 
                                 :
                             
                             Homologous 
                         
                     
                 
                
                
                 
                     
                          Estimated full-length sequence:  
                         
                          ? 
                             
Click checkbox to select multiple seuqences.
Fasta header format:
  UID|accession.versionSTRAND start-end 
                         
                     
                     >uid:5|CP024315.1rc 4145560-4145867
UACGGAAGGACGGAAGGCCAAGAGCGAAUCGGAAGAGAAGGUUCGAUCUCCCGACCGGAC
UUCCCAGCACGGACACCGAGCACCCACGCGGAGCCGAUGCCACUAUAAGGGCAAAAGCGU
UGUGGGCUUGCGAGAUUCGGGUCUUCGACGGCGAGCACCCCGCACCCGUUGCGAGGACGA
ACCAGCGAUAUCCGAACCCUCGCCGAGGCCAGUGACACACAACCCAUGCAGCACGCUUGG
UAACUCGGCAGUCCGUGCUGGCGGGCGACGCCACCGAAGUCAUUCGGCGACGUCGCCCGC
UUACAUGU
 
                 
                
             
            
             
                
                     
                         
                         
                             TurboFold 
                             
                              ? 
                                 
Visualisation of predicted secondary structure.
To save the image:
  Right click on the image -> Save Image as. 
                             
                         
                     
                
                     
                         
                         
                             rfam-Rc 
                             
                              ? 
                                 
Visualisation of predicted secondary structure.
To save the image:
  Right click on the image -> Save Image as. 
                             
                         
                     
                
                     
                         
                         
                             rnafold 
                             
                              ? 
                                 
Visualisation of predicted secondary structure.
To save the image:
  Right click on the image -> Save Image as. 
                             
                         
                     
                
             
            

        
             
                 Load Sequence viewer 
             
        
        
         
     
    

    
     
         
            Hit: CP015529.1
         
         
             
                 
                     CP015529.1 Rhodococcus sp. WB1, complete genome 
                     
                         
 ?  
This is BLAST alignment as read from the input file 
                         
 Score = 123.0 bits (112.2), Expect = 1.96E-20
 Identities = 208/295 (71%), Gaps = 18/295 (6%)
 Strand = Plus/Plus
Query      15 AGGCCAAGACCCAGCCGGAAGAGAAGGCTAGATCTCCCGACCCAAGCTCCTAGCACGGATAC 76     
              ||||||||| | |  |||||||||||| | ||||||||||||  |  ||| |||||||| ||        
Sbjct 4825131 AGGCCAAGAGCGAATCGGAAGAGAAGGTTCGATCTCCCGACCGGAATTCCCAGCACGGACAC 4825192

Query      77 CGAGCACCCACGCGGAGCACATGCCGCGGAATAGGCAAAAGTGTTGCGGACCTGCGTAGTTT 138    
              ||||||||||||||||||  ||||| |   |  ||||||||||||| || | |||| || ||        
Sbjct 4825193 CGAGCACCCACGCGGAGCCGATGCCACTATAAGGGCAAAAGTGTTGTGGGCTTGCG-AGATT 4825253

Query     139 CGAAAAGCGGACGGC---CACGACGG-CCCTTTGGGTGGG-GTTGCAGCCGTAGCGCATCGC 195    
              ||       || |||   |||  ||  ||| ||| | ||  |   ||||  || |  | | |        
Sbjct 4825254 CGGGTCTTCGAAGGCGAACACCCCGCACCCGTTGCGAGGACGAACCAGCGATATCCGAACCC 4825315

Query     196 AAAGACGCCGAGGTCACC--CACGCAACCCACATCGCACGCTTGGTCACTCGG-GGTCCGTG 254    
                   |||||||| ||    ||| |||||||    ||||||||||| ||||||  |||||||        
Sbjct 4825316 T----CGCCGAGGCCAGTGACACACAACCCATGCAGCACGCTTGGTAACTCGGCAGTCCGTG 4825373

Query     255 CTAGCGGGCGGCG-AACCG--GACATTGTCCGGGACGCCGCCCGATT 298    
              |  ||||||| ||  ||||  | |||  || | |||| |||||| ||        
Sbjct 4825374 CCGGCGGGCGACGCCACCGAAGTCAT--TCGGCGACGTCGCCCGCTT 4825418

 
                 
                
                 
                      Report:  
                     
                         
                             sequence start
                                   ?  
                                     
Start position of the estimated full-length sequence in genome.
Start index  
                                 :
                             
                             4825118 
                         
                         
                             sequence end
                                   ?  
                                     
End position of the estimated full-length sequence in genome.
Start index  
                                 :
                             
                             4825424 
                         
                         
                             bit score (CM)
                                   ?  
                                     
The score for aligning estimated full-length sequence to CM model
  (computed by RSEARCH -> default,
  infered from Rfam or provided by user) 
                                 :
                             
                             164.41 
                         
                         
                             Homology estimate
                                   ?  
                                     
Quick homology estimate:
  Not homologous: bit score   20 and bit score > 0.5 * query length
  Uncertain otherwise 
                                 :
                             
                             Homologous 
                         
                     
                 
                
                
                 
                     
                          Estimated full-length sequence:  
                         
                          ? 
                             
Click checkbox to select multiple seuqences.
Fasta header format:
  UID|accession.versionSTRAND start-end 
                         
                     
                     >uid:6|CP015529.1fw 4825118-4825424
ACGGAAGGACGGAAGGCCAAGAGCGAAUCGGAAGAGAAGGUUCGAUCUCCCGACCGGAAU
UCCCAGCACGGACACCGAGCACCCACGCGGAGCCGAUGCCACUAUAAGGGCAAAAGUGUU
GUGGGCUUGCGAGAUUCGGGUCUUCGAAGGCGAACACCCCGCACCCGUUGCGAGGACGAA
CCAGCGAUAUCCGAACCCUCGCCGAGGCCAGUGACACACAACCCAUGCAGCACGCUUGGU
AACUCGGCAGUCCGUGCCGGCGGGCGACGCCACCGAAGUCAUUCGGCGACGUCGCCCGCU
UACAUGU
 
                 
                
             
            
             
                
                     
                         
                         
                             TurboFold 
                             
                              ? 
                                 
Visualisation of predicted secondary structure.
To save the image:
  Right click on the image -> Save Image as. 
                             
                         
                     
                
                     
                         
                         
                             rfam-Rc 
                             
                              ? 
                                 
Visualisation of predicted secondary structure.
To save the image:
  Right click on the image -> Save Image as. 
                             
                         
                     
                
                     
                         
                         
                             rnafold 
                             
                              ? 
                                 
Visualisation of predicted secondary structure.
To save the image:
  Right click on the image -> Save Image as. 
                             
                         
                     
                
             
            

        
             
                 Load Sequence viewer 
             
        
        
         
     
    

    
     
         
            Hit: CP011341.1
         
         
             
                 
                     CP011341.1 Rhodococcus aetherivorans strain IcdP1, complete genome 
                     
                         
 ?  
This is BLAST alignment as read from the input file 
                         
 Score = 123.0 bits (112.2), Expect = 1.96E-20
 Identities = 101/126 (80%), Gaps = 1/126 (1%)
 Strand = Plus/Minus
Query     15 AGGCCAAGACCCAGCCGGAAGAGAAGGCTAGATCTCCCGACCCAAGCTCCTAGCACGGATACCG 78    
             ||||||||| | |  |||||||||||| | ||||||||||||  |  ||| |||||||| ||||       
Sbjct 700390 AGGCCAAGAGCGAATCGGAAGAGAAGGTTCGATCTCCCGACCGGAATTCCCAGCACGGACACCG 700327

Query     79 AGCACCCACGCGGAGCACATGCCGCGGAATAGGCAAAAGTGTTGCGGACCTGCGTAGTTTCG 140   
             ||||||||||||||||  ||||| |   |  ||||||||||||| || | |||| || ||||       
Sbjct 700326 AGCACCCACGCGGAGCCGATGCCACTATAAGGGCAAAAGTGTTGTGGGCTTGCG-AGATTCG 700266

 
                 
                
                 
                      Report:  
                     
                         
                             sequence start
                                   ?  
                                     
Start position of the estimated full-length sequence in genome.
Start index  
                                 :
                             
                             700103 
                         
                         
                             sequence end
                                   ?  
                                     
End position of the estimated full-length sequence in genome.
Start index  
                                 :
                             
                             700409 
                         
                         
                             bit score (CM)
                                   ?  
                                     
The score for aligning estimated full-length sequence to CM model
  (computed by RSEARCH -> default,
  infered from Rfam or provided by user) 
                                 :
                             
                             164.41 
                         
                         
                             Homology estimate
                                   ?  
                                     
Quick homology estimate:
  Not homologous: bit score   20 and bit score > 0.5 * query length
  Uncertain otherwise 
                                 :
                             
                             Homologous 
                         
                     
                 
                
                
                 
                     
                          Estimated full-length sequence:  
                         
                          ? 
                             
Click checkbox to select multiple seuqences.
Fasta header format:
  UID|accession.versionSTRAND start-end 
                         
                     
                     >uid:7|CP011341.1rc 700103-700409
ACGGAAGGACGGAAGGCCAAGAGCGAAUCGGAAGAGAAGGUUCGAUCUCCCGACCGGAAU
UCCCAGCACGGACACCGAGCACCCACGCGGAGCCGAUGCCACUAUAAGGGCAAAAGUGUU
GUGGGCUUGCGAGAUUCGGGUCUUCGAAGGCGAACACCCCGCACCCGUUGCGAGGACGAA
CCAGCGAUAUCCGAACCCUCGCCGAGGCCAGUGACACACAACCCAUGCAGCACGCUUGGU
AACUCGGCAGUCCGUGCCGGCGGGCGACGCCACCGAAGUCAUUCGGCGACGUCGCCCGCU
UACAUGU
 
                 
                
             
            
             
                
                     
                         
                         
                             TurboFold 
                             
                              ? 
                                 
Visualisation of predicted secondary structure.
To save the image:
  Right click on the image -> Save Image as. 
                             
                         
                     
                
                     
                         
                         
                             rfam-Rc 
                             
                              ? 
                                 
Visualisation of predicted secondary structure.
To save the image:
  Right click on the image -> Save Image as. 
                             
                         
                     
                
                     
                         
                         
                             rnafold 
                             
                              ? 
                                 
Visualisation of predicted secondary structure.
To save the image:
  Right click on the image -> Save Image as. 
                             
                         
                     
                
             
            

        
             
                 Load Sequence viewer 
             
        
        
         
     
    

    
     
         
            Hit: CP011341.1
         
         
             
                 
                     CP011341.1 Rhodococcus aetherivorans strain IcdP1, complete genome 
                     
                         
 ?  
This is BLAST alignment as read from the input file 
                         
 Score = 56.0 bits (51.8), Expect = 1.61E-02
 Identities = 77/104 (74%), Gaps = 8/104 (8%)
 Strand = Plus/Minus
Query    201 CGCCGAGGTCACC--CACGCAACCCACATCGCACGCTTGGTCACTCGG-GGTCCGTGCTAGCGG 261   
             |||||||| ||    ||| |||||||    ||||||||||| ||||||  ||||||||  ||||       
Sbjct 700204 CGCCGAGGCCAGTGACACACAACCCATGCAGCACGCTTGGTAACTCGGCAGTCCGTGCCGGCGG 700141

Query    262 GCGGCG-AACCG--GACATTGTCCGGGACGCCGCCCGATT 298   
             ||| ||  ||||  | |||  || | |||| |||||| ||       
Sbjct 700140 GCGACGCCACCGAAGTCAT--TCGGCGACGTCGCCCGCTT 700103

 
                 
                
                 
                      Report:  
                     
                         
                             sequence start
                                   ?  
                                     
Start position of the estimated full-length sequence in genome.
Start index  
                                 :
                             
                             700098 
                         
                         
                             sequence end
                                   ?  
                                     
End position of the estimated full-length sequence in genome.
Start index  
                                 :
                             
                             700404 
                         
                         
                             bit score (CM)
                                   ?  
                                     
The score for aligning estimated full-length sequence to CM model
  (computed by RSEARCH -> default,
  infered from Rfam or provided by user) 
                                 :
                             
                             164.41 
                         
                         
                             Homology estimate
                                   ?  
                                     
Quick homology estimate:
  Not homologous: bit score   20 and bit score > 0.5 * query length
  Uncertain otherwise 
                                 :
                             
                             Homologous 
                         
                     
                 
                
                
                 
                     
                          Estimated full-length sequence:  
                         
                          ? 
                             
Click checkbox to select multiple seuqences.
Fasta header format:
  UID|accession.versionSTRAND start-end 
                         
                     
                     >uid:8|CP011341.1rc 700098-700404
ACGGAAGGACGGAAGGCCAAGAGCGAAUCGGAAGAGAAGGUUCGAUCUCCCGACCGGAAU
UCCCAGCACGGACACCGAGCACCCACGCGGAGCCGAUGCCACUAUAAGGGCAAAAGUGUU
GUGGGCUUGCGAGAUUCGGGUCUUCGAAGGCGAACACCCCGCACCCGUUGCGAGGACGAA
CCAGCGAUAUCCGAACCCUCGCCGAGGCCAGUGACACACAACCCAUGCAGCACGCUUGGU
AACUCGGCAGUCCGUGCCGGCGGGCGACGCCACCGAAGUCAUUCGGCGACGUCGCCCGCU
UACAUGU
 
                 
                
             
            
             
                
                     
                         
                         
                             TurboFold 
                             
                              ? 
                                 
Visualisation of predicted secondary structure.
To save the image:
  Right click on the image -> Save Image as. 
                             
                         
                     
                
                     
                         
                         
                             rfam-Rc 
                             
                              ? 
                                 
Visualisation of predicted secondary structure.
To save the image:
  Right click on the image -> Save Image as. 
                             
                         
                     
                
                     
                         
                         
                             rnafold 
                             
                              ? 
                                 
Visualisation of predicted secondary structure.
To save the image:
  Right click on the image -> Save Image as. 
                             
                         
                     
                
             
            

        
             
                 Load Sequence viewer 
             
        
        
         
     
    

    
     
         
            Hit: FO082843.1
         
         
             
                 
                     FO082843.1 Nocardia cyriacigeorgica GUH-2 chromosome complete genome 
                     
                         
 ?  
This is BLAST alignment as read from the input file 
                         
 Score = 121.0 bits (110.4), Expect = 6.85E-20
 Identities = 209/292 (72%), Gaps = 17/292 (6%)
 Strand = Plus/Minus
Query     15 AGGCCAAGACCCAGCCGGAAGAGAAGGCTAGATCTCCCGACCCAAGCT-CCTAGCACGGATACC 77    
             |||||| |||  || ||||||||||||||    | |||  ||||  || || ||||||||  ||       
Sbjct 383868 AGGCCAGGACGGAGTCGGAAGAGAAGGCTCAGCCCCCCATCCCACCCTTCCCAGCACGGACGCC 383805

Query     78 GAGCACCCACGCGGAGCACATGCCGCGGAATAGGCAAAAGTGTTGCGGACCTGCGTAGTTTCGA 141   
               ||||||||||||||| |  ||||||  |  |||||||| |||| || |||||| |  |||||       
Sbjct 383804 AGGCACCCACGCGGAGCGC--GCCGCGACAAGGGCAAAAGCGTTGTGGGCCTGCG-ACATTCGA 383744

Query    142 AAAGCGGACGGCCA-CGAC-GGCCCTTTGGGTGGGGTTGCAGCCGTAGCGCA-TCGCA-AAGAC 201   
                || |||||| | || |  || | ||  | |||| || |||||  | | | ||| |  |  |       
Sbjct 383743 GCTGCCGACGGCGAGCGCCTCGCACATT-TGCGGGGATG-AGCCGCGGTGGATTCGGATCAATC 383682

Query    202 GCCGAGGTC-ACCCACGCAACCCACATCGCACGCTTGGTCACTCGGG--GTCCGTGCTAGCGGG 262   
             ||||||| | |   || ||||||||   ||||||||||| || ||||  |||||||||||||||       
Sbjct 383681 GCCGAGGCCGAAGAACACAACCCACTCAGCACGCTTGGTAAC-CGGGTTGTCCGTGCTAGCGGG 383619

Query    263 CGGCGAACCGGACATTGTCCGGGACGCCGCCCGATT 298   
             ||| ||   || |  || | |  |||||||||| ||       
Sbjct 383618 CGGTGA---GGTCGGTGACGGCAACGCCGCCCGCTT 383586

 
                 
                
                 
                      Report:  
                     
                         
                             sequence start
                                   ?  
                                     
Start position of the estimated full-length sequence in genome.
Start index  
                                 :
                             
                             383581 
                         
                         
                             sequence end
                                   ?  
                                     
End position of the estimated full-length sequence in genome.
Start index  
                                 :
                             
                             383882 
                         
                         
                             bit score (CM)
                                   ?  
                                     
The score for aligning estimated full-length sequence to CM model
  (computed by RSEARCH -> default,
  infered from Rfam or provided by user) 
                                 :
                             
                             138.69 
                         
                         
                             Homology estimate
                                   ?  
                                     
Quick homology estimate:
  Not homologous: bit score   20 and bit score > 0.5 * query length
  Uncertain otherwise 
                                 :
                             
                             Uncertain 
                         
                     
                 
                
                
                 
                     
                          Estimated full-length sequence:  
                         
                          ? 
                             
Click checkbox to select multiple seuqences.
Fasta header format:
  UID|accession.versionSTRAND start-end 
                         
                     
                     >uid:9|FO082843.1rc 383581-383882
ACGGAAGGACGGAAGGCCAGGACGGAGUCGGAAGAGAAGGCUCAGCCCCCCAUCCCACCC
UUCCCAGCACGGACGCCAGGCACCCACGCGGAGCGCGCCGCGACAAGGGCAAAAGCGUUG
UGGGCCUGCGACAUUCGAGCUGCCGACGGCGAGCGCCUCGCACAUUUGCGGGGAUGAGCC
GCGGUGGAUUCGGAUCAAUCGCCGAGGCCGAAGAACACAACCCACUCAGCACGCUUGGUA
ACCGGGUUGUCCGUGCUAGCGGGCGGUGAGGUCGGUGACGGCAACGCCGCCCGCUUCGAU
AU
 
                 
                
             
            
             
                
                     
                         
                         
                             TurboFold 
                             
                              ? 
                                 
Visualisation of predicted secondary structure.
To save the image:
  Right click on the image -> Save Image as. 
                             
                         
                     
                
                     
                         
                         
                             rfam-Rc 
                             
                              ? 
                                 
Visualisation of predicted secondary structure.
To save the image:
  Right click on the image -> Save Image as. 
                             
                         
                     
                
                     
                         
                         
                             rnafold 
                             
                              ? 
                                 
Visualisation of predicted secondary structure.
To save the image:
  Right click on the image -> Save Image as. 
                             
                         
                     
                
             
            

        
             
                 Load Sequence viewer 
             
        
        
         
     
    

    
     
         
            Hit: CP026746.1
         
         
             
                 
                     CP026746.1 Nocardia cyriacigeorgica strain MDA3349 chromosome, complete genome 
                     
                         
 ?  
This is BLAST alignment as read from the input file 
                         
 Score = 119.0 bits (108.6), Expect = 2.39E-19
 Identities = 188/261 (72%), Gaps = 12/261 (5%)
 Strand = Plus/Plus
Query      15 AGGCCAAGACCCAGCCGGAAGAGAAGGCTAGATCTCCCGACCCAAGCT-CCTAGCACGGATA 75     
              |||||| |||  || ||||||||||||||    | |||  ||||  || || ||||||||          
Sbjct 1293452 AGGCCAGGACGGAGTCGGAAGAGAAGGCTCAGCCCCCCATCCCACCCTTCCCAGCACGGACG 1293513

Query      76 CCGAGCACCCACGCGGAGCACATGCCGCGGAATAGGCAAAAGTGTTGCGGACCTGCGTAGTT 137    
              ||  ||||||||||||||| |  ||||||  |  |||||||| |||| || |||||| |  |        
Sbjct 1293514 CCAGGCACCCACGCGGAGCGC--GCCGCGACAAGGGCAAAAGCGTTGTGGGCCTGCG-ACAT 1293572

Query     138 TCGAAAAGCGGACGGCCA-CGACGGCCCTTTGGGTGGGGTTGCAGCCGTAG-CGCATCGCA- 196    
              ||||   || |||||| | || |   | |    | |||| || |||||  |  | |||| |         
Sbjct 1293573 TCGAGCTGCCGACGGCGAGCGCCTCGCATAATTGCGGGGATG-AGCCGCGGTGGAATCGGAT 1293633

Query     197 AAGACGCCGAGGTC-ACCCACGCAACCCACATCGCACGCTTGGTCACTCGGG--GTCCGTGC 255    
               || |||||||| | |   || ||||||||   ||||||||||| || ||||  ||||||||        
Sbjct 1293634 CAGTCGCCGAGGCCGAAGAACACAACCCACTCAGCACGCTTGGTAAC-CGGGTTGTCCGTGC 1293694

Query     256 TAGCGGGCGGCGA 268    
              |||||||||| ||        
Sbjct 1293695 TAGCGGGCGGTGA 1293707

 
                 
                
                 
                      Report:  
                     
                         
                             sequence start
                                   ?  
                                     
Start position of the estimated full-length sequence in genome.
Start index  
                                 :
                             
                             1293439 
                         
                         
                             sequence end
                                   ?  
                                     
End position of the estimated full-length sequence in genome.
Start index  
                                 :
                             
                             1293740 
                         
                         
                             bit score (CM)
                                   ?  
                                     
The score for aligning estimated full-length sequence to CM model
  (computed by RSEARCH -> default,
  infered from Rfam or provided by user) 
                                 :
                             
                             143.06 
                         
                         
                             Homology estimate
                                   ?  
                                     
Quick homology estimate:
  Not homologous: bit score   20 and bit score > 0.5 * query length
  Uncertain otherwise 
                                 :
                             
                             Uncertain 
                         
                     
                 
                
                
                 
                     
                          Estimated full-length sequence:  
                         
                          ? 
                             
Click checkbox to select multiple seuqences.
Fasta header format:
  UID|accession.versionSTRAND start-end 
                         
                     
                     >uid:10|CP026746.1fw 1293439-1293740
ACGGAAGGACGGAAGGCCAGGACGGAGUCGGAAGAGAAGGCUCAGCCCCCCAUCCCACCC
UUCCCAGCACGGACGCCAGGCACCCACGCGGAGCGCGCCGCGACAAGGGCAAAAGCGUUG
UGGGCCUGCGACAUUCGAGCUGCCGACGGCGAGCGCCUCGCAUAAUUGCGGGGAUGAGCC
GCGGUGGAAUCGGAUCAGUCGCCGAGGCCGAAGAACACAACCCACUCAGCACGCUUGGUA
ACCGGGUUGUCCGUGCUAGCGGGCGGUGAGGUCGGUGACGACAACGCCGCCCGCUUCGAU
AU
 
                 
                
             
            
             
                
                     
                         
                         
                             TurboFold 
                             
                              ? 
                                 
Visualisation of predicted secondary structure.
To save the image:
  Right click on the image -> Save Image as. 
                             
                         
                     
                
                     
                         
                         
                             rfam-Rc 
                             
                              ? 
                                 
Visualisation of predicted secondary structure.
To save the image:
  Right click on the image -> Save Image as. 
                             
                         
                     
                
                     
                         
                         
                             rnafold 
                             
                              ? 
                                 
Visualisation of predicted secondary structure.
To save the image:
  Right click on the image -> Save Image as. 
                             
                         
                     
                
             
            

        
             
                 Load Sequence viewer 
             
        
        
         
     
    

    
     
         
            Hit: CP018082.1
         
         
             
                 
                     CP018082.1 Nocardia sp. Y48 chromosome, complete genome 
                     
                         
 ?  
This is BLAST alignment as read from the input file 
                         
 Score = 116.0 bits (105.9), Expect = 8.35E-19
 Identities = 186/260 (72%), Gaps = 16/260 (6%)
 Strand = Plus/Minus
Query     15 AGGCCAAGACCCAGCCGGAAGAGAAGGCTAGATCTCCCGACCCAAGC-TCCTAGCACGGATACC 77    
             ||||||||||  ||||||||||||||| |    |||||  ||||  | ||| ||||||||  ||       
Sbjct 360701 AGGCCAAGACGGAGCCGGAAGAGAAGGTTTCGCCTCCCATCCCACCCTTCCCAGCACGGACGCC 360638

Query     78 GAGCACCCACGCGGAGCACATGCCGCGGAATAGGCAAAAGTGTTGCGGACCTGCGTAGTTTCGA 141   
               ||||||||||||||| |  ||||||  |  ||||  || |||| || |||||| |  ||||        
Sbjct 360637 AGGCACCCACGCGGAGCGC--GCCGCGACAAGGGCAGCAGAGTTGTGGGCCTGCG-AAATTCG- 360578

Query    142 AAAGCGGACGGCCACGACGGCC---CTTTGG--GTGGGGTTGCAGCCGTAGCGCATCGCAAAGA 200   
               ||||| || |  ||| ||||   |   ||  | |||| || | |||  | |  |||             
Sbjct 360577 --AGCGGTCGTCGGCGAAGGCCTCGCACAGGTTGCGGGGATG-AACCGGCGGGGTTCGGGTGAT 360517

Query    201 CGCCGAGGTCACC-CACGCAACCCACATC-GCACGCTTGGTCACTCGG-GGTCCGTGCTAGCGG 261   
             |||||||| ||||  || |||||||| || ||||||||||| ||  || | |||||||||||||       
Sbjct 360516 CGCCGAGGCCACCGAACACAACCCACCTCAGCACGCTTGGTAACCGGGTGTTCCGTGCTAGCGG 360453

Query    262 GCGG 265   
             ||||       
Sbjct 360452 GCGG 360449

 
                 
                
                 
                      Report:  
                     
                         
                             sequence start
                                   ?  
                                     
Start position of the estimated full-length sequence in genome.
Start index  
                                 :
                             
                             360411 
                         
                         
                             sequence end
                                   ?  
                                     
End position of the estimated full-length sequence in genome.
Start index  
                                 :
                             
                             360714 
                         
                         
                             bit score (CM)
                                   ?  
                                     
The score for aligning estimated full-length sequence to CM model
  (computed by RSEARCH -> default,
  infered from Rfam or provided by user) 
                                 :
                             
                             137.66 
                         
                         
                             Homology estimate
                                   ?  
                                     
Quick homology estimate:
  Not homologous: bit score   20 and bit score > 0.5 * query length
  Uncertain otherwise 
                                 :
                             
                             Uncertain 
                         
                     
                 
                
                
                 
                     
                          Estimated full-length sequence:  
                         
                          ? 
                             
Click checkbox to select multiple seuqences.
Fasta header format:
  UID|accession.versionSTRAND start-end 
                         
                     
                     >uid:11|CP018082.1rc 360411-360714
ACGGAAGGACGGAAGGCCAAGACGGAGCCGGAAGAGAAGGUUUCGCCUCCCAUCCCACCC
UUCCCAGCACGGACGCCAGGCACCCACGCGGAGCGCGCCGCGACAAGGGCAGCAGAGUUG
UGGGCCUGCGAAAUUCGAGCGGUCGUCGGCGAAGGCCUCGCACAGGUUGCGGGGAUGAAC
CGGCGGGGUUCGGGUGAUCGCCGAGGCCACCGAACACAACCCACCUCAGCACGCUUGGUA
ACCGGGUGUUCCGUGCUAGCGGGCGGUGCGGUCGAGUAGUCGGCUACGCCGCCCGUUUCG
AUGU
 
                 
                
             
            
             
                
                     
                         
                         
                             TurboFold 
                             
                              ? 
                                 
Visualisation of predicted secondary structure.
To save the image:
  Right click on the image -> Save Image as. 
                             
                         
                     
                
                     
                         
                         
                             rfam-Rc 
                             
                              ? 
                                 
Visualisation of predicted secondary structure.
To save the image:
  Right click on the image -> Save Image as. 
                             
                         
                     
                
                     
                         
                         
                             rnafold 
                             
                              ? 
                                 
Visualisation of predicted secondary structure.
To save the image:
  Right click on the image -> Save Image as. 
                             
                         
                     
                
             
            

        
             
                 Load Sequence viewer 
             
        
        
         
     
    

    
     
         
            Hit: LR134352.1
         
         
             
                 
                     LR134352.1 Nocardia asteroides strain NCTC11293 genome assembly, chromosome: 1 
                     
                         
 ?  
This is BLAST alignment as read from the input file 
                         
 Score = 112.0 bits (102.3), Expect = 1.02E-17
 Identities = 203/290 (70%), Gaps = 12/290 (4%)
 Strand = Plus/Minus
Query      15 AGGCCAAGACCCAGCCGGAAGAGAAGGC-TAGATCTCCCGACCCAAGC-TCCTAGCACGGAT 74     
              ||||||||||  |||||||||||||||| |    |||||  ||||  | ||| ||||||||         
Sbjct 1139756 AGGCCAAGACGGAGCCGGAAGAGAAGGCATTCGCCTCCCATCCCACCCTTCCCAGCACGGAC 1139695

Query      75 ACCGAGCACCCACGCGGAGCACATGCCGCGGAATAGGCAAAAGTGTTGCGGACCTGCGTAGT 136    
               ||  ||||||||||||||| |  ||||||  |  ||||  || |||| || |||||| |          
Sbjct 1139694 GCCAGGCACCCACGCGGAGCGC--GCCGCGACAAGGGCAGCAGAGTTGTGGGCCTGCG-AAA 1139636

Query     137 TTCGAAAAGCGGACGGCCACGACGGCCCTTTGG--GTGGGGTTGCAGCCGTAGCGCATCGCA 196    
              ||||   ||  | |||| | | |  | |   ||  | |||| || | |||  | |  ||| |        
Sbjct 1139635 TTCGGGCAGTCGTCGGCGAAGGCCTCGCACAGGTTGCGGGGATG-AACCGGCGGGGTTCGGA 1139575

Query     197 AAGACGCCGAGGTCACC-CACGCAACCCACATCGCACGCTTGGTCACTCGG-GGTCCGTGCT 256    
                  |||||||| ||||  || ||||||||   ||||||||||| ||  || | ||||||||        
Sbjct 1139574 TGATCGCCGAGGCCACCGAACACAACCCACCGAGCACGCTTGGTAACCGGGTGTTCCGTGCT 1139513

Query     257 AGCGGGCGGCGAACCGGACATTGTCCGGGACGCCGCCCGATT 298    
              ||||||||| ||  | |  | |   ||| |||||||||| ||        
Sbjct 1139512 AGCGGGCGGTGAGGCCG--AGTAATCGGCACGCCGCCCGCTT 1139473

 
                 
                
                 
                      Report:  
                     
                         
                             sequence start
                                   ?  
                                     
Start position of the estimated full-length sequence in genome.
Start index  
                                 :
                             
                             1139468 
                         
                         
                             sequence end
                                   ?  
                                     
End position of the estimated full-length sequence in genome.
Start index  
                                 :
                             
                             1139770 
                         
                         
                             bit score (CM)
                                   ?  
                                     
The score for aligning estimated full-length sequence to CM model
  (computed by RSEARCH -> default,
  infered from Rfam or provided by user) 
                                 :
                             
                             137.32 
                         
                         
                             Homology estimate
                                   ?  
                                     
Quick homology estimate:
  Not homologous: bit score   20 and bit score > 0.5 * query length
  Uncertain otherwise 
                                 :
                             
                             Uncertain 
                         
                     
                 
                
                
                 
                     
                          Estimated full-length sequence:  
                         
                          ? 
                             
Click checkbox to select multiple seuqences.
Fasta header format:
  UID|accession.versionSTRAND start-end 
                         
                     
                     >uid:12|LR134352.1rc 1139468-1139770
ACGGAAGGACGGAAGGCCAAGACGGAGCCGGAAGAGAAGGCAUUCGCCUCCCAUCCCACC
CUUCCCAGCACGGACGCCAGGCACCCACGCGGAGCGCGCCGCGACAAGGGCAGCAGAGUU
GUGGGCCUGCGAAAUUCGGGCAGUCGUCGGCGAAGGCCUCGCACAGGUUGCGGGGAUGAA
CCGGCGGGGUUCGGAUGAUCGCCGAGGCCACCGAACACAACCCACCGAGCACGCUUGGUA
ACCGGGUGUUCCGUGCUAGCGGGCGGUGAGGCCGAGUAAUCGGCACGCCGCCCGCUUCGA
UGU
 
                 
                
             
            
             
                
                     
                         
                         
                             TurboFold 
                             
                              ? 
                                 
Visualisation of predicted secondary structure.
To save the image:
  Right click on the image -> Save Image as. 
                             
                         
                     
                
                     
                         
                         
                             rfam-Rc 
                             
                              ? 
                                 
Visualisation of predicted secondary structure.
To save the image:
  Right click on the image -> Save Image as. 
                             
                         
                     
                
                     
                         
                         
                             rnafold 
                             
                              ? 
                                 
Visualisation of predicted secondary structure.
To save the image:
  Right click on the image -> Save Image as. 
                             
                         
                     
                
             
            

        
             
                 Load Sequence viewer 
             
        
        
         
     
    

    
     
         
            Hit: CP031418.1
         
         
             
                 
                     CP031418.1 Nocardia farcinica strain W6977 chromosome, complete genome 
                     
                         
 ?  
This is BLAST alignment as read from the input file 
                         
 Score = 110.0 bits (100.5), Expect = 3.55E-17
 Identities = 189/264 (72%), Gaps = 17/264 (6%)
 Strand = Plus/Plus
Query      15 AGGCCAAGACCCAGCCGGAAGAGAAGGCTAGATCTCCCGACCCAAGCTCCTAGCACGGATAC 76     
              |||||||| |  | |||||||||||||  |  ||||||||||     ||| ||||||||  |        
Sbjct 3365486 AGGCCAAGGCGGAACCGGAAGAGAAGGTCACGTCTCCCGACCACCTTTCCCAGCACGGAGGC 3365547

Query      77 CGAGCACCCACGCGGAGCACATGCCGC-GGAATAGGCAAAAGTGTTGCGGACCTGCGTAGTT 137    
              |  | ||||||||||||| |  |||||  | || |||| ||| |||| || ||||||||| |        
Sbjct 3365548 CAGGTACCCACGCGGAGCGC--GCCGCTTGGAT-GGCAGAAGCGTTGTGGGCCTGCGTAG-T 3365605

Query     138 TCGAAAAGCGGACGGCCACGACGGCCCTTTGG--GTGGGGTTGCAGCCGTAGCGCA--TCGC 195    
              ||||   || |||||| |   |  | |   ||  | |||| || | |||  ||| |  |||         
Sbjct 3365606 TCGAGCTGCCGACGGCGAGAGCCTCGCACAGGTTGCGGGGATG-AACCG--GCGGAGTTCGG 3365664

Query     196 AAAGACGCCGAGGTCA-CCCACGCAACCCACATC-GCACGCTTGGTCACTCGGG--GTCCGT 253    
              |    |||||||| ||    || |||||||| || ||||||||||| || ||||   |||||        
Sbjct 3365665 ATGATCGCCGAGGCCATGGAACACAACCCACCTCAGCACGCTTGGTAAC-CGGGTACTCCGT 3365725

Query     254 GCTAGCGGGCGGCGAA 269    
              |||||||||||| |||        
Sbjct 3365726 GCTAGCGGGCGGTGAA 3365741

 
                 
                
                 
                      Report:  
                     
                         
                             sequence start
                                   ?  
                                     
Start position of the estimated full-length sequence in genome.
Start index  
                                 :
                             
                             3365472 
                         
                         
                             sequence end
                                   ?  
                                     
End position of the estimated full-length sequence in genome.
Start index  
                                 :
                             
                             3365777 
                         
                         
                             bit score (CM)
                                   ?  
                                     
The score for aligning estimated full-length sequence to CM model
  (computed by RSEARCH -> default,
  infered from Rfam or provided by user) 
                                 :
                             
                             135.94 
                         
                         
                             Homology estimate
                                   ?  
                                     
Quick homology estimate:
  Not homologous: bit score   20 and bit score > 0.5 * query length
  Uncertain otherwise 
                                 :
                             
                             Uncertain 
                         
                     
                 
                
                
                 
                     
                          Estimated full-length sequence:  
                         
                          ? 
                             
Click checkbox to select multiple seuqences.
Fasta header format:
  UID|accession.versionSTRAND start-end 
                         
                     
                     >uid:13|CP031418.1fw 3365472-3365777
UACGGAAAGGCGGAAGGCCAAGGCGGAACCGGAAGAGAAGGUCACGUCUCCCGACCACCU
UUCCCAGCACGGAGGCCAGGUACCCACGCGGAGCGCGCCGCUUGGAUGGCAGAAGCGUUG
UGGGCCUGCGUAGUUCGAGCUGCCGACGGCGAGAGCCUCGCACAGGUUGCGGGGAUGAAC
CGGCGGAGUUCGGAUGAUCGCCGAGGCCAUGGAACACAACCCACCUCAGCACGCUUGGUA
ACCGGGUACUCCGUGCUAGCGGGCGGUGAAGUCGUCGAAGGACGGCAGCGCCGCCCGCUU
CGAUGU
 
                 
                
             
            
             
                
                     
                         
                         
                             TurboFold 
                             
                              ? 
                                 
Visualisation of predicted secondary structure.
To save the image:
  Right click on the image -> Save Image as. 
                             
                         
                     
                
                     
                         
                         
                             rfam-Rc 
                             
                              ? 
                                 
Visualisation of predicted secondary structure.
To save the image:
  Right click on the image -> Save Image as. 
                             
                         
                     
                
                     
                         
                         
                             rnafold 
                             
                              ? 
                                 
Visualisation of predicted secondary structure.
To save the image:
  Right click on the image -> Save Image as. 
                             
                         
                     
                
             
            

        
             
                 Load Sequence viewer 
             
        
        
         
     
    

    
     
         
            Hit: LN868939.1
         
         
             
                 
                     LN868939.1 Nocardia farcinica genome assembly NCTC11134, plasmid : 2 
                     
                         
 ?  
This is BLAST alignment as read from the input file 
                         
 Score = 110.0 bits (100.5), Expect = 3.55E-17
 Identities = 189/264 (72%), Gaps = 17/264 (6%)
 Strand = Plus/Plus
Query     15 AGGCCAAGACCCAGCCGGAAGAGAAGGCTAGATCTCCCGACCCAAGCTCCTAGCACGGATACCG 78    
             |||||||| |  | |||||||||||||  |  ||||||||||     ||| ||||||||  ||        
Sbjct 391854 AGGCCAAGGCGGAACCGGAAGAGAAGGTCACGTCTCCCGACCACCTTTCCCAGCACGGAGGCCA 391917

Query     79 AGCACCCACGCGGAGCACATGCCGC-GGAATAGGCAAAAGTGTTGCGGACCTGCGTAGTTTCGA 141   
              | ||||||||||||| |  |||||  | || |||| ||| |||| || ||||||||| |||||       
Sbjct 391918 GGTACCCACGCGGAGCGC--GCCGCTTGGAT-GGCAGAAGCGTTGTGGGCCTGCGTAG-TTCGA 391977

Query    142 AAAGCGGACGGCCACGACGGCCCTTTGG--GTGGGGTTGCAGCCGTAGCGCA--TCGCAAAGAC 201   
                || |||||| |   |  | |   ||  | |||| || | |||  ||| |  ||| |    |       
Sbjct 391978 GCTGCCGACGGCGAGAGCCTCGCACAGGTTGCGGGGATG-AACCG--GCGGAGTTCGGATGATC 392038

Query    202 GCCGAGGTCA-CCCACGCAACCCACATC-GCACGCTTGGTCACTCGGG--GTCCGTGCTAGCGG 261   
             ||||||| ||    || |||||||| || ||||||||||| || ||||   |||||||||||||       
Sbjct 392039 GCCGAGGCCATGGAACACAACCCACCTCAGCACGCTTGGTAAC-CGGGTACTCCGTGCTAGCGG 392101

Query    262 GCGGCGAA 269   
             |||| |||       
Sbjct 392102 GCGGTGAA 392109

 
                 
                
                 
                      Report:  
                     
                         
                             sequence start
                                   ?  
                                     
Start position of the estimated full-length sequence in genome.
Start index  
                                 :
                             
                             391840 
                         
                         
                             sequence end
                                   ?  
                                     
End position of the estimated full-length sequence in genome.
Start index  
                                 :
                             
                             392145 
                         
                         
                             bit score (CM)
                                   ?  
                                     
The score for aligning estimated full-length sequence to CM model
  (computed by RSEARCH -> default,
  infered from Rfam or provided by user) 
                                 :
                             
                             135.94 
                         
                         
                             Homology estimate
                                   ?  
                                     
Quick homology estimate:
  Not homologous: bit score   20 and bit score > 0.5 * query length
  Uncertain otherwise 
                                 :
                             
                             Uncertain 
                         
                     
                 
                
                
                 
                     
                          Estimated full-length sequence:  
                         
                          ? 
                             
Click checkbox to select multiple seuqences.
Fasta header format:
  UID|accession.versionSTRAND start-end 
                         
                     
                     >uid:14|LN868939.1fw 391840-392145
UACGGAAAGGCGGAAGGCCAAGGCGGAACCGGAAGAGAAGGUCACGUCUCCCGACCACCU
UUCCCAGCACGGAGGCCAGGUACCCACGCGGAGCGCGCCGCUUGGAUGGCAGAAGCGUUG
UGGGCCUGCGUAGUUCGAGCUGCCGACGGCGAGAGCCUCGCACAGGUUGCGGGGAUGAAC
CGGCGGAGUUCGGAUGAUCGCCGAGGCCAUGGAACACAACCCACCUCAGCACGCUUGGUA
ACCGGGUACUCCGUGCUAGCGGGCGGUGAAGUCGUCGAAGGACGGCAGCGCCGCCCGCUU
CGAUGU
 
                 
                
             
            
             
                
                     
                         
                         
                             TurboFold 
                             
                              ? 
                                 
Visualisation of predicted secondary structure.
To save the image:
  Right click on the image -> Save Image as. 
                             
                         
                     
                
                     
                         
                         
                             rfam-Rc 
                             
                              ? 
                                 
Visualisation of predicted secondary structure.
To save the image:
  Right click on the image -> Save Image as. 
                             
                         
                     
                
                     
                         
                         
                             rnafold 
                             
                              ? 
                                 
Visualisation of predicted secondary structure.
To save the image:
  Right click on the image -> Save Image as. 
                             
                         
                     
                
             
            

        
             
                 Load Sequence viewer 
             
        
        
         
     
    

    
     
         
            Hit: AP006618.1
         
         
             
                 
                     AP006618.1 Nocardia farcinica IFM 10152 DNA, complete genome 
                     
                         
 ?  
This is BLAST alignment as read from the input file 
                         
 Score = 110.0 bits (100.5), Expect = 3.55E-17
 Identities = 189/264 (72%), Gaps = 17/264 (6%)
 Strand = Plus/Minus
Query     15 AGGCCAAGACCCAGCCGGAAGAGAAGGCTAGATCTCCCGACCCAAGCTCCTAGCACGGATACCG 78    
             |||||||| |  | |||||||||||||  |  ||||||||||     ||| ||||||||  ||        
Sbjct 367388 AGGCCAAGGCGGAACCGGAAGAGAAGGTCACGTCTCCCGACCACCTTTCCCAGCACGGAGGCCA 367325

Query     79 AGCACCCACGCGGAGCACATGCCGC-GGAATAGGCAAAAGTGTTGCGGACCTGCGTAGTTTCGA 141   
              | ||||||||||||| |  |||||  | || |||| ||| |||| || ||||||||| |||||       
Sbjct 367324 GGTACCCACGCGGAGCGC--GCCGCTTGGAT-GGCAGAAGCGTTGTGGGCCTGCGTAG-TTCGA 367265

Query    142 AAAGCGGACGGCCACGACGGCCCTTTGG--GTGGGGTTGCAGCCGTAGCGCA--TCGCAAAGAC 201   
                || |||||| |   |  | |   ||  | |||| || | |||  ||| |  ||| |    |       
Sbjct 367264 GCTGCCGACGGCGAGAGCCTCGCACAGGTTGCGGGGATG-AACCG--GCGGAGTTCGGATGATC 367204

Query    202 GCCGAGGTCA-CCCACGCAACCCACATC-GCACGCTTGGTCACTCGGG--GTCCGTGCTAGCGG 261   
             ||||||| ||    || |||||||| || ||||||||||| || ||||   |||||||||||||       
Sbjct 367203 GCCGAGGCCATGGAACACAACCCACCTCAGCACGCTTGGTAAC-CGGGTACTCCGTGCTAGCGG 367141

Query    262 GCGGCGAA 269   
             |||| |||       
Sbjct 367140 GCGGTGAA 367133

 
                 
                
                 
                      Report:  
                     
                         
                             sequence start
                                   ?  
                                     
Start position of the estimated full-length sequence in genome.
Start index  
                                 :
                             
                             367098 
                         
                         
                             sequence end
                                   ?  
                                     
End position of the estimated full-length sequence in genome.
Start index  
                                 :
                             
                             367403 
                         
                         
                             bit score (CM)
                                   ?  
                                     
The score for aligning estimated full-length sequence to CM model
  (computed by RSEARCH -> default,
  infered from Rfam or provided by user) 
                                 :
                             
                             135.94 
                         
                         
                             Homology estimate
                                   ?  
                                     
Quick homology estimate:
  Not homologous: bit score   20 and bit score > 0.5 * query length
  Uncertain otherwise 
                                 :
                             
                             Uncertain 
                         
                     
                 
                
                
                 
                     
                          Estimated full-length sequence:  
                         
                          ? 
                             
Click checkbox to select multiple seuqences.
Fasta header format:
  UID|accession.versionSTRAND start-end 
                         
                     
                     >uid:15|AP006618.1rc 367098-367403
UACGGAAAGGCGGAAGGCCAAGGCGGAACCGGAAGAGAAGGUCACGUCUCCCGACCACCU
UUCCCAGCACGGAGGCCAGGUACCCACGCGGAGCGCGCCGCUUGGAUGGCAGAAGCGUUG
UGGGCCUGCGUAGUUCGAGCUGCCGACGGCGAGAGCCUCGCACAGGUUGCGGGGAUGAAC
CGGCGGAGUUCGGAUGAUCGCCGAGGCCAUGGAACACAACCCACCUCAGCACGCUUGGUA
ACCGGGUACUCCGUGCUAGCGGGCGGUGAAGUCGUCGAAGGACGGCAGCGCCGCCCGCUU
CGAUGU
 
                 
                
             
            
             
                
                     
                         
                         
                             TurboFold 
                             
                              ? 
                                 
Visualisation of predicted secondary structure.
To save the image:
  Right click on the image -> Save Image as. 
                             
                         
                     
                
                     
                         
                         
                             rfam-Rc 
                             
                              ? 
                                 
Visualisation of predicted secondary structure.
To save the image:
  Right click on the image -> Save Image as. 
                             
                         
                     
                
                     
                         
                         
                             rnafold 
                             
                              ? 
                                 
Visualisation of predicted secondary structure.
To save the image:
  Right click on the image -> Save Image as. 
                             
                         
                     
                
             
            

        
             
                 Load Sequence viewer 
             
        
        
         
     
    

    
     
         
            Hit: LR215973.1
         
         
             
                 
                     LR215973.1 Nocardia cyriacigeorgica strain 3012STDY6756504 genome assembly, chromosome: 1 
                     
                         
 ?  
This is BLAST alignment as read from the input file 
                         
 Score = 109.0 bits (99.6), Expect = 1.24E-16
 Identities = 186/261 (71%), Gaps = 12/261 (5%)
 Strand = Plus/Minus
Query      15 AGGCCAAGACCCAGCCGGAAGAGAAGGCTAGATCTCCCGACCCAAGC-TCCTAGCACGGATA 75     
              |||||| |||  || ||||||||||||||    | |||  ||||  | ||| ||||||||          
Sbjct 5134570 AGGCCAGGACGGAGTCGGAAGAGAAGGCTCAGCCCCCCATCCCACCCTTCCCAGCACGGACG 5134509

Query      76 CCGAGCACCCACGCGGAGCACATGCCGCGGAATAGGCAAAAGTGTTGCGGACCTGCGTAGTT 137    
              ||  | ||||||||||||| |  ||||||  |  |||||||| |||| || |||||| |  |        
Sbjct 5134508 CCAGGTACCCACGCGGAGCGC--GCCGCGACAAGGGCAAAAGCGTTGTGGGCCTGCG-ACAT 5134450

Query     138 TCGAAAAGCGGACGGCCA-CGACGGCCCTTTGGGTGGGGTTGCAGCCGTAG-CGCATCGCA- 196    
              ||||   || |||||| | || |   | |    | |||| || |||||  |  | |||| |         
Sbjct 5134449 TCGAGCTGCCGACGGCGAGCGCCTCGCATAATTGCGGGGATG-AGCCGCGGTGGAATCGGAT 5134389

Query     197 AAGACGCCGAGGTC-ACCCACGCAACCCACATCGCACGCTTGGTCACTCGGG--GTCCGTGC 255    
               |  |||||||| | |   || ||||||||   ||||||||||| || ||||  ||||||||        
Sbjct 5134388 CACTCGCCGAGGCCGAAGAACACAACCCACTCAGCACGCTTGGTAAC-CGGGTTGTCCGTGC 5134328

Query     256 TAGCGGGCGGCGA 268    
              |||||||||| ||        
Sbjct 5134327 TAGCGGGCGGTGA 5134315

 
                 
                
                 
                      Report:  
                     
                         
                             sequence start
                                   ?  
                                     
Start position of the estimated full-length sequence in genome.
Start index  
                                 :
                             
                             5134280 
                         
                         
                             sequence end
                                   ?  
                                     
End position of the estimated full-length sequence in genome.
Start index  
                                 :
                             
                             5134581 
                         
                         
                             bit score (CM)
                                   ?  
                                     
The score for aligning estimated full-length sequence to CM model
  (computed by RSEARCH -> default,
  infered from Rfam or provided by user) 
                                 :
                             
                             140.38 
                         
                         
                             Homology estimate
                                   ?  
                                     
Quick homology estimate:
  Not homologous: bit score   20 and bit score > 0.5 * query length
  Uncertain otherwise 
                                 :
                             
                             Uncertain 
                         
                     
                 
                
                
                 
                     
                          Estimated full-length sequence:  
                         
                          ? 
                             
Click checkbox to select multiple seuqences.
Fasta header format:
  UID|accession.versionSTRAND start-end 
                         
                     
                     >uid:16|LR215973.1rc 5134280-5134581
ACGGAAGGACGGAAGGCCAGGACGGAGUCGGAAGAGAAGGCUCAGCCCCCCAUCCCACCC
UUCCCAGCACGGACGCCAGGUACCCACGCGGAGCGCGCCGCGACAAGGGCAAAAGCGUUG
UGGGCCUGCGACAUUCGAGCUGCCGACGGCGAGCGCCUCGCAUAAUUGCGGGGAUGAGCC
GCGGUGGAAUCGGAUCACUCGCCGAGGCCGAAGAACACAACCCACUCAGCACGCUUGGUA
ACCGGGUUGUCCGUGCUAGCGGGCGGUGAGGUCGGUGACGACAACGCCGCCCGCUUCGAU
AU
 
                 
                
             
            
             
                
                     
                         
                         
                             TurboFold 
                             
                              ? 
                                 
Visualisation of predicted secondary structure.
To save the image:
  Right click on the image -> Save Image as. 
                             
                         
                     
                
                     
                         
                         
                             rfam-Rc 
                             
                              ? 
                                 
Visualisation of predicted secondary structure.
To save the image:
  Right click on the image -> Save Image as. 
                             
                         
                     
                
                     
                         
                         
                             rnafold 
                             
                              ? 
                                 
Visualisation of predicted secondary structure.
To save the image:
  Right click on the image -> Save Image as. 
                             
                         
                     
                
             
            

        
             
                 Load Sequence viewer 
             
        
        
         
     
    

    
     
         
            Hit: CP017014.1
         
         
             
                 
                     CP017014.1 Rhodococcus sp. WMMA185, complete genome 
                     
                         
 ?  
This is BLAST alignment as read from the input file 
                         
 Score = 108.0 bits (98.7), Expect = 1.24E-16
 Identities = 195/285 (68%), Gaps = 13/285 (5%)
 Strand = Plus/Plus
Query      15 AGGCCAAGACCCAGCCGGAAGAGAAGGCTAGATCTCCCGACCCAAGCTCCTAGCACGGATAC 76     
              ||||||| | | | ||||||||||||| | ||| ||||||||     |||||||||||  ||        
Sbjct 3561730 AGGCCAAAATCGACCCGGAAGAGAAGGATCGATTTCCCGACCAGGATTCCTAGCACGGGCAC 3561791

Query      77 CGAGCACCCACGCGGAGCACATGCCGCGGAATAGGCAAAAGTGTTGCGGACCTGCGTAGTTT 138    
                 | |||||||||||||||| ||| |   |  ||| |||| |||| || |||||| ||  |        
Sbjct 3561792 TCGGTACCCACGCGGAGCACAAGCCACTATAAGGGCGAAAGCGTTGTGGGCCTGCG-AGACT 3561852

Query     139 CGAAAAGCGGACGGCCACGACGGCCCTTTGGGTGGGGTTGCAG-----CCGTAGCGCATCGC 195    
              || |   ||  |||| | ||    |||   |    ||||||||     ||  |  ||  ||         
Sbjct 3561853 CG-AGTTCGATCGGCAAGGA---ACCT---GCACCGGTTGCAGGACGACCCGACGGCTACGA 3561907

Query     196 AAAGACGCCGAGGTCACCCACGCAACCCACATCGCACGCTTGGTCACTCGGGGTCCGTGCTA 257    
              |    |||||||| ||  ||| ||||||||   ||||||||||| ||  |  |||||||||         
Sbjct 3561908 ACTCTCGCCGAGGCCAGTCACACAACCCACCGAGCACGCTTGGTAACCAGTTGTCCGTGCTG 3561969

Query     258 GCGGGCGGCGAACCGGACATTGTCCGGGACGCCGCCC 294    
                |||||||||| | ||| | ||| |   ||||||||        
Sbjct 3561970 ATGGGCGGCGAAGCTGACGTAGTCGGTAGCGCCGCCC 3562006

 
                 
                
                 
                      Report:  
                     
                         
                             sequence start
                                   ?  
                                     
Start position of the estimated full-length sequence in genome.
Start index  
                                 :
                             
                             3561717 
                         
                         
                             sequence end
                                   ?  
                                     
End position of the estimated full-length sequence in genome.
Start index  
                                 :
                             
                             3562016 
                         
                         
                             bit score (CM)
                                   ?  
                                     
The score for aligning estimated full-length sequence to CM model
  (computed by RSEARCH -> default,
  infered from Rfam or provided by user) 
                                 :
                             
                             153.91 
                         
                         
                             Homology estimate
                                   ?  
                                     
Quick homology estimate:
  Not homologous: bit score   20 and bit score > 0.5 * query length
  Uncertain otherwise 
                                 :
                             
                             Homologous 
                         
                     
                 
                
                
                 
                     
                          Estimated full-length sequence:  
                         
                          ? 
                             
Click checkbox to select multiple seuqences.
Fasta header format:
  UID|accession.versionSTRAND start-end 
                         
                     
                     >uid:17|CP017014.1fw 3561717-3562016
ACGGAAGAACGGAAGGCCAAAAUCGACCCGGAAGAGAAGGAUCGAUUUCCCGACCAGGAU
UCCUAGCACGGGCACUCGGUACCCACGCGGAGCACAAGCCACUAUAAGGGCGAAAGCGUU
GUGGGCCUGCGAGACUCGAGUUCGAUCGGCAAGGAACCUGCACCGGUUGCAGGACGACCC
GACGGCUACGAACUCUCGCCGAGGCCAGUCACACAACCCACCGAGCACGCUUGGUAACCA
GUUGUCCGUGCUGAUGGGCGGCGAAGCUGACGUAGUCGGUAGCGCCGCCCUCGUUGCGUC
 
                 
                
             
            
             
                
                     
                         
                         
                             TurboFold 
                             
                              ? 
                                 
Visualisation of predicted secondary structure.
To save the image:
  Right click on the image -> Save Image as. 
                             
                         
                     
                
                     
                         
                         
                             rfam-Rc 
                             
                              ? 
                                 
Visualisation of predicted secondary structure.
To save the image:
  Right click on the image -> Save Image as. 
                             
                         
                     
                
                     
                         
                         
                             rnafold 
                             
                              ? 
                                 
Visualisation of predicted secondary structure.
To save the image:
  Right click on the image -> Save Image as. 
                             
                         
                     
                
             
            

        
             
                 Load Sequence viewer 
             
        
        
         
     
    

    
     
         
            Hit: CP006850.1
         
         
             
                 
                     CP006850.1 Nocardia nova SH22a, complete genome 
                     
                         
 ?  
This is BLAST alignment as read from the input file 
                         
 Score = 106.0 bits (96.9), Expect = 4.32E-16
 Identities = 214/307 (70%), Gaps = 22/307 (7%)
 Strand = Plus/Minus
Query      1 ACGGAAGCTTGGCG--AGGCCAAGACCCAGCCGGAAGAGAAGGCTAGATCTCCCGACCCAAGCT 62    
             |||||||   ||||  ||||||||| | |  |||||||||||| | ||||||||| ||  |  |       
Sbjct 431367 ACGGAAG---GGCGGAAGGCCAAGATCGAAACGGAAGAGAAGGTTCGATCTCCCGGCCGCACTT 431307

Query     63 CCTAGCACGGATACCGAGCACCCACGCGGAGCACATGCCGCGGAATAGGCAAAAGTGTTGCGGA 126   
             || |||||||| |||  ||||||||||||||| |  ||||||  |  |||| ||| |||| ||        
Sbjct 431306 CCCAGCACGGACACCAGGCACCCACGCGGAGCGC--GCCGCGACAAGGGCAGAAGCGTTGTGGG 431245

Query    127 CCTGCGTAGTTTCGAAAAGCGGACGGCCACGACGGCCCTTTGG--GTGGGGTTGCAGCCGTAGC 188   
             |||||| |  |||| |       |||| | | |  | |   ||  | |||| || | |||  ||       
Sbjct 431244 CCTGCG-AAATTCGCATTCACCTCGGCAATGGCCTCGCACAGGTTGCGGGGATG-AACCG--GC 431185

Query    189 GCATCGC-AAAGA-CGCCGAGGTCAC-CCACGCAACCCACATCGCACGCTTGGTCACTCGGG-- 247   
             | |  ||  | || |||||||| | |   || ||||||     ||||||||||| || ||||         
Sbjct 431184 GGAGTGCGGATGATCGCCGAGGCCGCAGAACACAACCCGACCAGCACGCTTGGTAAC-CGGGCA 431122

Query    248 GTCCGTGCTAGCGGGCGGCGAACCGGACATTGTCCGGGACGCCGCCCGATT 298   
             |||||||||||||||||| ||  | |||   | | |  |||||||||| ||       
Sbjct 431121 GTCCGTGCTAGCGGGCGGTGAGGCCGAC---GACGGCTACGCCGCCCGCTT 431074

 
                 
                
                 
                      Report:  
                     
                         
                             sequence start
                                   ?  
                                     
Start position of the estimated full-length sequence in genome.
Start index  
                                 :
                             
                             431068 
                         
                         
                             sequence end
                                   ?  
                                     
End position of the estimated full-length sequence in genome.
Start index  
                                 :
                             
                             431367 
                         
                         
                             bit score (CM)
                                   ?  
                                     
The score for aligning estimated full-length sequence to CM model
  (computed by RSEARCH -> default,
  infered from Rfam or provided by user) 
                                 :
                             
                             158.47 
                         
                         
                             Homology estimate
                                   ?  
                                     
Quick homology estimate:
  Not homologous: bit score   20 and bit score > 0.5 * query length
  Uncertain otherwise 
                                 :
                             
                             Homologous 
                         
                     
                 
                
                
                 
                     
                          Estimated full-length sequence:  
                         
                          ? 
                             
Click checkbox to select multiple seuqences.
Fasta header format:
  UID|accession.versionSTRAND start-end 
                         
                     
                     >uid:18|CP006850.1rc 431068-431367
ACGGAAGGGCGGAAGGCCAAGAUCGAAACGGAAGAGAAGGUUCGAUCUCCCGGCCGCACU
UCCCAGCACGGACACCAGGCACCCACGCGGAGCGCGCCGCGACAAGGGCAGAAGCGUUGU
GGGCCUGCGAAAUUCGCAUUCACCUCGGCAAUGGCCUCGCACAGGUUGCGGGGAUGAACC
GGCGGAGUGCGGAUGAUCGCCGAGGCCGCAGAACACAACCCGACCAGCACGCUUGGUAAC
CGGGCAGUCCGUGCUAGCGGGCGGUGAGGCCGACGACGGCUACGCCGCCCGCUUCGACGU
 
                 
                
             
            
             
                
                     
                         
                         
                             TurboFold 
                             
                              ? 
                                 
Visualisation of predicted secondary structure.
To save the image:
  Right click on the image -> Save Image as. 
                             
                         
                     
                
                     
                         
                         
                             rfam-Rc 
                             
                              ? 
                                 
Visualisation of predicted secondary structure.
To save the image:
  Right click on the image -> Save Image as. 
                             
                         
                     
                
                     
                         
                         
                             rnafold 
                             
                              ? 
                                 
Visualisation of predicted secondary structure.
To save the image:
  Right click on the image -> Save Image as. 
                             
                         
                     
                
             
            

        
             
                 Load Sequence viewer 
             
        
        
         
     
    

    
     
         
            Hit: CP021354.1
         
         
             
                 
                     CP021354.1 Rhodococcus sp. S2-17 chromosome, complete genome 
                     
                         
 ?  
This is BLAST alignment as read from the input file 
                         
 Score = 105.0 bits (96.0), Expect = 1.51E-15
 Identities = 197/287 (69%), Gaps = 6/287 (2%)
 Strand = Plus/Minus
Query     15 AGGCCAAGACCCAGCCGGAAGAGAAGGCTAGATCTCCCGACCCAAGCTCCTAGCACGGATACCG 78    
             ||||||||| | | ||||||||||||| | ||||||||||||     |||  ||||||  ||         
Sbjct 676927 AGGCCAAGATCGACCCGGAAGAGAAGGATCGATCTCCCGACCGCGATTCCCGGCACGGGCACTA 676864

Query     79 AGCACCCACGCGGAGCACATGCCGCGGAATAGGCAAAAGTGTTGCGGACCTGCGTAGTTTCGAA 142   
              | |||||||||||||||| ||| |   |  |||| |||||||| || |||||| ||  |||         
Sbjct 676863 GGTACCCACGCGGAGCACAAGCCACTATAAGGGCAGAAGTGTTGTGGGCCTGCG-AGACTCGCG 676801

Query    143 AAGCGGACGGCCACGACGGCCCTTTGGGTG--GGGTTGCAGCCGTAGCGCATCGCAAAGACGCC 204   
                |   |||| | |||    |   || ||  |||  | | |||  |  | ||| |    ||||       
Sbjct 676800 TTTC-ATCGGCAAGGACCCTGCACCGGTTGCAGGGACG-ACCCGACGATCGTCGAACTCTCGCC 676739

Query    205 GAGGTCACCCACGCAACCCACATCGCACGCTTGGTCACTCGGGGTCCGTGCTAGCGGGCGGCGA 268   
             |||| || |||| ||||||||   ||||||||||| ||  |  ||||| |||  ||||||||||       
Sbjct 676738 GAGGCCATCCACACAACCCACCGAGCACGCTTGGTAACCGGTAGTCCGCGCTGACGGGCGGCGA 676675

Query    269 ACCGGAC-ATTGTCCGGGACGCCGCCCGATT 298   
             |   ||| |  ||| |   ||||||||| ||       
Sbjct 676674 AGTCGACGAGAGTCGGCAGCGCCGCCCGTTT 676644

 
                 
                
                 
                      Report:  
                     
                         
                             sequence start
                                   ?  
                                     
Start position of the estimated full-length sequence in genome.
Start index  
                                 :
                             
                             676639 
                         
                         
                             sequence end
                                   ?  
                                     
End position of the estimated full-length sequence in genome.
Start index  
                                 :
                             
                             676941 
                         
                         
                             bit score (CM)
                                   ?  
                                     
The score for aligning estimated full-length sequence to CM model
  (computed by RSEARCH -> default,
  infered from Rfam or provided by user) 
                                 :
                             
                             156.32 
                         
                         
                             Homology estimate
                                   ?  
                                     
Quick homology estimate:
  Not homologous: bit score   20 and bit score > 0.5 * query length
  Uncertain otherwise 
                                 :
                             
                             Homologous 
                         
                     
                 
                
                
                 
                     
                          Estimated full-length sequence:  
                         
                          ? 
                             
Click checkbox to select multiple seuqences.
Fasta header format:
  UID|accession.versionSTRAND start-end 
                         
                     
                     >uid:19|CP021354.1rc 676639-676941
ACGGAAGAACGGAAGGCCAAGAUCGACCCGGAAGAGAAGGAUCGAUCUCCCGACCGCGAU
UCCCGGCACGGGCACUAGGUACCCACGCGGAGCACAAGCCACUAUAAGGGCAGAAGUGUU
GUGGGCCUGCGAGACUCGCGUUUCAUCGGCAAGGACCCUGCACCGGUUGCAGGGACGACC
CGACGAUCGUCGAACUCUCGCCGAGGCCAUCCACACAACCCACCGAGCACGCUUGGUAAC
CGGUAGUCCGCGCUGACGGGCGGCGAAGUCGACGAGAGUCGGCAGCGCCGCCCGUUUCGU
GUG
 
                 
                
             
            
             
                
                     
                         
                         
                             TurboFold 
                             
                              ? 
                                 
Visualisation of predicted secondary structure.
To save the image:
  Right click on the image -> Save Image as. 
                             
                         
                     
                
                     
                         
                         
                             rfam-Rc 
                             
                              ? 
                                 
Visualisation of predicted secondary structure.
To save the image:
  Right click on the image -> Save Image as. 
                             
                         
                     
                
                     
                         
                         
                             rnafold 
                             
                              ? 
                                 
Visualisation of predicted secondary structure.
To save the image:
  Right click on the image -> Save Image as. 
                             
                         
                     
                
             
            

        
             
                 Load Sequence viewer 
             
        
        
         
     
    

    
     
         
            Hit: CP041769.1
         
         
             
                 
                     CP041769.1 Rhodococcus sp. WB9 chromosome, complete genome 
                     
                         
 ?  
This is BLAST alignment as read from the input file 
                         
 Score = 104.0 bits (95.1), Expect = 1.51E-15
 Identities = 196/287 (68%), Gaps = 5/287 (2%)
 Strand = Plus/Minus
Query     15 AGGCCAAGACCCAGCCGGAAGAGAAGGCTAGATCTCCCGACCCAAGCTCCTAGCACGGATACCG 78    
             ||||||||  | | || |||||||||| | ||||||||||||     ||| |||||||  ||         
Sbjct 813715 AGGCCAAGGTCGACCCAGAAGAGAAGGATCGATCTCCCGACCAGGATTCCCAGCACGGGCACTA 813652

Query     79 AGCACCCACGCGGAGCACATGCCGCGGAATAGGCAAAAGTGTTGCGGACCTGCGTAGTTTCGAA 142   
              | ||||||||| |||| | ||| |   |  |||| ||| |||| || |||||| ||  ||||        
Sbjct 813651 GGTACCCACGCGAAGCATAAGCCACTATAAGGGCAGAAGCGTTGTGGGCCTGCG-AGACTCGAG 813589

Query    143 AAGCGGACGGCCACGACGGCCCTTTGGGTGG--GGTTGCAGCCGTAGCGCATCGCAAAGACGCC 204   
                    |||| | |||    |   || ||   ||  | | |||  |    ||| |    ||||       
Sbjct 813588 TCTTCACCGGCAAGGACCCTGCACAGGTTGCAAGGACG-ACCCGACGATATTCGGACTCTCGCC 813526

Query    205 GAGGTCACCCACGCAACCCACATCGCACGCTTGGTCACTCGGGGTCCGTGCTAGCGGGCGGCGA 268   
             |||| ||||||| |||||||||| ||||||||||| ||  |  |||||||||| ||||||||||       
Sbjct 813525 GAGGCCACCCACACAACCCACATTGCACGCTTGGTAACCGGTTGTCCGTGCTAACGGGCGGCGA 813462

Query    269 ACCGGACA-TTGTCCGGGACGCCGCCCGATT 298   
             |   ||||   ||| |   ||||||||| ||       
Sbjct 813461 AGTCGACAGCAGTCGGCAGCGCCGCCCGTTT 813431

 
                 
                
                 
                      Report:  
                     
                         
                             sequence start
                                   ?  
                                     
Start position of the estimated full-length sequence in genome.
Start index  
                                 :
                             
                             813426 
                         
                         
                             sequence end
                                   ?  
                                     
End position of the estimated full-length sequence in genome.
Start index  
                                 :
                             
                             813728 
                         
                         
                             bit score (CM)
                                   ?  
                                     
The score for aligning estimated full-length sequence to CM model
  (computed by RSEARCH -> default,
  infered from Rfam or provided by user) 
                                 :
                             
                             152.13 
                         
                         
                             Homology estimate
                                   ?  
                                     
Quick homology estimate:
  Not homologous: bit score   20 and bit score > 0.5 * query length
  Uncertain otherwise 
                                 :
                             
                             Homologous 
                         
                     
                 
                
                
                 
                     
                          Estimated full-length sequence:  
                         
                          ? 
                             
Click checkbox to select multiple seuqences.
Fasta header format:
  UID|accession.versionSTRAND start-end 
                         
                     
                     >uid:20|CP041769.1rc 813426-813728
ACGGAAGAACGGAAGGCCAAGGUCGACCCAGAAGAGAAGGAUCGAUCUCCCGACCAGGAU
UCCCAGCACGGGCACUAGGUACCCACGCGAAGCAUAAGCCACUAUAAGGGCAGAAGCGUU
GUGGGCCUGCGAGACUCGAGUCUUCACCGGCAAGGACCCUGCACAGGUUGCAAGGACGAC
CCGACGAUAUUCGGACUCUCGCCGAGGCCACCCACACAACCCACAUUGCACGCUUGGUAA
CCGGUUGUCCGUGCUAACGGGCGGCGAAGUCGACAGCAGUCGGCAGCGCCGCCCGUUUCA
CAU
 
                 
                
             
            
             
                
                     
                         
                         
                             TurboFold 
                             
                              ? 
                                 
Visualisation of predicted secondary structure.
To save the image:
  Right click on the image -> Save Image as. 
                             
                         
                     
                
                     
                         
                         
                             rfam-Rc 
                             
                              ? 
                                 
Visualisation of predicted secondary structure.
To save the image:
  Right click on the image -> Save Image as. 
                             
                         
                     
                
                     
                         
                         
                             rnafold 
                             
                              ? 
                                 
Visualisation of predicted secondary structure.
To save the image:
  Right click on the image -> Save Image as. 
                             
                         
                     
                
             
            

        
             
                 Load Sequence viewer 
             
        
        
         
     
    

    
     
         
            Hit: CP027793.1
         
         
             
                 
                     CP027793.1 Rhodococcus hoagii strain DSSKP-R-001 chromosome, complete genome 
                     
                         
 ?  
This is BLAST alignment as read from the input file 
                         
 Score = 104.0 bits (95.1), Expect = 1.51E-15
 Identities = 177/256 (69%), Gaps = 7/256 (3%)
 Strand = Plus/Minus
Query     17 GCCAAGACCCAGCCGGAAGAGAAGGCTAGATCTCCCGACCCAAGCTCCTAGCACGGATACCGAG 80    
             |||| || | | |||||||||||||   |||  |||||||  |  ||| ||||||| ||||  |       
Sbjct 624372 GCCAGGATCGATCCGGAAGAGAAGGACCGATTCCCCGACCGGACTTCCCAGCACGGGTACCAGG 624309

Query     81 CACCCACGCGGAGCACATGCCGCGGAATAGGCAAAAGTGTTGCGGACCTGCGTAGTTTCGAAAA 144   
              |||||||||||||||| ||| |   |  |||| ||| |||| || | ||||| |  ||| |         
Sbjct 624308 TACCCACGCGGAGCACAAGCCACTATAAGGGCAGAAGCGTTGTGGGCTTGCGT-GACTCGGATG 624246

Query    145 GCGGACGGCCACGAC---GGCCCTTTGGGTGGGGTTGCAGCCGTAGCGCATCGCAAAGACGCCG 205   
              | |||||| | |||   |  |   | |   |||  | | | | | |||  ||  |  ||||||       
Sbjct 624245 ACTGACGGCAAGGACCCTGCACACGTTGCACGGGACGAACCGGCACCGC-CCGAGACCACGCCG 624183

Query    206 AGGTC--ACCCACGCAACCCACATCGCACGCTTGGTCACTCGGGGTCCGTGCTAGCGGGCGGCG 267   
             ||| |     ||| |||||||| | ||||||||||| || |||  |||||||||||||| ||||       
Sbjct 624182 AGGCCGTTGACACACAACCCACCTTGCACGCTTGGTAACCCGGAATCCGTGCTAGCGGGTGGCG 624119

 
                 
                
                 
                      Report:  
                     
                         
                             sequence start
                                   ?  
                                     
Start position of the estimated full-length sequence in genome.
Start index  
                                 :
                             
                             624083 
                         
                         
                             sequence end
                                   ?  
                                     
End position of the estimated full-length sequence in genome.
Start index  
                                 :
                             
                             624389 
                         
                         
                             bit score (CM)
                                   ?  
                                     
The score for aligning estimated full-length sequence to CM model
  (computed by RSEARCH -> default,
  infered from Rfam or provided by user) 
                                 :
                             
                             149.7 
                         
                         
                             Homology estimate
                                   ?  
                                     
Quick homology estimate:
  Not homologous: bit score   20 and bit score > 0.5 * query length
  Uncertain otherwise 
                                 :
                             
                             Uncertain 
                         
                     
                 
                
                
                 
                     
                          Estimated full-length sequence:  
                         
                          ? 
                             
Click checkbox to select multiple seuqences.
Fasta header format:
  UID|accession.versionSTRAND start-end 
                         
                     
                     >uid:21|CP027793.1rc 624083-624389
ACGGAAGGCCGGAAAGCCAGGAUCGAUCCGGAAGAGAAGGACCGAUUCCCCGACCGGACU
UCCCAGCACGGGUACCAGGUACCCACGCGGAGCACAAGCCACUAUAAGGGCAGAAGCGUU
GUGGGCUUGCGUGACUCGGAUGACUGACGGCAAGGACCCUGCACACGUUGCACGGGACGA
ACCGGCACCGCCCGAGACCACGCCGAGGCCGUUGACACACAACCCACCUUGCACGCUUGG
UAACCCGGAAUCCGUGCUAGCGGGUGGCGGCGCCGACCACGGUCGGUGACACCACCCGCU
UCGCAUG
 
                 
                
             
            
             
                
                     
                         
                         
                             TurboFold 
                             
                              ? 
                                 
Visualisation of predicted secondary structure.
To save the image:
  Right click on the image -> Save Image as. 
                             
                         
                     
                
                     
                         
                         
                             rfam-Rc 
                             
                              ? 
                                 
Visualisation of predicted secondary structure.
To save the image:
  Right click on the image -> Save Image as. 
                             
                         
                     
                
                     
                         
                         
                             rnafold 
                             
                              ? 
                                 
Visualisation of predicted secondary structure.
To save the image:
  Right click on the image -> Save Image as. 
                             
                         
                     
                
             
            

        
             
                 Load Sequence viewer 
             
        
        
         
     
    

    
     
         
            Hit: CP009111.1
         
         
             
                 
                     CP009111.1 Rhodococcus opacus strain 1CP, complete genome 
                     
                         
 ?  
This is BLAST alignment as read from the input file 
                         
 Score = 104.0 bits (95.1), Expect = 1.51E-15
 Identities = 196/287 (68%), Gaps = 5/287 (2%)
 Strand = Plus/Minus
Query     15 AGGCCAAGACCCAGCCGGAAGAGAAGGCTAGATCTCCCGACCCAAGCTCCTAGCACGGATACCG 78    
             ||||||||  | | || |||||||||| | ||||||||||||     ||| |||||||  ||         
Sbjct 641373 AGGCCAAGGTCGACCCAGAAGAGAAGGATCGATCTCCCGACCAGGATTCCCAGCACGGGCACTA 641310

Query     79 AGCACCCACGCGGAGCACATGCCGCGGAATAGGCAAAAGTGTTGCGGACCTGCGTAGTTTCGAA 142   
              | ||||||||| |||| | ||| |   |  |||| ||| |||| || |||||| ||  ||||        
Sbjct 641309 GGTACCCACGCGAAGCATAAGCCACTATAAGGGCAGAAGCGTTGTGGGCCTGCG-AGACTCGAG 641247

Query    143 AAGCGGACGGCCACGACGGCCCTTTGGGTGG--GGTTGCAGCCGTAGCGCATCGCAAAGACGCC 204   
                    |||| | |||    |   || ||   ||  | | |||  |    ||| |    ||||       
Sbjct 641246 TCTTCACCGGCAAGGACCCTGCACAGGTTGCAAGGACG-ACCCGACGATATTCGGACTCTCGCC 641184

Query    205 GAGGTCACCCACGCAACCCACATCGCACGCTTGGTCACTCGGGGTCCGTGCTAGCGGGCGGCGA 268   
             |||| ||||||| |||||||||| ||||||||||| ||  |  |||||||||| ||||||||||       
Sbjct 641183 GAGGCCACCCACACAACCCACATTGCACGCTTGGTAACCGGTTGTCCGTGCTAACGGGCGGCGA 641120

Query    269 ACCGGACA-TTGTCCGGGACGCCGCCCGATT 298   
             |   ||||   ||| |   ||||||||| ||       
Sbjct 641119 AGTCGACAGCAGTCGGCAGCGCCGCCCGTTT 641089

 
                 
                
                 
                      Report:  
                     
                         
                             sequence start
                                   ?  
                                     
Start position of the estimated full-length sequence in genome.
Start index  
                                 :
                             
                             641084 
                         
                         
                             sequence end
                                   ?  
                                     
End position of the estimated full-length sequence in genome.
Start index  
                                 :
                             
                             641386 
                         
                         
                             bit score (CM)
                                   ?  
                                     
The score for aligning estimated full-length sequence to CM model
  (computed by RSEARCH -> default,
  infered from Rfam or provided by user) 
                                 :
                             
                             152.13 
                         
                         
                             Homology estimate
                                   ?  
                                     
Quick homology estimate:
  Not homologous: bit score   20 and bit score > 0.5 * query length
  Uncertain otherwise 
                                 :
                             
                             Homologous 
                         
                     
                 
                
                
                 
                     
                          Estimated full-length sequence:  
                         
                          ? 
                             
Click checkbox to select multiple seuqences.
Fasta header format:
  UID|accession.versionSTRAND start-end 
                         
                     
                     >uid:22|CP009111.1rc 641084-641386
ACGGAAGAACGGAAGGCCAAGGUCGACCCAGAAGAGAAGGAUCGAUCUCCCGACCAGGAU
UCCCAGCACGGGCACUAGGUACCCACGCGAAGCAUAAGCCACUAUAAGGGCAGAAGCGUU
GUGGGCCUGCGAGACUCGAGUCUUCACCGGCAAGGACCCUGCACAGGUUGCAAGGACGAC
CCGACGAUAUUCGGACUCUCGCCGAGGCCACCCACACAACCCACAUUGCACGCUUGGUAA
CCGGUUGUCCGUGCUAACGGGCGGCGAAGUCGACAGCAGUCGGCAGCGCCGCCCGUUUCA
CAU
 
                 
                
             
            
             
                
                     
                         
                         
                             TurboFold 
                             
                              ? 
                                 
Visualisation of predicted secondary structure.
To save the image:
  Right click on the image -> Save Image as. 
                             
                         
                     
                
                     
                         
                         
                             rfam-Rc 
                             
                              ? 
                                 
Visualisation of predicted secondary structure.
To save the image:
  Right click on the image -> Save Image as. 
                             
                         
                     
                
                     
                         
                         
                             rnafold 
                             
                              ? 
                                 
Visualisation of predicted secondary structure.
To save the image:
  Right click on the image -> Save Image as. 
                             
                         
                     
                
             
            

        
             
                 Load Sequence viewer 
             
        
        
         
     
    

    
     
         
            Hit: CP003949.1
         
         
             
                 
                     CP003949.1 Rhodococcus opacus PD630, complete genome 
                     
                         
 ?  
This is BLAST alignment as read from the input file 
                         
 Score = 104.0 bits (95.1), Expect = 1.51E-15
 Identities = 196/287 (68%), Gaps = 5/287 (2%)
 Strand = Plus/Minus
Query     15 AGGCCAAGACCCAGCCGGAAGAGAAGGCTAGATCTCCCGACCCAAGCTCCTAGCACGGATACCG 78    
             ||||||||  | | || |||||||||| | ||||||||||||     ||| |||||||  ||         
Sbjct 797082 AGGCCAAGGTCGACCCAGAAGAGAAGGATCGATCTCCCGACCAGGATTCCCAGCACGGGCACTA 797019

Query     79 AGCACCCACGCGGAGCACATGCCGCGGAATAGGCAAAAGTGTTGCGGACCTGCGTAGTTTCGAA 142   
              | ||||||||| |||| | ||| |   |  |||| ||| |||| || |||||| ||  ||||        
Sbjct 797018 GGTACCCACGCGAAGCATAAGCCACTATAAGGGCAGAAGCGTTGTGGGCCTGCG-AGACTCGAG 796956

Query    143 AAGCGGACGGCCACGACGGCCCTTTGGGTGG--GGTTGCAGCCGTAGCGCATCGCAAAGACGCC 204   
                    |||| | |||    |   || ||   ||  | | |||  |    ||| |    ||||       
Sbjct 796955 TCTTCACCGGCAAGGACCCTGCACAGGTTGCAAGGACG-ACCCGACGATATTCGGACTCTCGCC 796893

Query    205 GAGGTCACCCACGCAACCCACATCGCACGCTTGGTCACTCGGGGTCCGTGCTAGCGGGCGGCGA 268   
             |||| ||||||| |||||||||| ||||||||||| ||  |  |||||||||| ||||||||||       
Sbjct 796892 GAGGCCACCCACACAACCCACATTGCACGCTTGGTAACCGGTTGTCCGTGCTAACGGGCGGCGA 796829

Query    269 ACCGGACA-TTGTCCGGGACGCCGCCCGATT 298   
             |   ||||   ||| |   ||||||||| ||       
Sbjct 796828 AGTCGACAGCAGTCGGCAGCGCCGCCCGTTT 796798

 
                 
                
                 
                      Report:  
                     
                         
                             sequence start
                                   ?  
                                     
Start position of the estimated full-length sequence in genome.
Start index  
                                 :
                             
                             796793 
                         
                         
                             sequence end
                                   ?  
                                     
End position of the estimated full-length sequence in genome.
Start index  
                                 :
                             
                             797095 
                         
                         
                             bit score (CM)
                                   ?  
                                     
The score for aligning estimated full-length sequence to CM model
  (computed by RSEARCH -> default,
  infered from Rfam or provided by user) 
                                 :
                             
                             152.13 
                         
                         
                             Homology estimate
                                   ?  
                                     
Quick homology estimate:
  Not homologous: bit score   20 and bit score > 0.5 * query length
  Uncertain otherwise 
                                 :
                             
                             Homologous 
                         
                     
                 
                
                
                 
                     
                          Estimated full-length sequence:  
                         
                          ? 
                             
Click checkbox to select multiple seuqences.
Fasta header format:
  UID|accession.versionSTRAND start-end 
                         
                     
                     >uid:23|CP003949.1rc 796793-797095
ACGGAAGAACGGAAGGCCAAGGUCGACCCAGAAGAGAAGGAUCGAUCUCCCGACCAGGAU
UCCCAGCACGGGCACUAGGUACCCACGCGAAGCAUAAGCCACUAUAAGGGCAGAAGCGUU
GUGGGCCUGCGAGACUCGAGUCUUCACCGGCAAGGACCCUGCACAGGUUGCAAGGACGAC
CCGACGAUAUUCGGACUCUCGCCGAGGCCACCCACACAACCCACAUUGCACGCUUGGUAA
CCGGUUGUCCGUGCUAACGGGCGGCGAAGUCGACAGCAGUCGGCAGCGCCGCCCGUUUCA
CAU
 
                 
                
             
            
             
                
                     
                         
                         
                             TurboFold 
                             
                              ? 
                                 
Visualisation of predicted secondary structure.
To save the image:
  Right click on the image -> Save Image as. 
                             
                         
                     
                
                     
                         
                         
                             rfam-Rc 
                             
                              ? 
                                 
Visualisation of predicted secondary structure.
To save the image:
  Right click on the image -> Save Image as. 
                             
                         
                     
                
                     
                         
                         
                             rnafold 
                             
                              ? 
                                 
Visualisation of predicted secondary structure.
To save the image:
  Right click on the image -> Save Image as. 
                             
                         
                     
                
             
            

        
             
                 Load Sequence viewer 
             
        
        
         
     
    

    
     
         
            Hit: CP032221.1
         
         
             
                 
                     CP032221.1 Rhodococcus rhodochrous strain EP4 chromosome 
                     
                         
 ?  
This is BLAST alignment as read from the input file 
                         
 Score = 103.0 bits (94.2), Expect = 5.27E-15
 Identities = 97/126 (77%), Gaps = 1/126 (1%)
 Strand = Plus/Minus
Query      15 AGGCCAAGACCCAGCCGGAAGAGAAGGCTAGATCTCCCGACCCAAGCTCCTAGCACGGATAC 76     
              ||||||||||| |  |||||||||||  | |||||||||||      ||| |||||||| ||        
Sbjct 2302701 AGGCCAAGACCGAATCGGAAGAGAAGATTCGATCTCCCGACTGTTCTTCCCAGCACGGACAC 2302640

Query      77 CGAGCACCCACGCGGAGCACATGCCGCGGAATAGGCAAAAGTGTTGCGGACCTGCGTAGTTT 138    
              |||| ||||||||| |||  ||||| |   |  |||| |||||||| || |||||| ||||         
Sbjct 2302639 CGAGTACCCACGCGAAGCCGATGCCACTATAAGGGCACAAGTGTTGTGGGCCTGCG-AGTTC 2302579

Query     139 CG 140    
              ||        
Sbjct 2302578 CG 2302577

 
                 
                
                 
                      Report:  
                     
                         
                             sequence start
                                   ?  
                                     
Start position of the estimated full-length sequence in genome.
Start index  
                                 :
                             
                             2302414 
                         
                         
                             sequence end
                                   ?  
                                     
End position of the estimated full-length sequence in genome.
Start index  
                                 :
                             
                             2302721 
                         
                         
                             bit score (CM)
                                   ?  
                                     
The score for aligning estimated full-length sequence to CM model
  (computed by RSEARCH -> default,
  infered from Rfam or provided by user) 
                                 :
                             
                             163.26 
                         
                         
                             Homology estimate
                                   ?  
                                     
Quick homology estimate:
  Not homologous: bit score   20 and bit score > 0.5 * query length
  Uncertain otherwise 
                                 :
                             
                             Homologous 
                         
                     
                 
                
                
                 
                     
                          Estimated full-length sequence:  
                         
                          ? 
                             
Click checkbox to select multiple seuqences.
Fasta header format:
  UID|accession.versionSTRAND start-end 
                         
                     
                     >uid:24|CP032221.1rc 2302414-2302721
ACGGAAGGACGGAAGGCCAAGACCGAAUCGGAAGAGAAGAUUCGAUCUCCCGACUGUUCU
UCCCAGCACGGACACCGAGUACCCACGCGAAGCCGAUGCCACUAUAAGGGCACAAGUGUU
GUGGGCCUGCGAGUUCCGGAUUUUCGAAGACGAGUACCCCGCACCCGUUGCGAGGACGAA
UCGACGAUACCCGGAUCAUCGCCGAGGCCGUUGAAUCACAACCCAUUUCAGCACGCAUGG
UAACUCGGACCGCCCGUGCUAGCGGGUGGCGGAAUCGAUCUCGAUCGAUAUGCCACCCGC
UUUCGCGU
 
                 
                
             
            
             
                
                     
                         
                         
                             TurboFold 
                             
                              ? 
                                 
Visualisation of predicted secondary structure.
To save the image:
  Right click on the image -> Save Image as. 
                             
                         
                     
                
                     
                         
                         
                             rfam-Rc 
                             
                              ? 
                                 
Visualisation of predicted secondary structure.
To save the image:
  Right click on the image -> Save Image as. 
                             
                         
                     
                
                     
                         
                         
                             rnafold 
                             
                              ? 
                                 
Visualisation of predicted secondary structure.
To save the image:
  Right click on the image -> Save Image as. 
                             
                         
                     
                
             
            

        
             
                 Load Sequence viewer 
             
        
        
         
     
    

    
     
         
            Hit: CP032221.1
         
         
             
                 
                     CP032221.1 Rhodococcus rhodochrous strain EP4 chromosome 
                     
                         
 ?  
This is BLAST alignment as read from the input file 
                         
 Score = 49.0 bits (45.5), Expect = 2.39E+00
 Identities = 43/53 (81%), Gaps = 3/53 (6%)
 Strand = Plus/Minus
Query     218 CAACCCACATC-GCACGCTTGGTCACTCGGG--GTCCGTGCTAGCGGGCGGCG 267    
              |||||||  || |||||| |||| ||||||   | ||||||||||||| ||||        
Sbjct 2302496 CAACCCATTTCAGCACGCATGGTAACTCGGACCGCCCGTGCTAGCGGGTGGCG 2302444

 
                 
                
                 
                      Report:  
                     
                         
                             sequence start
                                   ?  
                                     
Start position of the estimated full-length sequence in genome.
Start index  
                                 :
                             
                             2302406 
                         
                         
                             sequence end
                                   ?  
                                     
End position of the estimated full-length sequence in genome.
Start index  
                                 :
                             
                             2302713 
                         
                         
                             bit score (CM)
                                   ?  
                                     
The score for aligning estimated full-length sequence to CM model
  (computed by RSEARCH -> default,
  infered from Rfam or provided by user) 
                                 :
                             
                             163.26 
                         
                         
                             Homology estimate
                                   ?  
                                     
Quick homology estimate:
  Not homologous: bit score   20 and bit score > 0.5 * query length
  Uncertain otherwise 
                                 :
                             
                             Homologous 
                         
                     
                 
                
                
                 
                     
                          Estimated full-length sequence:  
                         
                          ? 
                             
Click checkbox to select multiple seuqences.
Fasta header format:
  UID|accession.versionSTRAND start-end 
                         
                     
                     >uid:25|CP032221.1rc 2302406-2302713
ACGGAAGGACGGAAGGCCAAGACCGAAUCGGAAGAGAAGAUUCGAUCUCCCGACUGUUCU
UCCCAGCACGGACACCGAGUACCCACGCGAAGCCGAUGCCACUAUAAGGGCACAAGUGUU
GUGGGCCUGCGAGUUCCGGAUUUUCGAAGACGAGUACCCCGCACCCGUUGCGAGGACGAA
UCGACGAUACCCGGAUCAUCGCCGAGGCCGUUGAAUCACAACCCAUUUCAGCACGCAUGG
UAACUCGGACCGCCCGUGCUAGCGGGUGGCGGAAUCGAUCUCGAUCGAUAUGCCACCCGC
UUUCGCGU
 
                 
                
             
            
             
                
                     
                         
                         
                             TurboFold 
                             
                              ? 
                                 
Visualisation of predicted secondary structure.
To save the image:
  Right click on the image -> Save Image as. 
                             
                         
                     
                
                     
                         
                         
                             rfam-Rc 
                             
                              ? 
                                 
Visualisation of predicted secondary structure.
To save the image:
  Right click on the image -> Save Image as. 
                             
                         
                     
                
                     
                         
                         
                             rnafold 
                             
                              ? 
                                 
Visualisation of predicted secondary structure.
To save the image:
  Right click on the image -> Save Image as. 
                             
                         
                     
                
             
            

        
             
                 Load Sequence viewer 
             
        
        
         
     
    

    
     
         
            Hit: LT906450.1
         
         
             
                 
                     LT906450.1 Rhodococcus rhodochrous strain NCTC10210 genome assembly, chromosome: 1 
                     
                         
 ?  
This is BLAST alignment as read from the input file 
                         
 Score = 103.0 bits (94.2), Expect = 5.27E-15
 Identities = 97/126 (77%), Gaps = 1/126 (1%)
 Strand = Plus/Minus
Query     15 AGGCCAAGACCCAGCCGGAAGAGAAGGCTAGATCTCCCGACCCAAGCTCCTAGCACGGATACCG 78    
             ||||||||||| |  |||||||||||  | |||||||||||      ||| |||||||| ||||       
Sbjct 511270 AGGCCAAGACCGAATCGGAAGAGAAGATTCGATCTCCCGACTGTTCTTCCCAGCACGGACACCG 511207

Query     79 AGCACCCACGCGGAGCACATGCCGCGGAATAGGCAAAAGTGTTGCGGACCTGCGTAGTTTCG 140   
             || ||||||||| |||  ||||| |   |  |||| |||||||| || |||||| |||| ||       
Sbjct 511206 AGTACCCACGCGAAGCCGATGCCACTATAAGGGCACAAGTGTTGTGGGCCTGCG-AGTTCCG 511146

 
                 
                
                 
                      Report:  
                     
                         
                             sequence start
                                   ?  
                                     
Start position of the estimated full-length sequence in genome.
Start index  
                                 :
                             
                             510983 
                         
                         
                             sequence end
                                   ?  
                                     
End position of the estimated full-length sequence in genome.
Start index  
                                 :
                             
                             511290 
                         
                         
                             bit score (CM)
                                   ?  
                                     
The score for aligning estimated full-length sequence to CM model
  (computed by RSEARCH -> default,
  infered from Rfam or provided by user) 
                                 :
                             
                             163.26 
                         
                         
                             Homology estimate
                                   ?  
                                     
Quick homology estimate:
  Not homologous: bit score   20 and bit score > 0.5 * query length
  Uncertain otherwise 
                                 :
                             
                             Homologous 
                         
                     
                 
                
                
                 
                     
                          Estimated full-length sequence:  
                         
                          ? 
                             
Click checkbox to select multiple seuqences.
Fasta header format:
  UID|accession.versionSTRAND start-end 
                         
                     
                     >uid:26|LT906450.1rc 510983-511290
ACGGAAGGACGGAAGGCCAAGACCGAAUCGGAAGAGAAGAUUCGAUCUCCCGACUGUUCU
UCCCAGCACGGACACCGAGUACCCACGCGAAGCCGAUGCCACUAUAAGGGCACAAGUGUU
GUGGGCCUGCGAGUUCCGGAUUUUCGAAGACGAGUACCCCGCACCCGUUGCGAGGACGAA
UCGACGAUACCCGGAUCAUCGCCGAGGCCGUUGAAUCACAACCCAUUUCAGCACGCAUGG
UAACUCGGACCGCCCGUGCUAGCGGGUGGCGGAAUCGAUCUCGAUCGAUAUGCCACCCGC
UUUCGCGU
 
                 
                
             
            
             
                
                     
                         
                         
                             TurboFold 
                             
                              ? 
                                 
Visualisation of predicted secondary structure.
To save the image:
  Right click on the image -> Save Image as. 
                             
                         
                     
                
                     
                         
                         
                             rfam-Rc 
                             
                              ? 
                                 
Visualisation of predicted secondary structure.
To save the image:
  Right click on the image -> Save Image as. 
                             
                         
                     
                
                     
                         
                         
                             rnafold 
                             
                              ? 
                                 
Visualisation of predicted secondary structure.
To save the image:
  Right click on the image -> Save Image as. 
                             
                         
                     
                
             
            

        
             
                 Load Sequence viewer 
             
        
        
         
     
    

    
     
         
            Hit: LT906450.1
         
         
             
                 
                     LT906450.1 Rhodococcus rhodochrous strain NCTC10210 genome assembly, chromosome: 1 
                     
                         
 ?  
This is BLAST alignment as read from the input file 
                         
 Score = 49.0 bits (45.5), Expect = 2.39E+00
 Identities = 43/53 (81%), Gaps = 3/53 (6%)
 Strand = Plus/Minus
Query    218 CAACCCACATC-GCACGCTTGGTCACTCGGG--GTCCGTGCTAGCGGGCGGCG 267   
             |||||||  || |||||| |||| ||||||   | ||||||||||||| ||||       
Sbjct 511065 CAACCCATTTCAGCACGCATGGTAACTCGGACCGCCCGTGCTAGCGGGTGGCG 511013

 
                 
                
                 
                      Report:  
                     
                         
                             sequence start
                                   ?  
                                     
Start position of the estimated full-length sequence in genome.
Start index  
                                 :
                             
                             510975 
                         
                         
                             sequence end
                                   ?  
                                     
End position of the estimated full-length sequence in genome.
Start index  
                                 :
                             
                             511282 
                         
                         
                             bit score (CM)
                                   ?  
                                     
The score for aligning estimated full-length sequence to CM model
  (computed by RSEARCH -> default,
  infered from Rfam or provided by user) 
                                 :
                             
                             163.26 
                         
                         
                             Homology estimate
                                   ?  
                                     
Quick homology estimate:
  Not homologous: bit score   20 and bit score > 0.5 * query length
  Uncertain otherwise 
                                 :
                             
                             Homologous 
                         
                     
                 
                
                
                 
                     
                          Estimated full-length sequence:  
                         
                          ? 
                             
Click checkbox to select multiple seuqences.
Fasta header format:
  UID|accession.versionSTRAND start-end 
                         
                     
                     >uid:27|LT906450.1rc 510975-511282
ACGGAAGGACGGAAGGCCAAGACCGAAUCGGAAGAGAAGAUUCGAUCUCCCGACUGUUCU
UCCCAGCACGGACACCGAGUACCCACGCGAAGCCGAUGCCACUAUAAGGGCACAAGUGUU
GUGGGCCUGCGAGUUCCGGAUUUUCGAAGACGAGUACCCCGCACCCGUUGCGAGGACGAA
UCGACGAUACCCGGAUCAUCGCCGAGGCCGUUGAAUCACAACCCAUUUCAGCACGCAUGG
UAACUCGGACCGCCCGUGCUAGCGGGUGGCGGAAUCGAUCUCGAUCGAUAUGCCACCCGC
UUUCGCGU
 
                 
                
             
            
             
                
                     
                         
                         
                             TurboFold 
                             
                              ? 
                                 
Visualisation of predicted secondary structure.
To save the image:
  Right click on the image -> Save Image as. 
                             
                         
                     
                
                     
                         
                         
                             rfam-Rc 
                             
                              ? 
                                 
Visualisation of predicted secondary structure.
To save the image:
  Right click on the image -> Save Image as. 
                             
                         
                     
                
                     
                         
                         
                             rnafold 
                             
                              ? 
                                 
Visualisation of predicted secondary structure.
To save the image:
  Right click on the image -> Save Image as. 
                             
                         
                     
                
             
            

        
             
                 Load Sequence viewer 
             
        
        
         
     
    

    
     
         
            Hit: AP011115.1
         
         
             
                 
                     AP011115.1 Rhodococcus opacus B4 DNA, complete genome 
                     
                         
 ?  
This is BLAST alignment as read from the input file 
                         
 Score = 102.0 bits (93.3), Expect = 5.27E-15
 Identities = 197/288 (68%), Gaps = 6/288 (2%)
 Strand = Plus/Minus
Query      15 AGGCCAAGACCCAGCCGGAAGAGAAGGCTAGATCTCCCGACCCAAGCTCCTAGCACGGATAC 76     
              ||||||||  | | || |||||||||| | ||||||||||||     ||| |||||||  ||        
Sbjct 4576105 AGGCCAAGGTCGACCCAGAAGAGAAGGATCGATCTCCCGACCAGGATTCCCAGCACGGGCAC 4576044

Query      77 CGAGCACCCACGCGGAGCACATGCCGCGGAATAGGCAAAAGTGTTGCGGACCTGCGTAGTTT 138    
                 | ||||||||| |||| | ||| |   |  |||||||| |||| || |||||| ||  |        
Sbjct 4576043 TAGGTACCCACGCGAAGCATAAGCCACTATAAGGGCAAAAGCGTTGTGGGCCTGCG-AGACT 4575983

Query     139 CGAAAAGCGGACGGCCACGACGGCCCTTTGGGTGG--GGTTGCAGCCGTAGCGCATCGCAAA 198    
              |||        |||| | |||    |   || ||   ||  | | |||  |    ||| |          
Sbjct 4575982 CGAGTCTTCACCGGCAAGGACCCTGCACCGGTTGCAAGGACG-ACCCGATGATATTCGGACT 4575922

Query     199 GACGCCGAGGTCACCCACGCAACCCAC-ATCGCACGCTTGGTCACTCGGGGTCCGTGCTAGC 259    
                |||||||| ||||||| |||||||| || ||||||||||| ||  |  |||||||||| |        
Sbjct 4575921 CTCGCCGAGGCCACCCACACAACCCACCATTGCACGCTTGGTAACCGGTTGTCCGTGCTAAC 4575860

Query     260 GGGCGGCGAACCGGACA-TTGTCCGGGACGCCGCCCGATT 298    
              ||||||||||   ||||   ||| |   ||||||||| ||        
Sbjct 4575859 GGGCGGCGAAGTCGACACCAGTCGGCAGCGCCGCCCGTTT 4575820

 
                 
                
                 
                      Report:  
                     
                         
                             sequence start
                                   ?  
                                     
Start position of the estimated full-length sequence in genome.
Start index  
                                 :
                             
                             4575815 
                         
                         
                             sequence end
                                   ?  
                                     
End position of the estimated full-length sequence in genome.
Start index  
                                 :
                             
                             4576118 
                         
                         
                             bit score (CM)
                                   ?  
                                     
The score for aligning estimated full-length sequence to CM model
  (computed by RSEARCH -> default,
  infered from Rfam or provided by user) 
                                 :
                             
                             147.32 
                         
                         
                             Homology estimate
                                   ?  
                                     
Quick homology estimate:
  Not homologous: bit score   20 and bit score > 0.5 * query length
  Uncertain otherwise 
                                 :
                             
                             Uncertain 
                         
                     
                 
                
                
                 
                     
                          Estimated full-length sequence:  
                         
                          ? 
                             
Click checkbox to select multiple seuqences.
Fasta header format:
  UID|accession.versionSTRAND start-end 
                         
                     
                     >uid:28|AP011115.1rc 4575815-4576118
ACGGAAGAACGGAAGGCCAAGGUCGACCCAGAAGAGAAGGAUCGAUCUCCCGACCAGGAU
UCCCAGCACGGGCACUAGGUACCCACGCGAAGCAUAAGCCACUAUAAGGGCAAAAGCGUU
GUGGGCCUGCGAGACUCGAGUCUUCACCGGCAAGGACCCUGCACCGGUUGCAAGGACGAC
CCGAUGAUAUUCGGACUCUCGCCGAGGCCACCCACACAACCCACCAUUGCACGCUUGGUA
ACCGGUUGUCCGUGCUAACGGGCGGCGAAGUCGACACCAGUCGGCAGCGCCGCCCGUUUC
ACAU
 
                 
                
             
            
             
                
                     
                         
                         
                             TurboFold 
                             
                              ? 
                                 
Visualisation of predicted secondary structure.
To save the image:
  Right click on the image -> Save Image as. 
                             
                         
                     
                
                     
                         
                         
                             rfam-Rc 
                             
                              ? 
                                 
Visualisation of predicted secondary structure.
To save the image:
  Right click on the image -> Save Image as. 
                             
                         
                     
                
                     
                         
                         
                             rnafold 
                             
                              ? 
                                 
Visualisation of predicted secondary structure.
To save the image:
  Right click on the image -> Save Image as. 
                             
                         
                     
                
             
            

        
             
                 Load Sequence viewer 
             
        
        
         
     
    

    
     
         
            Hit: LS483468.1
         
         
             
                 
                     LS483468.1 Rhodococcus coprophilus strain NCTC10994 genome assembly, chromosome: 1 
                     
                         
 ?  
This is BLAST alignment as read from the input file 
                         
 Score = 100.0 bits (91.5), Expect = 1.84E-14
 Identities = 97/127 (76%), Gaps = 1/127 (1%)
 Strand = Plus/Plus
Query      15 AGGCCAAGACCCAGCCGGAAGAGAAGGCTAGATCTCCCGACCCAAGCTCCTAGCACGGATAC 76     
              ||||||||| |    | |||||||||| | | ||||||| ||  || ||| |||||||| ||        
Sbjct 2467403 AGGCCAAGAGCGGATCAGAAGAGAAGGTTCGGTCTCCCGCCCGGAGTTCCCAGCACGGACAC 2467464

Query      77 CGAGCACCCACGCGGAGCACATGCCGCGGAATAGGCAAAAGTGTTGCGGACCTGCGTAGTTT 138    
              |||| |||||||||||||  ||||| |   |  |||| ||| |||| || |||||| ||||         
Sbjct 2467465 CGAGTACCCACGCGGAGCCGATGCCACTATAAGGGCAGAAGCGTTGTGGGCCTGCG-AGTTG 2467525

Query     139 CGA 141    
              |||        
Sbjct 2467526 CGA 2467528

 
                 
                
                 
                      Report:  
                     
                         
                             sequence start
                                   ?  
                                     
Start position of the estimated full-length sequence in genome.
Start index  
                                 :
                             
                             2467389 
                         
                         
                             sequence end
                                   ?  
                                     
End position of the estimated full-length sequence in genome.
Start index  
                                 :
                             
                             2467696 
                         
                         
                             bit score (CM)
                                   ?  
                                     
The score for aligning estimated full-length sequence to CM model
  (computed by RSEARCH -> default,
  infered from Rfam or provided by user) 
                                 :
                             
                             107.41 
                         
                         
                             Homology estimate
                                   ?  
                                     
Quick homology estimate:
  Not homologous: bit score   20 and bit score > 0.5 * query length
  Uncertain otherwise 
                                 :
                             
                             Uncertain 
                         
                     
                 
                
                
                 
                     
                          Estimated full-length sequence:  
                         
                          ? 
                             
Click checkbox to select multiple seuqences.
Fasta header format:
  UID|accession.versionSTRAND start-end 
                         
                     
                     >uid:29|LS483468.1fw 2467389-2467696
UACGGAACACCGGAAGGCCAAGAGCGGAUCAGAAGAGAAGGUUCGGUCUCCCGCCCGGAG
UUCCCAGCACGGACACCGAGUACCCACGCGGAGCCGAUGCCACUAUAAGGGCAGAAGCGU
UGUGGGCCUGCGAGUUGCGACUCUUCGAGGGUUAGGAUCCCGCACCCGUUGCGGGGCCGG
CCGGAGAUAAUCGUUUCAUCGCCGAGGCCGUUGAGACACAACCCAUUUGAGCACGCAUGG
UAACACGGUAAUUCCGUGUCAGCGGGUGGUACGCCGAUUCGAUAUCGACGCGUCACCCGC
UUUUUCUG
 
                 
                
             
            
             
                
                     
                         
                         
                             TurboFold 
                             
                              ? 
                                 
Visualisation of predicted secondary structure.
To save the image:
  Right click on the image -> Save Image as. 
                             
                         
                     
                
                     
                         
                         
                             rfam-Rc 
                             
                              ? 
                                 
Visualisation of predicted secondary structure.
To save the image:
  Right click on the image -> Save Image as. 
                             
                         
                     
                
                     
                         
                         
                             rnafold 
                             
                              ? 
                                 
Visualisation of predicted secondary structure.
To save the image:
  Right click on the image -> Save Image as. 
                             
                         
                     
                
             
            

        
             
                 Load Sequence viewer 
             
        
        
         
     
    

    
     
         
            Hit: CP019572.1
         
         
             
                 
                     CP019572.1 Rhodococcus sp. MTM3W5.2, complete genome 
                     
                         
 ?  
This is BLAST alignment as read from the input file 
                         
 Score = 100.0 bits (91.5), Expect = 1.84E-14
 Identities = 185/266 (70%), Gaps = 18/266 (7%)
 Strand = Plus/Plus
Query      15 AGGCCAAGACCCAGCCGGAAGAGAAGGCTAGATCTCCCGACC-CAAGCTCCTAGCACGGATA 75     
              ||||||||  | | ||||||||||||| | | ||||||| || | |  ||| |||||||| |        
Sbjct 5216151 AGGCCAAGGTCGACCCGGAAGAGAAGGATCGGTCTCCCGGCCGCGAAGTCCCAGCACGGACA 5216212

Query      76 CCGAGCACCCACGCGGAG-CACATGCCGCGGAATAGGCAAAAGTGTTGCGGACCTGCGTAGT 136    
              ||  |||||||||||||| | || ||| |   |  ||||  || |||| || ||||||| |         
Sbjct 5216213 CCAGGCACCCACGCGGAGCCGCAAGCCACTATAAGGGCAGCAGCGTTGTGGGCCTGCGT-GA 5216273

Query     137 TTCGAAAAGCGGACGGCCACGACGGCCCTTTGGGTGGGGTTGCAG-------CCGTAGCGCA 191    
               ||| |   |   ||||  ||| || ||||  |   | |||||||       | |  |            
Sbjct 5216274 CTCGGA---CATTCGGCAGCGA-GGACCTT--GCACGAGTTGCAGAGGCGAACTGACGATGC 5216329

Query     192 TCGCAAAGACGCCGAGGTCACCCACGCAACCCA-CATCGCACGCTTGGTCACTCGG-GGTCC 251    
              |||     ||||||||| |   ||| ||||||| ||  ||||||||||| || ||| | |||        
Sbjct 5216330 TCGAGCCTACGCCGAGGCCGGACACACAACCCACCAAAGCACGCTTGGTAACCCGGCGATCC 5216391

Query     252 GTGCTAGCGGGCGGCGAA 269    
              ||||||||||||||||||        
Sbjct 5216392 GTGCTAGCGGGCGGCGAA 5216409

 
                 
                
                 
                      Report:  
                     
                         
                             sequence start
                                   ?  
                                     
Start position of the estimated full-length sequence in genome.
Start index  
                                 :
                             
                             5216138 
                         
                         
                             sequence end
                                   ?  
                                     
End position of the estimated full-length sequence in genome.
Start index  
                                 :
                             
                             5216445 
                         
                         
                             bit score (CM)
                                   ?  
                                     
The score for aligning estimated full-length sequence to CM model
  (computed by RSEARCH -> default,
  infered from Rfam or provided by user) 
                                 :
                             
                             133.42 
                         
                         
                             Homology estimate
                                   ?  
                                     
Quick homology estimate:
  Not homologous: bit score   20 and bit score > 0.5 * query length
  Uncertain otherwise 
                                 :
                             
                             Uncertain 
                         
                     
                 
                
                
                 
                     
                          Estimated full-length sequence:  
                         
                          ? 
                             
Click checkbox to select multiple seuqences.
Fasta header format:
  UID|accession.versionSTRAND start-end 
                         
                     
                     >uid:30|CP019572.1fw 5216138-5216445
ACGGAUGAACGGCAGGCCAAGGUCGACCCGGAAGAGAAGGAUCGGUCUCCCGGCCGCGAA
GUCCCAGCACGGACACCAGGCACCCACGCGGAGCCGCAAGCCACUAUAAGGGCAGCAGCG
UUGUGGGCCUGCGUGACUCGGACAUUCGGCAGCGAGGACCUUGCACGAGUUGCAGAGGCG
AACUGACGAUGCUCGAGCCUACGCCGAGGCCGGACACACAACCCACCAAAGCACGCUUGG
UAACCCGGCGAUCCGUGCUAGCGGGCGGCGAAGUCGACAAGAGUCGGCAGCGCCGCUCGC
AUUGCGUC
 
                 
                
             
            
             
                
                     
                         
                         
                             TurboFold 
                             
                              ? 
                                 
Visualisation of predicted secondary structure.
To save the image:
  Right click on the image -> Save Image as. 
                             
                         
                     
                
                     
                         
                         
                             rfam-Rc 
                             
                              ? 
                                 
Visualisation of predicted secondary structure.
To save the image:
  Right click on the image -> Save Image as. 
                             
                         
                     
                
                     
                         
                         
                             rnafold 
                             
                              ? 
                                 
Visualisation of predicted secondary structure.
To save the image:
  Right click on the image -> Save Image as. 
                             
                         
                     
                
             
            

        
             
                 Load Sequence viewer 
             
        
        
         
     
    

    
     
         
            Hit: CP041647.1
         
         
             
                 
                     CP041647.1 Rhodococcus hoagii strain WY chromosome, complete genome 
                     
                         
 ?  
This is BLAST alignment as read from the input file 
                         
 Score = 99.0 bits (90.6), Expect = 6.42E-14
 Identities = 176/256 (69%), Gaps = 7/256 (3%)
 Strand = Plus/Minus
Query      17 GCCAAGACCCAGCCGGAAGAGAAGGCTAGATCTCCCGACCCAAGCTCCTAGCACGGATACCG 78     
              |||| || | | |||||||||||||   |||  |||||||  |  ||| |||||||  |||         
Sbjct 4671000 GCCAGGATCGATCCGGAAGAGAAGGACCGATTCCCCGACCGGACTTCCCAGCACGGGCACCA 4670939

Query      79 AGCACCCACGCGGAGCACATGCCGCGGAATAGGCAAAAGTGTTGCGGACCTGCGTAGTTTCG 140    
               | |||||||||||||||| ||| |   |  |||| ||| |||| || | ||||| |  |||        
Sbjct 4670938 GGTACCCACGCGGAGCACAAGCCACTATAAGGGCAGAAGCGTTGTGGGCTTGCGT-GACTCG 4670878

Query     141 AAAAGCGGACGGCCACGAC---GGCCCTTTGGGTGGGGTTGCAGCCGTAGCGCATCGCAAAG 199    
               |   | |||||| | |||   |  |   | |   |||  | | | | | |||  ||  |          
Sbjct 4670877 GATGACTGACGGCAAGGACCCTGCACACGTTGCACGGGACGAACCGGCACCGC-CCGAGACC 4670817

Query     200 ACGCCGAGGTC--ACCCACGCAACCCACATCGCACGCTTGGTCACTCGGGGTCCGTGCTAGC 259    
              ||||||||| |     ||| |||||||| | ||||||||||| || |||  |||||||||||        
Sbjct 4670816 ACGCCGAGGCCGTTGACACACAACCCACCTTGCACGCTTGGTAACCCGGAATCCGTGCTAGC 4670755

Query     260 GGGCGGCG 267    
              ||| ||||        
Sbjct 4670754 GGGTGGCG 4670747

 
                 
                
                 
                      Report:  
                     
                         
                             sequence start
                                   ?  
                                     
Start position of the estimated full-length sequence in genome.
Start index  
                                 :
                             
                             4670711 
                         
                         
                             sequence end
                                   ?  
                                     
End position of the estimated full-length sequence in genome.
Start index  
                                 :
                             
                             4671017 
                         
                         
                             bit score (CM)
                                   ?  
                                     
The score for aligning estimated full-length sequence to CM model
  (computed by RSEARCH -> default,
  infered from Rfam or provided by user) 
                                 :
                             
                             139.31 
                         
                         
                             Homology estimate
                                   ?  
                                     
Quick homology estimate:
  Not homologous: bit score   20 and bit score > 0.5 * query length
  Uncertain otherwise 
                                 :
                             
                             Uncertain 
                         
                     
                 
                
                
                 
                     
                          Estimated full-length sequence:  
                         
                          ? 
                             
Click checkbox to select multiple seuqences.
Fasta header format:
  UID|accession.versionSTRAND start-end 
                         
                     
                     >uid:31|CP041647.1rc 4670711-4671017
ACGGAAGGCCGGAAAGCCAGGAUCGAUCCGGAAGAGAAGGACCGAUUCCCCGACCGGACU
UCCCAGCACGGGCACCAGGUACCCACGCGGAGCACAAGCCACUAUAAGGGCAGAAGCGUU
GUGGGCUUGCGUGACUCGGAUGACUGACGGCAAGGACCCUGCACACGUUGCACGGGACGA
ACCGGCACCGCCCGAGACCACGCCGAGGCCGUUGACACACAACCCACCUUGCACGCUUGG
UAACCCGGAAUCCGUGCUAGCGGGUGGCGGCGCCGAUCACGGUCGGUGACACCACCCGCU
UCGCAUG
 
                 
                
             
            
             
                
                     
                         
                         
                             TurboFold 
                             
                              ? 
                                 
Visualisation of predicted secondary structure.
To save the image:
  Right click on the image -> Save Image as. 
                             
                         
                     
                
                     
                         
                         
                             rfam-Rc 
                             
                              ? 
                                 
Visualisation of predicted secondary structure.
To save the image:
  Right click on the image -> Save Image as. 
                             
                         
                     
                
                     
                         
                         
                             rnafold 
                             
                              ? 
                                 
Visualisation of predicted secondary structure.
To save the image:
  Right click on the image -> Save Image as. 
                             
                         
                     
                
             
            

        
             
                 Load Sequence viewer 
             
        
        
         
     
    

    
     
         
            Hit: FN563149.1
         
         
             
                 
                     FN563149.1 Rhodococcus equi 103S chromosome 
                     
                         
 ?  
This is BLAST alignment as read from the input file 
                         
 Score = 99.0 bits (90.6), Expect = 6.42E-14
 Identities = 176/256 (69%), Gaps = 7/256 (3%)
 Strand = Plus/Minus
Query     17 GCCAAGACCCAGCCGGAAGAGAAGGCTAGATCTCCCGACCCAAGCTCCTAGCACGGATACCGAG 80    
             |||| || | | |||||||||||||   |||  |||||||  |  ||| |||||||  |||  |       
Sbjct 560162 GCCAGGATCGATCCGGAAGAGAAGGACCGATTCCCCGACCGGACTTCCCAGCACGGGCACCAGG 560099

Query     81 CACCCACGCGGAGCACATGCCGCGGAATAGGCAAAAGTGTTGCGGACCTGCGTAGTTTCGAAAA 144   
              |||||||||||||||| ||| |   |  |||| ||| |||| || | ||||| |  ||| |         
Sbjct 560098 TACCCACGCGGAGCACAAGCCACTATAAGGGCAGAAGCGTTGTGGGCTTGCGT-GACTCGGATG 560036

Query    145 GCGGACGGCCACGAC---GGCCCTTTGGGTGGGGTTGCAGCCGTAGCGCATCGCAAAGACGCCG 205   
              | |||||| | |||   |  |   | |   |||  | | | | | |||  ||  |  ||||||       
Sbjct 560035 ACTGACGGCAAGGACCCTGCACACGTTGCACGGGACGAACCGGCACCGC-CCGAGACCACGCCG 559973

Query    206 AGGTC--ACCCACGCAACCCACATCGCACGCTTGGTCACTCGGGGTCCGTGCTAGCGGGCGGCG 267   
             ||| |     ||| |||||||| | ||||||||||| || |||  |||||||||||||| ||||       
Sbjct 559972 AGGCCGTTGACACACAACCCACCTTGCACGCTTGGTAACCCGGAATCCGTGCTAGCGGGTGGCG 559909

 
                 
                
                 
                      Report:  
                     
                         
                             sequence start
                                   ?  
                                     
Start position of the estimated full-length sequence in genome.
Start index  
                                 :
                             
                             559873 
                         
                         
                             sequence end
                                   ?  
                                     
End position of the estimated full-length sequence in genome.
Start index  
                                 :
                             
                             560179 
                         
                         
                             bit score (CM)
                                   ?  
                                     
The score for aligning estimated full-length sequence to CM model
  (computed by RSEARCH -> default,
  infered from Rfam or provided by user) 
                                 :
                             
                             141.76 
                         
                         
                             Homology estimate
                                   ?  
                                     
Quick homology estimate:
  Not homologous: bit score   20 and bit score > 0.5 * query length
  Uncertain otherwise 
                                 :
                             
                             Uncertain 
                         
                     
                 
                
                
                 
                     
                          Estimated full-length sequence:  
                         
                          ? 
                             
Click checkbox to select multiple seuqences.
Fasta header format:
  UID|accession.versionSTRAND start-end 
                         
                     
                     >uid:32|FN563149.1rc 559873-560179
ACGGAAGGCCGGAAAGCCAGGAUCGAUCCGGAAGAGAAGGACCGAUUCCCCGACCGGACU
UCCCAGCACGGGCACCAGGUACCCACGCGGAGCACAAGCCACUAUAAGGGCAGAAGCGUU
GUGGGCUUGCGUGACUCGGAUGACUGACGGCAAGGACCCUGCACACGUUGCACGGGACGA
ACCGGCACCGCCCGAGACCACGCCGAGGCCGUUGACACACAACCCACCUUGCACGCUUGG
UAACCCGGAAUCCGUGCUAGCGGGUGGCGGCGCCGACCACGGUCGGUGACACCACCCGCU
UCGCAUG
 
                 
                
             
            
             
                
                     
                         
                         
                             TurboFold 
                             
                              ? 
                                 
Visualisation of predicted secondary structure.
To save the image:
  Right click on the image -> Save Image as. 
                             
                         
                     
                
                     
                         
                         
                             rfam-Rc 
                             
                              ? 
                                 
Visualisation of predicted secondary structure.
To save the image:
  Right click on the image -> Save Image as. 
                             
                         
                     
                
                     
                         
                         
                             rnafold 
                             
                              ? 
                                 
Visualisation of predicted secondary structure.
To save the image:
  Right click on the image -> Save Image as. 
                             
                         
                     
                
             
            

        
             
                 Load Sequence viewer 
             
        
        
         
     
    

    
     
         
            Hit: CP040719.1
         
         
             
                 
                     CP040719.1 Rhodococcus pyridinivorans strain YF3 chromosome, complete genome 
                     
                         
 ?  
This is BLAST alignment as read from the input file 
                         
 Score = 98.0 bits (89.7), Expect = 6.42E-14
 Identities = 96/126 (76%), Gaps = 1/126 (1%)
 Strand = Plus/Minus
Query     15 AGGCCAAGACCCAGCCGGAAGAGAAGGCTAGATCTCCCGACCCAAGCTCCTAGCACGGATACCG 78    
             ||||||||||| |  |||||||||||  | |||||||||||      ||| |||||||| ||||       
Sbjct 320498 AGGCCAAGACCGAATCGGAAGAGAAGATTCGATCTCCCGACTGTTCTTCCCAGCACGGACACCG 320435

Query     79 AGCACCCACGCGGAGCACATGCCGCGGAATAGGCAAAAGTGTTGCGGACCTGCGTAGTTTCG 140   
             || ||||||||| |||  ||||| |   |  ||||  ||||||| || |||||| |||| ||       
Sbjct 320434 AGTACCCACGCGAAGCCGATGCCACTATAAGGGCACGAGTGTTGTGGGCCTGCG-AGTTCCG 320374

 
                 
                
                 
                      Report:  
                     
                         
                             sequence start
                                   ?  
                                     
Start position of the estimated full-length sequence in genome.
Start index  
                                 :
                             
                             320211 
                         
                         
                             sequence end
                                   ?  
                                     
End position of the estimated full-length sequence in genome.
Start index  
                                 :
                             
                             320518 
                         
                         
                             bit score (CM)
                                   ?  
                                     
The score for aligning estimated full-length sequence to CM model
  (computed by RSEARCH -> default,
  infered from Rfam or provided by user) 
                                 :
                             
                             161.38 
                         
                         
                             Homology estimate
                                   ?  
                                     
Quick homology estimate:
  Not homologous: bit score   20 and bit score > 0.5 * query length
  Uncertain otherwise 
                                 :
                             
                             Homologous 
                         
                     
                 
                
                
                 
                     
                          Estimated full-length sequence:  
                         
                          ? 
                             
Click checkbox to select multiple seuqences.
Fasta header format:
  UID|accession.versionSTRAND start-end 
                         
                     
                     >uid:33|CP040719.1rc 320211-320518
ACGGAAGGACGGAAGGCCAAGACCGAAUCGGAAGAGAAGAUUCGAUCUCCCGACUGUUCU
UCCCAGCACGGACACCGAGUACCCACGCGAAGCCGAUGCCACUAUAAGGGCACGAGUGUU
GUGGGCCUGCGAGUUCCGGAUUUUCGAAGACGAGUACCCCGCACCCGUUGCGAGGACGAA
UCGACGAUACCCGGAUCAUCGCCGAGGCCGUUGAAUCACAACCCAUUUCAGCACGCAUGG
UAACUCGGACCGCCCGUGCUAGCGGGUGGCGGAAUCGAUCUCGAUCGAUAUGCCACCCGC
UUUCGCGU
 
                 
                
             
            
             
                
                     
                         
                         
                             TurboFold 
                             
                              ? 
                                 
Visualisation of predicted secondary structure.
To save the image:
  Right click on the image -> Save Image as. 
                             
                         
                     
                
                     
                         
                         
                             rfam-Rc 
                             
                              ? 
                                 
Visualisation of predicted secondary structure.
To save the image:
  Right click on the image -> Save Image as. 
                             
                         
                     
                
                     
                         
                         
                             rnafold 
                             
                              ? 
                                 
Visualisation of predicted secondary structure.
To save the image:
  Right click on the image -> Save Image as. 
                             
                         
                     
                
             
            

        
             
                 Load Sequence viewer 
             
        
        
         
     
    

    
     
         
            Hit: CP040719.1
         
         
             
                 
                     CP040719.1 Rhodococcus pyridinivorans strain YF3 chromosome, complete genome 
                     
                         
 ?  
This is BLAST alignment as read from the input file 
                         
 Score = 49.0 bits (45.5), Expect = 2.39E+00
 Identities = 43/53 (81%), Gaps = 3/53 (6%)
 Strand = Plus/Minus
Query    218 CAACCCACATC-GCACGCTTGGTCACTCGGG--GTCCGTGCTAGCGGGCGGCG 267   
             |||||||  || |||||| |||| ||||||   | ||||||||||||| ||||       
Sbjct 320293 CAACCCATTTCAGCACGCATGGTAACTCGGACCGCCCGTGCTAGCGGGTGGCG 320241

 
                 
                
                 
                      Report:  
                     
                         
                             sequence start
                                   ?  
                                     
Start position of the estimated full-length sequence in genome.
Start index  
                                 :
                             
                             320203 
                         
                         
                             sequence end
                                   ?  
                                     
End position of the estimated full-length sequence in genome.
Start index  
                                 :
                             
                             320510 
                         
                         
                             bit score (CM)
                                   ?  
                                     
The score for aligning estimated full-length sequence to CM model
  (computed by RSEARCH -> default,
  infered from Rfam or provided by user) 
                                 :
                             
                             161.38 
                         
                         
                             Homology estimate
                                   ?  
                                     
Quick homology estimate:
  Not homologous: bit score   20 and bit score > 0.5 * query length
  Uncertain otherwise 
                                 :
                             
                             Homologous 
                         
                     
                 
                
                
                 
                     
                          Estimated full-length sequence:  
                         
                          ? 
                             
Click checkbox to select multiple seuqences.
Fasta header format:
  UID|accession.versionSTRAND start-end 
                         
                     
                     >uid:34|CP040719.1rc 320203-320510
ACGGAAGGACGGAAGGCCAAGACCGAAUCGGAAGAGAAGAUUCGAUCUCCCGACUGUUCU
UCCCAGCACGGACACCGAGUACCCACGCGAAGCCGAUGCCACUAUAAGGGCACGAGUGUU
GUGGGCCUGCGAGUUCCGGAUUUUCGAAGACGAGUACCCCGCACCCGUUGCGAGGACGAA
UCGACGAUACCCGGAUCAUCGCCGAGGCCGUUGAAUCACAACCCAUUUCAGCACGCAUGG
UAACUCGGACCGCCCGUGCUAGCGGGUGGCGGAAUCGAUCUCGAUCGAUAUGCCACCCGC
UUUCGCGU
 
                 
                
             
            
             
                
                     
                         
                         
                             TurboFold 
                             
                              ? 
                                 
Visualisation of predicted secondary structure.
To save the image:
  Right click on the image -> Save Image as. 
                             
                         
                     
                
                     
                         
                         
                             rfam-Rc 
                             
                              ? 
                                 
Visualisation of predicted secondary structure.
To save the image:
  Right click on the image -> Save Image as. 
                             
                         
                     
                
                     
                         
                         
                             rnafold 
                             
                              ? 
                                 
Visualisation of predicted secondary structure.
To save the image:
  Right click on the image -> Save Image as. 
                             
                         
                     
                
             
            

        
             
                 Load Sequence viewer 
             
        
        
         
     
    

    
     
         
            Hit: CP032762.1
         
         
             
                 
                     CP032762.1 Rhodococcus sp. P1Y chromosome, complete genome 
                     
                         
 ?  
This is BLAST alignment as read from the input file 
                         
 Score = 98.0 bits (89.7), Expect = 6.42E-14
 Identities = 98/127 (77%), Gaps = 4/127 (3%)
 Strand = Plus/Plus
Query     15 AGGCCAAGACCCAGCCGGAAGAGAAGGCTAGAT-CTCCCGACCCAAGCTCCTAGCACGGATACC 77    
             ||||||||  | | ||||||||||||| | | | |||||||||     ||| |||||||||| |       
Sbjct 131477 AGGCCAAGTTCGAACCGGAAGAGAAGGTTCGTTTCTCCCGACCAGGATTCCCAGCACGGATATC 131540

Query     78 GAGCACCCACGCGGAGCACATGCCGCGGAATAGGCAAAAGTGTTGCGGACCTGCGTAGTTTCG 140   
               ||||||||||||||| |  ||||| |||  |||| ||| | || || |||||| |||||||       
Sbjct 131541 AGGCACCCACGCGGAGCGC--GCCGCTGAAATGGCAGAAGCGCTGTGGGCCTGCG-AGTTTCG 131600

 
                 
                
                 
                      Report:  
                     
                         
                             sequence start
                                   ?  
                                     
Start position of the estimated full-length sequence in genome.
Start index  
                                 :
                             
                             131464 
                         
                         
                             sequence end
                                   ?  
                                     
End position of the estimated full-length sequence in genome.
Start index  
                                 :
                             
                             131780 
                         
                         
                             bit score (CM)
                                   ?  
                                     
The score for aligning estimated full-length sequence to CM model
  (computed by RSEARCH -> default,
  infered from Rfam or provided by user) 
                                 :
                             
                             109.86 
                         
                         
                             Homology estimate
                                   ?  
                                     
Quick homology estimate:
  Not homologous: bit score   20 and bit score > 0.5 * query length
  Uncertain otherwise 
                                 :
                             
                             Uncertain 
                         
                     
                 
                
                
                 
                     
                          Estimated full-length sequence:  
                         
                          ? 
                             
Click checkbox to select multiple seuqences.
Fasta header format:
  UID|accession.versionSTRAND start-end 
                         
                     
                     >uid:35|CP032762.1fw 131464-131780
ACGGAAGAACGGAAGGCCAAGUUCGAACCGGAAGAGAAGGUUCGUUUCUCCCGACCAGGA
UUCCCAGCACGGAUAUCAGGCACCCACGCGGAGCGCGCCGCUGAAAUGGCAGAAGCGCUG
UGGGCCUGCGAGUUUCGGGAAGUAGCAGCAAGAACCCUGCACCGGUUGCAGGGAUGACCU
CCUCCUUCACCGGAUCUACGCCGAGGCCUCGCGAUCAUUGAUCGCCGCAGCCCACCUUUG
CACGCUUGGUAACCAGAUAAUCCGUGCUAACGGGCGGCGAAGUCGACUCAGGUCGGCGGC
GCCGCCCUUUUACGUUC
 
                 
                
             
            
             
                
                     
                         
                         
                             TurboFold 
                             
                              ? 
                                 
Visualisation of predicted secondary structure.
To save the image:
  Right click on the image -> Save Image as. 
                             
                         
                     
                
                     
                         
                         
                             rfam-Rc 
                             
                              ? 
                                 
Visualisation of predicted secondary structure.
To save the image:
  Right click on the image -> Save Image as. 
                             
                         
                     
                
                     
                         
                         
                             rnafold 
                             
                              ? 
                                 
Visualisation of predicted secondary structure.
To save the image:
  Right click on the image -> Save Image as. 
                             
                         
                     
                
             
            

        
             
                 Load Sequence viewer 
             
        
        
         
     
    

    
     
         
            Hit: CP022208.1
         
         
             
                 
                     CP022208.1 Rhodococcus biphenylivorans strain TG9 chromosome, complete genome 
                     
                         
 ?  
This is BLAST alignment as read from the input file 
                         
 Score = 98.0 bits (89.7), Expect = 6.42E-14
 Identities = 96/126 (76%), Gaps = 1/126 (1%)
 Strand = Plus/Minus
Query      15 AGGCCAAGACCCAGCCGGAAGAGAAGGCTAGATCTCCCGACCCAAGCTCCTAGCACGGATAC 76     
              ||||||||||| |  |||||||||||  | |||||||||||      ||| |||||||| ||        
Sbjct 1030603 AGGCCAAGACCGAATCGGAAGAGAAGATTCGATCTCCCGACTGTTCTTCCCAGCACGGACAC 1030542

Query      77 CGAGCACCCACGCGGAGCACATGCCGCGGAATAGGCAAAAGTGTTGCGGACCTGCGTAGTTT 138    
              |||| ||||||||| |||  ||||| |   |  ||||  ||||||| || |||||| ||||         
Sbjct 1030541 CGAGTACCCACGCGAAGCCGATGCCACTATAAGGGCACGAGTGTTGTGGGCCTGCG-AGTTC 1030481

Query     139 CG 140    
              ||        
Sbjct 1030480 CG 1030479

 
                 
                
                 
                      Report:  
                     
                         
                             sequence start
                                   ?  
                                     
Start position of the estimated full-length sequence in genome.
Start index  
                                 :
                             
                             1030316 
                         
                         
                             sequence end
                                   ?  
                                     
End position of the estimated full-length sequence in genome.
Start index  
                                 :
                             
                             1030623 
                         
                         
                             bit score (CM)
                                   ?  
                                     
The score for aligning estimated full-length sequence to CM model
  (computed by RSEARCH -> default,
  infered from Rfam or provided by user) 
                                 :
                             
                             161.38 
                         
                         
                             Homology estimate
                                   ?  
                                     
Quick homology estimate:
  Not homologous: bit score   20 and bit score > 0.5 * query length
  Uncertain otherwise 
                                 :
                             
                             Homologous 
                         
                     
                 
                
                
                 
                     
                          Estimated full-length sequence:  
                         
                          ? 
                             
Click checkbox to select multiple seuqences.
Fasta header format:
  UID|accession.versionSTRAND start-end 
                         
                     
                     >uid:36|CP022208.1rc 1030316-1030623
ACGGAAGGACGGAAGGCCAAGACCGAAUCGGAAGAGAAGAUUCGAUCUCCCGACUGUUCU
UCCCAGCACGGACACCGAGUACCCACGCGAAGCCGAUGCCACUAUAAGGGCACGAGUGUU
GUGGGCCUGCGAGUUCCGGAUUUUCGAAGACGAGUACCCCGCACCCGUUGCGAGGACGAA
UCGACGAUACCCGGAUCAUCGCCGAGGCCGUUGAAUCACAACCCAUUUCAGCACGCAUGG
UAACUCGGACCGCCCGUGCUAGCGGGUGGCGGAAUCGAUCUCGAUCGAUAUGCCACCCGC
UUUCGCGU
 
                 
                
             
            
             
                
                     
                         
                         
                             TurboFold 
                             
                              ? 
                                 
Visualisation of predicted secondary structure.
To save the image:
  Right click on the image -> Save Image as. 
                             
                         
                     
                
                     
                         
                         
                             rfam-Rc 
                             
                              ? 
                                 
Visualisation of predicted secondary structure.
To save the image:
  Right click on the image -> Save Image as. 
                             
                         
                     
                
                     
                         
                         
                             rnafold 
                             
                              ? 
                                 
Visualisation of predicted secondary structure.
To save the image:
  Right click on the image -> Save Image as. 
                             
                         
                     
                
             
            

        
             
                 Load Sequence viewer 
             
        
        
         
     
    

    
     
         
            Hit: CP022208.1
         
         
             
                 
                     CP022208.1 Rhodococcus biphenylivorans strain TG9 chromosome, complete genome 
                     
                         
 ?  
This is BLAST alignment as read from the input file 
                         
 Score = 49.0 bits (45.5), Expect = 2.39E+00
 Identities = 43/53 (81%), Gaps = 3/53 (6%)
 Strand = Plus/Minus
Query     218 CAACCCACATC-GCACGCTTGGTCACTCGGG--GTCCGTGCTAGCGGGCGGCG 267    
              |||||||  || |||||| |||| ||||||   | ||||||||||||| ||||        
Sbjct 1030398 CAACCCATTTCAGCACGCATGGTAACTCGGACCGCCCGTGCTAGCGGGTGGCG 1030346

 
                 
                
                 
                      Report:  
                     
                         
                             sequence start
                                   ?  
                                     
Start position of the estimated full-length sequence in genome.
Start index  
                                 :
                             
                             1030308 
                         
                         
                             sequence end
                                   ?  
                                     
End position of the estimated full-length sequence in genome.
Start index  
                                 :
                             
                             1030615 
                         
                         
                             bit score (CM)
                                   ?  
                                     
The score for aligning estimated full-length sequence to CM model
  (computed by RSEARCH -> default,
  infered from Rfam or provided by user) 
                                 :
                             
                             161.38 
                         
                         
                             Homology estimate
                                   ?  
                                     
Quick homology estimate:
  Not homologous: bit score   20 and bit score > 0.5 * query length
  Uncertain otherwise 
                                 :
                             
                             Homologous 
                         
                     
                 
                
                
                 
                     
                          Estimated full-length sequence:  
                         
                          ? 
                             
Click checkbox to select multiple seuqences.
Fasta header format:
  UID|accession.versionSTRAND start-end 
                         
                     
                     >uid:37|CP022208.1rc 1030308-1030615
ACGGAAGGACGGAAGGCCAAGACCGAAUCGGAAGAGAAGAUUCGAUCUCCCGACUGUUCU
UCCCAGCACGGACACCGAGUACCCACGCGAAGCCGAUGCCACUAUAAGGGCACGAGUGUU
GUGGGCCUGCGAGUUCCGGAUUUUCGAAGACGAGUACCCCGCACCCGUUGCGAGGACGAA
UCGACGAUACCCGGAUCAUCGCCGAGGCCGUUGAAUCACAACCCAUUUCAGCACGCAUGG
UAACUCGGACCGCCCGUGCUAGCGGGUGGCGGAAUCGAUCUCGAUCGAUAUGCCACCCGC
UUUCGCGU
 
                 
                
             
            
             
                
                     
                         
                         
                             TurboFold 
                             
                              ? 
                                 
Visualisation of predicted secondary structure.
To save the image:
  Right click on the image -> Save Image as. 
                             
                         
                     
                
                     
                         
                         
                             rfam-Rc 
                             
                              ? 
                                 
Visualisation of predicted secondary structure.
To save the image:
  Right click on the image -> Save Image as. 
                             
                         
                     
                
                     
                         
                         
                             rnafold 
                             
                              ? 
                                 
Visualisation of predicted secondary structure.
To save the image:
  Right click on the image -> Save Image as. 
                             
                         
                     
                
             
            

        
             
                 Load Sequence viewer 
             
        
        
         
     
    

    
     
         
            Hit: CP022915.1
         
         
             
                 
                     CP022915.1 Rhodococcus pyridinivorans strain GF3, complete genome 
                     
                         
 ?  
This is BLAST alignment as read from the input file 
                         
 Score = 98.0 bits (89.7), Expect = 6.42E-14
 Identities = 96/126 (76%), Gaps = 1/126 (1%)
 Strand = Plus/Plus
Query     15 AGGCCAAGACCCAGCCGGAAGAGAAGGCTAGATCTCCCGACCCAAGCTCCTAGCACGGATACCG 78    
             ||||||||||| |  |||||||||||  | |||||||||||      ||| |||||||| ||||       
Sbjct 406014 AGGCCAAGACCGAATCGGAAGAGAAGATTCGATCTCCCGACTGTTCTTCCCAGCACGGACACCG 406077

Query     79 AGCACCCACGCGGAGCACATGCCGCGGAATAGGCAAAAGTGTTGCGGACCTGCGTAGTTTCG 140   
             || ||||||||| |||  ||||| |   |  ||||  ||||||| || |||||| |||| ||       
Sbjct 406078 AGTACCCACGCGAAGCCGATGCCACTATAAGGGCACGAGTGTTGTGGGCCTGCG-AGTTCCG 406138

 
                 
                
                 
                      Report:  
                     
                         
                             sequence start
                                   ?  
                                     
Start position of the estimated full-length sequence in genome.
Start index  
                                 :
                             
                             406001 
                         
                         
                             sequence end
                                   ?  
                                     
End position of the estimated full-length sequence in genome.
Start index  
                                 :
                             
                             406308 
                         
                         
                             bit score (CM)
                                   ?  
                                     
The score for aligning estimated full-length sequence to CM model
  (computed by RSEARCH -> default,
  infered from Rfam or provided by user) 
                                 :
                             
                             161.38 
                         
                         
                             Homology estimate
                                   ?  
                                     
Quick homology estimate:
  Not homologous: bit score   20 and bit score > 0.5 * query length
  Uncertain otherwise 
                                 :
                             
                             Homologous 
                         
                     
                 
                
                
                 
                     
                          Estimated full-length sequence:  
                         
                          ? 
                             
Click checkbox to select multiple seuqences.
Fasta header format:
  UID|accession.versionSTRAND start-end 
                         
                     
                     >uid:38|CP022915.1fw 406001-406308
ACGGAAGGACGGAAGGCCAAGACCGAAUCGGAAGAGAAGAUUCGAUCUCCCGACUGUUCU
UCCCAGCACGGACACCGAGUACCCACGCGAAGCCGAUGCCACUAUAAGGGCACGAGUGUU
GUGGGCCUGCGAGUUCCGGAUUUUCGAAGACGAGUACCCCGCACCCGUUGCGAGGACGAA
UCGACGAUACCCGGAUCAUCGCCGAGGCCGUUGAAUCACAACCCAUUUCAGCACGCAUGG
UAACUCGGACCGCCCGUGCUAGCGGGUGGCGGAAUCGAUCUCGAUCGAUAUGCCACCCGC
UUUCGCGU
 
                 
                
             
            
             
                
                     
                         
                         
                             TurboFold 
                             
                              ? 
                                 
Visualisation of predicted secondary structure.
To save the image:
  Right click on the image -> Save Image as. 
                             
                         
                     
                
                     
                         
                         
                             rfam-Rc 
                             
                              ? 
                                 
Visualisation of predicted secondary structure.
To save the image:
  Right click on the image -> Save Image as. 
                             
                         
                     
                
                     
                         
                         
                             rnafold 
                             
                              ? 
                                 
Visualisation of predicted secondary structure.
To save the image:
  Right click on the image -> Save Image as. 
                             
                         
                     
                
             
            

        
             
                 Load Sequence viewer 
             
        
        
         
     
    

    
     
         
            Hit: CP022915.1
         
         
             
                 
                     CP022915.1 Rhodococcus pyridinivorans strain GF3, complete genome 
                     
                         
 ?  
This is BLAST alignment as read from the input file 
                         
 Score = 49.0 bits (45.5), Expect = 2.39E+00
 Identities = 43/53 (81%), Gaps = 3/53 (6%)
 Strand = Plus/Plus
Query    218 CAACCCACATC-GCACGCTTGGTCACTCGGG--GTCCGTGCTAGCGGGCGGCG 267   
             |||||||  || |||||| |||| ||||||   | ||||||||||||| ||||       
Sbjct 406219 CAACCCATTTCAGCACGCATGGTAACTCGGACCGCCCGTGCTAGCGGGTGGCG 406271

 
                 
                
                 
                      Report:  
                     
                         
                             sequence start
                                   ?  
                                     
Start position of the estimated full-length sequence in genome.
Start index  
                                 :
                             
                             406001 
                         
                         
                             sequence end
                                   ?  
                                     
End position of the estimated full-length sequence in genome.
Start index  
                                 :
                             
                             406308 
                         
                         
                             bit score (CM)
                                   ?  
                                     
The score for aligning estimated full-length sequence to CM model
  (computed by RSEARCH -> default,
  infered from Rfam or provided by user) 
                                 :
                             
                             161.38 
                         
                         
                             Homology estimate
                                   ?  
                                     
Quick homology estimate:
  Not homologous: bit score   20 and bit score > 0.5 * query length
  Uncertain otherwise 
                                 :
                             
                             Homologous 
                         
                     
                 
                
                
                 
                     
                          Estimated full-length sequence:  
                         
                          ? 
                             
Click checkbox to select multiple seuqences.
Fasta header format:
  UID|accession.versionSTRAND start-end 
                         
                     
                     >uid:39|CP022915.1fw 406001-406308
ACGGAAGGACGGAAGGCCAAGACCGAAUCGGAAGAGAAGAUUCGAUCUCCCGACUGUUCU
UCCCAGCACGGACACCGAGUACCCACGCGAAGCCGAUGCCACUAUAAGGGCACGAGUGUU
GUGGGCCUGCGAGUUCCGGAUUUUCGAAGACGAGUACCCCGCACCCGUUGCGAGGACGAA
UCGACGAUACCCGGAUCAUCGCCGAGGCCGUUGAAUCACAACCCAUUUCAGCACGCAUGG
UAACUCGGACCGCCCGUGCUAGCGGGUGGCGGAAUCGAUCUCGAUCGAUAUGCCACCCGC
UUUCGCGU
 
                 
                
             
            
             
                
                     
                         
                         
                             TurboFold 
                             
                              ? 
                                 
Visualisation of predicted secondary structure.
To save the image:
  Right click on the image -> Save Image as. 
                             
                         
                     
                
                     
                         
                         
                             rfam-Rc 
                             
                              ? 
                                 
Visualisation of predicted secondary structure.
To save the image:
  Right click on the image -> Save Image as. 
                             
                         
                     
                
                     
                         
                         
                             rnafold 
                             
                              ? 
                                 
Visualisation of predicted secondary structure.
To save the image:
  Right click on the image -> Save Image as. 
                             
                         
                     
                
             
            

        
             
                 Load Sequence viewer 
             
        
        
         
     
    

    
     
         
            Hit: CP018063.1
         
         
             
                 
                     CP018063.1 Rhodococcus sp. 2G, complete genome 
                     
                         
 ?  
This is BLAST alignment as read from the input file 
                         
 Score = 98.0 bits (89.7), Expect = 6.42E-14
 Identities = 96/126 (76%), Gaps = 1/126 (1%)
 Strand = Plus/Minus
Query      15 AGGCCAAGACCCAGCCGGAAGAGAAGGCTAGATCTCCCGACCCAAGCTCCTAGCACGGATAC 76     
              ||||||||||| |  |||||||||||  | |||||||||||      ||| |||||||| ||        
Sbjct 4914091 AGGCCAAGACCGAATCGGAAGAGAAGATTCGATCTCCCGACTGTTCTTCCCAGCACGGACAC 4914030

Query      77 CGAGCACCCACGCGGAGCACATGCCGCGGAATAGGCAAAAGTGTTGCGGACCTGCGTAGTTT 138    
              |||| ||||||||| |||  ||||| |   |  ||||  ||||||| || |||||| ||||         
Sbjct 4914029 CGAGTACCCACGCGAAGCCGATGCCACTATAAGGGCACGAGTGTTGTGGGCCTGCG-AGTTC 4913969

Query     139 CG 140    
              ||        
Sbjct 4913968 CG 4913967

 
                 
                
                 
                      Report:  
                     
                         
                             sequence start
                                   ?  
                                     
Start position of the estimated full-length sequence in genome.
Start index  
                                 :
                             
                             4913804 
                         
                         
                             sequence end
                                   ?  
                                     
End position of the estimated full-length sequence in genome.
Start index  
                                 :
                             
                             4914111 
                         
                         
                             bit score (CM)
                                   ?  
                                     
The score for aligning estimated full-length sequence to CM model
  (computed by RSEARCH -> default,
  infered from Rfam or provided by user) 
                                 :
                             
                             161.38 
                         
                         
                             Homology estimate
                                   ?  
                                     
Quick homology estimate:
  Not homologous: bit score   20 and bit score > 0.5 * query length
  Uncertain otherwise 
                                 :
                             
                             Homologous 
                         
                     
                 
                
                
                 
                     
                          Estimated full-length sequence:  
                         
                          ? 
                             
Click checkbox to select multiple seuqences.
Fasta header format:
  UID|accession.versionSTRAND start-end 
                         
                     
                     >uid:40|CP018063.1rc 4913804-4914111
ACGGAAGGACGGAAGGCCAAGACCGAAUCGGAAGAGAAGAUUCGAUCUCCCGACUGUUCU
UCCCAGCACGGACACCGAGUACCCACGCGAAGCCGAUGCCACUAUAAGGGCACGAGUGUU
GUGGGCCUGCGAGUUCCGGAUUUUCGAAGACGAGUACCCCGCACCCGUUGCGAGGACGAA
UCGACGAUACCCGGAUCAUCGCCGAGGCCGUUGAAUCACAACCCAUUUCAGCACGCAUGG
UAACUCGGACCGCCCGUGCUAGCGGGUGGCGGAAUCGAUCUCGAUCGAUAUGCCACCCGC
UUUCGCGU
 
                 
                
             
            
             
                
                     
                         
                         
                             TurboFold 
                             
                              ? 
                                 
Visualisation of predicted secondary structure.
To save the image:
  Right click on the image -> Save Image as. 
                             
                         
                     
                
                     
                         
                         
                             rfam-Rc 
                             
                              ? 
                                 
Visualisation of predicted secondary structure.
To save the image:
  Right click on the image -> Save Image as. 
                             
                         
                     
                
                     
                         
                         
                             rnafold 
                             
                              ? 
                                 
Visualisation of predicted secondary structure.
To save the image:
  Right click on the image -> Save Image as. 
                             
                         
                     
                
             
            

        
             
                 Load Sequence viewer 
             
        
        
         
     
    

    
     
         
            Hit: CP018063.1
         
         
             
                 
                     CP018063.1 Rhodococcus sp. 2G, complete genome 
                     
                         
 ?  
This is BLAST alignment as read from the input file 
                         
 Score = 49.0 bits (45.5), Expect = 2.39E+00
 Identities = 43/53 (81%), Gaps = 3/53 (6%)
 Strand = Plus/Minus
Query     218 CAACCCACATC-GCACGCTTGGTCACTCGGG--GTCCGTGCTAGCGGGCGGCG 267    
              |||||||  || |||||| |||| ||||||   | ||||||||||||| ||||        
Sbjct 4913886 CAACCCATTTCAGCACGCATGGTAACTCGGACCGCCCGTGCTAGCGGGTGGCG 4913834

 
                 
                
                 
                      Report:  
                     
                         
                             sequence start
                                   ?  
                                     
Start position of the estimated full-length sequence in genome.
Start index  
                                 :
                             
                             4913796 
                         
                         
                             sequence end
                                   ?  
                                     
End position of the estimated full-length sequence in genome.
Start index  
                                 :
                             
                             4914103 
                         
                         
                             bit score (CM)
                                   ?  
                                     
The score for aligning estimated full-length sequence to CM model
  (computed by RSEARCH -> default,
  infered from Rfam or provided by user) 
                                 :
                             
                             161.38 
                         
                         
                             Homology estimate
                                   ?  
                                     
Quick homology estimate:
  Not homologous: bit score   20 and bit score > 0.5 * query length
  Uncertain otherwise 
                                 :
                             
                             Homologous 
                         
                     
                 
                
                
                 
                     
                          Estimated full-length sequence:  
                         
                          ? 
                             
Click checkbox to select multiple seuqences.
Fasta header format:
  UID|accession.versionSTRAND start-end 
                         
                     
                     >uid:41|CP018063.1rc 4913796-4914103
ACGGAAGGACGGAAGGCCAAGACCGAAUCGGAAGAGAAGAUUCGAUCUCCCGACUGUUCU
UCCCAGCACGGACACCGAGUACCCACGCGAAGCCGAUGCCACUAUAAGGGCACGAGUGUU
GUGGGCCUGCGAGUUCCGGAUUUUCGAAGACGAGUACCCCGCACCCGUUGCGAGGACGAA
UCGACGAUACCCGGAUCAUCGCCGAGGCCGUUGAAUCACAACCCAUUUCAGCACGCAUGG
UAACUCGGACCGCCCGUGCUAGCGGGUGGCGGAAUCGAUCUCGAUCGAUAUGCCACCCGC
UUUCGCGU
 
                 
                
             
            
             
                
                     
                         
                         
                             TurboFold 
                             
                              ? 
                                 
Visualisation of predicted secondary structure.
To save the image:
  Right click on the image -> Save Image as. 
                             
                         
                     
                
                     
                         
                         
                             rfam-Rc 
                             
                              ? 
                                 
Visualisation of predicted secondary structure.
To save the image:
  Right click on the image -> Save Image as. 
                             
                         
                     
                
                     
                         
                         
                             rnafold 
                             
                              ? 
                                 
Visualisation of predicted secondary structure.
To save the image:
  Right click on the image -> Save Image as. 
                             
                         
                     
                
             
            

        
             
                 Load Sequence viewer 
             
        
        
         
     
    

    
     
         
            Hit: CP016819.1
         
         
             
                 
                     CP016819.1 Rhodococcus sp. p52, complete genome 
                     
                         
 ?  
This is BLAST alignment as read from the input file 
                         
 Score = 98.0 bits (89.7), Expect = 6.42E-14
 Identities = 96/126 (76%), Gaps = 1/126 (1%)
 Strand = Plus/Minus
Query      15 AGGCCAAGACCCAGCCGGAAGAGAAGGCTAGATCTCCCGACCCAAGCTCCTAGCACGGATAC 76     
              ||||||||||| |  |||||||||||  | |||||||||||      ||| |||||||| ||        
Sbjct 1814749 AGGCCAAGACCGAATCGGAAGAGAAGATTCGATCTCCCGACTGTTCTTCCCAGCACGGACAC 1814688

Query      77 CGAGCACCCACGCGGAGCACATGCCGCGGAATAGGCAAAAGTGTTGCGGACCTGCGTAGTTT 138    
              |||| ||||||||| |||  ||||| |   |  ||||  ||||||| || |||||| ||||         
Sbjct 1814687 CGAGTACCCACGCGAAGCCGATGCCACTATAAGGGCACGAGTGTTGTGGGCCTGCG-AGTTC 1814627

Query     139 CG 140    
              ||        
Sbjct 1814626 CG 1814625

 
                 
                
                 
                      Report:  
                     
                         
                             sequence start
                                   ?  
                                     
Start position of the estimated full-length sequence in genome.
Start index  
                                 :
                             
                             1814462 
                         
                         
                             sequence end
                                   ?  
                                     
End position of the estimated full-length sequence in genome.
Start index  
                                 :
                             
                             1814769 
                         
                         
                             bit score (CM)
                                   ?  
                                     
The score for aligning estimated full-length sequence to CM model
  (computed by RSEARCH -> default,
  infered from Rfam or provided by user) 
                                 :
                             
                             161.38 
                         
                         
                             Homology estimate
                                   ?  
                                     
Quick homology estimate:
  Not homologous: bit score   20 and bit score > 0.5 * query length
  Uncertain otherwise 
                                 :
                             
                             Homologous 
                         
                     
                 
                
                
                 
                     
                          Estimated full-length sequence:  
                         
                          ? 
                             
Click checkbox to select multiple seuqences.
Fasta header format:
  UID|accession.versionSTRAND start-end 
                         
                     
                     >uid:42|CP016819.1rc 1814462-1814769
ACGGAAGGACGGAAGGCCAAGACCGAAUCGGAAGAGAAGAUUCGAUCUCCCGACUGUUCU
UCCCAGCACGGACACCGAGUACCCACGCGAAGCCGAUGCCACUAUAAGGGCACGAGUGUU
GUGGGCCUGCGAGUUCCGGAUUUUCGAAGACGAGUACCCCGCACCCGUUGCGAGGACGAA
UCGACGAUACCCGGAUCAUCGCCGAGGCCGUUGAAUCACAACCCAUUUCAGCACGCAUGG
UAACUCGGACCGCCCGUGCUAGCGGGUGGCGGAAUCGAUCUCGAUCGAUAUGCCACCCGC
UUUCGCGU
 
                 
                
             
            
             
                
                     
                         
                         
                             TurboFold 
                             
                              ? 
                                 
Visualisation of predicted secondary structure.
To save the image:
  Right click on the image -> Save Image as. 
                             
                         
                     
                
                     
                         
                         
                             rfam-Rc 
                             
                              ? 
                                 
Visualisation of predicted secondary structure.
To save the image:
  Right click on the image -> Save Image as. 
                             
                         
                     
                
                     
                         
                         
                             rnafold 
                             
                              ? 
                                 
Visualisation of predicted secondary structure.
To save the image:
  Right click on the image -> Save Image as. 
                             
                         
                     
                
             
            

        
             
                 Load Sequence viewer 
             
        
        
         
     
    

    
     
         
            Hit: CP016819.1
         
         
             
                 
                     CP016819.1 Rhodococcus sp. p52, complete genome 
                     
                         
 ?  
This is BLAST alignment as read from the input file 
                         
 Score = 49.0 bits (45.5), Expect = 2.39E+00
 Identities = 43/53 (81%), Gaps = 3/53 (6%)
 Strand = Plus/Minus
Query     218 CAACCCACATC-GCACGCTTGGTCACTCGGG--GTCCGTGCTAGCGGGCGGCG 267    
              |||||||  || |||||| |||| ||||||   | ||||||||||||| ||||        
Sbjct 1814544 CAACCCATTTCAGCACGCATGGTAACTCGGACCGCCCGTGCTAGCGGGTGGCG 1814492

 
                 
                
                 
                      Report:  
                     
                         
                             sequence start
                                   ?  
                                     
Start position of the estimated full-length sequence in genome.
Start index  
                                 :
                             
                             1814454 
                         
                         
                             sequence end
                                   ?  
                                     
End position of the estimated full-length sequence in genome.
Start index  
                                 :
                             
                             1814761 
                         
                         
                             bit score (CM)
                                   ?  
                                     
The score for aligning estimated full-length sequence to CM model
  (computed by RSEARCH -> default,
  infered from Rfam or provided by user) 
                                 :
                             
                             161.38 
                         
                         
                             Homology estimate
                                   ?  
                                     
Quick homology estimate:
  Not homologous: bit score   20 and bit score > 0.5 * query length
  Uncertain otherwise 
                                 :
                             
                             Homologous 
                         
                     
                 
                
                
                 
                     
                          Estimated full-length sequence:  
                         
                          ? 
                             
Click checkbox to select multiple seuqences.
Fasta header format:
  UID|accession.versionSTRAND start-end 
                         
                     
                     >uid:43|CP016819.1rc 1814454-1814761
ACGGAAGGACGGAAGGCCAAGACCGAAUCGGAAGAGAAGAUUCGAUCUCCCGACUGUUCU
UCCCAGCACGGACACCGAGUACCCACGCGAAGCCGAUGCCACUAUAAGGGCACGAGUGUU
GUGGGCCUGCGAGUUCCGGAUUUUCGAAGACGAGUACCCCGCACCCGUUGCGAGGACGAA
UCGACGAUACCCGGAUCAUCGCCGAGGCCGUUGAAUCACAACCCAUUUCAGCACGCAUGG
UAACUCGGACCGCCCGUGCUAGCGGGUGGCGGAAUCGAUCUCGAUCGAUAUGCCACCCGC
UUUCGCGU
 
                 
                
             
            
             
                
                     
                         
                         
                             TurboFold 
                             
                              ? 
                                 
Visualisation of predicted secondary structure.
To save the image:
  Right click on the image -> Save Image as. 
                             
                         
                     
                
                     
                         
                         
                             rfam-Rc 
                             
                              ? 
                                 
Visualisation of predicted secondary structure.
To save the image:
  Right click on the image -> Save Image as. 
                             
                         
                     
                
                     
                         
                         
                             rnafold 
                             
                              ? 
                                 
Visualisation of predicted secondary structure.
To save the image:
  Right click on the image -> Save Image as. 
                             
                         
                     
                
             
            

        
             
                 Load Sequence viewer 
             
        
        
         
     
    

    
     
         
            Hit: CP006996.1
         
         
             
                 
                     CP006996.1 Rhodococcus pyridinivorans SB3094, complete genome 
                     
                         
 ?  
This is BLAST alignment as read from the input file 
                         
 Score = 98.0 bits (89.7), Expect = 6.42E-14
 Identities = 96/126 (76%), Gaps = 1/126 (1%)
 Strand = Plus/Minus
Query      15 AGGCCAAGACCCAGCCGGAAGAGAAGGCTAGATCTCCCGACCCAAGCTCCTAGCACGGATAC 76     
              ||||||||||| |  |||||||||||  | |||||||||||      ||| |||||||| ||        
Sbjct 3958186 AGGCCAAGACCGAATCGGAAGAGAAGATTCGATCTCCCGACTGTTCTTCCCAGCACGGACAC 3958125

Query      77 CGAGCACCCACGCGGAGCACATGCCGCGGAATAGGCAAAAGTGTTGCGGACCTGCGTAGTTT 138    
              |||| ||||||||| |||  ||||| |   |  ||||  ||||||| || |||||| ||||         
Sbjct 3958124 CGAGTACCCACGCGAAGCCGATGCCACTATAAGGGCACGAGTGTTGTGGGCCTGCG-AGTTC 3958064

Query     139 CG 140    
              ||        
Sbjct 3958063 CG 3958062

 
                 
                
                 
                      Report:  
                     
                         
                             sequence start
                                   ?  
                                     
Start position of the estimated full-length sequence in genome.
Start index  
                                 :
                             
                             3957899 
                         
                         
                             sequence end
                                   ?  
                                     
End position of the estimated full-length sequence in genome.
Start index  
                                 :
                             
                             3958206 
                         
                         
                             bit score (CM)
                                   ?  
                                     
The score for aligning estimated full-length sequence to CM model
  (computed by RSEARCH -> default,
  infered from Rfam or provided by user) 
                                 :
                             
                             161.38 
                         
                         
                             Homology estimate
                                   ?  
                                     
Quick homology estimate:
  Not homologous: bit score   20 and bit score > 0.5 * query length
  Uncertain otherwise 
                                 :
                             
                             Homologous 
                         
                     
                 
                
                
                 
                     
                          Estimated full-length sequence:  
                         
                          ? 
                             
Click checkbox to select multiple seuqences.
Fasta header format:
  UID|accession.versionSTRAND start-end 
                         
                     
                     >uid:44|CP006996.1rc 3957899-3958206
ACGGAAGGACGGAAGGCCAAGACCGAAUCGGAAGAGAAGAUUCGAUCUCCCGACUGUUCU
UCCCAGCACGGACACCGAGUACCCACGCGAAGCCGAUGCCACUAUAAGGGCACGAGUGUU
GUGGGCCUGCGAGUUCCGGAUUUUCGAAGACGAGUACCCCGCACCCGUUGCGAGGACGAA
UCGACGAUACCCGGAUCAUCGCCGAGGCCGUUGAAUCACAACCCAUUUCAGCACGCAUGG
UAACUCGGACCGCCCGUGCUAGCGGGUGGCGGAAUCGAUCUCGAUCGAUAUGCCACCCGC
UUUCGCGU
 
                 
                
             
            
             
                
                     
                         
                         
                             TurboFold 
                             
                              ? 
                                 
Visualisation of predicted secondary structure.
To save the image:
  Right click on the image -> Save Image as. 
                             
                         
                     
                
                     
                         
                         
                             rfam-Rc 
                             
                              ? 
                                 
Visualisation of predicted secondary structure.
To save the image:
  Right click on the image -> Save Image as. 
                             
                         
                     
                
                     
                         
                         
                             rnafold 
                             
                              ? 
                                 
Visualisation of predicted secondary structure.
To save the image:
  Right click on the image -> Save Image as. 
                             
                         
                     
                
             
            

        
             
                 Load Sequence viewer 
             
        
        
         
     
    

    
     
         
            Hit: CP006996.1
         
         
             
                 
                     CP006996.1 Rhodococcus pyridinivorans SB3094, complete genome 
                     
                         
 ?  
This is BLAST alignment as read from the input file 
                         
 Score = 49.0 bits (45.5), Expect = 2.39E+00
 Identities = 43/53 (81%), Gaps = 3/53 (6%)
 Strand = Plus/Minus
Query     218 CAACCCACATC-GCACGCTTGGTCACTCGGG--GTCCGTGCTAGCGGGCGGCG 267    
              |||||||  || |||||| |||| ||||||   | ||||||||||||| ||||        
Sbjct 3957981 CAACCCATTTCAGCACGCATGGTAACTCGGACCGCCCGTGCTAGCGGGTGGCG 3957929

 
                 
                
                 
                      Report:  
                     
                         
                             sequence start
                                   ?  
                                     
Start position of the estimated full-length sequence in genome.
Start index  
                                 :
                             
                             3957891 
                         
                         
                             sequence end
                                   ?  
                                     
End position of the estimated full-length sequence in genome.
Start index  
                                 :
                             
                             3958198 
                         
                         
                             bit score (CM)
                                   ?  
                                     
The score for aligning estimated full-length sequence to CM model
  (computed by RSEARCH -> default,
  infered from Rfam or provided by user) 
                                 :
                             
                             161.38 
                         
                         
                             Homology estimate
                                   ?  
                                     
Quick homology estimate:
  Not homologous: bit score   20 and bit score > 0.5 * query length
  Uncertain otherwise 
                                 :
                             
                             Homologous 
                         
                     
                 
                
                
                 
                     
                          Estimated full-length sequence:  
                         
                          ? 
                             
Click checkbox to select multiple seuqences.
Fasta header format:
  UID|accession.versionSTRAND start-end 
                         
                     
                     >uid:45|CP006996.1rc 3957891-3958198
ACGGAAGGACGGAAGGCCAAGACCGAAUCGGAAGAGAAGAUUCGAUCUCCCGACUGUUCU
UCCCAGCACGGACACCGAGUACCCACGCGAAGCCGAUGCCACUAUAAGGGCACGAGUGUU
GUGGGCCUGCGAGUUCCGGAUUUUCGAAGACGAGUACCCCGCACCCGUUGCGAGGACGAA
UCGACGAUACCCGGAUCAUCGCCGAGGCCGUUGAAUCACAACCCAUUUCAGCACGCAUGG
UAACUCGGACCGCCCGUGCUAGCGGGUGGCGGAAUCGAUCUCGAUCGAUAUGCCACCCGC
UUUCGCGU
 
                 
                
             
            
             
                
                     
                         
                         
                             TurboFold 
                             
                              ? 
                                 
Visualisation of predicted secondary structure.
To save the image:
  Right click on the image -> Save Image as. 
                             
                         
                     
                
                     
                         
                         
                             rfam-Rc 
                             
                              ? 
                                 
Visualisation of predicted secondary structure.
To save the image:
  Right click on the image -> Save Image as. 
                             
                         
                     
                
                     
                         
                         
                             rnafold 
                             
                              ? 
                                 
Visualisation of predicted secondary structure.
To save the image:
  Right click on the image -> Save Image as. 
                             
                         
                     
                
             
            

        
             
                 Load Sequence viewer 
             
        
        
         
     
    

    
     
         
            Hit: CP041695.1
         
         
             
                 
                     CP041695.1 Nocardia otitidiscaviarum strain NEB252 chromosome, complete genome 
                     
                         
 ?  
This is BLAST alignment as read from the input file 
                         
 Score = 95.0 bits (86.9), Expect = 7.82E-13
 Identities = 184/264 (70%), Gaps = 17/264 (6%)
 Strand = Plus/Plus
Query      15 AGGCCAAGACCCAGCCGGAAGAGAAGGCTAGATCTCCCGACCCAAGCT-CCTAGCACGGATA 75     
              ||||||||| |    ||||||||||||    |||||||  ||||  || || |||||||| |        
Sbjct 6582331 AGGCCAAGATCGGATCGGAAGAGAAGGTCCTATCTCCCATCCCACCCTTCCCAGCACGGACA 6582392

Query      76 CCGAGCACCCACGCGGAGCACATGCCGCGGAATAGGCAAAAGTGTTGCGGACCTGCGTAGTT 137    
              ||  |||||||||||||||||  ||||||  |  ||||  ||||||| || |||||||   |        
Sbjct 6582393 CCAGGCACCCACGCGGAGCAC--GCCGCGACAAGGGCACGAGTGTTGTGGGCCTGCGT-TAT 6582451

Query     138 TCGAAAAGCGGACGGCCACGACGGCC-----CTTTGGGTGGGGTTGCAGCCGTAGCGCATCG 194    
               ||  |  |   || | | |||||||     |     |||||| || |  ||  | |   ||        
Sbjct 6582452 CCGTGATTC---CGTCGATGACGGCCCCGCACACGCTGTGGGGATG-AATCGGCGGGGTCCG 6582509

Query     195 CAAAG-ACGCCGAGGTC-ACCCACGCAACCC-ACATCGCACGCTTGGTCACTCGG-GGTCCG 252    
               | ||  |||||||| | |  ||| |||||| | |  ||||||||||| ||  || ||||||        
Sbjct 6582510 GAGAGCTCGCCGAGGCCGAATCACACAACCCGATACAGCACGCTTGGTAACCGGGTGGTCCG 6582571

Query     253 TGCTAGCGGGCGGCGA 268    
              ||||||||||||| ||        
Sbjct 6582572 TGCTAGCGGGCGGTGA 6582587

 
                 
                
                 
                      Report:  
                     
                         
                             sequence start
                                   ?  
                                     
Start position of the estimated full-length sequence in genome.
Start index  
                                 :
                             
                             6582317 
                         
                         
                             sequence end
                                   ?  
                                     
End position of the estimated full-length sequence in genome.
Start index  
                                 :
                             
                             6582622 
                         
                         
                             bit score (CM)
                                   ?  
                                     
The score for aligning estimated full-length sequence to CM model
  (computed by RSEARCH -> default,
  infered from Rfam or provided by user) 
                                 :
                             
                             127.81 
                         
                         
                             Homology estimate
                                   ?  
                                     
Quick homology estimate:
  Not homologous: bit score   20 and bit score > 0.5 * query length
  Uncertain otherwise 
                                 :
                             
                             Uncertain 
                         
                     
                 
                
                
                 
                     
                          Estimated full-length sequence:  
                         
                          ? 
                             
Click checkbox to select multiple seuqences.
Fasta header format:
  UID|accession.versionSTRAND start-end 
                         
                     
                     >uid:46|CP041695.1fw 6582317-6582622
CAAGGAAGGACGGAAGGCCAAGAUCGGAUCGGAAGAGAAGGUCCUAUCUCCCAUCCCACC
CUUCCCAGCACGGACACCAGGCACCCACGCGGAGCACGCCGCGACAAGGGCACGAGUGUU
GUGGGCCUGCGUUAUCCGUGAUUCCGUCGAUGACGGCCCCGCACACGCUGUGGGGAUGAA
UCGGCGGGGUCCGGAGAGCUCGCCGAGGCCGAAUCACACAACCCGAUACAGCACGCUUGG
UAACCGGGUGGUCCGUGCUAGCGGGCGGUGAGGUCGCAACAGCGACAACGCCGCCCGCUC
UGAUGU
 
                 
                
             
            
             
                
                     
                         
                         
                             TurboFold 
                             
                              ? 
                                 
Visualisation of predicted secondary structure.
To save the image:
  Right click on the image -> Save Image as. 
                             
                         
                     
                
                     
                         
                         
                             rfam-Rc 
                             
                              ? 
                                 
Visualisation of predicted secondary structure.
To save the image:
  Right click on the image -> Save Image as. 
                             
                         
                     
                
                     
                         
                         
                             rnafold 
                             
                              ? 
                                 
Visualisation of predicted secondary structure.
To save the image:
  Right click on the image -> Save Image as. 
                             
                         
                     
                
             
            

        
             
                 Load Sequence viewer 
             
        
        
         
     
    

    
     
         
            Hit: CP008947.1
         
         
             
                 
                     CP008947.1 Rhodococcus opacus strain R7 sequence 
                     
                         
 ?  
This is BLAST alignment as read from the input file 
                         
 Score = 94.0 bits (86.0), Expect = 7.82E-13
 Identities = 194/287 (68%), Gaps = 5/287 (2%)
 Strand = Plus/Plus
Query      15 AGGCCAAGACCCAGCCGGAAGAGAAGGCTAGATCTCCCGACCCAAGCTCCTAGCACGGATAC 76     
              ||||||||  | | || |||||||||| | |||||||| |||     ||| |||||||  ||        
Sbjct 4334485 AGGCCAAGGTCGACCCAGAAGAGAAGGATCGATCTCCCAACCAGGATTCCCAGCACGGGCAC 4334546

Query      77 CGAGCACCCACGCGGAGCACATGCCGCGGAATAGGCAAAAGTGTTGCGGACCTGCGTAGTTT 138    
                 | ||||||||| |||| | ||| |   |  |||| ||| |||| || |||||| ||  |        
Sbjct 4334547 TAGGTACCCACGCGAAGCATAAGCCACTATAAGGGCAGAAGCGTTGTGGGCCTGCG-AGACT 4334607

Query     139 CGAAAAGCGGACGGCCACGACGGCCCTTTGGGTGG--GGTTGCAGCCGTAGCGCATCGCAAA 198    
              |||        |||| | |||    |   || ||   ||  | | |||  |    ||| |          
Sbjct 4334608 CGAGTCTTCACCGGCAAGGACCCTGCACAGGTTGCAAGGACG-ACCCGACGATATTCGGACT 4334668

Query     199 GACGCCGAGGTCACCCACGCAACCCACATCGCACGCTTGGTCACTCGGGGTCCGTGCTAGCG 260    
                |||||||| ||||||| |||||||| | ||||||||||| ||  |  |||||||||| ||        
Sbjct 4334669 CTCGCCGAGGCCACCCACACAACCCACCTTGCACGCTTGGTAACCGGTTGTCCGTGCTAACG 4334730

Query     261 GGCGGCGAACCGGACA-TTGTCCGGGACGCCGCCCGATT 298    
              |||||||||   ||||   ||| |   ||||||||| ||        
Sbjct 4334731 GGCGGCGAAGTCGACACCAGTCGGCAGCGCCGCCCGTTT 4334769

 
                 
                
                 
                      Report:  
                     
                         
                             sequence start
                                   ?  
                                     
Start position of the estimated full-length sequence in genome.
Start index  
                                 :
                             
                             4334472 
                         
                         
                             sequence end
                                   ?  
                                     
End position of the estimated full-length sequence in genome.
Start index  
                                 :
                             
                             4334774 
                         
                         
                             bit score (CM)
                                   ?  
                                     
The score for aligning estimated full-length sequence to CM model
  (computed by RSEARCH -> default,
  infered from Rfam or provided by user) 
                                 :
                             
                             148.67 
                         
                         
                             Homology estimate
                                   ?  
                                     
Quick homology estimate:
  Not homologous: bit score   20 and bit score > 0.5 * query length
  Uncertain otherwise 
                                 :
                             
                             Uncertain 
                         
                     
                 
                
                
                 
                     
                          Estimated full-length sequence:  
                         
                          ? 
                             
Click checkbox to select multiple seuqences.
Fasta header format:
  UID|accession.versionSTRAND start-end 
                         
                     
                     >uid:47|CP008947.1fw 4334472-4334774
ACGGAAGAACGGAAGGCCAAGGUCGACCCAGAAGAGAAGGAUCGAUCUCCCAACCAGGAU
UCCCAGCACGGGCACUAGGUACCCACGCGAAGCAUAAGCCACUAUAAGGGCAGAAGCGUU
GUGGGCCUGCGAGACUCGAGUCUUCACCGGCAAGGACCCUGCACAGGUUGCAAGGACGAC
CCGACGAUAUUCGGACUCUCGCCGAGGCCACCCACACAACCCACCUUGCACGCUUGGUAA
CCGGUUGUCCGUGCUAACGGGCGGCGAAGUCGACACCAGUCGGCAGCGCCGCCCGUUUCA
CGU
 
                 
                
             
            
             
                
                     
                         
                         
                             TurboFold 
                             
                              ? 
                                 
Visualisation of predicted secondary structure.
To save the image:
  Right click on the image -> Save Image as. 
                             
                         
                     
                
                     
                         
                         
                             rfam-Rc 
                             
                              ? 
                                 
Visualisation of predicted secondary structure.
To save the image:
  Right click on the image -> Save Image as. 
                             
                         
                     
                
                     
                         
                         
                             rnafold 
                             
                              ? 
                                 
Visualisation of predicted secondary structure.
To save the image:
  Right click on the image -> Save Image as. 
                             
                         
                     
                
             
            

        
             
                 Load Sequence viewer 
             
        
        
         
     
    

    
     
         
            Hit: CP025959.1
         
         
             
                 
                     CP025959.1 Rhodococcus sp. djl-6-2 chromosome, complete genome 
                     
                         
 ?  
This is BLAST alignment as read from the input file 
                         
 Score = 93.0 bits (85.1), Expect = 2.73E-12
 Identities = 171/247 (69%), Gaps = 14/247 (6%)
 Strand = Plus/Minus
Query     30 CGGAAGAGAAGGCTAGATCTCCCGACCCAAGCTCCTAGCACGGATACC-GAGCACCCACGCGGA 92    
             ||||||||||||   | |  ||| ||| ||  |||||||||||  ||  | |||||||||||||       
Sbjct 591434 CGGAAGAGAAGGACCGTTTCCCCAACCGAAATTCCTAGCACGGGCACTAGGGCACCCACGCGGA 591371

Query     93 GCACATGCCGCGGAATAGGCAAAAGTGTTGCGGACCTGCGTAGTTTCGAA-AAGCGGACGGCCA 155   
             ||||| ||| |   |  |||| ||| |||| || |||||| || ||||   ||   ||  || |       
Sbjct 591370 GCACAAGCCACTATAAGGGCAGAAGCGTTGTGGGCCTGCG-AGATTCGTTCAATTTGA--GCGA 591310

Query    156 CGACGGCCCTTTGGGTGGGGTTGCAGCCGTAG----CGC-ATCGCAAAGACGCCGAGGTCACCC 214   
              |||  | |   || || ||   |  ||  ||    ||| ||| |     |||||||| |||||       
Sbjct 591309 GGACCCCGCACAGGTTGCGGGGACGACCTCAGAAAGCGCGATCCCT----CGCCGAGGCCACCC 591250

Query    215 ACGCAACCCACATCGCACGCTTGGTCACTCGGGGTCCGTGCTAGCGGGCGGCGAA 269   
             || ||||||||   ||||||||||| ||  |  |||||||||  |||||||||||       
Sbjct 591249 ACACAACCCACCGAGCACGCTTGGTAACGAGTAGTCCGTGCTGACGGGCGGCGAA 591195

 
                 
                
                 
                      Report:  
                     
                         
                             sequence start
                                   ?  
                                     
Start position of the estimated full-length sequence in genome.
Start index  
                                 :
                             
                             591160 
                         
                         
                             sequence end
                                   ?  
                                     
End position of the estimated full-length sequence in genome.
Start index  
                                 :
                             
                             591461 
                         
                         
                             bit score (CM)
                                   ?  
                                     
The score for aligning estimated full-length sequence to CM model
  (computed by RSEARCH -> default,
  infered from Rfam or provided by user) 
                                 :
                             
                             135.59 
                         
                         
                             Homology estimate
                                   ?  
                                     
Quick homology estimate:
  Not homologous: bit score   20 and bit score > 0.5 * query length
  Uncertain otherwise 
                                 :
                             
                             Uncertain 
                         
                     
                 
                
                
                 
                     
                          Estimated full-length sequence:  
                         
                          ? 
                             
Click checkbox to select multiple seuqences.
Fasta header format:
  UID|accession.versionSTRAND start-end 
                         
                     
                     >uid:48|CP025959.1rc 591160-591461
UACGGAAGAACGGAAGGCCAGGAACGGCUCGGAAGAGAAGGACCGUUUCCCCAACCGAAA
UUCCUAGCACGGGCACUAGGGCACCCACGCGGAGCACAAGCCACUAUAAGGGCAGAAGCG
UUGUGGGCCUGCGAGAUUCGUUCAAUUUGAGCGAGGACCCCGCACAGGUUGCGGGGACGA
CCUCAGAAAGCGCGAUCCCUCGCCGAGGCCACCCACACAACCCACCGAGCACGCUUGGUA
ACGAGUAGUCCGUGCUGACGGGCGGCGAAGUCGAUUUAUCGGCAGCGCCGCCCGUUCGUU
AU
 
                 
                
             
            
             
                
                     
                         
                         
                             TurboFold 
                             
                              ? 
                                 
Visualisation of predicted secondary structure.
To save the image:
  Right click on the image -> Save Image as. 
                             
                         
                     
                
                     
                         
                         
                             rfam-Rc 
                             
                              ? 
                                 
Visualisation of predicted secondary structure.
To save the image:
  Right click on the image -> Save Image as. 
                             
                         
                     
                
                     
                         
                         
                             rnafold 
                             
                              ? 
                                 
Visualisation of predicted secondary structure.
To save the image:
  Right click on the image -> Save Image as. 
                             
                         
                     
                
             
            

        
             
                 Load Sequence viewer 
             
        
        
         
     
    

    
     
         
            Hit: CP017299.1
         
         
             
                 
                     CP017299.1 Rhodococcus sp. YL-1, complete genome 
                     
                         
 ?  
This is BLAST alignment as read from the input file 
                         
 Score = 93.0 bits (85.1), Expect = 2.73E-12
 Identities = 171/247 (69%), Gaps = 14/247 (6%)
 Strand = Plus/Minus
Query     30 CGGAAGAGAAGGCTAGATCTCCCGACCCAAGCTCCTAGCACGGATACC-GAGCACCCACGCGGA 92    
             ||||||||||||   | |  ||| ||| ||  |||||||||||  ||  | |||||||||||||       
Sbjct 573559 CGGAAGAGAAGGACCGTTTCCCCAACCGAAATTCCTAGCACGGGCACTAGGGCACCCACGCGGA 573496

Query     93 GCACATGCCGCGGAATAGGCAAAAGTGTTGCGGACCTGCGTAGTTTCGAA-AAGCGGACGGCCA 155   
             ||||| ||| |   |  |||| ||| |||| || |||||| || ||||   ||   ||  || |       
Sbjct 573495 GCACAAGCCACTATAAGGGCAGAAGCGTTGTGGGCCTGCG-AGATTCGTTCAATTTGA--GCGA 573435

Query    156 CGACGGCCCTTTGGGTGGGGTTGCAGCCGTAG----CGC-ATCGCAAAGACGCCGAGGTCACCC 214   
              |||  | |   || || ||   |  ||  ||    ||| ||| |     |||||||| |||||       
Sbjct 573434 GGACCCCGCACAGGTTGCGGGGACGACCTCAGAAAGCGCGATCCCT----CGCCGAGGCCACCC 573375

Query    215 ACGCAACCCACATCGCACGCTTGGTCACTCGGGGTCCGTGCTAGCGGGCGGCGAA 269   
             || ||||||||   ||||||||||| ||  |  |||||||||  |||||||||||       
Sbjct 573374 ACACAACCCACCGAGCACGCTTGGTAACGAGTAGTCCGTGCTGACGGGCGGCGAA 573320

 
                 
                
                 
                      Report:  
                     
                         
                             sequence start
                                   ?  
                                     
Start position of the estimated full-length sequence in genome.
Start index  
                                 :
                             
                             573285 
                         
                         
                             sequence end
                                   ?  
                                     
End position of the estimated full-length sequence in genome.
Start index  
                                 :
                             
                             573586 
                         
                         
                             bit score (CM)
                                   ?  
                                     
The score for aligning estimated full-length sequence to CM model
  (computed by RSEARCH -> default,
  infered from Rfam or provided by user) 
                                 :
                             
                             135.59 
                         
                         
                             Homology estimate
                                   ?  
                                     
Quick homology estimate:
  Not homologous: bit score   20 and bit score > 0.5 * query length
  Uncertain otherwise 
                                 :
                             
                             Uncertain 
                         
                     
                 
                
                
                 
                     
                          Estimated full-length sequence:  
                         
                          ? 
                             
Click checkbox to select multiple seuqences.
Fasta header format:
  UID|accession.versionSTRAND start-end 
                         
                     
                     >uid:49|CP017299.1rc 573285-573586
UACGGAAGAACGGAAGGCCAGGAACGGCUCGGAAGAGAAGGACCGUUUCCCCAACCGAAA
UUCCUAGCACGGGCACUAGGGCACCCACGCGGAGCACAAGCCACUAUAAGGGCAGAAGCG
UUGUGGGCCUGCGAGAUUCGUUCAAUUUGAGCGAGGACCCCGCACAGGUUGCGGGGACGA
CCUCAGAAAGCGCGAUCCCUCGCCGAGGCCACCCACACAACCCACCGAGCACGCUUGGUA
ACGAGUAGUCCGUGCUGACGGGCGGCGAAGUCGAUUUAUCGGCAGCGCCGCCCGUUCGUU
AU
 
                 
                
             
            
             
                
                     
                         
                         
                             TurboFold 
                             
                              ? 
                                 
Visualisation of predicted secondary structure.
To save the image:
  Right click on the image -> Save Image as. 
                             
                         
                     
                
                     
                         
                         
                             rfam-Rc 
                             
                              ? 
                                 
Visualisation of predicted secondary structure.
To save the image:
  Right click on the image -> Save Image as. 
                             
                         
                     
                
                     
                         
                         
                             rnafold 
                             
                              ? 
                                 
Visualisation of predicted secondary structure.
To save the image:
  Right click on the image -> Save Image as. 
                             
                         
                     
                
             
            

        
             
                 Load Sequence viewer 
             
        
        
         
     
    

    
     
         
            Hit: CP012749.1
         
         
             
                 
                     CP012749.1 Rhodococcus sp. 008, complete genome 
                     
                         
 ?  
This is BLAST alignment as read from the input file 
                         
 Score = 93.0 bits (85.1), Expect = 2.73E-12
 Identities = 171/247 (69%), Gaps = 14/247 (6%)
 Strand = Plus/Minus
Query      30 CGGAAGAGAAGGCTAGATCTCCCGACCCAAGCTCCTAGCACGGATACC-GAGCACCCACGCG 90     
              ||||||||||||   | |  ||| ||| ||  |||||||||||  ||  | |||||||||||        
Sbjct 2811539 CGGAAGAGAAGGACCGTTTCCCCAACCGAAATTCCTAGCACGGGCACTAGGGCACCCACGCG 2811478

Query      91 GAGCACATGCCGCGGAATAGGCAAAAGTGTTGCGGACCTGCGTAGTTTCGAA-AAGCGGACG 151    
              ||||||| ||| |   |  |||| ||| |||| || |||||| || ||||   ||   ||          
Sbjct 2811477 GAGCACAAGCCACTATAAGGGCAGAAGCGTTGTGGGCCTGCG-AGATTCGTTCAATTTGA-- 2811419

Query     152 GCCACGACGGCCCTTTGGGTGGGGTTGCAGCCGTAG----CGC-ATCGCAAAGACGCCGAGG 208    
              || | |||  | |   || || ||   |  ||  ||    ||| ||| |     ||||||||        
Sbjct 2811418 GCGAGGACCCCGCACAGGTTGCGGGGACGACCTCAGAAAGCGCGATCCCT----CGCCGAGG 2811361

Query     209 TCACCCACGCAACCCACATCGCACGCTTGGTCACTCGGGGTCCGTGCTAGCGGGCGGCGAA 269    
               ||||||| ||||||||   ||||||||||| ||  |  |||||||||  |||||||||||        
Sbjct 2811360 CCACCCACACAACCCACCGAGCACGCTTGGTAACGAGTAGTCCGTGCTGACGGGCGGCGAA 2811300

 
                 
                
                 
                      Report:  
                     
                         
                             sequence start
                                   ?  
                                     
Start position of the estimated full-length sequence in genome.
Start index  
                                 :
                             
                             2811265 
                         
                         
                             sequence end
                                   ?  
                                     
End position of the estimated full-length sequence in genome.
Start index  
                                 :
                             
                             2811566 
                         
                         
                             bit score (CM)
                                   ?  
                                     
The score for aligning estimated full-length sequence to CM model
  (computed by RSEARCH -> default,
  infered from Rfam or provided by user) 
                                 :
                             
                             135.59 
                         
                         
                             Homology estimate
                                   ?  
                                     
Quick homology estimate:
  Not homologous: bit score   20 and bit score > 0.5 * query length
  Uncertain otherwise 
                                 :
                             
                             Uncertain 
                         
                     
                 
                
                
                 
                     
                          Estimated full-length sequence:  
                         
                          ? 
                             
Click checkbox to select multiple seuqences.
Fasta header format:
  UID|accession.versionSTRAND start-end 
                         
                     
                     >uid:50|CP012749.1rc 2811265-2811566
UACGGAAGAACGGAAGGCCAGGAACGGCUCGGAAGAGAAGGACCGUUUCCCCAACCGAAA
UUCCUAGCACGGGCACUAGGGCACCCACGCGGAGCACAAGCCACUAUAAGGGCAGAAGCG
UUGUGGGCCUGCGAGAUUCGUUCAAUUUGAGCGAGGACCCCGCACAGGUUGCGGGGACGA
CCUCAGAAAGCGCGAUCCCUCGCCGAGGCCACCCACACAACCCACCGAGCACGCUUGGUA
ACGAGUAGUCCGUGCUGACGGGCGGCGAAGUCGAUUUAUCGGCAGCGCCGCCCGUUCGUU
AU
 
                 
                
             
            
             
                
                     
                         
                         
                             TurboFold 
                             
                              ? 
                                 
Visualisation of predicted secondary structure.
To save the image:
  Right click on the image -> Save Image as. 
                             
                         
                     
                
                     
                         
                         
                             rfam-Rc 
                             
                              ? 
                                 
Visualisation of predicted secondary structure.
To save the image:
  Right click on the image -> Save Image as. 
                             
                         
                     
                
                     
                         
                         
                             rnafold 
                             
                              ? 
                                 
Visualisation of predicted secondary structure.
To save the image:
  Right click on the image -> Save Image as. 
                             
                         
                     
                
             
            

        
             
                 Load Sequence viewer 
             
        
        
         
     
    

    
     
         
            Hit: CP029297.1
         
         
             
                 
                     CP029297.1 Rhodococcus qingshengii strain IGTS8 chromosome 
                     
                         
 ?  
This is BLAST alignment as read from the input file 
                         
 Score = 88.0 bits (80.6), Expect = 3.32E-11
 Identities = 170/247 (69%), Gaps = 14/247 (6%)
 Strand = Plus/Minus
Query     30 CGGAAGAGAAGGCTAGATCTCCCGACCCAAGCTCCTAGCACGGATACC-GAGCACCCACGCGGA 92    
             ||||||||||||   | |  ||| ||| ||  |||||||||||  ||  | |||||||||||||       
Sbjct 566268 CGGAAGAGAAGGACCGTTTCCCCAACCGAAATTCCTAGCACGGGCACTAGGGCACCCACGCGGA 566205

Query     93 GCACATGCCGCGGAATAGGCAAAAGTGTTGCGGACCTGCGTAGTTTCGAA-AAGCGGACGGCCA 155   
             ||||| ||| |   |  |||| ||| |||| || |||||| || ||||   ||   ||  || |       
Sbjct 566204 GCACAAGCCACTATAAGGGCAGAAGCGTTGTGGGCCTGCG-AGATTCGTTCAATTTGA--GCGA 566144

Query    156 CGACGGCCCTTTGGGTGGGGTTGCAGCCGTAGC----GC-ATCGCAAAGACGCCGAGGTCACCC 214   
              |||  | |   || || ||   |  ||  ||     || ||| |     |||||||| |||||       
Sbjct 566143 GGACCCCGCACAGGTTGCGGGGACGACCTCAGAAAGTGCGATCCCT----CGCCGAGGCCACCC 566084

Query    215 ACGCAACCCACATCGCACGCTTGGTCACTCGGGGTCCGTGCTAGCGGGCGGCGAA 269   
             || ||||||||   ||||||||||| ||  |  |||||||||  |||||||||||       
Sbjct 566083 ACACAACCCACCGAGCACGCTTGGTAACGAGTAGTCCGTGCTGACGGGCGGCGAA 566029

 
                 
                
                 
                      Report:  
                     
                         
                             sequence start
                                   ?  
                                     
Start position of the estimated full-length sequence in genome.
Start index  
                                 :
                             
                             565995 
                         
                         
                             sequence end
                                   ?  
                                     
End position of the estimated full-length sequence in genome.
Start index  
                                 :
                             
                             566296 
                         
                         
                             bit score (CM)
                                   ?  
                                     
The score for aligning estimated full-length sequence to CM model
  (computed by RSEARCH -> default,
  infered from Rfam or provided by user) 
                                 :
                             
                             146.09 
                         
                         
                             Homology estimate
                                   ?  
                                     
Quick homology estimate:
  Not homologous: bit score   20 and bit score > 0.5 * query length
  Uncertain otherwise 
                                 :
                             
                             Uncertain 
                         
                     
                 
                
                
                 
                     
                          Estimated full-length sequence:  
                         
                          ? 
                             
Click checkbox to select multiple seuqences.
Fasta header format:
  UID|accession.versionSTRAND start-end 
                         
                     
                     >uid:51|CP029297.1rc 565995-566296
ACGGAAGAACGGAAGGCCAGGAACGGCUCGGAAGAGAAGGACCGUUUCCCCAACCGAAAU
UCCUAGCACGGGCACUAGGGCACCCACGCGGAGCACAAGCCACUAUAAGGGCAGAAGCGU
UGUGGGCCUGCGAGAUUCGUUCAAUUUGAGCGAGGACCCCGCACAGGUUGCGGGGACGAC
CUCAGAAAGUGCGAUCCCUCGCCGAGGCCACCCACACAACCCACCGAGCACGCUUGGUAA
CGAGUAGUCCGUGCUGACGGGCGGCGAAGUCGAUUUAUCGGCAGCGCCGCCCGUUCGUUA
UG
 
                 
                
             
            
             
                
                     
                         
                         
                             TurboFold 
                             
                              ? 
                                 
Visualisation of predicted secondary structure.
To save the image:
  Right click on the image -> Save Image as. 
                             
                         
                     
                
                     
                         
                         
                             rfam-Rc 
                             
                              ? 
                                 
Visualisation of predicted secondary structure.
To save the image:
  Right click on the image -> Save Image as. 
                             
                         
                     
                
                     
                         
                         
                             rnafold 
                             
                              ? 
                                 
Visualisation of predicted secondary structure.
To save the image:
  Right click on the image -> Save Image as. 
                             
                         
                     
                
             
            

        
             
                 Load Sequence viewer 
             
        
        
         
     
    

    
     
         
            Hit: CP035319.1
         
         
             
                 
                     CP035319.1 Rhodococcus sp. ABRD24 chromosome, complete genome 
                     
                         
 ?  
This is BLAST alignment as read from the input file 
                         
 Score = 88.0 bits (80.6), Expect = 3.32E-11
 Identities = 181/265 (68%), Gaps = 23/265 (9%)
 Strand = Plus/Minus
Query      16 GGCCAAGACCCAGCCGGAAGAGAAGGCTAGATCTCCCGACCCAAGCTCCTAGCACGGATACC 77     
              ||||| || | |  |||||||||||||| |||  ||||| |  |  ||| |||||||| |||        
Sbjct 3259575 GGCCAGGATCGACTCGGAAGAGAAGGCTCGATTCCCCGAACGGACTTCCCAGCACGGACACC 3259514

Query      78 GAGCACCCACGCGGAGC-ACATGCCGCGGAATAGGCAAAAGTGTTGCGGACCTGCGTAGTTT 138    
                |||||||||||||||  || ||| |   |  |||| ||| |||| || ||||||||   |        
Sbjct 3259513 AGGCACCCACGCGGAGCTGCAAGCCACTATAAGGGCAGAAGCGTTGTGGGCCTGCGTA-ACT 3259453

Query     139 CGAAAAGCG--GACGGCCACGACGGCCCTTTGGGTGGGGTTGCAG-------CCGTAGCGCA 191    
              ||    |||    |||| |  |   ||||   |   | |||||||       |||     |         
Sbjct 3259452 CG---GGCGTTCTCGGCAAGTA---CCCT---GCACGAGTTGCAGGGGCGACCCGGCAACCT 3259400

Query     192 TCGCAAAGACGCCGAGGTC--ACCCACGCAACCCACATCGCACGCTTGGTCACTCGG-GGTC 250    
              |||     ||||||||| |     ||| |||||||| ||||||||||||| || ||| ||||        
Sbjct 3259399 TCGAGCCTACGCCGAGGCCGTTGACACACAACCCACCTCGCACGCTTGGTAACCCGGCGGTC 3259338

Query     251 CGTGCTAGCGGGCGGCG 267    
              |||||||||||| ||||        
Sbjct 3259337 CGTGCTAGCGGGTGGCG 3259321

 
                 
                
                 
                      Report:  
                     
                         
                             sequence start
                                   ?  
                                     
Start position of the estimated full-length sequence in genome.
Start index  
                                 :
                             
                             3259284 
                         
                         
                             sequence end
                                   ?  
                                     
End position of the estimated full-length sequence in genome.
Start index  
                                 :
                             
                             3259590 
                         
                         
                             bit score (CM)
                                   ?  
                                     
The score for aligning estimated full-length sequence to CM model
  (computed by RSEARCH -> default,
  infered from Rfam or provided by user) 
                                 :
                             
                             142.05 
                         
                         
                             Homology estimate
                                   ?  
                                     
Quick homology estimate:
  Not homologous: bit score   20 and bit score > 0.5 * query length
  Uncertain otherwise 
                                 :
                             
                             Uncertain 
                         
                     
                 
                
                
                 
                     
                          Estimated full-length sequence:  
                         
                          ? 
                             
Click checkbox to select multiple seuqences.
Fasta header format:
  UID|accession.versionSTRAND start-end 
                         
                     
                     >uid:52|CP035319.1rc 3259284-3259590
UACGGAAGGCCGGAUGGCCAGGAUCGACUCGGAAGAGAAGGCUCGAUUCCCCGAACGGAC
UUCCCAGCACGGACACCAGGCACCCACGCGGAGCUGCAAGCCACUAUAAGGGCAGAAGCG
UUGUGGGCCUGCGUAACUCGGGCGUUCUCGGCAAGUACCCUGCACGAGUUGCAGGGGCGA
CCCGGCAACCUUCGAGCCUACGCCGAGGCCGUUGACACACAACCCACCUCGCACGCUUGG
UAACCCGGCGGUCCGUGCUAGCGGGUGGCGGUAUCGACUCUGAUCGGUAGCGCCACCCGC
UUCGUAU
 
                 
                
             
            
             
                
                     
                         
                         
                             TurboFold 
                             
                              ? 
                                 
Visualisation of predicted secondary structure.
To save the image:
  Right click on the image -> Save Image as. 
                             
                         
                     
                
                     
                         
                         
                             rfam-Rc 
                             
                              ? 
                                 
Visualisation of predicted secondary structure.
To save the image:
  Right click on the image -> Save Image as. 
                             
                         
                     
                
                     
                         
                         
                             rnafold 
                             
                              ? 
                                 
Visualisation of predicted secondary structure.
To save the image:
  Right click on the image -> Save Image as. 
                             
                         
                     
                
             
            

        
             
                 Load Sequence viewer 
             
        
        
         
     
    

    
     
         
            Hit: CP034152.1
         
         
             
                 
                     CP034152.1 Rhodococcus sp. NJ-530 chromosome, complete genome 
                     
                         
 ?  
This is BLAST alignment as read from the input file 
                         
 Score = 88.0 bits (80.6), Expect = 3.32E-11
 Identities = 170/247 (69%), Gaps = 14/247 (6%)
 Strand = Plus/Minus
Query     30 CGGAAGAGAAGGCTAGATCTCCCGACCCAAGCTCCTAGCACGGATACC-GAGCACCCACGCGGA 92    
             ||||||||||||   | |  ||| ||| ||  |||||||||||  ||  | |||||||||||||       
Sbjct 552507 CGGAAGAGAAGGACCGTTTCCCCAACCGAAATTCCTAGCACGGGCACTAGGGCACCCACGCGGA 552444

Query     93 GCACATGCCGCGGAATAGGCAAAAGTGTTGCGGACCTGCGTAGTTTCGAA-AAGCGGACGGCCA 155   
             ||||| ||| |   |  |||| ||| |||| || |||||| || ||||   ||   ||  || |       
Sbjct 552443 GCACAAGCCACTATAAGGGCAGAAGCGTTGTGGGCCTGCG-AGATTCGTTCAATTTGA--GCGA 552383

Query    156 CGACGGCCCTTTGGGTGGGGTTGCAGCCGTAGC----GC-ATCGCAAAGACGCCGAGGTCACCC 214   
              |||  | |   || || ||   |  ||  ||     || ||| |     |||||||| |||||       
Sbjct 552382 GGACCCCGCACAGGTTGAGGGGACGACCTCAGAAAGTGCGATCCCT----CGCCGAGGCCACCC 552323

Query    215 ACGCAACCCACATCGCACGCTTGGTCACTCGGGGTCCGTGCTAGCGGGCGGCGAA 269   
             || ||||||||   ||||||||||| ||  |  |||||||||  |||||||||||       
Sbjct 552322 ACACAACCCACCGAGCACGCTTGGTAACGAGTAGTCCGTGCTGACGGGCGGCGAA 552268

 
                 
                
                 
                      Report:  
                     
                         
                             sequence start
                                   ?  
                                     
Start position of the estimated full-length sequence in genome.
Start index  
                                 :
                             
                             552234 
                         
                         
                             sequence end
                                   ?  
                                     
End position of the estimated full-length sequence in genome.
Start index  
                                 :
                             
                             552535 
                         
                         
                             bit score (CM)
                                   ?  
                                     
The score for aligning estimated full-length sequence to CM model
  (computed by RSEARCH -> default,
  infered from Rfam or provided by user) 
                                 :
                             
                             142.65 
                         
                         
                             Homology estimate
                                   ?  
                                     
Quick homology estimate:
  Not homologous: bit score   20 and bit score > 0.5 * query length
  Uncertain otherwise 
                                 :
                             
                             Uncertain 
                         
                     
                 
                
                
                 
                     
                          Estimated full-length sequence:  
                         
                          ? 
                             
Click checkbox to select multiple seuqences.
Fasta header format:
  UID|accession.versionSTRAND start-end 
                         
                     
                     >uid:53|CP034152.1rc 552234-552535
ACGGAAGAACGGAAGGCCAGGAACGGCUCGGAAGAGAAGGACCGUUUCCCCAACCGAAAU
UCCUAGCACGGGCACUAGGGCACCCACGCGGAGCACAAGCCACUAUAAGGGCAGAAGCGU
UGUGGGCCUGCGAGAUUCGUUCAAUUUGAGCGAGGACCCCGCACAGGUUGAGGGGACGAC
CUCAGAAAGUGCGAUCCCUCGCCGAGGCCACCCACACAACCCACCGAGCACGCUUGGUAA
CGAGUAGUCCGUGCUGACGGGCGGCGAAGUCGAUUUAUCGGCAGCGCCGCCCGUUCGUUA
UG
 
                 
                
             
            
             
                
                     
                         
                         
                             TurboFold 
                             
                              ? 
                                 
Visualisation of predicted secondary structure.
To save the image:
  Right click on the image -> Save Image as. 
                             
                         
                     
                
                     
                         
                         
                             rfam-Rc 
                             
                              ? 
                                 
Visualisation of predicted secondary structure.
To save the image:
  Right click on the image -> Save Image as. 
                             
                         
                     
                
                     
                         
                         
                             rnafold 
                             
                              ? 
                                 
Visualisation of predicted secondary structure.
To save the image:
  Right click on the image -> Save Image as. 
                             
                         
                     
                
             
            

        
             
                 Load Sequence viewer 
             
        
        
         
     
    

    
     
         
            Hit: CP022580.1
         
         
             
                 
                     CP022580.1 Gordonia rubripertincta strain CWB2, complete genome 
                     
                         
 ?  
This is BLAST alignment as read from the input file 
                         
 Score = 88.0 bits (80.6), Expect = 3.32E-11
 Identities = 102/135 (76%), Gaps = 8/135 (6%)
 Strand = Plus/Minus
Query      1 ACGGAAGCTTGGCGAGGCCAAGACCCAGCCGGAAGAGAAGGCTAGATCTCCCGACCCAAGC--- 61    
             ||||||| | || | ||||||| || | |||||||||||||   || | ||| || | |||          
Sbjct 559840 ACGGAAGTTCGGAG-GGCCAAGCCCGATCCGGAAGAGAAGGACCGAGCCCCC-ACTCCAGCACT 559779

Query     62 TCCTAGCACGGATACCGAGCACCCACGCGGAGCACATGCCGCGGAATAGGCAAAAGTGTTGCGG 125   
             ||| |||||||    | ||||||||||||||||||  |||||  || ||||||||| |||| |        
Sbjct 559778 TCCCAGCACGGGCGGCAAGCACCCACGCGGAGCAC--GCCGCATAA-AGGCAAAAGCGTTGTGC 559718

Query    126 ACCTGCG 132   
              ||||||       
Sbjct 559717 GCCTGCG 559711

 
                 
                
                 
                      Report:  
                     
                         
                             sequence start
                                   ?  
                                     
Start position of the estimated full-length sequence in genome.
Start index  
                                 :
                             
                             559539 
                         
                         
                             sequence end
                                   ?  
                                     
End position of the estimated full-length sequence in genome.
Start index  
                                 :
                             
                             559834 
                         
                         
                             bit score (CM)
                                   ?  
                                     
The score for aligning estimated full-length sequence to CM model
  (computed by RSEARCH -> default,
  infered from Rfam or provided by user) 
                                 :
                             
                             4.62 
                         
                         
                             Homology estimate
                                   ?  
                                     
Quick homology estimate:
  Not homologous: bit score   20 and bit score > 0.5 * query length
  Uncertain otherwise 
                                 :
                             
                             Uncertain 
                         
                     
                 
                
                
                 
                     
                          Estimated full-length sequence:  
                         
                          ? 
                             
Click checkbox to select multiple seuqences.
Fasta header format:
  UID|accession.versionSTRAND start-end 
                         
                     
                     >uid:54|CP022580.1rc 559539-559834
ACGGAAGUUCGGAGGGCCAAGCCCGAUCCGGAAGAGAAGGACCGAGCCCCCACUCCAGCA
CUUCCCAGCACGGGCGGCAAGCACCCACGCGGAGCACGCCGCAUAAAGGCAAAAGCGUUG
UGCGCCUGCGAAAUCGCGGCAGGAGAAACGACCGCCACACUAGUUGCGCCACUGCCUUCC
CCCGCCGCAAGGAGCCCGGGGAGAACGGCCCUGAAGAUGGCAGCACGUGGACGGUACGGG
CGACCUGACCCGCGUUCAAUCGCCGAGGCGGUGACCACAACCCACCAAGCACGCUU
 
                 
                
             
            
             
                
                     
                         
                         
                             TurboFold 
                             
                              ? 
                                 
Visualisation of predicted secondary structure.
To save the image:
  Right click on the image -> Save Image as. 
                             
                         
                     
                
                     
                         
                         
                             rfam-Rc 
                             
                              ? 
                                 
Visualisation of predicted secondary structure.
To save the image:
  Right click on the image -> Save Image as. 
                             
                         
                     
                
                     
                         
                         
                             rnafold 
                             
                              ? 
                                 
Visualisation of predicted secondary structure.
To save the image:
  Right click on the image -> Save Image as. 
                             
                         
                     
                
             
            

        
             
                 Load Sequence viewer 
             
        
        
         
     
    

    
     
         
            Hit: CP022580.1
         
         
             
                 
                     CP022580.1 Gordonia rubripertincta strain CWB2, complete genome 
                     
                         
 ?  
This is BLAST alignment as read from the input file 
                         
 Score = 53.0 bits (49.1), Expect = 1.96E-01
 Identities = 46/55 (84%), Gaps = 3/55 (5%)
 Strand = Plus/Minus
Query    218 CAACCCACATCGCACGCTTGGTCACTCGGGGTCCGTGCTA-GCGGGCGGCGAACC 271   
             ||||||||   ||||||||||| |||  | |||||||||| ||||||||| ||||       
Sbjct 559563 CAACCCACCAAGCACGCTTGGTAACT-TGAGTCCGTGCTACGCGGGCGGC-AACC 559511

 
                 
                
                 
                      Report:  
                     
                         
                             sequence start
                                   ?  
                                     
Start position of the estimated full-length sequence in genome.
Start index  
                                 :
                             
                             559448 
                         
                         
                             sequence end
                                   ?  
                                     
End position of the estimated full-length sequence in genome.
Start index  
                                 :
                             
                             559785 
                         
                         
                             bit score (CM)
                                   ?  
                                     
The score for aligning estimated full-length sequence to CM model
  (computed by RSEARCH -> default,
  infered from Rfam or provided by user) 
                                 :
                             
                             74.93 
                         
                         
                             Homology estimate
                                   ?  
                                     
Quick homology estimate:
  Not homologous: bit score   20 and bit score > 0.5 * query length
  Uncertain otherwise 
                                 :
                             
                             Uncertain 
                         
                     
                 
                
                
                 
                     
                          Estimated full-length sequence:  
                         
                          ? 
                             
Click checkbox to select multiple seuqences.
Fasta header format:
  UID|accession.versionSTRAND start-end 
                         
                     
                     >uid:55|CP022580.1rc 559448-559785
GAAGAGAAGGACCGAGCCCCCACUCCAGCACUUCCCAGCACGGGCGGCAAGCACCCACGC
GGAGCACGCCGCAUAAAGGCAAAAGCGUUGUGCGCCUGCGAAAUCGCGGCAGGAGAAACG
ACCGCCACACUAGUUGCGCCACUGCCUUCCCCCGCCGCAAGGAGCCCGGGGAGAACGGCC
CUGAAGAUGGCAGCACGUGGACGGUACGGGCGACCUGACCCGCGUUCAAUCGCCGAGGCG
GUGACCACAACCCACCAAGCACGCUUGGUAACUUGAGUCCGUGCUACGCGGGCGGCAACC
UCGUCGGUGAACGACAGGGAGGCCGCCCGCAGUGUUGU
 
                 
                
             
            
             
                
                     
                         
                         
                             TurboFold 
                             
                              ? 
                                 
Visualisation of predicted secondary structure.
To save the image:
  Right click on the image -> Save Image as. 
                             
                         
                     
                
                     
                         
                         
                             rfam-Rc 
                             
                              ? 
                                 
Visualisation of predicted secondary structure.
To save the image:
  Right click on the image -> Save Image as. 
                             
                         
                     
                
                     
                         
                         
                             rnafold 
                             
                              ? 
                                 
Visualisation of predicted secondary structure.
To save the image:
  Right click on the image -> Save Image as. 
                             
                         
                     
                
             
            

        
             
                 Load Sequence viewer 
             
        
        
         
     
    

    
     
         
            Hit: CP014941.1
         
         
             
                 
                     CP014941.1 Rhodococcus sp. BH4, complete genome 
                     
                         
 ?  
This is BLAST alignment as read from the input file 
                         
 Score = 88.0 bits (80.6), Expect = 3.32E-11
 Identities = 170/247 (69%), Gaps = 14/247 (6%)
 Strand = Plus/Minus
Query     30 CGGAAGAGAAGGCTAGATCTCCCGACCCAAGCTCCTAGCACGGATACC-GAGCACCCACGCGGA 92    
             ||||||||||||   | |  ||| ||| ||  |||||||||||  ||  | |||||||||||||       
Sbjct 553588 CGGAAGAGAAGGACCGTTTCCCCAACCGAAATTCCTAGCACGGGCACTAGGGCACCCACGCGGA 553525

Query     93 GCACATGCCGCGGAATAGGCAAAAGTGTTGCGGACCTGCGTAGTTTCGAA-AAGCGGACGGCCA 155   
             ||||| ||| |   |  |||| ||| |||| || |||||| || ||||   ||   ||  || |       
Sbjct 553524 GCACAAGCCACTATAAGGGCAGAAGCGTTGTGGGCCTGCG-AGATTCGTTCAATTTGA--GCGA 553464

Query    156 CGACGGCCCTTTGGGTGGGGTTGCAGCCGTAGC----GC-ATCGCAAAGACGCCGAGGTCACCC 214   
              |||  | |   || || ||   |  ||  ||     || ||| |     |||||||| |||||       
Sbjct 553463 GGACCCCGCACAGGTTGCGGGGACGACCTCAGAAAGTGCGATCCCT----CGCCGAGGCCACCC 553404

Query    215 ACGCAACCCACATCGCACGCTTGGTCACTCGGGGTCCGTGCTAGCGGGCGGCGAA 269   
             || ||||||||   ||||||||||| ||  |  |||||||||  |||||||||||       
Sbjct 553403 ACACAACCCACCGAGCACGCTTGGTAACGAGTAGTCCGTGCTGACGGGCGGCGAA 553349

 
                 
                
                 
                      Report:  
                     
                         
                             sequence start
                                   ?  
                                     
Start position of the estimated full-length sequence in genome.
Start index  
                                 :
                             
                             553315 
                         
                         
                             sequence end
                                   ?  
                                     
End position of the estimated full-length sequence in genome.
Start index  
                                 :
                             
                             553616 
                         
                         
                             bit score (CM)
                                   ?  
                                     
The score for aligning estimated full-length sequence to CM model
  (computed by RSEARCH -> default,
  infered from Rfam or provided by user) 
                                 :
                             
                             146.09 
                         
                         
                             Homology estimate
                                   ?  
                                     
Quick homology estimate:
  Not homologous: bit score   20 and bit score > 0.5 * query length
  Uncertain otherwise 
                                 :
                             
                             Uncertain 
                         
                     
                 
                
                
                 
                     
                          Estimated full-length sequence:  
                         
                          ? 
                             
Click checkbox to select multiple seuqences.
Fasta header format:
  UID|accession.versionSTRAND start-end 
                         
                     
                     >uid:56|CP014941.1rc 553315-553616
ACGGAAGAACGGAAGGCCAGGAACGGCUCGGAAGAGAAGGACCGUUUCCCCAACCGAAAU
UCCUAGCACGGGCACUAGGGCACCCACGCGGAGCACAAGCCACUAUAAGGGCAGAAGCGU
UGUGGGCCUGCGAGAUUCGUUCAAUUUGAGCGAGGACCCCGCACAGGUUGCGGGGACGAC
CUCAGAAAGUGCGAUCCCUCGCCGAGGCCACCCACACAACCCACCGAGCACGCUUGGUAA
CGAGUAGUCCGUGCUGACGGGCGGCGAAGUCGAUUUAUCGGCAGCGCCGCCCGUUCGUUA
UG
 
                 
                
             
            
             
                
                     
                         
                         
                             TurboFold 
                             
                              ? 
                                 
Visualisation of predicted secondary structure.
To save the image:
  Right click on the image -> Save Image as. 
                             
                         
                     
                
                     
                         
                         
                             rfam-Rc 
                             
                              ? 
                                 
Visualisation of predicted secondary structure.
To save the image:
  Right click on the image -> Save Image as. 
                             
                         
                     
                
                     
                         
                         
                             rnafold 
                             
                              ? 
                                 
Visualisation of predicted secondary structure.
To save the image:
  Right click on the image -> Save Image as. 
                             
                         
                     
                
             
            

        
             
                 Load Sequence viewer 
             
        
        
         
     
    

    
     
         
            Hit: CP015235.1
         
         
             
                 
                     CP015235.1 Rhodococcus fascians D188, complete genome 
                     
                         
 ?  
This is BLAST alignment as read from the input file 
                         
 Score = 88.0 bits (80.6), Expect = 3.32E-11
 Identities = 92/119 (77%), Gaps = 5/119 (4%)
 Strand = Plus/Minus
Query      15 AGGCCAAGACCCAGCCGGAAGAGAAGGCTAG-ATCTCCCGACCCAAGCTCCTAGCACGGATA 75     
              ||||||||| | | ||||||||||||| | |  ||||||||||     ||| |||||||| |        
Sbjct 2503679 AGGCCAAGAACGAACCGGAAGAGAAGGTTCGCTTCTCCCGACCGCGATTCCCAGCACGGACA 2503618

Query      76 CCGAGCACCCACGCGGAGCACATGCCGCGGAATAGGCAAAAGTGTTGCGGACCTGCG 132    
              ||| ||||||||||||||  | |||| |  ||| |||| ||| | || || ||||||        
Sbjct 2503617 CCG-GCACCCACGCGGAG--CTTGCCACCTAAT-GGCAGAAGCGCTGTGGGCCTGCG 2503565

 
                 
                
                 
                      Report:  
                     
                         
                             sequence start
                                   ?  
                                     
Start position of the estimated full-length sequence in genome.
Start index  
                                 :
                             
                             2503393 
                         
                         
                             sequence end
                                   ?  
                                     
End position of the estimated full-length sequence in genome.
Start index  
                                 :
                             
                             2503704 
                         
                         
                             bit score (CM)
                                   ?  
                                     
The score for aligning estimated full-length sequence to CM model
  (computed by RSEARCH -> default,
  infered from Rfam or provided by user) 
                                 :
                             
                             103.77 
                         
                         
                             Homology estimate
                                   ?  
                                     
Quick homology estimate:
  Not homologous: bit score   20 and bit score > 0.5 * query length
  Uncertain otherwise 
                                 :
                             
                             Uncertain 
                         
                     
                 
                
                
                 
                     
                          Estimated full-length sequence:  
                         
                          ? 
                             
Click checkbox to select multiple seuqences.
Fasta header format:
  UID|accession.versionSTRAND start-end 
                         
                     
                     >uid:57|CP015235.1rc 2503393-2503704
UACGGAAGAACGGAAGGCCAAGAACGAACCGGAAGAGAAGGUUCGCUUCUCCCGACCGCG
AUUCCCAGCACGGACACCGGCACCCACGCGGAGCUUGCCACCUAAUGGCAGAAGCGCUGU
GGGCCUGCGAUAUUCGUUGGUGAUCAGCAAGAACCCUGCACCGGUUGCAGGGAUGACCUC
AUCACCGUCGAGUUCUCGCCGAGGCCACGUGGUAGUCCAUCACAGCCCACCUGUGCACGC
UUGGUAACCAGGCGCUCCGUGCUAACGGGCGGCGACGUCGACGCAAGUCGGCAUCGCCGC
CCUUUUACGUGU
 
                 
                
             
            
             
                
                     
                         
                         
                             TurboFold 
                             
                              ? 
                                 
Visualisation of predicted secondary structure.
To save the image:
  Right click on the image -> Save Image as. 
                             
                         
                     
                
                     
                         
                         
                             rfam-Rc 
                             
                              ? 
                                 
Visualisation of predicted secondary structure.
To save the image:
  Right click on the image -> Save Image as. 
                             
                         
                     
                
                     
                         
                         
                             rnafold 
                             
                              ? 
                                 
Visualisation of predicted secondary structure.
To save the image:
  Right click on the image -> Save Image as. 
                             
                         
                     
                
             
            

        
             
                 Load Sequence viewer 
             
        
        
         
     
    

    
     
         
            Hit: CP015220.1
         
         
             
                 
                     CP015220.1 Rhodococcus sp. PBTS2, complete genome 
                     
                         
 ?  
This is BLAST alignment as read from the input file 
                         
 Score = 88.0 bits (80.6), Expect = 3.32E-11
 Identities = 92/119 (77%), Gaps = 5/119 (4%)
 Strand = Plus/Plus
Query     15 AGGCCAAGACCCAGCCGGAAGAGAAGGCTAG-ATCTCCCGACCCAAGCTCCTAGCACGGATACC 77    
             ||||||||| | | ||||||||||||| | |  ||||||||||     ||| |||||||| |||       
Sbjct 974084 AGGCCAAGAACGAACCGGAAGAGAAGGTTCGCTTCTCCCGACCGCGATTCCCAGCACGGACACC 974147

Query     78 GAGCACCCACGCGGAGCACATGCCGCGGAATAGGCAAAAGTGTTGCGGACCTGCG 132   
             | ||||||||||||||  | |||| |  ||| |||| ||| | || || ||||||       
Sbjct 974148 G-GCACCCACGCGGAG--CTTGCCACCTAAT-GGCAGAAGCGCTGTGGGCCTGCG 974198

 
                 
                
                 
                      Report:  
                     
                         
                             sequence start
                                   ?  
                                     
Start position of the estimated full-length sequence in genome.
Start index  
                                 :
                             
                             974070 
                         
                         
                             sequence end
                                   ?  
                                     
End position of the estimated full-length sequence in genome.
Start index  
                                 :
                             
                             974381 
                         
                         
                             bit score (CM)
                                   ?  
                                     
The score for aligning estimated full-length sequence to CM model
  (computed by RSEARCH -> default,
  infered from Rfam or provided by user) 
                                 :
                             
                             103.77 
                         
                         
                             Homology estimate
                                   ?  
                                     
Quick homology estimate:
  Not homologous: bit score   20 and bit score > 0.5 * query length
  Uncertain otherwise 
                                 :
                             
                             Uncertain 
                         
                     
                 
                
                
                 
                     
                          Estimated full-length sequence:  
                         
                          ? 
                             
Click checkbox to select multiple seuqences.
Fasta header format:
  UID|accession.versionSTRAND start-end 
                         
                     
                     >uid:58|CP015220.1fw 974070-974381
UACGGAAGAACGGAAGGCCAAGAACGAACCGGAAGAGAAGGUUCGCUUCUCCCGACCGCG
AUUCCCAGCACGGACACCGGCACCCACGCGGAGCUUGCCACCUAAUGGCAGAAGCGCUGU
GGGCCUGCGAUAUUCGUUGGUGAUCAGCAAGAACCCUGCACCGGUUGCAGGGAUGACCUC
AUCACCGUCGAGUUCUCGCCGAGGCCACGUGGUAGUCCAUCACAGCCCACCUGUGCACGC
UUGGUAACCAGGCGCUCCGUGCUAACGGGCGGCGACGUCGACGCAAGUCGGCAUCGCCGC
CCUUUUACGUGU
 
                 
                
             
            
             
                
                     
                         
                         
                             TurboFold 
                             
                              ? 
                                 
Visualisation of predicted secondary structure.
To save the image:
  Right click on the image -> Save Image as. 
                             
                         
                     
                
                     
                         
                         
                             rfam-Rc 
                             
                              ? 
                                 
Visualisation of predicted secondary structure.
To save the image:
  Right click on the image -> Save Image as. 
                             
                         
                     
                
                     
                         
                         
                             rnafold 
                             
                              ? 
                                 
Visualisation of predicted secondary structure.
To save the image:
  Right click on the image -> Save Image as. 
                             
                         
                     
                
             
            

        
             
                 Load Sequence viewer 
             
        
        
         
     
    

    
     
         
            Hit: CP003761.1
         
         
             
                 
                     CP003761.1 Rhodococcus erythropolis CCM2595, complete genome 
                     
                         
 ?  
This is BLAST alignment as read from the input file 
                         
 Score = 88.0 bits (80.6), Expect = 3.32E-11
 Identities = 170/247 (69%), Gaps = 14/247 (6%)
 Strand = Plus/Minus
Query     30 CGGAAGAGAAGGCTAGATCTCCCGACCCAAGCTCCTAGCACGGATACC-GAGCACCCACGCGGA 92    
             ||||||||||||   | |  ||| ||| ||  |||||||||||  ||  | |||||||||||||       
Sbjct 526261 CGGAAGAGAAGGACCGTTTCCCCAACCGAAATTCCTAGCACGGGCACTAGGGCACCCACGCGGA 526198

Query     93 GCACATGCCGCGGAATAGGCAAAAGTGTTGCGGACCTGCGTAGTTTCGAA-AAGCGGACGGCCA 155   
             ||||| ||| |   |  |||| ||| |||| || |||||| || ||||   ||   ||  || |       
Sbjct 526197 GCACAAGCCACTATAAGGGCAGAAGCGTTGTGGGCCTGCG-AGATTCGTTCAATTTGA--GCGA 526137

Query    156 CGACGGCCCTTTGGGTGGGGTTGCAGCCGTAGC----GC-ATCGCAAAGACGCCGAGGTCACCC 214   
              |||  | |   || || ||   |  ||  ||     || ||| |     |||||||| |||||       
Sbjct 526136 GGACCCCGCACCGGTTGCGGGGACGACCTCAGAAAGTGCGATCCCT----CGCCGAGGCCACCC 526077

Query    215 ACGCAACCCACATCGCACGCTTGGTCACTCGGGGTCCGTGCTAGCGGGCGGCGAA 269   
             || ||||||||   ||||||||||| ||  |  |||||||||  |||||||||||       
Sbjct 526076 ACACAACCCACCGAGCACGCTTGGTAACGAGTAGTCCGTGCTGACGGGCGGCGAA 526022

 
                 
                
                 
                      Report:  
                     
                         
                             sequence start
                                   ?  
                                     
Start position of the estimated full-length sequence in genome.
Start index  
                                 :
                             
                             525987 
                         
                         
                             sequence end
                                   ?  
                                     
End position of the estimated full-length sequence in genome.
Start index  
                                 :
                             
                             526289 
                         
                         
                             bit score (CM)
                                   ?  
                                     
The score for aligning estimated full-length sequence to CM model
  (computed by RSEARCH -> default,
  infered from Rfam or provided by user) 
                                 :
                             
                             136.28 
                         
                         
                             Homology estimate
                                   ?  
                                     
Quick homology estimate:
  Not homologous: bit score   20 and bit score > 0.5 * query length
  Uncertain otherwise 
                                 :
                             
                             Uncertain 
                         
                     
                 
                
                
                 
                     
                          Estimated full-length sequence:  
                         
                          ? 
                             
Click checkbox to select multiple seuqences.
Fasta header format:
  UID|accession.versionSTRAND start-end 
                         
                     
                     >uid:59|CP003761.1rc 525987-526289
UACGGAAGAACGGAAGGCCAGGAACGGCUCGGAAGAGAAGGACCGUUUCCCCAACCGAAA
UUCCUAGCACGGGCACUAGGGCACCCACGCGGAGCACAAGCCACUAUAAGGGCAGAAGCG
UUGUGGGCCUGCGAGAUUCGUUCAAUUUGAGCGAGGACCCCGCACCGGUUGCGGGGACGA
CCUCAGAAAGUGCGAUCCCUCGCCGAGGCCACCCACACAACCCACCGAGCACGCUUGGUA
ACGAGUAGUCCGUGCUGACGGGCGGCGAAGUCGAUUUAUCGGCAGCGCCGCCCGUUCGUU
AUG
 
                 
                
             
            
             
                
                     
                         
                         
                             TurboFold 
                             
                              ? 
                                 
Visualisation of predicted secondary structure.
To save the image:
  Right click on the image -> Save Image as. 
                             
                         
                     
                
                     
                         
                         
                             rfam-Rc 
                             
                              ? 
                                 
Visualisation of predicted secondary structure.
To save the image:
  Right click on the image -> Save Image as. 
                             
                         
                     
                
                     
                         
                         
                             rnafold 
                             
                              ? 
                                 
Visualisation of predicted secondary structure.
To save the image:
  Right click on the image -> Save Image as. 
                             
                         
                     
                
             
            

        
             
                 Load Sequence viewer 
             
        
        
         
     
    

    
     
         
            Hit: AP008957.1
         
         
             
                 
                     AP008957.1 Rhodococcus erythropolis PR4 DNA, complete genome 
                     
                         
 ?  
This is BLAST alignment as read from the input file 
                         
 Score = 88.0 bits (80.6), Expect = 3.32E-11
 Identities = 170/247 (69%), Gaps = 14/247 (6%)
 Strand = Plus/Minus
Query     30 CGGAAGAGAAGGCTAGATCTCCCGACCCAAGCTCCTAGCACGGATACC-GAGCACCCACGCGGA 92    
             ||||||||||||   | |  ||| ||| ||  |||||||||||  ||  | |||||||||||||       
Sbjct 556109 CGGAAGAGAAGGACCGTTTCCCCAACCGAAATTCCTAGCACGGGCACTAGGGCACCCACGCGGA 556046

Query     93 GCACATGCCGCGGAATAGGCAAAAGTGTTGCGGACCTGCGTAGTTTCGAA-AAGCGGACGGCCA 155   
             ||||| ||| |   |  |||| ||| |||| || |||||| || ||||   ||   ||  || |       
Sbjct 556045 GCACAAGCCACTATAAGGGCAGAAGCGTTGTGGGCCTGCG-AGATTCGTTCAATTTGA--GCGA 555985

Query    156 CGACGGCCCTTTGGGTGGGGTTGCAGCCGTAGC----GC-ATCGCAAAGACGCCGAGGTCACCC 214   
              |||  | |   || || ||   |  ||  ||     || ||| |     |||||||| |||||       
Sbjct 555984 GGACCCCGCACCGGTTGCGGGGACGACCTCAGAAAGTGCGATCCCT----CGCCGAGGCCACCC 555925

Query    215 ACGCAACCCACATCGCACGCTTGGTCACTCGGGGTCCGTGCTAGCGGGCGGCGAA 269   
             || ||||||||   ||||||||||| ||  |  |||||||||  |||||||||||       
Sbjct 555924 ACACAACCCACCGAGCACGCTTGGTAACGAGTAGTCCGTGCTGACGGGCGGCGAA 555870

 
                 
                
                 
                      Report:  
                     
                         
                             sequence start
                                   ?  
                                     
Start position of the estimated full-length sequence in genome.
Start index  
                                 :
                             
                             555835 
                         
                         
                             sequence end
                                   ?  
                                     
End position of the estimated full-length sequence in genome.
Start index  
                                 :
                             
                             556137 
                         
                         
                             bit score (CM)
                                   ?  
                                     
The score for aligning estimated full-length sequence to CM model
  (computed by RSEARCH -> default,
  infered from Rfam or provided by user) 
                                 :
                             
                             136.28 
                         
                         
                             Homology estimate
                                   ?  
                                     
Quick homology estimate:
  Not homologous: bit score   20 and bit score > 0.5 * query length
  Uncertain otherwise 
                                 :
                             
                             Uncertain 
                         
                     
                 
                
                
                 
                     
                          Estimated full-length sequence:  
                         
                          ? 
                             
Click checkbox to select multiple seuqences.
Fasta header format:
  UID|accession.versionSTRAND start-end 
                         
                     
                     >uid:60|AP008957.1rc 555835-556137
UACGGAAGAACGGAAGGCCAGGAACGGCUCGGAAGAGAAGGACCGUUUCCCCAACCGAAA
UUCCUAGCACGGGCACUAGGGCACCCACGCGGAGCACAAGCCACUAUAAGGGCAGAAGCG
UUGUGGGCCUGCGAGAUUCGUUCAAUUUGAGCGAGGACCCCGCACCGGUUGCGGGGACGA
CCUCAGAAAGUGCGAUCCCUCGCCGAGGCCACCCACACAACCCACCGAGCACGCUUGGUA
ACGAGUAGUCCGUGCUGACGGGCGGCGAAGUCGAUUUAUCGGCAGCGCCGCCCGUUCGUU
AUG
 
                 
                
             
            
             
                
                     
                         
                         
                             TurboFold 
                             
                              ? 
                                 
Visualisation of predicted secondary structure.
To save the image:
  Right click on the image -> Save Image as. 
                             
                         
                     
                
                     
                         
                         
                             rfam-Rc 
                             
                              ? 
                                 
Visualisation of predicted secondary structure.
To save the image:
  Right click on the image -> Save Image as. 
                             
                         
                     
                
                     
                         
                         
                             rnafold 
                             
                              ? 
                                 
Visualisation of predicted secondary structure.
To save the image:
  Right click on the image -> Save Image as. 
                             
                         
                     
                
             
            

        
             
                 Load Sequence viewer 
             
        
        
         
     
    

    
     
         
            Hit: CP027114.1
         
         
             
                 
                     CP027114.1 Gordonia alkanivorans strain YC-RL2 chromosome, complete genome 
                     
                         
 ?  
This is BLAST alignment as read from the input file 
                         
 Score = 87.0 bits (79.7), Expect = 1.16E-10
 Identities = 101/135 (75%), Gaps = 7/135 (5%)
 Strand = Plus/Plus
Query      1 ACGGAAGCTTGGCGAGGCCAAGACCCAGCCGGAAGAGAAGGCTAGATCTCCCGACCCAAGC--- 61    
             ||||||| | || | ||||||| || | |||||||||||||   ||   ||||  || |||          
Sbjct 534527 ACGGAAGTTCGGAG-GGCCAAGCCCGATCCGGAAGAGAAGGACCGAGTCCCCGCTCCGAGCACT 534589

Query     62 TCCTAGCACGGATACCGAGCACCCACGCGGAGCACATGCCGCGGAATAGGCAAAAGTGTTGCGG 125   
             ||| |||||||    | ||||||||||||||||||  |||||  || ||||||||| |||| |        
Sbjct 534590 TCCCAGCACGGGCGGCAAGCACCCACGCGGAGCAC--GCCGCATAA-AGGCAAAAGCGTTGTGC 534650

Query    126 ACCTGCG 132   
              ||||||       
Sbjct 534651 GCCTGCG 534657

 
                 
                
                 
                      Report:  
                     
                         
                             sequence start
                                   ?  
                                     
Start position of the estimated full-length sequence in genome.
Start index  
                                 :
                             
                             534527 
                         
                         
                             sequence end
                                   ?  
                                     
End position of the estimated full-length sequence in genome.
Start index  
                                 :
                             
                             534825 
                         
                         
                             bit score (CM)
                                   ?  
                                     
The score for aligning estimated full-length sequence to CM model
  (computed by RSEARCH -> default,
  infered from Rfam or provided by user) 
                                 :
                             
                             13.51 
                         
                         
                             Homology estimate
                                   ?  
                                     
Quick homology estimate:
  Not homologous: bit score   20 and bit score > 0.5 * query length
  Uncertain otherwise 
                                 :
                             
                             Uncertain 
                         
                     
                 
                
                
                 
                     
                          Estimated full-length sequence:  
                         
                          ? 
                             
Click checkbox to select multiple seuqences.
Fasta header format:
  UID|accession.versionSTRAND start-end 
                         
                     
                     >uid:61|CP027114.1fw 534527-534825
ACGGAAGUUCGGAGGGCCAAGCCCGAUCCGGAAGAGAAGGACCGAGUCCCCGCUCCGAGC
ACUUCCCAGCACGGGCGGCAAGCACCCACGCGGAGCACGCCGCAUAAAGGCAAAAGCGUU
GUGCGCCUGCGAAAUCGCGGCAGGAGAAACGACCGCCACAUUAGUUGCGCCACUGCCUUC
CCCCCGCCGGCGAGGAGCCCGGGGAGAAUGGCCUUGAAGAUGGCCGCACGUGGACGGUAC
GGGCGACCUGACCCGCGUUCAAUCGCCGAGGCGGUGACCACAACCCACCAAGCACGCUU
 
                 
                
             
            
             
                
                     
                         
                         
                             TurboFold 
                             
                              ? 
                                 
Visualisation of predicted secondary structure.
To save the image:
  Right click on the image -> Save Image as. 
                             
                         
                     
                
                     
                         
                         
                             rfam-Rc 
                             
                              ? 
                                 
Visualisation of predicted secondary structure.
To save the image:
  Right click on the image -> Save Image as. 
                             
                         
                     
                
                     
                         
                         
                             rnafold 
                             
                              ? 
                                 
Visualisation of predicted secondary structure.
To save the image:
  Right click on the image -> Save Image as. 
                             
                         
                     
                
             
            

        
             
                 Load Sequence viewer 
             
        
        
         
     
    

    
     
         
            Hit: CP027114.1
         
         
             
                 
                     CP027114.1 Gordonia alkanivorans strain YC-RL2 chromosome, complete genome 
                     
                         
 ?  
This is BLAST alignment as read from the input file 
                         
 Score = 53.0 bits (49.1), Expect = 1.96E-01
 Identities = 46/55 (84%), Gaps = 3/55 (5%)
 Strand = Plus/Plus
Query    218 CAACCCACATCGCACGCTTGGTCACTCGGGGTCCGTGCTA-GCGGGCGGCGAACC 271   
             ||||||||   ||||||||||| |||  | |||||||||| ||||||||| ||||       
Sbjct 534807 CAACCCACCAAGCACGCTTGGTAACT-TGAGTCCGTGCTACGCGGGCGGC-AACC 534859

 
                 
                
                 
                      Report:  
                     
                         
                             sequence start
                                   ?  
                                     
Start position of the estimated full-length sequence in genome.
Start index  
                                 :
                             
                             534601 
                         
                         
                             sequence end
                                   ?  
                                     
End position of the estimated full-length sequence in genome.
Start index  
                                 :
                             
                             534896 
                         
                         
                             bit score (CM)
                                   ?  
                                     
The score for aligning estimated full-length sequence to CM model
  (computed by RSEARCH -> default,
  infered from Rfam or provided by user) 
                                 :
                             
                             56.11 
                         
                         
                             Homology estimate
                                   ?  
                                     
Quick homology estimate:
  Not homologous: bit score   20 and bit score > 0.5 * query length
  Uncertain otherwise 
                                 :
                             
                             Uncertain 
                         
                     
                 
                
                
                 
                     
                          Estimated full-length sequence:  
                         
                          ? 
                             
Click checkbox to select multiple seuqences.
Fasta header format:
  UID|accession.versionSTRAND start-end 
                         
                     
                     >uid:62|CP027114.1fw 534601-534896
GCGGCAAGCACCCACGCGGAGCACGCCGCAUAAAGGCAAAAGCGUUGUGCGCCUGCGAAA
UCGCGGCAGGAGAAACGACCGCCACAUUAGUUGCGCCACUGCCUUCCCCCCGCCGGCGAG
GAGCCCGGGGAGAAUGGCCUUGAAGAUGGCCGCACGUGGACGGUACGGGCGACCUGACCC
GCGUUCAAUCGCCGAGGCGGUGACCACAACCCACCAAGCACGCUUGGUAACUUGAGUCCG
UGCUACGCGGGCGGCAACCUCGUCGGUAACGACAGGGAGGCCGCCCGCAGUGUUGU
 
                 
                
             
            
             
                
                     
                         
                         
                             TurboFold 
                             
                              ? 
                                 
Visualisation of predicted secondary structure.
To save the image:
  Right click on the image -> Save Image as. 
                             
                         
                     
                
                     
                         
                         
                             rfam-Rc 
                             
                              ? 
                                 
Visualisation of predicted secondary structure.
To save the image:
  Right click on the image -> Save Image as. 
                             
                         
                     
                
                     
                         
                         
                             rnafold 
                             
                              ? 
                                 
Visualisation of predicted secondary structure.
To save the image:
  Right click on the image -> Save Image as. 
                             
                         
                     
                
             
            

        
             
                 Load Sequence viewer 
             
        
        
         
     
    

    
     
         
            Hit: CP000431.1
         
         
             
                 
                     CP000431.1 Rhodococcus jostii RHA1, complete genome 
                     
                         
 ?  
This is BLAST alignment as read from the input file 
                         
 Score = 87.0 bits (79.7), Expect = 1.16E-10
 Identities = 194/288 (67%), Gaps = 6/288 (2%)
 Strand = Plus/Minus
Query      15 AGGCCAAGACCCAGCCGGAAGAGAAGGCTAGATCTCCCGACCCAAGCTCCTAGCACGGATAC 76     
              ||||||||  | | || |||||||||| | ||||||||||||     ||| |||||||  ||        
Sbjct 4570369 AGGCCAAGGTCGACCCAGAAGAGAAGGATCGATCTCCCGACCAGGATTCCCAGCACGGGCAC 4570308

Query      77 CGAGCACCCACGCGGAGCACATGCCGCGGAATAGGCAAAAGTGTTGCGGACCTGCGTAGTTT 138    
                 | ||||||||| |||| | ||| |   |  |||| ||| |||| || |||||| ||           
Sbjct 4570307 TAGGTACCCACGCGAAGCATAAGCCACTATAAGGGCAGAAGCGTTGTGGGCCTGCG-AGACC 4570247

Query     139 CGAAAAGCGGACGGCCACGACGGCCCTTTGGGTGG--GGTTGCAGCCGTAGCGCATCGCAAA 198    
              |||        |||| | |||    |   || ||   ||  | | |||  |    ||| |          
Sbjct 4570246 CGAGTCTTCACCGGCAAGGACCCTGCACAGGTTGCAAGGACG-ACCCGAGGATATTCGGACT 4570186

Query     199 GACGCCGAGGTCACCCACGCAACCCACATC-GCACGCTTGGTCACTCGGGGTCCGTGCTAGC 259    
                |||||||| ||||||| |||||||| |  ||||||||||| ||  |  |||||||||| |        
Sbjct 4570185 CTCGCCGAGGCCACCCACACAACCCACCTTTGCACGCTTGGTAACCGGTTGTCCGTGCTAAC 4570124

Query     260 GGGCGGCGAACCGGACA-TTGTCCGGGACGCCGCCCGATT 298    
              ||||||||||   ||||   ||| |   ||||||||| ||        
Sbjct 4570123 GGGCGGCGAAGTCGACACCAGTCGGCAGCGCCGCCCGTTT 4570084

 
                 
                
                 
                      Report:  
                     
                         
                             sequence start
                                   ?  
                                     
Start position of the estimated full-length sequence in genome.
Start index  
                                 :
                             
                             4570079 
                         
                         
                             sequence end
                                   ?  
                                     
End position of the estimated full-length sequence in genome.
Start index  
                                 :
                             
                             4570382 
                         
                         
                             bit score (CM)
                                   ?  
                                     
The score for aligning estimated full-length sequence to CM model
  (computed by RSEARCH -> default,
  infered from Rfam or provided by user) 
                                 :
                             
                             144.64 
                         
                         
                             Homology estimate
                                   ?  
                                     
Quick homology estimate:
  Not homologous: bit score   20 and bit score > 0.5 * query length
  Uncertain otherwise 
                                 :
                             
                             Uncertain 
                         
                     
                 
                
                
                 
                     
                          Estimated full-length sequence:  
                         
                          ? 
                             
Click checkbox to select multiple seuqences.
Fasta header format:
  UID|accession.versionSTRAND start-end 
                         
                     
                     >uid:63|CP000431.1rc 4570079-4570382
ACGGAAGAACGGAAGGCCAAGGUCGACCCAGAAGAGAAGGAUCGAUCUCCCGACCAGGAU
UCCCAGCACGGGCACUAGGUACCCACGCGAAGCAUAAGCCACUAUAAGGGCAGAAGCGUU
GUGGGCCUGCGAGACCCGAGUCUUCACCGGCAAGGACCCUGCACAGGUUGCAAGGACGAC
CCGAGGAUAUUCGGACUCUCGCCGAGGCCACCCACACAACCCACCUUUGCACGCUUGGUA
ACCGGUUGUCCGUGCUAACGGGCGGCGAAGUCGACACCAGUCGGCAGCGCCGCCCGUUUC
ACAU
 
                 
                
             
            
             
                
                     
                         
                         
                             TurboFold 
                             
                              ? 
                                 
Visualisation of predicted secondary structure.
To save the image:
  Right click on the image -> Save Image as. 
                             
                         
                     
                
                     
                         
                         
                             rfam-Rc 
                             
                              ? 
                                 
Visualisation of predicted secondary structure.
To save the image:
  Right click on the image -> Save Image as. 
                             
                         
                     
                
                     
                         
                         
                             rnafold 
                             
                              ? 
                                 
Visualisation of predicted secondary structure.
To save the image:
  Right click on the image -> Save Image as. 
                             
                         
                     
                
             
            

        
             
                 Load Sequence viewer 
             
        
        
         
     
    

    
     
         
            Hit: CP000431.1
         
         
             
                 
                     CP000431.1 Rhodococcus jostii RHA1, complete genome 
                     
                         
 ?  
This is BLAST alignment as read from the input file 
                         
 Score = 52.0 bits (48.2), Expect = 1.96E-01
 Identities = 76/103 (74%), Gaps = 11/103 (11%)
 Strand = Plus/Plus
Query     199 GACGCCGAGGTCAC---CCACGCAACCCA--CATCGCACGCTTGGTCACTCGGGGTCCGTGC 255    
              |||||||||| |||   |||| |  | ||  ||| ||| ||||||| || ||  || |||||        
Sbjct 7240192 GACGCCGAGGCCACATTCCACCCCGCGCAACCAT-GCA-GCTTGGTAACCCGACGTTCGTGC 7240251

Query     256 TAGCGGGCGGCGAACCGGACATTGTCCGGGACGCCGCCCGA 296    
              | ||||||||||  ||   |||   | ||||||||||||||        
Sbjct 7240252 TCGCGGGCGGCGCCCCCT-CAT---CAGGGACGCCGCCCGA 7240288

 
                 
                
                 
                      Report:  
                     
                         
                             sequence start
                                   ?  
                                     
Start position of the estimated full-length sequence in genome.
Start index  
                                 :
                             
                             7239987 
                         
                         
                             sequence end
                                   ?  
                                     
End position of the estimated full-length sequence in genome.
Start index  
                                 :
                             
                             7240296 
                         
                         
                             bit score (CM)
                                   ?  
                                     
The score for aligning estimated full-length sequence to CM model
  (computed by RSEARCH -> default,
  infered from Rfam or provided by user) 
                                 :
                             
                             53.47 
                         
                         
                             Homology estimate
                                   ?  
                                     
Quick homology estimate:
  Not homologous: bit score   20 and bit score > 0.5 * query length
  Uncertain otherwise 
                                 :
                             
                             Uncertain 
                         
                     
                 
                
                
                 
                     
                          Estimated full-length sequence:  
                         
                          ? 
                             
Click checkbox to select multiple seuqences.
Fasta header format:
  UID|accession.versionSTRAND start-end 
                         
                     
                     >uid:64|CP000431.1fw 7239987-7240296
GCGGGCUCUGGCUCACGUUCGUUGCCUUAUCCGGAUAUGCGGGGUAGACCUGAGGUACGG
AUCCACGGCAGACCAAGGUCGGACCGGAAGAGAAGAUUCCACCUCUCGGUCGUGGCGCCC
AGCACGAGUUGUAGGCACCCACGCACAAGUACAGCCCACUAACUGGGCUCAGCGCGGUGG
GCUUGCGUCAGGCGGGGGGAUGUCUGACGCCGAGGCCACAUUCCACCCCGCGCAACCAUG
CAGCUUGGUAACCCGACGUUCGUGCUCGCGGGCGGCGCCCCCUCAUCAGGGACGCCGCCC
GACGUGUGUG
 
                 
                
             
            
             
                
                     
                         
                         
                             TurboFold 
                             
                              ? 
                                 
Visualisation of predicted secondary structure.
To save the image:
  Right click on the image -> Save Image as. 
                             
                         
                     
                
                     
                         
                         
                             rfam-Rc 
                             
                              ? 
                                 
Visualisation of predicted secondary structure.
To save the image:
  Right click on the image -> Save Image as. 
                             
                         
                     
                
                     
                         
                         
                             rnafold 
                             
                              ? 
                                 
Visualisation of predicted secondary structure.
To save the image:
  Right click on the image -> Save Image as. 
                             
                         
                     
                
             
            

        
             
                 Load Sequence viewer 
             
        
        
         
     
    

    
     
         
            Hit: CP032568.1
         
         
             
                 
                     CP032568.1 Nocardia sp. CFHS0054 chromosome 
                     
                         
 ?  
This is BLAST alignment as read from the input file 
                         
 Score = 86.0 bits (78.8), Expect = 1.16E-10
 Identities = 92/121 (76%), Gaps = 4/121 (3%)
 Strand = Plus/Minus
Query     15 AGGCCAAGACCCAGCCGGAAGAGAAGGCTAGATCTCCCGACCCAAGCT-CCTAGCACGGATACC 77    
             ||||||||| |    ||||||||||||   || |||||  ||||  || || |||||||| |||       
Sbjct 821583 AGGCCAAGATCGGATCGGAAGAGAAGGTCCGACCTCCCATCCCACCCTTCCCAGCACGGACACC 821520

Query     78 GAGCACCCACGCGGAGCACATGCCGCGGAATAGGCAAAAGT-GTTGCGGACCTGCGT 133   
               |||||||||||||||||  ||||||  |  ||||  ||| |||| || |||||||       
Sbjct 821519 AGGCACCCACGCGGAGCAC--GCCGCGACAAGGGCACGAGTAGTTGTGGGCCTGCGT 821465

 
                 
                
                 
                      Report:  
                     
                         
                             sequence start
                                   ?  
                                     
Start position of the estimated full-length sequence in genome.
Start index  
                                 :
                             
                             821294 
                         
                         
                             sequence end
                                   ?  
                                     
End position of the estimated full-length sequence in genome.
Start index  
                                 :
                             
                             821600 
                         
                         
                             bit score (CM)
                                   ?  
                                     
The score for aligning estimated full-length sequence to CM model
  (computed by RSEARCH -> default,
  infered from Rfam or provided by user) 
                                 :
                             
                             121.85 
                         
                         
                             Homology estimate
                                   ?  
                                     
Quick homology estimate:
  Not homologous: bit score   20 and bit score > 0.5 * query length
  Uncertain otherwise 
                                 :
                             
                             Uncertain 
                         
                     
                 
                
                
                 
                     
                          Estimated full-length sequence:  
                         
                          ? 
                             
Click checkbox to select multiple seuqences.
Fasta header format:
  UID|accession.versionSTRAND start-end 
                         
                     
                     >uid:65|CP032568.1rc 821294-821600
CACGGAAGGACGGAAGGCCAAGAUCGGAUCGGAAGAGAAGGUCCGACCUCCCAUCCCACC
CUUCCCAGCACGGACACCAGGCACCCACGCGGAGCACGCCGCGACAAGGGCACGAGUAGU
UGUGGGCCUGCGUUAUCCGGGAUAUCGACGGCGAGGGCCUCGCAUCCAUGCGGGGAUGAA
CCACCGGAUCCUGGAGAGCUCGCCGAGGCCGAAGAAACACAACCCGAGAAGCACGCUUGG
UAACCGGGUAGUCCGUGCUAGCGGGCGGUGAGGUCGCAAAACGCGACAACGCCGCCCGCU
CUGAUGU
 
                 
                
             
            
             
                
                     
                         
                         
                             TurboFold 
                             
                              ? 
                                 
Visualisation of predicted secondary structure.
To save the image:
  Right click on the image -> Save Image as. 
                             
                         
                     
                
                     
                         
                         
                             rfam-Rc 
                             
                              ? 
                                 
Visualisation of predicted secondary structure.
To save the image:
  Right click on the image -> Save Image as. 
                             
                         
                     
                
                     
                         
                         
                             rnafold 
                             
                              ? 
                                 
Visualisation of predicted secondary structure.
To save the image:
  Right click on the image -> Save Image as. 
                             
                         
                     
                
             
            

        
             
                 Load Sequence viewer 
             
        
        
         
     
    

    
     
         
            Hit: CP032568.1
         
         
             
                 
                     CP032568.1 Nocardia sp. CFHS0054 chromosome 
                     
                         
 ?  
This is BLAST alignment as read from the input file 
                         
 Score = 52.0 bits (48.2), Expect = 1.96E-01
 Identities = 37/42 (88%), Gaps = 3/42 (7%)
 Strand = Plus/Minus
Query    229 GCACGCTTGGTCACTCGGG--GTCCGTGCTAGCGGGCGGCGA 268   
             ||||||||||| || ||||  |||||||||||||||||| ||       
Sbjct 821367 GCACGCTTGGTAAC-CGGGTAGTCCGTGCTAGCGGGCGGTGA 821327

 
                 
                
                 
                      Report:  
                     
                         
                             sequence start
                                   ?  
                                     
Start position of the estimated full-length sequence in genome.
Start index  
                                 :
                             
                             821289 
                         
                         
                             sequence end
                                   ?  
                                     
End position of the estimated full-length sequence in genome.
Start index  
                                 :
                             
                             821595 
                         
                         
                             bit score (CM)
                                   ?  
                                     
The score for aligning estimated full-length sequence to CM model
  (computed by RSEARCH -> default,
  infered from Rfam or provided by user) 
                                 :
                             
                             121.85 
                         
                         
                             Homology estimate
                                   ?  
                                     
Quick homology estimate:
  Not homologous: bit score   20 and bit score > 0.5 * query length
  Uncertain otherwise 
                                 :
                             
                             Uncertain 
                         
                     
                 
                
                
                 
                     
                          Estimated full-length sequence:  
                         
                          ? 
                             
Click checkbox to select multiple seuqences.
Fasta header format:
  UID|accession.versionSTRAND start-end 
                         
                     
                     >uid:66|CP032568.1rc 821289-821595
CACGGAAGGACGGAAGGCCAAGAUCGGAUCGGAAGAGAAGGUCCGACCUCCCAUCCCACC
CUUCCCAGCACGGACACCAGGCACCCACGCGGAGCACGCCGCGACAAGGGCACGAGUAGU
UGUGGGCCUGCGUUAUCCGGGAUAUCGACGGCGAGGGCCUCGCAUCCAUGCGGGGAUGAA
CCACCGGAUCCUGGAGAGCUCGCCGAGGCCGAAGAAACACAACCCGAGAAGCACGCUUGG
UAACCGGGUAGUCCGUGCUAGCGGGCGGUGAGGUCGCAAAACGCGACAACGCCGCCCGCU
CUGAUGU
 
                 
                
             
            
             
                
                     
                         
                         
                             TurboFold 
                             
                              ? 
                                 
Visualisation of predicted secondary structure.
To save the image:
  Right click on the image -> Save Image as. 
                             
                         
                     
                
                     
                         
                         
                             rfam-Rc 
                             
                              ? 
                                 
Visualisation of predicted secondary structure.
To save the image:
  Right click on the image -> Save Image as. 
                             
                         
                     
                
                     
                         
                         
                             rnafold 
                             
                              ? 
                                 
Visualisation of predicted secondary structure.
To save the image:
  Right click on the image -> Save Image as. 
                             
                         
                     
                
             
            

        
             
                 Load Sequence viewer 
             
        
        
         
     
    

    
     
         
            Hit: AP017900.1
         
         
             
                 
                     AP017900.1 Nocardia seriolae DNA, complete genome, strain: UTF1 
                     
                         
 ?  
This is BLAST alignment as read from the input file 
                         
 Score = 86.0 bits (78.8), Expect = 1.16E-10
 Identities = 182/263 (69%), Gaps = 15/263 (6%)
 Strand = Plus/Minus
Query     15 AGGCCAAGACCCAGCCGGAAGAGAAGGCTAGATCTCCCGACCCAAGCT-CCTAGCACGGATACC 77    
             ||||||||| |    |||||||||||  | || |||||  ||||  || || |||||||| |||       
Sbjct 442973 AGGCCAAGATCGGATCGGAAGAGAAGATTCGACCTCCCATCCCACCCTTCCCAGCACGGACACC 442910

Query     78 GAGCACCCACGCGGAGCACATGCCGCGGAATAGGCA-AAAGTGTTGCGGACCTGCGTAGTTTCG 140   
               |||||||||||||||||  ||||||  |  ||||  ||  |||| || |||||||  |   |       
Sbjct 442909 AGGCACCCACGCGGAGCAC--GCCGCGACAAGGGCACGAATGGTTGTGGGCCTGCGTTATCCGG 442848

Query    141 AAAAGCGGACGGCCACGACGGC-CCTTTGGGTGGGGTTGCAGCCGTAGCGCATC--GCAAAG-A 200   
              | | | |||||| | | |  | | |    | |||| || | |   | || |||  | | ||         
Sbjct 442847 GATATC-GACGGCGAGGGCCTCGCATAATCGCGGGGATGAACC---ACCGGATCCTGGAGAGCT 442788

Query    201 CGCCGAGGTC--ACCCACGCAACCCACATCGCACGCTTGGTCACTCGG-GGTCCGTGCTAGCGG 261   
             |||||||| |  |  ||| ||||||     ||||||||||| ||  || |||||||||||||||       
Sbjct 442787 CGCCGAGGCCGAAAACACACAACCCGAGAAGCACGCTTGGTAACCAGGTGGTCCGTGCTAGCGG 442724

Query    262 GCGGCGA 268   
             |||| ||       
Sbjct 442723 GCGGTGA 442717

 
                 
                
                 
                      Report:  
                     
                         
                             sequence start
                                   ?  
                                     
Start position of the estimated full-length sequence in genome.
Start index  
                                 :
                             
                             442681 
                         
                         
                             sequence end
                                   ?  
                                     
End position of the estimated full-length sequence in genome.
Start index  
                                 :
                             
                             442988 
                         
                         
                             bit score (CM)
                                   ?  
                                     
The score for aligning estimated full-length sequence to CM model
  (computed by RSEARCH -> default,
  infered from Rfam or provided by user) 
                                 :
                             
                             120.47 
                         
                         
                             Homology estimate
                                   ?  
                                     
Quick homology estimate:
  Not homologous: bit score   20 and bit score > 0.5 * query length
  Uncertain otherwise 
                                 :
                             
                             Uncertain 
                         
                     
                 
                
                
                 
                     
                          Estimated full-length sequence:  
                         
                          ? 
                             
Click checkbox to select multiple seuqences.
Fasta header format:
  UID|accession.versionSTRAND start-end 
                         
                     
                     >uid:67|AP017900.1rc 442681-442988
CACGGAAGGACGGAAGGCCAAGAUCGGAUCGGAAGAGAAGAUUCGACCUCCCAUCCCACC
CUUCCCAGCACGGACACCAGGCACCCACGCGGAGCACGCCGCGACAAGGGCACGAAUGGU
UGUGGGCCUGCGUUAUCCGGGAUAUCGACGGCGAGGGCCUCGCAUAAUCGCGGGGAUGAA
CCACCGGAUCCUGGAGAGCUCGCCGAGGCCGAAAACACACAACCCGAGAAGCACGCUUGG
UAACCAGGUGGUCCGUGCUAGCGGGCGGUGAGGUCGCAAUUCAGCGACAACGCCGCCCGC
UCUGAUGU
 
                 
                
             
            
             
                
                     
                         
                         
                             TurboFold 
                             
                              ? 
                                 
Visualisation of predicted secondary structure.
To save the image:
  Right click on the image -> Save Image as. 
                             
                         
                     
                
                     
                         
                         
                             rfam-Rc 
                             
                              ? 
                                 
Visualisation of predicted secondary structure.
To save the image:
  Right click on the image -> Save Image as. 
                             
                         
                     
                
                     
                         
                         
                             rnafold 
                             
                              ? 
                                 
Visualisation of predicted secondary structure.
To save the image:
  Right click on the image -> Save Image as. 
                             
                         
                     
                
             
            

        
             
                 Load Sequence viewer 
             
        
        
         
     
    

    
     
         
            Hit: CP017839.1
         
         
             
                 
                     CP017839.1 Nocardia seriolae strain EM150506, complete genome 
                     
                         
 ?  
This is BLAST alignment as read from the input file 
                         
 Score = 86.0 bits (78.8), Expect = 1.16E-10
 Identities = 182/263 (69%), Gaps = 15/263 (6%)
 Strand = Plus/Plus
Query     15 AGGCCAAGACCCAGCCGGAAGAGAAGGCTAGATCTCCCGACCCAAGCT-CCTAGCACGGATACC 77    
             ||||||||| |    |||||||||||  | || |||||  ||||  || || |||||||| |||       
Sbjct 371263 AGGCCAAGATCGGATCGGAAGAGAAGATTCGACCTCCCATCCCACCCTTCCCAGCACGGACACC 371326

Query     78 GAGCACCCACGCGGAGCACATGCCGCGGAATAGGCA-AAAGTGTTGCGGACCTGCGTAGTTTCG 140   
               |||||||||||||||||  ||||||  |  ||||  ||  |||| || |||||||  |   |       
Sbjct 371327 AGGCACCCACGCGGAGCAC--GCCGCGACAAGGGCACGAATGGTTGTGGGCCTGCGTTATCCGG 371388

Query    141 AAAAGCGGACGGCCACGACGGC-CCTTTGGGTGGGGTTGCAGCCGTAGCGCATC--GCAAAG-A 200   
              | | | |||||| | | |  | | |    | |||| || | |   | || |||  | | ||         
Sbjct 371389 GATATC-GACGGCGAGGGCCTCGCATAATCGCGGGGATGAACC---ACCGGATCCTGGAGAGCT 371448

Query    201 CGCCGAGGTC--ACCCACGCAACCCACATCGCACGCTTGGTCACTCGG-GGTCCGTGCTAGCGG 261   
             |||||||| |  |  ||| ||||||     ||||||||||| ||  || |||||||||||||||       
Sbjct 371449 CGCCGAGGCCGAAAACACACAACCCGAGAAGCACGCTTGGTAACCAGGTGGTCCGTGCTAGCGG 371512

Query    262 GCGGCGA 268   
             |||| ||       
Sbjct 371513 GCGGTGA 371519

 
                 
                
                 
                      Report:  
                     
                         
                             sequence start
                                   ?  
                                     
Start position of the estimated full-length sequence in genome.
Start index  
                                 :
                             
                             371249 
                         
                         
                             sequence end
                                   ?  
                                     
End position of the estimated full-length sequence in genome.
Start index  
                                 :
                             
                             371556 
                         
                         
                             bit score (CM)
                                   ?  
                                     
The score for aligning estimated full-length sequence to CM model
  (computed by RSEARCH -> default,
  infered from Rfam or provided by user) 
                                 :
                             
                             120.47 
                         
                         
                             Homology estimate
                                   ?  
                                     
Quick homology estimate:
  Not homologous: bit score   20 and bit score > 0.5 * query length
  Uncertain otherwise 
                                 :
                             
                             Uncertain 
                         
                     
                 
                
                
                 
                     
                          Estimated full-length sequence:  
                         
                          ? 
                             
Click checkbox to select multiple seuqences.
Fasta header format:
  UID|accession.versionSTRAND start-end 
                         
                     
                     >uid:68|CP017839.1fw 371249-371556
CACGGAAGGACGGAAGGCCAAGAUCGGAUCGGAAGAGAAGAUUCGACCUCCCAUCCCACC
CUUCCCAGCACGGACACCAGGCACCCACGCGGAGCACGCCGCGACAAGGGCACGAAUGGU
UGUGGGCCUGCGUUAUCCGGGAUAUCGACGGCGAGGGCCUCGCAUAAUCGCGGGGAUGAA
CCACCGGAUCCUGGAGAGCUCGCCGAGGCCGAAAACACACAACCCGAGAAGCACGCUUGG
UAACCAGGUGGUCCGUGCUAGCGGGCGGUGAGGUCGCAAUUCAGCGACAACGCCGCCCGC
UCUGAUGU
 
                 
                
             
            
             
                
                     
                         
                         
                             TurboFold 
                             
                              ? 
                                 
Visualisation of predicted secondary structure.
To save the image:
  Right click on the image -> Save Image as. 
                             
                         
                     
                
                     
                         
                         
                             rfam-Rc 
                             
                              ? 
                                 
Visualisation of predicted secondary structure.
To save the image:
  Right click on the image -> Save Image as. 
                             
                         
                     
                
                     
                         
                         
                             rnafold 
                             
                              ? 
                                 
Visualisation of predicted secondary structure.
To save the image:
  Right click on the image -> Save Image as. 
                             
                         
                     
                
             
            

        
             
                 Load Sequence viewer 
             
        
        
         
     
    

    
     
         
            Hit: CP039253.1
         
         
             
                 
                     CP039253.1 Rhodococcus sp. PAMC28707 chromosome, complete genome 
                     
                         
 ?  
This is BLAST alignment as read from the input file 
                         
 Score = 85.0 bits (77.9), Expect = 4.05E-10
 Identities = 94/126 (75%), Gaps = 3/126 (2%)
 Strand = Plus/Minus
Query      15 AGGCCAAGACCCAGCCGGAAGAGAAGGCTAGATCTCCCGACCCAAGCTCCTAGCACGGATAC 76     
              ||||||||| | | ||||||||||||| |   |||||| |||     ||| |||||||| ||        
Sbjct 2627284 AGGCCAAGATCGAACCGGAAGAGAAGGTTCCCTCTCCCAACCGCGATTCCCAGCACGGACAC 2627223

Query      77 CGAGCACCCACGCGGAGCACATGCCGCGGAATAGGCAAAAGTGTTGCGGACCTGCGTAGTTT 138    
              |  |||||||||| |||| |  ||||| ||   |||||||| | || ||  ||||| || ||        
Sbjct 2627222 CAGGCACCCACGCTGAGCTC--GCCGCTGACATGGCAAAAGCGCTGTGGGTCTGCG-AGATT 2627164

Query     139 CG 140    
              ||        
Sbjct 2627163 CG 2627162

 
                 
                
                 
                      Report:  
                     
                         
                             sequence start
                                   ?  
                                     
Start position of the estimated full-length sequence in genome.
Start index  
                                 :
                             
                             2626999 
                         
                         
                             sequence end
                                   ?  
                                     
End position of the estimated full-length sequence in genome.
Start index  
                                 :
                             
                             2627316 
                         
                         
                             bit score (CM)
                                   ?  
                                     
The score for aligning estimated full-length sequence to CM model
  (computed by RSEARCH -> default,
  infered from Rfam or provided by user) 
                                 :
                             
                             96.52 
                         
                         
                             Homology estimate
                                   ?  
                                     
Quick homology estimate:
  Not homologous: bit score   20 and bit score > 0.5 * query length
  Uncertain otherwise 
                                 :
                             
                             Uncertain 
                         
                     
                 
                
                
                 
                     
                          Estimated full-length sequence:  
                         
                          ? 
                             
Click checkbox to select multiple seuqences.
Fasta header format:
  UID|accession.versionSTRAND start-end 
                         
                     
                     >uid:69|CP039253.1rc 2626999-2627316
ACGGAAGAACGGAAGGCCAAGAUCGAACCGGAAGAGAAGGUUCCCUCUCCCAACCGCGAU
UCCCAGCACGGACACCAGGCACCCACGCUGAGCUCGCCGCUGACAUGGCAAAAGCGCUGU
GGGUCUGCGAGAUUCGGGAAGUAGCAGCAAGAACCCUGCACCGGUUGCAGGGAUGACCUC
CUACCUCACCGGGCCAUCGCCGAGGCCAUGCGAUCCUAGCGAUCGUGACAGCCCACCAAU
GCACGCUUGGUUACCCGGCAGUCCGUGCUAACGGGCGGCGAAGUCGACUCAGGUCGGCGG
CGCCGCCCUUUUAAUGCG
 
                 
                
             
            
             
                
                     
                         
                         
                             TurboFold 
                             
                              ? 
                                 
Visualisation of predicted secondary structure.
To save the image:
  Right click on the image -> Save Image as. 
                             
                         
                     
                
                     
                         
                         
                             rfam-Rc 
                             
                              ? 
                                 
Visualisation of predicted secondary structure.
To save the image:
  Right click on the image -> Save Image as. 
                             
                         
                     
                
                     
                         
                         
                             rnafold 
                             
                              ? 
                                 
Visualisation of predicted secondary structure.
To save the image:
  Right click on the image -> Save Image as. 
                             
                         
                     
                
             
            

        
             
                 Load Sequence viewer 
             
        
        
         
     
    

    
     
         
            Hit: CP039253.1
         
         
             
                 
                     CP039253.1 Rhodococcus sp. PAMC28707 chromosome, complete genome 
                     
                         
 ?  
This is BLAST alignment as read from the input file 
                         
 Score = 55.0 bits (50.9), Expect = 5.63E-02
 Identities = 37/42 (88%), Gaps = 1/42 (2%)
 Strand = Plus/Minus
Query     229 GCACGCTTGGTCACTCGGG-GTCCGTGCTAGCGGGCGGCGAA 269    
              ||||||||||| || |||  |||||||||| |||||||||||        
Sbjct 2627057 GCACGCTTGGTTACCCGGCAGTCCGTGCTAACGGGCGGCGAA 2627016

 
                 
                
                 
                      Report:  
                     
                         
                             sequence start
                                   ?  
                                     
Start position of the estimated full-length sequence in genome.
Start index  
                                 :
                             
                             2626969 
                         
                         
                             sequence end
                                   ?  
                                     
End position of the estimated full-length sequence in genome.
Start index  
                                 :
                             
                             2627286 
                         
                         
                             bit score (CM)
                                   ?  
                                     
The score for aligning estimated full-length sequence to CM model
  (computed by RSEARCH -> default,
  infered from Rfam or provided by user) 
                                 :
                             
                             96.52 
                         
                         
                             Homology estimate
                                   ?  
                                     
Quick homology estimate:
  Not homologous: bit score   20 and bit score > 0.5 * query length
  Uncertain otherwise 
                                 :
                             
                             Uncertain 
                         
                     
                 
                
                
                 
                     
                          Estimated full-length sequence:  
                         
                          ? 
                             
Click checkbox to select multiple seuqences.
Fasta header format:
  UID|accession.versionSTRAND start-end 
                         
                     
                     >uid:70|CP039253.1rc 2626969-2627286
ACGGAAGAACGGAAGGCCAAGAUCGAACCGGAAGAGAAGGUUCCCUCUCCCAACCGCGAU
UCCCAGCACGGACACCAGGCACCCACGCUGAGCUCGCCGCUGACAUGGCAAAAGCGCUGU
GGGUCUGCGAGAUUCGGGAAGUAGCAGCAAGAACCCUGCACCGGUUGCAGGGAUGACCUC
CUACCUCACCGGGCCAUCGCCGAGGCCAUGCGAUCCUAGCGAUCGUGACAGCCCACCAAU
GCACGCUUGGUUACCCGGCAGUCCGUGCUAACGGGCGGCGAAGUCGACUCAGGUCGGCGG
CGCCGCCCUUUUAAUGCG
 
                 
                
             
            
             
                
                     
                         
                         
                             TurboFold 
                             
                              ? 
                                 
Visualisation of predicted secondary structure.
To save the image:
  Right click on the image -> Save Image as. 
                             
                         
                     
                
                     
                         
                         
                             rfam-Rc 
                             
                              ? 
                                 
Visualisation of predicted secondary structure.
To save the image:
  Right click on the image -> Save Image as. 
                             
                         
                     
                
                     
                         
                         
                             rnafold 
                             
                              ? 
                                 
Visualisation of predicted secondary structure.
To save the image:
  Right click on the image -> Save Image as. 
                             
                         
                     
                
             
            

        
             
                 Load Sequence viewer 
             
        
        
         
     
    

    
     
         
            Hit: CP039254.1
         
         
             
                 
                     CP039254.1 Rhodococcus sp. PAMC28705 chromosome, complete genome 
                     
                         
 ?  
This is BLAST alignment as read from the input file 
                         
 Score = 85.0 bits (77.9), Expect = 4.05E-10
 Identities = 94/126 (75%), Gaps = 3/126 (2%)
 Strand = Plus/Plus
Query     15 AGGCCAAGACCCAGCCGGAAGAGAAGGCTAGATCTCCCGACCCAAGCTCCTAGCACGGATACCG 78    
             ||||||||| | | ||||||||||||| |   |||||| |||     ||| |||||||| |||        
Sbjct 429873 AGGCCAAGATCGAACCGGAAGAGAAGGTTCCCTCTCCCAACCGCGATTCCCAGCACGGACACCA 429936

Query     79 AGCACCCACGCGGAGCACATGCCGCGGAATAGGCAAAAGTGTTGCGGACCTGCGTAGTTTCG 140   
              |||||||||| |||| |  ||||| ||   |||||||| | || ||  ||||| || ||||       
Sbjct 429937 GGCACCCACGCTGAGCTC--GCCGCTGACATGGCAAAAGCGCTGTGGGTCTGCG-AGATTCG 429995

 
                 
                
                 
                      Report:  
                     
                         
                             sequence start
                                   ?  
                                     
Start position of the estimated full-length sequence in genome.
Start index  
                                 :
                             
                             429860 
                         
                         
                             sequence end
                                   ?  
                                     
End position of the estimated full-length sequence in genome.
Start index  
                                 :
                             
                             430177 
                         
                         
                             bit score (CM)
                                   ?  
                                     
The score for aligning estimated full-length sequence to CM model
  (computed by RSEARCH -> default,
  infered from Rfam or provided by user) 
                                 :
                             
                             96.52 
                         
                         
                             Homology estimate
                                   ?  
                                     
Quick homology estimate:
  Not homologous: bit score   20 and bit score > 0.5 * query length
  Uncertain otherwise 
                                 :
                             
                             Uncertain 
                         
                     
                 
                
                
                 
                     
                          Estimated full-length sequence:  
                         
                          ? 
                             
Click checkbox to select multiple seuqences.
Fasta header format:
  UID|accession.versionSTRAND start-end 
                         
                     
                     >uid:71|CP039254.1fw 429860-430177
ACGGAAGAACGGAAGGCCAAGAUCGAACCGGAAGAGAAGGUUCCCUCUCCCAACCGCGAU
UCCCAGCACGGACACCAGGCACCCACGCUGAGCUCGCCGCUGACAUGGCAAAAGCGCUGU
GGGUCUGCGAGAUUCGGGAAGUAGCAGCAAGAACCCUGCACCGGUUGCAGGGAUGACCUC
CUACCUCACCGGGCCAUCGCCGAGGCCAUGCGAUCCUAGCGAUCGUGACAGCCCACCAAU
GCACGCUUGGUUACCCGGCAGUCCGUGCUAACGGGCGGCGAAGUCGACUCAGGUCGGCGG
CGCCGCCCUUUUAAUGCG
 
                 
                
             
            
             
                
                     
                         
                         
                             TurboFold 
                             
                              ? 
                                 
Visualisation of predicted secondary structure.
To save the image:
  Right click on the image -> Save Image as. 
                             
                         
                     
                
                     
                         
                         
                             rfam-Rc 
                             
                              ? 
                                 
Visualisation of predicted secondary structure.
To save the image:
  Right click on the image -> Save Image as. 
                             
                         
                     
                
                     
                         
                         
                             rnafold 
                             
                              ? 
                                 
Visualisation of predicted secondary structure.
To save the image:
  Right click on the image -> Save Image as. 
                             
                         
                     
                
             
            

        
             
                 Load Sequence viewer 
             
        
        
         
     
    

    
     
         
            Hit: CP039254.1
         
         
             
                 
                     CP039254.1 Rhodococcus sp. PAMC28705 chromosome, complete genome 
                     
                         
 ?  
This is BLAST alignment as read from the input file 
                         
 Score = 55.0 bits (50.9), Expect = 5.63E-02
 Identities = 45/54 (83%), Gaps = 2/54 (4%)
 Strand = Plus/Plus
Query    218 CAACCCAC-ATCGCACGCTTGGTCACTCGGG-GTCCGTGCTAGCGGGCGGCGAA 269   
             || ||||| |  ||||||||||| || |||  |||||||||| |||||||||||       
Sbjct 430088 CAGCCCACCAATGCACGCTTGGTTACCCGGCAGTCCGTGCTAACGGGCGGCGAA 430141

 
                 
                
                 
                      Report:  
                     
                         
                             sequence start
                                   ?  
                                     
Start position of the estimated full-length sequence in genome.
Start index  
                                 :
                             
                             429860 
                         
                         
                             sequence end
                                   ?  
                                     
End position of the estimated full-length sequence in genome.
Start index  
                                 :
                             
                             430177 
                         
                         
                             bit score (CM)
                                   ?  
                                     
The score for aligning estimated full-length sequence to CM model
  (computed by RSEARCH -> default,
  infered from Rfam or provided by user) 
                                 :
                             
                             96.52 
                         
                         
                             Homology estimate
                                   ?  
                                     
Quick homology estimate:
  Not homologous: bit score   20 and bit score > 0.5 * query length
  Uncertain otherwise 
                                 :
                             
                             Uncertain 
                         
                     
                 
                
                
                 
                     
                          Estimated full-length sequence:  
                         
                          ? 
                             
Click checkbox to select multiple seuqences.
Fasta header format:
  UID|accession.versionSTRAND start-end 
                         
                     
                     >uid:72|CP039254.1fw 429860-430177
ACGGAAGAACGGAAGGCCAAGAUCGAACCGGAAGAGAAGGUUCCCUCUCCCAACCGCGAU
UCCCAGCACGGACACCAGGCACCCACGCUGAGCUCGCCGCUGACAUGGCAAAAGCGCUGU
GGGUCUGCGAGAUUCGGGAAGUAGCAGCAAGAACCCUGCACCGGUUGCAGGGAUGACCUC
CUACCUCACCGGGCCAUCGCCGAGGCCAUGCGAUCCUAGCGAUCGUGACAGCCCACCAAU
GCACGCUUGGUUACCCGGCAGUCCGUGCUAACGGGCGGCGAAGUCGACUCAGGUCGGCGG
CGCCGCCCUUUUAAUGCG
 
                 
                
             
            
             
                
                     
                         
                         
                             TurboFold 
                             
                              ? 
                                 
Visualisation of predicted secondary structure.
To save the image:
  Right click on the image -> Save Image as. 
                             
                         
                     
                
                     
                         
                         
                             rfam-Rc 
                             
                              ? 
                                 
Visualisation of predicted secondary structure.
To save the image:
  Right click on the image -> Save Image as. 
                             
                         
                     
                
                     
                         
                         
                             rnafold 
                             
                              ? 
                                 
Visualisation of predicted secondary structure.
To save the image:
  Right click on the image -> Save Image as. 
                             
                         
                     
                
             
            

        
             
                 Load Sequence viewer 
             
        
        
         
     
    

    
     
         
            Hit: CP010797.1
         
         
             
                 
                     CP010797.1 Rhodococcus sp. B7740, complete genome 
                     
                         
 ?  
This is BLAST alignment as read from the input file 
                         
 Score = 85.0 bits (77.9), Expect = 4.05E-10
 Identities = 90/118 (76%), Gaps = 4/118 (3%)
 Strand = Plus/Minus
Query      15 AGGCCAAGACCCAGCCGGAAGAGAAGGCTAGATCTCCCGACCCAAGCTCCTAGCACGGATAC 76     
              ||||||||| | |  |||||||||||  | | ||||||||||     ||| |||||||| ||        
Sbjct 4393968 AGGCCAAGAACGAATCGGAAGAGAAGATTCGCTCTCCCGACCGGGATTCCCAGCACGGACAC 4393907

Query      77 CGAGCACCCACGCGGAGCACATGCCGCGGAATAGGCAAAAGTGTTGCGGACCTGCG 132    
              || ||||||||||||||  | |||| |  ||| |||| ||| | || || ||||||        
Sbjct 4393906 CG-GCACCCACGCGGAG--CTTGCCACCTAAT-GGCAGAAGCGCTGTGGGCCTGCG 4393855

 
                 
                
                 
                      Report:  
                     
                         
                             sequence start
                                   ?  
                                     
Start position of the estimated full-length sequence in genome.
Start index  
                                 :
                             
                             4393684 
                         
                         
                             sequence end
                                   ?  
                                     
End position of the estimated full-length sequence in genome.
Start index  
                                 :
                             
                             4393993 
                         
                         
                             bit score (CM)
                                   ?  
                                     
The score for aligning estimated full-length sequence to CM model
  (computed by RSEARCH -> default,
  infered from Rfam or provided by user) 
                                 :
                             
                             117.03 
                         
                         
                             Homology estimate
                                   ?  
                                     
Quick homology estimate:
  Not homologous: bit score   20 and bit score > 0.5 * query length
  Uncertain otherwise 
                                 :
                             
                             Uncertain 
                         
                     
                 
                
                
                 
                     
                          Estimated full-length sequence:  
                         
                          ? 
                             
Click checkbox to select multiple seuqences.
Fasta header format:
  UID|accession.versionSTRAND start-end 
                         
                     
                     >uid:73|CP010797.1rc 4393684-4393993
ACGGAAGAACGGAAGGCCAAGAACGAAUCGGAAGAGAAGAUUCGCUCUCCCGACCGGGAU
UCCCAGCACGGACACCGGCACCCACGCGGAGCUUGCCACCUAAUGGCAGAAGCGCUGUGG
GCCUGCGACAUUCGUCAGUGAUCAGCAAGAACCCUGCACCGGUUGCAGGGAUGACCUCAU
CACCGUCGAAUUCUCGCCGAGGCCACGUGGUAGUCCAUCACAGCCCACCUGUGCACGCUU
GGUAACCAGGCGCUCCGUGCUAACGGGCGGCGACGUCGACGCAAGUCGGCAUCGCCGCCC
UUUUAUAUGC
 
                 
                
             
            
             
                
                     
                         
                         
                             TurboFold 
                             
                              ? 
                                 
Visualisation of predicted secondary structure.
To save the image:
  Right click on the image -> Save Image as. 
                             
                         
                     
                
                     
                         
                         
                             rfam-Rc 
                             
                              ? 
                                 
Visualisation of predicted secondary structure.
To save the image:
  Right click on the image -> Save Image as. 
                             
                         
                     
                
                     
                         
                         
                             rnafold 
                             
                              ? 
                                 
Visualisation of predicted secondary structure.
To save the image:
  Right click on the image -> Save Image as. 
                             
                         
                     
                
             
            

        
             
                 Load Sequence viewer 
             
        
        
         
     
    

    
     
         
            Hit: CP023720.1
         
         
             
                 
                     CP023720.1 Rhodococcus sp. H-CA8f chromosome, complete genome 
                     
                         
 ?  
This is BLAST alignment as read from the input file 
                         
 Score = 83.0 bits (76.1), Expect = 1.41E-09
 Identities = 169/247 (68%), Gaps = 14/247 (6%)
 Strand = Plus/Plus
Query      30 CGGAAGAGAAGGCTAGATCTCCCGACCCAAGCTCCTAGCACGGATACC-GAGCACCCACGCG 90     
              ||||||||||||   | |  ||| ||| ||  |||||||||||  ||  | |||||||||||        
Sbjct 5680311 CGGAAGAGAAGGACCGTTTCCCCAACCGAAATTCCTAGCACGGGCACTAGGGCACCCACGCG 5680372

Query      91 GAGCACATGCCGCGGAATAGGCAAAAGTGTTGCGGACCTGCGTAGTTTCG-AAAAGCGGACG 151    
              ||||||| ||| |   |  |||| ||| |||| || |||||| || ||||   ||   ||          
Sbjct 5680373 GAGCACAAGCCACTATAAGGGCAGAAGCGTTGTGGGCCTGCG-AGATTCGTTCAATTTGA-- 5680431

Query     152 GCCACGACGGCCCTTTGGGTGGGGTTGCAGCC----GTAGCGC-ATCGCAAAGACGCCGAGG 208    
              || | |||  | |   || || ||   |  ||      || || ||| |     ||||||||        
Sbjct 5680432 GCGAGGACCCCGCACCGGTTGCGGGGACGACCTCAGAAAGTGCGATCCC----TCGCCGAGG 5680489

Query     209 TCACCCACGCAACCCACATCGCACGCTTGGTCACTCGGGGTCCGTGCTAGCGGGCGGCGAA 269    
               ||||||| ||||||||   ||||||||||| ||  |  ||||||| |  |||||||||||        
Sbjct 5680490 CCACCCACACAACCCACCGAGCACGCTTGGTAACGAGTAGTCCGTGGTGACGGGCGGCGAA 5680550

 
                 
                
                 
                      Report:  
                     
                         
                             sequence start
                                   ?  
                                     
Start position of the estimated full-length sequence in genome.
Start index  
                                 :
                             
                             5680283 
                         
                         
                             sequence end
                                   ?  
                                     
End position of the estimated full-length sequence in genome.
Start index  
                                 :
                             
                             5680584 
                         
                         
                             bit score (CM)
                                   ?  
                                     
The score for aligning estimated full-length sequence to CM model
  (computed by RSEARCH -> default,
  infered from Rfam or provided by user) 
                                 :
                             
                             136.73 
                         
                         
                             Homology estimate
                                   ?  
                                     
Quick homology estimate:
  Not homologous: bit score   20 and bit score > 0.5 * query length
  Uncertain otherwise 
                                 :
                             
                             Uncertain 
                         
                     
                 
                
                
                 
                     
                          Estimated full-length sequence:  
                         
                          ? 
                             
Click checkbox to select multiple seuqences.
Fasta header format:
  UID|accession.versionSTRAND start-end 
                         
                     
                     >uid:74|CP023720.1fw 5680283-5680584
ACGGAAGAACGGAAGGCCAGGAACGGCUCGGAAGAGAAGGACCGUUUCCCCAACCGAAAU
UCCUAGCACGGGCACUAGGGCACCCACGCGGAGCACAAGCCACUAUAAGGGCAGAAGCGU
UGUGGGCCUGCGAGAUUCGUUCAAUUUGAGCGAGGACCCCGCACCGGUUGCGGGGACGAC
CUCAGAAAGUGCGAUCCCUCGCCGAGGCCACCCACACAACCCACCGAGCACGCUUGGUAA
CGAGUAGUCCGUGGUGACGGGCGGCGAAGUCGAUUUAUCGGCAGCGCCGCCCGUUCGUUA
UG
 
                 
                
             
            
             
                
                     
                         
                         
                             TurboFold 
                             
                              ? 
                                 
Visualisation of predicted secondary structure.
To save the image:
  Right click on the image -> Save Image as. 
                             
                         
                     
                
                     
                         
                         
                             rfam-Rc 
                             
                              ? 
                                 
Visualisation of predicted secondary structure.
To save the image:
  Right click on the image -> Save Image as. 
                             
                         
                     
                
                     
                         
                         
                             rnafold 
                             
                              ? 
                                 
Visualisation of predicted secondary structure.
To save the image:
  Right click on the image -> Save Image as. 
                             
                         
                     
                
             
            

        
             
                 Load Sequence viewer 
             
        
        
         
     
    

    
     
         
            Hit: CP011295.1
         
         
             
                 
                     CP011295.1 Rhodococcus erythropolis strain BG43, complete genome 
                     
                         
 ?  
This is BLAST alignment as read from the input file 
                         
 Score = 83.0 bits (76.1), Expect = 1.41E-09
 Identities = 169/247 (68%), Gaps = 14/247 (6%)
 Strand = Plus/Minus
Query     30 CGGAAGAGAAGGCTAGATCTCCCGACCCAAGCTCCTAGCACGGATACC-GAGCACCCACGCGGA 92    
             ||||||||||||   | |  ||| ||  ||  |||||||||||  ||  | |||||||||||||       
Sbjct 566651 CGGAAGAGAAGGACCGTTTCCCCAACTGAAATTCCTAGCACGGGCACTAGGGCACCCACGCGGA 566588

Query     93 GCACATGCCGCGGAATAGGCAAAAGTGTTGCGGACCTGCGTAGTTTCGAA-AAGCGGACGGCCA 155   
             ||||| ||| |   |  |||| ||| |||| || |||||| || ||||   ||   ||  || |       
Sbjct 566587 GCACAAGCCACTATAAGGGCAGAAGCGTTGTGGGCCTGCG-AGATTCGTTCAATTTGA--GCGA 566527

Query    156 CGACGGCCCTTTGGGTGGGGTTGCAGCCGTAGC----GC-ATCGCAAAGACGCCGAGGTCACCC 214   
              |||  | |   || || ||   |  ||  ||     || ||| |     |||||||| |||||       
Sbjct 566526 GGACCCCGCACCGGTTGCGGGGACGACCTCAGAAAGTGCGATCCCT----CGCCGAGGCCACCC 566467

Query    215 ACGCAACCCACATCGCACGCTTGGTCACTCGGGGTCCGTGCTAGCGGGCGGCGAA 269   
             || ||||||||   ||||||||||| ||  |  |||||||||  |||||||||||       
Sbjct 566466 ACACAACCCACCGAGCACGCTTGGTAACGAGTAGTCCGTGCTGACGGGCGGCGAA 566412

 
                 
                
                 
                      Report:  
                     
                         
                             sequence start
                                   ?  
                                     
Start position of the estimated full-length sequence in genome.
Start index  
                                 :
                             
                             566378 
                         
                         
                             sequence end
                                   ?  
                                     
End position of the estimated full-length sequence in genome.
Start index  
                                 :
                             
                             566679 
                         
                         
                             bit score (CM)
                                   ?  
                                     
The score for aligning estimated full-length sequence to CM model
  (computed by RSEARCH -> default,
  infered from Rfam or provided by user) 
                                 :
                             
                             142.23 
                         
                         
                             Homology estimate
                                   ?  
                                     
Quick homology estimate:
  Not homologous: bit score   20 and bit score > 0.5 * query length
  Uncertain otherwise 
                                 :
                             
                             Uncertain 
                         
                     
                 
                
                
                 
                     
                          Estimated full-length sequence:  
                         
                          ? 
                             
Click checkbox to select multiple seuqences.
Fasta header format:
  UID|accession.versionSTRAND start-end 
                         
                     
                     >uid:75|CP011295.1rc 566378-566679
ACGGAAGAACGGAAGGCCAGGAACGGCUCGGAAGAGAAGGACCGUUUCCCCAACUGAAAU
UCCUAGCACGGGCACUAGGGCACCCACGCGGAGCACAAGCCACUAUAAGGGCAGAAGCGU
UGUGGGCCUGCGAGAUUCGUUCAAUUUGAGCGAGGACCCCGCACCGGUUGCGGGGACGAC
CUCAGAAAGUGCGAUCCCUCGCCGAGGCCACCCACACAACCCACCGAGCACGCUUGGUAA
CGAGUAGUCCGUGCUGACGGGCGGCGAAGUCGAUUUAUCGGCAGCGCCGCCCGUUCGUUA
UG
 
                 
                
             
            
             
                
                     
                         
                         
                             TurboFold 
                             
                              ? 
                                 
Visualisation of predicted secondary structure.
To save the image:
  Right click on the image -> Save Image as. 
                             
                         
                     
                
                     
                         
                         
                             rfam-Rc 
                             
                              ? 
                                 
Visualisation of predicted secondary structure.
To save the image:
  Right click on the image -> Save Image as. 
                             
                         
                     
                
                     
                         
                         
                             rnafold 
                             
                              ? 
                                 
Visualisation of predicted secondary structure.
To save the image:
  Right click on the image -> Save Image as. 
                             
                         
                     
                
             
            

        
             
                 Load Sequence viewer 
             
        
        
         
     
    

    
     
         
            Hit: CP029710.1
         
         
             
                 
                     CP029710.1 Nocardia sp. CS682 chromosome, complete genome 
                     
                         
 ?  
This is BLAST alignment as read from the input file 
                         
 Score = 82.0 bits (75.2), Expect = 1.41E-09
 Identities = 97/132 (73%), Gaps = 3/132 (2%)
 Strand = Plus/Minus
Query       1 ACGGAAGCTTGGCGAGGCCAAGACCCAGCCGGAAGAGAAGGCTAGATCTCCCGACCCAAGCT 62     
              ||||||| | ||  |||||||| |    ||||||||||||| |   ||||||| ||     |        
Sbjct 5064716 ACGGAAGGTCGGA-AGGCCAAGGCAGGACCGGAAGAGAAGGTTCCGTCTCCCGTCCATCCTT 5064656

Query      63 CCTAGCACGGATACCGAGCACCCACGCGGAGCACATGCCGCGGAATAGGCAAAAGTGTTGCG 124    
              || |||||||  |||  ||||||||||||||| |  ||||||  |  |||| ||| |||| |        
Sbjct 5064655 CCCAGCACGGGCACCAGGCACCCACGCGGAGCGC--GCCGCGACAAGGGCAGAAGCGTTGTG 5064596

Query     125 GACCTGCG 132    
              | ||||||        
Sbjct 5064595 GGCCTGCG 5064588

 
                 
                
                 
                      Report:  
                     
                         
                             sequence start
                                   ?  
                                     
Start position of the estimated full-length sequence in genome.
Start index  
                                 :
                             
                             5064416 
                         
                         
                             sequence end
                                   ?  
                                     
End position of the estimated full-length sequence in genome.
Start index  
                                 :
                             
                             5064716 
                         
                         
                             bit score (CM)
                                   ?  
                                     
The score for aligning estimated full-length sequence to CM model
  (computed by RSEARCH -> default,
  infered from Rfam or provided by user) 
                                 :
                             
                             124.5 
                         
                         
                             Homology estimate
                                   ?  
                                     
Quick homology estimate:
  Not homologous: bit score   20 and bit score > 0.5 * query length
  Uncertain otherwise 
                                 :
                             
                             Uncertain 
                         
                     
                 
                
                
                 
                     
                          Estimated full-length sequence:  
                         
                          ? 
                             
Click checkbox to select multiple seuqences.
Fasta header format:
  UID|accession.versionSTRAND start-end 
                         
                     
                     >uid:76|CP029710.1rc 5064416-5064716
ACGGAAGGUCGGAAGGCCAAGGCAGGACCGGAAGAGAAGGUUCCGUCUCCCGUCCAUCCU
UCCCAGCACGGGCACCAGGCACCCACGCGGAGCGCGCCGCGACAAGGGCAGAAGCGUUGU
GGGCCUGCGAAAUCCGGGUCUUCGUCGGCGAUGGCCUCGCACAGGUUGCGGGGAUGAACC
GCCGGAGGUCGGACUCUCGCCGAGGCCGUAGAACACAACCCACCCAGCACGCUUGGUAAC
CGGGUAGUCCGUGCUACGCGGGCGGUGAAGUCGGCAACGACGCGCCGCCCGCUUUGACGU
G
 
                 
                
             
            
             
                
                     
                         
                         
                             TurboFold 
                             
                              ? 
                                 
Visualisation of predicted secondary structure.
To save the image:
  Right click on the image -> Save Image as. 
                             
                         
                     
                
                     
                         
                         
                             rfam-Rc 
                             
                              ? 
                                 
Visualisation of predicted secondary structure.
To save the image:
  Right click on the image -> Save Image as. 
                             
                         
                     
                
                     
                         
                         
                             rnafold 
                             
                              ? 
                                 
Visualisation of predicted secondary structure.
To save the image:
  Right click on the image -> Save Image as. 
                             
                         
                     
                
             
            

        
             
                 Load Sequence viewer 
             
        
        
         
     
    

    
     
         
            Hit: CP029710.1
         
         
             
                 
                     CP029710.1 Nocardia sp. CS682 chromosome, complete genome 
                     
                         
 ?  
This is BLAST alignment as read from the input file 
                         
 Score = 54.0 bits (50.0), Expect = 5.63E-02
 Identities = 46/55 (84%), Gaps = 4/55 (7%)
 Strand = Plus/Minus
Query     218 CAACCCACATCGCACGCTTGGTCACTCGGG--GTCCGTGCTA-GCGGGCGGCGAA 269    
              ||||||||   ||||||||||| || ||||  |||||||||| |||||||| |||        
Sbjct 5064501 CAACCCACCCAGCACGCTTGGTAAC-CGGGTAGTCCGTGCTACGCGGGCGGTGAA 5064448

 
                 
                
                 
                      Report:  
                     
                         
                             sequence start
                                   ?  
                                     
Start position of the estimated full-length sequence in genome.
Start index  
                                 :
                             
                             5064415 
                         
                         
                             sequence end
                                   ?  
                                     
End position of the estimated full-length sequence in genome.
Start index  
                                 :
                             
                             5064714 
                         
                         
                             bit score (CM)
                                   ?  
                                     
The score for aligning estimated full-length sequence to CM model
  (computed by RSEARCH -> default,
  infered from Rfam or provided by user) 
                                 :
                             
                             130.11 
                         
                         
                             Homology estimate
                                   ?  
                                     
Quick homology estimate:
  Not homologous: bit score   20 and bit score > 0.5 * query length
  Uncertain otherwise 
                                 :
                             
                             Uncertain 
                         
                     
                 
                
                
                 
                     
                          Estimated full-length sequence:  
                         
                          ? 
                             
Click checkbox to select multiple seuqences.
Fasta header format:
  UID|accession.versionSTRAND start-end 
                         
                     
                     >uid:77|CP029710.1rc 5064415-5064714
ACGGAAGGUCGGAAGGCCAAGGCAGGACCGGAAGAGAAGGUUCCGUCUCCCGUCCAUCCU
UCCCAGCACGGGCACCAGGCACCCACGCGGAGCGCGCCGCGACAAGGGCAGAAGCGUUGU
GGGCCUGCGAAAUCCGGGUCUUCGUCGGCGAUGGCCUCGCACAGGUUGCGGGGAUGAACC
GCCGGAGGUCGGACUCUCGCCGAGGCCGUAGAACACAACCCACCCAGCACGCUUGGUAAC
CGGGUAGUCCGUGCUACGCGGGCGGUGAAGUCGGCAACGACGCGCCGCCCGCUUUGACGU
 
                 
                
             
            
             
                
                     
                         
                         
                             TurboFold 
                             
                              ? 
                                 
Visualisation of predicted secondary structure.
To save the image:
  Right click on the image -> Save Image as. 
                             
                         
                     
                
                     
                         
                         
                             rfam-Rc 
                             
                              ?
[truncated: 327,411 more chars]
