# Supplementary material for: rboAnalyzer: A Software to Improve Characterization of Non-coding RNAs From Sequence Database Search Output
Source: Front Genet. 2020 Jul 28;11:675. doi: 10.3389/fgene.2020.00675 (PMC7401326; doi:10.3389/fgene.2020.00675)
Supplement: Supplementary file 3 [file Data_Sheet_2.ZIP › TR/TR_rboAnalyzer_output_alternative.html]

rboAnalyzer


## Please wait while sequence viewer is loading...

# rboAnalyzer report

```
BLAST output file:   /media/big-user/data-raid/clanek_rbo_analyzer/examples/tel_rna_sel/R7P5G0KU015-Alignment.xml
Query sequence file: /media/big-user/data-raid/clanek_rbo_analyzer/examples/tel_rna_sel/telo_rna_query.fasta

RFAM model with best score to a query sequence

?

```
Infered from query sequence by cmscan program.
```

Family name: Telomerase-vert
E-value:     7.6e-26
```

### Hit: AF221915.1

AF221915.1 Rhizoprionodon porosus telomerase RNA gene, sequence

```
?

```
This is BLAST alignment as read from the input file
```

Score = 1096.0 bits (989.5), Expect = 0.00E+00
 Identities = 548/548 (100%), Gaps = 0/548 (0%)
 Strand = Plus/Plus
Query   1 TCACATTGCTGCGGGGTGGAGGCGTTTTTAACTAACCCTAATGCAGAGTAAGTGGCTGGGCCTTCCTCAC 70 
          ||||||||||||||||||||||||||||||||||||||||||||||||||||||||||||||||||||||    
Sbjct 120 TCACATTGCTGCGGGGTGGAGGCGTTTTTAACTAACCCTAATGCAGAGTAAGTGGCTGGGCCTTCCTCAC 189

Query  71 ACTGCCTCCCGCTGCGTGAAACCTCGCTGTTTTCTTGGCTAACTTTCAGCGAGGTGAGGCAAGGCGGCAA 140
          ||||||||||||||||||||||||||||||||||||||||||||||||||||||||||||||||||||||    
Sbjct 190 ACTGCCTCCCGCTGCGTGAAACCTCGCTGTTTTCTTGGCTAACTTTCAGCGAGGTGAGGCAAGGCGGCAA 259

Query 141 AAAAAAGTTGGGAGCAGCAACGGCAAGCAAAAAAAAAGTTCCAGCCGAGGCCTCCTCACCACGCGGCCTG 210
          ||||||||||||||||||||||||||||||||||||||||||||||||||||||||||||||||||||||    
Sbjct 260 AAAAAAGTTGGGAGCAGCAACGGCAAGCAAAAAAAAAGTTCCAGCCGAGGCCTCCTCACCACGCGGCCTG 329

Query 211 GGTCTGAGGGTAACATGGCCCTGCGGGAGAGAGCCAACACGGGGGGGCCCCGGTCCGGCTAACAGGGCAG 280
          ||||||||||||||||||||||||||||||||||||||||||||||||||||||||||||||||||||||    
Sbjct 330 GGTCTGAGGGTAACATGGCCCTGCGGGAGAGAGCCAACACGGGGGGGCCCCGGTCCGGCTAACAGGGCAG 399

Query 281 GAGGTCGGCCCCAATTGCCTCAGGCCCAGCCACTCCGGGCCGCGATCAGCCCGGGCTTTCCCTACTTTGG 350
          ||||||||||||||||||||||||||||||||||||||||||||||||||||||||||||||||||||||    
Sbjct 400 GAGGTCGGCCCCAATTGCCTCAGGCCCAGCCACTCCGGGCCGCGATCAGCCCGGGCTTTCCCTACTTTGG 469

Query 351 GGCCCAATGCTGTCGCGAAGAGTTCGGCTCTGTCAGCCCGGCTGGGTCCGGGTGGGGGCCGGCACAGAGG 420
          ||||||||||||||||||||||||||||||||||||||||||||||||||||||||||||||||||||||    
Sbjct 470 GGCCCAATGCTGTCGCGAAGAGTTCGGCTCTGTCAGCCCGGCTGGGTCCGGGTGGGGGCCGGCACAGAGG 539

Query 421 GACACACCGGGCCGGGGCGCCGAGGATAGCTACCGCCCGCAGGGATAGTAAATATCCGTGACCAGTGGCC 490
          ||||||||||||||||||||||||||||||||||||||||||||||||||||||||||||||||||||||    
Sbjct 540 GACACACCGGGCCGGGGCGCCGAGGATAGCTACCGCCCGCAGGGATAGTAAATATCCGTGACCAGTGGCC 609

Query 491 TCGGTGGCTGCGTCTCCCGGAGCAATGGGACCCGCAAGGCTGCCCTGGTCATACACGC 548
          ||||||||||||||||||||||||||||||||||||||||||||||||||||||||||    
Sbjct 610 TCGGTGGCTGCGTCTCCCGGAGCAATGGGACCCGCAAGGCTGCCCTGGTCATACACGC 667
```

Report:

|  |  |
| --- | --- |
| sequence start ?  ``` Start position of the estimated full-length sequence in genome. Start index < end index. ``` : | 120 |
| sequence end ?  ``` End position of the estimated full-length sequence in genome. Start index < end index. ``` : | 667 |
| bit score (CM) ?  ``` The score for aligning estimated full-length sequence to CM model   (computed by RSEARCH -> default,   infered from Rfam or provided by user) ``` : | 722.67 |
| Homology estimate ?  ``` Quick homology estimate:   Not homologous: bit score < 0   Homologous: bit score > 20 and bit score > 0.5 * query length   Uncertain otherwise ``` : | Homologous |

Estimated full-length sequence:


?

```
Click checkbox to select multiple seuqences.
Fasta header format:
  UID|accession.versionSTRAND start-end
```

>uid:0|AF221915.1fw 120-667
UCACAUUGCUGCGGGGUGGAGGCGUUUUUAACUAACCCUAAUGCAGAGUAAGUGGCUGGG
CCUUCCUCACACUGCCUCCCGCUGCGUGAAACCUCGCUGUUUUCUUGGCUAACUUUCAGC
GAGGUGAGGCAAGGCGGCAAAAAAAAGUUGGGAGCAGCAACGGCAAGCAAAAAAAAAGUU
CCAGCCGAGGCCUCCUCACCACGCGGCCUGGGUCUGAGGGUAACAUGGCCCUGCGGGAGA
GAGCCAACACGGGGGGGCCCCGGUCCGGCUAACAGGGCAGGAGGUCGGCCCCAAUUGCCU
CAGGCCCAGCCACUCCGGGCCGCGAUCAGCCCGGGCUUUCCCUACUUUGGGGCCCAAUGC
UGUCGCGAAGAGUUCGGCUCUGUCAGCCCGGCUGGGUCCGGGUGGGGGCCGGCACAGAGG
GACACACCGGGCCGGGGCGCCGAGGAUAGCUACCGCCCGCAGGGAUAGUAAAUAUCCGUG
ACCAGUGGCCUCGGUGGCUGCGUCUCCCGGAGCAAUGGGACCCGCAAGGCUGCCCUGGUC
AUACACGC

C-A-r-Rc


?

```
Visualisation of predicted secondary structure.
To save the image:
  Right click on the image -> Save Image as.
```


centroid


?

```
Visualisation of predicted secondary structure.
To save the image:
  Right click on the image -> Save Image as.
```


fq-sub


?

```
Visualisation of predicted secondary structure.
To save the image:
  Right click on the image -> Save Image as.
```

Load Sequence viewer

uid:0|AF221915.1: Sequence cannot be extended sufficiently. Missing nt downstream in the genome.

### Hit: AF221933.1

AF221933.1 Mustelus canis telomerase RNA gene, sequence

```
?

```
This is BLAST alignment as read from the input file
```

Score = 909.0 bits (820.9), Expect = 0.00E+00
 Identities = 521/558 (93%), Gaps = 13/558 (2%)
 Strand = Plus/Plus
Query   1 TCACATTGCTGCGGGGTGGAGGCGTTTTTAACTAACCCTAATGCAGAGTAAGTGGCTGGGCCTTCCTCAC 70 
          |||||||||||||||||||||||| |||||||||||||||||||||||||||||||||||||||||||||    
Sbjct 121 TCACATTGCTGCGGGGTGGAGGCGATTTTAACTAACCCTAATGCAGAGTAAGTGGCTGGGCCTTCCTCAC 190

Query  71 ACTGCCTCCCGCTGCGTGAAACCTCGCTGTTTTCTTGGCTAACTTTCAGCGAGGTGAGGCAAGGCGGCAA 140
          |||||||||||||||| ||||||||||||||||||||||||||||||||||||||||||  |||||||||    
Sbjct 191 ACTGCCTCCCGCTGCGCGAAACCTCGCTGTTTTCTTGGCTAACTTTCAGCGAGGTGAGGAGAGGCGGCAA 260

Query 141 AAAAAA---GTTGGGAGCAGCAACGGCAAGCAAAAAAAAA-GTTCCAGCCGAGGCCTCCTCACCAC-GCG 205
          ||||||   ||||||||||||| |||||| |||||||||| ||||||||||||||||||||||| | |||    
Sbjct 261 AAAAAAAAGGTTGGGAGCAGCAGCGGCAAACAAAAAAAAAAGTTCCAGCCGAGGCCTCCTCACCCCCGCG 330

Query 206 GCCTGGGTCTGAGGGTAACATGGCCCTGCGGGAGAGAGCCAACACGGGGGGGCCCCGGTCCGGCTAACAG 275
          |||||||||||||||||||| ||||||||||||||||| |||||||||||  |||||||||||| |||||    
Sbjct 331 GCCTGGGTCTGAGGGTAACACGGCCCTGCGGGAGAGAGGCAACACGGGGGC-CCCCGGTCCGGCGAACAG 399

Query 276 GGCAGGAGGTCGGCCCCAATTGCCTCA--GGCCCAGCCACTCCGGGCCGCGATCAGCCCGGG-CTTTCCC 342
          ||||||||||||| |||||| ||||||  |||||||||||||||||||||| |||||||||| |||||||    
Sbjct 400 GGCAGGAGGTCGGTCCCAATCGCCTCACAGGCCCAGCCACTCCGGGCCGCGGTCAGCCCGGGGCTTTCCC 469

Query 343 TACT----TTGGGGCCCAATGCTGTCGCGAAGAGTTCGGCTCTGTCAGCCCGGCTGGGTCCGGGTGGGGG 408
            |     | |||||||||||||| ||||||||||||||||||||||||||||||||| | |||||||||    
Sbjct 470 CCCCACCATGGGGGCCCAATGCTGCCGCGAAGAGTTCGGCTCTGTCAGCCCGGCTGGGCCAGGGTGGGGG 539

Query 409 CCGGCACAGAGGGACACACCGGGCCGGGGCGCCGAGGATAGCTACCGCCCGCAGGGATAGTAAATATCCG 478
          |||||||||||||||||||||||||||||||||| ||||||||||||||||||||||||| |||||||||    
Sbjct 540 CCGGCACAGAGGGACACACCGGGCCGGGGCGCCGGGGATAGCTACCGCCCGCAGGGATAGCAAATATCCG 609

Query 479 TGACCAGTGGCCTCGGTGGCTGCGTCTCCCGGAGCAATGGGACCCGCAAGGCTGCCCTGGTCATACAC 546
          |||||||||| |||||||||||||||||||||||||||||||||||||||||||||||||||||||||    
Sbjct 610 TGACCAGTGGTCTCGGTGGCTGCGTCTCCCGGAGCAATGGGACCCGCAAGGCTGCCCTGGTCATACAC 677
```

Report:

|  |  |
| --- | --- |
| sequence start ?  ``` Start position of the estimated full-length sequence in genome. Start index < end index. ``` : | 121 |
| sequence end ?  ``` End position of the estimated full-length sequence in genome. Start index < end index. ``` : | 679 |
| bit score (CM) ?  ``` The score for aligning estimated full-length sequence to CM model   (computed by RSEARCH -> default,   infered from Rfam or provided by user) ``` : | 603.1 |
| Homology estimate ?  ``` Quick homology estimate:   Not homologous: bit score < 0   Homologous: bit score > 20 and bit score > 0.5 * query length   Uncertain otherwise ``` : | Homologous |

Estimated full-length sequence:


?

```
Click checkbox to select multiple seuqences.
Fasta header format:
  UID|accession.versionSTRAND start-end
```

>uid:1|AF221933.1fw 121-679
UCACAUUGCUGCGGGGUGGAGGCGAUUUUAACUAACCCUAAUGCAGAGUAAGUGGCUGGG
CCUUCCUCACACUGCCUCCCGCUGCGCGAAACCUCGCUGUUUUCUUGGCUAACUUUCAGC
GAGGUGAGGAGAGGCGGCAAAAAAAAAAGGUUGGGAGCAGCAGCGGCAAACAAAAAAAAA
AGUUCCAGCCGAGGCCUCCUCACCCCCGCGGCCUGGGUCUGAGGGUAACACGGCCCUGCG
GGAGAGAGGCAACACGGGGGCCCCCGGUCCGGCGAACAGGGCAGGAGGUCGGUCCCAAUC
GCCUCACAGGCCCAGCCACUCCGGGCCGCGGUCAGCCCGGGGCUUUCCCCCCCACCAUGG
GGGCCCAAUGCUGCCGCGAAGAGUUCGGCUCUGUCAGCCCGGCUGGGCCAGGGUGGGGGC
CGGCACAGAGGGACACACCGGGCCGGGGCGCCGGGGAUAGCUACCGCCCGCAGGGAUAGC
AAAUAUCCGUGACCAGUGGUCUCGGUGGCUGCGUCUCCCGGAGCAAUGGGACCCGCAAGG
CUGCCCUGGUCAUACACAU

C-A-r-Rc


?

```
Visualisation of predicted secondary structure.
To save the image:
  Right click on the image -> Save Image as.
```


centroid


?

```
Visualisation of predicted secondary structure.
To save the image:
  Right click on the image -> Save Image as.
```


fq-sub


?

```
Visualisation of predicted secondary structure.
To save the image:
  Right click on the image -> Save Image as.
```

Load Sequence viewer

uid:1|AF221933.1: Sequence cannot be extended sufficiently. Missing nt downstream in the genome.  
TurboFold: Number of sequences is less then required. n=2 (4)

### Hit: AF221935.1

AF221935.1 Rhinoptera bonasus telomerase RNA gene, sequence

```
?

```
This is BLAST alignment as read from the input file
```

Score = 82.0 bits (75.2), Expect = 9.91E-10
 Identities = 91/119 (76%), Gaps = 9/119 (8%)
 Strand = Plus/Plus
Query 285 TCGGCCCCAA---TTGC-CTCAGGCCCAGCC-ACTCCGGGCCGCGATCAGCCCGGGCTTTCCCTAC--TT 347
          |||||||| |   |||| | || |||||||| | |   ||||||| ||||| | ||||| | |  |  |     
Sbjct 366 TCGGCCCCCACCTTTGCTCGCATGCCCAGCCGAGTA--GGCCGCGGTCAGCGCCGGCTTCCACGCCAGTG 433

Query 348 TGGGGCCCAATGCTGTCGCGAAGAGTTCGGCTCTGTCAGCCCGGCTGGG 396
          ||||||   |||||||||||||||||||| ||||||||||| ||| |||    
Sbjct 434 TGGGGCTTGATGCTGTCGCGAAGAGTTCGTCTCTGTCAGCCTGGCAGGG 482
```

Report:

|  |  |
| --- | --- |
| sequence start ?  ``` Start position of the estimated full-length sequence in genome. Start index < end index. ``` : | 110 |
| sequence end ?  ``` End position of the estimated full-length sequence in genome. Start index < end index. ``` : | 602 |
| bit score (CM) ?  ``` The score for aligning estimated full-length sequence to CM model   (computed by RSEARCH -> default,   infered from Rfam or provided by user) ``` : | 75.57 |
| Homology estimate ?  ``` Quick homology estimate:   Not homologous: bit score < 0   Homologous: bit score > 20 and bit score > 0.5 * query length   Uncertain otherwise ``` : | Uncertain |

Estimated full-length sequence:


?

```
Click checkbox to select multiple seuqences.
Fasta header format:
  UID|accession.versionSTRAND start-end
```

>uid:2|AF221935.1fw 110-602
GCGUCCAGCUGCGGGGAGAAAGUGUCGUUCUCUAACCCUAAUGCGAGGGCAAUGCGGGCC
CUUUGUUUAAAGUCCCGUUGUCGCGCGAAGCGGGAGCCCUCGCUGUUUUCUUGGCUAACU
UUCAGCGAUGGUGAAAGGGCCUAGCAAAGGUUGGGAAGCGUGAUUUAUUGUUGGUGGGCA
GAGAGAAAAAAGUUUCAGCCGCGGCCUCCUCCCCGGCGGCCCAGGACCCCCUGUUGCAAC
GUGCGAAAUGGCGGCCUCGGCCCCCACCUUUGCUCGCAUGCCCAGCCGAGUAGGCCGCGG
UCAGCGCCGGCUUCCACGCCAGUGUGGGGCUUGAUGCUGUCGCGAAGAGUUCGUCUCUGU
CAGCCUGGCAGGGGGUGCUACGCAGGGUCGGGGUACGGGGGACAGGGAGAUUAAAUAGCU
UGACCAGACGCUAUAACCUGUACUGCAUCUCCCAGAGCUGUGGGACCUGCAAAUCUGCGC
UGGUCAUACAUUU

C-A-r-Rc


?

```
Visualisation of predicted secondary structure.
To save the image:
  Right click on the image -> Save Image as.
```


centroid


?

```
Visualisation of predicted secondary structure.
To save the image:
  Right click on the image -> Save Image as.
```


fq-sub


?

```
Visualisation of predicted secondary structure.
To save the image:
  Right click on the image -> Save Image as.
```

Load Sequence viewer

TurboFold: Number of sequences is less then required. n=2 (4)  
uid:2|AF221935.1: Sequence cannot be extended sufficiently. Missing nt downstream in the genome.  
uid:2|AF221935.1: Sequence cannot be extended sufficiently by unalined portion of query. THIS IS PROBABLY FRAGMENT! Trimmed downstream.

### Hit: AF221935.1

AF221935.1 Rhinoptera bonasus telomerase RNA gene, sequence

```
?

```
This is BLAST alignment as read from the input file
```

Score = 67.0 bits (61.7), Expect = 2.18E-05
 Identities = 58/71 (82%), Gaps = 5/71 (7%)
 Strand = Plus/Plus
Query  84 GCGTGAAACCTCGCTGTTTTCTTGGCTAACTTTCAGCGA-GGTGAGGCAAGGCGGCAAAAAAAAGTTGGG 152
          ||| ||  ||||||||||||||||||||||||||||||| |||||   |||| | | |  ||| ||||||    
Sbjct 199 GCGGGAGCCCTCGCTGTTTTCTTGGCTAACTTTCAGCGATGGTGA---AAGG-GCCTAGCAAAGGTTGGG 264

Query 153 A 153
          |    
Sbjct 265 A 265
```

Report:

|  |  |
| --- | --- |
| sequence start ?  ``` Start position of the estimated full-length sequence in genome. Start index < end index. ``` : | 110 |
| sequence end ?  ``` End position of the estimated full-length sequence in genome. Start index < end index. ``` : | 602 |
| bit score (CM) ?  ``` The score for aligning estimated full-length sequence to CM model   (computed by RSEARCH -> default,   infered from Rfam or provided by user) ``` : | 75.57 |
| Homology estimate ?  ``` Quick homology estimate:   Not homologous: bit score < 0   Homologous: bit score > 20 and bit score > 0.5 * query length   Uncertain otherwise ``` : | Uncertain |

Estimated full-length sequence:


?

```
Click checkbox to select multiple seuqences.
Fasta header format:
  UID|accession.versionSTRAND start-end
```

>uid:3|AF221935.1fw 110-602
GCGUCCAGCUGCGGGGAGAAAGUGUCGUUCUCUAACCCUAAUGCGAGGGCAAUGCGGGCC
CUUUGUUUAAAGUCCCGUUGUCGCGCGAAGCGGGAGCCCUCGCUGUUUUCUUGGCUAACU
UUCAGCGAUGGUGAAAGGGCCUAGCAAAGGUUGGGAAGCGUGAUUUAUUGUUGGUGGGCA
GAGAGAAAAAAGUUUCAGCCGCGGCCUCCUCCCCGGCGGCCCAGGACCCCCUGUUGCAAC
GUGCGAAAUGGCGGCCUCGGCCCCCACCUUUGCUCGCAUGCCCAGCCGAGUAGGCCGCGG
UCAGCGCCGGCUUCCACGCCAGUGUGGGGCUUGAUGCUGUCGCGAAGAGUUCGUCUCUGU
CAGCCUGGCAGGGGGUGCUACGCAGGGUCGGGGUACGGGGGACAGGGAGAUUAAAUAGCU
UGACCAGACGCUAUAACCUGUACUGCAUCUCCCAGAGCUGUGGGACCUGCAAAUCUGCGC
UGGUCAUACAUUU

C-A-r-Rc


?

```
Visualisation of predicted secondary structure.
To save the image:
  Right click on the image -> Save Image as.
```


centroid


?

```
Visualisation of predicted secondary structure.
To save the image:
  Right click on the image -> Save Image as.
```


fq-sub


?

```
Visualisation of predicted secondary structure.
To save the image:
  Right click on the image -> Save Image as.
```

Load Sequence viewer

uid:3|AF221935.1: Sequence cannot be extended sufficiently by unalined portion of query. THIS IS PROBABLY FRAGMENT! Trimmed downstream.  
TurboFold: Number of sequences is less then required. n=2 (4)  
uid:3|AF221935.1: Sequence cannot be extended sufficiently. Missing nt downstream in the genome.

### Hit: AF221935.1

AF221935.1 Rhinoptera bonasus telomerase RNA gene, sequence

```
?

```
This is BLAST alignment as read from the input file
```

Score = 56.0 bits (51.8), Expect = 1.13E-02
 Identities = 40/48 (83%), Gaps = 0/48 (0%)
 Strand = Plus/Plus
Query 498 CTGCGTCTCCCGGAGCAATGGGACCCGCAAGGCTGCCCTGGTCATACA 545
          |||| |||||| ||||  ||||||| ||||  |||| |||||||||||    
Sbjct 552 CTGCATCTCCCAGAGCTGTGGGACCTGCAAATCTGCGCTGGTCATACA 599
```

Report:

|  |  |
| --- | --- |
| sequence start ?  ``` Start position of the estimated full-length sequence in genome. Start index < end index. ``` : | 110 |
| sequence end ?  ``` End position of the estimated full-length sequence in genome. Start index < end index. ``` : | 602 |
| bit score (CM) ?  ``` The score for aligning estimated full-length sequence to CM model   (computed by RSEARCH -> default,   infered from Rfam or provided by user) ``` : | 75.57 |
| Homology estimate ?  ``` Quick homology estimate:   Not homologous: bit score < 0   Homologous: bit score > 20 and bit score > 0.5 * query length   Uncertain otherwise ``` : | Uncertain |

Estimated full-length sequence:


?

```
Click checkbox to select multiple seuqences.
Fasta header format:
  UID|accession.versionSTRAND start-end
```

>uid:4|AF221935.1fw 110-602
GCGUCCAGCUGCGGGGAGAAAGUGUCGUUCUCUAACCCUAAUGCGAGGGCAAUGCGGGCC
CUUUGUUUAAAGUCCCGUUGUCGCGCGAAGCGGGAGCCCUCGCUGUUUUCUUGGCUAACU
UUCAGCGAUGGUGAAAGGGCCUAGCAAAGGUUGGGAAGCGUGAUUUAUUGUUGGUGGGCA
GAGAGAAAAAAGUUUCAGCCGCGGCCUCCUCCCCGGCGGCCCAGGACCCCCUGUUGCAAC
GUGCGAAAUGGCGGCCUCGGCCCCCACCUUUGCUCGCAUGCCCAGCCGAGUAGGCCGCGG
UCAGCGCCGGCUUCCACGCCAGUGUGGGGCUUGAUGCUGUCGCGAAGAGUUCGUCUCUGU
CAGCCUGGCAGGGGGUGCUACGCAGGGUCGGGGUACGGGGGACAGGGAGAUUAAAUAGCU
UGACCAGACGCUAUAACCUGUACUGCAUCUCCCAGAGCUGUGGGACCUGCAAAUCUGCGC
UGGUCAUACAUUU

C-A-r-Rc


?

```
Visualisation of predicted secondary structure.
To save the image:
  Right click on the image -> Save Image as.
```


centroid


?

```
Visualisation of predicted secondary structure.
To save the image:
  Right click on the image -> Save Image as.
```


fq-sub


?

```
Visualisation of predicted secondary structure.
To save the image:
  Right click on the image -> Save Image as.
```

Load Sequence viewer

uid:4|AF221935.1: Sequence cannot be extended sufficiently. Missing nt downstream in the genome.  
TurboFold: Number of sequences is less then required. n=2 (4)

### Hit: AF221914.1

AF221914.1 Dasyatis sabina telomerase RNA gene, sequence

```
?

```
This is BLAST alignment as read from the input file
```

Score = 78.0 bits (71.6), Expect = 1.21E-08
 Identities = 88/116 (76%), Gaps = 6/116 (5%)
 Strand = Plus/Plus
Query 285 TCGGCCCCAATTGCCTCAGGCCCAGCCACTCCGGGCCGCGATCAGCCCGGGCTTTCC---CTACTTTGGG 351
          ||||||||  | ||  || ||||||||  |  || ||||| ||||| | || |||||   | | | ||||    
Sbjct 358 TCGGCCCCCCT-GCTGCACGCCCAGCCGTTTAGG-CCGCGGTCAGCGCCGG-TTTCCACGCCAGTGTGGG 424

Query 352 GCCCAATGCTGTCGCGAAGAGTTCGGCTCTGTCAGCCCGGCTGGGT 397
          ||   |||||||||||||||||||| |||||| |||| ||| ||||    
Sbjct 425 GCTTGATGCTGTCGCGAAGAGTTCGTCTCTGTTAGCCTGGCAGGGT 470
```

Report:

|  |  |
| --- | --- |
| sequence start ?  ``` Start position of the estimated full-length sequence in genome. Start index < end index. ``` : | 57 |
| sequence end ?  ``` End position of the estimated full-length sequence in genome. Start index < end index. ``` : | 589 |
| bit score (CM) ?  ``` The score for aligning estimated full-length sequence to CM model   (computed by RSEARCH -> default,   infered from Rfam or provided by user) ``` : | 62.6 |
| Homology estimate ?  ``` Quick homology estimate:   Not homologous: bit score < 0   Homologous: bit score > 20 and bit score > 0.5 * query length   Uncertain otherwise ``` : | Uncertain |

Estimated full-length sequence:


?

```
Click checkbox to select multiple seuqences.
Fasta header format:
  UID|accession.versionSTRAND start-end
```

>uid:5|AF221914.1fw 57-589
GCCGGGUUAAUGACGUCACGAAGCCUAUAUCAGCGCAGGUUCCCGGCGGCGCCGUGUCCA
GCUGCGGGGGAGACAGUGUCGCUCUCUAACCCUAAUGUGGGGGCAACGCGGGCCCUUUGU
UUAAAGUCCCGCUGUCGGGAGCCCUCGCUGUUUUCUUGGCUAACUUUCAGCGAUGGCGAA
AGGGCCCAGCAAAGGUUGGGAAGCGUGAUUUAUUGUUGGUGGGCAGAGAGAAAAAAGUUU
AAGCCGCGGCCUCCUCCCCGGCGGCCCAGGACCCCCUGUUGCAACGUGCGGAAUGGCGGC
CUCGGCCCCCCUGCUGCACGCCCAGCCGUUUAGGCCGCGGUCAGCGCCGGUUUCCACGCC
AGUGUGGGGCUUGAUGCUGUCGCGAAGAGUUCGUCUCUGUUAGCCUGGCAGGGUGUGCUA
CGCAGGGUCGGGGUGCGGAGGGCAGGGAGAUUAAAUAGCUUGACCAGACGCUAUGGCCUG
UACUGCAUUUCCCAGAGCUGUGGGACCUGCAAAUCUGCGCUGGUCAUACAUUU

C-A-r-Rc


?

```
Visualisation of predicted secondary structure.
To save the image:
  Right click on the image -> Save Image as.
```


centroid


?

```
Visualisation of predicted secondary structure.
To save the image:
  Right click on the image -> Save Image as.
```


fq-sub


?

```
Visualisation of predicted secondary structure.
To save the image:
  Right click on the image -> Save Image as.
```

Load Sequence viewer

TurboFold: Number of sequences is less then required. n=2 (4)  
uid:5|AF221914.1: Sequence cannot be extended sufficiently. Missing nt downstream in the genome.  
uid:5|AF221914.1: Sequence cannot be extended sufficiently by unalined portion of query. THIS IS PROBABLY FRAGMENT! Trimmed downstream.

### Hit: AF221914.1

AF221914.1 Dasyatis sabina telomerase RNA gene, sequence

```
?

```
This is BLAST alignment as read from the input file
```

Score = 70.0 bits (64.4), Expect = 1.79E-06
 Identities = 43/47 (91%), Gaps = 1/47 (2%)
 Strand = Plus/Plus
Query  77 TCCCGCTG-CGTGAAACCTCGCTGTTTTCTTGGCTAACTTTCAGCGA 122
          |||||||| || ||  |||||||||||||||||||||||||||||||    
Sbjct 183 TCCCGCTGTCGGGAGCCCTCGCTGTTTTCTTGGCTAACTTTCAGCGA 229
```

Report:

|  |  |
| --- | --- |
| sequence start ?  ``` Start position of the estimated full-length sequence in genome. Start index < end index. ``` : | 113 |
| sequence end ?  ``` End position of the estimated full-length sequence in genome. Start index < end index. ``` : | 589 |
| bit score (CM) ?  ``` The score for aligning estimated full-length sequence to CM model   (computed by RSEARCH -> default,   infered from Rfam or provided by user) ``` : | 100.81 |
| Homology estimate ?  ``` Quick homology estimate:   Not homologous: bit score < 0   Homologous: bit score > 20 and bit score > 0.5 * query length   Uncertain otherwise ``` : | Uncertain |

Estimated full-length sequence:


?

```
Click checkbox to select multiple seuqences.
Fasta header format:
  UID|accession.versionSTRAND start-end
```

>uid:6|AF221914.1fw 113-589
UCCAGCUGCGGGGGAGACAGUGUCGCUCUCUAACCCUAAUGUGGGGGCAACGCGGGCCCU
UUGUUUAAAGUCCCGCUGUCGGGAGCCCUCGCUGUUUUCUUGGCUAACUUUCAGCGAUGG
CGAAAGGGCCCAGCAAAGGUUGGGAAGCGUGAUUUAUUGUUGGUGGGCAGAGAGAAAAAA
GUUUAAGCCGCGGCCUCCUCCCCGGCGGCCCAGGACCCCCUGUUGCAACGUGCGGAAUGG
CGGCCUCGGCCCCCCUGCUGCACGCCCAGCCGUUUAGGCCGCGGUCAGCGCCGGUUUCCA
CGCCAGUGUGGGGCUUGAUGCUGUCGCGAAGAGUUCGUCUCUGUUAGCCUGGCAGGGUGU
GCUACGCAGGGUCGGGGUGCGGAGGGCAGGGAGAUUAAAUAGCUUGACCAGACGCUAUGG
CCUGUACUGCAUUUCCCAGAGCUGUGGGACCUGCAAAUCUGCGCUGGUCAUACAUUU

C-A-r-Rc


?

```
Visualisation of predicted secondary structure.
To save the image:
  Right click on the image -> Save Image as.
```


centroid


?

```
Visualisation of predicted secondary structure.
To save the image:
  Right click on the image -> Save Image as.
```


fq-sub


?

```
Visualisation of predicted secondary structure.
To save the image:
  Right click on the image -> Save Image as.
```

Load Sequence viewer

uid:6|AF221914.1: Sequence cannot be extended sufficiently. Missing nt downstream in the genome.  
TurboFold: Number of sequences is less then required. n=2 (4)  
uid:6|AF221914.1: Sequence cannot be extended sufficiently by unalined portion of query. THIS IS PROBABLY FRAGMENT! Trimmed downstream.

### Hit: AF221914.1

AF221914.1 Dasyatis sabina telomerase RNA gene, sequence

```
?

```
This is BLAST alignment as read from the input file
```

Score = 56.0 bits (51.8), Expect = 1.13E-02
 Identities = 69/93 (74%), Gaps = 10/93 (11%)
 Strand = Plus/Plus
Query 459 GCAGGGATAGTAAATATCCGTGACCAG------TGGCCTCGGTGGCTGCGTCTCCCGGAGCAATGGGACC 522
          ||||||| | |||||| |  |||||||      ||||||  ||  |||| | |||| ||||  |||||||    
Sbjct 498 GCAGGGAGATTAAATAGCT-TGACCAGACGCTATGGCCT--GTA-CTGCATTTCCCAGAGCTGTGGGACC 563

Query 523 CGCAAGGCTGCCCTGGTCATACA 545
           ||||  |||| |||||||||||    
Sbjct 564 TGCAAATCTGCGCTGGTCATACA 586
```

Report:

|  |  |
| --- | --- |
| sequence start ?  ``` Start position of the estimated full-length sequence in genome. Start index < end index. ``` : | 54 |
| sequence end ?  ``` End position of the estimated full-length sequence in genome. Start index < end index. ``` : | 589 |
| bit score (CM) ?  ``` The score for aligning estimated full-length sequence to CM model   (computed by RSEARCH -> default,   infered from Rfam or provided by user) ``` : | 63.35 |
| Homology estimate ?  ``` Quick homology estimate:   Not homologous: bit score < 0   Homologous: bit score > 20 and bit score > 0.5 * query length   Uncertain otherwise ``` : | Uncertain |

Estimated full-length sequence:


?

```
Click checkbox to select multiple seuqences.
Fasta header format:
  UID|accession.versionSTRAND start-end
```

>uid:7|AF221914.1fw 54-589
UGGGCCGGGUUAAUGACGUCACGAAGCCUAUAUCAGCGCAGGUUCCCGGCGGCGCCGUGU
CCAGCUGCGGGGGAGACAGUGUCGCUCUCUAACCCUAAUGUGGGGGCAACGCGGGCCCUU
UGUUUAAAGUCCCGCUGUCGGGAGCCCUCGCUGUUUUCUUGGCUAACUUUCAGCGAUGGC
GAAAGGGCCCAGCAAAGGUUGGGAAGCGUGAUUUAUUGUUGGUGGGCAGAGAGAAAAAAG
UUUAAGCCGCGGCCUCCUCCCCGGCGGCCCAGGACCCCCUGUUGCAACGUGCGGAAUGGC
GGCCUCGGCCCCCCUGCUGCACGCCCAGCCGUUUAGGCCGCGGUCAGCGCCGGUUUCCAC
GCCAGUGUGGGGCUUGAUGCUGUCGCGAAGAGUUCGUCUCUGUUAGCCUGGCAGGGUGUG
CUACGCAGGGUCGGGGUGCGGAGGGCAGGGAGAUUAAAUAGCUUGACCAGACGCUAUGGC
CUGUACUGCAUUUCCCAGAGCUGUGGGACCUGCAAAUCUGCGCUGGUCAUACAUUU

C-A-r-Rc


?

```
Visualisation of predicted secondary structure.
To save the image:
  Right click on the image -> Save Image as.
```


centroid


?

```
Visualisation of predicted secondary structure.
To save the image:
  Right click on the image -> Save Image as.
```


fq-sub


?

```
Visualisation of predicted secondary structure.
To save the image:
  Right click on the image -> Save Image as.
```

Load Sequence viewer

uid:7|AF221914.1: Sequence cannot be extended sufficiently. Missing nt downstream in the genome.  
TurboFold: Number of sequences is less then required. n=2 (4)

### Hit: XM\_011380099.2

XM\_011380099.2 PREDICTED: Pteropus vampyrus actin related protein T3 (ACTRT3), mRNA

```
?

```
This is BLAST alignment as read from the input file
```

Score = 72.0 bits (66.2), Expect = 5.13E-07
 Identities = 72/93 (77%), Gaps = 6/93 (6%)
 Strand = Plus/Plus
Query  318 GGCCGCGATCAGCCCGGGCTTTCCCTACTTTGGGGCCCAATGCTGTCGCGAAGAGTTCGGCTCTGTCA 385 
           ||||||| || |||||||  ||| |  |   || ||||||||| | ||||||||||| ||||||||||     
Sbjct 4800 GGCCGCGGTCGGCCCGGGACTTCTC--CGGAGGTGCCCAATGCCGCCGCGAAGAGTTGGGCTCTGTCA 4865

Query  386 GCCCGGCTGGGTCCGGGTGGGGGCC 410 
           |||  || || |||   ||||||||     
Sbjct 4866 GCC--GCGGGTTCC--TTGGGGGCC 4886
```

Report:

|  |  |
| --- | --- |
| sequence start ?  ``` Start position of the estimated full-length sequence in genome. Start index < end index. ``` : | 4491 |
| sequence end ?  ``` End position of the estimated full-length sequence in genome. Start index < end index. ``` : | 4990 |
| bit score (CM) ?  ``` The score for aligning estimated full-length sequence to CM model   (computed by RSEARCH -> default,   infered from Rfam or provided by user) ``` : | 2.76 |
| Homology estimate ?  ``` Quick homology estimate:   Not homologous: bit score < 0   Homologous: bit score > 20 and bit score > 0.5 * query length   Uncertain otherwise ``` : | Uncertain |

Estimated full-length sequence:


?

```
Click checkbox to select multiple seuqences.
Fasta header format:
  UID|accession.versionSTRAND start-end
```

>uid:8|XM\_011380099.2fw 4491-4990
GGCCAAUCCGCGCGGGCAGCGCCCGCUCCUUUAUAAGGAGAUGCGGCUGGUGCGCGCUGG
GUUGCGGAGGAUGGGUCUGGGUUGAGUGACGGCCUGUUUCUUUCUAACCCUAUUUGAGAA
UGGCGUAGGCGCCGUGCUUUUGCUCCCCGCUCGCUGUUUUUCUCGCUGGCUUUCAGCGCG
CGGAAAAGCCUCGGCCUACCGCCGUCCACCGUCCGAUCUAGAACAAACAAAAAAUGUCAG
CGACUGGUCAAGUCACCCUUCCCAGGAACCUGUGGAAUUCGCCCGCCCGGCCCCCGCACC
CCGCCUAGAGGCCGCGGUCGGCCCGGGACUUCUCCGGAGGUGCCCAAUGCCGCCGCGAAG
AGUUGGGCUCUGUCAGCCGCGGGUUCCUUGGGGGCCAAGGGCGAGGCUGUGGCCACAGGG
AGAGAAACGGAGCGGGUUCCCGCGCGCGGUGCACUUCCCUGAGCUGUGGGACUUGCAUCC
GGGACUGGGCUCGGACAUAC

C-A-r-Rc


?

```
Visualisation of predicted secondary structure.
To save the image:
  Right click on the image -> Save Image as.
```


centroid


?

```
Visualisation of predicted secondary structure.
To save the image:
  Right click on the image -> Save Image as.
```


fq-sub


?

```
Visualisation of predicted secondary structure.
To save the image:
  Right click on the image -> Save Image as.
```

Load Sequence viewer

TurboFold: Number of sequences is less then required. n=2 (4)  
uid:8|XM\_011380099.2: Sequence cannot be extended sufficiently by unalined portion of query. THIS IS PROBABLY FRAGMENT! Trimmed downstream.  
uid:8|XM\_011380099.2: Sequence cannot be extended sufficiently. Missing nt downstream in the genome.

### Hit: EU486822.1

EU486822.1 Equus asinus telomerase RNA gene, complete sequence >EU486824.1 Equus grevyi telomerase RNA gene, complete sequence

```
?

```
This is BLAST alignment as read from the input file
```

Score = 70.0 bits (64.4), Expect = 1.79E-06
 Identities = 67/86 (78%), Gaps = 3/86 (3%)
 Strand = Plus/Plus
Query 318 GGCCGCGATCAGCCCGGGCTTTCCCTACTTTGGGGCCCAATGCTGTCGCGAAGAGTTCGGCTCTGTCAGC 387
          ||||||| || |||||||  ||| |  |   || ||||||||| | ||||||||||| ||||||||||||    
Sbjct 253 GGCCGCGGTCGGCCCGGGGCTTCTC--CGGAGGCGCCCAATGCCGCCGCGAAGAGTTGGGCTCTGTCAGC 320

Query 388 C-CGGCTGGGTCCGGG 402
          | ||| ||  || |||    
Sbjct 321 CGCGGGTGCCTCGGGG 336
```

Report:

|  |  |
| --- | --- |
| sequence start ?  ``` Start position of the estimated full-length sequence in genome. Start index < end index. ``` : | 1 |
| sequence end ?  ``` End position of the estimated full-length sequence in genome. Start index < end index. ``` : | 443 |
| bit score (CM) ?  ``` The score for aligning estimated full-length sequence to CM model   (computed by RSEARCH -> default,   infered from Rfam or provided by user) ``` : | 31.18 |
| Homology estimate ?  ``` Quick homology estimate:   Not homologous: bit score < 0   Homologous: bit score > 20 and bit score > 0.5 * query length   Uncertain otherwise ``` : | Uncertain |

Estimated full-length sequence:


?

```
Click checkbox to select multiple seuqences.
Fasta header format:
  UID|accession.versionSTRAND start-end
```

>uid:9|EU486822.1fw 1-443
GGGUGGGGGAGAGUGGGUCUGGGCGGGGCGGCGGUCACGUUUUGUCUAACCCUAACUGAG
CUGGGCGGAGGCGCCGCGCUUUUGCUCCCCGCGCGCUGUUUUUCUCGCUGACUUUCAGCG
GGCGGAAAAGCCUCGGUCUACCGCCACUUACCAUCCAGUCUGGAGCAAACAAAAAAUGUC
AGCCGCUGGCUCGCUCGCCCCUCCCGGGACCCUGCGACGGCUCGCCCGCCCAGCCCCCGC
GCCCCGCCUGGAGGCCGCGGUCGGCCCGGGGCUUCUCCGGAGGCGCCCAAUGCCGCCGCG
AAGAGUUGGGCUCUGUCAGCCGCGGGUGCCUCGGGGGCCAGGGACGAGGCUCUGGCCGCA
GGGAGAGGAACGGAGCGGGUCCCCGCGCGCGGUGCGCUUCCCUGAGCUGUGGGACGUGCA
CCCGGGACUCGGCUCAAACACGU

C-A-r-Rc


?

```
Visualisation of predicted secondary structure.
To save the image:
  Right click on the image -> Save Image as.
```


centroid


?

```
Visualisation of predicted secondary structure.
To save the image:
  Right click on the image -> Save Image as.
```


fq-sub


?

```
Visualisation of predicted secondary structure.
To save the image:
  Right click on the image -> Save Image as.
```

Load Sequence viewer

uid:9|EU486822.1: Sequence cannot be extended sufficiently by unaligned portion of query. THIS IS PROBABLY FRAGMENT! Trimmed upstream.  
TurboFold: Number of sequences is less then required. n=2 (4)  
uid:9|EU486822.1: Sequence cannot be extended sufficiently. Missing nt downstream in the genome.  
uid:9|EU486822.1: Sequence cannot be extended sufficiently. Missing -94 nt upstream in the genome.

### Hit: AF221925.1

AF221925.1 Equus caballus telomerase RNA gene, sequence

```
?

```
This is BLAST alignment as read from the input file
```

Score = 70.0 bits (64.4), Expect = 1.79E-06
 Identities = 67/86 (78%), Gaps = 3/86 (3%)
 Strand = Plus/Plus
Query 318 GGCCGCGATCAGCCCGGGCTTTCCCTACTTTGGGGCCCAATGCTGTCGCGAAGAGTTCGGCTCTGTCAGC 387
          ||||||| || |||||||  ||| |  |   || ||||||||| | ||||||||||| ||||||||||||    
Sbjct 350 GGCCGCGGTCGGCCCGGGGCTTCTC--CGGAGGCGCCCAATGCCGCCGCGAAGAGTTGGGCTCTGTCAGC 417

Query 388 C-CGGCTGGGTCCGGG 402
          | ||| ||  || |||    
Sbjct 418 CGCGGGTGCCTCGGGG 433
```

Report:

|  |  |
| --- | --- |
| sequence start ?  ``` Start position of the estimated full-length sequence in genome. Start index < end index. ``` : | 40 |
| sequence end ?  ``` End position of the estimated full-length sequence in genome. Start index < end index. ``` : | 540 |
| bit score (CM) ?  ``` The score for aligning estimated full-length sequence to CM model   (computed by RSEARCH -> default,   infered from Rfam or provided by user) ``` : | 30.91 |
| Homology estimate ?  ``` Quick homology estimate:   Not homologous: bit score < 0   Homologous: bit score > 20 and bit score > 0.5 * query length   Uncertain otherwise ``` : | Uncertain |

Estimated full-length sequence:


?

```
Click checkbox to select multiple seuqences.
Fasta header format:
  UID|accession.versionSTRAND start-end
```

>uid:10|AF221925.1fw 40-540
GCCAAUGGGCGCGGGCGGCGGUGCUCCCUUUAUAAGGAGGUGCGGCCAGGCACGCGGCGG
GUGGGGGAGAGUGGGUCUGGGCGGGGCGGCGGUCACGUUUUGUCUAACCCUAACUGAGCU
GGGCGGAGGCGCCGCGCUUUUGCUCCCCGCGCGCUGUUUUUCUCGCUGACUUUCAGCGGG
CGGAAAAGCCUCGGUCUACCGCCACUUACCAUCCAGUCUGGAGUAAACAAAAAAUGUCAG
CCGCUGGCUCGCUCGCCCCUCCCGGGACCCUGCGACGGCUCGCCCGCCCAGCCCCCGCGC
CCCGCCUGGAGGCCGCGGUCGGCCCGGGGCUUCUCCGGAGGCGCCCAAUGCCGCCGCGAA
GAGUUGGGCUCUGUCAGCCGCGGGUGCCUCGGGGGCCAGGGACGAGGCUCUGGCCGCAGG
GAGAGGAACGGAGCGGGUCCCCGCGCGCGGUGCGCUUCCCUGAGCUGUGGGACGUGCACC
CGGGACUCGGCUCAAACACGU

C-A-r-Rc


?

```
Visualisation of predicted secondary structure.
To save the image:
  Right click on the image -> Save Image as.
```


centroid


?

```
Visualisation of predicted secondary structure.
To save the image:
  Right click on the image -> Save Image as.
```


fq-sub


?

```
Visualisation of predicted secondary structure.
To save the image:
  Right click on the image -> Save Image as.
```

Load Sequence viewer

uid:10|AF221925.1: Sequence cannot be extended sufficiently. Missing nt downstream in the genome.  
TurboFold: Number of sequences is less then required. n=2 (4)  
uid:10|AF221925.1: Sequence cannot be extended sufficiently by unalined portion of query. THIS IS PROBABLY FRAGMENT! Trimmed downstream.

### Hit: AY833720.1

AY833720.1 Canis familiaris telomerase RNA (TR) gene, partial sequence

```
?

```
This is BLAST alignment as read from the input file
```

Score = 69.0 bits (63.5), Expect = 6.25E-06
 Identities = 72/94 (77%), Gaps = 6/94 (6%)
 Strand = Plus/Plus
Query 318 GGCCGCGATCAGCCCGGGCTTTCCCTACTTTGGGGCCCAATGCTGTCGCGAAGAGTTCGGCTCTGTCAGC 387
          |||||||  | |||||||  ||| |  |   || ||||| ||| ||||||||||||| ||||||||||||    
Sbjct 155 GGCCGCGGCCGGCCCGGGGCTTCTC--CGGAGGCGCCCACTGCCGTCGCGAAGAGTTGGGCTCTGTCAGC 222

Query 388 CCGGCTGGGTCCGGGTGGGGGCCG 411
          |  ||  || |||  |||||||||    
Sbjct 223 C--GC--GGGCCGCCTGGGGGCCG 242
```

Report:

|  |  |
| --- | --- |
| sequence start ?  ``` Start position of the estimated full-length sequence in genome. Start index < end index. ``` : | 1 |
| sequence end ?  ``` End position of the estimated full-length sequence in genome. Start index < end index. ``` : | 290 |
| bit score (CM) ?  ``` The score for aligning estimated full-length sequence to CM model   (computed by RSEARCH -> default,   infered from Rfam or provided by user) ``` : | 16.69 |
| Homology estimate ?  ``` Quick homology estimate:   Not homologous: bit score < 0   Homologous: bit score > 20 and bit score > 0.5 * query length   Uncertain otherwise ``` : | Uncertain |

Estimated full-length sequence:


?

```
Click checkbox to select multiple seuqences.
Fasta header format:
  UID|accession.versionSTRAND start-end
```

>uid:11|AY833720.1fw 1-290
UUCGCUGACUUUCAGCGGGCGGAAAAGCCUCGGCCUACCGCCGUCCACCGUCCGGGUCUG
CAGCCAACAACAAAAAAUGUCAGCCGCUGGCUCGCUCGCCCCUCCCGGGAGCCUGCGGCC
GCUCGCCCGCUCGGCCCCCCGCGUCCCGCCCGGAGGCCGCGGCCGGCCCGGGGCUUCUCC
GGAGGCGCCCACUGCCGUCGCGAAGAGUUGGGCUCUGUCAGCCGCGGGCCGCCUGGGGGC
CGCAGGGCGGGGCUCGGGCCGCAGGGAGAGCAACGGAGCGGGUCCCCGCG

C-A-r-Rc


?

```
Visualisation of predicted secondary structure.
To save the image:
  Right click on the image -> Save Image as.
```


centroid


?

```
Visualisation of predicted secondary structure.
To save the image:
  Right click on the image -> Save Image as.
```


fq-sub


?

```
Visualisation of predicted secondary structure.
To save the image:
  Right click on the image -> Save Image as.
```

Load Sequence viewer

uid:11|AY833720.1: Sequence cannot be extended sufficiently. Missing nt downstream in the genome.  
TurboFold: Number of sequences is less then required. n=2 (4)  
uid:11|AY833720.1: Sequence cannot be extended sufficiently. Missing -192 nt upstream in the genome.  
uid:11|AY833720.1: Sequence cannot be extended sufficiently by unaligned portion of query. THIS IS PROBABLY FRAGMENT! Trimmed upstream.

### Hit: AF221918.1

AF221918.1 Oryctolagus cuniculus telomerase RNA gene, sequence

```
?

```
This is BLAST alignment as read from the input file
```

Score = 69.0 bits (63.5), Expect = 6.25E-06
 Identities = 57/71 (80%), Gaps = 2/71 (3%)
 Strand = Plus/Plus
Query 318 GGCCGCGATCAGCCCGGGCTTTCCCTACTTTGGGGCCCAATGCTGTCGCGAAGAGTTCGGCTCTGTCAGC 387
          ||||||| || |||||||  ||| |  |   || ||||||||| | ||||||||||| ||||||||||||    
Sbjct 349 GGCCGCGGTCGGCCCGGGGCTTCTC--CGGAGGTGCCCAATGCCGCCGCGAAGAGTTAGGCTCTGTCAGC 416

Query 388 C 388
          |    
Sbjct 417 C 417
```

Report:

|  |  |
| --- | --- |
| sequence start ?  ``` Start position of the estimated full-length sequence in genome. Start index < end index. ``` : | 26 |
| sequence end ?  ``` End position of the estimated full-length sequence in genome. Start index < end index. ``` : | 547 |
| bit score (CM) ?  ``` The score for aligning estimated full-length sequence to CM model   (computed by RSEARCH -> default,   infered from Rfam or provided by user) ``` : | 32.03 |
| Homology estimate ?  ``` Quick homology estimate:   Not homologous: bit score < 0   Homologous: bit score > 20 and bit score > 0.5 * query length   Uncertain otherwise ``` : | Uncertain |

Estimated full-length sequence:


?

```
Click checkbox to select multiple seuqences.
Fasta header format:
  UID|accession.versionSTRAND start-end
```

>uid:12|AF221918.1fw 26-547
ACGCUCGGCCUCGGCCAAUCCGCGCGCGCAGCGCCGCUCCCUUUAUAAGACGACUCCGGC
CGGCGCGCGGCGGGCUGAGGAGGGUGGGCUCGGGAGGGGCCCGGUCAUUUCUCAUCUAAC
CCUAACUGAGCAGGGCGUAGGCGCCGCGCUUUUGUUUCCCCGCGCGCUGUUUUUCUCGCU
GACUUUCAGCGCGUGGGAAAAGCCUUGGCCUACCGCCGUCCACCGUUCAUUUCGCAGUAA
ACAAAAAAUGUCAGCCGCUGGCCGGUUCGCCCUUCCCGGGGACCUGCGGUGGCUCGCCCG
CCCGGCCCCCGUGCCCCGCCUGAGGCCGCGGUCGGCCCGGGGCUUCUCCGGAGGUGCCCA
AUGCCGCCGCGAAGAGUUAGGCUCUGUCAGCCGCGGGUCCCUCGGGGGCCAAGGGCGAGG
CGCAGGCCGUCUGGCCGCAGGGAGAGGAACGGAGCGGGUCCCCCAGCGUGGUGCGCUUCC
CUGAGCUGUGGGACUUGCACCCGGGACUCGGCUCAAACACGC

C-A-r-Rc


?

```
Visualisation of predicted secondary structure.
To save the image:
  Right click on the image -> Save Image as.
```


centroid


?

```
Visualisation of predicted secondary structure.
To save the image:
  Right click on the image -> Save Image as.
```


fq-sub


?

```
Visualisation of predicted secondary structure.
To save the image:
  Right click on the image -> Save Image as.
```

Load Sequence viewer

uid:12|AF221918.1: Sequence cannot be extended sufficiently. Missing nt downstream in the genome.  
uid:12|AF221918.1: Sequence cannot be extended sufficiently by unalined portion of query. THIS IS PROBABLY FRAGMENT! Trimmed downstream.  
TurboFold: Number of sequences is less then required. n=2 (4)

### Hit: AF221910.1

AF221910.1 Typhlonectes natans telomerase RNA gene, sequence

```
?

```
This is BLAST alignment as read from the input file
```

Score = 68.0 bits (62.6), Expect = 6.25E-06
 Identities = 58/73 (79%), Gaps = 2/73 (3%)
 Strand = Plus/Plus
Query 318 GGCCGCGATCAGCCCGGGC--TTTCCCTACTTTGGGGCCCAATGCTGTCGCGAAGAGTTCGGCTCTGTCA 385
          ||||||| |||| ||||||  || | ||    || || ||| ||||| ||||||||||||| ||||||||    
Sbjct 351 GGCCGCGGTCAGTCCGGGCCCTTGCTCTGGCATGAGGTCCATTGCTGCCGCGAAGAGTTCGTCTCTGTCA 420

Query 386 GCC 388
          |||    
Sbjct 421 GCC 423
```

Report:

|  |  |
| --- | --- |
| sequence start ?  ``` Start position of the estimated full-length sequence in genome. Start index < end index. ``` : | 41 |
| sequence end ?  ``` End position of the estimated full-length sequence in genome. Start index < end index. ``` : | 554 |
| bit score (CM) ?  ``` The score for aligning estimated full-length sequence to CM model   (computed by RSEARCH -> default,   infered from Rfam or provided by user) ``` : | 28.93 |
| Homology estimate ?  ``` Quick homology estimate:   Not homologous: bit score < 0   Homologous: bit score > 20 and bit score > 0.5 * query length   Uncertain otherwise ``` : | Uncertain |

Estimated full-length sequence:


?

```
Click checkbox to select multiple seuqences.
Fasta header format:
  UID|accession.versionSTRAND start-end
```

>uid:13|AF221910.1fw 41-554
CCAAUCAGCUGCCGGCUGGAGGCGCGCGGUGAAAACGAGAGUAUAAAAUGCAGAACUUGC
ACUCAUAGCAUUGUCAUUCCGGAGAGGGGCGUUUUCUUCUUUCUCUAACCCUAAUGUAUU
GCUUGGGCAGCAGCUCUCUCGGUAUGCUCGCUGUUUUGCUGGCUAACUUUCAGCGAGCAG
AGGGCGAAGUCGAAUUUCGGACAAGGGAGAAAAAAUGUUAGCCGGGGAACGCCCCUUUCC
CGAAUGCCCGUGCCGUCCUUCCUCUCCUCUGGGCCCGUUGGCGAUAGCCCCCGGCCUCCC
UCGCUCCUUAGGCCGCGGUCAGUCCGGGCCCUUGCUCUGGCAUGAGGUCCAUUGCUGCCG
CGAAGAGUUCGUCUCUGUCAGCCUUGAGGUGGCCAGCAUGGAAUUGGCGGGCUUGUGGCG
GCGCGCGGGAAAAAGCAAAUGGUGAACUAGGUGCGUGUUGUGGUCGUGUUCCUGAAGCGU
GGAAUGUGCGAUCUGUGCGCUAGUUCAUACACAC

C-A-r-Rc


?

```
Visualisation of predicted secondary structure.
To save the image:
  Right click on the image -> Save Image as.
```


centroid


?

```
Visualisation of predicted secondary structure.
To save the image:
  Right click on the image -> Save Image as.
```


fq-sub


?

```
Visualisation of predicted secondary structure.
To save the image:
  Right click on the image -> Save Image as.
```

Load Sequence viewer

TurboFold: Number of sequences is less then required. n=2 (4)  
uid:13|AF221910.1: Sequence cannot be extended sufficiently. Missing nt downstream in the genome.  
uid:13|AF221910.1: Sequence cannot be extended sufficiently by unalined portion of query. THIS IS PROBABLY FRAGMENT! Trimmed downstream.

### Hit: AF221910.1

AF221910.1 Typhlonectes natans telomerase RNA gene, sequence

```
?

```
This is BLAST alignment as read from the input file
```

Score = 54.0 bits (50.0), Expect = 3.95E-02
 Identities = 33/37 (89%), Gaps = 0/37 (0%)
 Strand = Plus/Plus
Query  93 CTCGCTGTTTTCTTGGCTAACTTTCAGCGAGGTGAGG 129
          |||||||||||  ||||||||||||||||||  ||||    
Sbjct 187 CTCGCTGTTTTGCTGGCTAACTTTCAGCGAGCAGAGG 223
```

Report:

|  |  |
| --- | --- |
| sequence start ?  ``` Start position of the estimated full-length sequence in genome. Start index < end index. ``` : | 108 |
| sequence end ?  ``` End position of the estimated full-length sequence in genome. Start index < end index. ``` : | 554 |
| bit score (CM) ?  ``` The score for aligning estimated full-length sequence to CM model   (computed by RSEARCH -> default,   infered from Rfam or provided by user) ``` : | 57.3 |
| Homology estimate ?  ``` Quick homology estimate:   Not homologous: bit score < 0   Homologous: bit score > 20 and bit score > 0.5 * query length   Uncertain otherwise ``` : | Uncertain |

Estimated full-length sequence:


?

```
Click checkbox to select multiple seuqences.
Fasta header format:
  UID|accession.versionSTRAND start-end
```

>uid:14|AF221910.1fw 108-554
GCAUUGUCAUUCCGGAGAGGGGCGUUUUCUUCUUUCUCUAACCCUAAUGUAUUGCUUGGG
CAGCAGCUCUCUCGGUAUGCUCGCUGUUUUGCUGGCUAACUUUCAGCGAGCAGAGGGCGA
AGUCGAAUUUCGGACAAGGGAGAAAAAAUGUUAGCCGGGGAACGCCCCUUUCCCGAAUGC
CCGUGCCGUCCUUCCUCUCCUCUGGGCCCGUUGGCGAUAGCCCCCGGCCUCCCUCGCUCC
UUAGGCCGCGGUCAGUCCGGGCCCUUGCUCUGGCAUGAGGUCCAUUGCUGCCGCGAAGAG
UUCGUCUCUGUCAGCCUUGAGGUGGCCAGCAUGGAAUUGGCGGGCUUGUGGCGGCGCGCG
GGAAAAAGCAAAUGGUGAACUAGGUGCGUGUUGUGGUCGUGUUCCUGAAGCGUGGAAUGU
GCGAUCUGUGCGCUAGUUCAUACACAC

C-A-r-Rc


?

```
Visualisation of predicted secondary structure.
To save the image:
  Right click on the image -> Save Image as.
```


centroid


?

```
Visualisation of predicted secondary structure.
To save the image:
  Right click on the image -> Save Image as.
```


fq-sub


?

```
Visualisation of predicted secondary structure.
To save the image:
  Right click on the image -> Save Image as.
```

Load Sequence viewer

TurboFold: Number of sequences is less then required. n=2 (4)  
uid:14|AF221910.1: Sequence cannot be extended sufficiently by unalined portion of query. THIS IS PROBABLY FRAGMENT! Trimmed downstream.  
uid:14|AF221910.1: Sequence cannot be extended sufficiently. Missing nt downstream in the genome.

### Hit: AF221939.1

AF221939.1 Felis catus telomerase RNA gene, sequence

```
?

```
This is BLAST alignment as read from the input file
```

Score = 67.0 bits (61.7), Expect = 2.18E-05
 Identities = 71/94 (76%), Gaps = 4/94 (4%)
 Strand = Plus/Plus
Query 318 GGCCGCGATCAGCCCGGGCTTTCCCTACTTTGGGGCCCAATGCTGTCGCGAAGAGTTCGGCTCTGTCAGC 387
          ||||||| || |||||||  ||| |  |   ||  |||| ||| ||||||||||||| ||||||||||||    
Sbjct 351 GGCCGCGGTCGGCCCGGGGCTTCTC--CGGAGGCACCCATTGCCGTCGCGAAGAGTTGGGCTCTGTCAGC 418

Query 388 CCGGCTGGGTCCGGGTGGGGGCCG 411
          |  || ||  ||  | ||||||||    
Sbjct 419 C--GCGGGTCCCTTGGGGGGGCCG 440
```

Report:

|  |  |
| --- | --- |
| sequence start ?  ``` Start position of the estimated full-length sequence in genome. Start index < end index. ``` : | 40 |
| sequence end ?  ``` End position of the estimated full-length sequence in genome. Start index < end index. ``` : | 544 |
| bit score (CM) ?  ``` The score for aligning estimated full-length sequence to CM model   (computed by RSEARCH -> default,   infered from Rfam or provided by user) ``` : | 28.42 |
| Homology estimate ?  ``` Quick homology estimate:   Not homologous: bit score < 0   Homologous: bit score > 20 and bit score > 0.5 * query length   Uncertain otherwise ``` : | Uncertain |

Estimated full-length sequence:


?

```
Click checkbox to select multiple seuqences.
Fasta header format:
  UID|accession.versionSTRAND start-end
```

>uid:15|AF221939.1fw 40-544
GCCAAUCGGCGCGUACGGCGGCCGCUGUCUUUAUAGGGAGCCGCGGCGUUUUGCACCUCG
GGUUGUGGAGGGUGGGCCUGGGAGGGGAAGCGGUCAGUUUUUGUCUAACCCUAACUGAGA
AGGGCGUAGGCGCCGCGCUUUUGUUUCCCGCACGCUGUUUUUUUCGCUGACUUUCAGCGG
GCGGAAAAGCCUCGGCCUACCGCCGUCCACCGUACAGUUUGGAGCAAACAAAAAAUGUCA
GCUGCUGACUUGCUCGCCCCUCCCAGGACCCUGCGGUGGCUCGCCUCCUUAGCCCCCGCG
UCCCGCCUAGAGGCCGCGGUCGGCCCGGGGCUUCUCCGGAGGCACCCAUUGCCGUCGCGA
AGAGUUGGGCUCUGUCAGCCGCGGGUCCCUUGGGGGGGCCGAGGGCGAGGCUCUGACCGC
AGGGAGAGAAACGGGAGCAGGUCCCCGCGCGCGGUGCGCUUCCCUGAGCUGUGGGACUUG
CACCCGGGACUGGGCUCAGACACAU

C-A-r-Rc


?

```
Visualisation of predicted secondary structure.
To save the image:
  Right click on the image -> Save Image as.
```


centroid


?

```
Visualisation of predicted secondary structure.
To save the image:
  Right click on the image -> Save Image as.
```


fq-sub


?

```
Visualisation of predicted secondary structure.
To save the image:
  Right click on the image -> Save Image as.
```

Load Sequence viewer

TurboFold: Number of sequences is less then required. n=2 (4)  
uid:15|AF221939.1: Sequence cannot be extended sufficiently. Missing nt downstream in the genome.  
uid:15|AF221939.1: Sequence cannot be extended sufficiently by unalined portion of query. THIS IS PROBABLY FRAGMENT! Trimmed downstream.

### Hit: AF221927.1

AF221927.1 Herpele squalostoma telomerase RNA gene, sequence

```
?

```
This is BLAST alignment as read from the input file
```

Score = 67.0 bits (61.7), Expect = 2.18E-05
 Identities = 56/70 (80%), Gaps = 2/70 (3%)
 Strand = Plus/Plus
Query 315 CCGGGCCGCGATCAGCCCGGGCTTT--CCCTACTTTGGGGCCCAATGCTGTCGCGAAGAGTTCGGCTCTG 382
          || ||||||| |||||||||||  |  | || ||  || | ||||||||| ||||||||||||| |||||    
Sbjct 349 CCAGGCCGCGGTCAGCCCGGGCCCTGGCTCTGCTAGGGCGTCCAATGCTGCCGCGAAGAGTTCGTCTCTG 418
```

Report:

|  |  |
| --- | --- |
| sequence start ?  ``` Start position of the estimated full-length sequence in genome. Start index < end index. ``` : | 28 |
| sequence end ?  ``` End position of the estimated full-length sequence in genome. Start index < end index. ``` : | 554 |
| bit score (CM) ?  ``` The score for aligning estimated full-length sequence to CM model   (computed by RSEARCH -> default,   infered from Rfam or provided by user) ``` : | 14.05 |
| Homology estimate ?  ``` Quick homology estimate:   Not homologous: bit score < 0   Homologous: bit score > 20 and bit score > 0.5 * query length   Uncertain otherwise ``` : | Uncertain |

Estimated full-length sequence:


?

```
Click checkbox to select multiple seuqences.
Fasta header format:
  UID|accession.versionSTRAND start-end
```

>uid:16|AF221927.1fw 28-554
AAAUUCUGCGAACCAAUCAGCUUCCAGCUAGAGGGCGCGUGAGUUGGAAUGGGUGUAUAA
AAAGGGGAACCAGCCACAGAACCAUUCUCUCUGGAGCGGGGCGAUUUCUCUUCCUUCUAA
CCCUAAUGCGGUGCUUAGGCGACAGUUCUCCGGGUGCGCUCGCUGUUUUUCUGGCUAACU
UUCAGCGAGCGGAGAGAGCGAAGCCGUGGUUUACGACGAUAGAGGAGAAAAAAUGUUAGC
UGGGGAACGUCCCUUUCCCGAGAGCCCGCGCCAUCCUCUUCCUCGGGCCCAUUGGCGAUA
GCCCCCGGCCUCCCUCGCUCACCAGGCCGCGGUCAGCCCGGGCCCUGGCUCUGCUAGGGC
GUCCAAUGCUGCCGCGAAGAGUUCGUCUCUGAUUGCCUUGAGGUAGCCGGGCUCGAAUAG
GUGGGCUCGCGGCGAUGCGCAGGAAAGAGCAAAACAGUGAACUGGGCGUGUGCUGGGGUC
GCGUCCCUGAAGUGUGGGAUGUGCGAUCUGUCCGCUUGUUCAGACAU

C-A-r-Rc


?

```
Visualisation of predicted secondary structure.
To save the image:
  Right click on the image -> Save Image as.
```


centroid


?

```
Visualisation of predicted secondary structure.
To save the image:
  Right click on the image -> Save Image as.
```


fq-sub


?

```
Visualisation of predicted secondary structure.
To save the image:
  Right click on the image -> Save Image as.
```

Load Sequence viewer

uid:16|AF221927.1: Sequence cannot be extended sufficiently by unalined portion of query. THIS IS PROBABLY FRAGMENT! Trimmed downstream.  
TurboFold: Number of sequences is less then required. n=2 (4)  
uid:16|AF221927.1: Sequence cannot be extended sufficiently. Missing nt downstream in the genome.

### Hit: AF221927.1

AF221927.1 Herpele squalostoma telomerase RNA gene, sequence

```
?

```
This is BLAST alignment as read from the input file
```

Score = 52.0 bits (48.2), Expect = 1.38E-01
 Identities = 29/31 (94%), Gaps = 0/31 (0%)
 Strand = Plus/Plus
Query  93 CTCGCTGTTTTCTTGGCTAACTTTCAGCGAG 123
          |||||||||||  ||||||||||||||||||    
Sbjct 186 CTCGCTGTTTTTCTGGCTAACTTTCAGCGAG 216
```

Report:

|  |  |
| --- | --- |
| sequence start ?  ``` Start position of the estimated full-length sequence in genome. Start index < end index. ``` : | 113 |
| sequence end ?  ``` End position of the estimated full-length sequence in genome. Start index < end index. ``` : | 556 |
| bit score (CM) ?  ``` The score for aligning estimated full-length sequence to CM model   (computed by RSEARCH -> default,   infered from Rfam or provided by user) ``` : | 50.56 |
| Homology estimate ?  ``` Quick homology estimate:   Not homologous: bit score < 0   Homologous: bit score > 20 and bit score > 0.5 * query length   Uncertain otherwise ``` : | Uncertain |

Estimated full-length sequence:


?

```
Click checkbox to select multiple seuqences.
Fasta header format:
  UID|accession.versionSTRAND start-end
```

>uid:17|AF221927.1fw 113-556
UCUCUCUGGAGCGGGGCGAUUUCUCUUCCUUCUAACCCUAAUGCGGUGCUUAGGCGACAG
UUCUCCGGGUGCGCUCGCUGUUUUUCUGGCUAACUUUCAGCGAGCGGAGAGAGCGAAGCC
GUGGUUUACGACGAUAGAGGAGAAAAAAUGUUAGCUGGGGAACGUCCCUUUCCCGAGAGC
CCGCGCCAUCCUCUUCCUCGGGCCCAUUGGCGAUAGCCCCCGGCCUCCCUCGCUCACCAG
GCCGCGGUCAGCCCGGGCCCUGGCUCUGCUAGGGCGUCCAAUGCUGCCGCGAAGAGUUCG
UCUCUGAUUGCCUUGAGGUAGCCGGGCUCGAAUAGGUGGGCUCGCGGCGAUGCGCAGGAA
AGAGCAAAACAGUGAACUGGGCGUGUGCUGGGGUCGCGUCCCUGAAGUGUGGGAUGUGCG
AUCUGUCCGCUUGUUCAGACAUUG

C-A-r-Rc


?

```
Visualisation of predicted secondary structure.
To save the image:
  Right click on the image -> Save Image as.
```


centroid


?

```
Visualisation of predicted secondary structure.
To save the image:
  Right click on the image -> Save Image as.
```


fq-sub


?

```
Visualisation of predicted secondary structure.
To save the image:
  Right click on the image -> Save Image as.
```

Load Sequence viewer

uid:17|AF221927.1: Sequence cannot be extended sufficiently by unalined portion of query. THIS IS PROBABLY FRAGMENT! Trimmed downstream.  
uid:17|AF221927.1: Sequence cannot be extended sufficiently. Missing nt downstream in the genome.  
TurboFold: Number of sequences is less then required. n=2 (4)

### Hit: XR\_334786.3

XR\_334786.3 PREDICTED: Tupaia chinensis uncharacterized LOC102488102 (LOC102488102), ncRNA

```
?

```
This is BLAST alignment as read from the input file
```

Score = 65.0 bits (59.9), Expect = 7.62E-05
 Identities = 72/95 (76%), Gaps = 5/95 (5%)
 Strand = Plus/Plus
Query 318 GGCCGCGATCAGCCCGGGCTTTCCCTACTTTGGGGCCCAATGCTGTCGCGAAGAGTTCGGCTCTGTCAGC 387
          ||||||| || |||||||  ||| |  |   || ||||| ||| | ||||||||||| ||||||||||||    
Sbjct 305 GGCCGCGGTCGGCCCGGGGCTTCTC--CGGAGGTGCCCATTGCCGCCGCGAAGAGTTAGGCTCTGTCAGC 372

Query 388 CCGGCTGGGTCCGGGTGGGGGCCGG 412
          |  || || ||  || |||| ||||    
Sbjct 373 C--GCGGGCTCTCGG-GGGGCCCGG 394
```

Report:

|  |  |
| --- | --- |
| sequence start ?  ``` Start position of the estimated full-length sequence in genome. Start index < end index. ``` : | 1 |
| sequence end ?  ``` End position of the estimated full-length sequence in genome. Start index < end index. ``` : | 550 |
| bit score (CM) ?  ``` The score for aligning estimated full-length sequence to CM model   (computed by RSEARCH -> default,   infered from Rfam or provided by user) ``` : | 4.44 |
| Homology estimate ?  ``` Quick homology estimate:   Not homologous: bit score < 0   Homologous: bit score > 20 and bit score > 0.5 * query length   Uncertain otherwise ``` : | Uncertain |

Estimated full-length sequence:


?

```
Click checkbox to select multiple seuqences.
Fasta header format:
  UID|accession.versionSTRAND start-end
```

>uid:18|XR\_334786.3fw 1-550
UCCCCUUUAUAAGGACACCGGGCAGGCGGCGCGGAGGGAGUGGAGGGUGGGCCCGGGACG
GGCGCGGACCCGGUAAUCUAACCCUGACUCGCAAGAGGCGUAGGCGCCGUGCUUUUGCUC
CCCGCGCGCUGUUUUUCUCGCUGACUUUCAGCGUUCGGAAAAGCCCUGUGGCCUAUCGCC
AUCCACCAUCCUUUCUGGAACAAACAGACAAAAAAAAAAAAAUGUCAGCUGCUGGUCCGU
UCGCUCCUCCCGGGCCCUGCGGUGGCCGCCAGCCCGCCCUCUCAUCCCCCGCGUCCCGCC
UAGAGGCCGCGGUCGGCCCGGGGCUUCUCCGGAGGUGCCCAUUGCCGCCGCGAAGAGUUA
GGCUCUGUCAGCCGCGGGCUCUCGGGGGGCCCGGGGCGAGGCUUAGGCCUCGUGGCCGCA
GGGACAGCAACGGAGCGGGUCCCCGCGAGCCUGUGCGCGUCCCUGAGCCGUGGGACUUGC
ACCGGGGACUUUGCUCGGACAAUCUGCAGCCGGCUCGGAGACGGCCCCGGAAGUGUGCUU
UCACUGGACC

C-A-r-Rc


?

```
Visualisation of predicted secondary structure.
To save the image:
  Right click on the image -> Save Image as.
```


centroid


?

```
Visualisation of predicted secondary structure.
To save the image:
  Right click on the image -> Save Image as.
```


fq-sub


?

```
Visualisation of predicted secondary structure.
To save the image:
  Right click on the image -> Save Image as.
```

Load Sequence viewer

TurboFold: Number of sequences is less then required. n=2 (4)  
uid:18|XR\_334786.3: Sequence cannot be extended sufficiently by unaligned portion of query. THIS IS PROBABLY FRAGMENT! Trimmed upstream.  
uid:18|XR\_334786.3: Sequence cannot be extended sufficiently. Missing -42 nt upstream in the genome.

### Hit: NR\_033817.1

NR\_033817.1 Callithrix jacchus telomerase RNA component (TERC), telomerase RNA

```
?

```
This is BLAST alignment as read from the input file
```

Score = 65.0 bits (59.9), Expect = 7.62E-05
 Identities = 66/86 (77%), Gaps = 3/86 (3%)
 Strand = Plus/Plus
Query 318 GGCCGCGATCAGCCCGGGCTTTCCCTACTTTGGGGCCCAATGCTGTCGCGAAGAGTTCGGCTCTGTCAGC 387
          ||||||| || |||||||  ||| |  |   || ||||| ||| | ||||||||||| ||||||||||||    
Sbjct 252 GGCCGCGGTCGGCCCGGGGCTTCTC--CGGAGGCGCCCATTGCCGCCGCGAAGAGTTGGGCTCTGTCAGC 319

Query 388 C-CGGCTGGGTCCGGG 402
          | ||| ||  || |||    
Sbjct 320 CGCGGGTGTCTCGGGG 335
```

Report:

|  |  |
| --- | --- |
| sequence start ?  ``` Start position of the estimated full-length sequence in genome. Start index < end index. ``` : | 1 |
| sequence end ?  ``` End position of the estimated full-length sequence in genome. Start index < end index. ``` : | 450 |
| bit score (CM) ?  ``` The score for aligning estimated full-length sequence to CM model   (computed by RSEARCH -> default,   infered from Rfam or provided by user) ``` : | 19.07 |
| Homology estimate ?  ``` Quick homology estimate:   Not homologous: bit score < 0   Homologous: bit score > 20 and bit score > 0.5 * query length   Uncertain otherwise ``` : | Uncertain |

Estimated full-length sequence:


?

```
Click checkbox to select multiple seuqences.
Fasta header format:
  UID|accession.versionSTRAND start-end
```

>uid:19|NR\_033817.1fw 1-450
GGGUUGCGGAGGGUGGGCCUGGGAGGGUGGUGGCCAUUUUUUUUCUAACCCUAACUGAGA
CGGGCGUAGGCGCCGUGCUUUUGCUCCCCGCGCGCUGUUUUUCUCGCUGACUUUCAGCGU
UCGGAAAAGCCUCGGCCUACCGCCUUCCACCGUUCAUUCUGGAGCAAACAAAAAAUGUCA
GCCGCUGGCCAGUUCGCCCCUCCCGGGGACCUGCGGCGGGUAGCCUGCCUAGCCCCCGAA
CCCCGCCUGGAGGCCGCGGUCGGCCCGGGGCUUCUCCGGAGGCGCCCAUUGCCGCCGCGA
AGAGUUGGGCUCUGUCAGCCGCGGGUGUCUCGGGGGCGAGGGUGAGGCUCAGGCCUUUCA
GGCCGCAGGAAGAGGAACGGAGCGAGUUCCCACGCACGGCGCGCUUCCCUGAGCUGUGGG
ACGUGCACCCAGAACUCGGCUCACACAUGC

C-A-r-Rc


?

```
Visualisation of predicted secondary structure.
To save the image:
  Right click on the image -> Save Image as.
```


centroid


?

```
Visualisation of predicted secondary structure.
To save the image:
  Right click on the image -> Save Image as.
```


fq-sub


?

```
Visualisation of predicted secondary structure.
To save the image:
  Right click on the image -> Save Image as.
```

Load Sequence viewer

TurboFold: Number of sequences is less then required. n=2 (4)  
uid:19|NR\_033817.1: Sequence cannot be extended sufficiently by unaligned portion of query. THIS IS PROBABLY FRAGMENT! Trimmed upstream.  
uid:19|NR\_033817.1: Sequence cannot be extended sufficiently. Missing -95 nt upstream in the genome.  
uid:19|NR\_033817.1: Sequence cannot be extended sufficiently. Missing nt downstream in the genome.

### Hit: EU486823.1

EU486823.1 Equus burchellii telomerase RNA gene, complete sequence

```
?

```
This is BLAST alignment as read from the input file
```

Score = 65.0 bits (59.9), Expect = 7.62E-05
 Identities = 66/86 (77%), Gaps = 3/86 (3%)
 Strand = Plus/Plus
Query 318 GGCCGCGATCAGCCCGGGCTTTCCCTACTTTGGGGCCCAATGCTGTCGCGAAGAGTTCGGCTCTGTCAGC 387
          ||||||| || |||||||  ||| |  |   || ||||| ||| | ||||||||||| ||||||||||||    
Sbjct 253 GGCCGCGGTCGGCCCGGGGCTTCTC--CGGAGGCGCCCAGTGCCGCCGCGAAGAGTTGGGCTCTGTCAGC 320

Query 388 C-CGGCTGGGTCCGGG 402
          | ||| ||  || |||    
Sbjct 321 CGCGGGTGCCTCGGGG 336
```

Report:

|  |  |
| --- | --- |
| sequence start ?  ``` Start position of the estimated full-length sequence in genome. Start index < end index. ``` : | 1 |
| sequence end ?  ``` End position of the estimated full-length sequence in genome. Start index < end index. ``` : | 443 |
| bit score (CM) ?  ``` The score for aligning estimated full-length sequence to CM model   (computed by RSEARCH -> default,   infered from Rfam or provided by user) ``` : | 29.4 |
| Homology estimate ?  ``` Quick homology estimate:   Not homologous: bit score < 0   Homologous: bit score > 20 and bit score > 0.5 * query length   Uncertain otherwise ``` : | Uncertain |

Estimated full-length sequence:


?

```
Click checkbox to select multiple seuqences.
Fasta header format:
  UID|accession.versionSTRAND start-end
```

>uid:20|EU486823.1fw 1-443
GGGUGGGGGAGAGUGGGUCUGGGCGGGGCGGCGGUCACGUUUUGUCUAACCCUAACUGAG
CUGGGCGGAGGCGCCGCGCUUUUGCUCCCCGCGCGCUGUUUUUCUCGCUGACUUUCAGCG
GGCGGAAAAGCCUCGGUCUACCGCCACUUACCAUCCAGUCUGGAGCAAACAAAAAAUGUC
AGCCGCUGGCUCGCUCGCCCCUCCCGGGACCCUGCGACGGCUCGCCCGCCCAGCCCCCGC
GCCCCGCCUGGAGGCCGCGGUCGGCCCGGGGCUUCUCCGGAGGCGCCCAGUGCCGCCGCG
AAGAGUUGGGCUCUGUCAGCCGCGGGUGCCUCGGGGGCCAGGGACGAGGCUCUGGCCGCA
GGGAGAGGAACGGAGCGGGUCCCCGCGCGCGGUGCGCUUCCCUGAGCUGUGGGACGUGCA
CCCGGGACUCGGCUCAAACACGU

C-A-r-Rc


?

```
Visualisation of predicted secondary structure.
To save the image:
  Right click on the image -> Save Image as.
```


centroid


?

```
Visualisation of predicted secondary structure.
To save the image:
  Right click on the image -> Save Image as.
```


fq-sub


?

```
Visualisation of predicted secondary structure.
To save the image:
  Right click on the image -> Save Image as.
```

Load Sequence viewer

uid:20|EU486823.1: Sequence cannot be extended sufficiently. Missing nt downstream in the genome.  
TurboFold: Number of sequences is less then required. n=2 (4)  
uid:20|EU486823.1: Sequence cannot be extended sufficiently. Missing -94 nt upstream in the genome.  
uid:20|EU486823.1: Sequence cannot be extended sufficiently by unaligned portion of query. THIS IS PROBABLY FRAGMENT! Trimmed upstream.

### Hit: AF221931.1

AF221931.1 Mustela putorius furo telomerase RNA gene, sequence

```
?

```
This is BLAST alignment as read from the input file
```

Score = 64.0 bits (59.0), Expect = 7.62E-05
 Identities = 56/71 (79%), Gaps = 2/71 (3%)
 Strand = Plus/Plus
Query 318 GGCCGCGATCAGCCCGGGCTTTCCCTACTTTGGGGCCCAATGCTGTCGCGAAGAGTTCGGCTCTGTCAGC 387
          ||||||| || |||||||  ||| |  |   ||  |||| ||| ||||||||||||| ||||||||||||    
Sbjct 351 GGCCGCGGTCGGCCCGGGGCTTCTC--CGGAGGCACCCATTGCCGTCGCGAAGAGTTGGGCTCTGTCAGC 418

Query 388 C 388
          |    
Sbjct 419 C 419
```

Report:

|  |  |
| --- | --- |
| sequence start ?  ``` Start position of the estimated full-length sequence in genome. Start index < end index. ``` : | 30 |
| sequence end ?  ``` End position of the estimated full-length sequence in genome. Start index < end index. ``` : | 541 |
| bit score (CM) ?  ``` The score for aligning estimated full-length sequence to CM model   (computed by RSEARCH -> default,   infered from Rfam or provided by user) ``` : | 10.14 |
| Homology estimate ?  ``` Quick homology estimate:   Not homologous: bit score < 0   Homologous: bit score > 20 and bit score > 0.5 * query length   Uncertain otherwise ``` : | Uncertain |

Estimated full-length sequence:


?

```
Click checkbox to select multiple seuqences.
Fasta header format:
  UID|accession.versionSTRAND start-end
```

>uid:21|AF221931.1fw 30-541
AGGGGCUCCGGCCAAUCGGCGCGGGCCGCGGCCGCUCCCUUUAUAGGGAGACGCGGCCGG
GUGCAGUUCGGGUUGCGGAGGGUGGGCUCGGGAGGGGUGGCGGUCAUUUUCUGUCUAACC
CUAACUGAAACGGGCGUAGGCGCUGCGCUUUUGUUCCCCGCACGCUGUUUUUCUCGCUGA
CUUUCAGCGGGCGGAAAAGCCUUGGCCUACUGCCACACACCAUCCAGUUUGGAGCAAACA
AAAAAUGUCAGCGGCUGGCCUGCUCGCCCCUCCCGGGAGCCUGCGGCGACUCGCCCGCUU
AGCCCCCGCAUCCCGCCUGGAGGCCGCGGUCGGCCCGGGGCUUCUCCGGAGGCACCCAUU
GCCGUCGCGAAGAGUUGGGCUCUGUCAGCCGCGGGACCCUUGGGGGCCAAGGGCGAGGCU
CUGGCCGCAGGGAGAGAAACGGAGCGGGUCCCCUCGCGCGGUGCGCUUCCCUGAGCUGUG
GGACUUGCACCCGGGACUAGGCUCACACACAC

C-A-r-Rc


?

```
Visualisation of predicted secondary structure.
To save the image:
  Right click on the image -> Save Image as.
```


centroid


?

```
Visualisation of predicted secondary structure.
To save the image:
  Right click on the image -> Save Image as.
```


fq-sub


?

```
Visualisation of predicted secondary structure.
To save the image:
  Right click on the image -> Save Image as.
```

Load Sequence viewer

uid:21|AF221931.1: Sequence cannot be extended sufficiently. Missing nt downstream in the genome.  
TurboFold: Number of sequences is less then required. n=2 (4)  
uid:21|AF221931.1: Sequence cannot be extended sufficiently by unalined portion of query. THIS IS PROBABLY FRAGMENT! Trimmed downstream.

### Hit: AF221920.1

AF221920.1 Sus scrofa telomerase RNA gene, sequence

```
?

```
This is BLAST alignment as read from the input file
```

Score = 64.0 bits (59.0), Expect = 7.62E-05
 Identities = 56/71 (79%), Gaps = 2/71 (3%)
 Strand = Plus/Plus
Query 318 GGCCGCGATCAGCCCGGGCTTTCCCTACTTTGGGGCCCAATGCTGTCGCGAAGAGTTCGGCTCTGTCAGC 387
          ||||||| || |||||||  ||| |  |   || ||||| ||| | ||||||||||| ||||||||||||    
Sbjct 352 GGCCGCGGTCGGCCCGGGGCTTCTC--CGGAGGCGCCCATTGCCGCCGCGAAGAGTTGGGCTCTGTCAGC 419

Query 388 C 388
          |    
Sbjct 420 C 420
```

Report:

|  |  |
| --- | --- |
| sequence start ?  ``` Start position of the estimated full-length sequence in genome. Start index < end index. ``` : | 40 |
| sequence end ?  ``` End position of the estimated full-length sequence in genome. Start index < end index. ``` : | 541 |
| bit score (CM) ?  ``` The score for aligning estimated full-length sequence to CM model   (computed by RSEARCH -> default,   infered from Rfam or provided by user) ``` : | 17.26 |
| Homology estimate ?  ``` Quick homology estimate:   Not homologous: bit score < 0   Homologous: bit score > 20 and bit score > 0.5 * query length   Uncertain otherwise ``` : | Uncertain |

Estimated full-length sequence:


?

```
Click checkbox to select multiple seuqences.
Fasta header format:
  UID|accession.versionSTRAND start-end
```

>uid:22|AF221920.1fw 40-541
GCCAAUCGGCGCGGUCGGCGGCCGCUCCUUUUAUAGGGAGACGCAUCCGCCUGAUCGGCG
GGUUGCGGAGGGUGGGCCCAGGAGCGGUGGCGGCCAUUUUUUAAGUCUAACCCUAACUGA
AAGAGGCGUAGGCGCUGCGCUUUUGCUUCACGCGCGCUGUUUUUCUCGCUGACUUUCAGC
GGGCGGAAAAGCCUCGGCCUACCGCCAUCCACCAUCCAGUCUGAAACAAACAAAAAAUGU
CAGCCUCUGGCUCGCUCACUGCUCCCGGGAACCUGCGGUGGUUCGCCCGCCCAGCCCCCG
CGCCCCGCCUGAGGCCGCGGUCGGCCCGGGGCUUCUCCGGAGGCGCCCAUUGCCGCCGCG
AAGAGUUGGGCUCUGUCAGCCGCGGGUCCCUCGGGGGCCAAGGCGAGGCUCUGACCGCAG
GGAAAGGAACGGAGUUGGUCCCCGCGCGCUGUGCGCUUCCCUAAGCUGUGGGCUGUGCAC
CUGGGACUCGGCUCAGACACUU

C-A-r-Rc


?

```
Visualisation of predicted secondary structure.
To save the image:
  Right click on the image -> Save Image as.
```


centroid


?

```
Visualisation of predicted secondary structure.
To save the image:
  Right click on the image -> Save Image as.
```


fq-sub


?

```
Visualisation of predicted secondary structure.
To save the image:
  Right click on the image -> Save Image as.
```

Load Sequence viewer

TurboFold: Number of sequences is less then required. n=2 (4)  
uid:22|AF221920.1: Sequence cannot be extended sufficiently. Missing nt downstream in the genome.  
uid:22|AF221920.1: Sequence cannot be extended sufficiently by unalined portion of query. THIS IS PROBABLY FRAGMENT! Trimmed downstream.

### Hit: AF221917.1

AF221917.1 Procyon lotor telomerase RNA gene, sequence

```
?

```
This is BLAST alignment as read from the input file
```

Score = 64.0 bits (59.0), Expect = 7.62E-05
 Identities = 56/71 (79%), Gaps = 2/71 (3%)
 Strand = Plus/Plus
Query 318 GGCCGCGATCAGCCCGGGCTTTCCCTACTTTGGGGCCCAATGCTGTCGCGAAGAGTTCGGCTCTGTCAGC 387
          ||||||| || |||||||  ||| |  |   ||  |||| ||| ||||||||||||| ||||||||||||    
Sbjct 351 GGCCGCGGTCGGCCCGGGGCTTCTC--CGGAGGCACCCATTGCCGTCGCGAAGAGTTGGGCTCTGTCAGC 418

Query 388 C 388
          |    
Sbjct 419 C 419
```

Report:

|  |  |
| --- | --- |
| sequence start ?  ``` Start position of the estimated full-length sequence in genome. Start index < end index. ``` : | 40 |
| sequence end ?  ``` End position of the estimated full-length sequence in genome. Start index < end index. ``` : | 542 |
| bit score (CM) ?  ``` The score for aligning estimated full-length sequence to CM model   (computed by RSEARCH -> default,   infered from Rfam or provided by user) ``` : | 31.45 |
| Homology estimate ?  ``` Quick homology estimate:   Not homologous: bit score < 0   Homologous: bit score > 20 and bit score > 0.5 * query length   Uncertain otherwise ``` : | Uncertain |

Estimated full-length sequence:


?

```
Click checkbox to select multiple seuqences.
Fasta header format:
  UID|accession.versionSTRAND start-end
```

>uid:23|AF221917.1fw 40-542
GCCAAUCGGCGCGGGCCGCGGCCGCUCCCUUUAUAGGGAGACGCGGCGGGCUGUAGCUCG
GGUUGCGGAGGGUGGGCCUGGGAGGGGUGGCGGUCGUUUUAUGUCUAACCCUAACUGAGA
AGGGCAUAGGCGCUGCGCUUUUGUUCCCCGCACGCUGUUUUUCUCGCUGACUUUCAGCGG
GCGGAAAAGCCUCGGCCUACUGCCAUCCACCAUCCAGUUUGGAGCAAACAAAAAAUGUCA
GCCGCCGGCCUGCUCGCCCCUCCCGGGAUCCUGCGGUGGCUCGCCCGCUUAGCCCCCGCG
UCCCGCCUGGAGGCCGCGGUCGGCCCGGGGCUUCUCCGGAGGCACCCAUUGCCGUCGCGA
AGAGUUGGGCUCUGUCAGCCGCGGGACCUUUGGGGGCCAAGGGCGAGGCUCUGGCCGCAG
GGAGAGAAAACGGAGCGGGUCCCCUUGCGCGGUGCGCUUCCCUGAGCUGUGGGACUUGCA
CCCGGGACUAGGCUCACACACAU

C-A-r-Rc


?

```
Visualisation of predicted secondary structure.
To save the image:
  Right click on the image -> Save Image as.
```


centroid


?

```
Visualisation of predicted secondary structure.
To save the image:
  Right click on the image -> Save Image as.
```


fq-sub


?

```
Visualisation of predicted secondary structure.
To save the image:
  Right click on the image -> Save Image as.
```

Load Sequence viewer

TurboFold: Number of sequences is less then required. n=2 (4)  
uid:23|AF221917.1: Sequence cannot be extended sufficiently by unalined portion of query. THIS IS PROBABLY FRAGMENT! Trimmed downstream.  
uid:23|AF221917.1: Sequence cannot be extended sufficiently. Missing nt downstream in the genome.

### Hit: AF221912.1

AF221912.1 Tupaia glis belangeri telomerase RNA gene, sequence

```
?

```
This is BLAST alignment as read from the input file
```

Score = 64.0 bits (59.0), Expect = 7.62E-05
 Identities = 56/71 (79%), Gaps = 2/71 (3%)
 Strand = Plus/Plus
Query 318 GGCCGCGATCAGCCCGGGCTTTCCCTACTTTGGGGCCCAATGCTGTCGCGAAGAGTTCGGCTCTGTCAGC 387
          ||||||| || |||||||  ||| |  |   || ||||| ||| | ||||||||||| ||||||||||||    
Sbjct 365 GGCCGCGGTCGGCCCGGGGCTTCTC--CGGAGGTGCCCATTGCCGCCGCGAAGAGTTAGGCTCTGTCAGC 432

Query 388 C 388
          |    
Sbjct 433 C 433
```

Report:

|  |  |
| --- | --- |
| sequence start ?  ``` Start position of the estimated full-length sequence in genome. Start index < end index. ``` : | 109 |
| sequence end ?  ``` End position of the estimated full-length sequence in genome. Start index < end index. ``` : | 564 |
| bit score (CM) ?  ``` The score for aligning estimated full-length sequence to CM model   (computed by RSEARCH -> default,   infered from Rfam or provided by user) ``` : | 30.73 |
| Homology estimate ?  ``` Quick homology estimate:   Not homologous: bit score < 0   Homologous: bit score > 20 and bit score > 0.5 * query length   Uncertain otherwise ``` : | Uncertain |

Estimated full-length sequence:


?

```
Click checkbox to select multiple seuqences.
Fasta header format:
  UID|accession.versionSTRAND start-end
```

>uid:24|AF221912.1fw 109-564
GUGGGCCCGGGACGGGCGCGGACCCGGUAAUCUAACCCUGACUCACAAGAGGCGUAGGCG
CCGUGCUUUUGCUCCCCGCGCGCUGUUUUUCUCGCUGACUUUCAGCGUUCGGAAAAGCCC
UGUGGCCUAUCGCCAUCCACCAUCCUUUCUGGAACAAACAGACAAAAAAAAAAAUGUCAG
CUGCUGGUCCGUUCGCUCCUCCCGGGCCCUGCGGUGGCCGCCAGCCCGCCCUCUCAUCCC
CCGCGUCCCGCCUAGAGGCCGCGGUCGGCCCGGGGCUUCUCCGGAGGUGCCCAUUGCCGC
CGCGAAGAGUUAGGCUCUGUCAGCCGCGGGCUCUCGGGGGGCCCAGGGCGAGGUUUAGGC
CUCGUGGCCGCAGGGACAGCAACGGAGCGGGUCCCCGCGAGCCUGUGCGCGUCCCUGAGC
CGUGGGACUUGCACCGGGGACUUUGCUCGGACAAUC

C-A-r-Rc


?

```
Visualisation of predicted secondary structure.
To save the image:
  Right click on the image -> Save Image as.
```


centroid


?

```
Visualisation of predicted secondary structure.
To save the image:
  Right click on the image -> Save Image as.
```


fq-sub


?

```
Visualisation of predicted secondary structure.
To save the image:
  Right click on the image -> Save Image as.
```

Load Sequence viewer

uid:24|AF221912.1: Sequence cannot be extended sufficiently. Missing nt downstream in the genome.  
TurboFold: Number of sequences is less then required. n=2 (4)  
uid:24|AF221912.1: Sequence cannot be extended sufficiently by unalined portion of query. THIS IS PROBABLY FRAGMENT! Trimmed downstream.

### Hit: AF221906.1

AF221906.1 Dasypus novemcinctus telomerase RNA gene, sequence

```
?

```
This is BLAST alignment as read from the input file
```

Score = 64.0 bits (59.0), Expect = 7.62E-05
 Identities = 56/71 (79%), Gaps = 2/71 (3%)
 Strand = Plus/Plus
Query 318 GGCCGCGATCAGCCCGGGCTTTCCCTACTTTGGGGCCCAATGCTGTCGCGAAGAGTTCGGCTCTGTCAGC 387
          ||||||| || |||||||  ||| |  |   ||  |||||||| | ||||||||||| ||||||||||||    
Sbjct 348 GGCCGCGGTCCGCCCGGGGCTTCTC--CGGAGGCACCCAATGCAGCCGCGAAGAGTTAGGCTCTGTCAGC 415

Query 388 C 388
          |    
Sbjct 416 C 416
```

Report:

|  |  |
| --- | --- |
| sequence start ?  ``` Start position of the estimated full-length sequence in genome. Start index < end index. ``` : | 38 |
| sequence end ?  ``` End position of the estimated full-length sequence in genome. Start index < end index. ``` : | 545 |
| bit score (CM) ?  ``` The score for aligning estimated full-length sequence to CM model   (computed by RSEARCH -> default,   infered from Rfam or provided by user) ``` : | -2.98 |
| Homology estimate ?  ``` Quick homology estimate:   Not homologous: bit score < 0   Homologous: bit score > 20 and bit score > 0.5 * query length   Uncertain otherwise ``` : | Not homologous |

Estimated full-length sequence:


?

```
Click checkbox to select multiple seuqences.
Fasta header format:
  UID|accession.versionSTRAND start-end
```

>uid:25|AF221906.1fw 38-545
GGCCAAUCCGCGCCGCCGCCUCGUUCCCUUUAUAAGGAGGCUGCGCGCGCCCAGCGGCGG
GUUGCGGAGGGAGGGCCCGGGAGGGGUGAGCGUCCAUUAUCGUCUAACCCUAACUGAGAU
GGGCGUAGGCGCCGCGCUUUUGCUUCCCGCGCGCUGUUUUUCUCGCUGACUUUCAGCGGG
CGGAAAAGCCUCGGCCUACUGCCGUCUACUGUCGUAUCUGGAGCAAACAAAAAAUGUCAG
CCGCUGGUCCGCUCGCCCCAUCCGGGAACCUGCGGUGGCUCGUCCGCCCUGCCCCCGCGC
UCCGCCUAGAGGCCGCGGUCCGCCCGGGGCUUCUCCGGAGGCACCCAAUGCAGCCGCGAA
GAGUUAGGCUCUGUCAGCCGCGGAUCCCGCGGGGGCCAAGGGUGAGGCUUAGGCCGCGGG
CCGCAGGAAGAAAAACCGAGCGAGUUCUCACGCGCGGUGCGCUUCCCUGAGCUGUGGGUC
GUGCGCCUGGGACUCGGCUCAGACACGU

C-A-r-Rc


?

```
Visualisation of predicted secondary structure.
To save the image:
  Right click on the image -> Save Image as.
```


centroid


?

```
Visualisation of predicted secondary structure.
To save the image:
  Right click on the image -> Save Image as.
```


fq-sub


?

```
Visualisation of predicted secondary structure.
To save the image:
  Right click on the image -> Save Image as.
```

Load Sequence viewer

TurboFold: Number of sequences is less then required. n=2 (4)  
uid:25|AF221906.1: Sequence cannot be extended sufficiently by unalined portion of query. THIS IS PROBABLY FRAGMENT! Trimmed downstream.  
uid:25|AF221906.1: Sequence cannot be extended sufficiently. Missing nt downstream in the genome.

### Hit: XR\_003858639.1

XR\_003858639.1 PREDICTED: Rhinatrema bivittatum uncharacterized LOC115098914 (LOC115098914), transcript variant X3, ncRNA

```
?

```
This is BLAST alignment as read from the input file
```

Score = 63.0 bits (58.1), Expect = 2.66E-04
 Identities = 60/76 (79%), Gaps = 6/76 (8%)
 Strand = Plus/Plus
Query 314 TCCG-GGCCGCGATCAGCCCGGGCTTTCCCTACTTTGGGGCCCAATGCTGTCGCGAAGAGTTCGGCTCTG 382
          |||| ||||||| |||||||||||   |||  |   | || ||| ||||| ||||||||||||| |||||    
Sbjct 293 TCCGAGGCCGCGGTCAGCCCGGGC---CCCAGCC--GCGGTCCACTGCTGCCGCGAAGAGTTCGTCTCTG 357

Query 383 TCAGCC 388
          | ||||    
Sbjct 358 TTAGCC 363
```

Report:

|  |  |
| --- | --- |
| sequence start ?  ``` Start position of the estimated full-length sequence in genome. Start index < end index. ``` : | 1 |
| sequence end ?  ``` End position of the estimated full-length sequence in genome. Start index < end index. ``` : | 550 |
| bit score (CM) ?  ``` The score for aligning estimated full-length sequence to CM model   (computed by RSEARCH -> default,   infered from Rfam or provided by user) ``` : | 16.01 |
| Homology estimate ?  ``` Quick homology estimate:   Not homologous: bit score < 0   Homologous: bit score > 20 and bit score > 0.5 * query length   Uncertain otherwise ``` : | Uncertain |

Estimated full-length sequence:


?

```
Click checkbox to select multiple seuqences.
Fasta header format:
  UID|accession.versionSTRAND start-end
```

>uid:26|XR\_003858639.1fw 1-550
UGCUUUCCGAGAGGCGCGUGGGGGGAGCGUUCUCUGCUUUCUAACCCUAACGUGGUGUCC
GGACGCCCGUCGGCGCUUUCACGCGCACUCGCUGUUUUUUCCGGGUAACUUUCAGCGAGC
AGAAAGCUUAUAGCAAGCUUUGCCACGACAAGGGCGAACGCAAAAAAAAAAAAAAAAAAA
AGUUAUCCGGAGAACGUCCCUCCCUUUCCCUAGAGCCCGCGCUGUCUUUCCUUCGCCUGC
GGGGCCUGUCCACGGUAGUCGCCCCCCCCCCCCUCCCCCGGCCUUCCUCGCUUCCGAGGC
CGCGGUCAGCCCGGGCCCCAGCCGCGGUCCACUGCUGCCGCGAAGAGUUCGUCUCUGUUA
GCCUUGAGGCGGCCGGGGUUGAAGUAGGCGGGGCUCGCGGCAGCACGCGGGGAAAAAGCA
AAACGUGAGCUGGGCUCGUGCUAGGGUCGCCUCCCUGAGACGUGGGUCGUGUGAUCUGGG
CCCGAUUCAGACAUUCAAUGCAAUGCAAUGCACGUCUCCAGCAAAUCAAGCCACGCGUCG
UACUUAUUCC

C-A-r-Rc


?

```
Visualisation of predicted secondary structure.
To save the image:
  Right click on the image -> Save Image as.
```


centroid


?

```
Visualisation of predicted secondary structure.
To save the image:
  Right click on the image -> Save Image as.
```


fq-sub


?

```
Visualisation of predicted secondary structure.
To save the image:
  Right click on the image -> Save Image as.
```

Load Sequence viewer

TurboFold: Number of sequences is less then required. n=2 (4)  
uid:26|XR\_003858639.1: Sequence cannot be extended sufficiently. Missing -50 nt upstream in the genome.  
uid:26|XR\_003858639.1: Sequence cannot be extended sufficiently by unaligned portion of query. THIS IS PROBABLY FRAGMENT! Trimmed upstream.

### Hit: XR\_003858638.1

XR\_003858638.1 PREDICTED: Rhinatrema bivittatum uncharacterized LOC115098914 (LOC115098914), transcript variant X2, ncRNA

```
?

```
This is BLAST alignment as read from the input file
```

Score = 63.0 bits (58.1), Expect = 2.66E-04
 Identities = 60/76 (79%), Gaps = 6/76 (8%)
 Strand = Plus/Plus
Query 314 TCCG-GGCCGCGATCAGCCCGGGCTTTCCCTACTTTGGGGCCCAATGCTGTCGCGAAGAGTTCGGCTCTG 382
          |||| ||||||| |||||||||||   |||  |   | || ||| ||||| ||||||||||||| |||||    
Sbjct 293 TCCGAGGCCGCGGTCAGCCCGGGC---CCCAGCC--GCGGTCCACTGCTGCCGCGAAGAGTTCGTCTCTG 357

Query 383 TCAGCC 388
          | ||||    
Sbjct 358 TTAGCC 363
```

Report:

|  |  |
| --- | --- |
| sequence start ?  ``` Start position of the estimated full-length sequence in genome. Start index < end index. ``` : | 1 |
| sequence end ?  ``` End position of the estimated full-length sequence in genome. Start index < end index. ``` : | 550 |
| bit score (CM) ?  ``` The score for aligning estimated full-length sequence to CM model   (computed by RSEARCH -> default,   infered from Rfam or provided by user) ``` : | 16.01 |
| Homology estimate ?  ``` Quick homology estimate:   Not homologous: bit score < 0   Homologous: bit score > 20 and bit score > 0.5 * query length   Uncertain otherwise ``` : | Uncertain |

Estimated full-length sequence:


?

```
Click checkbox to select multiple seuqences.
Fasta header format:
  UID|accession.versionSTRAND start-end
```

>uid:27|XR\_003858638.1fw 1-550
UGCUUUCCGAGAGGCGCGUGGGGGGAGCGUUCUCUGCUUUCUAACCCUAACGUGGUGUCC
GGACGCCCGUCGGCGCUUUCACGCGCACUCGCUGUUUUUUCCGGGUAACUUUCAGCGAGC
AGAAAGCUUAUAGCAAGCUUUGCCACGACAAGGGCGAACGCAAAAAAAAAAAAAAAAAAA
AGUUAUCCGGAGAACGUCCCUCCCUUUCCCUAGAGCCCGCGCUGUCUUUCCUUCGCCUGC
GGGGCCUGUCCACGGUAGUCGCCCCCCCCCCCCUCCCCCGGCCUUCCUCGCUUCCGAGGC
CGCGGUCAGCCCGGGCCCCAGCCGCGGUCCACUGCUGCCGCGAAGAGUUCGUCUCUGUUA
GCCUUGAGGCGGCCGGGGUUGAAGUAGGCGGGGCUCGCGGCAGCACGCGGGGAAAAAGCA
AAACGUGAGCUGGGCUCGUGCUAGGGUCGCCUCCCUGAGACGUGGGUCGUGUGAUCUGGG
CCCGAUUCAGACAUUCAAUGCAAUGCAAUGCACGUCUCCAGCAAAUCAAGCCACGCGUCG
UACUUAUUCC

C-A-r-Rc


?

```
Visualisation of predicted secondary structure.
To save the image:
  Right click on the image -> Save Image as.
```


centroid


?

```
Visualisation of predicted secondary structure.
To save the image:
  Right click on the image -> Save Image as.
```


fq-sub


?

```
Visualisation of predicted secondary structure.
To save the image:
  Right click on the image -> Save Image as.
```

Load Sequence viewer

TurboFold: Number of sequences is less then required. n=2 (4)  
uid:27|XR\_003858638.1: Sequence cannot be extended sufficiently. Missing -50 nt upstream in the genome.  
uid:27|XR\_003858638.1: Sequence cannot be extended sufficiently by unaligned portion of query. THIS IS PROBABLY FRAGMENT! Trimmed upstream.

### Hit: XR\_003858637.1

XR\_003858637.1 PREDICTED: Rhinatrema bivittatum uncharacterized LOC115098914 (LOC115098914), transcript variant X1, ncRNA

```
?

```
This is BLAST alignment as read from the input file
```

Score = 63.0 bits (58.1), Expect = 2.66E-04
 Identities = 60/76 (79%), Gaps = 6/76 (8%)
 Strand = Plus/Plus
Query 314 TCCG-GGCCGCGATCAGCCCGGGCTTTCCCTACTTTGGGGCCCAATGCTGTCGCGAAGAGTTCGGCTCTG 382
          |||| ||||||| |||||||||||   |||  |   | || ||| ||||| ||||||||||||| |||||    
Sbjct 293 TCCGAGGCCGCGGTCAGCCCGGGC---CCCAGCC--GCGGTCCACTGCTGCCGCGAAGAGTTCGTCTCTG 357

Query 383 TCAGCC 388
          | ||||    
Sbjct 358 TTAGCC 363
```

Report:

|  |  |
| --- | --- |
| sequence start ?  ``` Start position of the estimated full-length sequence in genome. Start index < end index. ``` : | 1 |
| sequence end ?  ``` End position of the estimated full-length sequence in genome. Start index < end index. ``` : | 550 |
| bit score (CM) ?  ``` The score for aligning estimated full-length sequence to CM model   (computed by RSEARCH -> default,   infered from Rfam or provided by user) ``` : | 16.01 |
| Homology estimate ?  ``` Quick homology estimate:   Not homologous: bit score < 0   Homologous: bit score > 20 and bit score > 0.5 * query length   Uncertain otherwise ``` : | Uncertain |

Estimated full-length sequence:


?

```
Click checkbox to select multiple seuqences.
Fasta header format:
  UID|accession.versionSTRAND start-end
```

>uid:28|XR\_003858637.1fw 1-550
UGCUUUCCGAGAGGCGCGUGGGGGGAGCGUUCUCUGCUUUCUAACCCUAACGUGGUGUCC
GGACGCCCGUCGGCGCUUUCACGCGCACUCGCUGUUUUUUCCGGGUAACUUUCAGCGAGC
AGAAAGCUUAUAGCAAGCUUUGCCACGACAAGGGCGAACGCAAAAAAAAAAAAAAAAAAA
AGUUAUCCGGAGAACGUCCCUCCCUUUCCCUAGAGCCCGCGCUGUCUUUCCUUCGCCUGC
GGGGCCUGUCCACGGUAGUCGCCCCCCCCCCCCUCCCCCGGCCUUCCUCGCUUCCGAGGC
CGCGGUCAGCCCGGGCCCCAGCCGCGGUCCACUGCUGCCGCGAAGAGUUCGUCUCUGUUA
GCCUUGAGGCGGCCGGGGUUGAAGUAGGCGGGGCUCGCGGCAGCACGCGGGGAAAAAGCA
AAACGUGAGCUGGGCUCGUGCUAGGGUCGCCUCCCUGAGACGUGGGUCGUGUGAUCUGGG
CCCGAUUCAGACAUUCAAUGCAAUGCAAUGCACGUCUCCAGCAAAUCAAGCCACGCGUCG
UACUUAUUCC

C-A-r-Rc


?

```
Visualisation of predicted secondary structure.
To save the image:
  Right click on the image -> Save Image as.
```


centroid


?

```
Visualisation of predicted secondary structure.
To save the image:
  Right click on the image -> Save Image as.
```


fq-sub


?

```
Visualisation of predicted secondary structure.
To save the image:
  Right click on the image -> Save Image as.
```

Load Sequence viewer

TurboFold: Number of sequences is less then required. n=2 (4)  
uid:28|XR\_003858637.1: Sequence cannot be extended sufficiently by unaligned portion of query. THIS IS PROBABLY FRAGMENT! Trimmed upstream.  
uid:28|XR\_003858637.1: Sequence cannot be extended sufficiently. Missing -50 nt upstream in the genome.

### Hit: XM\_016148125.1

XM\_016148125.1 PREDICTED: Rousettus aegyptiacus actin related protein T3 (ACTRT3), mRNA

```
?

```
This is BLAST alignment as read from the input file
```

Score = 60.0 bits (55.4), Expect = 9.28E-04
 Identities = 69/92 (75%), Gaps = 6/92 (7%)
 Strand = Plus/Plus
Query  318 GGCCGCGATCAGCCCGGGCTTTCCCTACTTTGGGGCCCAATGCTGTCGCGAAGAGTTCGGCTCTGTCA 385 
           ||||||| || |||||||  ||| |  |   ||  |||||||| | ||||||||||| |||||||| |     
Sbjct 5232 GGCCGCGGTCGGCCCGGGGCTTCTC--CGGAGGTTCCCAATGCCGCCGCGAAGAGTTGGGCTCTGTTA 5297

Query  386 GCCCGGCTGGGTCCGGGTGGGGGC 409 
           |||  || || |||   |||||||     
Sbjct 5298 GCC--GCGGGTTCC--TTGGGGGC 5317
```

Report:

|  |  |
| --- | --- |
| sequence start ?  ``` Start position of the estimated full-length sequence in genome. Start index < end index. ``` : | 4908 |
| sequence end ?  ``` End position of the estimated full-length sequence in genome. Start index < end index. ``` : | 5422 |
| bit score (CM) ?  ``` The score for aligning estimated full-length sequence to CM model   (computed by RSEARCH -> default,   infered from Rfam or provided by user) ``` : | 0.96 |
| Homology estimate ?  ``` Quick homology estimate:   Not homologous: bit score < 0   Homologous: bit score > 20 and bit score > 0.5 * query length   Uncertain otherwise ``` : | Uncertain |

Estimated full-length sequence:


?

```
Click checkbox to select multiple seuqences.
Fasta header format:
  UID|accession.versionSTRAND start-end
```

>uid:29|XM\_016148125.1fw 4908-5422
UCUCGCGAGAGCUGGGUAAGGCGGGACUCGGCAAAUCCGCGCGGGCAGCGCCCGCUCCUU
UAUAAGGAGAUGCGGCCGGUGCGCGCUGGUCUGGGUUAGGUGACGGCCUGCUUUUUUUCU
AACCCUAUCUGAGAAGGGCGUAGGCGCCGUGCUUUUGCUCCCCGCUCGCUGUUUUUCUCG
CUGGCUUUCAGCGCGCGGAAAAGCCUCGGCCUACCGCCGUACAUCUUCCGAUUUAGAGCA
AACAAAAAAUGUCAGCGACUGGCCAAGUCACCCUUCCCAGGAACCUGUGGAAUUCGCCCG
CCCGGCCCCCGCGCCCCGCCUAAAGGCCGCGGUCGGCCCGGGGCUUCUCCGGAGGUUCCC
AAUGCCGCCGCGAAGAGUUGGGCUCUGUUAGCCGCGGGUUCCUUGGGGGCUAAGGGCGAG
GCUGUGGCCACAGGGAGAGAAACGGAGCGGGUUCCCGCGUGCGGUGCACUUCCCUGAGCU
GUGGGACUUGCAUCCGGGACUGGGCUCAAACAUAC

C-A-r-Rc


?

```
Visualisation of predicted secondary structure.
To save the image:
  Right click on the image -> Save Image as.
```


centroid


?

```
Visualisation of predicted secondary structure.
To save the image:
  Right click on the image -> Save Image as.
```


fq-sub


?

```
Visualisation of predicted secondary structure.
To save the image:
  Right click on the image -> Save Image as.
```

Load Sequence viewer

TurboFold: Number of sequences is less then required. n=2 (4)  
uid:29|XM\_016148125.1: Sequence cannot be extended sufficiently by unalined portion of query. THIS IS PROBABLY FRAGMENT! Trimmed downstream.  
uid:29|XM\_016148125.1: Sequence cannot be extended sufficiently. Missing nt downstream in the genome.

### Hit: AY760074.1

AY760074.1 Muntiacus muntjak vaginalis telomerase RNA (TERC) gene, partial sequence

```
?

```
This is BLAST alignment as read from the input file
```

Score = 60.0 bits (55.4), Expect = 9.28E-04
 Identities = 68/90 (76%), Gaps = 5/90 (6%)
 Strand = Plus/Plus
Query 318 GGCCGCGATCAGCCCGGGCTTTCCCTACTTTGGGGCCCAATGCTGTCGCGAAGAGTTCGGCTCTGTCAGC 387
          ||||||| || |||||||  ||| |  |   || ||||| ||| | || |||||||| ||||||||||||    
Sbjct 159 GGCCGCGGTCGGCCCGGGGCTTCTC--CGGAGGTGCCCATTGCCGCCGTGAAGAGTTGGGCTCTGTCAGC 226

Query 388 C-CGGCTGGGTCCGGGTGGG 406
          | ||| | | ||  ||||||    
Sbjct 227 CGCGGGTCGCTC--GGTGGG 244
```

Report:

|  |  |
| --- | --- |
| sequence start ?  ``` Start position of the estimated full-length sequence in genome. Start index < end index. ``` : | 1 |
| sequence end ?  ``` End position of the estimated full-length sequence in genome. Start index < end index. ``` : | 321 |
| bit score (CM) ?  ``` The score for aligning estimated full-length sequence to CM model   (computed by RSEARCH -> default,   infered from Rfam or provided by user) ``` : | 10.66 |
| Homology estimate ?  ``` Quick homology estimate:   Not homologous: bit score < 0   Homologous: bit score > 20 and bit score > 0.5 * query length   Uncertain otherwise ``` : | Uncertain |

Estimated full-length sequence:


?

```
Click checkbox to select multiple seuqences.
Fasta header format:
  UID|accession.versionSTRAND start-end
```

>uid:30|AY760074.1fw 1-321
CGCUGUUUUUCUCGCUGACUUUCAGCGGGCGGAAAAGCCUCGGCCUACCGCCAGACACCA
UCCAGUGUGGAACAAACAAAAAAUGUCAGCCGCUGGCUCGCUCACCUCUCCCGGGAACCU
GCGGUGGCCCGCCCGCCCAGCCCCAGCGCCCCGCCUGAGGCCGCGGUCGGCCCGGGGCUU
CUCCGGAGGUGCCCAUUGCCGCCGUGAAGAGUUGGGCUCUGUCAGCCGCGGGUCGCUCGG
UGGGCCGAGGCAUGGCUGUAACCGCAGGGAAAGGAACGGAGCGGGGUCCCCGCGCGCGGU
GCGCUUCCCUGAGCUGUGGGA

C-A-r-Rc


?

```
Visualisation of predicted secondary structure.
To save the image:
  Right click on the image -> Save Image as.
```


centroid


?

```
Visualisation of predicted secondary structure.
To save the image:
  Right click on the image -> Save Image as.
```


fq-sub


?

```
Visualisation of predicted secondary structure.
To save the image:
  Right click on the image -> Save Image as.
```

Load Sequence viewer

TurboFold: Number of sequences is less then required. n=2 (4)  
uid:30|AY760074.1: Sequence cannot be extended sufficiently by unaligned portion of query. THIS IS PROBABLY FRAGMENT! Trimmed upstream.  
uid:30|AY760074.1: Sequence cannot be extended sufficiently. Missing -188 nt upstream in the genome.  
uid:30|AY760074.1: Sequence cannot be extended sufficiently. Missing nt downstream in the genome.

### Hit: AF221921.1

AF221921.1 Suncus murinus telomerase RNA gene, sequence

```
?

```
This is BLAST alignment as read from the input file
```

Score = 60.0 bits (55.4), Expect = 9.28E-04
 Identities = 58/76 (76%), Gaps = 8/76 (11%)
 Strand = Plus/Plus
Query 315 CCGGGCCGCGATCAGCCCGGGCTTTCCCTACTTTGG--GGCCCAATGCTGTCGCGAAGAGTTCGGCTCTG 382
          || ||||||| || | ||||||||      ||| ||  | |||  ||| ||||||||||||||| |||||    
Sbjct 363 CCAGGCCGCGGTCGGTCCGGGCTT------CTTCGGAAGTCCCGTTGCCGTCGCGAAGAGTTCGCCTCTG 426

Query 383 TCAGCC 388
          ||||||    
Sbjct 427 TCAGCC 432
```

Report:

|  |  |
| --- | --- |
| sequence start ?  ``` Start position of the estimated full-length sequence in genome. Start index < end index. ``` : | 88 |
| sequence end ?  ``` End position of the estimated full-length sequence in genome. Start index < end index. ``` : | 548 |
| bit score (CM) ?  ``` The score for aligning estimated full-length sequence to CM model   (computed by RSEARCH -> default,   infered from Rfam or provided by user) ``` : | 9.47 |
| Homology estimate ?  ``` Quick homology estimate:   Not homologous: bit score < 0   Homologous: bit score > 20 and bit score > 0.5 * query length   Uncertain otherwise ``` : | Uncertain |

Estimated full-length sequence:


?

```
Click checkbox to select multiple seuqences.
Fasta header format:
  UID|accession.versionSTRAND start-end
```

>uid:31|AF221921.1fw 88-548
CCGCCAUGUUGGCGGGUUGCGGGAGCUGCGAGCGGCCGUCUCGUCUAACCCUAAAGAGAA
AGGCGUAGGUGCUUGGCCUUGGCGACUCGCCCGCUGUUUUUUGGCUGGCUUUCAGCGGGU
GAAGAGGCCCAAGACCUACCGCCACCCACCGUCUAGUGUCUUAAGGGCACAAAGUCCUGC
CCGCCACCCUUCGAGGAGCGAAACCCAAAAAAGUCAGCCCCUGGCCGCCUCUCGCCCUCU
CGCAACCCGCCUCCGUCCCAGCCUCCAUCCCCGCCCCAGGCCGCGGUCGGUCCGGGCUUC
UUCGGAAGUCCCGUUGCCGUCGCGAAGAGUUCGCCUCUGUCAGCCGCGGGGCUUGGGGCC
AGGGACGGGACCCUGUCCGCAGGGAGAGAAACUGGAGCCGGGCCCUCCACGGUGCCUCCC
CGAGCUGUGGGAUCUGCACCCGGGACUCGAACCCUACACUU

C-A-r-Rc


?

```
Visualisation of predicted secondary structure.
To save the image:
  Right click on the image -> Save Image as.
```


centroid


?

```
Visualisation of predicted secondary structure.
To save the image:
  Right click on the image -> Save Image as.
```


fq-sub


?

```
Visualisation of predicted secondary structure.
To save the image:
  Right click on the image -> Save Image as.
```

Load Sequence viewer

TurboFold: Number of sequences is less then required. n=2 (4)  
uid:31|AF221921.1: Sequence cannot be extended sufficiently by unalined portion of query. THIS IS PROBABLY FRAGMENT! Trimmed downstream.  
uid:31|AF221921.1: Sequence cannot be extended sufficiently. Missing nt downstream in the genome.

### Hit: XM\_024245284.1

XM\_024245284.1 PREDICTED: Pongo abelii uncharacterized LOC112132745 (LOC112132745), mRNA

```
?

```
This is BLAST alignment as read from the input file
```

Score = 59.0 bits (54.5), Expect = 3.24E-03
 Identities = 55/71 (77%), Gaps = 2/71 (3%)
 Strand = Plus/Plus
Query 318 GGCCGCGATCAGCCCGGGCTTTCCCTACTTTGGGGCCCAATGCTGTCGCGAAGAGTTCGGCTCTGTCAGC 387
          ||||||| || |||||||  ||| |  |   ||  |||| ||| | ||||||||||| ||||||||||||    
Sbjct 247 GGCCGCGGTCGGCCCGGGGCTTCTC--CGGAGGCACCCACTGCCGCCGCGAAGAGTTGGGCTCTGTCAGC 314

Query 388 C 388
          |    
Sbjct 315 C 315
```

Report:

|  |  |
| --- | --- |
| sequence start ?  ``` Start position of the estimated full-length sequence in genome. Start index < end index. ``` : | 1 |
| sequence end ?  ``` End position of the estimated full-length sequence in genome. Start index < end index. ``` : | 497 |
| bit score (CM) ?  ``` The score for aligning estimated full-length sequence to CM model   (computed by RSEARCH -> default,   infered from Rfam or provided by user) ``` : | 6.67 |
| Homology estimate ?  ``` Quick homology estimate:   Not homologous: bit score < 0   Homologous: bit score > 20 and bit score > 0.5 * query length   Uncertain otherwise ``` : | Uncertain |

Estimated full-length sequence:


?

```
Click checkbox to select multiple seuqences.
Fasta header format:
  UID|accession.versionSTRAND start-end
```

>uid:32|XM\_024245284.1fw 1-497
CGGAGGGUGGGCCUGGGAGGGGUGGCGGCCAUAUUUUGUCUAACCCUAACUGAGAAGGGC
GUAGGCGCCGUGCUUUUGCUCCCCGCGCGCUGUUUUUUUCGCUGACUUUCAGCGGGCGGA
AAAGCCUCGGCCUGCCGCCUUCCACCGUUCAUUCUGGAGCAAACAAAAAAUGUCAGCUGC
UGGCCCGUUCGCCCCUCCCGGGGACCUGCGGCGGGUCGCCUGCCCAGCCCCCGAACCCCG
CCUGGAGGCCGCGGUCGGCCCGGGGCUUCUCCGGAGGCACCCACUGCCGCCGCGAAGAGU
UGGGCUCUGUCAGCCGCGGGUCUCUCGGGGGCGAGGGCGAGGUUCAGGCCUUUCAGGCCG
CAGGAAGAGGAACGGAGCGAGUUCCCGCGCGCGGCGCGCUUCCCUGAGCUGUGGGACGUG
CACCCAGGACUCGGCUCACACAAUGCAGUUCGCUUUCCUGUUGGGAACGCCGAUCGUGCG
CAUCCGUCACCCCUCGC

C-A-r-Rc


?

```
Visualisation of predicted secondary structure.
To save the image:
  Right click on the image -> Save Image as.
```


centroid


?

```
Visualisation of predicted secondary structure.
To save the image:
  Right click on the image -> Save Image as.
```


fq-sub


?

```
Visualisation of predicted secondary structure.
To save the image:
  Right click on the image -> Save Image as.
```

Load Sequence viewer

TurboFold: Number of sequences is less then required. n=2 (4)  
uid:32|XM\_024245284.1: Sequence cannot be extended sufficiently by unaligned portion of query. THIS IS PROBABLY FRAGMENT! Trimmed upstream.  
uid:32|XM\_024245284.1: Sequence cannot be extended sufficiently. Missing -100 nt upstream in the genome.

### Hit: XM\_019023020.1

XM\_019023020.1 PREDICTED: Gorilla gorilla gorilla uncharacterized LOC109026131 (LOC109026131), mRNA

```
?

```
This is BLAST alignment as read from the input file
```

Score = 59.0 bits (54.5), Expect = 3.24E-03
 Identities = 55/71 (77%), Gaps = 2/71 (3%)
 Strand = Plus/Plus
Query 318 GGCCGCGATCAGCCCGGGCTTTCCCTACTTTGGGGCCCAATGCTGTCGCGAAGAGTTCGGCTCTGTCAGC 387
          ||||||| || |||||||  ||| |  |   ||  |||| ||| | ||||||||||| ||||||||||||    
Sbjct 247 GGCCGCGGTCGGCCCGGGGCTTCTC--CGGAGGCACCCACTGCCGCCGCGAAGAGTTGGGCTCTGTCAGC 314

Query 388 C 388
          |    
Sbjct 315 C 315
```

Report:

|  |  |
| --- | --- |
| sequence start ?  ``` Start position of the estimated full-length sequence in genome. Start index < end index. ``` : | 1 |
| sequence end ?  ``` End position of the estimated full-length sequence in genome. Start index < end index. ``` : | 505 |
| bit score (CM) ?  ``` The score for aligning estimated full-length sequence to CM model   (computed by RSEARCH -> default,   infered from Rfam or provided by user) ``` : | 3.58 |
| Homology estimate ?  ``` Quick homology estimate:   Not homologous: bit score < 0   Homologous: bit score > 20 and bit score > 0.5 * query length   Uncertain otherwise ``` : | Uncertain |

Estimated full-length sequence:


?

```
Click checkbox to select multiple seuqences.
Fasta header format:
  UID|accession.versionSTRAND start-end
```

>uid:33|XM\_019023020.1fw 1-505
CGGAGGGUGGGCCUGGGAGGGGUGGUGGCCAUUUUUUGUCUAACCCUAACUGAGAAGGGC
GUAGGCGCCGUGCUUUUGCUCCCCGCGCGCUGUUUUUCUCGCUGACUUUCAGCGGGCGGA
AAAGCCUCGGCCUGCCGCCUUCCACCGUUCAUUCUGGAGCAAACAAAAAAUGUCAGCUGC
UGGCCCGUUCGCCCCUCCCGGGGACCUGCGGCGGGUCGCCUGCCCAGCCCCCGAACCCCG
CCUGGAGGCCGCGGUCGGCCCGGGGCUUCUCCGGAGGCACCCACUGCCGCCGCGAAGAGU
UGGGCUCUGUCAGCCGCGGGUUUCUCGGGGGCGAGGGCGAGGUUCAGGCCUUUCAGGCCG
CAGGAAGAGGAACGGAGCGAGUCCCCGCGCGCGGCGCGCUUCCCUGAGCUGUGGGACGUG
CACCCAGGACUCGGCUCACACAUGCAGUUCGCUUUCCUAUUGGUGGGGGGAACGCCGAUC
GUGCGCAUCAGUCACCCCUCGCCGG

C-A-r-Rc


?

```
Visualisation of predicted secondary structure.
To save the image:
  Right click on the image -> Save Image as.
```


centroid


?

```
Visualisation of predicted secondary structure.
To save the image:
  Right click on the image -> Save Image as.
```


fq-sub


?

```
Visualisation of predicted secondary structure.
To save the image:
  Right click on the image -> Save Image as.
```

Load Sequence viewer

TurboFold: Number of sequences is less then required. n=2 (4)  
uid:33|XM\_019023020.1: Sequence cannot be extended sufficiently. Missing -100 nt upstream in the genome.  
uid:33|XM\_019023020.1: Sequence cannot be extended sufficiently by unaligned portion of query. THIS IS PROBABLY FRAGMENT! Trimmed upstream.

### Hit: NR\_033816.1

NR\_033816.1 Macaca mulatta telomerase RNA component (TERC), telomerase RNA

```
?

```
This is BLAST alignment as read from the input file
```

Score = 59.0 bits (54.5), Expect = 3.24E-03
 Identities = 55/71 (77%), Gaps = 2/71 (3%)
 Strand = Plus/Plus
Query 318 GGCCGCGATCAGCCCGGGCTTTCCCTACTTTGGGGCCCAATGCTGTCGCGAAGAGTTCGGCTCTGTCAGC 387
          ||||||| || |||||||  ||| |  |   || ||||| ||  | ||||||||||| ||||||||||||    
Sbjct 253 GGCCGCGGTCGGCCCGGGGCTTCTC--CGGAGGCGCCCATTGTCGCCGCGAAGAGTTGGGCTCTGTCAGC 320

Query 388 C 388
          |    
Sbjct 321 C 321
```

Report:

|  |  |
| --- | --- |
| sequence start ?  ``` Start position of the estimated full-length sequence in genome. Start index < end index. ``` : | 1 |
| sequence end ?  ``` End position of the estimated full-length sequence in genome. Start index < end index. ``` : | 452 |
| bit score (CM) ?  ``` The score for aligning estimated full-length sequence to CM model   (computed by RSEARCH -> default,   infered from Rfam or provided by user) ``` : | 14.6 |
| Homology estimate ?  ``` Quick homology estimate:   Not homologous: bit score < 0   Homologous: bit score > 20 and bit score > 0.5 * query length   Uncertain otherwise ``` : | Uncertain |

Estimated full-length sequence:


?

```
Click checkbox to select multiple seuqences.
Fasta header format:
  UID|accession.versionSTRAND start-end
```

>uid:34|NR\_033816.1fw 1-452
GGGUUGCGGAGGGUGGGCCUGGGAGAGGUGGCGGCCAUUUUUUGUCUAACCCUAACUGAG
AAGGGCGUAGGCGCCGCGCUUUUGCUCCCCGCGCGCUGUUUUUCUCGCUGACUUUCAGCG
GGCGGAAAAGCCUCGGCCUACCGCCUUCCACCGUUCAUUCUGGAGCAAACAAAAAAUGUC
AGCUGCUGGCCCGUUCGCCCCUCCCGGGGACCUGCGGCGGGUCGCCUGCCCAGCCCCCGA
ACCCCGCCUGGAGGCCGCGGUCGGCCCGGGGCUUCUCCGGAGGCGCCCAUUGUCGCCGCG
AAGAGUUGGGCUCUGUCAGCCGCGGGUCUCUCGGGGGUGAGGGCGAGGUACAGGCCUUUC
AGGCCGCAGGAAGAGGAACGGAGCGGAGUCCCCGCGCGCGGCGCGCUUCCCUGAGCUGUG
GGACGUGCACCCAGGACUCGGCUCACACAUGC

C-A-r-Rc


?

```
Visualisation of predicted secondary structure.
To save the image:
  Right click on the image -> Save Image as.
```


centroid


?

```
Visualisation of predicted secondary structure.
To save the image:
  Right click on the image -> Save Image as.
```


fq-sub


?

```
Visualisation of predicted secondary structure.
To save the image:
  Right click on the image -> Save Image as.
```

Load Sequence viewer

TurboFold: Number of sequences is less then required. n=2 (4)  
uid:34|NR\_033816.1: Sequence cannot be extended sufficiently by unaligned portion of query. THIS IS PROBABLY FRAGMENT! Trimmed upstream.  
uid:34|NR\_033816.1: Sequence cannot be extended sufficiently. Missing -94 nt upstream in the genome.  
uid:34|NR\_033816.1: Sequence cannot be extended sufficiently. Missing nt downstream in the genome.

### Hit: AC201932.3

AC201932.3 MACACA MULATTA BAC clone CH250-413I20 from chromosome 2, complete sequence

```
?

```
This is BLAST alignment as read from the input file
```

Score = 59.0 bits (54.5), Expect = 3.24E-03
 Identities = 55/71 (77%), Gaps = 2/71 (3%)
 Strand = Plus/Minus
Query  318 GGCCGCGATCAGCCCGGGCTTTCCCTACTTTGGGGCCCAATGCTGTCGCGAAGAGTTCGGCTCTGTCA 385 
           ||||||| || |||||||  ||| |  |   || ||||| ||  | ||||||||||| ||||||||||     
Sbjct 5810 GGCCGCGGTCGGCCCGGGGCTTCTC--CGGAGGCGCCCATTGTCGCCGCGAAGAGTTGGGCTCTGTCA 5745

Query  386 GCC 388 
           |||     
Sbjct 5744 GCC 5742
```

Report:

|  |  |
| --- | --- |
| sequence start ?  ``` Start position of the estimated full-length sequence in genome. Start index < end index. ``` : | 5581 |
| sequence end ?  ``` End position of the estimated full-length sequence in genome. Start index < end index. ``` : | 6131 |
| bit score (CM) ?  ``` The score for aligning estimated full-length sequence to CM model   (computed by RSEARCH -> default,   infered from Rfam or provided by user) ``` : | 3.54 |
| Homology estimate ?  ``` Quick homology estimate:   Not homologous: bit score < 0   Homologous: bit score > 20 and bit score > 0.5 * query length   Uncertain otherwise ``` : | Uncertain |

Estimated full-length sequence:


?

```
Click checkbox to select multiple seuqences.
Fasta header format:
  UID|accession.versionSTRAND start-end
```

>uid:35|AC201932.3rc 5581-6131
AAGCUUGGCCAAUCCGCGCGGUCGGCGGCCGCUCCCUUUAUAAGCGGACUCGCCCGGCAG
UGCUCUGGGUUGCGGAGGGUGGGCCUGGGAGAGGUGGCGGCCAUUUUUUGUCUAACCCUA
ACUGAGAAGGGCGUAGGCGCCGCGCUUUUGCUCCCCGCGCGCUGUUUUUCUCGCUGACUU
UCAGCGGGCGGAAAAGCCUCGGCCUACCGCCUUCCACCGUUCAUUCUGGAGCAAACAAAA
AAUGUCAGCUGCUGGCCCGUUCGCCCCUCCCGGGGACCUGCGGCGGGUCGCCUGCCCAGC
CCCCGAACCCCGCCUGGAGGCCGCGGUCGGCCCGGGGCUUCUCCGGAGGCGCCCAUUGUC
GCCGCGAAGAGUUGGGCUCUGUCAGCCGCGGGUCUCUCGGGGGUGAGGGCGAGGUACAGG
CCUUUCAGGCCGCAGGAAGAGGAACGGAGCGGAGUCCCCGCGCGCGGCGCGCUUCCCUGA
GCUGUGGGACGUGCACCCAGGACUCGGCUCACACAUGCAGUUCGCUUUCCUGCUGGUGGG
GGGACGCCGAU

C-A-r-Rc


?

```
Visualisation of predicted secondary structure.
To save the image:
  Right click on the image -> Save Image as.
```


centroid


?

```
Visualisation of predicted secondary structure.
To save the image:
  Right click on the image -> Save Image as.
```


fq-sub


?

```
Visualisation of predicted secondary structure.
To save the image:
  Right click on the image -> Save Image as.
```

Load Sequence viewer

TurboFold: Number of sequences is less then required. n=2 (4)

### Hit: AF221930.1

AF221930.1 Geomys breviceps telomerase RNA gene, sequence

```
?

```
This is BLAST alignment as read from the input file
```

Score = 58.0 bits (53.6), Expect = 3.24E-03
 Identities = 47/58 (81%), Gaps = 2/58 (3%)
 Strand = Plus/Plus
Query 349 GGGGCCCAATGCTGTCGCGAAGAGTTCGGCTCTGTCAGCCCGGCTGGGTCCGGGTGGG 406
          || ||||| ||| | ||||||||||| |||||||||||||  || || |||| | |||    
Sbjct 381 GGTGCCCATTGCCGCCGCGAAGAGTTAGGCTCTGTCAGCC--GCGGGATCCGCGCGGG 436
```

Report:

|  |  |
| --- | --- |
| sequence start ?  ``` Start position of the estimated full-length sequence in genome. Start index < end index. ``` : | 30 |
| sequence end ?  ``` End position of the estimated full-length sequence in genome. Start index < end index. ``` : | 546 |
| bit score (CM) ?  ``` The score for aligning estimated full-length sequence to CM model   (computed by RSEARCH -> default,   infered from Rfam or provided by user) ``` : | 15.36 |
| Homology estimate ?  ``` Quick homology estimate:   Not homologous: bit score < 0   Homologous: bit score > 20 and bit score > 0.5 * query length   Uncertain otherwise ``` : | Uncertain |

Estimated full-length sequence:


?

```
Click checkbox to select multiple seuqences.
Fasta header format:
  UID|accession.versionSTRAND start-end
```

>uid:36|AF221930.1fw 30-546
CUGGAGCUCAGCUAAUACGCAGGCGCGGCACGCGCCCUUUAUAAGGCGCGGGCGCGCGUG
GCUCGGCCGGCGUGCCUGGGAGGGGGGGCUGGUCCCACUCUUCUAACCCUAAGGGUGUCG
GCUGUAGGCGCCGUGCUUUUUACUUCCCCGCGCGCUGUUUUUCUGGCUGGCUUUCAGCGA
GCGGAAAAAGCUUUGGUCUACAGGCCACUCACUUUGUAUCCCGAAACCAAAUUGAAAAAA
AAAAAUGCCAGCUCCGGCCGGUCCACCCCUCCCGGGGUCCUGCGGCCCGGCCGCCCGCCC
GAACCCCCCGCGACCCGCCUGAGGCCACGGCCGGCCCGGGGCCUCUGCGGAGGUGCCCAU
UGCCGCCGCGAAGAGUUAGGCUCUGUCAGCCGCGGGAUCCGCGCGGGCCGGGCCGCGGCC
GGACGCCGAUCCGCAGGGACAGCAACGGAACCGGCCCCUUCGCCCUGUGCGCUUCCCGGA
GCUGUGGGAUGAGCACCCGGGGUCGGCUCCUACAGUU

C-A-r-Rc


?

```
Visualisation of predicted secondary structure.
To save the image:
  Right click on the image -> Save Image as.
```


centroid


?

```
Visualisation of predicted secondary structure.
To save the image:
  Right click on the image -> Save Image as.
```


fq-sub


?

```
Visualisation of predicted secondary structure.
To save the image:
  Right click on the image -> Save Image as.
```

Load Sequence viewer

TurboFold: Number of sequences is less then required. n=2 (4)  
uid:36|AF221930.1: Sequence cannot be extended sufficiently by unalined portion of query. THIS IS PROBABLY FRAGMENT! Trimmed downstream.  
uid:36|AF221930.1: Sequence cannot be extended sufficiently. Missing nt downstream in the genome.

### Hit: XR\_004001997.1

XR\_004001997.1 PREDICTED: Gopherus evgoodei uncharacterized LOC115657533 (LOC115657533), ncRNA

```
?

```
This is BLAST alignment as read from the input file
```

Score = 56.0 bits (51.8), Expect = 1.13E-02
 Identities = 42/50 (84%), Gaps = 1/50 (2%)
 Strand = Plus/Plus
Query 354 CCAATGCTGTCGCGAAGAGTTCGGCTCTGTCAGCC-CGGCTGGGTCCGGG 402
          ||| ||||| ||||||||||||| ||||||||||| |||  | | |||||    
Sbjct 224 CCACTGCTGCCGCGAAGAGTTCGTCTCTGTCAGCCTCGGGGGCGGCCGGG 273
```

Report:

|  |  |
| --- | --- |
| sequence start ?  ``` Start position of the estimated full-length sequence in genome. Start index < end index. ``` : | 1 |
| sequence end ?  ``` End position of the estimated full-length sequence in genome. Start index < end index. ``` : | 449 |
| bit score (CM) ?  ``` The score for aligning estimated full-length sequence to CM model   (computed by RSEARCH -> default,   infered from Rfam or provided by user) ``` : | 12.18 |
| Homology estimate ?  ``` Quick homology estimate:   Not homologous: bit score < 0   Homologous: bit score > 20 and bit score > 0.5 * query length   Uncertain otherwise ``` : | Uncertain |

Estimated full-length sequence:


?

```
Click checkbox to select multiple seuqences.
Fasta header format:
  UID|accession.versionSTRAND start-end
```

>uid:37|XR\_004001997.1fw 1-449
GGCUCGGUCUUUCUAACCCUAAGCGGAACGUGGCCCCUCCCCGCCGCAGCCGUCCGCUGU
UUUACUCGCUGACUUUCAGCGGGCGGGGGGAGCGGGCGGAGACACCAACCAAAAAACGUC
AGCGAGGGGCCCUCCCCUCCCACGCCGACCUGGGCCGGUGUGGGGCCCGCCAGGGAAGUC
CCCGCCGCCCCGCCCUGGUGAGGCCGCGGUCAGCCGGUUCGCGCCACUGCUGCCGCGAAG
AGUUCGUCUCUGUCAGCCUCGGGGGCGGCCGGGGUGGAAGGGCGGGUCCCGUGCGGGUCG
GCCGGGAGAGCAAACAUUUCCUGCCAGUUAAAAGUAAGCAGGGAACAGCUCAAGGUUCUG
CAUCUCUCUGGCCAGUAGGGGUAUGCUCCCUGAAUAAUUGAGUACUCUCAAAAUCACUGA
AUCCCUGAAAAUACUCUUACUUUGCUCUG

C-A-r-Rc


?

```
Visualisation of predicted secondary structure.
To save the image:
  Right click on the image -> Save Image as.
```


centroid


?

```
Visualisation of predicted secondary structure.
To save the image:
  Right click on the image -> Save Image as.
```


fq-sub


?

```
Visualisation of predicted secondary structure.
To save the image:
  Right click on the image -> Save Image as.
```

Load Sequence viewer

uid:37|XR\_004001997.1: Sequence cannot be extended sufficiently by unaligned portion of query. THIS IS PROBABLY FRAGMENT! Trimmed upstream.  
TurboFold: Number of sequences is less then required. n=2 (4)  
uid:37|XR\_004001997.1: Sequence cannot be extended sufficiently. Missing -159 nt upstream in the genome.

### Hit: AF221911.1

AF221911.1 Chelydra serpentina telomerase RNA gene, sequence

```
?

```
This is BLAST alignment as read from the input file
```

Score = 56.0 bits (51.8), Expect = 1.13E-02
 Identities = 42/50 (84%), Gaps = 1/50 (2%)
 Strand = Plus/Plus
Query 354 CCAATGCTGTCGCGAAGAGTTCGGCTCTGTCAGCC-CGGCTGGGTCCGGG 402
          ||| ||||| ||||||||||||| ||||||||||| |||  | | |||||    
Sbjct 348 CCACTGCTGCCGCGAAGAGTTCGTCTCTGTCAGCCTCGGGGGCGGCCGGG 397
```

Report:

|  |  |
| --- | --- |
| sequence start ?  ``` Start position of the estimated full-length sequence in genome. Start index < end index. ``` : | 2 |
| sequence end ?  ``` End position of the estimated full-length sequence in genome. Start index < end index. ``` : | 513 |
| bit score (CM) ?  ``` The score for aligning estimated full-length sequence to CM model   (computed by RSEARCH -> default,   infered from Rfam or provided by user) ``` : | 18.05 |
| Homology estimate ?  ``` Quick homology estimate:   Not homologous: bit score < 0   Homologous: bit score > 20 and bit score > 0.5 * query length   Uncertain otherwise ``` : | Uncertain |

Estimated full-length sequence:


?

```
Click checkbox to select multiple seuqences.
Fasta header format:
  UID|accession.versionSTRAND start-end
```

>uid:38|AF221911.1fw 2-513
CCGCCGAGCCAAUGGGAAUAGAGGAGACUCCCGCUAGCCAAUCCAUGCGCGGGAGGGCGG
GACGGUGGAAGGUAUAUAAGACCCGCGGCCAGGCGGGUCUGACCGCUGCGGCGGCAGGUG
GGGGCUCAGUCUUUCUAACCCUAAGCGAAAUGUGACCCCUCCCCGCUGCAGCCGUCCGCU
GUUUUACUCGCUGACUUUCAGCGGACGGGGGGAGCGGGUGGAGACGCCAACCAAAAAACG
UCAGCGAGGGGCCCUCCCCUCCCACGCCGACCUGGGCCUGUGGUGGGGCCCGCCAGCGAA
GUCCCCGCCGCCCCGCCCCGGUGAGGCCGCGGUCAGCCGGCUCGCGCCACUGCUGCCGCG
AAGAGUUCGUCUCUGUCAGCCUCGGGGGCGGCCGGGGUGGAAGGGCGGGUCCCGAGCCCG
UCGGCCGGGAGAGCAAACGUGAGCGGCAGCCCCUGCGCCACCGCCCUCCCCUAAGCUGUG
GGGCCCGCGGUCGGGGCUGCGCUCAGACACGC

C-A-r-Rc


?

```
Visualisation of predicted secondary structure.
To save the image:
  Right click on the image -> Save Image as.
```


centroid


?

```
Visualisation of predicted secondary structure.
To save the image:
  Right click on the image -> Save Image as.
```


fq-sub


?

```
Visualisation of predicted secondary structure.
To save the image:
  Right click on the image -> Save Image as.
```

Load Sequence viewer

TurboFold: Number of sequences is less then required. n=2 (4)  
uid:38|AF221911.1: Sequence cannot be extended sufficiently by unaligned portion of query. THIS IS PROBABLY FRAGMENT! Trimmed upstream.  
uid:38|AF221911.1: Sequence cannot be extended sufficiently. Missing nt downstream in the genome.  
uid:38|AF221911.1: Sequence cannot be extended sufficiently by unalined portion of query. THIS IS PROBABLY FRAGMENT! Trimmed downstream.  
uid:38|AF221911.1: Sequence cannot be extended sufficiently. Missing -35 nt upstream in the genome.

### Hit: NR\_003556.1

NR\_003556.1 Xenopus laevis telomerase RNA component L homeolog (terc.L), telomerase RNA

```
?

```
This is BLAST alignment as read from the input file
```

Score = 55.0 bits (50.9), Expect = 3.95E-02
 Identities = 41/50 (82%), Gaps = 0/50 (0%)
 Strand = Plus/Plus
Query 358 TGCTGTCGCGAAGAGTTCGGCTCTGTCAGCCCGGCTGGGTCCGGGTGGGG 407
          ||||||||||||||||||| |||||| |||||    ||| || |||| ||    
Sbjct 283 TGCTGTCGCGAAGAGTTCGTCTCTGTTAGCCCTTGGGGGCCCTGGTGCGG 332
```

Report:

|  |  |
| --- | --- |
| sequence start ?  ``` Start position of the estimated full-length sequence in genome. Start index < end index. ``` : | 1 |
| sequence end ?  ``` End position of the estimated full-length sequence in genome. Start index < end index. ``` : | 449 |
| bit score (CM) ?  ``` The score for aligning estimated full-length sequence to CM model   (computed by RSEARCH -> default,   infered from Rfam or provided by user) ``` : | -7.07 |
| Homology estimate ?  ``` Quick homology estimate:   Not homologous: bit score < 0   Homologous: bit score > 20 and bit score > 0.5 * query length   Uncertain otherwise ``` : | Not homologous |

Estimated full-length sequence:


?

```
Click checkbox to select multiple seuqences.
Fasta header format:
  UID|accession.versionSTRAND start-end
```

>uid:39|NR\_003556.1fw 1-449
AAUCAGCGUUUAAAGCUCAAUGUGGACGGAGGUCUCUGUUUCGCUAACCCUAAUACACUG
GCUUCAGGGCGAUGGCUCUUCGCGGCGGUGCCUGUUGUUUUACUUGCUGACUUUCAGCGG
GCACGGAGAGCAAGCGUAGACGACGACUAAAAAACGUCAGCUGGGAGACUCCUCCGUUCG
CACAGCCCGACCUGCUCCAUUGCCCAAGAGCCCCGGGUUUUCUCUGUGGAAUGUAUCAGG
CGCUCCCCGGUCUGUCUGUCUGGGGCCGCGGUCGGCAUCAUCUGCUGUCGCGAAGAGUUC
GUCUCUGUUAGCCCUUGGGGGCCCUGGUGCGGAGUGGAGAGUCCGGGUCUGGGGGGUCGG
GAGAACAAAAGGGGGCGCGCUGGUGCUCAGGCUCAGUCAUGCUUUCCCUUAGUUGUGGGA
UAUGCGUGUUCAGCCAGUCCCCGACAUGU

C-A-r-Rc


?

```
Visualisation of predicted secondary structure.
To save the image:
  Right click on the image -> Save Image as.
```


centroid


?

```
Visualisation of predicted secondary structure.
To save the image:
  Right click on the image -> Save Image as.
```


fq-sub


?

```
Visualisation of predicted secondary structure.
To save the image:
  Right click on the image -> Save Image as.
```

Load Sequence viewer

TurboFold: Number of sequences is less then required. n=2 (4)  
uid:39|NR\_003556.1: Sequence cannot be extended sufficiently. Missing nt downstream in the genome.  
uid:39|NR\_003556.1: Sequence cannot be extended sufficiently by unaligned portion of query. THIS IS PROBABLY FRAGMENT! Trimmed upstream.  
uid:39|NR\_003556.1: Sequence cannot be extended sufficiently. Missing -104 nt upstream in the genome.

### Hit: AF221934.1

AF221934.1 Dermophis mexicanus telomerase RNA gene, sequence

```
?

```
This is BLAST alignment as read from the input file
```

Score = 55.0 bits (50.9), Expect = 3.95E-02
 Identities = 32/35 (91%), Gaps = 0/35 (0%)
 Strand = Plus/Plus
Query 354 CCAATGCTGTCGCGAAGAGTTCGGCTCTGTCAGCC 388
          ||| ||||| ||||||||||||| |||||||||||    
Sbjct 400 CCACTGCTGCCGCGAAGAGTTCGCCTCTGTCAGCC 434
```

Report:

|  |  |
| --- | --- |
| sequence start ?  ``` Start position of the estimated full-length sequence in genome. Start index < end index. ``` : | 121 |
| sequence end ?  ``` End position of the estimated full-length sequence in genome. Start index < end index. ``` : | 565 |
| bit score (CM) ?  ``` The score for aligning estimated full-length sequence to CM model   (computed by RSEARCH -> default,   infered from Rfam or provided by user) ``` : | 56.59 |
| Homology estimate ?  ``` Quick homology estimate:   Not homologous: bit score < 0   Homologous: bit score > 20 and bit score > 0.5 * query length   Uncertain otherwise ``` : | Uncertain |

Estimated full-length sequence:


?

```
Click checkbox to select multiple seuqences.
Fasta header format:
  UID|accession.versionSTRAND start-end
```

>uid:40|AF221934.1fw 121-565
UCUCUCCGGAGAGGGGCGGUUUCUCUGUCUUCUAACCCUAAUGCGGUGCUUCGGCAGAAG
CUCUCCACUGCGUACGCUCACUGUUUUUCUAGCUAACUUUCAGUGAGCAGGGAGAGCGAA
GUCCAGUUUCACGACAACGGAGAAAAAAUGUUAGCUGGGGAACGUCCCUUUCCCGAGAGC
CCGCGCCGUCCUUUUUCUUCCUCGGGCCCGUUGGCAUAGCCCCUGGCCUCCUCGCUCUAU
AGGCCGCGGUCAGCUCGGGCCCCAGCUCCGGCAGGGGUUCCACUGCUGCCGCGAAGAGUU
CGCCUCUGUCAGCCUUGAGGUGGCCGGGAUAGAAUAGGCGGGCUCGCGGCAGCGCGCGGG
AAAGAGCAAAUGGUGAACUGGGUGCCUGUUGGGGUCGCGUCCCUGAAGAGUGGGAAGUGC
GAUCUGUGUUCCUGUUCAGACACAC

C-A-r-Rc


?

```
Visualisation of predicted secondary structure.
To save the image:
  Right click on the image -> Save Image as.
```


centroid


?

```
Visualisation of predicted secondary structure.
To save the image:
  Right click on the image -> Save Image as.
```


fq-sub


?

```
Visualisation of predicted secondary structure.
To save the image:
  Right click on the image -> Save Image as.
```

Load Sequence viewer

uid:40|AF221934.1: Sequence cannot be extended sufficiently by unalined portion of query. THIS IS PROBABLY FRAGMENT! Trimmed downstream.  
TurboFold: Number of sequences is less then required. n=2 (4)  
uid:40|AF221934.1: Sequence cannot be extended sufficiently. Missing nt downstream in the genome.

### Hit: AF221908.1

AF221908.1 Xenopus laevis telomerase RNA gene, sequence

```
?

```
This is BLAST alignment as read from the input file
```

Score = 55.0 bits (50.9), Expect = 3.95E-02
 Identities = 41/50 (82%), Gaps = 0/50 (0%)
 Strand = Plus/Plus
Query 358 TGCTGTCGCGAAGAGTTCGGCTCTGTCAGCCCGGCTGGGTCCGGGTGGGG 407
          ||||||||||||||||||| |||||| |||||    ||| || |||| ||    
Sbjct 418 TGCTGTCGCGAAGAGTTCGTCTCTGTTAGCCCTTGGGGGCCCTGGTGCGG 467
```

Report:

|  |  |
| --- | --- |
| sequence start ?  ``` Start position of the estimated full-length sequence in genome. Start index < end index. ``` : | 148 |
| sequence end ?  ``` End position of the estimated full-length sequence in genome. Start index < end index. ``` : | 584 |
| bit score (CM) ?  ``` The score for aligning estimated full-length sequence to CM model   (computed by RSEARCH -> default,   infered from Rfam or provided by user) ``` : | 6.37 |
| Homology estimate ?  ``` Quick homology estimate:   Not homologous: bit score < 0   Homologous: bit score > 20 and bit score > 0.5 * query length   Uncertain otherwise ``` : | Uncertain |

Estimated full-length sequence:


?

```
Click checkbox to select multiple seuqences.
Fasta header format:
  UID|accession.versionSTRAND start-end
```

>uid:41|AF221908.1fw 148-584
AAGCUCAAUGUGGACGGAGGUCUCUGUUUCGCUAACCCUAAUACACUGGCUUCAGGGCGA
UGGCUCUUCGCGGCGGUGCCUGUUGUUUUACUUGCUGACUUUCAGCGGGCACGGAGAGCA
AGCGUAGACGACGACUAAAAAACGUCAGCUGGGAGACUCCUCCGUUCGCACAGCCCGACC
UGCUCCAUUGCCCAAGAGCCCCGGGUUUUCUCUGUGGAAUGUAUCAGGCGCUCCCCGGUC
UGUCUGUCUGGGGCCGCGGUCGGCAUCAUCUGCUGUCGCGAAGAGUUCGUCUCUGUUAGC
CCUUGGGGGCCCUGGUGCGGAGUGGAGAGUCCGGGUCUGGGGGGUCGGGAGAACAAAAGG
GGGCGCGCUGGUGCUCAGGCUCAGUCAUGCUUUCCCUUAGUUGUGGGAUAUGCGUGUUCA
GCCAGUCCCCGACAUGU

C-A-r-Rc


?

```
Visualisation of predicted secondary structure.
To save the image:
  Right click on the image -> Save Image as.
```


centroid


?

```
Visualisation of predicted secondary structure.
To save the image:
  Right click on the image -> Save Image as.
```


fq-sub


?

```
Visualisation of predicted secondary structure.
To save the image:
  Right click on the image -> Save Image as.
```

Load Sequence viewer

TurboFold: Number of sequences is less then required. n=2 (4)  
uid:41|AF221908.1: Sequence cannot be extended sufficiently by unalined portion of query. THIS IS PROBABLY FRAGMENT! Trimmed downstream.  
uid:41|AF221908.1: Sequence cannot be extended sufficiently. Missing nt downstream in the genome.

### Hit: NG\_016363.1

NG\_016363.1 Homo sapiens telomerase RNA component (TERC), RefSeqGene (LRG\_347) on chromosome 3

```
?

```
This is BLAST alignment as read from the input file
```

Score = 54.0 bits (50.0), Expect = 3.95E-02
 Identities = 54/71 (76%), Gaps = 2/71 (3%)
 Strand = Plus/Plus
Query  318 GGCCGCGATCAGCCCGGGCTTTCCCTACTTTGGGGCCCAATGCTGTCGCGAAGAGTTCGGCTCTGTCA 385 
           ||||||| || |||||||  ||| |  |   ||  |||| |||   ||||||||||| ||||||||||     
Sbjct 5253 GGCCGCGGTCGGCCCGGGGCTTCTC--CGGAGGCACCCACTGCCACCGCGAAGAGTTGGGCTCTGTCA 5318

Query  386 GCC 388 
           |||     
Sbjct 5319 GCC 5321
```

Report:

|  |  |
| --- | --- |
| sequence start ?  ``` Start position of the estimated full-length sequence in genome. Start index < end index. ``` : | 4942 |
| sequence end ?  ``` End position of the estimated full-length sequence in genome. Start index < end index. ``` : | 5483 |
| bit score (CM) ?  ``` The score for aligning estimated full-length sequence to CM model   (computed by RSEARCH -> default,   infered from Rfam or provided by user) ``` : | 4.6 |
| Homology estimate ?  ``` Quick homology estimate:   Not homologous: bit score < 0   Homologous: bit score > 20 and bit score > 0.5 * query length   Uncertain otherwise ``` : | Uncertain |

Estimated full-length sequence:


?

```
Click checkbox to select multiple seuqences.
Fasta header format:
  UID|accession.versionSTRAND start-end
```

>uid:42|NG\_016363.1fw 4942-5483
GCCAAUCCGUGCGGUCGGCGGCCGCUCCCUUUAUAAGCCGACUCGCCCGGCAGCGCACCG
GGUUGCGGAGGGUGGGCCUGGGAGGGGUGGUGGCCAUUUUUUGUCUAACCCUAACUGAGA
AGGGCGUAGGCGCCGUGCUUUUGCUCCCCGCGCGCUGUUUUUCUCGCUGACUUUCAGCGG
GCGGAAAAGCCUCGGCCUGCCGCCUUCCACCGUUCAUUCUAGAGCAAACAAAAAAUGUCA
GCUGCUGGCCCGUUCGCCCCUCCCGGGGACCUGCGGCGGGUCGCCUGCCCAGCCCCCGAA
CCCCGCCUGGAGGCCGCGGUCGGCCCGGGGCUUCUCCGGAGGCACCCACUGCCACCGCGA
AGAGUUGGGCUCUGUCAGCCGCGGGUCUCUCGGGGGCGAGGGCGAGGUUCAGGCCUUUCA
GGCCGCAGGAAGAGGAACGGAGCGAGUCCCCGCGCGCGGCGCGAUUCCCUGAGCUGUGGG
ACGUGCACCCAGGACUCGGCUCACACAUGCAGUUCGCUUUCCUGUUGGUGGGGGGAACGC
CG

C-A-r-Rc


?

```
Visualisation of predicted secondary structure.
To save the image:
  Right click on the image -> Save Image as.
```


centroid


?

```
Visualisation of predicted secondary structure.
To save the image:
  Right click on the image -> Save Image as.
```


fq-sub


?

```
Visualisation of predicted secondary structure.
To save the image:
  Right click on the image -> Save Image as.
```

Load Sequence viewer

TurboFold: Number of sequences is less then required. n=2 (4)

### Hit: NR\_001566.1

NR\_001566.1 Homo sapiens telomerase RNA component (TERC), telomerase RNA >BC128029.1 Homo sapiens cDNA clone IMAGE:40002477

```
?

```
This is BLAST alignment as read from the input file
```

Score = 54.0 bits (50.0), Expect = 3.95E-02
 Identities = 54/71 (76%), Gaps = 2/71 (3%)
 Strand = Plus/Plus
Query 318 GGCCGCGATCAGCCCGGGCTTTCCCTACTTTGGGGCCCAATGCTGTCGCGAAGAGTTCGGCTCTGTCAGC 387
          ||||||| || |||||||  ||| |  |   ||  |||| |||   ||||||||||| ||||||||||||    
Sbjct 253 GGCCGCGGTCGGCCCGGGGCTTCTC--CGGAGGCACCCACTGCCACCGCGAAGAGTTGGGCTCTGTCAGC 320

Query 388 C 388
          |    
Sbjct 321 C 321
```

Report:

|  |  |
| --- | --- |
| sequence start ?  ``` Start position of the estimated full-length sequence in genome. Start index < end index. ``` : | 1 |
| sequence end ?  ``` End position of the estimated full-length sequence in genome. Start index < end index. ``` : | 451 |
| bit score (CM) ?  ``` The score for aligning estimated full-length sequence to CM model   (computed by RSEARCH -> default,   infered from Rfam or provided by user) ``` : | 10.14 |
| Homology estimate ?  ``` Quick homology estimate:   Not homologous: bit score < 0   Homologous: bit score > 20 and bit score > 0.5 * query length   Uncertain otherwise ``` : | Uncertain |

Estimated full-length sequence:


?

```
Click checkbox to select multiple seuqences.
Fasta header format:
  UID|accession.versionSTRAND start-end
```

>uid:43|NR\_001566.1fw 1-451
GGGUUGCGGAGGGUGGGCCUGGGAGGGGUGGUGGCCAUUUUUUGUCUAACCCUAACUGAG
AAGGGCGUAGGCGCCGUGCUUUUGCUCCCCGCGCGCUGUUUUUCUCGCUGACUUUCAGCG
GGCGGAAAAGCCUCGGCCUGCCGCCUUCCACCGUUCAUUCUAGAGCAAACAAAAAAUGUC
AGCUGCUGGCCCGUUCGCCCCUCCCGGGGACCUGCGGCGGGUCGCCUGCCCAGCCCCCGA
ACCCCGCCUGGAGGCCGCGGUCGGCCCGGGGCUUCUCCGGAGGCACCCACUGCCACCGCG
AAGAGUUGGGCUCUGUCAGCCGCGGGUCUCUCGGGGGCGAGGGCGAGGUUCAGGCCUUUC
AGGCCGCAGGAAGAGGAACGGAGCGAGUCCCCGCGCGCGGCGCGAUUCCCUGAGCUGUGG
GACGUGCACCCAGGACUCGGCUCACACAUGC

C-A-r-Rc


?

```
Visualisation of predicted secondary structure.
To save the image:
  Right click on the image -> Save Image as.
```


centroid


?

```
Visualisation of predicted secondary structure.
To save the image:
  Right click on the image -> Save Image as.
```


fq-sub


?

```
Visualisation of predicted secondary structure.
To save the image:
  Right click on the image -> Save Image as.
```

Load Sequence viewer

TurboFold: Number of sequences is less then required. n=2 (4)  
uid:43|NR\_001566.1: Sequence cannot be extended sufficiently by unaligned portion of query. THIS IS PROBABLY FRAGMENT! Trimmed upstream.  
uid:43|NR\_001566.1: Sequence cannot be extended sufficiently. Missing -94 nt upstream in the genome.  
uid:43|NR\_001566.1: Sequence cannot be extended sufficiently. Missing nt downstream in the genome.

### Hit: NR\_001576.1

NR\_001576.1 Bos taurus telomerase RNA component (TERC), telomerase RNA

```
?

```
This is BLAST alignment as read from the input file
```

Score = 54.0 bits (50.0), Expect = 3.95E-02
 Identities = 68/91 (75%), Gaps = 7/91 (8%)
 Strand = Plus/Plus
Query 318 GGCCGCGATCAGCCCGG-GCTTTCCCTACTTTGGGGCCCAATGCTGTCGCGAAGAGTTCGGCTCTGTCAG 386
          ||||||| || |||||| ||||   || |   || | ||| ||| | || |||||||| |||||||||||    
Sbjct 252 GGCCGCGGTCGGCCCGGTGCTT---CTCCGGAGGTGTCCATTGCCGCCGTGAAGAGTTGGGCTCTGTCAG 318

Query 387 CC-CGGCTGGGTCCGGGTGGG 406
          || ||| | | ||  ||||||    
Sbjct 319 CCGCGGGTCGCTC--GGTGGG 337
```

Report:

|  |  |
| --- | --- |
| sequence start ?  ``` Start position of the estimated full-length sequence in genome. Start index < end index. ``` : | 1 |
| sequence end ?  ``` End position of the estimated full-length sequence in genome. Start index < end index. ``` : | 443 |
| bit score (CM) ?  ``` The score for aligning estimated full-length sequence to CM model   (computed by RSEARCH -> default,   infered from Rfam or provided by user) ``` : | 13.43 |
| Homology estimate ?  ``` Quick homology estimate:   Not homologous: bit score < 0   Homologous: bit score > 20 and bit score > 0.5 * query length   Uncertain otherwise ``` : | Uncertain |

Estimated full-length sequence:


?

```
Click checkbox to select multiple seuqences.
Fasta header format:
  UID|accession.versionSTRAND start-end
```

>uid:44|NR\_001576.1fw 1-443
GGGUUGCGGAGGGUGGGCCCCGGGUUGGUGGCAGCCAUUUCUCAUCUAACCCUAAUUGAG
ACAGGCGUAGGCGCUGUGCUUUUGGUUACCGCGCGCUGUUUUUCUCGCUGACUUUCAGCG
GGCGGAAAAGCCUCGGCCUACCGCCAUCCACCAUCCAGUCUGCAACAAACAAAAAAUGUC
AGCCGCUGGCUCGCUCACCUCUCCCGGGAACCUGCGGUGGUCCGCCCGCCCAGCCCCAGU
GCCCCGCCUGAGGCCGCGGUCGGCCCGGUGCUUCUCCGGAGGUGUCCAUUGCCGCCGUGA
AGAGUUGGGCUCUGUCAGCCGCGGGUCGCUCGGUGGGCCGAGGCAUGGCUGUAACCGCAG
GGAAAGGAACGGAGUGGGGUCCCCGCGCGCGGUGCGCUUCCCUGAGCUGUGGGACUUGCA
CCCGGGACUCGGCUCAGACAUCC

C-A-r-Rc


?

```
Visualisation of predicted secondary structure.
To save the image:
  Right click on the image -> Save Image as.
```


centroid


?

```
Visualisation of predicted secondary structure.
To save the image:
  Right click on the image -> Save Image as.
```


fq-sub


?

```
Visualisation of predicted secondary structure.
To save the image:
  Right click on the image -> Save Image as.
```

Load Sequence viewer

TurboFold: Number of sequences is less then required. n=2 (4)  
uid:44|NR\_001576.1: Sequence cannot be extended sufficiently by unaligned portion of query. THIS IS PROBABLY FRAGMENT! Trimmed upstream.  
uid:44|NR\_001576.1: Sequence cannot be extended sufficiently. Missing nt downstream in the genome.  
uid:44|NR\_001576.1: Sequence cannot be extended sufficiently. Missing -95 nt upstream in the genome.

### Hit: U86046.1

U86046.1 Human telomerase RNA (hTR) gene sequence

```
?

```
This is BLAST alignment as read from the input file
```

Score = 54.0 bits (50.0), Expect = 3.95E-02
 Identities = 54/71 (76%), Gaps = 2/71 (3%)
 Strand = Plus/Plus
Query 318 GGCCGCGATCAGCCCGGGCTTTCCCTACTTTGGGGCCCAATGCTGTCGCGAAGAGTTCGGCTCTGTCAGC 387
          ||||||| || |||||||  ||| |  |   ||  |||| |||   ||||||||||| ||||||||||||    
Sbjct 347 GGCCGCGGTCGGCCCGGGGCTTCTC--CGGAGGCACCCACTGCCACCGCGAAGAGTTGGGCTCTGTCAGC 414

Query 388 C 388
          |    
Sbjct 415 C 415
```

Report:

|  |  |
| --- | --- |
| sequence start ?  ``` Start position of the estimated full-length sequence in genome. Start index < end index. ``` : | 29 |
| sequence end ?  ``` End position of the estimated full-length sequence in genome. Start index < end index. ``` : | 545 |
| bit score (CM) ?  ``` The score for aligning estimated full-length sequence to CM model   (computed by RSEARCH -> default,   infered from Rfam or provided by user) ``` : | 9.69 |
| Homology estimate ?  ``` Quick homology estimate:   Not homologous: bit score < 0   Homologous: bit score > 20 and bit score > 0.5 * query length   Uncertain otherwise ``` : | Uncertain |

Estimated full-length sequence:


?

```
Click checkbox to select multiple seuqences.
Fasta header format:
  UID|accession.versionSTRAND start-end
```

>uid:45|U86046.1fw 29-545
CAGCUUGGCCAAUCCGUGCGGUCGGCGGCCGCUCCCUUUAUAAGCCGACUCGCCCGGCAG
CGCACCGGGUUGCGGAGGGUGGGCCUGGGAGGGGUGGUGGCCAUUUUUUGUCUAACCCUA
ACUGAGAAGGGCGUAGGCGCCGUGCUUUUGCUCCCCGCGCGCUGUUUUUCUCGCUGACUU
UCAGCGGGCGGAAAAGCCUCGGCCUGCCGCCUUCCACCGUUCAUUCUAGAGCAAACAAAA
AAUGUCAGCUGCUGGCCCGUUCGCCCCUCCCGGGGACCUGCGGCGGGUCGCCUGCCCAGC
CCCCGAACCCCGCCUGGAGGCCGCGGUCGGCCCGGGGCUUCUCCGGAGGCACCCACUGCC
ACCGCGAAGAGUUGGGCUCUGUCAGCCGCGGGUCUCUCGGGGGCGAGGGCGAGGUUCAGG
CCUUUCAGGCCGCAGGAAGAGGAACGGAGCGAGUCCCCGCGCGCGGCGCGAUUCCCUGAG
CUGUGGGACGUGCACCCAGGACUCGGCUCACACAUGC

C-A-r-Rc


?

```
Visualisation of predicted secondary structure.
To save the image:
  Right click on the image -> Save Image as.
```


centroid


?

```
Visualisation of predicted secondary structure.
To save the image:
  Right click on the image -> Save Image as.
```


fq-sub


?

```
Visualisation of predicted secondary structure.
To save the image:
  Right click on the image -> Save Image as.
```

Load Sequence viewer

TurboFold: Number of sequences is less then required. n=2 (4)  
uid:45|U86046.1: Sequence cannot be extended sufficiently. Missing 0 nt upstream in the genome.  
uid:45|U86046.1: Sequence cannot be extended sufficiently. Missing nt downstream in the genome.

### Hit: AC078802.14

AC078802.14 Homo sapiens 3 BAC RP11-816J6 (Roswell Park Cancer Institute Human BAC Library) complete sequence

```
?

```
This is BLAST alignment as read from the input file
```

Score = 54.0 bits (50.0), Expect = 3.95E-02
 Identities = 54/71 (76%), Gaps = 2/71 (3%)
 Strand = Plus/Minus
Query   318 GGCCGCGATCAGCCCGGGCTTTCCCTACTTTGGGGCCCAATGCTGTCGCGAAGAGTTCGGCTCTGT 383  
            ||||||| || |||||||  ||| |  |   ||  |||| |||   ||||||||||| ||||||||      
Sbjct 35344 GGCCGCGGTCGGCCCGGGGCTTCTC--CGGAGGCACCCACTGCCACCGCGAAGAGTTGGGCTCTGT 35281

Query   384 CAGCC 388  
            |||||      
Sbjct 35280 CAGCC 35276
```

Report:

|  |  |
| --- | --- |
| sequence start ?  ``` Start position of the estimated full-length sequence in genome. Start index < end index. ``` : | 35122 |
| sequence end ?  ``` End position of the estimated full-length sequence in genome. Start index < end index. ``` : | 35663 |
| bit score (CM) ?  ``` The score for aligning estimated full-length sequence to CM model   (computed by RSEARCH -> default,   infered from Rfam or provided by user) ``` : | 4.6 |
| Homology estimate ?  ``` Quick homology estimate:   Not homologous: bit score < 0   Homologous: bit score > 20 and bit score > 0.5 * query length   Uncertain otherwise ``` : | Uncertain |

Estimated full-length sequence:


?

```
Click checkbox to select multiple seuqences.
Fasta header format:
  UID|accession.versionSTRAND start-end
```

>uid:46|AC078802.14rc 35122-35663
GCCAAUCCGUGCGGUCGGCGGCCGCUCCCUUUAUAAGCCGACUCGCCCGGCAGCGCACCG
GGUUGCGGAGGGUGGGCCUGGGAGGGGUGGUGGCCAUUUUUUGUCUAACCCUAACUGAGA
AGGGCGUAGGCGCCGUGCUUUUGCUCCCCGCGCGCUGUUUUUCUCGCUGACUUUCAGCGG
GCGGAAAAGCCUCGGCCUGCCGCCUUCCACCGUUCAUUCUAGAGCAAACAAAAAAUGUCA
GCUGCUGGCCCGUUCGCCCCUCCCGGGGACCUGCGGCGGGUCGCCUGCCCAGCCCCCGAA
CCCCGCCUGGAGGCCGCGGUCGGCCCGGGGCUUCUCCGGAGGCACCCACUGCCACCGCGA
AGAGUUGGGCUCUGUCAGCCGCGGGUCUCUCGGGGGCGAGGGCGAGGUUCAGGCCUUUCA
GGCCGCAGGAAGAGGAACGGAGCGAGUCCCCGCGCGCGGCGCGAUUCCCUGAGCUGUGGG
ACGUGCACCCAGGACUCGGCUCACACAUGCAGUUCGCUUUCCUGUUGGUGGGGGGAACGC
CG

C-A-r-Rc


?

```
Visualisation of predicted secondary structure.
To save the image:
  Right click on the image -> Save Image as.
```


centroid


?

```
Visualisation of predicted secondary structure.
To save the image:
  Right click on the image -> Save Image as.
```


fq-sub


?

```
Visualisation of predicted secondary structure.
To save the image:
  Right click on the image -> Save Image as.
```

Load Sequence viewer

TurboFold: Number of sequences is less then required. n=2 (4)

### Hit: AF221936.1

AF221936.1 Bos taurus telomerase RNA gene, sequence

```
?

```
This is BLAST alignment as read from the input file
```

Score = 54.0 bits (50.0), Expect = 3.95E-02
 Identities = 68/91 (75%), Gaps = 7/91 (8%)
 Strand = Plus/Plus
Query 318 GGCCGCGATCAGCCCGG-GCTTTCCCTACTTTGGGGCCCAATGCTGTCGCGAAGAGTTCGGCTCTGTCAG 386
          ||||||| || |||||| ||||   || |   || | ||| ||| | || |||||||| |||||||||||    
Sbjct 349 GGCCGCGGTCGGCCCGGTGCTT---CTCCGGAGGTGTCCATTGCCGCCGTGAAGAGTTGGGCTCTGTCAG 415

Query 387 CC-CGGCTGGGTCCGGGTGGG 406
          || ||| | | ||  ||||||    
Sbjct 416 CCGCGGGTCGCTC--GGTGGG 434
```

Report:

|  |  |
| --- | --- |
| sequence start ?  ``` Start position of the estimated full-length sequence in genome. Start index < end index. ``` : | 37 |
| sequence end ?  ``` End position of the estimated full-length sequence in genome. Start index < end index. ``` : | 540 |
| bit score (CM) ?  ``` The score for aligning estimated full-length sequence to CM model   (computed by RSEARCH -> default,   infered from Rfam or provided by user) ``` : | 14.79 |
| Homology estimate ?  ``` Quick homology estimate:   Not homologous: bit score < 0   Homologous: bit score > 20 and bit score > 0.5 * query length   Uncertain otherwise ``` : | Uncertain |

Estimated full-length sequence:


?

```
Click checkbox to select multiple seuqences.
Fasta header format:
  UID|accession.versionSTRAND start-end
```

>uid:47|AF221936.1fw 37-540
CGGCCAAUCAGCGUAGGCGCCGGCUGCUGCAUUCAUAAGGAGACGCACCCAGCGGCGCGG
CGGGUUGCGGAGGGUGGGCCCCGGGUUGGUGGCAGCCAUUUCUCAUCUAACCCUAAUUGA
GACAGGCGUAGGCGCUGUGCUUUUGGUUACCGCGCGCUGUUUUUCUCGCUGACUUUCAGC
GGGCGGAAAAGCCUCGGCCUACCGCCAUCCACCAUCCAGUCUGCAACAAACAAAAAAUGU
CAGCCGCUGGCUCGCUCACCUCUCCCGGGAACCUGCGGUGGUCCGCCCGCCCAGCCCCAG
UGCCCCGCCUGAGGCCGCGGUCGGCCCGGUGCUUCUCCGGAGGUGUCCAUUGCCGCCGUG
AAGAGUUGGGCUCUGUCAGCCGCGGGUCGCUCGGUGGGCCGAGGCAUGGCUGUAACCGCA
GGGAAAGGAACGGAGUGGGGUCCCCGCGCGCGGUGCGCUUCCCUGAGCUGUGGGACUUGC
ACCCGGGACUCGGCUCAGACAUCC

C-A-r-Rc


?

```
Visualisation of predicted secondary structure.
To save the image:
  Right click on the image -> Save Image as.
```


centroid


?

```
Visualisation of predicted secondary structure.
To save the image:
  Right click on the image -> Save Image as.
```


fq-sub


?

```
Visualisation of predicted secondary structure.
To save the image:
  Right click on the image -> Save Image as.
```

Load Sequence viewer

TurboFold: Number of sequences is less then required. n=2 (4)  
uid:47|AF221936.1: Sequence cannot be extended sufficiently. Missing nt downstream in the genome.  
uid:47|AF221936.1: Sequence cannot be extended sufficiently by unalined portion of query. THIS IS PROBABLY FRAGMENT! Trimmed downstream.

### Hit: AF221923.1

AF221923.1 Trichechus manatus telomerase RNA gene, sequence

```
?

```
This is BLAST alignment as read from the input file
```

Score = 54.0 bits (50.0), Expect = 3.95E-02
 Identities = 54/71 (76%), Gaps = 2/71 (3%)
 Strand = Plus/Plus
Query 318 GGCCGCGATCAGCCCGGGCTTTCCCTACTTTGGGGCCCAATGCTGTCGCGAAGAGTTCGGCTCTGTCAGC 387
          ||||||| || ||| |||  ||| |  |   ||  |||| ||| | ||||||||||| ||||||||||||    
Sbjct 357 GGCCGCGGTCGGCCTGGGGCTTCTC--CGGAGGTTCCCATTGCCGCCGCGAAGAGTTAGGCTCTGTCAGC 424

Query 388 C 388
          |    
Sbjct 425 C 425
```

Report:

|  |  |
| --- | --- |
| sequence start ?  ``` Start position of the estimated full-length sequence in genome. Start index < end index. ``` : | 40 |
| sequence end ?  ``` End position of the estimated full-length sequence in genome. Start index < end index. ``` : | 554 |
| bit score (CM) ?  ``` The score for aligning estimated full-length sequence to CM model   (computed by RSEARCH -> default,   infered from Rfam or provided by user) ``` : | 7.5600000000000005 |
| Homology estimate ?  ``` Quick homology estimate:   Not homologous: bit score < 0   Homologous: bit score > 20 and bit score > 0.5 * query length   Uncertain otherwise ``` : | Uncertain |

Estimated full-length sequence:


?

```
Click checkbox to select multiple seuqences.
Fasta header format:
  UID|accession.versionSTRAND start-end
```

>uid:48|AF221923.1fw 40-554
GCCAAUCCGCGCGGGCGGCGGCCUCUCCCUUUAUAAGGAGACUUGGCGCGCGAGGCUUGG
CGUGGAGGGUUGAGGAUGGCGCCCCCGGGUCGGGCAGUGGUCUUUUUUGUUCUAACCCUA
ACUGGCAAGGGCGUAGGUGCUGUGCUUUUGUUCCCCGCGCGUUGUUUUUCUCGCUGACUU
UCAGCGGGCGGGAAAAGCCCUGGCCUACCGCCGUCUACCGAUAGUUUGGAGCAAACAAAA
AAAUGUCAGCCGCUGGCCGCUCACCCCUCCCGGGAACCUGUGGUGGCUCGCCCGCCCAGC
CCCGCGCCCCGCCUGGAGGCCGCGGUCGGCCUGGGGCUUCUCCGGAGGUUCCCAUUGCCG
CCGCGAAGAGUUAGGCUCUGUCAGCCGCGGGUCCUGCGGGAACCAAGGGCGAGGCUUAGG
CCUCCUGAACGCAGGGAGAGAAAUGGAGCGAUUCCCCGAGUACGUGUGCUUCCCUGAGUU
GUGGGAUGUGCGUCCGGGACUCAGCUCCGACAGGU

C-A-r-Rc


?

```
Visualisation of predicted secondary structure.
To save the image:
  Right click on the image -> Save Image as.
```


centroid


?

```
Visualisation of predicted secondary structure.
To save the image:
  Right click on the image -> Save Image as.
```


fq-sub


?

```
Visualisation of predicted secondary structure.
To save the image:
  Right click on the image -> Save Image as.
```

Load Sequence viewer

TurboFold: Number of sequences is less then required. n=2 (4)  
uid:48|AF221923.1: Sequence cannot be extended sufficiently by unalined portion of query. THIS IS PROBABLY FRAGMENT! Trimmed downstream.  
uid:48|AF221923.1: Sequence cannot be extended sufficiently. Missing nt downstream in the genome.

### Hit: AF221907.1

AF221907.1 Homo sapiens telomerase RNA gene, sequence

```
?

```
This is BLAST alignment as read from the input file
```

Score = 54.0 bits (50.0), Expect = 3.95E-02
 Identities = 54/71 (76%), Gaps = 2/71 (3%)
 Strand = Plus/Plus
Query 318 GGCCGCGATCAGCCCGGGCTTTCCCTACTTTGGGGCCCAATGCTGTCGCGAAGAGTTCGGCTCTGTCAGC 387
          ||||||| || |||||||  ||| |  |   ||  |||| |||   ||||||||||| ||||||||||||    
Sbjct 350 GGCCGCGGTCGGCCCGGGGCTTCTC--CGGAGGCACCCACTGCCACCGCGAAGAGTTGGGCTCTGTCAGC 417

Query 388 C 388
          |    
Sbjct 418 C 418
```

Report:

|  |  |
| --- | --- |
| sequence start ?  ``` Start position of the estimated full-length sequence in genome. Start index < end index. ``` : | 32 |
| sequence end ?  ``` End position of the estimated full-length sequence in genome. Start index < end index. ``` : | 548 |
| bit score (CM) ?  ``` The score for aligning estimated full-length sequence to CM model   (computed by RSEARCH -> default,   infered from Rfam or provided by user) ``` : | 9.69 |
| Homology estimate ?  ``` Quick homology estimate:   Not homologous: bit score < 0   Homologous: bit score > 20 and bit score > 0.5 * query length   Uncertain otherwise ``` : | Uncertain |

Estimated full-length sequence:


?

```
Click checkbox to select multiple seuqences.
Fasta header format:
  UID|accession.versionSTRAND start-end
```

>uid:49|AF221907.1fw 32-548
CAGCUUGGCCAAUCCGUGCGGUCGGCGGCCGCUCCCUUUAUAAGCCGACUCGCCCGGCAG
CGCACCGGGUUGCGGAGGGUGGGCCUGGGAGGGGUGGUGGCCAUUUUUUGUCUAACCCUA
ACUGAGAAGGGCGUAGGCGCCGUGCUUUUGCUCCCCGCGCGCUGUUUUUCUCGCUGACUU
UCAGCGGGCGGAAAAGCCUCGGCCUGCCGCCUUCCACCGUUCAUUCUAGAGCAAACAAAA
AAUGUCAGCUGCUGGCCCGUUCGCCCCUCCCGGGGACCUGCGGCGGGUCGCCUGCCCAGC
CCCCGAACCCCGCCUGGAGGCCGCGGUCGGCCCGGGGCUUCUCCGGAGGCACCCACUGCC
ACCGCGAAGAGUUGGGCUCUGUCAGCCGCGGGUCUCUCGGGGGCGAGGGCGAGGUUCAGG
CCUUUCAGGCCGCAGGAAGAGGAACGGAGCGAGUCCCCGCGCGCGGCGCGAUUCCCUGAG
CUGUGGGACGUGCACCCAGGACUCGGCUCACACAUGC

C-A-r-Rc


?

```
Visualisation of predicted secondary structure.
To save the image:
  Right click on the image -> Save Image as.
```


centroid


?

```
Visualisation of predicted secondary structure.
To save the image:
  Right click on the image -> Save Image as.
```


fq-sub


?

```
Visualisation of predicted secondary structure.
To save the image:
  Right click on the image -> Save Image as.
```

Load Sequence viewer

TurboFold: Number of sequences is less then required. n=2 (4)  
uid:49|AF221907.1: Sequence cannot be extended sufficiently. Missing nt downstream in the genome.  
uid:49|AF221907.1: Sequence cannot be extended sufficiently by unalined portion of query. THIS IS PROBABLY FRAGMENT! Trimmed downstream.

### Hit: U85256.1

U85256.1 Human telomerase RNA, partial sequence

```
?

```
This is BLAST alignment as read from the input file
```

Score = 54.0 bits (50.0), Expect = 3.95E-02
 Identities = 54/71 (76%), Gaps = 2/71 (3%)
 Strand = Plus/Plus
Query 318 GGCCGCGATCAGCCCGGGCTTTCCCTACTTTGGGGCCCAATGCTGTCGCGAAGAGTTCGGCTCTGTCAGC 387
          ||||||| || |||||||  ||| |  |   ||  |||| |||   ||||||||||| ||||||||||||    
Sbjct 253 GGCCGCGGTCGGCCCGGGGCTTCTC--CGGAGGCACCCACTGCCACCGCGAAGAGTTGGGCTCTGTCAGC 320

Query 388 C 388
          |    
Sbjct 321 C 321
```

Report:

|  |  |
| --- | --- |
| sequence start ?  ``` Start position of the estimated full-length sequence in genome. Start index < end index. ``` : | 1 |
| sequence end ?  ``` End position of the estimated full-length sequence in genome. Start index < end index. ``` : | 511 |
| bit score (CM) ?  ``` The score for aligning estimated full-length sequence to CM model   (computed by RSEARCH -> default,   infered from Rfam or provided by user) ``` : | -6.06 |
| Homology estimate ?  ``` Quick homology estimate:   Not homologous: bit score < 0   Homologous: bit score > 20 and bit score > 0.5 * query length   Uncertain otherwise ``` : | Not homologous |

Estimated full-length sequence:


?

```
Click checkbox to select multiple seuqences.
Fasta header format:
  UID|accession.versionSTRAND start-end
```

>uid:50|U85256.1fw 1-511
GGGUUGCGGAGGGUGGGCCUGGGAGGGGUGGUGGCCAUUUUUUGUCUAACCCUAACUGAG
AAGGGCGUAGGCGCCGUGCUUUUGCUCCCCGCGCGCUGUUUUUCUCGCUGACUUUCAGCG
GGCGGAAAAGCCUCGGCCUGCCGCCUUCCACCGUUCAUUCUAGAGCAAACAAAAAAUGUC
AGCUGCUGGCCCGUUCGCCCCUCCCGGGGACCUGCGGCGGGUCGCCUGCCCAGCCCCCGA
ACCCCGCCUGGAGGCCGCGGUCGGCCCGGGGCUUCUCCGGAGGCACCCACUGCCACCGCG
AAGAGUUGGGCUCUGUCAGCCGCGGGUCUCUCGGGGGCGAGGGCGAGGUUCAGGCCUUUC
AGGCCGCAGGAAGAGGAACGGAGCGAGUCCCCGCGCGCGGCGCGAUUCCCUGAGCUGUGG
GACGUGCACCCAGGACUCGGCUCACACAUGCAGUUCGCUUUCCUGUUGGUGGGGGGAACG
CCGAUCGUGCGCAUCCGUCACCCCUCGCCGG

C-A-r-Rc


?

```
Visualisation of predicted secondary structure.
To save the image:
  Right click on the image -> Save Image as.
```


centroid


?

```
Visualisation of predicted secondary structure.
To save the image:
  Right click on the image -> Save Image as.
```


fq-sub


?

```
Visualisation of predicted secondary structure.
To save the image:
  Right click on the image -> Save Image as.
```

Load Sequence viewer

TurboFold: Number of sequences is less then required. n=2 (4)  
uid:50|U85256.1: Sequence cannot be extended sufficiently. Missing -94 nt upstream in the genome.  
uid:50|U85256.1: Sequence cannot be extended sufficiently by unaligned portion of query. THIS IS PROBABLY FRAGMENT! Trimmed upstream.

### Hit: AY312571.1

AY312571.1 Gallus gallus gallus telomerase RNA gene, complete sequence

```
?

```
This is BLAST alignment as read from the input file
```

Score = 52.0 bits (48.2), Expect = 1.38E-01
 Identities = 43/53 (81%), Gaps = 1/53 (2%)
 Strand = Plus/Plus
Query 354 CCAATGCTGTCGCGAAGAGTTCGGCTCTGTCAGCC-CGGCTGGGTCCGGGTGG 405
          ||| ||| | ||||||||||||| ||||||||||| |||| | |  |||| ||    
Sbjct 906 CCATTGCCGCCGCGAAGAGTTCGCCTCTGTCAGCCTCGGCGGCGCGCGGGAGG 958
```

Report:

|  |  |
| --- | --- |
| sequence start ?  ``` Start position of the estimated full-length sequence in genome. Start index < end index. ``` : | 605 |
| sequence end ?  ``` End position of the estimated full-length sequence in genome. Start index < end index. ``` : | 1091 |
| bit score (CM) ?  ``` The score for aligning estimated full-length sequence to CM model   (computed by RSEARCH -> default,   infered from Rfam or provided by user) ``` : | 19.75 |
| Homology estimate ?  ``` Quick homology estimate:   Not homologous: bit score < 0   Homologous: bit score > 20 and bit score > 0.5 * query length   Uncertain otherwise ``` : | Uncertain |

Estimated full-length sequence:


?

```
Click checkbox to select multiple seuqences.
Fasta header format:
  UID|accession.versionSTRAND start-end
```

>uid:51|AY312571.1fw 605-1091
ACGCGUGGCGGGUGGAAGGCUCCGCUGUGCCUAACCCUAAUCGGGGGAAUUGAUGGUGCU
GUCGCCGCGCUCCCUCCGCCCGCCCGCUGUUUUACUCGCUGACUUUCAGCGGGCGAGAGG
AGCCGCCCCGGGGGGGAGGCGGGCGGCGGGAGGGGGCCGGGGCGCCGCGGCGGUGGGGGU
CGGGGGGGGGAGAGAAAGGGCCGAAAGGGGCUCCGCGGCCAAAAAAACGUCAGCGAGGGG
UCCGCUCGCCCCGAUCCGCCCUGGGGUCCCCGCUCGCGUGGCCGCGGUCGGCCGGCACCC
GCCAUUGCCGCCGCGAAGAGUUCGCCUCUGUCAGCCUCGGCGGCGCGCGGGAGGUGCGGC
GCGCGGCCCCGCGCCCCCAGCAGAGCAAACGGGAGCGGCGCCCCCGGGGUAACCCCCGCG
CUCCCCUGCGCCGUGGGGCGCGCGGACGGCGUCGCUCCCACACGCGCGGCCCCGCGCGCA
CGACCGU

C-A-r-Rc


?

```
Visualisation of predicted secondary structure.
To save the image:
  Right click on the image -> Save Image as.
```


centroid


?

```
Visualisation of predicted secondary structure.
To save the image:
  Right click on the image -> Save Image as.
```


fq-sub


?

```
Visualisation of predicted secondary structure.
To save the image:
  Right click on the image -> Save Image as.
```

Load Sequence viewer

TurboFold: Number of sequences is less then required. n=2 (4)

### Hit: NR\_001594.1

NR\_001594.1 Gallus gallus telomerase RNA component (TERC), telomerase RNA

```
?

```
This is BLAST alignment as read from the input file
```

Score = 52.0 bits (48.2), Expect = 1.38E-01
 Identities = 43/53 (81%), Gaps = 1/53 (2%)
 Strand = Plus/Plus
Query 354 CCAATGCTGTCGCGAAGAGTTCGGCTCTGTCAGCC-CGGCTGGGTCCGGGTGG 405
          ||| ||| | ||||||||||||| ||||||||||| |||| | |  |||| ||    
Sbjct 302 CCATTGCCGCCGCGAAGAGTTCGCCTCTGTCAGCCTCGGCGGCGCGCGGGAGG 354
```

Report:

|  |  |
| --- | --- |
| sequence start ?  ``` Start position of the estimated full-length sequence in genome. Start index < end index. ``` : | 1 |
| sequence end ?  ``` End position of the estimated full-length sequence in genome. Start index < end index. ``` : | 465 |
| bit score (CM) ?  ``` The score for aligning estimated full-length sequence to CM model   (computed by RSEARCH -> default,   infered from Rfam or provided by user) ``` : | 16.25 |
| Homology estimate ?  ``` Quick homology estimate:   Not homologous: bit score < 0   Homologous: bit score > 20 and bit score > 0.5 * query length   Uncertain otherwise ``` : | Uncertain |

Estimated full-length sequence:


?

```
Click checkbox to select multiple seuqences.
Fasta header format:
  UID|accession.versionSTRAND start-end
```

>uid:52|NR\_001594.1fw 1-465
ACGCGUGGCGGGUGGAAGGCUCCGCUGUGCCUAACCCUAAUCGGGGGAAUUGAUGGUGCU
GUCGCCGCGCUCCCUCCGCCCGCCCGCUGUUUUACUCGCUGACUUUCAGCGGGCGAGAGG
AGCCGCCCCGGGGGGGAGGCGGGCGGCGGGAGGGGGCCGGGGCGCCGCGGCGGUGGGGGU
CGGGGGGGGGAGAGAAAGGGCCGAAAGGGGCUCCGCGGCCAAAAAAACGUCAGCGAGGGG
UCCGCUCGCCCCGAUCCGCCCUGGGGUCCCCGCUCGCGUGGCCGCGGUCGGCCGGCACCC
GCCAUUGCCGCCGCGAAGAGUUCGCCUCUGUCAGCCUCGGCGGCGCGCGGGAGGUGCGGC
GCGCGGCCCCGCGCCCCCAGCAGAGCAAACGGGAGCGGCGCCCCCGGGGUAACCCCCGCG
CUCCCCUGCGCCGUGGGGCGCGCGGACGGCGUCGCUCCCACACGC

C-A-r-Rc


?

```
Visualisation of predicted secondary structure.
To save the image:
  Right click on the image -> Save Image as.
```


centroid


?

```
Visualisation of predicted secondary structure.
To save the image:
  Right click on the image -> Save Image as.
```


fq-sub


?

```
Visualisation of predicted secondary structure.
To save the image:
  Right click on the image -> Save Image as.
```

Load Sequence viewer

TurboFold: Number of sequences is less then required. n=2 (4)  
uid:52|NR\_001594.1: Sequence cannot be extended sufficiently by unaligned portion of query. THIS IS PROBABLY FRAGMENT! Trimmed upstream.  
uid:52|NR\_001594.1: Sequence cannot be extended sufficiently. Missing -81 nt upstream in the genome.  
uid:52|NR\_001594.1: Sequence cannot be extended sufficiently. Missing nt downstream in the genome.

### Hit: AY626232.1

AY626232.1 Gallus gallus telomerase RNA gene, partial sequence

```
?

```
This is BLAST alignment as read from the input file
```

Score = 52.0 bits (48.2), Expect = 1.38E-01
 Identities = 43/53 (81%), Gaps = 1/53 (2%)
 Strand = Plus/Plus
Query 354 CCAATGCTGTCGCGAAGAGTTCGGCTCTGTCAGCC-CGGCTGGGTCCGGGTGG 405
          ||| ||| | ||||||||||||| ||||||||||| |||| | |  |||| ||    
Sbjct 232 CCATTGCCGCCGCGAAGAGTTCGCCTCTGTCAGCCTCGGCGGCGCGCGGGAGG 284
```

Report:

|  |  |
| --- | --- |
| sequence start ?  ``` Start position of the estimated full-length sequence in genome. Start index < end index. ``` : | 1 |
| sequence end ?  ``` End position of the estimated full-length sequence in genome. Start index < end index. ``` : | 331 |
| bit score (CM) ?  ``` The score for aligning estimated full-length sequence to CM model   (computed by RSEARCH -> default,   infered from Rfam or provided by user) ``` : | -5.11 |
| Homology estimate ?  ``` Quick homology estimate:   Not homologous: bit score < 0   Homologous: bit score > 20 and bit score > 0.5 * query length   Uncertain otherwise ``` : | Not homologous |

Estimated full-length sequence:


?

```
Click checkbox to select multiple seuqences.
Fasta header format:
  UID|accession.versionSTRAND start-end
```

>uid:53|AY626232.1fw 1-331
AGGCUCCGCUGUGCCUAACCCUAAUCGGGGGAAUUGAUGGUGCUGUCGCCGCGCUCCCUC
CGCCCGCCCGCUGUUUUACUCGCUGACUUUCAGCGGGCGAGAGGAGCCGCCCCGGGGGGG
GAGAGAAAGGGCCGAAAGGGGCUCCGCGGCCAAAAAACGUCAGCGAGGGGUCCGCUCGCC
CCGAUCCGCCCUGGGGUCCCCGCUCGCGUGGCCGCGGUCGGCCGGCACCCGCCAUUGCCG
CCGCGAAGAGUUCGCCUCUGUCAGCCUCGGCGGCGCGCGGGAGGUGCGGCGCGCGGCCCC
GCGCCCCCAGCAGAGCAAACGGGAGCGGCGC

C-A-r-Rc


?

```
Visualisation of predicted secondary structure.
To save the image:
  Right click on the image -> Save Image as.
```


centroid


?

```
Visualisation of predicted secondary structure.
To save the image:
  Right click on the image -> Save Image as.
```


fq-sub


?

```
Visualisation of predicted secondary structure.
To save the image:
  Right click on the image -> Save Image as.
```

Load Sequence viewer

TurboFold: Number of sequences is less then required. n=2 (4)  
uid:53|AY626232.1: Sequence cannot be extended sufficiently. Missing nt downstream in the genome.  
uid:53|AY626232.1: Sequence cannot be extended sufficiently by unaligned portion of query. THIS IS PROBABLY FRAGMENT! Trimmed upstream.  
uid:53|AY626232.1: Sequence cannot be extended sufficiently. Missing -151 nt upstream in the genome.

### Hit: AF221938.1

AF221938.1 Gallus gallus telomerase RNA gene, sequence

```
?

```
This is BLAST alignment as read from the input file
```

Score = 52.0 bits (48.2), Expect = 1.38E-01
 Identities = 43/53 (81%), Gaps = 1/53 (2%)
 Strand = Plus/Plus
Query 354 CCAATGCTGTCGCGAAGAGTTCGGCTCTGTCAGCC-CGGCTGGGTCCGGGTGG 405
          ||| ||| | ||||||||||||| ||||||||||| |||| | |  |||| ||    
Sbjct 406 CCATTGCCGCCGCGAAGAGTTCGCCTCTGTCAGCCTCGGCGGCGCGCGGGAGG 458
```

Report:

|  |  |
| --- | --- |
| sequence start ?  ``` Start position of the estimated full-length sequence in genome. Start index < end index. ``` : | 105 |
| sequence end ?  ``` End position of the estimated full-length sequence in genome. Start index < end index. ``` : | 569 |
| bit score (CM) ?  ``` The score for aligning estimated full-length sequence to CM model   (computed by RSEARCH -> default,   infered from Rfam or provided by user) ``` : | 16.25 |
| Homology estimate ?  ``` Quick homology estimate:   Not homologous: bit score < 0   Homologous: bit score > 20 and bit score > 0.5 * query length   Uncertain otherwise ``` : | Uncertain |

Estimated full-length sequence:


?

```
Click checkbox to select multiple seuqences.
Fasta header format:
  UID|accession.versionSTRAND start-end
```

>uid:54|AF221938.1fw 105-569
ACGCGUGGCGGGUGGAAGGCUCCGCUGUGCCUAACCCUAAUCGGGGGAAUUGAUGGUGCU
GUCGCCGCGCUCCCUCCGCCCGCCCGCUGUUUUACUCGCUGACUUUCAGCGGGCGAGAGG
AGCCGCCCCGGGGGGGAGGCGGGCGGCGGGAGGGGGCCGGGGCGCCGCGGCGGUGGGGGU
CGGGGGGGGGAGAGAAAGGGCCGAAAGGGGCUCCGCGGCCAAAAAAACGUCAGCGAGGGG
UCCGCUCGCCCCGAUCCGCCCUGGGGUCCCCGCUCGCGUGGCCGCGGUCGGCCGGCACCC
GCCAUUGCCGCCGCGAAGAGUUCGCCUCUGUCAGCCUCGGCGGCGCGCGGGAGGUGCGGC
GCGCGGCCCCGCGCCCCCAGCAGAGCAAACGGGAGCGGCGCCCCCGGGGUAACCCCCGCG
CUCCCCUGCGCCGUGGGGCGCGCGGACGGCGUCGCUCCCACACGC

C-A-r-Rc


?

```
Visualisation of predicted secondary structure.
To save the image:
  Right click on the image -> Save Image as.
```


centroid


?

```
Visualisation of predicted secondary structure.
To save the image:
  Right click on the image -> Save Image as.
```


fq-sub


?

```
Visualisation of predicted secondary structure.
To save the image:
  Right click on the image -> Save Image as.
```

Load Sequence viewer

TurboFold: Number of sequences is less then required. n=2 (4)  
uid:54|AF221938.1: Sequence cannot be extended sufficiently. Missing nt downstream in the genome.  
uid:54|AF221938.1: Sequence cannot be extended sufficiently by unalined portion of query. THIS IS PROBABLY FRAGMENT! Trimmed downstream.

### Hit: AF221929.1

AF221929.1 Cavia porcellus telomerase RNA gene, sequence

```
?

```
This is BLAST alignment as read from the input file
```

Score = 52.0 bits (48.2), Expect = 1.38E-01
 Identities = 53/70 (76%), Gaps = 2/70 (3%)
 Strand = Plus/Plus
Query 318 GGCCGCGATCAGCCCGGGCTTTCCCTACTTTGGGGCCCAATGCTGTCGCGAAGAGTTCGGCTCTGTCAGC 387
          |||||||  | || ||||  ||||||     || |||||  || | ||||||||||||| ||||||||||    
Sbjct 325 GGCCGCGGCCGGCGCGGGGCTTCCCTGGA--GGCGCCCATGGCCGCCGCGAAGAGTTCGTCTCTGTCAGC 392
```

Report:

|  |  |
| --- | --- |
| sequence start ?  ``` Start position of the estimated full-length sequence in genome. Start index < end index. ``` : | 18 |
| sequence end ?  ``` End position of the estimated full-length sequence in genome. Start index < end index. ``` : | 517 |
| bit score (CM) ?  ``` The score for aligning estimated full-length sequence to CM model   (computed by RSEARCH -> default,   infered from Rfam or provided by user) ``` : | 16.23 |
| Homology estimate ?  ``` Quick homology estimate:   Not homologous: bit score < 0   Homologous: bit score > 20 and bit score > 0.5 * query length   Uncertain otherwise ``` : | Uncertain |

Estimated full-length sequence:


?

```
Click checkbox to select multiple seuqences.
Fasta header format:
  UID|accession.versionSTRAND start-end
```

>uid:55|AF221929.1fw 18-517
CGACAGCCGUGGCAGGCGUCAGCCAAUCCGCGCGGGCGCCGACCACUGUUUUAUAAGGAG
CCUCUGCGAGCCGCUGGGCCGGGAGGGGUGGUGGUCUUCCCUGUCUAACCCUAAGGUGAA
GAGGACGUGGGUGCCGUGUUUUUCGCUCCCGCACGCUGUUUUUCUCGCUGACUUUCAGCG
UGCAGAAAAGCCUUGGCCUACCGUCGGUUAUUGUCUAAUUAGAAGCAAACAAAAAAUGUC
AGCGUGGCCGGGCCGCCCCUCCCGGAUACCUGCGGCGGCUCGUCCACCGGCCCCCGAGCC
CCGCCUAGGCCGCGGCCGGCGCGGGGCUUCCCUGGAGGCGCCCAUGGCCGCCGCGAAGAG
UUCGUCUCUGUCAGCUGCGGGUCGCCCGGGGGCCGCGGGAGAGUCCCAGGCCUUGGCCGC
AGGGAGAGAAACGGAGCAGGUCCUCGCGCGGUGCACUCCCCUGAGCUGUGGGAAGUGCAC
CGGGACGGGCUCCUACAAGC

C-A-r-Rc


?

```
Visualisation of predicted secondary structure.
To save the image:
  Right click on the image -> Save Image as.
```


centroid


?

```
Visualisation of predicted secondary structure.
To save the image:
  Right click on the image -> Save Image as.
```


fq-sub


?

```
Visualisation of predicted secondary structure.
To save the image:
  Right click on the image -> Save Image as.
```

Load Sequence viewer

uid:55|AF221929.1: Sequence cannot be extended sufficiently. Missing -22 nt upstream in the genome.  
TurboFold: Number of sequences is less then required. n=2 (4)  
uid:55|AF221929.1: Sequence cannot be extended sufficiently. Missing nt downstream in the genome.  
uid:55|AF221929.1: Sequence cannot be extended sufficiently by unalined portion of query. THIS IS PROBABLY FRAGMENT! Trimmed downstream.

### Hit: AF221913.1

AF221913.1 Bufo japonicus telomerase RNA gene, sequence

```
?

```
This is BLAST alignment as read from the input file
```

Score = 52.0 bits (48.2), Expect = 1.38E-01
 Identities = 29/31 (94%), Gaps = 0/31 (0%)
 Strand = Plus/Plus
Query 358 TGCTGTCGCGAAGAGTTCGGCTCTGTCAGCC 388
          |||||| |||||||||||| |||||||||||    
Sbjct 357 TGCTGTTGCGAAGAGTTCGTCTCTGTCAGCC 387
```

Report:

|  |  |
| --- | --- |
| sequence start ?  ``` Start position of the estimated full-length sequence in genome. Start index < end index. ``` : | 29 |
| sequence end ?  ``` End position of the estimated full-length sequence in genome. Start index < end index. ``` : | 520 |
| bit score (CM) ?  ``` The score for aligning estimated full-length sequence to CM model   (computed by RSEARCH -> default,   infered from Rfam or provided by user) ``` : | -18.85 |
| Homology estimate ?  ``` Quick homology estimate:   Not homologous: bit score < 0   Homologous: bit score > 20 and bit score > 0.5 * query length   Uncertain otherwise ``` : | Not homologous |

Estimated full-length sequence:


?

```
Click checkbox to select multiple seuqences.
Fasta header format:
  UID|accession.versionSTRAND start-end
```

>uid:56|AF221913.1fw 29-520
GUAGACCGAUAACCAAUCAAAUGGUAAUACAUACAUUACGUAAUUUUAUGUAUAAAUACG
UAUGUUUUUUUACCGGUAGUUUAAUUAGAGGGAUUGGAAGGUUCCGCUUAUGCUAACCCU
AAUAUUGGGGGUCUGUUGAAAACCUCUUUAAGAUAUGCGUGUUGUUUUAUUGGCUGACUU
UCAGCGCGCAUUGAGAGGAGUUGCUGCCCAGGACUAAAAAAUGUCAGCUGGGAGUCCUUC
CUCUCCCUUAUUUCUGCCUCACAACCUGGACUCUUUAUUUAGCGGUGCCCCAUUUGUCGA
GGCCGCAGUCAGUCUUGUUCUUAUACGCUGCUGUUGCGAAGAGUUCGUCUCUGUCAGCCU
CCGGGGCAACGCCUUGAAUUUGGAGAGCCUGGGAAUGUAACAAGGGGUAGGGAAAAUAAC
GAGAGCUGAGUUGGCUUCUCCUGUGCUGUUCCUGAGCUGUGGAACUUGCAAUCGCAGUCG
GCUCUGACACUU

C-A-r-Rc


?

```
Visualisation of predicted secondary structure.
To save the image:
  Right click on the image -> Save Image as.
```


centroid


?

```
Visualisation of predicted secondary structure.
To save the image:
  Right click on the image -> Save Image as.
```


fq-sub


?

```
Visualisation of predicted secondary structure.
To save the image:
  Right click on the image -> Save Image as.
```

Load Sequence viewer

uid:56|AF221913.1: Sequence cannot be extended sufficiently. Missing nt downstream in the genome.  
uid:56|AF221913.1: Sequence cannot be extended sufficiently by unaligned portion of query. THIS IS PROBABLY FRAGMENT! Trimmed upstream.  
uid:56|AF221913.1: Sequence cannot be extended sufficiently. Missing -30 nt upstream in the genome.  
TurboFold: Number of sequences is less then required. n=2 (4)  
uid:56|AF221913.1: Sequence cannot be extended sufficiently by unalined portion of query. THIS IS PROBABLY FRAGMENT! Trimmed downstream.

### Hit: LR606190.1

LR606190.1 Aquila chrysaetos chrysaetos genome assembly, chromosome: 10

```
?

```
This is BLAST alignment as read from the input file
```

Score = 51.0 bits (47.3), Expect = 4.81E-01
 Identities = 41/50 (82%), Gaps = 1/50 (2%)
 Strand = Plus/Minus
Query     354 CCAATGCTGTCGCGAAGAGTTCGGCTCTGTCAGCC-CGGCTGGGTCCGGG 402    
              ||| ||| | ||||||||| ||| ||||||||||| |||| | | |||||        
Sbjct 5913575 CCACTGCCGCCGCGAAGAGCTCGTCTCTGTCAGCCTCGGCGGCGGCCGGG 5913526
```

Report:

|  |  |
| --- | --- |
| sequence start ?  ``` Start position of the estimated full-length sequence in genome. Start index < end index. ``` : | 5913372 |
| sequence end ?  ``` End position of the estimated full-length sequence in genome. Start index < end index. ``` : | 5913957 |
| bit score (CM) ?  ``` The score for aligning estimated full-length sequence to CM model   (computed by RSEARCH -> default,   infered from Rfam or provided by user) ``` : | -12.84 |
| Homology estimate ?  ``` Quick homology estimate:   Not homologous: bit score < 0   Homologous: bit score > 20 and bit score > 0.5 * query length   Uncertain otherwise ``` : | Not homologous |

Estimated full-length sequence:


?

```
Click checkbox to select multiple seuqences.
Fasta header format:
  UID|accession.versionSTRAND start-end
```

>uid:57|LR606190.1rc 5913372-5913957
UUAAAAAGCCCCGCGGGGGCGCCCCCGCGUCGCGUGGCGGGUGGGGAGGCCCUGGUGUGA
CUAACCCUAAUGGCUGCUGCCGCGCUCCCCCGCGCCCGUCCGCUGUUUUACUCGCUGACU
UUCAGCGGACGAGGGGAGUAGCCUGGGUAGGGGUAGGGGGUGGUGAGAGGGAGCGGGGGG
GGGAAGGGGGUCCGCCACCAAAAAAAAAAAAAAACGUCAGCGAGGGGUCUCCUCACCCCG
GCCCGCCCUGGGGUCCCUGUGCCCCGUCCCCCGCCCCGCAGCCGGGGGCCCGCCGCGGAG
GUUCCCGCCGCCGCUAUCCCACCACCCUCUUCCCGCAGAGGCCGCGGUCGGCCGGCUCCC
GCCACUGCCGCCGCGAAGAGCUCGUCUCUGUCAGCCUCGGCGGCGGCCGGGAGCGAAGGG
GCGACCCCCCCCCCCCUCCUCCUCCUCCUCCUCUCCCACCCACCCGCUCCCCCUCUACCC
CCGCGCCGGGGGGGCCCCAGCAGAGCAAAACGGGAGCGGUGCCCCCGGGUCAACCGCCGC
GCUUCCCUCAGCCGUGGGGCGCGCGGCCGGCGCCGCUCCGACACGC

C-A-r-Rc


?

```
Visualisation of predicted secondary structure.
To save the image:
  Right click on the image -> Save Image as.
```


centroid


?

```
Visualisation of predicted secondary structure.
To save the image:
  Right click on the image -> Save Image as.
```


fq-sub


?

```
Visualisation of predicted secondary structure.
To save the image:
  Right click on the image -> Save Image as.
```

Load Sequence viewer

TurboFold: Number of sequences is less then required. n=2 (4)

### Hit: LR594560.1

LR594560.1 Streptopelia turtur genome assembly, chromosome: 9

```
?

```
This is BLAST alignment as read from the input file
```

Score = 51.0 bits (47.3), Expect = 4.81E-01
 Identities = 41/50 (82%), Gaps = 1/50 (2%)
 Strand = Plus/Plus
Query      354 CCAATGCTGTCGCGAAGAGTTCGGCTCTGTCAGCC-CGGCTGGGTCCGGG 402     
               ||| ||| | ||||||||| ||| ||||||||||| |||| | | |||||         
Sbjct 22927086 CCACTGCCGCCGCGAAGAGCTCGTCTCTGTCAGCCTCGGCGGCGGCCGGG 22927135
```

Report:

|  |  |
| --- | --- |
| sequence start ?  ``` Start position of the estimated full-length sequence in genome. Start index < end index. ``` : | 22926745 |
| sequence end ?  ``` End position of the estimated full-length sequence in genome. Start index < end index. ``` : | 22927287 |
| bit score (CM) ?  ``` The score for aligning estimated full-length sequence to CM model   (computed by RSEARCH -> default,   infered from Rfam or provided by user) ``` : | 15.46 |
| Homology estimate ?  ``` Quick homology estimate:   Not homologous: bit score < 0   Homologous: bit score > 20 and bit score > 0.5 * query length   Uncertain otherwise ``` : | Uncertain |

Estimated full-length sequence:


?

```
Click checkbox to select multiple seuqences.
Fasta header format:
  UID|accession.versionSTRAND start-end
```

>uid:58|LR594560.1fw 22926745-22927287
ACCAAUGGAGGCGCGGCGGGCGUGGCCGCGGAGGGUUUAAGAUGCCCCGCGGGGGCGCCC
CCACGUCGCGUGGCGGGUGGGGAGGCCCUGGUGUGACUAACCCUAAUGGCCGCCGCCGCG
CUCCCCGCGCCCGUCCGCUGUUUUACUCGCUGACUUUCAGCGGACGCUGGGAGUAGCUCG
GGAGGGGGGUGGAGGGGAAGGGGUUCCGCCAUCAAAAAACGUCAGCGAGGGGUCUCCUCA
CCCCGGCCCGCCCUGGGGUCCCAGUCCCCCCCAUAGCCGGGGGCCCGCCGCAGAGGCUCC
CGCCGCCGCACCGCCCGGAGGCCGCGGUCGGCCGGCUCCCGCCACUGCCGCCGCGAAGAG
CUCGUCUCUGUCAGCCUCGGCGGCGGCCGGGGAGCGGAGGGGCGCGCCCCCGCGAACCGG
GGGGCCCCAGCAGAGCAAAACGGGAGCGGCGCCCCCGGCACCGACCGCCGCCGCUUCCCU
CAGCCGUGGGGCGCGCGGCCGGCGCCGCUCCGACACGCGCGCGGCGGCCCCGCGCGGGUU
CGC

C-A-r-Rc


?

```
Visualisation of predicted secondary structure.
To save the image:
  Right click on the image -> Save Image as.
```


centroid


?

```
Visualisation of predicted secondary structure.
To save the image:
  Right click on the image -> Save Image as.
```


fq-sub


?

```
Visualisation of predicted secondary structure.
To save the image:
  Right click on the image -> Save Image as.
```

Load Sequence viewer

TurboFold: Number of sequences is less then required. n=2 (4)

### Hit: XM\_023733290.1

XM\_023733290.1 PREDICTED: Trichechus manatus latirostris sphingosine-1-phosphate receptor 4 (LOC101345635), mRNA

```
?

```
This is BLAST alignment as read from the input file
```

Score = 51.0 bits (47.3), Expect = 4.81E-01
 Identities = 30/33 (91%), Gaps = 0/33 (0%)
 Strand = Plus/Plus
Query  303 GGCCCAGCCACTCCGGGCCGCGATCAGCCCGGG 335 
           ||||||||||| ||||||||| ||| |||||||     
Sbjct 1243 GGCCCAGCCACACCGGGCCGCAATCTGCCCGGG 1275
```

Report:

|  |  |
| --- | --- |
| sequence start ?  ``` Start position of the estimated full-length sequence in genome. Start index < end index. ``` : | 937 |
| sequence end ?  ``` End position of the estimated full-length sequence in genome. Start index < end index. ``` : | 1467 |
| bit score (CM) ?  ``` The score for aligning estimated full-length sequence to CM model   (computed by RSEARCH -> default,   infered from Rfam or provided by user) ``` : | -54.61 |
| Homology estimate ?  ``` Quick homology estimate:   Not homologous: bit score < 0   Homologous: bit score > 20 and bit score > 0.5 * query length   Uncertain otherwise ``` : | Not homologous |

Estimated full-length sequence:


?

```
Click checkbox to select multiple seuqences.
Fasta header format:
  UID|accession.versionSTRAND start-end
```

>uid:59|XM\_023733290.1fw 937-1467
CCUGGAGCUGCCCUGGUUCAAAAGCACAUCGGUCAUCCUCUUCCUCAACAAGACCGACAU
CCUUGAGGAGAAGAUCUCCACCUCCCACCUGGCCACCUACUUCCCCAGUUUCCAGGAACA
CCUGUGCCUUUCAUGGGACUCCUCACGGGACAUCUGGCUGGCAUAGUUGGCCCCGCCGUC
UCCUCCCCAUGGCCAGAGGGCACCCCUGUCUCCGGCCCCACCACCACCCUGGGGGUACCU
UCAGCAUAACAGGAAAUUUCAAACAACAGGAAACAGCCCGGGCAAGGCAGCAGCUCCACC
CUGCCCGGCCCAGCCACACCGGGCCGCAAUCUGCCCGGGGCCCGCUAUGAACGCCACAGG
GCCCCCGGUGGCGGCCGCCGAGUCCUGCCAGCAGCUGGCAGACGAGGGGCACAGCCAGCU
CAUCCUGCUGCACUAUAACCACUCGGGGCGCCUGGCGUGGCGCGGGGGGCCUGAGGACGG
CGGCCUGGGGGCCCUGCGGGGCCUCUUCGUGGCCGUGAGUUGCCUGGUGGU

C-A-r-Rc


?

```
Visualisation of predicted secondary structure.
To save the image:
  Right click on the image -> Save Image as.
```


centroid


?

```
Visualisation of predicted secondary structure.
To save the image:
  Right click on the image -> Save Image as.
```


fq-sub


?

```
Visualisation of predicted secondary structure.
To save the image:
  Right click on the image -> Save Image as.
```

Load Sequence viewer

TurboFold: Number of sequences is less then required. n=2 (4)

### Hit: AF221924.1

AF221924.1 Anodorhynchus hyacinthinus telomerase RNA gene, sequence

```
?

```
This is BLAST alignment as read from the input file
```

Score = 51.0 bits (47.3), Expect = 4.81E-01
 Identities = 35/40 (88%), Gaps = 1/40 (2%)
 Strand = Plus/Plus
Query 354 CCAATGCTGTCGCGAAGAGTTCGGCTCTGTCAGCC-CGGC 392
          ||| ||| | ||||||||||||| ||||||||||| ||||    
Sbjct 369 CCACTGCCGCCGCGAAGAGTTCGTCTCTGTCAGCCTCGGC 408
```

Report:

|  |  |
| --- | --- |
| sequence start ?  ``` Start position of the estimated full-length sequence in genome. Start index < end index. ``` : | 81 |
| sequence end ?  ``` End position of the estimated full-length sequence in genome. Start index < end index. ``` : | 538 |
| bit score (CM) ?  ``` The score for aligning estimated full-length sequence to CM model   (computed by RSEARCH -> default,   infered from Rfam or provided by user) ``` : | 38.77 |
| Homology estimate ?  ``` Quick homology estimate:   Not homologous: bit score < 0   Homologous: bit score > 20 and bit score > 0.5 * query length   Uncertain otherwise ``` : | Uncertain |

Estimated full-length sequence:


?

```
Click checkbox to select multiple seuqences.
Fasta header format:
  UID|accession.versionSTRAND start-end
```

>uid:60|AF221924.1fw 81-538
AGGCGCCGCGAGGGGGCCGCUUGUCGCGUGGCGGAUGGGGAGGCUCCAGUCUCACUAACC
CUAAUGGCUGCCGCCGUGCUCCCCGCACCCGUCCGCUGUUUUAUUCGCUGACUUUCAGCG
GACGGGGGGAGCCGCCUGGGGGGGAAGGGGUUUGCAAUCAAAAAACGUCAGCGACGGGUC
UCCCCAGCCCAGCCCGCCCUGGGGUCUCCGUCCCCCCACGCAGCCGGGGGCCUGCCGCGG
AGGCUCCCUCCGCCGCACUUCACGGAGGCCGCGGUCGGCCGGUGUCCGCCACUGCCGCCG
CGAAGAGUUCGUCUCUGUCAGCCUCGGCGGCGGUGGGGAGCGAGAGGGCUCGUCCCCGCG
CCGGGGACCCCAGCAGAGCAAAACGGAGCGGCGUCCUCGGCACAGCCGCCGCGCUUCCCU
CAACCGUGGGAUGCGCGGACGGCGCCGCUUCGACACCC

C-A-r-Rc


?

```
Visualisation of predicted secondary structure.
To save the image:
  Right click on the image -> Save Image as.
```


centroid


?

```
Visualisation of predicted secondary structure.
To save the image:
  Right click on the image -> Save Image as.
```


fq-sub


?

```
Visualisation of predicted secondary structure.
To save the image:
  Right click on the image -> Save Image as.
```

Load Sequence viewer

TurboFold: Number of sequences is less then required. n=2 (4)  
uid:60|AF221924.1: Sequence cannot be extended sufficiently. Missing nt downstream in the genome.  
uid:60|AF221924.1: Sequence cannot be extended sufficiently by unalined portion of query. THIS IS PROBABLY FRAGMENT! Trimmed downstream.  
uid:60|AF221924.1: Sequence cannot be extended sufficiently. Missing -14 nt upstream in the genome.

### Hit: AF221937.1

AF221937.1 Chinchilla brevicaudata telomerase RNA gene, sequence

```
?

```
This is BLAST alignment as read from the input file
```

Score = 50.0 bits (46.4), Expect = 4.81E-01
 Identities = 41/48 (85%), Gaps = 4/48 (8%)
 Strand = Plus/Plus
Query 364 CGCGAAGAGTTCGGCTCTGTCAGCCCGGCTGGGTCCGGGTGGGGGCCG 411
          ||||||||||||| |||||||||||  || |||| || | ||||||||    
Sbjct 392 CGCGAAGAGTTCGTCTCTGTCAGCC--GC-GGGT-CGCGCGGGGGCCG 435
```

Report:

|  |  |
| --- | --- |
| sequence start ?  ``` Start position of the estimated full-length sequence in genome. Start index < end index. ``` : | 30 |
| sequence end ?  ``` End position of the estimated full-length sequence in genome. Start index < end index. ``` : | 545 |
| bit score (CM) ?  ``` The score for aligning estimated full-length sequence to CM model   (computed by RSEARCH -> default,   infered from Rfam or provided by user) ``` : | 7.42 |
| Homology estimate ?  ``` Quick homology estimate:   Not homologous: bit score < 0   Homologous: bit score > 20 and bit score > 0.5 * query length   Uncertain otherwise ``` : | Uncertain |

Estimated full-length sequence:


?

```
Click checkbox to select multiple seuqences.
Fasta header format:
  UID|accession.versionSTRAND start-end
```

>uid:61|AF221937.1fw 30-545
CAAGGCUUCAGCCAAUCCGAGCGGGCGCCUCCUGCCCUCUUUAUAAGGAGCCUCUGCGCA
CACGUCCGCGGGUUGAGAAUGGUGGGCCGGGAGGGGAGGUGGGCAUGUUUUGUCUAACCC
UAACUAGGAGGAGGACGUAGGCGCCGUGCUUUUGUUCCCCGCGCGCUGUUUUUCUCGCUG
ACUUUCAGCGUGCGGAAAAGCCUUGGCCUGCCGUCGACCACUGUCUAAUUAAAAGCAAAC
AAAAAAUGUCAGCGUGGCCGGUCCGCCCCUCCCGGGUACCUGCGGCAGCUCGCCCGGCUG
GCCCCCGAGCCCCGCCCAGGGCCACGGCUGGCGCGGGGCUUCUCCGGGAGCGCCAUGGCC
GCCGCGAAGAGUUCGUCUCUGUCAGCCGCGGGUCGCGCGGGGGCCGCGGGGGAGUCCUAG
GCCGAGUGGCCGCAGGAAGAGAAACGGAGCCUGUCCCUGUGCACGGGGCGCUUCUCUGAG
CUGUGGGAAGUGCCCCGAGACUCGGCUCCUACAAGC

C-A-r-Rc


?

```
Visualisation of predicted secondary structure.
To save the image:
  Right click on the image -> Save Image as.
```


centroid


?

```
Visualisation of predicted secondary structure.
To save the image:
  Right click on the image -> Save Image as.
```


fq-sub


?

```
Visualisation of predicted secondary structure.
To save the image:
  Right click on the image -> Save Image as.
```

Load Sequence viewer

uid:61|AF221937.1: Sequence cannot be extended sufficiently. Missing nt downstream in the genome.  
TurboFold: Number of sequences is less then required. n=2 (4)  
uid:61|AF221937.1: Sequence cannot be extended sufficiently. Missing -1 nt upstream in the genome.

### Hit: KX208097.1

KX208097.1 Scinax ruber voucher TNHC-GDC 5850 piccolo presynaptic cytomatrix protein (PCLO) gene, partial cds

```
?

```
This is BLAST alignment as read from the input file
```

Score = 49.0 bits (45.5), Expect = 1.68E+00
 Identities = 31/34 (91%), Gaps = 1/34 (3%)
 Strand = Plus/Plus
Query 151 GGAGCAGCAACGGCAAGCAAAAAAAAAGTTCCAG 184
          ||||||||||| |||| |||| ||||||||||||    
Sbjct 687 GGAGCAGCAACAGCAA-CAAAGAAAAAGTTCCAG 719
```

Report:

|  |  |
| --- | --- |
| sequence start ?  ``` Start position of the estimated full-length sequence in genome. Start index < end index. ``` : | 536 |
| sequence end ?  ``` End position of the estimated full-length sequence in genome. Start index < end index. ``` : | 927 |
| bit score (CM) ?  ``` The score for aligning estimated full-length sequence to CM model   (computed by RSEARCH -> default,   infered from Rfam or provided by user) ``` : | -41.31 |
| Homology estimate ?  ``` Quick homology estimate:   Not homologous: bit score < 0   Homologous: bit score > 20 and bit score > 0.5 * query length   Uncertain otherwise ``` : | Not homologous |

Estimated full-length sequence:


?

```
Click checkbox to select multiple seuqences.
Fasta header format:
  UID|accession.versionSTRAND start-end
```

>uid:62|KX208097.1fw 536-927
ACAAAAAGAUGUCAGGGGAAAGCAAACAGCAGAGGAAAGCAAGGCAUAGAUCUCAUGGGC
CUGUUUUACCCACUAUUGAAGACUCCUCUGAAGAAGAAGAAUUAAGAGAAGAGGAAGAAC
UUUUGAAAGAGCAGGAAAAGCAAAGAGAAUUGGAGCAGCAACAGCAACAAAGAAAAAGUU
CCAGUAAAAAAUCCAAAAAAGACAAAGAUGAACUAAGAGCACAGAGAAGAAGAGAAAGAC
CUAAGACCCCACCGAGCAACCUUUCUCCCAUUGAAGAUGCCUCUCCGACAGAAGAAUUGC
GUCAAGCAGCUGAAAUGGAAGAACUUCAUAGAUCCUCAUGUUCAGAAUAUUCACCUAGUA
UUGAGUCAGAACCAGAAGGAUUUGAAAUUAGU

C-A-r-Rc


?

```
Visualisation of predicted secondary structure.
To save the image:
  Right click on the image -> Save Image as.
```


centroid


?

```
Visualisation of predicted secondary structure.
To save the image:
  Right click on the image -> Save Image as.
```


fq-sub


?

```
Visualisation of predicted secondary structure.
To save the image:
  Right click on the image -> Save Image as.
```

Load Sequence viewer

TurboFold: Number of sequences is less then required. n=2 (4)  
uid:62|KX208097.1: Sequence cannot be extended sufficiently. Missing nt downstream in the genome.  
uid:62|KX208097.1: Sequence cannot be extended sufficiently by unalined portion of query. THIS IS PROBABLY FRAGMENT! Trimmed downstream.

### Hit: EU139123.1

EU139123.1 Ovis aries telomerase RNA gene, partial sequence

```
?

```
This is BLAST alignment as read from the input file
```

Score = 49.0 bits (45.5), Expect = 1.68E+00
 Identities = 67/91 (74%), Gaps = 7/91 (8%)
 Strand = Plus/Plus
Query 318 GGCCGCGATCAGCCCGG-GCTTTCCCTACTTTGGGGCCCAATGCTGTCGCGAAGAGTTCGGCTCTGTCAG 386
          ||||||  || |||||| ||||   || |   || | ||| ||| | || |||||||| |||||||||||    
Sbjct 277 GGCCGCAGTCGGCCCGGTGCTT---CTCCGGAGGTGTCCATTGCCGCCGTGAAGAGTTGGGCTCTGTCAG 343

Query 387 CC-CGGCTGGGTCCGGGTGGG 406
          || ||| | | ||  ||||||    
Sbjct 344 CCGCGGGTCGCTC--GGTGGG 362
```

Report:

|  |  |
| --- | --- |
| sequence start ?  ``` Start position of the estimated full-length sequence in genome. Start index < end index. ``` : | 1 |
| sequence end ?  ``` End position of the estimated full-length sequence in genome. Start index < end index. ``` : | 474 |
| bit score (CM) ?  ``` The score for aligning estimated full-length sequence to CM model   (computed by RSEARCH -> default,   infered from Rfam or provided by user) ``` : | -16.88 |
| Homology estimate ?  ``` Quick homology estimate:   Not homologous: bit score < 0   Homologous: bit score > 20 and bit score > 0.5 * query length   Uncertain otherwise ``` : | Not homologous |

Estimated full-length sequence:


?

```
Click checkbox to select multiple seuqences.
Fasta header format:
  UID|accession.versionSTRAND start-end
```

>uid:63|EU139123.1fw 1-474
UCAGCCUUCAAAAAUGAGGAGAUCCGGGUUGCGGAGGGUGGGCCCCGGGUUGGUGGCAGC
CAUUUCUCAUCUAACCCUAAUUGAGACAGGCGUAGGCGCUGUGCUUUUGGUUACCGCGCG
CUGUUUUUCUCGCUGACUUUCAGCGGGCGGAAAAGCCUCGGCCUACCGCCAUCCACCAUC
CAGUCUGCAACAAACAAAAAAUGUCAGCCGCUGGCUCGCUCACCUCUCCCGGGAACCUGC
GGUGGUCCGCCCGCCCAGCCCCAGUGCCCCGCCUGAGGCCGCAGUCGGCCCGGUGCUUCU
CCGGAGGUGUCCAUUGCCGCCGUGAAGAGUUGGGCUCUGUCAGCCGCGGGUCGCUCGGUG
GGCCGAGGCAUGGCUGUAACCGCAGGGAAAGGAACGGAGUGGGGUCCCCGCGCGCGGUGC
GCUUCCCUGAGCUGUGGGACUUGUACCCGGGACUCGGCUCAGACAUCUGAAAAA

C-A-r-Rc


?

```
Visualisation of predicted secondary structure.
To save the image:
  Right click on the image -> Save Image as.
```


centroid


?

```
Visualisation of predicted secondary structure.
To save the image:
  Right click on the image -> Save Image as.
```


fq-sub


?

```
Visualisation of predicted secondary structure.
To save the image:
  Right click on the image -> Save Image as.
```

Load Sequence viewer

TurboFold: Number of sequences is less then required. n=2 (4)  
uid:63|EU139123.1: Sequence cannot be extended sufficiently by unaligned portion of query. THIS IS PROBABLY FRAGMENT! Trimmed upstream.  
uid:63|EU139123.1: Sequence cannot be extended sufficiently. Missing -70 nt upstream in the genome.  
uid:63|EU139123.1: Sequence cannot be extended sufficiently. Missing nt downstream in the genome.

### Hit: AC092069.2

AC092069.2 Homo sapiens chromosome 19 clone CTC-425F1, complete sequence

```
?

```
This is BLAST alignment as read from the input file
```

Score = 49.0 bits (45.5), Expect = 1.68E+00
 Identities = 29/32 (91%), Gaps = 0/32 (0%)
 Strand = Plus/Minus
Query  375 CGGCTCTGTCAGCCCGGCTGGGTCCGGGTGGG 406 
           || ||||||| |||||| ||||||||||||||     
Sbjct 6155 CGTCTCTGTCGGCCCGGGTGGGTCCGGGTGGG 6124
```

Report:

|  |  |
| --- | --- |
| sequence start ?  ``` Start position of the estimated full-length sequence in genome. Start index < end index. ``` : | 5983 |
| sequence end ?  ``` End position of the estimated full-length sequence in genome. Start index < end index. ``` : | 6525 |
| bit score (CM) ?  ``` The score for aligning estimated full-length sequence to CM model   (computed by RSEARCH -> default,   infered from Rfam or provided by user) ``` : | -50.5 |
| Homology estimate ?  ``` Quick homology estimate:   Not homologous: bit score < 0   Homologous: bit score > 20 and bit score > 0.5 * query length   Uncertain otherwise ``` : | Not homologous |

Estimated full-length sequence:


?

```
Click checkbox to select multiple seuqences.
Fasta header format:
  UID|accession.versionSTRAND start-end
```

>uid:64|AC092069.2rc 5983-6525
UCUCCUGACCUCGUGAUCGGCCCGCCUCGGCCUCCUAAAGUGCUGGGAUUACACUGCGCU
CGGCCUCUCAUCUGUUUCUUUCCCCAUCCUUCUGUCUCUCAAUCUCUCCAGCUUUCUCCG
GCCCUCCCAGUGUCUUCAUCUCUCCCCAUUUCUGUCUCUCCCCACCAGGUCUCCCUAUCU
CUUCCCAUCUCUCUCCGCAUCUCUGGGUCUCCCAUCUCUGGUCUCUCCCUGUCUCUCUUU
UUUCUCUCCCCAUUUCUCUGUCUCUCCCCAUCUCCGUCUCUCUCAUCUCUGGUCUCUCUC
AUCUCUGGUCUCUCCCUGUCUCUCCUGUCUUUUUCCCUCCCCAUUUCUCUGUCUCUCCCC
AUCCCGGUCUCCCCGUCUCUGUCGGCCCGGGUGGGUCCGGGUGGGUCCGGGACGGCCCGG
CCCGGGCGCCGCGGCGAACAAUGCCCCAUUCACGCGGGCCGCCGGCGCCAUGGCAACGCG
CCCGCCCCGCCCUCCCUGCGCGCGGGCCGCGCGUGCCAAGAGGAUGCCAUGGUUACGUCA
GGC

C-A-r-Rc


?

```
Visualisation of predicted secondary structure.
To save the image:
  Right click on the image -> Save Image as.
```


centroid


?

```
Visualisation of predicted secondary structure.
To save the image:
  Right click on the image -> Save Image as.
```


fq-sub


?

```
Visualisation of predicted secondary structure.
To save the image:
  Right click on the image -> Save Image as.
```

Load Sequence viewer

TurboFold: Number of sequences is less then required. n=2 (4)

### Hit: AC138474.2

AC138474.2 Homo sapiens chromosome 19 clone CTC-239J10, complete sequence

```
?

```
This is BLAST alignment as read from the input file
```

Score = 49.0 bits (45.5), Expect = 1.68E+00
 Identities = 29/32 (91%), Gaps = 0/32 (0%)
 Strand = Plus/Minus
Query   375 CGGCTCTGTCAGCCCGGCTGGGTCCGGGTGGG 406  
            || ||||||| |||||| ||||||||||||||      
Sbjct 63772 CGTCTCTGTCGGCCCGGGTGGGTCCGGGTGGG 63741
```

Report:

|  |  |
| --- | --- |
| sequence start ?  ``` Start position of the estimated full-length sequence in genome. Start index < end index. ``` : | 63569 |
| sequence end ?  ``` End position of the estimated full-length sequence in genome. Start index < end index. ``` : | 63799 |
| bit score (CM) ?  ``` The score for aligning estimated full-length sequence to CM model   (computed by RSEARCH -> default,   infered from Rfam or provided by user) ``` : | -21.44 |
| Homology estimate ?  ``` Quick homology estimate:   Not homologous: bit score < 0   Homologous: bit score > 20 and bit score > 0.5 * query length   Uncertain otherwise ``` : | Not homologous |

Estimated full-length sequence:


?

```
Click checkbox to select multiple seuqences.
Fasta header format:
  UID|accession.versionSTRAND start-end
```

>uid:65|AC138474.2rc 63569-63799
CUCUGUCUCUCCCCAUCCCGGUCUCCCCGUCUCUGUCGGCCCGGGUGGGUCCGGGUGGGU
CCGGGACGGCCCGGCCCGGGCGCCGCGGCGAACAAUGCCCCAUUCACGCGGGCCGCCGGC
GCCAUGGCAACGCGCCCGCCCCGCCCUCCCUGCGCGCGGGCCGCGCGUGCCAAGAGGAUG
CCAUGGUUACGUCAGGCGCGUGCCCGUCGCGCGCGCCGCCGUCCCGCGCGC

C-A-r-Rc


?

```
Visualisation of predicted secondary structure.
To save the image:
  Right click on the image -> Save Image as.
```


centroid


?

```
Visualisation of predicted secondary structure.
To save the image:
  Right click on the image -> Save Image as.
```


fq-sub


?

```
Visualisation of predicted secondary structure.
To save the image:
  Right click on the image -> Save Image as.
```

Load Sequence viewer

uid:65|AC138474.2: Sequence cannot be extended sufficiently. Missing nt downstream in the genome.  
TurboFold: Number of sequences is less then required. n=2 (4)  
uid:65|AC138474.2: Sequence cannot be extended sufficiently by unalined portion of query. THIS IS PROBABLY FRAGMENT! Trimmed downstream.

### Hit: NG\_005076.1

NG\_005076.1 Bos taurus telomerase RNA pseudogene (LOC548614) on chromosome 18 >AF221941.1 Bos taurus telomerase RNA pseudogene

```
?

```
This is BLAST alignment as read from the input file
```

Score = 49.0 bits (45.5), Expect = 1.68E+00
 Identities = 67/91 (74%), Gaps = 7/91 (8%)
 Strand = Plus/Plus
Query 318 GGCCGCGATCAGCCCGG-GCTTTCCCTACTTTGGGGCCCAATGCTGTCGCGAAGAGTTCGGCTCTGTCAG 386
          ||||||  || |||||| ||||   || |   || | ||| ||| | || |||||||| |||||||||||    
Sbjct 505 GGCCGCAGTCGGCCCGGTGCTT---CTCCGGAGGTGTCCATTGCCGCCGTGAAGAGTTGGGCTCTGTCAG 571

Query 387 CC-CGGCTGGGTCCGGGTGGG 406
          || ||| | | ||  ||||||    
Sbjct 572 CCGCGGGTCGCTC--GGTGGG 590
```

Report:

|  |  |
| --- | --- |
| sequence start ?  ``` Start position of the estimated full-length sequence in genome. Start index < end index. ``` : | 196 |
| sequence end ?  ``` End position of the estimated full-length sequence in genome. Start index < end index. ``` : | 738 |
| bit score (CM) ?  ``` The score for aligning estimated full-length sequence to CM model   (computed by RSEARCH -> default,   infered from Rfam or provided by user) ``` : | -23.19 |
| Homology estimate ?  ``` Quick homology estimate:   Not homologous: bit score < 0   Homologous: bit score > 20 and bit score > 0.5 * query length   Uncertain otherwise ``` : | Not homologous |

Estimated full-length sequence:


?

```
Click checkbox to select multiple seuqences.
Fasta header format:
  UID|accession.versionSTRAND start-end
```

>uid:66|NG\_005076.1fw 196-738
UCCAAUUUCUAAAUGAACAUGAUGGAAUACUAUUCAGCCUUCAAAAAUGAGGAGAUCCGG
GUUGCGGAGGGUGGGCCCCGGGUUGGUGGCAGCCAUUUCUCAUCUAACCCUAAUUGAGAC
AGGCGUAGGCGCUGUGCUUUUGGUUACCGCGCGCUGUUUUUCUCGCUGACUUUCAGCGGG
CGGAAAAGCCUCGGCCUACCGCCAUCCACCAUCCAGUCUGCAACAAACAAAAAAUGUCAG
CCGCUGGCUCGCUCACCUCUCCCGGGAACCUGCGGUGGUCCGCCCGCCCAGCCCCAGUGC
CCCGCCUGAGGCCGCAGUCGGCCCGGUGCUUCUCCGGAGGUGUCCAUUGCCGCCGUGAAG
AGUUGGGCUCUGUCAGCCGCGGGUCGCUCGGUGGGCCGAGGCAUGGCUGUAACCGCAGGG
AAAGGAACGGAGUGGGGUCCCCGCGCGCGGUGCGCUUCCCUGAGCUGUGGGACUUGCACC
CGGGACUCGGCUCAGACAUCUGAAAAAAAAAAAAAUGAGGAGAUCCUACCAUAUGAAACA
AUA

C-A-r-Rc


?

```
Visualisation of predicted secondary structure.
To save the image:
  Right click on the image -> Save Image as.
```


centroid


?

```
Visualisation of predicted secondary structure.
To save the image:
  Right click on the image -> Save Image as.
```


fq-sub


?

```
Visualisation of predicted secondary structure.
To save the image:
  Right click on the image -> Save Image as.
```

Load Sequence viewer

TurboFold: Number of sequences is less then required. n=2 (4)

### Hit: AF221932.1

AF221932.1 Elephas maximus telomerase RNA gene, sequence

```
?

```
This is BLAST alignment as read from the input file
```

Score = 49.0 bits (45.5), Expect = 1.68E+00
 Identities = 53/71 (75%), Gaps = 2/71 (3%)
 Strand = Plus/Plus
Query 318 GGCCGCGATCAGCCCGGGCTTTCCCTACTTTGGGGCCCAATGCTGTCGCGAAGAGTTCGGCTCTGTCAGC 387
          ||||||| || ||| |||  ||| |  |   ||  |||  ||| | ||||||||||| ||||||||||||    
Sbjct 352 GGCCGCGGTCGGCCTGGGGCTTCTC--CGGAGGTTCCCGCTGCCGCCGCGAAGAGTTGGGCTCTGTCAGC 419

Query 388 C 388
          |    
Sbjct 420 C 420
```

Report:

|  |  |
| --- | --- |
| sequence start ?  ``` Start position of the estimated full-length sequence in genome. Start index < end index. ``` : | 37 |
| sequence end ?  ``` End position of the estimated full-length sequence in genome. Start index < end index. ``` : | 549 |
| bit score (CM) ?  ``` The score for aligning estimated full-length sequence to CM model   (computed by RSEARCH -> default,   infered from Rfam or provided by user) ``` : | 7.15 |
| Homology estimate ?  ``` Quick homology estimate:   Not homologous: bit score < 0   Homologous: bit score > 20 and bit score > 0.5 * query length   Uncertain otherwise ``` : | Uncertain |

Estimated full-length sequence:


?

```
Click checkbox to select multiple seuqences.
Fasta header format:
  UID|accession.versionSTRAND start-end
```

>uid:67|AF221932.1fw 37-549
UGGCCAAUCCGCGCGGUGGCGGUCUCUCCCUUUAUAAAGAGGUGCGGCGGCGCGGCUGGU
GCGGUGGGUUGAGGAGGGUACGCCCGGGAGGGCGGUGGUCUGUUCUGUUCUAACCCUAAC
UGAUAAGGGCGUAGGCGCCGUGCUUUUGUUCCCCGCGCGUUGUUUUUCUCGCUGACUUUC
AGCGGGCGGGAAAAGCCUCGGUCUACCGCCGUCUACCGAUAGCCUGGAGCAAACAAAAAA
AUGUCAGCCGCCGGCCGCUCGCCCCUCCCGGGAACCUGCAGUGGCUCGCCCGCCCAGCCC
CGCUCCCCGCCUGGAGGCCGCGGUCGGCCUGGGGCUUCUCCGGAGGUUCCCGCUGCCGCC
GCGAAGAGUUGGGCUCUGUCAGCCGCGGGUCCCGCGGGAACCAAGGGCGAGGCUGGGGCC
UCCUGAACGCAGGGAGAGAAACGGAGCGGUUCCCCGCGUGCGUGCGCUUCCCUGAGUUGU
GGGAUGUGCGCUCGGGGCUCAGCUCCGACAGGU

C-A-r-Rc


?

```
Visualisation of predicted secondary structure.
To save the image:
  Right click on the image -> Save Image as.
```


centroid


?

```
Visualisation of predicted secondary structure.
To save the image:
  Right click on the image -> Save Image as.
```


fq-sub


?

```
Visualisation of predicted secondary structure.
To save the image:
  Right click on the image -> Save Image as.
```

Load Sequence viewer

TurboFold: Number of sequences is less then required. n=2 (4)  
uid:67|AF221932.1: Sequence cannot be extended sufficiently by unalined portion of query. THIS IS PROBABLY FRAGMENT! Trimmed downstream.  
uid:67|AF221932.1: Sequence cannot be extended sufficiently. Missing nt downstream in the genome.

### Hit: AF221926.1

AF221926.1 Ceratophrys ornata telomerase RNA gene, sequence

```
?

```
This is BLAST alignment as read from the input file
```

Score = 49.0 bits (45.5), Expect = 1.68E+00
 Identities = 26/27 (96%), Gaps = 0/27 (0%)
 Strand = Plus/Plus
Query 362 GTCGCGAAGAGTTCGGCTCTGTCAGCC 388
          ||||||||||||||| |||||||||||    
Sbjct 353 GTCGCGAAGAGTTCGTCTCTGTCAGCC 379
```

Report:

|  |  |
| --- | --- |
| sequence start ?  ``` Start position of the estimated full-length sequence in genome. Start index < end index. ``` : | 11 |
| sequence end ?  ``` End position of the estimated full-length sequence in genome. Start index < end index. ``` : | 507 |
| bit score (CM) ?  ``` The score for aligning estimated full-length sequence to CM model   (computed by RSEARCH -> default,   infered from Rfam or provided by user) ``` : | -8.76 |
| Homology estimate ?  ``` Quick homology estimate:   Not homologous: bit score < 0   Homologous: bit score > 20 and bit score > 0.5 * query length   Uncertain otherwise ``` : | Not homologous |

Estimated full-length sequence:


?

```
Click checkbox to select multiple seuqences.
Fasta header format:
  UID|accession.versionSTRAND start-end
```

>uid:68|AF221926.1fw 11-507
GUAAGUUUCCCGCCACGCUCCGUCUGGACAGCGCAGCCGGCGAGCGGUGACGUCAUGUUA
CGUAUAAAAGUCAGACCCGGCCAAUGCGGCUUACAGUGGGAAUAGGAGGGAGUCUAUAUU
UCUAACCCUAAUAUACCCGGUUCAGGGCUCUUAUGUGGCGCUCGUUGUUUUGCCGGUUGC
CUUUCAGCGGGCGAAAGAGCUCAGAGAAGCGAGGACCAAAAAACGGCAGCCGCGGGCCCU
CCUGUUCCCACCAUCCCAGCUUUUCCACACUGCGCCUGGGUUCUCACUCAAGUGUUCGGC
AGCUUCCACUUACGAGGCCGCGGUCUACCGCUGUCACUGGUAGUCGCGAAGAGUUCGUCU
CUGUCAGCCUUGGGAGCCGCGGACGGAGUAUGAGGUCCAGUAAUGAGAGCAGGGAAGAGU
AAAGCGAGCCGCGCUACUUGUCCUAUCUGCCGCUCCUAAGCUGUGGGGCGUGUAGGGUAC
ACAAGGCUCCGACAUUC

C-A-r-Rc


?

```
Visualisation of predicted secondary structure.
To save the image:
  Right click on the image -> Save Image as.
```


centroid


?

```
Visualisation of predicted secondary structure.
To save the image:
  Right click on the image -> Save Image as.
```


fq-sub


?

```
Visualisation of predicted secondary structure.
To save the image:
  Right click on the image -> Save Image as.
```

Load Sequence viewer

TurboFold: Number of sequences is less then required. n=2 (4)  
uid:68|AF221926.1: Sequence cannot be extended sufficiently. Missing nt downstream in the genome.  
uid:68|AF221926.1: Sequence cannot be extended sufficiently by unaligned portion of query. THIS IS PROBABLY FRAGMENT! Trimmed upstream.  
uid:68|AF221926.1: Sequence cannot be extended sufficiently by unalined portion of query. THIS IS PROBABLY FRAGMENT! Trimmed downstream.  
uid:68|AF221926.1: Sequence cannot be extended sufficiently. Missing -38 nt upstream in the genome.

### Hit: AF054814.1

AF054814.1 Bos taurus telomerase RNA gene, complete sequence

```
?

```
This is BLAST alignment as read from the input file
```

Score = 49.0 bits (45.5), Expect = 1.68E+00
 Identities = 67/91 (74%), Gaps = 7/91 (8%)
 Strand = Plus/Plus
Query 318 GGCCGCGATCAGCCCGG-GCTTTCCCTACTTTGGGGCCCAATGCTGTCGCGAAGAGTTCGGCTCTGTCAG 386
          ||||||| || |||||| ||||   || |   || | ||| ||| | || ||||| || |||||||||||    
Sbjct 244 GGCCGCGGTCGGCCCGGTGCTT---CTCCGGAGGTGTCCATTGCCGCCGTGAAGAATTGGGCTCTGTCAG 310

Query 387 CC-CGGCTGGGTCCGGGTGGG 406
          || ||| | | ||  ||||||    
Sbjct 311 CCGCGGGTCGCTC--GGTGGG 329
```

Report:

|  |  |
| --- | --- |
| sequence start ?  ``` Start position of the estimated full-length sequence in genome. Start index < end index. ``` : | 1 |
| sequence end ?  ``` End position of the estimated full-length sequence in genome. Start index < end index. ``` : | 435 |
| bit score (CM) ?  ``` The score for aligning estimated full-length sequence to CM model   (computed by RSEARCH -> default,   infered from Rfam or provided by user) ``` : | 11.02 |
| Homology estimate ?  ``` Quick homology estimate:   Not homologous: bit score < 0   Homologous: bit score > 20 and bit score > 0.5 * query length   Uncertain otherwise ``` : | Uncertain |

Estimated full-length sequence:


?

```
Click checkbox to select multiple seuqences.
Fasta header format:
  UID|accession.versionSTRAND start-end
```

>uid:69|AF054814.1fw 1-435
GAGGGUGGGCCCCGGGUUGGUGGCAGCCAUUUCUCAUCUAACCCUAAUUGAGACAGGCGU
AGGCGCUGUGCUUUUGGUUACCGCGCGCUGUUUUUCUCGCUGACUUUCAGCGGGCGGAAA
AGCCUCGGCCUACCGCCAUCCACCAUCCAGUCUGCAACAAACAAAAAAUGUCAGCCGCUG
GCUCGCUCACCUCUCCCGGGAACCUGCGGUGGUCCGCCCGCCCAGCCCCAGUGCCCCGCC
UGAGGCCGCGGUCGGCCCGGUGCUUCUCCGGAGGUGUCCAUUGCCGCCGUGAAGAAUUGG
GCUCUGUCAGCCGCGGGUCGCUCGGUGGGCCGAGGCAUGGCUGUAACCGCAGGGAAAGGA
ACGGAGUGGGGUCCCCGCGCGCGGUGCGCUUCCCUGAGCUGUGGGACUUGCACCCGGGAC
UCGGCUCAGACAUCC

C-A-r-Rc


?

```
Visualisation of predicted secondary structure.
To save the image:
  Right click on the image -> Save Image as.
```


centroid


?

```
Visualisation of predicted secondary structure.
To save the image:
  Right click on the image -> Save Image as.
```


fq-sub


?

```
Visualisation of predicted secondary structure.
To save the image:
  Right click on the image -> Save Image as.
```

Load Sequence viewer

TurboFold: Number of sequences is less then required. n=2 (4)  
uid:69|AF054814.1: Sequence cannot be extended sufficiently. Missing -103 nt upstream in the genome.  
uid:69|AF054814.1: Sequence cannot be extended sufficiently. Missing nt downstream in the genome.  
uid:69|AF054814.1: Sequence cannot be extended sufficiently by unaligned portion of query. THIS IS PROBABLY FRAGMENT! Trimmed upstream.

### Hit: AC194569.3

AC194569.3 Pan troglodytes BAC clone CH251-94G22 from chromosome 4, complete sequence

```
?

```
This is BLAST alignment as read from the input file
```

Score = 48.0 bits (44.6), Expect = 1.68E+00
 Identities = 33/38 (87%), Gaps = 2/38 (5%)
 Strand = Plus/Plus
Query   387 CCCGGCTGGGTCCGGGTGG--GGGCCGGCACAGAGGGA 422  
            ||||||||||||||| |||  ||| ||| |||||||||      
Sbjct 34188 CCCGGCTGGGTCCGGCTGGGTGGGACGGGACAGAGGGA 34225
```

Report:

|  |  |
| --- | --- |
| sequence start ?  ``` Start position of the estimated full-length sequence in genome. Start index < end index. ``` : | 33796 |
| sequence end ?  ``` End position of the estimated full-length sequence in genome. Start index < end index. ``` : | 34329 |
| bit score (CM) ?  ``` The score for aligning estimated full-length sequence to CM model   (computed by RSEARCH -> default,   infered from Rfam or provided by user) ``` : | -38.2 |
| Homology estimate ?  ``` Quick homology estimate:   Not homologous: bit score < 0   Homologous: bit score > 20 and bit score > 0.5 * query length   Uncertain otherwise ``` : | Not homologous |

Estimated full-length sequence:


?

```
Click checkbox to select multiple seuqences.
Fasta header format:
  UID|accession.versionSTRAND start-end
```

>uid:70|AC194569.3fw 33796-34329
UCUCUGCACUGUCAGACCCCAGGCAACCACCUGGCCUGUCUGGCCCUCGGUGUCCUCAUC
UGUGACAGGGAGGAGCCACCGGUGACUGAGUGGAUGGGAGGUGAGUCCAAGUCGCCCCAG
AGCCCGCAGAGCUCAGGCCAGGCCCAGGCUCGGGGAGGGGCAGGGUGGUCAGGGUGGCGC
CGGCCUUCACAGAGGCGAAUUCCUUCUCUGUUCACAGCUUUUUCCUCCGUUUUUUUUCCU
CUUUCUUCCUACUCCAUUCCUUCCUGGAGCCCCUCCAAGACAAUCGGCACCCGGCUCCAC
UUGGCGGCCACAAAGCCUCUUUGAUGGCCCAGGUCCAACAGCGGGCGGCGGGGGUGGAGG
CUGGGGCGGCAGACGCUGGAGGCCGGCAGGGCCCCGGCUGGGUCCGGCUGGGUGGGACGG
GACAGAGGGAGUCUUCUGCUGGGGCUGGUGAGGCCUGAAUCAGCCCCGGGGGUGUUGGGG
UGGGGGUGUCUCUUGUACACGGGUGUCAGACCCAGACUUCCCUGCCAAAGGCUC

C-A-r-Rc


?

```
Visualisation of predicted secondary structure.
To save the image:
  Right click on the image -> Save Image as.
```


centroid


?

```
Visualisation of predicted secondary structure.
To save the image:
  Right click on the image -> Save Image as.
```


fq-sub


?

```
Visualisation of predicted secondary structure.
To save the image:
  Right click on the image -> Save Image as.
```

Load Sequence viewer

TurboFold: Number of sequences is less then required. n=2 (4)

### Hit: AC019103.8

AC019103.8 Homo sapiens BAC clone RP11-460I19 from 4, complete sequence

```
?

```
This is BLAST alignment as read from the input file
```

Score = 48.0 bits (44.6), Expect = 1.68E+00
 Identities = 33/38 (87%), Gaps = 2/38 (5%)
 Strand = Plus/Plus
Query    387 CCCGGCTGGGTCCGGGTGG--GGGCCGGCACAGAGGGA 422   
             ||||||||||||||| |||  ||| ||| |||||||||       
Sbjct 111208 CCCGGCTGGGTCCGGCTGGCTGGGACGGGACAGAGGGA 111245
```

Report:

|  |  |
| --- | --- |
| sequence start ?  ``` Start position of the estimated full-length sequence in genome. Start index < end index. ``` : | 110850 |
| sequence end ?  ``` End position of the estimated full-length sequence in genome. Start index < end index. ``` : | 111349 |
| bit score (CM) ?  ``` The score for aligning estimated full-length sequence to CM model   (computed by RSEARCH -> default,   infered from Rfam or provided by user) ``` : | -36.35 |
| Homology estimate ?  ``` Quick homology estimate:   Not homologous: bit score < 0   Homologous: bit score > 20 and bit score > 0.5 * query length   Uncertain otherwise ``` : | Not homologous |

Estimated full-length sequence:


?

```
Click checkbox to select multiple seuqences.
Fasta header format:
  UID|accession.versionSTRAND start-end
```

>uid:71|AC019103.8fw 110850-111349
CCCCUCUGGCCCUCGGUGUCCUCAUCUGUGACAGGGAGGAGCCACCGGCGACUGAGUAGA
UGGGAGGUGAGUCCAAGUCGCCCCGGAGCCCGCAGAGCUCAGGCCAGGCCCAGGCUCGGG
GAGGGGCGGGGCGGUCAGGGUGGCGCCGGCCUUCACAGAGGCGAAUUCCUUCUCUGUUCA
CAGCUUUUUCCUCCGUUUUUUUUCCUCUCUCUUCUUACUCCAUUCCUUCCUGGAGCCCCU
CCAAGACAAUCGGCACCCGGCUCCACUUGGCGGCCACAAAGCCUCUUUGACGGCCCAGGU
CCAACAGCGGGCGGCGGGGGUGGAGGCUGGGGCGGCAGACGCUGGAGGCCGGCAGGGCCC
CGGCUGGGUCCGGCUGGCUGGGACGGGACAGAGGGAGUCUUCUGCUGGGGCUGGUGAGGC
CUGAAUCAGCCCCGGGGGUGUUGGGGUGGGGGUGUCUCUUGUACACGGGUGUCAGACCCA
GACUUCCCUGCCAAAGGCUC

C-A-r-Rc


?

```
Visualisation of predicted secondary structure.
To save the image:
  Right click on the image -> Save Image as.
```


centroid


?

```
Visualisation of predicted secondary structure.
To save the image:
  Right click on the image -> Save Image as.
```


fq-sub


?

```
Visualisation of predicted secondary structure.
To save the image:
  Right click on the image -> Save Image as.
```

Load Sequence viewer

TurboFold: Number of sequences is less then required. n=2 (4)

### Hit: AF221942.1

AF221942.1 Sus scrofa telomerase RNA pseudogene

```
?

```
This is BLAST alignment as read from the input file
```

Score = 48.0 bits (44.6), Expect = 1.68E+00
 Identities = 33/39 (85%), Gaps = 0/39 (0%)
 Strand = Plus/Plus
Query 350 GGGCCCAATGCTGTCGCGAAGAGTTCGGCTCTGTCAGCC 388
          ||||||| |||   ||||||||||| |||| ||||||||    
Sbjct 325 GGGCCCACTGCCTCCGCGAAGAGTTGGGCTGTGTCAGCC 363
```

Report:

|  |  |
| --- | --- |
| sequence start ?  ``` Start position of the estimated full-length sequence in genome. Start index < end index. ``` : | 1 |
| sequence end ?  ``` End position of the estimated full-length sequence in genome. Start index < end index. ``` : | 482 |
| bit score (CM) ?  ``` The score for aligning estimated full-length sequence to CM model   (computed by RSEARCH -> default,   infered from Rfam or provided by user) ``` : | -47.49 |
| Homology estimate ?  ``` Quick homology estimate:   Not homologous: bit score < 0   Homologous: bit score > 20 and bit score > 0.5 * query length   Uncertain otherwise ``` : | Not homologous |

Estimated full-length sequence:


?

```
Click checkbox to select multiple seuqences.
Fasta header format:
  UID|accession.versionSTRAND start-end
```

>uid:72|AF221942.1fw 1-482
GAGAGCUGCCUUAUUCUGAAUUCCCAAAAUGUUCAGUAAAUUAUGAUCUAAACAGGAGCU
GUUUUCACCUAUUAAAAGAUGUUAUCAGGCGGGUUGCAGAGGGCAGGCCGGGAGAGGAGU
GGCCAUUUUUAAAAUCUGACCCUAACUGAAACAGGUGUAGGCACUGCACUUUUGCUUCCU
CGAGCGCUGUUUUUCUUGCUGACUUUCAGCGGAUGGAAGAGCCACCAUCCAGUCUGAAAC
AAACAAAAAAUGUCAGCCACUGGCUCGGUCACUGCUCCCGGGAACCUAAGAGUCUCGCCC
GCCCAGCCCCCCGCUUCUCCCAAAGGGCCCACUGCCUCCGCGAAGAGUUGGGCUGUGUCA
GCCGCGGGUUUCUCGGGGCCAAGGCGAGGCUCUGACCGCAGGGAAAGGAAGAGUUCCCUA
AGCUGUGGCAUGUGCAGCCAGGACUUGGCUCAGAUACUUGCAAAGAAAAAAAAAAAAACC
CC

C-A-r-Rc


?

```
Visualisation of predicted secondary structure.
To save the image:
  Right click on the image -> Save Image as.
```


centroid


?

```
Visualisation of predicted secondary structure.
To save the image:
  Right click on the image -> Save Image as.
```


fq-sub


?

```
Visualisation of predicted secondary structure.
To save the image:
  Right click on the image -> Save Image as.
```

Load Sequence viewer

uid:72|AF221942.1: Sequence cannot be extended sufficiently by unaligned portion of query. THIS IS PROBABLY FRAGMENT! Trimmed upstream.  
TurboFold: Number of sequences is less then required. n=2 (4)  
uid:72|AF221942.1: Sequence cannot be extended sufficiently. Missing nt downstream in the genome.  
uid:72|AF221942.1: Sequence cannot be extended sufficiently by unalined portion of query. THIS IS PROBABLY FRAGMENT! Trimmed downstream.  
uid:72|AF221942.1: Sequence cannot be extended sufficiently. Missing -54 nt upstream in the genome.

### Hit: LR606189.1

LR606189.1 Aquila chrysaetos chrysaetos genome assembly, chromosome: 9

```
?

```
This is BLAST alignment as read from the input file
```

Score = 47.0 bits (43.7), Expect = 5.85E+00
 Identities = 30/33 (91%), Gaps = 1/33 (3%)
 Strand = Plus/Plus
Query     400 GGGTG-GGGGCCGGCACAGAGGGACACACCGGG 431    
              ||||| ||||||||||| ||||||| |||||||        
Sbjct 3661015 GGGTGCGGGGCCGGCACTGAGGGACCCACCGGG 3661047
```

Report:

|  |  |
| --- | --- |
| sequence start ?  ``` Start position of the estimated full-length sequence in genome. Start index < end index. ``` : | 3660602 |
| sequence end ?  ``` End position of the estimated full-length sequence in genome. Start index < end index. ``` : | 3661164 |
| bit score (CM) ?  ``` The score for aligning estimated full-length sequence to CM model   (computed by RSEARCH -> default,   infered from Rfam or provided by user) ``` : | -46.65 |
| Homology estimate ?  ``` Quick homology estimate:   Not homologous: bit score < 0   Homologous: bit score > 20 and bit score > 0.5 * query length   Uncertain otherwise ``` : | Not homologous |

Estimated full-length sequence:


?

```
Click checkbox to select multiple seuqences.
Fasta header format:
  UID|accession.versionSTRAND start-end
```

>uid:73|LR606189.1fw 3660602-3661164
UCCUCCCUCUUUUCUUCCCGUUUCCAAAGGAUGAUCUCUGCCCGCUGAUACAGACCGAAA
GGUUUCCCCCCCCCCCCCCUUACAUAAACCAAACAAAUACAGGGAUAAAUAAAACCUGCU
ACGUGAGCGAAGCAGCCAGCCUGGCGCAACGCGUUGCUGUCCACUGCGGGAAGCGCGACA
GCAUCCCGGGGGGGGGGACACGGGACACACACGGGGGGGGGGGGACACACGCUCCGCGGG
CAGCCGCAGCCGCGUAUUGCCGGGGGGGGGGGGGAAUAUUCUUUAGGCUCCCGUCUGGGC
AAAGGUAACCCACCAAAUCCCCCGCAAGGGAUUUUAUCAGAGGGGGGGACUCCCCCAGAU
GCUGCUGAAGGCAGCUGGAGACCCCCCCACCUUCUCCUGGCCCCCCCAGCGGUGGGUGCG
GGGCCGGCACUGAGGGACCCACCGGGUAAGGGGAGUUGCCCUCCCCCAAAAAUGCGGGCC
ACGCUCCGGGGUCACUGCUAAAAGCAACACAACUCCGUAUUUUUUAAUAAAGAGCGUUCU
CUUGCGAGCUGGCUGCCUUCCAC

C-A-r-Rc


?

```
Visualisation of predicted secondary structure.
To save the image:
  Right click on the image -> Save Image as.
```


centroid


?

```
Visualisation of predicted secondary structure.
To save the image:
  Right click on the image -> Save Image as.
```


fq-sub


?

```
Visualisation of predicted secondary structure.
To save the image:
  Right click on the image -> Save Image as.
```

Load Sequence viewer

TurboFold: Number of sequences is less then required. n=2 (4)

### Hit: KX208106.1

KX208106.1 Agalychnis callidryas voucher TNHC-GDC 3050 piccolo presynaptic cytomatrix protein (PCLO) gene, partial cds

```
?

```
This is BLAST alignment as read from the input file
```

Score = 47.0 bits (43.7), Expect = 5.85E+00
 Identities = 38/45 (84%), Gaps = 2/45 (4%)
 Strand = Plus/Plus
Query 140 AAAAAAAGTTGGGAGCAGCAACGGCAAGCAAAAAAAAAGTTCCAG 184
          ||| | |||||| ||||||||| |||| |||| ||||||||| ||    
Sbjct 677 AAAGAGAGTTGG-AGCAGCAACAGCAA-CAAAGAAAAAGTTCTAG 719
```

Report:

|  |  |
| --- | --- |
| sequence start ?  ``` Start position of the estimated full-length sequence in genome. Start index < end index. ``` : | 521 |
| sequence end ?  ``` End position of the estimated full-length sequence in genome. Start index < end index. ``` : | 927 |
| bit score (CM) ?  ``` The score for aligning estimated full-length sequence to CM model   (computed by RSEARCH -> default,   infered from Rfam or provided by user) ``` : | -45.88 |
| Homology estimate ?  ``` Quick homology estimate:   Not homologous: bit score < 0   Homologous: bit score > 20 and bit score > 0.5 * query length   Uncertain otherwise ``` : | Not homologous |

Estimated full-length sequence:


?

```
Click checkbox to select multiple seuqences.
Fasta header format:
  UID|accession.versionSTRAND start-end
```

>uid:74|KX208106.1fw 521-927
AUAGUAGCCCAAGUCACAAAAAAAUGUCAGGAGAAAGCAAACAGCAAAGAAAAGCAAGAC
AUCGAUCUCAUGGGCCAGUUCUCCCCACUAUUGAAGACUCCUCUGAAGAGGAAGAAUUAA
GAGAAGAGGAAGAAUUGUUAAAAGAGCAGGAAAAGCAAAGAGAGUUGGAGCAGCAACAGC
AACAAAGAAAAAGUUCUAGUAAGAAAUCUAAAAAAGACAAAGAUGAACUACGAGCACAGA
GAAGGAGAGAGCGACCAAAAACACCUCCGAGUAAUCUAUCUCCUAUCGAAGAUGCUUCUC
CAACAGAAGAAUUGCGUCAAGCAGCUGAAAUGGAAGAACUUCACAGAUCUUCAUGUUCAG
AGUAUUCACCGAGUAUUGAGUCCGAACCAGAAGGAUUUGAAAUUAGU

C-A-r-Rc


?

```
Visualisation of predicted secondary structure.
To save the image:
  Right click on the image -> Save Image as.
```


centroid


?

```
Visualisation of predicted secondary structure.
To save the image:
  Right click on the image -> Save Image as.
```


fq-sub


?

```
Visualisation of predicted secondary structure.
To save the image:
  Right click on the image -> Save Image as.
```

Load Sequence viewer

TurboFold: Number of sequences is less then required. n=2 (4)  
uid:74|KX208106.1: Sequence cannot be extended sufficiently. Missing nt downstream in the genome.  
uid:74|KX208106.1: Sequence cannot be extended sufficiently by unalined portion of query. THIS IS PROBABLY FRAGMENT! Trimmed downstream.

### Hit: AF221909.1

AF221909.1 Microtus ochrogaster telomerase RNA gene, sequence

```
?

```
This is BLAST alignment as read from the input file
```

Score = 47.0 bits (43.7), Expect = 5.85E+00
 Identities = 28/31 (90%), Gaps = 0/31 (0%)
 Strand = Plus/Plus
Query 358 TGCTGTCGCGAAGAGTTCGGCTCTGTCAGCC 388
          ||| | ||||||||||||| |||||||||||    
Sbjct 344 TGCCGCCGCGAAGAGTTCGTCTCTGTCAGCC 374
```

Report:

|  |  |
| --- | --- |
| sequence start ?  ``` Start position of the estimated full-length sequence in genome. Start index < end index. ``` : | 13 |
| sequence end ?  ``` End position of the estimated full-length sequence in genome. Start index < end index. ``` : | 496 |
| bit score (CM) ?  ``` The score for aligning estimated full-length sequence to CM model   (computed by RSEARCH -> default,   infered from Rfam or provided by user) ``` : | 0.14 |
| Homology estimate ?  ``` Quick homology estimate:   Not homologous: bit score < 0   Homologous: bit score > 20 and bit score > 0.5 * query length   Uncertain otherwise ``` : | Uncertain |

Estimated full-length sequence:


?

```
Click checkbox to select multiple seuqences.
Fasta header format:
  UID|accession.versionSTRAND start-end
```

>uid:75|AF221909.1fw 13-496
GCAAACACGGGCGAGAGUUGGGCGCCGACCAAUCAGCGAGCGCGCUGACCCAGGUAUUUA
AGGGCGUCCCGACGGGCGGACGGGCGACGCGCGCCUCUUCUAACCCUAAAAACUGGAGCU
GUAGGUGUUGCUCUUUCAGCGUCGCCCGCUGUUUUUCUCGCUGGCUUUCAGCGGGCCAGA
AAGUUCAGACCUCUCAGCAGAUCGUCGCGUCGUUCUCAACCACAAAAAAUGCCAGCGCAA
AGCGCGUCAGCCUAGAACCUUGCGGCCCCGGGCCGCCCAGCCCCGCACCCGCCUUGAGGC
CGCGGUUGGCCUGGAGUUCUCCGGACUCCGCUGCCGCCGCGAAGAGUUCGUCUCUGUCAG
CCGCGGAGUAUCAGGGGCUGGGGCCAGGCCCGGACAGCGUCGCAAGUACAGUAACGGAGC
UGGUCCUUGUUCGGUGGCUUCCCUGAGCUGUAGGAAGUACACCCAGAGCUCGGCUCCUAC
AACC

C-A-r-Rc


?

```
Visualisation of predicted secondary structure.
To save the image:
  Right click on the image -> Save Image as.
```


centroid


?

```
Visualisation of predicted secondary structure.
To save the image:
  Right click on the image -> Save Image as.
```


fq-sub


?

```
Visualisation of predicted secondary structure.
To save the image:
  Right click on the image -> Save Image as.
```

Load Sequence viewer

uid:75|AF221909.1: Sequence cannot be extended sufficiently. Missing nt downstream in the genome.  
TurboFold: Number of sequences is less then required. n=2 (4)  
uid:75|AF221909.1: Sequence cannot be extended sufficiently by unaligned portion of query. THIS IS PROBABLY FRAGMENT! Trimmed upstream.  
uid:75|AF221909.1: Sequence cannot be extended sufficiently. Missing -43 nt upstream in the genome.  
uid:75|AF221909.1: Sequence cannot be extended sufficiently by unalined portion of query. THIS IS PROBABLY FRAGMENT! Trimmed downstream.

### Hit: KX208094.1

KX208094.1 Rentapia hosii voucher TNHC-GDC 31201 piccolo presynaptic cytomatrix protein (PCLO) gene, partial cds

```
?

```
This is BLAST alignment as read from the input file
```

Score = 46.0 bits (42.8), Expect = 5.85E+00
 Identities = 40/49 (82%), Gaps = 3/49 (6%)
 Strand = Plus/Plus
Query 136 GGCAAAAAAAAGTTGGGAGCAGCAACGGCAAGCAAAAAAAAAGTTCCAG 184
          |||||| | || |  ||||||||||| |||| |||| ||||||||| ||    
Sbjct 674 GGCAAAGAGAATT--GGAGCAGCAACAGCAA-CAAAGAAAAAGTTCTAG 719
```

Report:

|  |  |
| --- | --- |
| sequence start ?  ``` Start position of the estimated full-length sequence in genome. Start index < end index. ``` : | 536 |
| sequence end ?  ``` End position of the estimated full-length sequence in genome. Start index < end index. ``` : | 927 |
| bit score (CM) ?  ``` The score for aligning estimated full-length sequence to CM model   (computed by RSEARCH -> default,   infered from Rfam or provided by user) ``` : | -39.62 |
| Homology estimate ?  ``` Quick homology estimate:   Not homologous: bit score < 0   Homologous: bit score > 20 and bit score > 0.5 * query length   Uncertain otherwise ``` : | Not homologous |

Estimated full-length sequence:


?

```
Click checkbox to select multiple seuqences.
Fasta header format:
  UID|accession.versionSTRAND start-end
```

>uid:76|KX208094.1fw 536-927
ACAAAAAGAUGUCAGGAGAAAGCAAACAGCAGAGAAAAGCAAGGCAUCGAUCUCAUGGAC
CAGUUCUACCCACUAUUGAAGACUCUUCUGAAGAAGAAGAAUUAAGAGAAGAGGAAGAAC
UUUUGAAAGAGCAGGAAAGGCAAAGAGAAUUGGAGCAGCAACAGCAACAAAGAAAAAGUU
CUAGUAAAAAAUCCAAAAAAGACAAAGAUGAACUAAGAGCACAGAGAAGGAGAGAGAGAC
CAAAGACACCUCCGAGCAACUUAUCUCCUAUUGAAGAUGCUUCUCCAACAGAAGAAUUGC
GUCAAGCAGCUGAAAUGGAAGAACUUCAUAGAUCUUCAUGUUCAGAGUAUUCACCUAGUA
UUGAGUCAGAACCAGAAGGAUUUGAAAUAAGU

C-A-r-Rc


?

```
Visualisation of predicted secondary structure.
To save the image:
  Right click on the image -> Save Image as.
```


centroid


?

```
Visualisation of predicted secondary structure.
To save the image:
  Right click on the image -> Save Image as.
```


fq-sub


?

```
Visualisation of predicted secondary structure.
To save the image:
  Right click on the image -> Save Image as.
```

Load Sequence viewer

TurboFold: Number of sequences is less then required. n=2 (4)  
uid:76|KX208094.1: Sequence cannot be extended sufficiently. Missing nt downstream in the genome.  
uid:76|KX208094.1: Sequence cannot be extended sufficiently by unalined portion of query. THIS IS PROBABLY FRAGMENT! Trimmed downstream.

### Hit: XR\_002743649.1

XR\_002743649.1 PREDICTED: Felis catus uncharacterized LOC109501310 (LOC109501310), transcript variant X4, ncRNA

```
?

```
This is BLAST alignment as read from the input file
```

Score = 46.0 bits (42.8), Expect = 5.85E+00
 Identities = 35/43 (81%), Gaps = 0/43 (0%)
 Strand = Plus/Minus
Query 385 AGCCCGGCTGGGTCCGGGTGGGGGCCGGCACAGAGGGACACAC 427
          |||| ||||||  ||||||||||| | || ||| ||||| |||    
Sbjct 560 AGCCAGGCTGGACCCGGGTGGGGGGCTGCCCAGCGGGACCCAC 518
```

Report:

|  |  |
| --- | --- |
| sequence start ?  ``` Start position of the estimated full-length sequence in genome. Start index < end index. ``` : | 387 |
| sequence end ?  ``` End position of the estimated full-length sequence in genome. Start index < end index. ``` : | 943 |
| bit score (CM) ?  ``` The score for aligning estimated full-length sequence to CM model   (computed by RSEARCH -> default,   infered from Rfam or provided by user) ``` : | -43.64 |
| Homology estimate ?  ``` Quick homology estimate:   Not homologous: bit score < 0   Homologous: bit score > 20 and bit score > 0.5 * query length   Uncertain otherwise ``` : | Not homologous |

Estimated full-length sequence:


?

```
Click checkbox to select multiple seuqences.
Fasta header format:
  UID|accession.versionSTRAND start-end
```

>uid:77|XR\_002743649.1rc 387-943
UUUCAUCUCAAUUCGCAGGAGCACUUGAGAGCUGACGUCAGACACUCAGGAGGAGGGCAC
UGCUUAGCCGGAAGUCUUAACCAUUACUGAUGAUUCUGCACAGGGCUCCUUUAUCGGGCU
GGAUACUCUCUAGGUCCCUCUGACCCAGGGGUCGGGGGUGCCGUGCCUGCGGAGGCCCCU
GAUCUGCUCAUUCUACCCCCAUUCGGUUGCCAUGAAGCCAAAAGAGCCUGGGGGAGGGGG
CAAAUGGCUGGCCUGGAGGGAGGCGACCCGAUUUCACCGACUGAAACAGUUCUGUGACUC
CCAAGUGCCUCAUGUUGAAAUCAGGAGACAUCACAGAGGUAUCUCCUGUUCGGACAGCGC
ACCCUGCCCCAUAUCACACGGCCGGGCUGGGCUGAGCCAGGCUGGACCCGGGUGGGGGGC
UGCCCAGCGGGACCCACACUGUGCGUCUGUCGCUCAGAACCGCAGCCCCCUUGCUCUGCC
CCUUUGUGAGCUAAACAGGACAAGUGGACAGGAAGGUGCCCAGCACCUCCUCACCCUGUC
CACCAACAUCUGUCCCC

C-A-r-Rc


?

```
Visualisation of predicted secondary structure.
To save the image:
  Right click on the image -> Save Image as.
```


centroid


?

```
Visualisation of predicted secondary structure.
To save the image:
  Right click on the image -> Save Image as.
```


fq-sub


?

```
Visualisation of predicted secondary structure.
To save the image:
  Right click on the image -> Save Image as.
```

Load Sequence viewer

TurboFold: Number of sequences is less then required. n=2 (4)

### Hit: XR\_002743648.1

XR\_002743648.1 PREDICTED: Felis catus uncharacterized LOC109501310 (LOC109501310), transcript variant X3, ncRNA

```
?

```
This is BLAST alignment as read from the input file
```

Score = 46.0 bits (42.8), Expect = 5.85E+00
 Identities = 35/43 (81%), Gaps = 0/43 (0%)
 Strand = Plus/Minus
Query 385 AGCCCGGCTGGGTCCGGGTGGGGGCCGGCACAGAGGGACACAC 427
          |||| ||||||  ||||||||||| | || ||| ||||| |||    
Sbjct 546 AGCCAGGCTGGACCCGGGTGGGGGGCTGCCCAGCGGGACCCAC 504
```

Report:

|  |  |
| --- | --- |
| sequence start ?  ``` Start position of the estimated full-length sequence in genome. Start index < end index. ``` : | 373 |
| sequence end ?  ``` End position of the estimated full-length sequence in genome. Start index < end index. ``` : | 929 |
| bit score (CM) ?  ``` The score for aligning estimated full-length sequence to CM model   (computed by RSEARCH -> default,   infered from Rfam or provided by user) ``` : | -43.64 |
| Homology estimate ?  ``` Quick homology estimate:   Not homologous: bit score < 0   Homologous: bit score > 20 and bit score > 0.5 * query length   Uncertain otherwise ``` : | Not homologous |

Estimated full-length sequence:


?

```
Click checkbox to select multiple seuqences.
Fasta header format:
  UID|accession.versionSTRAND start-end
```

>uid:78|XR\_002743648.1rc 373-929
UUUCAUCUCAAUUCGCAGGAGCACUUGAGAGCUGACGUCAGACACUCAGGAGGAGGGCAC
UGCUUAGCCGGAAGUCUUAACCAUUACUGAUGAUUCUGCACAGGGCUCCUUUAUCGGGCU
GGAUACUCUCUAGGUCCCUCUGACCCAGGGGUCGGGGGUGCCGUGCCUGCGGAGGCCCCU
GAUCUGCUCAUUCUACCCCCAUUCGGUUGCCAUGAAGCCAAAAGAGCCUGGGGGAGGGGG
CAAAUGGCUGGCCUGGAGGGAGGCGACCCGAUUUCACCGACUGAAACAGUUCUGUGACUC
CCAAGUGCCUCAUGUUGAAAUCAGGAGACAUCACAGAGGUAUCUCCUGUUCGGACAGCGC
ACCCUGCCCCAUAUCACACGGCCGGGCUGGGCUGAGCCAGGCUGGACCCGGGUGGGGGGC
UGCCCAGCGGGACCCACACUGUGCGUCUGUCGCUCAGAACCGCAGCCCCCUUGCUCUGCC
CCUUUGUGAGCUAAACAGGACAAGUGGACAGGAAGGUGCCCAGCACCUCCUCACCCUGUC
CACCAACAUCUGUCCCC

C-A-r-Rc


?

```
Visualisation of predicted secondary structure.
To save the image:
  Right click on the image -> Save Image as.
```


centroid


?

```
Visualisation of predicted secondary structure.
To save the image:
  Right click on the image -> Save Image as.
```


fq-sub


?

```
Visualisation of predicted secondary structure.
To save the image:
  Right click on the image -> Save Image as.
```

Load Sequence viewer

TurboFold: Number of sequences is less then required. n=2 (4)

### Hit: XR\_002743647.1

XR\_002743647.1 PREDICTED: Felis catus uncharacterized LOC109501310 (LOC109501310), transcript variant X2, ncRNA

```
?

```
This is BLAST alignment as read from the input file
```

Score = 46.0 bits (42.8), Expect = 5.85E+00
 Identities = 35/43 (81%), Gaps = 0/43 (0%)
 Strand = Plus/Minus
Query 385 AGCCCGGCTGGGTCCGGGTGGGGGCCGGCACAGAGGGACACAC 427
          |||| ||||||  ||||||||||| | || ||| ||||| |||    
Sbjct 560 AGCCAGGCTGGACCCGGGTGGGGGGCTGCCCAGCGGGACCCAC 518
```

Report:

|  |  |
| --- | --- |
| sequence start ?  ``` Start position of the estimated full-length sequence in genome. Start index < end index. ``` : | 387 |
| sequence end ?  ``` End position of the estimated full-length sequence in genome. Start index < end index. ``` : | 943 |
| bit score (CM) ?  ``` The score for aligning estimated full-length sequence to CM model   (computed by RSEARCH -> default,   infered from Rfam or provided by user) ``` : | -43.64 |
| Homology estimate ?  ``` Quick homology estimate:   Not homologous: bit score < 0   Homologous: bit score > 20 and bit score > 0.5 * query length   Uncertain otherwise ``` : | Not homologous |

Estimated full-length sequence:


?

```
Click checkbox to select multiple seuqences.
Fasta header format:
  UID|accession.versionSTRAND start-end
```

>uid:79|XR\_002743647.1rc 387-943
UUUCAUCUCAAUUCGCAGGAGCACUUGAGAGCUGACGUCAGACACUCAGGAGGAGGGCAC
UGCUUAGCCGGAAGUCUUAACCAUUACUGAUGAUUCUGCACAGGGCUCCUUUAUCGGGCU
GGAUACUCUCUAGGUCCCUCUGACCCAGGGGUCGGGGGUGCCGUGCCUGCGGAGGCCCCU
GAUCUGCUCAUUCUACCCCCAUUCGGUUGCCAUGAAGCCAAAAGAGCCUGGGGGAGGGGG
CAAAUGGCUGGCCUGGAGGGAGGCGACCCGAUUUCACCGACUGAAACAGUUCUGUGACUC
CCAAGUGCCUCAUGUUGAAAUCAGGAGACAUCACAGAGGUAUCUCCUGUUCGGACAGCGC
ACCCUGCCCCAUAUCACACGGCCGGGCUGGGCUGAGCCAGGCUGGACCCGGGUGGGGGGC
UGCCCAGCGGGACCCACACUGUGCGUCUGUCGCUCAGAACCGCAGCCCCCUUGCUCUGCC
CCUUUGUGAGCUAAACAGGACAAGUGGACAGGAAGGUGCCCAGCACCUCCUCACCCUGUC
CACCAACAUCUGUCCCC

C-A-r-Rc


?

```
Visualisation of predicted secondary structure.
To save the image:
  Right click on the image -> Save Image as.
```


centroid


?

```
Visualisation of predicted secondary structure.
To save the image:
  Right click on the image -> Save Image as.
```


fq-sub


?

```
Visualisation of predicted secondary structure.
To save the image:
  Right click on the image -> Save Image as.
```

Load Sequence viewer

TurboFold: Number of sequences is less then required. n=2 (4)

### Hit: XR\_002159268.2

XR\_002159268.2 PREDICTED: Felis catus uncharacterized LOC109501310 (LOC109501310), transcript variant X1, ncRNA

```
?

```
This is BLAST alignment as read from the input file
```

Score = 46.0 bits (42.8), Expect = 5.85E+00
 Identities = 35/43 (81%), Gaps = 0/43 (0%)
 Strand = Plus/Minus
Query 385 AGCCCGGCTGGGTCCGGGTGGGGGCCGGCACAGAGGGACACAC 427
          |||| ||||||  ||||||||||| | || ||| ||||| |||    
Sbjct 560 AGCCAGGCTGGACCCGGGTGGGGGGCTGCCCAGCGGGACCCAC 518
```

Report:

|  |  |
| --- | --- |
| sequence start ?  ``` Start position of the estimated full-length sequence in genome. Start index < end index. ``` : | 387 |
| sequence end ?  ``` End position of the estimated full-length sequence in genome. Start index < end index. ``` : | 943 |
| bit score (CM) ?  ``` The score for aligning estimated full-length sequence to CM model   (computed by RSEARCH -> default,   infered from Rfam or provided by user) ``` : | -43.64 |
| Homology estimate ?  ``` Quick homology estimate:   Not homologous: bit score < 0   Homologous: bit score > 20 and bit score > 0.5 * query length   Uncertain otherwise ``` : | Not homologous |

Estimated full-length sequence:


?

```
Click checkbox to select multiple seuqences.
Fasta header format:
  UID|accession.versionSTRAND start-end
```

>uid:80|XR\_002159268.2rc 387-943
UUUCAUCUCAAUUCGCAGGAGCACUUGAGAGCUGACGUCAGACACUCAGGAGGAGGGCAC
UGCUUAGCCGGAAGUCUUAACCAUUACUGAUGAUUCUGCACAGGGCUCCUUUAUCGGGCU
GGAUACUCUCUAGGUCCCUCUGACCCAGGGGUCGGGGGUGCCGUGCCUGCGGAGGCCCCU
GAUCUGCUCAUUCUACCCCCAUUCGGUUGCCAUGAAGCCAAAAGAGCCUGGGGGAGGGGG
CAAAUGGCUGGCCUGGAGGGAGGCGACCCGAUUUCACCGACUGAAACAGUUCUGUGACUC
CCAAGUGCCUCAUGUUGAAAUCAGGAGACAUCACAGAGGUAUCUCCUGUUCGGACAGCGC
ACCCUGCCCCAUAUCACACGGCCGGGCUGGGCUGAGCCAGGCUGGACCCGGGUGGGGGGC
UGCCCAGCGGGACCCACACUGUGCGUCUGUCGCUCAGAACCGCAGCCCCCUUGCUCUGCC
CCUUUGUGAGCUAAACAGGACAAGUGGACAGGAAGGUGCCCAGCACCUCCUCACCCUGUC
CACCAACAUCUGUCCCC

C-A-r-Rc


?

```
Visualisation of predicted secondary structure.
To save the image:
  Right click on the image -> Save Image as.
```


centroid


?

```
Visualisation of predicted secondary structure.
To save the image:
  Right click on the image -> Save Image as.
```


fq-sub


?

```
Visualisation of predicted secondary structure.
To save the image:
  Right click on the image -> Save Image as.
```

Load Sequence viewer

TurboFold: Number of sequences is less then required. n=2 (4)

### Hit: XR\_002616901.1

XR\_002616901.1 PREDICTED: Canis lupus familiaris uncharacterized LOC106560091 (LOC106560091), transcript variant X6, ncRNA

```
?

```
This is BLAST alignment as read from the input file
```

Score = 46.0 bits (42.8), Expect = 5.85E+00
 Identities = 34/40 (85%), Gaps = 1/40 (2%)
 Strand = Plus/Minus
Query 378 CTCTGTCAGCCCGGCTGGGTCCGGGTGGGGGCCGGCACAG 417
          ||||| | |||||| ||||||||||||||  |||| ||||    
Sbjct  53 CTCTGCCGGCCCGGGTGGGTCCGGGTGGGTCCCGG-ACAG 15
```

Report:

|  |  |
| --- | --- |
| sequence start ?  ``` Start position of the estimated full-length sequence in genome. Start index < end index. ``` : | 28 |
| sequence end ?  ``` End position of the estimated full-length sequence in genome. Start index < end index. ``` : | 460 |
| bit score (CM) ?  ``` The score for aligning estimated full-length sequence to CM model   (computed by RSEARCH -> default,   infered from Rfam or provided by user) ``` : | -30.86 |
| Homology estimate ?  ``` Quick homology estimate:   Not homologous: bit score < 0   Homologous: bit score > 20 and bit score > 0.5 * query length   Uncertain otherwise ``` : | Not homologous |

Estimated full-length sequence:


?

```
Click checkbox to select multiple seuqences.
Fasta header format:
  UID|accession.versionSTRAND start-end
```

>uid:81|XR\_002616901.1rc 28-460
AAACAAGGCAGAGAGAGUCCCCGUCUCCAAGUAGCUUGUAUUCUCUCAGCGUCUCCAACU
UUGUCUGGGGUCCUGCUCCUCUGCCUCCUCCCGUUGCCAGGCGCGGGCCGGGCACCGCGG
GGCUCUGUUCCCAGCCUCCGUCUUCCAUCUCGACCCCCGGCUCUGCCUCUCCUUGGGCCU
CCUCCUCUGUCCGUCUUUCCUCGCUUGUGCAUUUCUCUCCAUCUCGCAGGUCUCUGCCAU
CUCCGUCUCCAGAGCUGUCUCUCCCCCUUGUCUUCGAAGCCUCCUCUCUCCCACCUGCAG
CUCUGUGCGUCUCCCCACCUCUUGUCUCUCCCUGCCUCCCAUCUCUCCCCAUCUCUGUCU
UUCUCCGCCCCGUCCCCGGUCUCUGCCGGCCCGGGUGGGUCCGGGUGGGUCCCGGACAGC
CUGGCCCGGGCGC

C-A-r-Rc


?

```
Visualisation of predicted secondary structure.
To save the image:
  Right click on the image -> Save Image as.
```


centroid


?

```
Visualisation of predicted secondary structure.
To save the image:
  Right click on the image -> Save Image as.
```


fq-sub


?

```
Visualisation of predicted secondary structure.
To save the image:
  Right click on the image -> Save Image as.
```

Load Sequence viewer

TurboFold: Number of sequences is less then required. n=2 (4)  
uid:81|XR\_002616901.1: Sequence cannot be extended sufficiently by unaligned portion of query. THIS IS PROBABLY FRAGMENT! Trimmed upstream.  
uid:81|XR\_002616901.1: Sequence cannot be extended sufficiently. Missing -146 nt upstream in the genome.

### Hit: XR\_002616900.1

XR\_002616900.1 PREDICTED: Canis lupus familiaris uncharacterized LOC106560091 (LOC106560091), transcript variant X5, ncRNA

```
?

```
This is BLAST alignment as read from the input file
```

Score = 46.0 bits (42.8), Expect = 5.85E+00
 Identities = 34/40 (85%), Gaps = 1/40 (2%)
 Strand = Plus/Minus
Query 378 CTCTGTCAGCCCGGCTGGGTCCGGGTGGGGGCCGGCACAG 417
          ||||| | |||||| ||||||||||||||  |||| ||||    
Sbjct  53 CTCTGCCGGCCCGGGTGGGTCCGGGTGGGTCCCGG-ACAG 15
```

Report:

|  |  |
| --- | --- |
| sequence start ?  ``` Start position of the estimated full-length sequence in genome. Start index < end index. ``` : | 28 |
| sequence end ?  ``` End position of the estimated full-length sequence in genome. Start index < end index. ``` : | 460 |
| bit score (CM) ?  ``` The score for aligning estimated full-length sequence to CM model   (computed by RSEARCH -> default,   infered from Rfam or provided by user) ``` : | -30.86 |
| Homology estimate ?  ``` Quick homology estimate:   Not homologous: bit score < 0   Homologous: bit score > 20 and bit score > 0.5 * query length   Uncertain otherwise ``` : | Not homologous |

Estimated full-length sequence:


?

```
Click checkbox to select multiple seuqences.
Fasta header format:
  UID|accession.versionSTRAND start-end
```

>uid:82|XR\_002616900.1rc 28-460
AAACAAGGCAGAGAGAGUCCCCGUCUCCAAGUAGCUUGUAUUCUCUCAGCGUCUCCAACU
UUGUCUGGGGUCCUGCUCCUCUGCCUCCUCCCGUUGCCAGGCGCGGGCCGGGCACCGCGG
GGCUCUGUUCCCAGCCUCCGUCUUCCAUCUCGACCCCCGGCUCUGCCUCUCCUUGGGCCU
CCUCCUCUGUCCGUCUUUCCUCGCUUGUGCAUUUCUCUCCAUCUCGCAGGUCUCUGCCAU
CUCCGUCUCCAGAGCUGUCUCUCCCCCUUGUCUUCGAAGCCUCCUCUCUCCCACCUGCAG
CUCUGUGCGUCUCCCCACCUCUUGUCUCUCCCUGCCUCCCAUCUCUCCCCAUCUCUGUCU
UUCUCCGCCCCGUCCCCGGUCUCUGCCGGCCCGGGUGGGUCCGGGUGGGUCCCGGACAGC
CUGGCCCGGGCGC

C-A-r-Rc


?

```
Visualisation of predicted secondary structure.
To save the image:
  Right click on the image -> Save Image as.
```


centroid


?

```
Visualisation of predicted secondary structure.
To save the image:
  Right click on the image -> Save Image as.
```


fq-sub


?

```
Visualisation of predicted secondary structure.
To save the image:
  Right click on the image -> Save Image as.
```

Load Sequence viewer

TurboFold: Number of sequences is less then required. n=2 (4)  
uid:82|XR\_002616900.1: Sequence cannot be extended sufficiently. Missing -146 nt upstream in the genome.  
uid:82|XR\_002616900.1: Sequence cannot be extended sufficiently by unaligned portion of query. THIS IS PROBABLY FRAGMENT! Trimmed upstream.

### Hit: XR\_002616899.1

XR\_002616899.1 PREDICTED: Canis lupus familiaris uncharacterized LOC106560091 (LOC106560091), transcript variant X4, ncRNA

```
?

```
This is BLAST alignment as read from the input file
```

Score = 46.0 bits (42.8), Expect = 5.85E+00
 Identities = 34/40 (85%), Gaps = 1/40 (2%)
 Strand = Plus/Minus
Query 378 CTCTGTCAGCCCGGCTGGGTCCGGGTGGGGGCCGGCACAG 417
          ||||| | |||||| ||||||||||||||  |||| ||||    
Sbjct  53 CTCTGCCGGCCCGGGTGGGTCCGGGTGGGTCCCGG-ACAG 15
```

Report:

|  |  |
| --- | --- |
| sequence start ?  ``` Start position of the estimated full-length sequence in genome. Start index < end index. ``` : | 28 |
| sequence end ?  ``` End position of the estimated full-length sequence in genome. Start index < end index. ``` : | 460 |
| bit score (CM) ?  ``` The score for aligning estimated full-length sequence to CM model   (computed by RSEARCH -> default,   infered from Rfam or provided by user) ``` : | -30.86 |
| Homology estimate ?  ``` Quick homology estimate:   Not homologous: bit score < 0   Homologous: bit score > 20 and bit score > 0.5 * query length   Uncertain otherwise ``` : | Not homologous |

Estimated full-length sequence:


?

```
Click checkbox to select multiple seuqences.
Fasta header format:
  UID|accession.versionSTRAND start-end
```

>uid:83|XR\_002616899.1rc 28-460
AAACAAGGCAGAGAGAGUCCCCGUCUCCAAGUAGCUUGUAUUCUCUCAGCGUCUCCAACU
UUGUCUGGGGUCCUGCUCCUCUGCCUCCUCCCGUUGCCAGGCGCGGGCCGGGCACCGCGG
GGCUCUGUUCCCAGCCUCCGUCUUCCAUCUCGACCCCCGGCUCUGCCUCUCCUUGGGCCU
CCUCCUCUGUCCGUCUUUCCUCGCUUGUGCAUUUCUCUCCAUCUCGCAGGUCUCUGCCAU
CUCCGUCUCCAGAGCUGUCUCUCCCCCUUGUCUUCGAAGCCUCCUCUCUCCCACCUGCAG
CUCUGUGCGUCUCCCCACCUCUUGUCUCUCCCUGCCUCCCAUCUCUCCCCAUCUCUGUCU
UUCUCCGCCCCGUCCCCGGUCUCUGCCGGCCCGGGUGGGUCCGGGUGGGUCCCGGACAGC
CUGGCCCGGGCGC

C-A-r-Rc


?

```
Visualisation of predicted secondary structure.
To save the image:
  Right click on the image -> Save Image as.
```


centroid


?

```
Visualisation of predicted secondary structure.
To save the image:
  Right click on the image -> Save Image as.
```


fq-sub


?

```
Visualisation of predicted secondary structure.
To save the image:
  Right click on the image -> Save Image as.
```

Load Sequence viewer

TurboFold: Number of sequences is less then required. n=2 (4)  
uid:83|XR\_002616899.1: Sequence cannot be extended sufficiently. Missing -146 nt upstream in the genome.  
uid:83|XR\_002616899.1: Sequence cannot be extended sufficiently by unaligned portion of query. THIS IS PROBABLY FRAGMENT! Trimmed upstream.

### Hit: XR\_001318394.2

XR\_001318394.2 PREDICTED: Canis lupus familiaris uncharacterized LOC106560091 (LOC106560091), transcript variant X3, ncRNA

```
?

```
This is BLAST alignment as read from the input file
```

Score = 46.0 bits (42.8), Expect = 5.85E+00
 Identities = 34/40 (85%), Gaps = 1/40 (2%)
 Strand = Plus/Minus
Query 378 CTCTGTCAGCCCGGCTGGGTCCGGGTGGGGGCCGGCACAG 417
          ||||| | |||||| ||||||||||||||  |||| ||||    
Sbjct  53 CTCTGCCGGCCCGGGTGGGTCCGGGTGGGTCCCGG-ACAG 15
```

Report:

|  |  |
| --- | --- |
| sequence start ?  ``` Start position of the estimated full-length sequence in genome. Start index < end index. ``` : | 28 |
| sequence end ?  ``` End position of the estimated full-length sequence in genome. Start index < end index. ``` : | 460 |
| bit score (CM) ?  ``` The score for aligning estimated full-length sequence to CM model   (computed by RSEARCH -> default,   infered from Rfam or provided by user) ``` : | -30.86 |
| Homology estimate ?  ``` Quick homology estimate:   Not homologous: bit score < 0   Homologous: bit score > 20 and bit score > 0.5 * query length   Uncertain otherwise ``` : | Not homologous |

Estimated full-length sequence:


?

```
Click checkbox to select multiple seuqences.
Fasta header format:
  UID|accession.versionSTRAND start-end
```

>uid:84|XR\_001318394.2rc 28-460
AAACAAGGCAGAGAGAGUCCCCGUCUCCAAGUAGCUUGUAUUCUCUCAGCGUCUCCAACU
UUGUCUGGGGUCCUGCUCCUCUGCCUCCUCCCGUUGCCAGGCGCGGGCCGGGCACCGCGG
GGCUCUGUUCCCAGCCUCCGUCUUCCAUCUCGACCCCCGGCUCUGCCUCUCCUUGGGCCU
CCUCCUCUGUCCGUCUUUCCUCGCUUGUGCAUUUCUCUCCAUCUCGCAGGUCUCUGCCAU
CUCCGUCUCCAGAGCUGUCUCUCCCCCUUGUCUUCGAAGCCUCCUCUCUCCCACCUGCAG
CUCUGUGCGUCUCCCCACCUCUUGUCUCUCCCUGCCUCCCAUCUCUCCCCAUCUCUGUCU
UUCUCCGCCCCGUCCCCGGUCUCUGCCGGCCCGGGUGGGUCCGGGUGGGUCCCGGACAGC
CUGGCCCGGGCGC

C-A-r-Rc


?

```
Visualisation of predicted secondary structure.
To save the image:
  Right click on the image -> Save Image as.
```


centroid


?

```
Visualisation of predicted secondary structure.
To save the image:
  Right click on the image -> Save Image as.
```


fq-sub


?

```
Visualisation of predicted secondary structure.
To save the image:
  Right click on the image -> Save Image as.
```

Load Sequence viewer

TurboFold: Number of sequences is less then required. n=2 (4)  
uid:84|XR\_001318394.2: Sequence cannot be extended sufficiently. Missing -146 nt upstream in the genome.  
uid:84|XR\_001318394.2: Sequence cannot be extended sufficiently by unaligned portion of query. THIS IS PROBABLY FRAGMENT! Trimmed upstream.

### Hit: XR\_002616898.1

XR\_002616898.1 PREDICTED: Canis lupus familiaris uncharacterized LOC106560091 (LOC106560091), transcript variant X2, ncRNA

```
?

```
This is BLAST alignment as read from the input file
```

Score = 46.0 bits (42.8), Expect = 5.85E+00
 Identities = 34/40 (85%), Gaps = 1/40 (2%)
 Strand = Plus/Minus
Query 378 CTCTGTCAGCCCGGCTGGGTCCGGGTGGGGGCCGGCACAG 417
          ||||| | |||||| ||||||||||||||  |||| ||||    
Sbjct  53 CTCTGCCGGCCCGGGTGGGTCCGGGTGGGTCCCGG-ACAG 15
```

Report:

|  |  |
| --- | --- |
| sequence start ?  ``` Start position of the estimated full-length sequence in genome. Start index < end index. ``` : | 28 |
| sequence end ?  ``` End position of the estimated full-length sequence in genome. Start index < end index. ``` : | 460 |
| bit score (CM) ?  ``` The score for aligning estimated full-length sequence to CM model   (computed by RSEARCH -> default,   infered from Rfam or provided by user) ``` : | -30.86 |
| Homology estimate ?  ``` Quick homology estimate:   Not homologous: bit score < 0   Homologous: bit score > 20 and bit score > 0.5 * query length   Uncertain otherwise ``` : | Not homologous |

Estimated full-length sequence:


?

```
Click checkbox to select multiple seuqences.
Fasta header format:
  UID|accession.versionSTRAND start-end
```

>uid:85|XR\_002616898.1rc 28-460
AAACAAGGCAGAGAGAGUCCCCGUCUCCAAGUAGCUUGUAUUCUCUCAGCGUCUCCAACU
UUGUCUGGGGUCCUGCUCCUCUGCCUCCUCCCGUUGCCAGGCGCGGGCCGGGCACCGCGG
GGCUCUGUUCCCAGCCUCCGUCUUCCAUCUCGACCCCCGGCUCUGCCUCUCCUUGGGCCU
CCUCCUCUGUCCGUCUUUCCUCGCUUGUGCAUUUCUCUCCAUCUCGCAGGUCUCUGCCAU
CUCCGUCUCCAGAGCUGUCUCUCCCCCUUGUCUUCGAAGCCUCCUCUCUCCCACCUGCAG
CUCUGUGCGUCUCCCCACCUCUUGUCUCUCCCUGCCUCCCAUCUCUCCCCAUCUCUGUCU
UUCUCCGCCCCGUCCCCGGUCUCUGCCGGCCCGGGUGGGUCCGGGUGGGUCCCGGACAGC
CUGGCCCGGGCGC

C-A-r-Rc


?

```
Visualisation of predicted secondary structure.
To save the image:
  Right click on the image -> Save Image as.
```


centroid


?

```
Visualisation of predicted secondary structure.
To save the image:
  Right click on the image -> Save Image as.
```


fq-sub


?

```
Visualisation of predicted secondary structure.
To save the image:
  Right click on the image -> Save Image as.
```

Load Sequence viewer

uid:85|XR\_002616898.1: Sequence cannot be extended sufficiently. Missing -146 nt upstream in the genome.  
TurboFold: Number of sequences is less then required. n=2 (4)  
uid:85|XR\_002616898.1: Sequence cannot be extended sufficiently by unaligned portion of query. THIS IS PROBABLY FRAGMENT! Trimmed upstream.

### Hit: XR\_002616897.1

XR\_002616897.1 PREDICTED: Canis lupus familiaris uncharacterized LOC106560091 (LOC106560091), transcript variant X1, ncRNA

```
?

```
This is BLAST alignment as read from the input file
```

Score = 46.0 bits (42.8), Expect = 5.85E+00
 Identities = 34/40 (85%), Gaps = 1/40 (2%)
 Strand = Plus/Minus
Query 378 CTCTGTCAGCCCGGCTGGGTCCGGGTGGGGGCCGGCACAG 417
          ||||| | |||||| ||||||||||||||  |||| ||||    
Sbjct  53 CTCTGCCGGCCCGGGTGGGTCCGGGTGGGTCCCGG-ACAG 15
```

Report:

|  |  |
| --- | --- |
| sequence start ?  ``` Start position of the estimated full-length sequence in genome. Start index < end index. ``` : | 28 |
| sequence end ?  ``` End position of the estimated full-length sequence in genome. Start index < end index. ``` : | 460 |
| bit score (CM) ?  ``` The score for aligning estimated full-length sequence to CM model   (computed by RSEARCH -> default,   infered from Rfam or provided by user) ``` : | -30.86 |
| Homology estimate ?  ``` Quick homology estimate:   Not homologous: bit score < 0   Homologous: bit score > 20 and bit score > 0.5 * query length   Uncertain otherwise ``` : | Not homologous |

Estimated full-length sequence:


?

```
Click checkbox to select multiple seuqences.
Fasta header format:
  UID|accession.versionSTRAND start-end
```

>uid:86|XR\_002616897.1rc 28-460
AAACAAGGCAGAGAGAGUCCCCGUCUCCAAGUAGCUUGUAUUCUCUCAGCGUCUCCAACU
UUGUCUGGGGUCCUGCUCCUCUGCCUCCUCCCGUUGCCAGGCGCGGGCCGGGCACCGCGG
GGCUCUGUUCCCAGCCUCCGUCUUCCAUCUCGACCCCCGGCUCUGCCUCUCCUUGGGCCU
CCUCCUCUGUCCGUCUUUCCUCGCUUGUGCAUUUCUCUCCAUCUCGCAGGUCUCUGCCAU
CUCCGUCUCCAGAGCUGUCUCUCCCCCUUGUCUUCGAAGCCUCCUCUCUCCCACCUGCAG
CUCUGUGCGUCUCCCCACCUCUUGUCUCUCCCUGCCUCCCAUCUCUCCCCAUCUCUGUCU
UUCUCCGCCCCGUCCCCGGUCUCUGCCGGCCCGGGUGGGUCCGGGUGGGUCCCGGACAGC
CUGGCCCGGGCGC

C-A-r-Rc


?

```
Visualisation of predicted secondary structure.
To save the image:
  Right click on the image -> Save Image as.
```


centroid


?

```
Visualisation of predicted secondary structure.
To save the image:
  Right click on the image -> Save Image as.
```


fq-sub


?

```
Visualisation of predicted secondary structure.
To save the image:
  Right click on the image -> Save Image as.
```

Load Sequence viewer

TurboFold: Number of sequences is less then required. n=2 (4)  
uid:86|XR\_002616897.1: Sequence cannot be extended sufficiently. Missing -146 nt upstream in the genome.  
uid:86|XR\_002616897.1: Sequence cannot be extended sufficiently by unaligned portion of query. THIS IS PROBABLY FRAGMENT! Trimmed upstream.

### Hit: XM\_019648379.1

XM\_019648379.1 PREDICTED: Hipposideros armiger phosphatidylinositol glycan anchor biosynthesis class Q (PIGQ), transcript variant X2, mRNA

```
?

```
This is BLAST alignment as read from the input file
```

Score = 46.0 bits (42.8), Expect = 5.85E+00
 Identities = 28/30 (93%), Gaps = 1/30 (3%)
 Strand = Plus/Minus
Query   48 GTAAGTGGCTGGGCCTTCCTCACACTGCCT 77  
           ||||||| ||||||||||||||| ||||||     
Sbjct 2705 GTAAGTGCCTGGGCCTTCCTCAC-CTGCCT 2677
```

Report:

|  |  |
| --- | --- |
| sequence start ?  ``` Start position of the estimated full-length sequence in genome. Start index < end index. ``` : | 2208 |
| sequence end ?  ``` End position of the estimated full-length sequence in genome. Start index < end index. ``` : | 2749 |
| bit score (CM) ?  ``` The score for aligning estimated full-length sequence to CM model   (computed by RSEARCH -> default,   infered from Rfam or provided by user) ``` : | -42.44 |
| Homology estimate ?  ``` Quick homology estimate:   Not homologous: bit score < 0   Homologous: bit score > 20 and bit score > 0.5 * query length   Uncertain otherwise ``` : | Not homologous |

Estimated full-length sequence:


?

```
Click checkbox to select multiple seuqences.
Fasta header format:
  UID|accession.versionSTRAND start-end
```

>uid:87|XM\_019648379.1rc 2208-2749
GGUGCUUCCUAAGGAGCUGGUCCCUCUGUUGGCAGGCAGCACCUGGUAAGUGCCUGGGCC
UUCCUCACCUGCCUUCAGUGAGAGCCUCUCCAGGUGUCCUCCUGGCUCUCUGGGGAAGCA
GAAGGCUGGGACACUCGCGGGCAGAGGCUAACCAGGUAUGGGCAGGGCCACUAGGAACCC
CUAGGAACCCCGAAGUUCCUGCUUGUGCAUUUCCUCGGAGCAUGUCCUCCCCAGGGGAGU
GGCUGUUCCCUGGACUCUGGGUUACAGCAGACAUGGCCUCUGAUGUUUACAGAAAGGGUC
CAUCCACCCACAACACAAGGAAGGGUGCUUGGGAGUCAGUCUCAAGGGCUGCUGAGUGCA
GCCUGGGAGGGGUCCCUCCACCUACCUCAUGGCAGGGUGGGACCACUGUGGAGCAGCCAG
GGCGACCUGCGAGCUCACCCCACUGGGAAAGGGCAUGGUGAGCAGAGGUUAAGCUCUGAA
AACCCUCCCUGGGCAGGGUCUGAGCCCGAGUGGGGGCAGGAGGGGCUCCUUGGCAGGCUU
GG

C-A-r-Rc


?

```
Visualisation of predicted secondary structure.
To save the image:
  Right click on the image -> Save Image as.
```


centroid


?

```
Visualisation of predicted secondary structure.
To save the image:
  Right click on the image -> Save Image as.
```


fq-sub


?

```
Visualisation of predicted secondary structure.
To save the image:
  Right click on the image -> Save Image as.
```

Load Sequence viewer

TurboFold: Number of sequences is less then required. n=2 (4)

### Hit: XM\_019648377.1

XM\_019648377.1 PREDICTED: Hipposideros armiger phosphatidylinositol glycan anchor biosynthesis class Q (PIGQ), transcript variant X1, mRNA

```
?

```
This is BLAST alignment as read from the input file
```

Score = 46.0 bits (42.8), Expect = 5.85E+00
 Identities = 28/30 (93%), Gaps = 1/30 (3%)
 Strand = Plus/Minus
Query   48 GTAAGTGGCTGGGCCTTCCTCACACTGCCT 77  
           ||||||| ||||||||||||||| ||||||     
Sbjct 2775 GTAAGTGCCTGGGCCTTCCTCAC-CTGCCT 2747
```

Report:

|  |  |
| --- | --- |
| sequence start ?  ``` Start position of the estimated full-length sequence in genome. Start index < end index. ``` : | 2278 |
| sequence end ?  ``` End position of the estimated full-length sequence in genome. Start index < end index. ``` : | 2819 |
| bit score (CM) ?  ``` The score for aligning estimated full-length sequence to CM model   (computed by RSEARCH -> default,   infered from Rfam or provided by user) ``` : | -42.44 |
| Homology estimate ?  ``` Quick homology estimate:   Not homologous: bit score < 0   Homologous: bit score > 20 and bit score > 0.5 * query length   Uncertain otherwise ``` : | Not homologous |

Estimated full-length sequence:


?

```
Click checkbox to select multiple seuqences.
Fasta header format:
  UID|accession.versionSTRAND start-end
```

>uid:88|XM\_019648377.1rc 2278-2819
GGUGCUUCCUAAGGAGCUGGUCCCUCUGUUGGCAGGCAGCACCUGGUAAGUGCCUGGGCC
UUCCUCACCUGCCUUCAGUGAGAGCCUCUCCAGGUGUCCUCCUGGCUCUCUGGGGAAGCA
GAAGGCUGGGACACUCGCGGGCAGAGGCUAACCAGGUAUGGGCAGGGCCACUAGGAACCC
CUAGGAACCCCGAAGUUCCUGCUUGUGCAUUUCCUCGGAGCAUGUCCUCCCCAGGGGAGU
GGCUGUUCCCUGGACUCUGGGUUACAGCAGACAUGGCCUCUGAUGUUUACAGAAAGGGUC
CAUCCACCCACAACACAAGGAAGGGUGCUUGGGAGUCAGUCUCAAGGGCUGCUGAGUGCA
GCCUGGGAGGGGUCCCUCCACCUACCUCAUGGCAGGGUGGGACCACUGUGGAGCAGCCAG
GGCGACCUGCGAGCUCACCCCACUGGGAAAGGGCAUGGUGAGCAGAGGUUAAGCUCUGAA
AACCCUCCCUGGGCAGGGUCUGAGCCCGAGUGGGGGCAGGAGGGGCUCCUUGGCAGGCUU
GG

C-A-r-Rc


?

```
Visualisation of predicted secondary structure.
To save the image:
  Right click on the image -> Save Image as.
```


centroid


?

```
Visualisation of predicted secondary structure.
To save the image:
  Right click on the image -> Save Image as.
```


fq-sub


?

```
Visualisation of predicted secondary structure.
To save the image:
  Right click on the image -> Save Image as.
```

Load Sequence viewer

TurboFold: Number of sequences is less then required. n=2 (4)

### Command, parameters and program log

executed command:

/home/big-user/PycharmProjects/foldx/rna\_blast\_analyze/BA.py --blast\_in /media/big-user/data-raid/clanek\_rbo\_analyzer/examples/tel\_rna\_sel/R7P5G0KU015-Alignment.xml --blast\_query /media/big-user/data-raid/clanek\_rbo\_analyzer/examples/tel\_rna\_sel/telo\_rna\_query.fasta --blast\_db /media/big-user/4T/blast\_db/nt --mode locarna --b\_type xml --json /media/big-user/data-raid/clanek\_rbo\_analyzer/examples/tel\_rna\_sel/rbo\_output\_sel.json --html /media/big-user/data-raid/clanek\_rbo\_analyzer/examples/tel\_rna\_sel/rbo\_output\_sel.html --prediction\_method centroid fq-sub C-A-r-Rc --threads 6 --enable\_overwrite

date:
10:12:50 09. 09. 2019

program log:

M-A-U-r-Rc: STATUS: Only one sequence remained under defined "pred\_sim\_threshold" parameter.  
Mitigation: Adding the most dissimilar homologous sequence to the non redundant sequences list.

M-A-r-Rc: STATUS: Only one sequence remained under defined "pred\_sim\_threshold" parameter.  
Mitigation: Adding the most dissimilar homologous sequence to the non redundant sequences list.

```
parameters:

    b_type: xml

    blast_db: /media/big-user/4T/blast_db/nt

    blast_in: /media/big-user/data-raid/clanek_rbo_analyzer/examples/tel_rna_sel/R7P5G0KU015-Alignment.xml

    blast_query: /media/big-user/data-raid/clanek_rbo_analyzer/examples/tel_rna_sel/telo_rna_query.fasta

    blast_regexp: [A-Z][0-9]{5}\.[0-9]+|[A-Z]{2}[0-9]{6}\.[0-9]+|[A-Z]{2}[0-9]{8}\.[0-9]+|[A-Z]{4}[0-9]{8,}\.[0-9]+|[A-Z]{6}[0-9]{9,}\.[0-9]+|AC_[0-9A-Z]+\.[0-9]+|NC_[0-9A-Z]+\.[0-9]+|NG_[0-9A-Z]+\.[0-9]+|NT_[0-9A-Z]+\.[0-9]+|NW_[0-9A-Z]+\.[0-9]+|NZ_[0-9A-Z]+\.[0-9]+|NM_[0-9A-Z]+\.[0-9]+|NR_[0-9A-Z]+\.[0-9]+|XM_[0-9A-Z]+\.[0-9]+|XR_[0-9A-Z]+\.[0-9]+|AP_[0-9A-Z]+\.[0-9]+|NP_[0-9A-Z]+\.[0-9]+|YP_[0-9A-Z]+\.[0-9]+|XP_[0-9A-Z]+\.[0-9]+|WP_[0-9A-Z]+\.[0-9]+|[A-Z]{5}[0-9]{7}\.[0-9]+|[A-Z]{3}[0-9]{5}\.[0-9]+|[A-Z]{3}[0-9]{7}\.[0-9]+|[0-9A-Z]{4}[_|][0-9A-Z]{1,2}|[A-Z]{4}[0-9]{2}S?[0-9]{6,}\.[0-9]+|[A-Z]{6}[0-9]{2}S?[0-9]{7,}\.[0-9]+|1KPD

    centroid_fast_preset: False

    cm_file: None

    command: ['/home/big-user/PycharmProjects/foldx/rna_blast_analyze/BA.py', '--blast_in', '/media/big-user/data-raid/clanek_rbo_analyzer/examples/tel_rna_sel/R7P5G0KU015-Alignment.xml', '--blast_query', '/media/big-user/data-raid/clanek_rbo_analyzer/examples/tel_rna_sel/telo_rna_query.fasta', '--blast_db', '/media/big-user/4T/blast_db/nt', '--mode', 'locarna', '--b_type', 'xml', '--json', '/media/big-user/data-raid/clanek_rbo_analyzer/examples/tel_rna_sel/rbo_output_sel.json', '--html', '/media/big-user/data-raid/clanek_rbo_analyzer/examples/tel_rna_sel/rbo_output_sel.html', '--prediction_method', 'centroid', 'fq-sub', 'C-A-r-Rc', '--threads', '6', '--enable_overwrite']

    config_file: None

    csv: None

    db_type: blastdb

    dev_pred: False

    download_rfam: False

    dump: None

    enable_overwrite: True

    filter_by_bitscore: None

    filter_by_eval: None

    html: /media/big-user/data-raid/clanek_rbo_analyzer/examples/tel_rna_sel/rbo_output_sel.html

    json: /media/big-user/data-raid/clanek_rbo_analyzer/examples/tel_rna_sel/rbo_output_sel.json

    locarna_anchor_length: 7

    locarna_params: --struct-local=0 --sequ-local=0 --free-endgaps=++++

    logfile: None

    mode: locarna

    pandas_dump: None

    pm_param_file: None

    pred_params: 
    "C-A-r-Rc": {
        "cmscore_percent": 30,
        "pred_sim_threshold": 90,
        "query_max_len_diff": 0.05
    },
    "centroid": {
        "centroid_homfold": "--engine_s CONTRAfold",
        "cmscore_percent": 10,
        "pred_sim_threshold": 70,
        "query_max_len_diff": 0.15
    },
    "fq-sub": {
        "mfold": [
            5,
            2,
            5
        ]
    }


    prediction_method: ['centroid', 'fq-sub', 'C-A-r-Rc']

    repredict_file: None

    sha1: 3ac0768c96c2ef79f1ede6cc297f26c836708031

    show_gene_browser: True

    skip_missing: False

    subseq_window_locarna: 30

    threads: 6

    turbo_fast_preset: False

    use_rfam: False

    verbose: 0

    zip_json: False
```

Select all Seqs.
Select all Structs.
Export Sequences
Export Structures
Sort Eval desc.
View all Regions
