# Supplementary material for: rboAnalyzer: A Software to Improve Characterization of Non-coding RNAs From Sequence Database Search Output
Source: Front Genet. 2020 Jul 28;11:675. doi: 10.3389/fgene.2020.00675 (PMC7401326; doi:10.3389/fgene.2020.00675)
Supplement: Supplementary file 4 [file Data_Sheet_3.ZIP › WNGTW72H014-Alignment_cnidaria_new.html]

 
 
 
     
     rboAnalyzer 
     
 
 

    
     
    

     
 
     
         Please wait while sequence viewer is loading... 
         
              
         
     
     
         
              -->
             rboAnalyzer report 
            
 
 BLAST output file:   WNGTW72H014-Alignment_cnidaria.xml
Query sequence file: u2_reg.fasta

RFAM model with best score to a query sequence     ?  Infered from query sequence by cmscan program.  
Family name: U2
E-value:     5e-25
 
 


         
         
            
    


    
     
         
            Hit: NW_018384369.1
         
         
             
                 
                     NW_018384369.1 Exaiptasia pallida isolate CC7 unplaced genomic scaffold, Aiptasia genome 1.1 scaffold1243, whole genome shotgun sequence 
                     
                         
 ?  
This is BLAST alignment as read from the input file 
                         
 Score = 172.0 bits (156.4), Expect = 4.90E-36
 Identities = 143/176 (81%), Gaps = 5/176 (3%)
 Strand = Plus/Minus
Query    1 ATCGCTTCTCGGCCTTTTGGCTAAGATCAAGTGTAGTATCTGTTCTTATCAGTTTAATATCTGATACG 68  
           |||||||||||||||||||||||||||||||||||||||||||||||||||| |||||||||||||||     
Sbjct 1696 ATCGCTTCTCGGCCTTTTGGCTAAGATCAAGTGTAGTATCTGTTCTTATCAGCTTAATATCTGATACG 1629

Query   69 -TCCTCTATCCGAG--GACAATATATTAAATGGATTTTTGGAGCAGGGAGATGGAATAGGAGCTTGCT 133 
            | ||| || ||||  |   ||||||||||  |||||||||| | |||   ||||| ||  ||||||      
Sbjct 1628 CTGCTC-AT-CGAGTAGCTCATATATTAAACTGATTTTTGGAACTGGGCTGTGGAAAAGAGGCTTGCC 1563

Query  134 CTGTCCACTCCACGCATCGACCTGGTATTGCAGTACCTCC 173 
             ||||   |||||  | | |  ||||||||| |||||||     
Sbjct 1562 TCGTCCCAGCCACGGGTTGTCTCGGTATTGCACTACCTCC 1523

 
                 
                
                 
                      Report:  
                     
                         
                             sequence start
                                   ?  
                                     
Start position of the estimated full-length sequence in genome.
Start index  
                                 :
                             
                             1508 
                         
                         
                             sequence end
                                   ?  
                                     
End position of the estimated full-length sequence in genome.
Start index  
                                 :
                             
                             1696 
                         
                         
                             bit score (CM)
                                   ?  
                                     
The score for aligning estimated full-length sequence to CM model
  (computed by RSEARCH -> default,
  infered from Rfam or provided by user) 
                                 :
                             
                             164.95 
                         
                         
                             Homology estimate
                                   ?  
                                     
Quick homology estimate:
  Not homologous: bit score   20 and bit score > 0.5 * query length
  Uncertain otherwise 
                                 :
                             
                             Homologous   
                         
                     
                    
                 
                
                
                 
                     
                          Estimated full-length sequence:  
                         
                          ? 
                             
Click checkbox to select multiple seuqences.
Fasta header format:
  UID|accession.versionSTRAND start-end 
                         
                     
                     >uid:0|NW_018384369.1rc 1508-1696
AUCGCUUCUCGGCCUUUUGGCUAAGAUCAAGUGUAGUAUCUGUUCUUAUCAGCUUAAUAU
CUGAUACGCUGCUCAUCGAGUAGCUCAUAUAUUAAACUGAUUUUUGGAACUGGGCUGUGG
AAAAGAGGCUUGCCUCGUCCCAGCCACGGGUUGUCUCGGUAUUGCACUACCUCCGAGUUC
GGCCCACUU
 
                 
                
             
            
             
                
                     
                         
                         
                             rnafold 
                             
                              ? 
                                 
Visualisation of predicted secondary structure.
To save the image:
  Right click on the image -> Save Image as. 
                             
                         
                     
                
                     
                         
                         
                             rfam-Rc 
                             
                              ? 
                                 
Visualisation of predicted secondary structure.
To save the image:
  Right click on the image -> Save Image as. 
                             
                         
                     
                
                     
                         
                         
                             TurboFold 
                             
                              ? 
                                 
Visualisation of predicted secondary structure.
To save the image:
  Right click on the image -> Save Image as. 
                             
                         
                     
                
             
            

        
             
                 Load Sequence viewer 
             
        
        
         
     
    

    
     
         
            Hit: NW_018384369.1
         
         
             
                 
                     NW_018384369.1 Exaiptasia pallida isolate CC7 unplaced genomic scaffold, Aiptasia genome 1.1 scaffold1243, whole genome shotgun sequence 
                     
                         
 ?  
This is BLAST alignment as read from the input file 
                         
 Score = 172.0 bits (156.4), Expect = 4.90E-36
 Identities = 143/176 (81%), Gaps = 5/176 (3%)
 Strand = Plus/Minus
Query    1 ATCGCTTCTCGGCCTTTTGGCTAAGATCAAGTGTAGTATCTGTTCTTATCAGTTTAATATCTGATACG 68  
           |||||||||||||||||||||||||||||||||||||||||||||||||||| |||||||||||||||     
Sbjct 3280 ATCGCTTCTCGGCCTTTTGGCTAAGATCAAGTGTAGTATCTGTTCTTATCAGCTTAATATCTGATACG 3213

Query   69 -TCCTCTATCCGAG--GACAATATATTAAATGGATTTTTGGAGCAGGGAGATGGAATAGGAGCTTGCT 133 
            | ||| || ||||  |   ||||||||||  |||||||||| | |||   ||||| ||  ||||||      
Sbjct 3212 CTGCTC-AT-CGAGTAGCTCATATATTAAACTGATTTTTGGAACTGGGCTGTGGAAAAGAGGCTTGCC 3147

Query  134 CTGTCCACTCCACGCATCGACCTGGTATTGCAGTACCTCC 173 
             ||||   |||||  | | |  ||||||||| |||||||     
Sbjct 3146 TCGTCCCAGCCACGGGTTGTCTCGGTATTGCACTACCTCC 3107

 
                 
                
                 
                      Report:  
                     
                         
                             sequence start
                                   ?  
                                     
Start position of the estimated full-length sequence in genome.
Start index  
                                 :
                             
                             3092 
                         
                         
                             sequence end
                                   ?  
                                     
End position of the estimated full-length sequence in genome.
Start index  
                                 :
                             
                             3280 
                         
                         
                             bit score (CM)
                                   ?  
                                     
The score for aligning estimated full-length sequence to CM model
  (computed by RSEARCH -> default,
  infered from Rfam or provided by user) 
                                 :
                             
                             164.95 
                         
                         
                             Homology estimate
                                   ?  
                                     
Quick homology estimate:
  Not homologous: bit score   20 and bit score > 0.5 * query length
  Uncertain otherwise 
                                 :
                             
                             Homologous   
                         
                     
                    
                 
                
                
                 
                     
                          Estimated full-length sequence:  
                         
                          ? 
                             
Click checkbox to select multiple seuqences.
Fasta header format:
  UID|accession.versionSTRAND start-end 
                         
                     
                     >uid:1|NW_018384369.1rc 3092-3280
AUCGCUUCUCGGCCUUUUGGCUAAGAUCAAGUGUAGUAUCUGUUCUUAUCAGCUUAAUAU
CUGAUACGCUGCUCAUCGAGUAGCUCAUAUAUUAAACUGAUUUUUGGAACUGGGCUGUGG
AAAAGAGGCUUGCCUCGUCCCAGCCACGGGUUGUCUCGGUAUUGCACUACCUCCGAGUUC
GGCCCACUU
 
                 
                
             
            
             
                
                     
                         
                         
                             rnafold 
                             
                              ? 
                                 
Visualisation of predicted secondary structure.
To save the image:
  Right click on the image -> Save Image as. 
                             
                         
                     
                
                     
                         
                         
                             rfam-Rc 
                             
                              ? 
                                 
Visualisation of predicted secondary structure.
To save the image:
  Right click on the image -> Save Image as. 
                             
                         
                     
                
                     
                         
                         
                             TurboFold 
                             
                              ? 
                                 
Visualisation of predicted secondary structure.
To save the image:
  Right click on the image -> Save Image as. 
                             
                         
                     
                
             
            

        
             
                 Load Sequence viewer 
             
        
        
         
     
    

    
     
         
            Hit: NW_018384369.1
         
         
             
                 
                     NW_018384369.1 Exaiptasia pallida isolate CC7 unplaced genomic scaffold, Aiptasia genome 1.1 scaffold1243, whole genome shotgun sequence 
                     
                         
 ?  
This is BLAST alignment as read from the input file 
                         
 Score = 172.0 bits (156.4), Expect = 4.90E-36
 Identities = 143/176 (81%), Gaps = 5/176 (3%)
 Strand = Plus/Minus
Query    1 ATCGCTTCTCGGCCTTTTGGCTAAGATCAAGTGTAGTATCTGTTCTTATCAGTTTAATATCTGATACG 68  
           |||||||||||||||||||||||||||||||||||||||||||||||||||| |||||||||||||||     
Sbjct 4864 ATCGCTTCTCGGCCTTTTGGCTAAGATCAAGTGTAGTATCTGTTCTTATCAGCTTAATATCTGATACG 4797

Query   69 -TCCTCTATCCGAG--GACAATATATTAAATGGATTTTTGGAGCAGGGAGATGGAATAGGAGCTTGCT 133 
            | ||| || ||||  |   ||||||||||  |||||||||| | |||   ||||| ||  ||||||      
Sbjct 4796 CTGCTC-AT-CGAGTAGCTCATATATTAAACTGATTTTTGGAACTGGGCTGTGGAAAAGAGGCTTGCC 4731

Query  134 CTGTCCACTCCACGCATCGACCTGGTATTGCAGTACCTCC 173 
             ||||   |||||  | | |  ||||||||| |||||||     
Sbjct 4730 TCGTCCCAGCCACGGGTTGTCTCGGTATTGCACTACCTCC 4691

 
                 
                
                 
                      Report:  
                     
                         
                             sequence start
                                   ?  
                                     
Start position of the estimated full-length sequence in genome.
Start index  
                                 :
                             
                             4676 
                         
                         
                             sequence end
                                   ?  
                                     
End position of the estimated full-length sequence in genome.
Start index  
                                 :
                             
                             4864 
                         
                         
                             bit score (CM)
                                   ?  
                                     
The score for aligning estimated full-length sequence to CM model
  (computed by RSEARCH -> default,
  infered from Rfam or provided by user) 
                                 :
                             
                             164.95 
                         
                         
                             Homology estimate
                                   ?  
                                     
Quick homology estimate:
  Not homologous: bit score   20 and bit score > 0.5 * query length
  Uncertain otherwise 
                                 :
                             
                             Homologous   
                         
                     
                    
                 
                
                
                 
                     
                          Estimated full-length sequence:  
                         
                          ? 
                             
Click checkbox to select multiple seuqences.
Fasta header format:
  UID|accession.versionSTRAND start-end 
                         
                     
                     >uid:2|NW_018384369.1rc 4676-4864
AUCGCUUCUCGGCCUUUUGGCUAAGAUCAAGUGUAGUAUCUGUUCUUAUCAGCUUAAUAU
CUGAUACGCUGCUCAUCGAGUAGCUCAUAUAUUAAACUGAUUUUUGGAACUGGGCUGUGG
AAAAGAGGCUUGCCUCGUCCCAGCCACGGGUUGUCUCGGUAUUGCACUACCUCCGAGUUC
GGCCCACUU
 
                 
                
             
            
             
                
                     
                         
                         
                             rnafold 
                             
                              ? 
                                 
Visualisation of predicted secondary structure.
To save the image:
  Right click on the image -> Save Image as. 
                             
                         
                     
                
                     
                         
                         
                             rfam-Rc 
                             
                              ? 
                                 
Visualisation of predicted secondary structure.
To save the image:
  Right click on the image -> Save Image as. 
                             
                         
                     
                
                     
                         
                         
                             TurboFold 
                             
                              ? 
                                 
Visualisation of predicted secondary structure.
To save the image:
  Right click on the image -> Save Image as. 
                             
                         
                     
                
             
            

        
             
                 Load Sequence viewer 
             
        
        
         
     
    

    
     
         
            Hit: NW_018384369.1
         
         
             
                 
                     NW_018384369.1 Exaiptasia pallida isolate CC7 unplaced genomic scaffold, Aiptasia genome 1.1 scaffold1243, whole genome shotgun sequence 
                     
                         
 ?  
This is BLAST alignment as read from the input file 
                         
 Score = 172.0 bits (156.4), Expect = 4.90E-36
 Identities = 143/176 (81%), Gaps = 5/176 (3%)
 Strand = Plus/Minus
Query    1 ATCGCTTCTCGGCCTTTTGGCTAAGATCAAGTGTAGTATCTGTTCTTATCAGTTTAATATCTGATACG 68  
           |||||||||||||||||||||||||||||||||||||||||||||||||||| |||||||||||||||     
Sbjct 6448 ATCGCTTCTCGGCCTTTTGGCTAAGATCAAGTGTAGTATCTGTTCTTATCAGCTTAATATCTGATACG 6381

Query   69 -TCCTCTATCCGAG--GACAATATATTAAATGGATTTTTGGAGCAGGGAGATGGAATAGGAGCTTGCT 133 
            | ||| || ||||  |   ||||||||||  |||||||||| | |||   ||||| ||  ||||||      
Sbjct 6380 CTGCTC-AT-CGAGTAGCTCATATATTAAACTGATTTTTGGAACTGGGCTGTGGAAAAGAGGCTTGCC 6315

Query  134 CTGTCCACTCCACGCATCGACCTGGTATTGCAGTACCTCC 173 
             ||||   |||||  | | |  ||||||||| |||||||     
Sbjct 6314 TCGTCCCAGCCACGGGTTGTCTCGGTATTGCACTACCTCC 6275

 
                 
                
                 
                      Report:  
                     
                         
                             sequence start
                                   ?  
                                     
Start position of the estimated full-length sequence in genome.
Start index  
                                 :
                             
                             6260 
                         
                         
                             sequence end
                                   ?  
                                     
End position of the estimated full-length sequence in genome.
Start index  
                                 :
                             
                             6448 
                         
                         
                             bit score (CM)
                                   ?  
                                     
The score for aligning estimated full-length sequence to CM model
  (computed by RSEARCH -> default,
  infered from Rfam or provided by user) 
                                 :
                             
                             164.95 
                         
                         
                             Homology estimate
                                   ?  
                                     
Quick homology estimate:
  Not homologous: bit score   20 and bit score > 0.5 * query length
  Uncertain otherwise 
                                 :
                             
                             Homologous   
                         
                     
                    
                 
                
                
                 
                     
                          Estimated full-length sequence:  
                         
                          ? 
                             
Click checkbox to select multiple seuqences.
Fasta header format:
  UID|accession.versionSTRAND start-end 
                         
                     
                     >uid:3|NW_018384369.1rc 6260-6448
AUCGCUUCUCGGCCUUUUGGCUAAGAUCAAGUGUAGUAUCUGUUCUUAUCAGCUUAAUAU
CUGAUACGCUGCUCAUCGAGUAGCUCAUAUAUUAAACUGAUUUUUGGAACUGGGCUGUGG
AAAAGAGGCUUGCCUCGUCCCAGCCACGGGUUGUCUCGGUAUUGCACUACCUCCGAGUUC
GGCCCACUU
 
                 
                
             
            
             
                
                     
                         
                         
                             rnafold 
                             
                              ? 
                                 
Visualisation of predicted secondary structure.
To save the image:
  Right click on the image -> Save Image as. 
                             
                         
                     
                
                     
                         
                         
                             rfam-Rc 
                             
                              ? 
                                 
Visualisation of predicted secondary structure.
To save the image:
  Right click on the image -> Save Image as. 
                             
                         
                     
                
                     
                         
                         
                             TurboFold 
                             
                              ? 
                                 
Visualisation of predicted secondary structure.
To save the image:
  Right click on the image -> Save Image as. 
                             
                         
                     
                
             
            

        
             
                 Load Sequence viewer 
             
        
        
         
     
    

    
     
         
            Hit: NW_018384369.1
         
         
             
                 
                     NW_018384369.1 Exaiptasia pallida isolate CC7 unplaced genomic scaffold, Aiptasia genome 1.1 scaffold1243, whole genome shotgun sequence 
                     
                         
 ?  
This is BLAST alignment as read from the input file 
                         
 Score = 172.0 bits (156.4), Expect = 4.90E-36
 Identities = 143/176 (81%), Gaps = 5/176 (3%)
 Strand = Plus/Minus
Query    1 ATCGCTTCTCGGCCTTTTGGCTAAGATCAAGTGTAGTATCTGTTCTTATCAGTTTAATATCTGATACG 68  
           |||||||||||||||||||||||||||||||||||||||||||||||||||| |||||||||||||||     
Sbjct 8032 ATCGCTTCTCGGCCTTTTGGCTAAGATCAAGTGTAGTATCTGTTCTTATCAGCTTAATATCTGATACG 7965

Query   69 -TCCTCTATCCGAG--GACAATATATTAAATGGATTTTTGGAGCAGGGAGATGGAATAGGAGCTTGCT 133 
            | ||| || ||||  |   ||||||||||  |||||||||| | |||   ||||| ||  ||||||      
Sbjct 7964 CTGCTC-AT-CGAGTAGCTCATATATTAAACTGATTTTTGGAACTGGGCTGTGGAAAAGAGGCTTGCC 7899

Query  134 CTGTCCACTCCACGCATCGACCTGGTATTGCAGTACCTCC 173 
             ||||   |||||  | | |  ||||||||| |||||||     
Sbjct 7898 TCGTCCCAGCCACGGGTTGTCTCGGTATTGCACTACCTCC 7859

 
                 
                
                 
                      Report:  
                     
                         
                             sequence start
                                   ?  
                                     
Start position of the estimated full-length sequence in genome.
Start index  
                                 :
                             
                             7844 
                         
                         
                             sequence end
                                   ?  
                                     
End position of the estimated full-length sequence in genome.
Start index  
                                 :
                             
                             8032 
                         
                         
                             bit score (CM)
                                   ?  
                                     
The score for aligning estimated full-length sequence to CM model
  (computed by RSEARCH -> default,
  infered from Rfam or provided by user) 
                                 :
                             
                             164.95 
                         
                         
                             Homology estimate
                                   ?  
                                     
Quick homology estimate:
  Not homologous: bit score   20 and bit score > 0.5 * query length
  Uncertain otherwise 
                                 :
                             
                             Homologous   
                         
                     
                    
                 
                
                
                 
                     
                          Estimated full-length sequence:  
                         
                          ? 
                             
Click checkbox to select multiple seuqences.
Fasta header format:
  UID|accession.versionSTRAND start-end 
                         
                     
                     >uid:4|NW_018384369.1rc 7844-8032
AUCGCUUCUCGGCCUUUUGGCUAAGAUCAAGUGUAGUAUCUGUUCUUAUCAGCUUAAUAU
CUGAUACGCUGCUCAUCGAGUAGCUCAUAUAUUAAACUGAUUUUUGGAACUGGGCUGUGG
AAAAGAGGCUUGCCUCGUCCCAGCCACGGGUUGUCUCGGUAUUGCACUACCUCCGAGUUC
GGCCCACUU
 
                 
                
             
            
             
                
                     
                         
                         
                             rnafold 
                             
                              ? 
                                 
Visualisation of predicted secondary structure.
To save the image:
  Right click on the image -> Save Image as. 
                             
                         
                     
                
                     
                         
                         
                             rfam-Rc 
                             
                              ? 
                                 
Visualisation of predicted secondary structure.
To save the image:
  Right click on the image -> Save Image as. 
                             
                         
                     
                
                     
                         
                         
                             TurboFold 
                             
                              ? 
                                 
Visualisation of predicted secondary structure.
To save the image:
  Right click on the image -> Save Image as. 
                             
                         
                     
                
             
            

        
             
                 Load Sequence viewer 
             
        
        
         
     
    

    
     
         
            Hit: NW_018384369.1
         
         
             
                 
                     NW_018384369.1 Exaiptasia pallida isolate CC7 unplaced genomic scaffold, Aiptasia genome 1.1 scaffold1243, whole genome shotgun sequence 
                     
                         
 ?  
This is BLAST alignment as read from the input file 
                         
 Score = 172.0 bits (156.4), Expect = 4.90E-36
 Identities = 143/176 (81%), Gaps = 5/176 (3%)
 Strand = Plus/Minus
Query    1 ATCGCTTCTCGGCCTTTTGGCTAAGATCAAGTGTAGTATCTGTTCTTATCAGTTTAATATCTGATACG 68  
           |||||||||||||||||||||||||||||||||||||||||||||||||||| |||||||||||||||     
Sbjct 9616 ATCGCTTCTCGGCCTTTTGGCTAAGATCAAGTGTAGTATCTGTTCTTATCAGCTTAATATCTGATACG 9549

Query   69 -TCCTCTATCCGAG--GACAATATATTAAATGGATTTTTGGAGCAGGGAGATGGAATAGGAGCTTGCT 133 
            | ||| || ||||  |   ||||||||||  |||||||||| | |||   ||||| ||  ||||||      
Sbjct 9548 CTGCTC-AT-CGAGTAGCTCATATATTAAACTGATTTTTGGAACTGGGCTGTGGAAAAGAGGCTTGCC 9483

Query  134 CTGTCCACTCCACGCATCGACCTGGTATTGCAGTACCTCC 173 
             ||||   |||||  | | |  ||||||||| |||||||     
Sbjct 9482 TCGTCCCAGCCACGGGTTGTCTCGGTATTGCACTACCTCC 9443

 
                 
                
                 
                      Report:  
                     
                         
                             sequence start
                                   ?  
                                     
Start position of the estimated full-length sequence in genome.
Start index  
                                 :
                             
                             9428 
                         
                         
                             sequence end
                                   ?  
                                     
End position of the estimated full-length sequence in genome.
Start index  
                                 :
                             
                             9616 
                         
                         
                             bit score (CM)
                                   ?  
                                     
The score for aligning estimated full-length sequence to CM model
  (computed by RSEARCH -> default,
  infered from Rfam or provided by user) 
                                 :
                             
                             164.95 
                         
                         
                             Homology estimate
                                   ?  
                                     
Quick homology estimate:
  Not homologous: bit score   20 and bit score > 0.5 * query length
  Uncertain otherwise 
                                 :
                             
                             Homologous   
                         
                     
                    
                 
                
                
                 
                     
                          Estimated full-length sequence:  
                         
                          ? 
                             
Click checkbox to select multiple seuqences.
Fasta header format:
  UID|accession.versionSTRAND start-end 
                         
                     
                     >uid:5|NW_018384369.1rc 9428-9616
AUCGCUUCUCGGCCUUUUGGCUAAGAUCAAGUGUAGUAUCUGUUCUUAUCAGCUUAAUAU
CUGAUACGCUGCUCAUCGAGUAGCUCAUAUAUUAAACUGAUUUUUGGAACUGGGCUGUGG
AAAAGAGGCUUGCCUCGUCCCAGCCACGGGUUGUCUCGGUAUUGCACUACCUCCGAGUUC
GGCCCACUU
 
                 
                
             
            
             
                
                     
                         
                         
                             rnafold 
                             
                              ? 
                                 
Visualisation of predicted secondary structure.
To save the image:
  Right click on the image -> Save Image as. 
                             
                         
                     
                
                     
                         
                         
                             rfam-Rc 
                             
                              ? 
                                 
Visualisation of predicted secondary structure.
To save the image:
  Right click on the image -> Save Image as. 
                             
                         
                     
                
                     
                         
                         
                             TurboFold 
                             
                              ? 
                                 
Visualisation of predicted secondary structure.
To save the image:
  Right click on the image -> Save Image as. 
                             
                         
                     
                
             
            

        
             
                 Load Sequence viewer 
             
        
        
         
     
    

    
     
         
            Hit: NW_021126809.1
         
         
             
                 
                     NW_021126809.1 Acropora millepora isolate SF001 unplaced genomic scaffold, amil_sf_1.1 amil.Sc0000438, whole genome shotgun sequence 
                     
                         
 ?  
This is BLAST alignment as read from the input file 
                         
 Score = 164.0 bits (149.2), Expect = 7.28E-34
 Identities = 138/174 (79%), Gaps = 1/174 (1%)
 Strand = Plus/Plus
Query      1 ATCGCTTCTCGGCCTTTTGGCTAAGATCAAGTGTAGTATCTGTTCTTATCAGTTTAATATCTGA 64    
             |||||||||||||||||||||||||||||||||||||||||||||||||||| |||||||||||       
Sbjct 135528 ATCGCTTCTCGGCCTTTTGGCTAAGATCAAGTGTAGTATCTGTTCTTATCAGCTTAATATCTGA 135591

Query     65 TACG-TCCTCTATCCGAGGACAATATATTAAATGGATTTTTGGAGCAGGGAGATGGAATAGGAG 127   
             |||| | |||  |  |  |   ||||||||||  |||||||||| | |||   ||||| ||  |       
Sbjct 135592 TACGCTGCTCATTGAGCAGCTCATATATTAAACTGATTTTTGGAACCGGGCTGTGGAAAAGAGG 135655

Query    128 CTTGCTCTGTCCACTCCACGCATCGACCTGGTATTGCAGTACCTCC 173   
             |||||   ||||   |||||  | | |  ||||| ||| |||||||       
Sbjct 135656 CTTGCCTCGTCCCAGCCACGGGTTGCCTCGGTATAGCACTACCTCC 135701

 
                 
                
                 
                      Report:  
                     
                         
                             sequence start
                                   ?  
                                     
Start position of the estimated full-length sequence in genome.
Start index  
                                 :
                             
                             135528 
                         
                         
                             sequence end
                                   ?  
                                     
End position of the estimated full-length sequence in genome.
Start index  
                                 :
                             
                             135716 
                         
                         
                             bit score (CM)
                                   ?  
                                     
The score for aligning estimated full-length sequence to CM model
  (computed by RSEARCH -> default,
  infered from Rfam or provided by user) 
                                 :
                             
                             161.93 
                         
                         
                             Homology estimate
                                   ?  
                                     
Quick homology estimate:
  Not homologous: bit score   20 and bit score > 0.5 * query length
  Uncertain otherwise 
                                 :
                             
                             Homologous   
                         
                     
                    
                 
                
                
                 
                     
                          Estimated full-length sequence:  
                         
                          ? 
                             
Click checkbox to select multiple seuqences.
Fasta header format:
  UID|accession.versionSTRAND start-end 
                         
                     
                     >uid:6|NW_021126809.1fw 135528-135716
AUCGCUUCUCGGCCUUUUGGCUAAGAUCAAGUGUAGUAUCUGUUCUUAUCAGCUUAAUAU
CUGAUACGCUGCUCAUUGAGCAGCUCAUAUAUUAAACUGAUUUUUGGAACCGGGCUGUGG
AAAAGAGGCUUGCCUCGUCCCAGCCACGGGUUGCCUCGGUAUAGCACUACCUCCGAGCGC
GGCCCACUU
 
                 
                
             
            
             
                
                     
                         
                         
                             rnafold 
                             
                              ? 
                                 
Visualisation of predicted secondary structure.
To save the image:
  Right click on the image -> Save Image as. 
                             
                         
                     
                
                     
                         
                         
                             rfam-Rc 
                             
                              ? 
                                 
Visualisation of predicted secondary structure.
To save the image:
  Right click on the image -> Save Image as. 
                             
                         
                     
                
                     
                         
                         
                             TurboFold 
                             
                              ? 
                                 
Visualisation of predicted secondary structure.
To save the image:
  Right click on the image -> Save Image as. 
                             
                         
                     
                
             
            

        
             
                 Load Sequence viewer 
             
        
        
         
     
    

    
     
         
            Hit: NW_021126809.1
         
         
             
                 
                     NW_021126809.1 Acropora millepora isolate SF001 unplaced genomic scaffold, amil_sf_1.1 amil.Sc0000438, whole genome shotgun sequence 
                     
                         
 ?  
This is BLAST alignment as read from the input file 
                         
 Score = 164.0 bits (149.2), Expect = 7.28E-34
 Identities = 138/174 (79%), Gaps = 1/174 (1%)
 Strand = Plus/Plus
Query      1 ATCGCTTCTCGGCCTTTTGGCTAAGATCAAGTGTAGTATCTGTTCTTATCAGTTTAATATCTGA 64    
             |||||||||||||||||||||||||||||||||||||||||||||||||||| |||||||||||       
Sbjct 177315 ATCGCTTCTCGGCCTTTTGGCTAAGATCAAGTGTAGTATCTGTTCTTATCAGCTTAATATCTGA 177378

Query     65 TACG-TCCTCTATCCGAGGACAATATATTAAATGGATTTTTGGAGCAGGGAGATGGAATAGGAG 127   
             |||| | |||  |  |  |   ||||||||||  |||||||||| | |||   ||||| ||  |       
Sbjct 177379 TACGCTGCTCATTGAGCAGCTCATATATTAAACTGATTTTTGGAACCGGGCTGTGGAAAAGAGG 177442

Query    128 CTTGCTCTGTCCACTCCACGCATCGACCTGGTATTGCAGTACCTCC 173   
             |||||   ||||   |||||  | | |  ||||| ||| |||||||       
Sbjct 177443 CTTGCCTCGTCCCAGCCACGGGTTGCCTCGGTATAGCACTACCTCC 177488

 
                 
                
                 
                      Report:  
                     
                         
                             sequence start
                                   ?  
                                     
Start position of the estimated full-length sequence in genome.
Start index  
                                 :
                             
                             177315 
                         
                         
                             sequence end
                                   ?  
                                     
End position of the estimated full-length sequence in genome.
Start index  
                                 :
                             
                             177503 
                         
                         
                             bit score (CM)
                                   ?  
                                     
The score for aligning estimated full-length sequence to CM model
  (computed by RSEARCH -> default,
  infered from Rfam or provided by user) 
                                 :
                             
                             161.93 
                         
                         
                             Homology estimate
                                   ?  
                                     
Quick homology estimate:
  Not homologous: bit score   20 and bit score > 0.5 * query length
  Uncertain otherwise 
                                 :
                             
                             Homologous   
                         
                     
                    
                 
                
                
                 
                     
                          Estimated full-length sequence:  
                         
                          ? 
                             
Click checkbox to select multiple seuqences.
Fasta header format:
  UID|accession.versionSTRAND start-end 
                         
                     
                     >uid:7|NW_021126809.1fw 177315-177503
AUCGCUUCUCGGCCUUUUGGCUAAGAUCAAGUGUAGUAUCUGUUCUUAUCAGCUUAAUAU
CUGAUACGCUGCUCAUUGAGCAGCUCAUAUAUUAAACUGAUUUUUGGAACCGGGCUGUGG
AAAAGAGGCUUGCCUCGUCCCAGCCACGGGUUGCCUCGGUAUAGCACUACCUCCGAGCGC
GGCCCACUU
 
                 
                
             
            
             
                
                     
                         
                         
                             rnafold 
                             
                              ? 
                                 
Visualisation of predicted secondary structure.
To save the image:
  Right click on the image -> Save Image as. 
                             
                         
                     
                
                     
                         
                         
                             rfam-Rc 
                             
                              ? 
                                 
Visualisation of predicted secondary structure.
To save the image:
  Right click on the image -> Save Image as. 
                             
                         
                     
                
                     
                         
                         
                             TurboFold 
                             
                              ? 
                                 
Visualisation of predicted secondary structure.
To save the image:
  Right click on the image -> Save Image as. 
                             
                         
                     
                
             
            

        
             
                 Load Sequence viewer 
             
        
        
         
     
    

    
     
         
            Hit: NW_021126809.1
         
         
             
                 
                     NW_021126809.1 Acropora millepora isolate SF001 unplaced genomic scaffold, amil_sf_1.1 amil.Sc0000438, whole genome shotgun sequence 
                     
                         
 ?  
This is BLAST alignment as read from the input file 
                         
 Score = 98.0 bits (89.7), Expect = 5.98E-16
 Identities = 49/49 (100%), Gaps = 0/49 (0%)
 Strand = Plus/Plus
Query      1 ATCGCTTCTCGGCCTTTTGGCTAAGATCAAGTGTAGTATCTGTTCTTAT 49    
             |||||||||||||||||||||||||||||||||||||||||||||||||       
Sbjct 152754 ATCGCTTCTCGGCCTTTTGGCTAAGATCAAGTGTAGTATCTGTTCTTAT 152802

 
                 
                
                 
                      Report:  
                     
                         
                             sequence start
                                   ?  
                                     
Start position of the estimated full-length sequence in genome.
Start index  
                                 :
                             
                             152754 
                         
                         
                             sequence end
                                   ?  
                                     
End position of the estimated full-length sequence in genome.
Start index  
                                 :
                             
                             152947 
                         
                         
                             bit score (CM)
                                   ?  
                                     
The score for aligning estimated full-length sequence to CM model
  (computed by RSEARCH -> default,
  infered from Rfam or provided by user) 
                                 :
                             
                             26.32 
                         
                         
                             Homology estimate
                                   ?  
                                     
Quick homology estimate:
  Not homologous: bit score   20 and bit score > 0.5 * query length
  Uncertain otherwise 
                                 :
                             
                             Uncertain  ↴  
                         
                     
                    
                     
                        Check the secondary structure and sequence viewer
                        for supporting information about possible homology.
                     
                    
                 
                
                
                 
                     
                          Estimated full-length sequence:  
                         
                          ? 
                             
Click checkbox to select multiple seuqences.
Fasta header format:
  UID|accession.versionSTRAND start-end 
                         
                     
                     >uid:8|NW_021126809.1fw 152754-152947
AUCGCUUCUCGGCCUUUUGGCUAAGAUCAAGUGUAGUAUCUGUUCUUAUGUCCGAAAGUU
CCGAGAAUUCUCCUUUUUCGCGUCGUGUAGUUCGAGUUUUUUCCCUUUUACUGUGCGUGU
GUUCAUAUUUUAUUUUUCAUAGCGAUUAAGCUGUGUUCCCUCCGCUGUCUUGGUUCGUGG
AGAAUUUUUGGUGU
 
                 
                
             
            
             
                
                     
                         
                         
                             rnafold 
                             
                              ? 
                                 
Visualisation of predicted secondary structure.
To save the image:
  Right click on the image -> Save Image as. 
                             
                         
                     
                
                     
                         
                         
                             rfam-Rc 
                             
                              ? 
                                 
Visualisation of predicted secondary structure.
To save the image:
  Right click on the image -> Save Image as. 
                             
                         
                     
                
                     
                         
                         
                             TurboFold 
                             
                              ? 
                                 
Visualisation of predicted secondary structure.
To save the image:
  Right click on the image -> Save Image as. 
                             
                         
                     
                
             
            

        
             
                 Load Sequence viewer 
             
        
        
         
     
    

    
     
         
            Hit: NW_021126809.1
         
         
             
                 
                     NW_021126809.1 Acropora millepora isolate SF001 unplaced genomic scaffold, amil_sf_1.1 amil.Sc0000438, whole genome shotgun sequence 
                     
                         
 ?  
This is BLAST alignment as read from the input file 
                         
 Score = 90.0 bits (82.4), Expect = 8.88E-14
 Identities = 101/137 (74%), Gaps = 1/137 (1%)
 Strand = Plus/Plus
Query     38 ATCTGTTCTTATCAGTTTAATATCTGATACG-TCCTCTATCCGAGGACAATATATTAAATGGAT 100   
             ||||||||||||||| ||||||||||||||| | |||  |  |  |   ||||||||||  |||       
Sbjct 184948 ATCTGTTCTTATCAGCTTAATATCTGATACGCTGCTCATTGAGCAGCTCATATATTAAACTGAT 185011

Query    101 TTTTGGAGCAGGGAGATGGAATAGGAGCTTGCTCTGTCCACTCCACGCATCGACCTGGTATTGC 164   
             ||||||| | |||   ||||| ||  ||||||   ||||   |||||  | | |  ||||| ||       
Sbjct 185012 TTTTGGAACCGGGCTGTGGAAAAGAGGCTTGCCTCGTCCCAGCCACGGGTTGCCTCGGTATAGC 185075

Query    165 AGTACCTCC 173   
             | |||||||       
Sbjct 185076 ACTACCTCC 185084

 
                 
                
                 
                      Report:  
                     
                         
                             sequence start
                                   ?  
                                     
Start position of the estimated full-length sequence in genome.
Start index  
                                 :
                             
                             184907 
                         
                         
                             sequence end
                                   ?  
                                     
End position of the estimated full-length sequence in genome.
Start index  
                                 :
                             
                             185099 
                         
                         
                             bit score (CM)
                                   ?  
                                     
The score for aligning estimated full-length sequence to CM model
  (computed by RSEARCH -> default,
  infered from Rfam or provided by user) 
                                 :
                             
                             108.54 
                         
                         
                             Homology estimate
                                   ?  
                                     
Quick homology estimate:
  Not homologous: bit score   20 and bit score > 0.5 * query length
  Uncertain otherwise 
                                 :
                             
                             Homologous   
                         
                     
                    
                 
                
                
                 
                     
                          Estimated full-length sequence:  
                         
                          ? 
                             
Click checkbox to select multiple seuqences.
Fasta header format:
  UID|accession.versionSTRAND start-end 
                         
                     
                     >uid:9|NW_021126809.1fw 184907-185099
UGUUUGUAAUUCUUGAAUAAAGUUUUUAAAAUACAAAAAAGAUCUGUUCUUAUCAGCUUA
AUAUCUGAUACGCUGCUCAUUGAGCAGCUCAUAUAUUAAACUGAUUUUUGGAACCGGGCU
GUGGAAAAGAGGCUUGCCUCGUCCCAGCCACGGGUUGCCUCGGUAUAGCACUACCUCCGA
GCGCGGCCCACUU
 
                 
                
             
            
             
                
                     
                         
                         
                             rnafold 
                             
                              ? 
                                 
Visualisation of predicted secondary structure.
To save the image:
  Right click on the image -> Save Image as. 
                             
                         
                     
                
                     
                         
                         
                             rfam-Rc 
                             
                              ? 
                                 
Visualisation of predicted secondary structure.
To save the image:
  Right click on the image -> Save Image as. 
                             
                         
                     
                
                     
                         
                         
                             TurboFold 
                             
                              ? 
                                 
Visualisation of predicted secondary structure.
To save the image:
  Right click on the image -> Save Image as. 
                             
                         
                     
                
             
            

        
             
                 Load Sequence viewer 
             
        
        
         
     
    

    
     
         
            Hit: NW_021126809.1
         
         
             
                 
                     NW_021126809.1 Acropora millepora isolate SF001 unplaced genomic scaffold, amil_sf_1.1 amil.Sc0000438, whole genome shotgun sequence 
                     
                         
 ?  
This is BLAST alignment as read from the input file 
                         
 Score = 85.0 bits (77.9), Expect = 3.77E-12
 Identities = 109/152 (72%), Gaps = 1/152 (1%)
 Strand = Plus/Plus
Query   38 ATCTGTTCTTATCAGTTTAATATCTGATA-CGTCCTCTATCCGAGGACAATATATTAAATGGATTTTT 104 
           ||||||||||||||| ||||||||||||| | | |||  |  |  |   ||||||||||  |||||||     
Sbjct 2764 ATCTGTTCTTATCAGCTTAATATCTGATACCCTGCTCATTGAGCAGCTCATATATTAAACTGATTTTT 2831

Query  105 GGAGCAGGGAGATGGAATAGGAGCTTGCTCTGTCCACTCCACGCATCGACCTGGTATTGCAGTACCTC 172 
           ||| | |||   ||||| ||  ||||||   ||||   |||||  | | |  ||||| | | ||||||     
Sbjct 2832 GGAACCGGGCTGTGGAAAAGAGGCTTGCCTCGTCCCAGCCACGGGTTGCCTCGGTATAGGACTACCTC 2899

Query  173 CAGGACCGGTGCACTT 188 
           || |  |||  |||||     
Sbjct 2900 CAAGCACGGCCCACTT 2915

 
                 
                
                 
                      Report:  
                     
                         
                             sequence start
                                   ?  
                                     
Start position of the estimated full-length sequence in genome.
Start index  
                                 :
                             
                             2727 
                         
                         
                             sequence end
                                   ?  
                                     
End position of the estimated full-length sequence in genome.
Start index  
                                 :
                             
                             2915 
                         
                         
                             bit score (CM)
                                   ?  
                                     
The score for aligning estimated full-length sequence to CM model
  (computed by RSEARCH -> default,
  infered from Rfam or provided by user) 
                                 :
                             
                             88.83 
                         
                         
                             Homology estimate
                                   ?  
                                     
Quick homology estimate:
  Not homologous: bit score   20 and bit score > 0.5 * query length
  Uncertain otherwise 
                                 :
                             
                             Uncertain  ↴  
                         
                     
                    
                     
                        Check the secondary structure and sequence viewer
                        for supporting information about possible homology.
                     
                    
                 
                
                
                 
                     
                          Estimated full-length sequence:  
                         
                          ? 
                             
Click checkbox to select multiple seuqences.
Fasta header format:
  UID|accession.versionSTRAND start-end 
                         
                     
                     >uid:10|NW_021126809.1fw 2727-2915
UAUUGUAAAUAAACGGUUUUUUCAAAACAAAAAAAAAAUCUGUUCUUAUCAGCUUAAUAU
CUGAUACCCUGCUCAUUGAGCAGCUCAUAUAUUAAACUGAUUUUUGGAACCGGGCUGUGG
AAAAGAGGCUUGCCUCGUCCCAGCCACGGGUUGCCUCGGUAUAGGACUACCUCCAAGCAC
GGCCCACUU
 
                 
                
             
            
             
                
                     
                         
                         
                             rnafold 
                             
                              ? 
                                 
Visualisation of predicted secondary structure.
To save the image:
  Right click on the image -> Save Image as. 
                             
                         
                     
                
                     
                         
                         
                             rfam-Rc 
                             
                              ? 
                                 
Visualisation of predicted secondary structure.
To save the image:
  Right click on the image -> Save Image as. 
                             
                         
                     
                
                     
                         
                         
                             TurboFold 
                             
                              ? 
                                 
Visualisation of predicted secondary structure.
To save the image:
  Right click on the image -> Save Image as. 
                             
                         
                     
                
             
            

        
             
                 Load Sequence viewer 
             
        
        
         
     
    

    
     
         
            Hit: NW_021126809.1
         
         
             
                 
                     NW_021126809.1 Acropora millepora isolate SF001 unplaced genomic scaffold, amil_sf_1.1 amil.Sc0000438, whole genome shotgun sequence 
                     
                         
 ?  
This is BLAST alignment as read from the input file 
                         
 Score = 80.0 bits (73.4), Expect = 4.60E-11
 Identities = 99/137 (72%), Gaps = 1/137 (1%)
 Strand = Plus/Plus
Query    38 ATCTGTTCTTATCAGTTTAATATCTGATACG-TCCTCTATCCGAGGACAATATATTAAATGGATTT 102  
            ||||||||||||||| | ||||||||||| | | |||  |  |  |   ||||||||||  |||||      
Sbjct 11191 ATCTGTTCTTATCAGCTCAATATCTGATATGCTGCTCATTGAGCAGCTCATATATTAAACTGATTT 11256

Query   103 TTGGAGCAGGGAGATGGAATAGGAGCTTGCTCTGTCCACTCCACGCATCGACCTGGTATTGCAGTA 168  
            ||||| | |||   ||||| ||  ||||||  |||||   |||||  | | |   |||| ||| ||      
Sbjct 11257 TTGGAACCGGGCTGTGGAAAAGAGGCTTGCCTTGTCCCAGCCACGGGTTGCCTCAGTATAGCACTA 11322

Query   169 CCTCC 173  
            |||||      
Sbjct 11323 CCTCC 11327

 
                 
                
                 
                      Report:  
                     
                         
                             sequence start
                                   ?  
                                     
Start position of the estimated full-length sequence in genome.
Start index  
                                 :
                             
                             11154 
                         
                         
                             sequence end
                                   ?  
                                     
End position of the estimated full-length sequence in genome.
Start index  
                                 :
                             
                             11342 
                         
                         
                             bit score (CM)
                                   ?  
                                     
The score for aligning estimated full-length sequence to CM model
  (computed by RSEARCH -> default,
  infered from Rfam or provided by user) 
                                 :
                             
                             86.69 
                         
                         
                             Homology estimate
                                   ?  
                                     
Quick homology estimate:
  Not homologous: bit score   20 and bit score > 0.5 * query length
  Uncertain otherwise 
                                 :
                             
                             Uncertain  ↴  
                         
                     
                    
                     
                        Check the secondary structure and sequence viewer
                        for supporting information about possible homology.
                     
                    
                 
                
                
                 
                     
                          Estimated full-length sequence:  
                         
                          ? 
                             
Click checkbox to select multiple seuqences.
Fasta header format:
  UID|accession.versionSTRAND start-end 
                         
                     
                     >uid:11|NW_021126809.1fw 11154-11342
UAAUUAUCAAUGAAUAAAGUUUCAAAAUACAAAAAAAAUCUGUUCUUAUCAGCUCAAUAU
CUGAUAUGCUGCUCAUUGAGCAGCUCAUAUAUUAAACUGAUUUUUGGAACCGGGCUGUGG
AAAAGAGGCUUGCCUUGUCCCAGCCACGGGUUGCCUCAGUAUAGCACUACCUCCGAGCGC
GGCCCACUU
 
                 
                
             
            
             
                
                     
                         
                         
                             rnafold 
                             
                              ? 
                                 
Visualisation of predicted secondary structure.
To save the image:
  Right click on the image -> Save Image as. 
                             
                         
                     
                
                     
                         
                         
                             rfam-Rc 
                             
                              ? 
                                 
Visualisation of predicted secondary structure.
To save the image:
  Right click on the image -> Save Image as. 
                             
                         
                     
                
                     
                         
                         
                             TurboFold 
                             
                              ? 
                                 
Visualisation of predicted secondary structure.
To save the image:
  Right click on the image -> Save Image as. 
                             
                         
                     
                
             
            

        
             
                 Load Sequence viewer 
             
        
        
         
     
    

    
     
         
            Hit: NW_021126809.1
         
         
             
                 
                     NW_021126809.1 Acropora millepora isolate SF001 unplaced genomic scaffold, amil_sf_1.1 amil.Sc0000438, whole genome shotgun sequence 
                     
                         
 ?  
This is BLAST alignment as read from the input file 
                         
 Score = 69.0 bits (63.5), Expect = 8.31E-08
 Identities = 36/37 (97%), Gaps = 0/37 (0%)
 Strand = Plus/Plus
Query      1 ATCGCTTCTCGGCCTTTTGGCTAAGATCAAGTGTAGT 37    
             ||||||||||||||||||| |||||||||||||||||       
Sbjct 116348 ATCGCTTCTCGGCCTTTTGACTAAGATCAAGTGTAGT 116384

 
                 
                
                 
                      Report:  
                     
                         
                             sequence start
                                   ?  
                                     
Start position of the estimated full-length sequence in genome.
Start index  
                                 :
                             
                             116348 
                         
                         
                             sequence end
                                   ?  
                                     
End position of the estimated full-length sequence in genome.
Start index  
                                 :
                             
                             116533 
                         
                         
                             bit score (CM)
                                   ?  
                                     
The score for aligning estimated full-length sequence to CM model
  (computed by RSEARCH -> default,
  infered from Rfam or provided by user) 
                                 :
                             
                             19.0 
                         
                         
                             Homology estimate
                                   ?  
                                     
Quick homology estimate:
  Not homologous: bit score   20 and bit score > 0.5 * query length
  Uncertain otherwise 
                                 :
                             
                             Uncertain  ↴  
                         
                     
                    
                     
                        Check the secondary structure and sequence viewer
                        for supporting information about possible homology.
                     
                    
                 
                
                
                 
                     
                          Estimated full-length sequence:  
                         
                          ? 
                             
Click checkbox to select multiple seuqences.
Fasta header format:
  UID|accession.versionSTRAND start-end 
                         
                     
                     >uid:12|NW_021126809.1fw 116348-116533
AUCGCUUCUCGGCCUUUUGACUAAGAUCAAGUGUAGUUCUUGCAUCUUACCAUGAGCAUU
UUUUUACCGUCAAUUUUCUUUACCGUCCUGUGCUAAGUACCCGCCGUGGGGGAACGCCAA
GGACAGCCCCUUCCCCGUGAGAAGUGAUUGUUUGCUAGUCCCAGUCCCAGCCACGAUGGC
UCACGC
 
                 
                
             
            
             
                
                     
                         
                         
                             rnafold 
                             
                              ? 
                                 
Visualisation of predicted secondary structure.
To save the image:
  Right click on the image -> Save Image as. 
                             
                         
                     
                
                     
                         
                         
                             rfam-Rc 
                             
                              ? 
                                 
Visualisation of predicted secondary structure.
To save the image:
  Right click on the image -> Save Image as. 
                             
                         
                     
                
                     
                         
                         
                             TurboFold 
                             
                              ? 
                                 
Visualisation of predicted secondary structure.
To save the image:
  Right click on the image -> Save Image as. 
                             
                         
                     
                
             
            

        
             
                 Load Sequence viewer 
             
        
        
         
     
    

    
     
         
            Hit: NW_021126809.1
         
         
             
                 
                     NW_021126809.1 Acropora millepora isolate SF001 unplaced genomic scaffold, amil_sf_1.1 amil.Sc0000438, whole genome shotgun sequence 
                     
                         
 ?  
This is BLAST alignment as read from the input file 
                         
 Score = 63.0 bits (58.1), Expect = 3.53E-06
 Identities = 56/71 (79%), Gaps = 1/71 (1%)
 Strand = Plus/Plus
Query    38 ATCTGTTCTTATCAGTTTAATATCTGATACG-TCCTCTATCCGAGGACAATATATTAAATGGATTT 102  
            ||||||||||||||| ||||||||||||||| | |||  |  |  |   ||| ||||||  |||||      
Sbjct 77199 ATCTGTTCTTATCAGCTTAATATCTGATACGCTGCTCATTGAGCAGCTCATACATTAAACTGATTT 77264

Query   103 TTGGA 107  
            |||||      
Sbjct 77265 TTGGA 77269

 
                 
                
                 
                      Report:  
                     
                         
                             sequence start
                                   ?  
                                     
Start position of the estimated full-length sequence in genome.
Start index  
                                 :
                             
                             77164 
                         
                         
                             sequence end
                                   ?  
                                     
End position of the estimated full-length sequence in genome.
Start index  
                                 :
                             
                             77348 
                         
                         
                             bit score (CM)
                                   ?  
                                     
The score for aligning estimated full-length sequence to CM model
  (computed by RSEARCH -> default,
  infered from Rfam or provided by user) 
                                 :
                             
                             24.42 
                         
                         
                             Homology estimate
                                   ?  
                                     
Quick homology estimate:
  Not homologous: bit score   20 and bit score > 0.5 * query length
  Uncertain otherwise 
                                 :
                             
                             Uncertain  ↴  
                         
                     
                    
                     
                        Check the secondary structure and sequence viewer
                        for supporting information about possible homology.
                     
                    
                 
                
                
                 
                     
                          Estimated full-length sequence:  
                         
                          ? 
                             
Click checkbox to select multiple seuqences.
Fasta header format:
  UID|accession.versionSTRAND start-end 
                         
                     
                     >uid:13|NW_021126809.1fw 77164-77348
AUUGUAAAUGAACGGUUUUUUCACAAAAAAAAAAAAUCUGUUCUUAUCAGCUUAAUAUCU
GAUACGCUGCUCAUUGAGCAGCUCAUACAUUAAACUGAUUUUUGGAACCAGGCUGUGGAA
AAGAGGNNCCUCCGUAUAGCACUACCAAGCUCCGUAAUGCGGUGGCAGGCUGCGUCAGAA
AAGUU
 
                 
                
             
            
             
                
                     
                         
                         
                             rnafold 
                             
                              ? 
                                 
Visualisation of predicted secondary structure.
To save the image:
  Right click on the image -> Save Image as. 
                             
                         
                     
                
                     
                         
                         
                             rfam-Rc 
                             
                              ? 
                                 
Visualisation of predicted secondary structure.
To save the image:
  Right click on the image -> Save Image as. 
                             
                         
                     
                
             
            

        
             
                 Load Sequence viewer 
             
        
        
             
                 
                
                   Ambiguous base detected in uid:13|NW_021126809.1fw, violating base NN, pos 126 
                
                 
             
        
         
     
    

    
     
         
            Hit: NW_021126809.1
         
         
             
                 
                     NW_021126809.1 Acropora millepora isolate SF001 unplaced genomic scaffold, amil_sf_1.1 amil.Sc0000438, whole genome shotgun sequence 
                     
                         
 ?  
This is BLAST alignment as read from the input file 
                         
 Score = 58.0 bits (53.6), Expect = 4.31E-05
 Identities = 32/34 (94%), Gaps = 0/34 (0%)
 Strand = Plus/Plus
Query      1 ATCGCTTCTCGGCCTTTTGGCTAAGATCAAGTGT 34    
             ||||||||||||||||||||||||||||  ||||       
Sbjct 137356 ATCGCTTCTCGGCCTTTTGGCTAAGATCTTGTGT 137389

 
                 
                
                 
                      Report:  
                     
                         
                             sequence start
                                   ?  
                                     
Start position of the estimated full-length sequence in genome.
Start index  
                                 :
                             
                             137356 
                         
                         
                             sequence end
                                   ?  
                                     
End position of the estimated full-length sequence in genome.
Start index  
                                 :
                             
                             137525 
                         
                         
                             bit score (CM)
                                   ?  
                                     
The score for aligning estimated full-length sequence to CM model
  (computed by RSEARCH -> default,
  infered from Rfam or provided by user) 
                                 :
                             
                             16.96 
                         
                         
                             Homology estimate
                                   ?  
                                     
Quick homology estimate:
  Not homologous: bit score   20 and bit score > 0.5 * query length
  Uncertain otherwise 
                                 :
                             
                             Uncertain  ↴  
                         
                     
                    
                     
                        Check the secondary structure and sequence viewer
                        for supporting information about possible homology.
                     
                    
                 
                
                
                 
                     
                          Estimated full-length sequence:  
                         
                          ? 
                             
Click checkbox to select multiple seuqences.
Fasta header format:
  UID|accession.versionSTRAND start-end 
                         
                     
                     >uid:14|NW_021126809.1fw 137356-137525
AUCGCUUCUCGGCCUUUUGGCUAAGAUCUUGUGUGACUUGAAUCUUGCCAAUAGGAUUGU
GUGACUUUCCUUUUUACGUCCUGUGCUAAGUACCCGCCGGGGGGGAACGCCAUGGACCCU
CCCCGUGAGAGUAGCGAUUCCACGCAUGCAAUCGAGGCAAAUGCUGACGU
 
                 
                
             
            
             
                
                     
                         
                         
                             rnafold 
                             
                              ? 
                                 
Visualisation of predicted secondary structure.
To save the image:
  Right click on the image -> Save Image as. 
                             
                         
                     
                
                     
                         
                         
                             rfam-Rc 
                             
                              ? 
                                 
Visualisation of predicted secondary structure.
To save the image:
  Right click on the image -> Save Image as. 
                             
                         
                     
                
                     
                         
                         
                             TurboFold 
                             
                              ? 
                                 
Visualisation of predicted secondary structure.
To save the image:
  Right click on the image -> Save Image as. 
                             
                         
                     
                
             
            

        
             
                 Load Sequence viewer 
             
        
        
         
     
    

    
     
         
            Hit: NW_021126809.1
         
         
             
                 
                     NW_021126809.1 Acropora millepora isolate SF001 unplaced genomic scaffold, amil_sf_1.1 amil.Sc0000438, whole genome shotgun sequence 
                     
                         
 ?  
This is BLAST alignment as read from the input file 
                         
 Score = 56.0 bits (51.8), Expect = 1.50E-04
 Identities = 28/28 (100%), Gaps = 0/28 (0%)
 Strand = Plus/Plus
Query      1 ATCGCTTCTCGGCCTTTTGGCTAAGATC 28    
             ||||||||||||||||||||||||||||       
Sbjct 138904 ATCGCTTCTCGGCCTTTTGGCTAAGATC 138931

 
                 
                
                 
                      Report:  
                     
                         
                             sequence start
                                   ?  
                                     
Start position of the estimated full-length sequence in genome.
Start index  
                                 :
                             
                             138904 
                         
                         
                             sequence end
                                   ?  
                                     
End position of the estimated full-length sequence in genome.
Start index  
                                 :
                             
                             139090 
                         
                         
                             bit score (CM)
                                   ?  
                                     
The score for aligning estimated full-length sequence to CM model
  (computed by RSEARCH -> default,
  infered from Rfam or provided by user) 
                                 :
                             
                             18.92 
                         
                         
                             Homology estimate
                                   ?  
                                     
Quick homology estimate:
  Not homologous: bit score   20 and bit score > 0.5 * query length
  Uncertain otherwise 
                                 :
                             
                             Uncertain  ↴  
                         
                     
                    
                     
                        Check the secondary structure and sequence viewer
                        for supporting information about possible homology.
                     
                    
                 
                
                
                 
                     
                          Estimated full-length sequence:  
                         
                          ? 
                             
Click checkbox to select multiple seuqences.
Fasta header format:
  UID|accession.versionSTRAND start-end 
                         
                     
                     >uid:15|NW_021126809.1fw 138904-139090
AUCGCUUCUCGGCCUUUUGGCUAAGAUCUUGUGAAUCUUUUGCCAAUAGGAUUGUGACUU
UUCCUUUUACGUCCUGUGCUAAGUACCCGCCGGGGGGGAACGCCAUGGACCCUCCCCGUG
AGAGUAGCGAUUCCACGCCUGCAAUCGAGGCAAAUGCUGACGUCGGGGUGACGUCAGUGC
CUCAUGG
 
                 
                
             
            
             
                
                     
                         
                         
                             rnafold 
                             
                              ? 
                                 
Visualisation of predicted secondary structure.
To save the image:
  Right click on the image -> Save Image as. 
                             
                         
                     
                
                     
                         
                         
                             rfam-Rc 
                             
                              ? 
                                 
Visualisation of predicted secondary structure.
To save the image:
  Right click on the image -> Save Image as. 
                             
                         
                     
                
                     
                         
                         
                             TurboFold 
                             
                              ? 
                                 
Visualisation of predicted secondary structure.
To save the image:
  Right click on the image -> Save Image as. 
                             
                         
                     
                
             
            

        
             
                 Load Sequence viewer 
             
        
        
         
     
    

    
     
         
            Hit: NW_021126809.1
         
         
             
                 
                     NW_021126809.1 Acropora millepora isolate SF001 unplaced genomic scaffold, amil_sf_1.1 amil.Sc0000438, whole genome shotgun sequence 
                     
                         
 ?  
This is BLAST alignment as read from the input file 
                         
 Score = 51.0 bits (47.3), Expect = 6.39E-03
 Identities = 27/28 (96%), Gaps = 0/28 (0%)
 Strand = Plus/Plus
Query     1 ATCGCTTCTCGGCCTTTTGGCTAAGATC 28   
            ||||||||||||||||||||||||| ||      
Sbjct 87874 ATCGCTTCTCGGCCTTTTGGCTAAGTTC 87901

 
                 
                
                 
                      Report:  
                     
                         
                             sequence start
                                   ?  
                                     
Start position of the estimated full-length sequence in genome.
Start index  
                                 :
                             
                             87874 
                         
                         
                             sequence end
                                   ?  
                                     
End position of the estimated full-length sequence in genome.
Start index  
                                 :
                             
                             88067 
                         
                         
                             bit score (CM)
                                   ?  
                                     
The score for aligning estimated full-length sequence to CM model
  (computed by RSEARCH -> default,
  infered from Rfam or provided by user) 
                                 :
                             
                             -1.17 
                         
                         
                             Homology estimate
                                   ?  
                                     
Quick homology estimate:
  Not homologous: bit score   20 and bit score > 0.5 * query length
  Uncertain otherwise 
                                 :
                             
                             Not homologous   
                         
                     
                    
                 
                
                
                 
                     
                          Estimated full-length sequence:  
                         
                          ? 
                             
Click checkbox to select multiple seuqences.
Fasta header format:
  UID|accession.versionSTRAND start-end 
                         
                     
                     >uid:16|NW_021126809.1fw 87874-88067
AUCGCUUCUCGGCCUUUUGGCUAAGUUCCAAAAGGUUCGCUUUGCUAUCCUUGUUAUCUC
CAUAGAUUAUCAUGGCAAGUGCAGCUAUGGAGAUGACUGAAGACCUUCAAGUUCAAGAUG
CACAGGAAAAACAAAUUUGGAGUGAAGAAGUGGAACAACAAGUUCAGGGAAAUGCUAAUG
AUGGUGAACCACAG
 
                 
                
             
            
             
                
                     
                         
                         
                             rnafold 
                             
                              ? 
                                 
Visualisation of predicted secondary structure.
To save the image:
  Right click on the image -> Save Image as. 
                             
                         
                     
                
                     
                         
                         
                             rfam-Rc 
                             
                              ? 
                                 
Visualisation of predicted secondary structure.
To save the image:
  Right click on the image -> Save Image as. 
                             
                         
                     
                
                     
                         
                         
                             TurboFold 
                             
                              ? 
                                 
Visualisation of predicted secondary structure.
To save the image:
  Right click on the image -> Save Image as. 
                             
                         
                     
                
             
            

        
             
                 Load Sequence viewer 
             
        
        
         
     
    

    
     
         
            Hit: NW_021126809.1
         
         
             
                 
                     NW_021126809.1 Acropora millepora isolate SF001 unplaced genomic scaffold, amil_sf_1.1 amil.Sc0000438, whole genome shotgun sequence 
                     
                         
 ?  
This is BLAST alignment as read from the input file 
                         
 Score = 50.0 bits (46.4), Expect = 6.39E-03
 Identities = 25/25 (100%), Gaps = 0/25 (0%)
 Strand = Plus/Plus
Query     1 ATCGCTTCTCGGCCTTTTGGCTAAG 25   
            |||||||||||||||||||||||||      
Sbjct 78760 ATCGCTTCTCGGCCTTTTGGCTAAG 78784

 
                 
                
                 
                      Report:  
                     
                         
                             sequence start
                                   ?  
                                     
Start position of the estimated full-length sequence in genome.
Start index  
                                 :
                             
                             78760 
                         
                         
                             sequence end
                                   ?  
                                     
End position of the estimated full-length sequence in genome.
Start index  
                                 :
                             
                             78969 
                         
                         
                             bit score (CM)
                                   ?  
                                     
The score for aligning estimated full-length sequence to CM model
  (computed by RSEARCH -> default,
  infered from Rfam or provided by user) 
                                 :
                             
                             -4.8100000000000005 
                         
                         
                             Homology estimate
                                   ?  
                                     
Quick homology estimate:
  Not homologous: bit score   20 and bit score > 0.5 * query length
  Uncertain otherwise 
                                 :
                             
                             Not homologous   
                         
                     
                    
                 
                
                
                 
                     
                          Estimated full-length sequence:  
                         
                          ? 
                             
Click checkbox to select multiple seuqences.
Fasta header format:
  UID|accession.versionSTRAND start-end 
                         
                     
                     >uid:17|NW_021126809.1fw 78760-78969
AUCGCUUCUCGGCCUUUUGGCUAAGUCUUUUUCGAUUGGCUUUUGUUAUUUUUGGAGACC
GUCCUAAGUACCCGCCGGUGGGGAACGGCAGAGCCUGCUUCACCAAUUCAUCUUCAUUUU
AAGAAGCGAUUUCUCAAAAACUCUUUGUGGCGUUUUUUGAGACGGUCUCUACUUUGAGUC
AUGUCUGACUCCCACAAUGAUCAGGGAUUG
 
                 
                
             
            
             
                
                     
                         
                         
                             rnafold 
                             
                              ? 
                                 
Visualisation of predicted secondary structure.
To save the image:
  Right click on the image -> Save Image as. 
                             
                         
                     
                
                     
                         
                         
                             rfam-Rc 
                             
                              ? 
                                 
Visualisation of predicted secondary structure.
To save the image:
  Right click on the image -> Save Image as. 
                             
                         
                     
                
                     
                         
                         
                             TurboFold 
                             
                              ? 
                                 
Visualisation of predicted secondary structure.
To save the image:
  Right click on the image -> Save Image as. 
                             
                         
                     
                
             
            

        
             
                 Load Sequence viewer 
             
        
        
         
     
    

    
     
         
            Hit: NW_021126809.1
         
         
             
                 
                     NW_021126809.1 Acropora millepora isolate SF001 unplaced genomic scaffold, amil_sf_1.1 amil.Sc0000438, whole genome shotgun sequence 
                     
                         
 ?  
This is BLAST alignment as read from the input file 
                         
 Score = 46.0 bits (42.8), Expect = 7.79E-02
 Identities = 26/28 (93%), Gaps = 0/28 (0%)
 Strand = Plus/Plus
Query     1 ATCGCTTCTCGGCCTTTTGGCTAAGATC 28   
            ||||||||||||||||||||| ||| ||      
Sbjct 23874 ATCGCTTCTCGGCCTTTTGGCAAAGCTC 23901

 
                 
                
                 
                      Report:  
                     
                         
                             sequence start
                                   ?  
                                     
Start position of the estimated full-length sequence in genome.
Start index  
                                 :
                             
                             23874 
                         
                         
                             sequence end
                                   ?  
                                     
End position of the estimated full-length sequence in genome.
Start index  
                                 :
                             
                             24052 
                         
                         
                             bit score (CM)
                                   ?  
                                     
The score for aligning estimated full-length sequence to CM model
  (computed by RSEARCH -> default,
  infered from Rfam or provided by user) 
                                 :
                             
                             -13.52 
                         
                         
                             Homology estimate
                                   ?  
                                     
Quick homology estimate:
  Not homologous: bit score   20 and bit score > 0.5 * query length
  Uncertain otherwise 
                                 :
                             
                             Not homologous   
                         
                     
                    
                 
                
                
                 
                     
                          Estimated full-length sequence:  
                         
                          ? 
                             
Click checkbox to select multiple seuqences.
Fasta header format:
  UID|accession.versionSTRAND start-end 
                         
                     
                     >uid:18|NW_021126809.1fw 23874-24052
AUCGCUUCUCGGCCUUUUGGCAAAGCUCUUUCAGCCAGACACUUGUGGGAUAACCCAGGG
CGAAGCUCUAUCGGUGUGCUCUCAGAAGUUGUUUCGCUCUUCGCCUCUUCGGAGGUUUCA
UCCAAAUGGACAACGGUGAUGUUUUAGAUCCCCGUUCGCGGGCGAAUGUUAAUCAAGUU
 
                 
                
             
            
             
                
                     
                         
                         
                             rnafold 
                             
                              ? 
                                 
Visualisation of predicted secondary structure.
To save the image:
  Right click on the image -> Save Image as. 
                             
                         
                     
                
                     
                         
                         
                             rfam-Rc 
                             
                              ? 
                                 
Visualisation of predicted secondary structure.
To save the image:
  Right click on the image -> Save Image as. 
                             
                         
                     
                
                     
                         
                         
                             TurboFold 
                             
                              ? 
                                 
Visualisation of predicted secondary structure.
To save the image:
  Right click on the image -> Save Image as. 
                             
                         
                     
                
             
            

        
             
                 Load Sequence viewer 
             
        
        
         
     
    

    
     
         
            Hit: NW_021126809.1
         
         
             
                 
                     NW_021126809.1 Acropora millepora isolate SF001 unplaced genomic scaffold, amil_sf_1.1 amil.Sc0000438, whole genome shotgun sequence 
                     
                         
 ?  
This is BLAST alignment as read from the input file 
                         
 Score = 40.0 bits (37.4), Expect = 3.31E+00
 Identities = 23/25 (92%), Gaps = 0/25 (0%)
 Strand = Plus/Plus
Query     1 ATCGCTTCTCGGCCTTTTGGCTAAG 25   
            || ||||||||||||||| ||||||      
Sbjct 55486 ATTGCTTCTCGGCCTTTTCGCTAAG 55510

 
                 
                
                 
                      Report:  
                     
                         
                             sequence start
                                   ?  
                                     
Start position of the estimated full-length sequence in genome.
Start index  
                                 :
                             
                             55486 
                         
                         
                             sequence end
                                   ?  
                                     
End position of the estimated full-length sequence in genome.
Start index  
                                 :
                             
                             55683 
                         
                         
                             bit score (CM)
                                   ?  
                                     
The score for aligning estimated full-length sequence to CM model
  (computed by RSEARCH -> default,
  infered from Rfam or provided by user) 
                                 :
                             
                             -10.01 
                         
                         
                             Homology estimate
                                   ?  
                                     
Quick homology estimate:
  Not homologous: bit score   20 and bit score > 0.5 * query length
  Uncertain otherwise 
                                 :
                             
                             Not homologous   
                         
                     
                    
                 
                
                
                 
                     
                          Estimated full-length sequence:  
                         
                          ? 
                             
Click checkbox to select multiple seuqences.
Fasta header format:
  UID|accession.versionSTRAND start-end 
                         
                     
                     >uid:19|NW_021126809.1fw 55486-55683
AUUGCUUCUCGGCCUUUUCGCUAAGCCAUUUUGCUGCGUUGACUUGUGGGGUCCCCCAGG
GCGAAGGCCUAUCAGUCAGUGGAGAGAAGUUUUUUCGCCCUUUACCUCUUUGGAGGUAUC
UUCUAUGGAUAACGAUAUUGGUAAUGGUUCCCAGAAUUUGGGUGUUUCUUGCGAAAAUGA
UGUGAUGCCUGAAGUUCC
 
                 
                
             
            
             
                
                     
                         
                         
                             rnafold 
                             
                              ? 
                                 
Visualisation of predicted secondary structure.
To save the image:
  Right click on the image -> Save Image as. 
                             
                         
                     
                
                     
                         
                         
                             rfam-Rc 
                             
                              ? 
                                 
Visualisation of predicted secondary structure.
To save the image:
  Right click on the image -> Save Image as. 
                             
                         
                     
                
                     
                         
                         
                             TurboFold 
                             
                              ? 
                                 
Visualisation of predicted secondary structure.
To save the image:
  Right click on the image -> Save Image as. 
                             
                         
                     
                
             
            

        
             
                 Load Sequence viewer 
             
        
        
         
     
    

    
     
         
            Hit: NW_021126939.1
         
         
             
                 
                     NW_021126939.1 Acropora millepora isolate SF001 unplaced genomic scaffold, amil_sf_1.1 amil.Sc0000568, whole genome shotgun sequence 
                     
                         
 ?  
This is BLAST alignment as read from the input file 
                         
 Score = 164.0 bits (149.2), Expect = 7.28E-34
 Identities = 138/174 (79%), Gaps = 1/174 (1%)
 Strand = Plus/Plus
Query     1 ATCGCTTCTCGGCCTTTTGGCTAAGATCAAGTGTAGTATCTGTTCTTATCAGTTTAATATCTGATA 66   
            |||||||||||||||||||||||||||||||||||||||||||||||||||| |||||||||||||      
Sbjct 60715 ATCGCTTCTCGGCCTTTTGGCTAAGATCAAGTGTAGTATCTGTTCTTATCAGCTTAATATCTGATA 60780

Query    67 CG-TCCTCTATCCGAGGACAATATATTAAATGGATTTTTGGAGCAGGGAGATGGAATAGGAGCTTG 131  
            || | |||  |  |  |   ||||||||||  |||||||||| | |||   ||||| ||  |||||      
Sbjct 60781 CGCTGCTCATTGAGCAGCTCATATATTAAACTGATTTTTGGAACCGGGCTGTGGAAAAGAGGCTTG 60846

Query   132 CTCTGTCCACTCCACGCATCGACCTGGTATTGCAGTACCTCC 173  
            |   ||||   |||||  | | |  ||||| ||| |||||||      
Sbjct 60847 CCTCGTCCCAGCCACGGGTTGCCTCGGTATAGCACTACCTCC 60888

 
                 
                
                 
                      Report:  
                     
                         
                             sequence start
                                   ?  
                                     
Start position of the estimated full-length sequence in genome.
Start index  
                                 :
                             
                             60715 
                         
                         
                             sequence end
                                   ?  
                                     
End position of the estimated full-length sequence in genome.
Start index  
                                 :
                             
                             60903 
                         
                         
                             bit score (CM)
                                   ?  
                                     
The score for aligning estimated full-length sequence to CM model
  (computed by RSEARCH -> default,
  infered from Rfam or provided by user) 
                                 :
                             
                             161.93 
                         
                         
                             Homology estimate
                                   ?  
                                     
Quick homology estimate:
  Not homologous: bit score   20 and bit score > 0.5 * query length
  Uncertain otherwise 
                                 :
                             
                             Homologous   
                         
                     
                    
                 
                
                
                 
                     
                          Estimated full-length sequence:  
                         
                          ? 
                             
Click checkbox to select multiple seuqences.
Fasta header format:
  UID|accession.versionSTRAND start-end 
                         
                     
                     >uid:20|NW_021126939.1fw 60715-60903
AUCGCUUCUCGGCCUUUUGGCUAAGAUCAAGUGUAGUAUCUGUUCUUAUCAGCUUAAUAU
CUGAUACGCUGCUCAUUGAGCAGCUCAUAUAUUAAACUGAUUUUUGGAACCGGGCUGUGG
AAAAGAGGCUUGCCUCGUCCCAGCCACGGGUUGCCUCGGUAUAGCACUACCUCCGAGCGC
GGCCCACUU
 
                 
                
             
            
             
                
                     
                         
                         
                             rnafold 
                             
                              ? 
                                 
Visualisation of predicted secondary structure.
To save the image:
  Right click on the image -> Save Image as. 
                             
                         
                     
                
                     
                         
                         
                             rfam-Rc 
                             
                              ? 
                                 
Visualisation of predicted secondary structure.
To save the image:
  Right click on the image -> Save Image as. 
                             
                         
                     
                
                     
                         
                         
                             TurboFold 
                             
                              ? 
                                 
Visualisation of predicted secondary structure.
To save the image:
  Right click on the image -> Save Image as. 
                             
                         
                     
                
             
            

        
             
                 Load Sequence viewer 
             
        
        
         
     
    

    
     
         
            Hit: NW_021126939.1
         
         
             
                 
                     NW_021126939.1 Acropora millepora isolate SF001 unplaced genomic scaffold, amil_sf_1.1 amil.Sc0000568, whole genome shotgun sequence 
                     
                         
 ?  
This is BLAST alignment as read from the input file 
                         
 Score = 164.0 bits (149.2), Expect = 7.28E-34
 Identities = 138/174 (79%), Gaps = 1/174 (1%)
 Strand = Plus/Plus
Query     1 ATCGCTTCTCGGCCTTTTGGCTAAGATCAAGTGTAGTATCTGTTCTTATCAGTTTAATATCTGATA 66   
            |||||||||||||||||||||||||||||||||||||||||||||||||||| |||||||||||||      
Sbjct 85697 ATCGCTTCTCGGCCTTTTGGCTAAGATCAAGTGTAGTATCTGTTCTTATCAGCTTAATATCTGATA 85762

Query    67 CG-TCCTCTATCCGAGGACAATATATTAAATGGATTTTTGGAGCAGGGAGATGGAATAGGAGCTTG 131  
            || | |||  |  |  |   ||||||||||  |||||||||| | |||   ||||| ||  |||||      
Sbjct 85763 CGCTGCTCATTGAGCAGCTCATATATTAAACTGATTTTTGGAACCGGGCTGTGGAAAAGAGGCTTG 85828

Query   132 CTCTGTCCACTCCACGCATCGACCTGGTATTGCAGTACCTCC 173  
            |   ||||   |||||  | | |  ||||| ||| |||||||      
Sbjct 85829 CCTCGTCCCAGCCACGGGTTGCCTCGGTATAGCACTACCTCC 85870

 
                 
                
                 
                      Report:  
                     
                         
                             sequence start
                                   ?  
                                     
Start position of the estimated full-length sequence in genome.
Start index  
                                 :
                             
                             85697 
                         
                         
                             sequence end
                                   ?  
                                     
End position of the estimated full-length sequence in genome.
Start index  
                                 :
                             
                             85885 
                         
                         
                             bit score (CM)
                                   ?  
                                     
The score for aligning estimated full-length sequence to CM model
  (computed by RSEARCH -> default,
  infered from Rfam or provided by user) 
                                 :
                             
                             161.93 
                         
                         
                             Homology estimate
                                   ?  
                                     
Quick homology estimate:
  Not homologous: bit score   20 and bit score > 0.5 * query length
  Uncertain otherwise 
                                 :
                             
                             Homologous   
                         
                     
                    
                 
                
                
                 
                     
                          Estimated full-length sequence:  
                         
                          ? 
                             
Click checkbox to select multiple seuqences.
Fasta header format:
  UID|accession.versionSTRAND start-end 
                         
                     
                     >uid:21|NW_021126939.1fw 85697-85885
AUCGCUUCUCGGCCUUUUGGCUAAGAUCAAGUGUAGUAUCUGUUCUUAUCAGCUUAAUAU
CUGAUACGCUGCUCAUUGAGCAGCUCAUAUAUUAAACUGAUUUUUGGAACCGGGCUGUGG
AAAAGAGGCUUGCCUCGUCCCAGCCACGGGUUGCCUCGGUAUAGCACUACCUCCGAGCGC
GGCCCACUU
 
                 
                
             
            
             
                
                     
                         
                         
                             rnafold 
                             
                              ? 
                                 
Visualisation of predicted secondary structure.
To save the image:
  Right click on the image -> Save Image as. 
                             
                         
                     
                
                     
                         
                         
                             rfam-Rc 
                             
                              ? 
                                 
Visualisation of predicted secondary structure.
To save the image:
  Right click on the image -> Save Image as. 
                             
                         
                     
                
                     
                         
                         
                             TurboFold 
                             
                              ? 
                                 
Visualisation of predicted secondary structure.
To save the image:
  Right click on the image -> Save Image as. 
                             
                         
                     
                
             
            

        
             
                 Load Sequence viewer 
             
        
        
         
     
    

    
     
         
            Hit: NW_021126939.1
         
         
             
                 
                     NW_021126939.1 Acropora millepora isolate SF001 unplaced genomic scaffold, amil_sf_1.1 amil.Sc0000568, whole genome shotgun sequence 
                     
                         
 ?  
This is BLAST alignment as read from the input file 
                         
 Score = 121.0 bits (110.4), Expect = 6.39E-22
 Identities = 88/105 (84%), Gaps = 1/105 (1%)
 Strand = Plus/Plus
Query     4 GCTTCTCGGCCTTTTGGCTAAGATCAAGTGTAGTATCTGTTCTTATCAGTTTAATATCTGATACG- 68   
            |||||||||  ||||||||||||||||||||||| |||||||||||||| |||||||||||||||       
Sbjct 99250 GCTTCTCGGTTTTTTGGCTAAGATCAAGTGTAGTGTCTGTTCTTATCAGCTTAATATCTGATACGC 99315

Query    69 TCCTCTATCCGAGGACAATATATTAAATGGATTTTTGGA 107  
            | |||  |  |  |   ||||||||||  ||||||||||      
Sbjct 99316 TGCTCATTGAGCAGCTCATATATTAAACTGATTTTTGGA 99354

 
                 
                
                 
                      Report:  
                     
                         
                             sequence start
                                   ?  
                                     
Start position of the estimated full-length sequence in genome.
Start index  
                                 :
                             
                             99247 
                         
                         
                             sequence end
                                   ?  
                                     
End position of the estimated full-length sequence in genome.
Start index  
                                 :
                             
                             99433 
                         
                         
                             bit score (CM)
                                   ?  
                                     
The score for aligning estimated full-length sequence to CM model
  (computed by RSEARCH -> default,
  infered from Rfam or provided by user) 
                                 :
                             
                             58.95 
                         
                         
                             Homology estimate
                                   ?  
                                     
Quick homology estimate:
  Not homologous: bit score   20 and bit score > 0.5 * query length
  Uncertain otherwise 
                                 :
                             
                             Uncertain  ↴  
                         
                     
                    
                     
                        Check the secondary structure and sequence viewer
                        for supporting information about possible homology.
                     
                    
                 
                
                
                 
                     
                          Estimated full-length sequence:  
                         
                          ? 
                             
Click checkbox to select multiple seuqences.
Fasta header format:
  UID|accession.versionSTRAND start-end 
                         
                     
                     >uid:22|NW_021126939.1fw 99247-99433
GUUGCUUCUCGGUUUUUUGGCUAAGAUCAAGUGUAGUGUCUGUUCUUAUCAGCUUAAUAU
CUGAUACGCUGCUCAUUGAGCAGCUCAUAUAUUAAACUGAUUUUUGGAUUCUGUCUCGUC
CAAAUUGGAUGUCUCCGUACCCACCAUUCUUUGCGGUGAUUUUAACUGCGUUCUUGAUCG
UUCAGUU
 
                 
                
             
            
             
                
                     
                         
                         
                             rnafold 
                             
                              ? 
                                 
Visualisation of predicted secondary structure.
To save the image:
  Right click on the image -> Save Image as. 
                             
                         
                     
                
                     
                         
                         
                             rfam-Rc 
                             
                              ? 
                                 
Visualisation of predicted secondary structure.
To save the image:
  Right click on the image -> Save Image as. 
                             
                         
                     
                
                     
                         
                         
                             TurboFold 
                             
                              ? 
                                 
Visualisation of predicted secondary structure.
To save the image:
  Right click on the image -> Save Image as. 
                             
                         
                     
                
             
            

        
             
                 Load Sequence viewer 
             
        
        
         
     
    

    
     
         
            Hit: NW_021126939.1
         
         
             
                 
                     NW_021126939.1 Acropora millepora isolate SF001 unplaced genomic scaffold, amil_sf_1.1 amil.Sc0000568, whole genome shotgun sequence 
                     
                         
 ?  
This is BLAST alignment as read from the input file 
                         
 Score = 90.0 bits (82.4), Expect = 8.88E-14
 Identities = 101/137 (74%), Gaps = 1/137 (1%)
 Strand = Plus/Minus
Query    38 ATCTGTTCTTATCAGTTTAATATCTGATACG-TCCTCTATCCGAGGACAATATATTAAATGGATTT 102  
            ||||||||||||||| ||||||||||||||| | |||  |  |  |   ||||||||||  |||||      
Sbjct 30391 ATCTGTTCTTATCAGCTTAATATCTGATACGCTGCTCATTGAGCAGCTCATATATTAAACTGATTT 30326

Query   103 TTGGAGCAGGGAGATGGAATAGGAGCTTGCTCTGTCCACTCCACGCATCGACCTGGTATTGCAGTA 168  
            ||||| | |||   ||||| ||  ||||||   ||||   |||||  | | |  ||||| ||| ||      
Sbjct 30325 TTGGAACCGGGCTGTGGAAAAGAGGCTTGCCTCGTCCCAGCCACGGGTTGCCTCGGTATAGCACTA 30260

Query   169 CCTCC 173  
            |||||      
Sbjct 30259 CCTCC 30255

 
                 
                
                 
                      Report:  
                     
                         
                             sequence start
                                   ?  
                                     
Start position of the estimated full-length sequence in genome.
Start index  
                                 :
                             
                             30243 
                         
                         
                             sequence end
                                   ?  
                                     
End position of the estimated full-length sequence in genome.
Start index  
                                 :
                             
                             30428 
                         
                         
                             bit score (CM)
                                   ?  
                                     
The score for aligning estimated full-length sequence to CM model
  (computed by RSEARCH -> default,
  infered from Rfam or provided by user) 
                                 :
                             
                             97.05 
                         
                         
                             Homology estimate
                                   ?  
                                     
Quick homology estimate:
  Not homologous: bit score   20 and bit score > 0.5 * query length
  Uncertain otherwise 
                                 :
                             
                             Homologous   
                         
                     
                    
                 
                
                
                 
                     
                          Estimated full-length sequence:  
                         
                          ? 
                             
Click checkbox to select multiple seuqences.
Fasta header format:
  UID|accession.versionSTRAND start-end 
                         
                     
                     >uid:23|NW_021126939.1rc 30243-30428
CACAUUAACUGAAUACAAUUUCAAGAAAAAAAAAAUCUGUUCUUAUCAGCUUAAUAUCUG
AUACGCUGCUCAUUGAGCAGCUCAUAUAUUAAACUGAUUUUUGGAACCGGGCUGUGGAAA
AGAGGCUUGCCUCGUCCCAGCCACGGGUUGCCUCGGUAUAGCACUACCUCCGAGCGUGGC
CCACUU
 
                 
                
             
            
             
                
                     
                         
                         
                             rnafold 
                             
                              ? 
                                 
Visualisation of predicted secondary structure.
To save the image:
  Right click on the image -> Save Image as. 
                             
                         
                     
                
                     
                         
                         
                             rfam-Rc 
                             
                              ? 
                                 
Visualisation of predicted secondary structure.
To save the image:
  Right click on the image -> Save Image as. 
                             
                         
                     
                
                     
                         
                         
                             TurboFold 
                             
                              ? 
                                 
Visualisation of predicted secondary structure.
To save the image:
  Right click on the image -> Save Image as. 
                             
                         
                     
                
             
            

        
             
                 Load Sequence viewer 
             
        
        
         
     
    

    
     
         
            Hit: NW_021126939.1
         
         
             
                 
                     NW_021126939.1 Acropora millepora isolate SF001 unplaced genomic scaffold, amil_sf_1.1 amil.Sc0000568, whole genome shotgun sequence 
                     
                         
 ?  
This is BLAST alignment as read from the input file 
                         
 Score = 74.0 bits (68.0), Expect = 1.96E-09
 Identities = 104/145 (72%), Gaps = 4/145 (3%)
 Strand = Plus/Plus
Query    38 ATCTGTTCTTATCAGTTTAATATCTGATACG-TCCTCTATCCGAGGACAATATATTAAATGGATTT 102  
            |||||||| |||||| |||||||||||| || | |||  |  |  |   ||||||||||  |||||      
Sbjct 62213 ATCTGTTCGTATCAGCTTAATATCTGATTCGCTGCTCATTGAGCAGCTCATATATTAAACTGATTT 62278

Query   103 TTGGAGCAGGGAGATGG-AATAGGAGCTTGCTCTGTCCACTCCACGCATCGACCTGGTATTGCAGT 167  
            ||||| | |||   ||| || ||  ||||||   ||||   |||||  | | |  ||||| ||| |      
Sbjct 62279 TTGGAACCGGGCTGTGGCAAAAGAGGCTTGCCTCGTCCCAGCCACGGGTTGCCTCGGTATAGCACT 62344

Query   168 ACCTCCAGGACCG 180  
            ||||||  |||||      
Sbjct 62345 ACCTCC--GACCG 62355

 
                 
                
                 
                      Report:  
                     
                         
                             sequence start
                                   ?  
                                     
Start position of the estimated full-length sequence in genome.
Start index  
                                 :
                             
                             62176 
                         
                         
                             sequence end
                                   ?  
                                     
End position of the estimated full-length sequence in genome.
Start index  
                                 :
                             
                             62365 
                         
                         
                             bit score (CM)
                                   ?  
                                     
The score for aligning estimated full-length sequence to CM model
  (computed by RSEARCH -> default,
  infered from Rfam or provided by user) 
                                 :
                             
                             85.1 
                         
                         
                             Homology estimate
                                   ?  
                                     
Quick homology estimate:
  Not homologous: bit score   20 and bit score > 0.5 * query length
  Uncertain otherwise 
                                 :
                             
                             Uncertain  ↴  
                         
                     
                    
                     
                        Check the secondary structure and sequence viewer
                        for supporting information about possible homology.
                     
                    
                 
                
                
                 
                     
                          Estimated full-length sequence:  
                         
                          ? 
                             
Click checkbox to select multiple seuqences.
Fasta header format:
  UID|accession.versionSTRAND start-end 
                         
                     
                     >uid:24|NW_021126939.1fw 62176-62365
AUAUAUUUUCGAAUAAAAGUUUUAAAAUACAAAAAAAAUCUGUUCGUAUCAGCUUAAUAU
CUGAUUCGCUGCUCAUUGAGCAGCUCAUAUAUUAAACUGAUUUUUGGAACCGGGCUGUGG
CAAAAGAGGCUUGCCUCGUCCCAGCCACGGGUUGCCUCGGUAUAGCACUACCUCCGACCG
CGGCCCAAUU
 
                 
                
             
            
             
                
                     
                         
                         
                             rnafold 
                             
                              ? 
                                 
Visualisation of predicted secondary structure.
To save the image:
  Right click on the image -> Save Image as. 
                             
                         
                     
                
                     
                         
                         
                             rfam-Rc 
                             
                              ? 
                                 
Visualisation of predicted secondary structure.
To save the image:
  Right click on the image -> Save Image as. 
                             
                         
                     
                
                     
                         
                         
                             TurboFold 
                             
                              ? 
                                 
Visualisation of predicted secondary structure.
To save the image:
  Right click on the image -> Save Image as. 
                             
                         
                     
                
             
            

        
             
                 Load Sequence viewer 
             
        
        
         
     
    

    
     
         
            Hit: NW_021126939.1
         
         
             
                 
                     NW_021126939.1 Acropora millepora isolate SF001 unplaced genomic scaffold, amil_sf_1.1 amil.Sc0000568, whole genome shotgun sequence 
                     
                         
 ?  
This is BLAST alignment as read from the input file 
                         
 Score = 68.0 bits (62.6), Expect = 8.31E-08
 Identities = 34/34 (100%), Gaps = 0/34 (0%)
 Strand = Plus/Minus
Query     1 ATCGCTTCTCGGCCTTTTGGCTAAGATCAAGTGT 34   
            ||||||||||||||||||||||||||||||||||      
Sbjct 34780 ATCGCTTCTCGGCCTTTTGGCTAAGATCAAGTGT 34747

 
                 
                
                 
                      Report:  
                     
                         
                             sequence start
                                   ?  
                                     
Start position of the estimated full-length sequence in genome.
Start index  
                                 :
                             
                             34593 
                         
                         
                             sequence end
                                   ?  
                                     
End position of the estimated full-length sequence in genome.
Start index  
                                 :
                             
                             34787 
                         
                         
                             bit score (CM)
                                   ?  
                                     
The score for aligning estimated full-length sequence to CM model
  (computed by RSEARCH -> default,
  infered from Rfam or provided by user) 
                                 :
                             
                             20.87 
                         
                         
                             Homology estimate
                                   ?  
                                     
Quick homology estimate:
  Not homologous: bit score   20 and bit score > 0.5 * query length
  Uncertain otherwise 
                                 :
                             
                             Uncertain  ↴  
                         
                     
                    
                     
                        Check the secondary structure and sequence viewer
                        for supporting information about possible homology.
                     
                    
                 
                
                
                 
                     
                          Estimated full-length sequence:  
                         
                          ? 
                             
Click checkbox to select multiple seuqences.
Fasta header format:
  UID|accession.versionSTRAND start-end 
                         
                     
                     >uid:25|NW_021126939.1rc 34593-34787
AUCGCUUCUCGGCCUUUUGGCUAAGAUCAAGUGUUCUUGUAUCUUGCCAUUAGGAUUGUU
AGGUUAUUUUCUUCGCGUCCUGUGCUAAGUACCCGCCGGGGGGAACGCCAUGGAGUCUCC
UCGUGUGAGUAGCGAUUCUUCACACGCUAUCGAGGCCAAUGCUAACGUCUUGGUGACGUC
AGCGGCACAUGGUUU
 
                 
                
             
            
             
                
                     
                         
                         
                             rnafold 
                             
                              ? 
                                 
Visualisation of predicted secondary structure.
To save the image:
  Right click on the image -> Save Image as. 
                             
                         
                     
                
                     
                         
                         
                             rfam-Rc 
                             
                              ? 
                                 
Visualisation of predicted secondary structure.
To save the image:
  Right click on the image -> Save Image as. 
                             
                         
                     
                
                     
                         
                         
                             TurboFold 
                             
                              ? 
                                 
Visualisation of predicted secondary structure.
To save the image:
  Right click on the image -> Save Image as. 
                             
                         
                     
                
             
            

        
             
                 Load Sequence viewer 
             
        
        
         
     
    

    
     
         
            Hit: NW_021126939.1
         
         
             
                 
                     NW_021126939.1 Acropora millepora isolate SF001 unplaced genomic scaffold, amil_sf_1.1 amil.Sc0000568, whole genome shotgun sequence 
                     
                         
 ?  
This is BLAST alignment as read from the input file 
                         
 Score = 45.0 bits (41.9), Expect = 2.72E-01
 Identities = 24/25 (96%), Gaps = 0/25 (0%)
 Strand = Plus/Minus
Query     1 ATCGCTTCTCGGCCTTTTGGCTAAG 25   
            |||||||||| ||||||||||||||      
Sbjct 20354 ATCGCTTCTCAGCCTTTTGGCTAAG 20330

 
                 
                
                 
                      Report:  
                     
                         
                             sequence start
                                   ?  
                                     
Start position of the estimated full-length sequence in genome.
Start index  
                                 :
                             
                             20167 
                         
                         
                             sequence end
                                   ?  
                                     
End position of the estimated full-length sequence in genome.
Start index  
                                 :
                             
                             20335 
                         
                         
                             bit score (CM)
                                   ?  
                                     
The score for aligning estimated full-length sequence to CM model
  (computed by RSEARCH -> default,
  infered from Rfam or provided by user) 
                                 :
                             
                             -1.22 
                         
                         
                             Homology estimate
                                   ?  
                                     
Quick homology estimate:
  Not homologous: bit score   20 and bit score > 0.5 * query length
  Uncertain otherwise 
                                 :
                             
                             Not homologous   
                         
                     
                    
                 
                
                
                 
                     
                          Estimated full-length sequence:  
                         
                          ? 
                             
Click checkbox to select multiple seuqences.
Fasta header format:
  UID|accession.versionSTRAND start-end 
                         
                     
                     >uid:26|NW_021126939.1rc 20167-20335
AUCGCUUCUCAGCCUUUUGGCUAAGCCAUUUUGCUGCGUUGAGUUGUGGGGUCCCCCAGG
GUGAAGCCCUAUCGGUCAGUGAAGAGAAGUUGUUUUGCGCUCUACCUCUUUGGAGGUAUC
UACAAUGGAUAACGAUCUUGGUAAUGGUUCCCAGAAUUGGGGUGAUUCU
 
                 
                
             
            
             
                
                     
                         
                         
                             rnafold 
                             
                              ? 
                                 
Visualisation of predicted secondary structure.
To save the image:
  Right click on the image -> Save Image as. 
                             
                         
                     
                
                     
                         
                         
                             rfam-Rc 
                             
                              ? 
                                 
Visualisation of predicted secondary structure.
To save the image:
  Right click on the image -> Save Image as. 
                             
                         
                     
                
                     
                         
                         
                             TurboFold 
                             
                              ? 
                                 
Visualisation of predicted secondary structure.
To save the image:
  Right click on the image -> Save Image as. 
                             
                         
                     
                
             
            

        
             
                 Load Sequence viewer 
             
        
        
         
     
    

    
     
         
            Hit: NW_021126939.1
         
         
             
                 
                     NW_021126939.1 Acropora millepora isolate SF001 unplaced genomic scaffold, amil_sf_1.1 amil.Sc0000568, whole genome shotgun sequence 
                     
                         
 ?  
This is BLAST alignment as read from the input file 
                         
 Score = 41.0 bits (38.3), Expect = 3.31E+00
 Identities = 25/28 (89%), Gaps = 0/28 (0%)
 Strand = Plus/Minus
Query     1 ATCGCTTCTCGGCCTTTTGGCTAAGATC 28   
            |||||||||||||||||||  |||| ||      
Sbjct 10316 ATCGCTTCTCGGCCTTTTGAGTAAGGTC 10289

 
                 
                
                 
                      Report:  
                     
                         
                             sequence start
                                   ?  
                                     
Start position of the estimated full-length sequence in genome.
Start index  
                                 :
                             
                             10129 
                         
                         
                             sequence end
                                   ?  
                                     
End position of the estimated full-length sequence in genome.
Start index  
                                 :
                             
                             10306 
                         
                         
                             bit score (CM)
                                   ?  
                                     
The score for aligning estimated full-length sequence to CM model
  (computed by RSEARCH -> default,
  infered from Rfam or provided by user) 
                                 :
                             
                             -8.95 
                         
                         
                             Homology estimate
                                   ?  
                                     
Quick homology estimate:
  Not homologous: bit score   20 and bit score > 0.5 * query length
  Uncertain otherwise 
                                 :
                             
                             Not homologous   
                         
                     
                    
                 
                
                
                 
                     
                          Estimated full-length sequence:  
                         
                          ? 
                             
Click checkbox to select multiple seuqences.
Fasta header format:
  UID|accession.versionSTRAND start-end 
                         
                     
                     >uid:27|NW_021126939.1rc 10129-10306
AUCGCUUCUCGGCCUUUUGAGUAAGGUCGGUGUUUUCUUGCUUUUGCAGAGAGAUGGCCG
AGGCGAUAGAACCAUAUGACGAAAAUGCAAUGGUAGACGUGCCAGAAAGAGAAGAUUUAG
UGAUUACAGUAACCCGAGAACGUACCAGUGACGAAUCAGGAAGUUGGGCCGAGCGGGU
 
                 
                
             
            
             
                
                     
                         
                         
                             rnafold 
                             
                              ? 
                                 
Visualisation of predicted secondary structure.
To save the image:
  Right click on the image -> Save Image as. 
                             
                         
                     
                
                     
                         
                         
                             rfam-Rc 
                             
                              ? 
                                 
Visualisation of predicted secondary structure.
To save the image:
  Right click on the image -> Save Image as. 
                             
                         
                     
                
                     
                         
                         
                             TurboFold 
                             
                              ? 
                                 
Visualisation of predicted secondary structure.
To save the image:
  Right click on the image -> Save Image as. 
                             
                         
                     
                
             
            

        
             
                 Load Sequence viewer 
             
        
        
         
     
    

    
     
         
            Hit: NW_021127469.1
         
         
             
                 
                     NW_021127469.1 Acropora millepora isolate SF001 unplaced genomic scaffold, amil_sf_1.1 amil.xfSc0000099, whole genome shotgun sequence 
                     
                         
 ?  
This is BLAST alignment as read from the input file 
                         
 Score = 164.0 bits (149.2), Expect = 7.28E-34
 Identities = 138/174 (79%), Gaps = 1/174 (1%)
 Strand = Plus/Minus
Query      1 ATCGCTTCTCGGCCTTTTGGCTAAGATCAAGTGTAGTATCTGTTCTTATCAGTTTAATATCTGA 64    
             |||||||||||||||||||||||||||||||||||||||||||||||||||| |||||||||||       
Sbjct 171994 ATCGCTTCTCGGCCTTTTGGCTAAGATCAAGTGTAGTATCTGTTCTTATCAGCTTAATATCTGA 171931

Query     65 TACG-TCCTCTATCCGAGGACAATATATTAAATGGATTTTTGGAGCAGGGAGATGGAATAGGAG 127   
             |||| | |||  |  |  |   ||||||||||  |||||||||| | |||   ||||| ||  |       
Sbjct 171930 TACGCTGCTCATTGAGCAGCTCATATATTAAACTGATTTTTGGAACCGGGCTGTGGAAAAGAGG 171867

Query    128 CTTGCTCTGTCCACTCCACGCATCGACCTGGTATTGCAGTACCTCC 173   
             |||||   ||||   |||||  | | |  ||||| ||| |||||||       
Sbjct 171866 CTTGCCTCGTCCCAGCCACGGGTTGCCTCGGTATAGCACTACCTCC 171821

 
                 
                
                 
                      Report:  
                     
                         
                             sequence start
                                   ?  
                                     
Start position of the estimated full-length sequence in genome.
Start index  
                                 :
                             
                             171806 
                         
                         
                             sequence end
                                   ?  
                                     
End position of the estimated full-length sequence in genome.
Start index  
                                 :
                             
                             171994 
                         
                         
                             bit score (CM)
                                   ?  
                                     
The score for aligning estimated full-length sequence to CM model
  (computed by RSEARCH -> default,
  infered from Rfam or provided by user) 
                                 :
                             
                             161.93 
                         
                         
                             Homology estimate
                                   ?  
                                     
Quick homology estimate:
  Not homologous: bit score   20 and bit score > 0.5 * query length
  Uncertain otherwise 
                                 :
                             
                             Homologous   
                         
                     
                    
                 
                
                
                 
                     
                          Estimated full-length sequence:  
                         
                          ? 
                             
Click checkbox to select multiple seuqences.
Fasta header format:
  UID|accession.versionSTRAND start-end 
                         
                     
                     >uid:28|NW_021127469.1rc 171806-171994
AUCGCUUCUCGGCCUUUUGGCUAAGAUCAAGUGUAGUAUCUGUUCUUAUCAGCUUAAUAU
CUGAUACGCUGCUCAUUGAGCAGCUCAUAUAUUAAACUGAUUUUUGGAACCGGGCUGUGG
AAAAGAGGCUUGCCUCGUCCCAGCCACGGGUUGCCUCGGUAUAGCACUACCUCCGAGCGC
GGCCCACUU
 
                 
                
             
            
             
                
                     
                         
                         
                             rnafold 
                             
                              ? 
                                 
Visualisation of predicted secondary structure.
To save the image:
  Right click on the image -> Save Image as. 
                             
                         
                     
                
                     
                         
                         
                             rfam-Rc 
                             
                              ? 
                                 
Visualisation of predicted secondary structure.
To save the image:
  Right click on the image -> Save Image as. 
                             
                         
                     
                
                     
                         
                         
                             TurboFold 
                             
                              ? 
                                 
Visualisation of predicted secondary structure.
To save the image:
  Right click on the image -> Save Image as. 
                             
                         
                     
                
             
            

        
             
                 Load Sequence viewer 
             
        
        
         
     
    

    
     
         
            Hit: NW_021127469.1
         
         
             
                 
                     NW_021127469.1 Acropora millepora isolate SF001 unplaced genomic scaffold, amil_sf_1.1 amil.xfSc0000099, whole genome shotgun sequence 
                     
                         
 ?  
This is BLAST alignment as read from the input file 
                         
 Score = 61.0 bits (56.3), Expect = 1.23E-05
 Identities = 99/141 (70%), Gaps = 9/141 (6%)
 Strand = Plus/Minus
Query  38 ATCTGTTCTTATCAGTTTAATATCTGATACG-TCCTCTATCCGAGGACA----ATATATTAAATGGATTT 102
          |||||||||||| || ||||||||||||||| | ||| ||    | |||    ||||||||||   ||||    
Sbjct 878 ATCTGTTCTTATAAGCTTAATATCTGATACGCTGCTC-AT---TGAACAGCTCATATATTAAACTAATTT 813

Query 103 TTGGAGCAGGGAGATGGAATAGGAGCTTGCTCTGTCCACTCCACGCATCGACCTGGTATTGCAGTACCTC 172
          ||||| |  ||   ||||| ||  ||||||   ||||   ||||   | | |  ||||  ||| ||||||    
Sbjct 812 TTGGAACTAGGCTGTGGAAAAGAGGCTTGCCTCGTCCCAGCCACTGGTTGCCTCGGTACAGCACTACCTC 743

Query 173 C 173
          |    
Sbjct 742 C 742

 
                 
                
                 
                      Report:  
                     
                         
                             sequence start
                                   ?  
                                     
Start position of the estimated full-length sequence in genome.
Start index  
                                 :
                             
                             723 
                         
                         
                             sequence end
                                   ?  
                                     
End position of the estimated full-length sequence in genome.
Start index  
                                 :
                             
                             915 
                         
                         
                             bit score (CM)
                                   ?  
                                     
The score for aligning estimated full-length sequence to CM model
  (computed by RSEARCH -> default,
  infered from Rfam or provided by user) 
                                 :
                             
                             69.66 
                         
                         
                             Homology estimate
                                   ?  
                                     
Quick homology estimate:
  Not homologous: bit score   20 and bit score > 0.5 * query length
  Uncertain otherwise 
                                 :
                             
                             Uncertain  ↴  
                         
                     
                    
                     
                        Check the secondary structure and sequence viewer
                        for supporting information about possible homology.
                     
                    
                 
                
                
                 
                     
                          Estimated full-length sequence:  
                         
                          ? 
                             
Click checkbox to select multiple seuqences.
Fasta header format:
  UID|accession.versionSTRAND start-end 
                         
                     
                     >uid:29|NW_021127469.1rc 723-915
AUGAAGGACUUUGACCGAAUAAAGUUUCUAUAAAAAAAAAAAUCUGUUCUUAUAAGCUUA
AUAUCUGAUACGCUGCUCAUUGAACAGCUCAUAUAUUAAACUAAUUUUUGGAACUAGGCU
GUGGAAAAGAGGCUUGCCUCGUCCCAGCCACUGGUUGCCUCGGUACAGCACUACCUCCGA
GCGCGGCCCACCU
 
                 
                
             
            
             
                
                     
                         
                         
                             rnafold 
                             
                              ? 
                                 
Visualisation of predicted secondary structure.
To save the image:
  Right click on the image -> Save Image as. 
                             
                         
                     
                
                     
                         
                         
                             rfam-Rc 
                             
                              ? 
                                 
Visualisation of predicted secondary structure.
To save the image:
  Right click on the image -> Save Image as. 
                             
                         
                     
                
                     
                         
                         
                             TurboFold 
                             
                              ? 
                                 
Visualisation of predicted secondary structure.
To save the image:
  Right click on the image -> Save Image as. 
                             
                         
                     
                
             
            

        
             
                 Load Sequence viewer 
             
        
        
         
     
    

    
     
         
            Hit: NW_021127469.1
         
         
             
                 
                     NW_021127469.1 Acropora millepora isolate SF001 unplaced genomic scaffold, amil_sf_1.1 amil.xfSc0000099, whole genome shotgun sequence 
                     
                         
 ?  
This is BLAST alignment as read from the input file 
                         
 Score = 51.0 bits (47.3), Expect = 6.39E-03
 Identities = 87/127 (69%), Gaps = 7/127 (6%)
 Strand = Plus/Plus
Query    48 ATCAGTTTAATATCTGATAC-GTCCTCTATCCGAGGACAATATATTAAATGGATTTTTGGAGCAGG 112  
            ||||| |||||||||||||| || |||  |  |  |   || |||||||  |||||||||| | |       
Sbjct 49378 ATCAGCTTAATATCTGATACGGTGCTCATTGAGCAGCTCATGTATTAAACTGATTTTTGGAACCG- 49442

Query   113 GAGATGGAATAGGAGCTTGCTCTGTCCACTCCACGCATCGACCTGGTATTGCAGTACCTCC 173  
                 |||| ||  ||||||   ||||   ||| |  | | | |||||| ||| |||||||      
Sbjct 49443 -----GGAAAAGAGGCTTGCCTCGTCCCAGCCATGGGTTGCCTTGGTATAGCACTACCTCC 49498

 
                 
                
                 
                      Report:  
                     
                         
                             sequence start
                                   ?  
                                     
Start position of the estimated full-length sequence in genome.
Start index  
                                 :
                             
                             49341 
                         
                         
                             sequence end
                                   ?  
                                     
End position of the estimated full-length sequence in genome.
Start index  
                                 :
                             
                             49513 
                         
                         
                             bit score (CM)
                                   ?  
                                     
The score for aligning estimated full-length sequence to CM model
  (computed by RSEARCH -> default,
  infered from Rfam or provided by user) 
                                 :
                             
                             64.93 
                         
                         
                             Homology estimate
                                   ?  
                                     
Quick homology estimate:
  Not homologous: bit score   20 and bit score > 0.5 * query length
  Uncertain otherwise 
                                 :
                             
                             Uncertain  ↴  
                         
                     
                    
                     
                        Check the secondary structure and sequence viewer
                        for supporting information about possible homology.
                     
                    
                 
                
                
                 
                     
                          Estimated full-length sequence:  
                         
                          ? 
                             
Click checkbox to select multiple seuqences.
Fasta header format:
  UID|accession.versionSTRAND start-end 
                         
                     
                     >uid:30|NW_021127469.1fw 49341-49513
AAAAAUUUGUGAAUAAAGUUUACAAAAAUGUCUUAUAAUCAGCUUAAUAUCUGAUACGGU
GCUCAUUGAGCAGCUCAUGUAUUAAACUGAUUUUUGGAACCGGGAAAAGAGGCUUGCCUC
GUCCCAGCCAUGGGUUGCCUUGGUAUAGCACUACCUCCGAGCGCAGCCCACUU
 
                 
                
             
            
             
                
                     
                         
                         
                             rnafold 
                             
                              ? 
                                 
Visualisation of predicted secondary structure.
To save the image:
  Right click on the image -> Save Image as. 
                             
                         
                     
                
                     
                         
                         
                             rfam-Rc 
                             
                              ? 
                                 
Visualisation of predicted secondary structure.
To save the image:
  Right click on the image -> Save Image as. 
                             
                         
                     
                
                     
                         
                         
                             TurboFold 
                             
                              ? 
                                 
Visualisation of predicted secondary structure.
To save the image:
  Right click on the image -> Save Image as. 
                             
                         
                     
                
             
            

        
             
                 Load Sequence viewer 
             
        
        
         
     
    

    
     
         
            Hit: NW_021127469.1
         
         
             
                 
                     NW_021127469.1 Acropora millepora isolate SF001 unplaced genomic scaffold, amil_sf_1.1 amil.xfSc0000099, whole genome shotgun sequence 
                     
                         
 ?  
This is BLAST alignment as read from the input file 
                         
 Score = 50.0 bits (46.4), Expect = 6.39E-03
 Identities = 25/25 (100%), Gaps = 0/25 (0%)
 Strand = Plus/Minus
Query      1 ATCGCTTCTCGGCCTTTTGGCTAAG 25    
             |||||||||||||||||||||||||       
Sbjct 163136 ATCGCTTCTCGGCCTTTTGGCTAAG 163112

 
                 
                
                 
                      Report:  
                     
                         
                             sequence start
                                   ?  
                                     
Start position of the estimated full-length sequence in genome.
Start index  
                                 :
                             
                             162949 
                         
                         
                             sequence end
                                   ?  
                                     
End position of the estimated full-length sequence in genome.
Start index  
                                 :
                             
                             163130 
                         
                         
                             bit score (CM)
                                   ?  
                                     
The score for aligning estimated full-length sequence to CM model
  (computed by RSEARCH -> default,
  infered from Rfam or provided by user) 
                                 :
                             
                             -0.56 
                         
                         
                             Homology estimate
                                   ?  
                                     
Quick homology estimate:
  Not homologous: bit score   20 and bit score > 0.5 * query length
  Uncertain otherwise 
                                 :
                             
                             Not homologous   
                         
                     
                    
                 
                
                
                 
                     
                          Estimated full-length sequence:  
                         
                          ? 
                             
Click checkbox to select multiple seuqences.
Fasta header format:
  UID|accession.versionSTRAND start-end 
                         
                     
                     >uid:31|NW_021127469.1rc 162949-163130
AUCGCUUCUCGGCCUUUUGGCUAAGUCCAAAAGGCUCGGUUUGCUUUGUAUCUCCAUAGG
UUUUCGUCACGUUAAACACUGCUAUGGAGAGCGCUGAAGGUACUCAGGAUACGGAGACAU
UUGAAGAAAGUACAUCUUGGAACGAAACCAUGGAACAAGAAACGACUGAGGUUGCUGGAG
UG
 
                 
                
             
            
             
                
                     
                         
                         
                             rnafold 
                             
                              ? 
                                 
Visualisation of predicted secondary structure.
To save the image:
  Right click on the image -> Save Image as. 
                             
                         
                     
                
                     
                         
                         
                             rfam-Rc 
                             
                              ? 
                                 
Visualisation of predicted secondary structure.
To save the image:
  Right click on the image -> Save Image as. 
                             
                         
                     
                
                     
                         
                         
                             TurboFold 
                             
                              ? 
                                 
Visualisation of predicted secondary structure.
To save the image:
  Right click on the image -> Save Image as. 
                             
                         
                     
                
             
            

        
             
                 Load Sequence viewer 
             
        
        
         
     
    

    
     
         
            Hit: NW_021127469.1
         
         
             
                 
                     NW_021127469.1 Acropora millepora isolate SF001 unplaced genomic scaffold, amil_sf_1.1 amil.xfSc0000099, whole genome shotgun sequence 
                     
                         
 ?  
This is BLAST alignment as read from the input file 
                         
 Score = 50.0 bits (46.4), Expect = 6.39E-03
 Identities = 25/25 (100%), Gaps = 0/25 (0%)
 Strand = Plus/Minus
Query      1 ATCGCTTCTCGGCCTTTTGGCTAAG 25    
             |||||||||||||||||||||||||       
Sbjct 166663 ATCGCTTCTCGGCCTTTTGGCTAAG 166639

 
                 
                
                 
                      Report:  
                     
                         
                             sequence start
                                   ?  
                                     
Start position of the estimated full-length sequence in genome.
Start index  
                                 :
                             
                             166476 
                         
                         
                             sequence end
                                   ?  
                                     
End position of the estimated full-length sequence in genome.
Start index  
                                 :
                             
                             166667 
                         
                         
                             bit score (CM)
                                   ?  
                                     
The score for aligning estimated full-length sequence to CM model
  (computed by RSEARCH -> default,
  infered from Rfam or provided by user) 
                                 :
                             
                             6.13 
                         
                         
                             Homology estimate
                                   ?  
                                     
Quick homology estimate:
  Not homologous: bit score   20 and bit score > 0.5 * query length
  Uncertain otherwise 
                                 :
                             
                             Uncertain  ↴  
                         
                     
                    
                     
                        Check the secondary structure and sequence viewer
                        for supporting information about possible homology.
                     
                    
                 
                
                
                 
                     
                          Estimated full-length sequence:  
                         
                          ? 
                             
Click checkbox to select multiple seuqences.
Fasta header format:
  UID|accession.versionSTRAND start-end 
                         
                     
                     >uid:32|NW_021127469.1rc 166476-166667
AUCGCUUCUCGGCCUUUUGGCUAAGUCGUGAUCAGGUGACGUUUUCGUUUUUGUUCUUUC
AGAUUGUUAUGCCGAUGGAGGACGUAUUUCCGAAGAGUGUAGUGUUACACUUCCCGCCGA
GCAUCUAUCAGAGAACUGGAGCAAGUACCAUGUUACCGAAGUUGCUACAAAUCCUCAACU
CUGAUGAUCUCA
 
                 
                
             
            
             
                
                     
                         
                         
                             rnafold 
                             
                              ? 
                                 
Visualisation of predicted secondary structure.
To save the image:
  Right click on the image -> Save Image as. 
                             
                         
                     
                
                     
                         
                         
                             rfam-Rc 
                             
                              ? 
                                 
Visualisation of predicted secondary structure.
To save the image:
  Right click on the image -> Save Image as. 
                             
                         
                     
                
                     
                         
                         
                             TurboFold 
                             
                              ? 
                                 
Visualisation of predicted secondary structure.
To save the image:
  Right click on the image -> Save Image as. 
                             
                         
                     
                
             
            

        
             
                 Load Sequence viewer 
             
        
        
         
     
    

    
     
         
            Hit: NW_021127469.1
         
         
             
                 
                     NW_021127469.1 Acropora millepora isolate SF001 unplaced genomic scaffold, amil_sf_1.1 amil.xfSc0000099, whole genome shotgun sequence 
                     
                         
 ?  
This is BLAST alignment as read from the input file 
                         
 Score = 49.0 bits (45.5), Expect = 2.23E-02
 Identities = 26/27 (96%), Gaps = 0/27 (0%)
 Strand = Plus/Minus
Query      1 ATCGCTTCTCGGCCTTTTGGCTAAGAT 27    
             ||||||||||||||||||||| |||||       
Sbjct 155523 ATCGCTTCTCGGCCTTTTGGCCAAGAT 155497

 
                 
                
                 
                      Report:  
                     
                         
                             sequence start
                                   ?  
                                     
Start position of the estimated full-length sequence in genome.
Start index  
                                 :
                             
                             155336 
                         
                         
                             sequence end
                                   ?  
                                     
End position of the estimated full-length sequence in genome.
Start index  
                                 :
                             
                             155526 
                         
                         
                             bit score (CM)
                                   ?  
                                     
The score for aligning estimated full-length sequence to CM model
  (computed by RSEARCH -> default,
  infered from Rfam or provided by user) 
                                 :
                             
                             4.85 
                         
                         
                             Homology estimate
                                   ?  
                                     
Quick homology estimate:
  Not homologous: bit score   20 and bit score > 0.5 * query length
  Uncertain otherwise 
                                 :
                             
                             Uncertain  ↴  
                         
                     
                    
                     
                        Check the secondary structure and sequence viewer
                        for supporting information about possible homology.
                     
                    
                 
                
                
                 
                     
                          Estimated full-length sequence:  
                         
                          ? 
                             
Click checkbox to select multiple seuqences.
Fasta header format:
  UID|accession.versionSTRAND start-end 
                         
                     
                     >uid:33|NW_021127469.1rc 155336-155526
AUCGCUUCUCGGCCUUUUGGCCAAGAUUUUCUUUCUUGCUUUUUGCAGAGAGGGCGAUUA
GAAGCGAUGGCGGAUGCCGAUGCAGAUGCGGAGAUGUUAGUAGAAGUUGCGGAGAACCCA
AGAGAAGAUGUUGUGAUUACCUUGCCGAGCGAUGAACGAUCCAGUGACGUACCUCCGAGA
UGGGCAGAUAU
 
                 
                
             
            
             
                
                     
                         
                         
                             rnafold 
                             
                              ? 
                                 
Visualisation of predicted secondary structure.
To save the image:
  Right click on the image -> Save Image as. 
                             
                         
                     
                
                     
                         
                         
                             rfam-Rc 
                             
                              ? 
                                 
Visualisation of predicted secondary structure.
To save the image:
  Right click on the image -> Save Image as. 
                             
                         
                     
                
                     
                         
                         
                             TurboFold 
                             
                              ? 
                                 
Visualisation of predicted secondary structure.
To save the image:
  Right click on the image -> Save Image as. 
                             
                         
                     
                
             
            

        
             
                 Load Sequence viewer 
             
        
        
         
     
    

    
     
         
            Hit: NW_021127469.1
         
         
             
                 
                     NW_021127469.1 Acropora millepora isolate SF001 unplaced genomic scaffold, amil_sf_1.1 amil.xfSc0000099, whole genome shotgun sequence 
                     
                         
 ?  
This is BLAST alignment as read from the input file 
                         
 Score = 45.0 bits (41.9), Expect = 2.72E-01
 Identities = 24/25 (96%), Gaps = 0/25 (0%)
 Strand = Plus/Minus
Query      1 ATCGCTTCTCGGCCTTTTGGCTAAG 25    
             || ||||||||||||||||||||||       
Sbjct 107638 ATAGCTTCTCGGCCTTTTGGCTAAG 107614

 
                 
                
                 
                      Report:  
                     
                         
                             sequence start
                                   ?  
                                     
Start position of the estimated full-length sequence in genome.
Start index  
                                 :
                             
                             107451 
                         
                         
                             sequence end
                                   ?  
                                     
End position of the estimated full-length sequence in genome.
Start index  
                                 :
                             
                             107633 
                         
                         
                             bit score (CM)
                                   ?  
                                     
The score for aligning estimated full-length sequence to CM model
  (computed by RSEARCH -> default,
  infered from Rfam or provided by user) 
                                 :
                             
                             -2.6 
                         
                         
                             Homology estimate
                                   ?  
                                     
Quick homology estimate:
  Not homologous: bit score   20 and bit score > 0.5 * query length
  Uncertain otherwise 
                                 :
                             
                             Not homologous   
                         
                     
                    
                 
                
                
                 
                     
                          Estimated full-length sequence:  
                         
                          ? 
                             
Click checkbox to select multiple seuqences.
Fasta header format:
  UID|accession.versionSTRAND start-end 
                         
                     
                     >uid:34|NW_021127469.1rc 107451-107633
AUAGCUUCUCGGCCUUUUGGCUAAGUAUUGUCAAAUCGCCAACGCUAUUAUUAGCUGUUG
UUUUGAAGCAUUUCUUACUGCGGCCUCUUUUGCCAGUGUUGCAGCUAGUUCUAUGGUGGU
UCAACCUUCCCAGAAUAGUUCUUCGGUACGUGGUUCCUUUCGACCCCGCCGUCCUGAGUA
CUU
 
                 
                
             
            
             
                
                     
                         
                         
                             rnafold 
                             
                              ? 
                                 
Visualisation of predicted secondary structure.
To save the image:
  Right click on the image -> Save Image as. 
                             
                         
                     
                
                     
                         
                         
                             rfam-Rc 
                             
                              ? 
                                 
Visualisation of predicted secondary structure.
To save the image:
  Right click on the image -> Save Image as. 
                             
                         
                     
                
                     
                         
                         
                             TurboFold 
                             
                              ? 
                                 
Visualisation of predicted secondary structure.
To save the image:
  Right click on the image -> Save Image as. 
                             
                         
                     
                
             
            

        
             
                 Load Sequence viewer 
             
        
        
         
     
    

    
     
         
            Hit: NW_021127469.1
         
         
             
                 
                     NW_021127469.1 Acropora millepora isolate SF001 unplaced genomic scaffold, amil_sf_1.1 amil.xfSc0000099, whole genome shotgun sequence 
                     
                         
 ?  
This is BLAST alignment as read from the input file 
                         
 Score = 44.0 bits (41.0), Expect = 2.72E-01
 Identities = 25/27 (93%), Gaps = 0/27 (0%)
 Strand = Plus/Minus
Query     1 ATCGCTTCTCGGCCTTTTGGCTAAGAT 27   
            ||||||||| ||||||||||| |||||      
Sbjct 58908 ATCGCTTCTAGGCCTTTTGGCCAAGAT 58882

 
                 
                
                 
                      Report:  
                     
                         
                             sequence start
                                   ?  
                                     
Start position of the estimated full-length sequence in genome.
Start index  
                                 :
                             
                             58721 
                         
                         
                             sequence end
                                   ?  
                                     
End position of the estimated full-length sequence in genome.
Start index  
                                 :
                             
                             58906 
                         
                         
                             bit score (CM)
                                   ?  
                                     
The score for aligning estimated full-length sequence to CM model
  (computed by RSEARCH -> default,
  infered from Rfam or provided by user) 
                                 :
                             
                             -0.68 
                         
                         
                             Homology estimate
                                   ?  
                                     
Quick homology estimate:
  Not homologous: bit score   20 and bit score > 0.5 * query length
  Uncertain otherwise 
                                 :
                             
                             Not homologous   
                         
                     
                    
                 
                
                
                 
                     
                          Estimated full-length sequence:  
                         
                          ? 
                             
Click checkbox to select multiple seuqences.
Fasta header format:
  UID|accession.versionSTRAND start-end 
                         
                     
                     >uid:35|NW_021127469.1rc 58721-58906
AUCGCUUCUAGGCCUUUUGGCCAAGAUUUCUUUCUCGCUUUUUUGCAGAGAAGGCUAUUA
GAAGCGAUGGCAGAAGGAGAUGCAGAGAUGUUUGUCGAUGUUGCGGGGAAUCCAAGAGAA
GAUGUUAUUACUUUACCGAGUGCAGAAUGAACCAGUGAUGAUACUUUGAGCUGGGCAGAU
AGAGUA
 
                 
                
             
            
             
                
                     
                         
                         
                             rnafold 
                             
                              ? 
                                 
Visualisation of predicted secondary structure.
To save the image:
  Right click on the image -> Save Image as. 
                             
                         
                     
                
                     
                         
                         
                             rfam-Rc 
                             
                              ? 
                                 
Visualisation of predicted secondary structure.
To save the image:
  Right click on the image -> Save Image as. 
                             
                         
                     
                
                     
                         
                         
                             TurboFold 
                             
                              ? 
                                 
Visualisation of predicted secondary structure.
To save the image:
  Right click on the image -> Save Image as. 
                             
                         
                     
                
             
            

        
             
                 Load Sequence viewer 
             
        
        
         
     
    

    
     
         
            Hit: NW_021127469.1
         
         
             
                 
                     NW_021127469.1 Acropora millepora isolate SF001 unplaced genomic scaffold, amil_sf_1.1 amil.xfSc0000099, whole genome shotgun sequence 
                     
                         
 ?  
This is BLAST alignment as read from the input file 
                         
 Score = 42.0 bits (39.2), Expect = 9.49E-01
 Identities = 26/28 (93%), Gaps = 1/28 (4%)
 Strand = Plus/Minus
Query  11 GGCC-TTTTGGCTAAGATCAAGTGTAGT 37 
          |||| ||||||| |||||||||||||||    
Sbjct 264 GGCCATTTTGGCCAAGATCAAGTGTAGT 237

 
                 
                
                 
                      Report:  
                     
                         
                             sequence start
                                   ?  
                                     
Start position of the estimated full-length sequence in genome.
Start index  
                                 :
                             
                             86 
                         
                         
                             sequence end
                                   ?  
                                     
End position of the estimated full-length sequence in genome.
Start index  
                                 :
                             
                             275 
                         
                         
                             bit score (CM)
                                   ?  
                                     
The score for aligning estimated full-length sequence to CM model
  (computed by RSEARCH -> default,
  infered from Rfam or provided by user) 
                                 :
                             
                             -5.26 
                         
                         
                             Homology estimate
                                   ?  
                                     
Quick homology estimate:
  Not homologous: bit score   20 and bit score > 0.5 * query length
  Uncertain otherwise 
                                 :
                             
                             Not homologous   
                         
                     
                    
                 
                
                
                 
                     
                          Estimated full-length sequence:  
                         
                          ? 
                             
Click checkbox to select multiple seuqences.
Fasta header format:
  UID|accession.versionSTRAND start-end 
                         
                     
                     >uid:36|NW_021127469.1rc 86-275
AGCACCACCGGGCCAUUUUGGCCAAGAUCAAGUGUAGUUCUUGCAUCUUGCCAGUAAGAU
UUUUUUUACCGUCAAUUUUAUUUACCGUCCUGUGCUAAGUAACCGCCGCAGGGGAAUGCC
AAGGACAGCCCCUUCCCCAUGAAAAGUGAUUGUUUGCUCGUCCCAGUCCAAGCUGCGAUG
GCUGCAACUU
 
                 
                
             
            
             
                
                     
                         
                         
                             rnafold 
                             
                              ? 
                                 
Visualisation of predicted secondary structure.
To save the image:
  Right click on the image -> Save Image as. 
                             
                         
                     
                
                     
                         
                         
                             rfam-Rc 
                             
                              ? 
                                 
Visualisation of predicted secondary structure.
To save the image:
  Right click on the image -> Save Image as. 
                             
                         
                     
                
                     
                         
                         
                             TurboFold 
                             
                              ? 
                                 
Visualisation of predicted secondary structure.
To save the image:
  Right click on the image -> Save Image as. 
                             
                         
                     
                
             
            

        
             
                 Load Sequence viewer 
             
        
        
         
     
    

    
     
         
            Hit: NW_021127469.1
         
         
             
                 
                     NW_021127469.1 Acropora millepora isolate SF001 unplaced genomic scaffold, amil_sf_1.1 amil.xfSc0000099, whole genome shotgun sequence 
                     
                         
 ?  
This is BLAST alignment as read from the input file 
                         
 Score = 41.0 bits (38.3), Expect = 3.31E+00
 Identities = 25/28 (89%), Gaps = 0/28 (0%)
 Strand = Plus/Minus
Query     1 ATCGCTTCTCGGCCTTTTGGCTAAGATC 28   
            ||| |||| ||||||||||||| |||||      
Sbjct 33812 ATCACTTCGCGGCCTTTTGGCTTAGATC 33785

 
                 
                
                 
                      Report:  
                     
                         
                             sequence start
                                   ?  
                                     
Start position of the estimated full-length sequence in genome.
Start index  
                                 :
                             
                             33625 
                         
                         
                             sequence end
                                   ?  
                                     
End position of the estimated full-length sequence in genome.
Start index  
                                 :
                             
                             33815 
                         
                         
                             bit score (CM)
                                   ?  
                                     
The score for aligning estimated full-length sequence to CM model
  (computed by RSEARCH -> default,
  infered from Rfam or provided by user) 
                                 :
                             
                             -6.38 
                         
                         
                             Homology estimate
                                   ?  
                                     
Quick homology estimate:
  Not homologous: bit score   20 and bit score > 0.5 * query length
  Uncertain otherwise 
                                 :
                             
                             Not homologous   
                         
                     
                    
                 
                
                
                 
                     
                          Estimated full-length sequence:  
                         
                          ? 
                             
Click checkbox to select multiple seuqences.
Fasta header format:
  UID|accession.versionSTRAND start-end 
                         
                     
                     >uid:37|NW_021127469.1rc 33625-33815
AUCACUUCGCGGCCUUUUGGCUUAGAUCCAAAAGGUCCAGAUUGCUUUUUUUAUCUUCGC
AGGUUUUCACUAUGUCAAAUACAGCUAUGGAGAGCAUAGAAGUUACUCAAGAUGUGAAGA
AAAUAAAACAUGGAAUGAAACUGUGGAACAAGAAACUCCUUAGUGAAUCAUCCAGAUGAG
GAAUCUCAGAG
 
                 
                
             
            
             
                
                     
                         
                         
                             rnafold 
                             
                              ? 
                                 
Visualisation of predicted secondary structure.
To save the image:
  Right click on the image -> Save Image as. 
                             
                         
                     
                
                     
                         
                         
                             rfam-Rc 
                             
                              ? 
                                 
Visualisation of predicted secondary structure.
To save the image:
  Right click on the image -> Save Image as. 
                             
                         
                     
                
                     
                         
                         
                             TurboFold 
                             
                              ? 
                                 
Visualisation of predicted secondary structure.
To save the image:
  Right click on the image -> Save Image as. 
                             
                         
                     
                
             
            

        
             
                 Load Sequence viewer 
             
        
        
         
     
    

    
     
         
            Hit: NW_021127469.1
         
         
             
                 
                     NW_021127469.1 Acropora millepora isolate SF001 unplaced genomic scaffold, amil_sf_1.1 amil.xfSc0000099, whole genome shotgun sequence 
                     
                         
 ?  
This is BLAST alignment as read from the input file 
                         
 Score = 40.0 bits (37.4), Expect = 3.31E+00
 Identities = 23/25 (92%), Gaps = 0/25 (0%)
 Strand = Plus/Minus
Query     1 ATCGCTTCTCGGCCTTTTGGCTAAG 25   
            ||||||||||| ||||||| |||||      
Sbjct 40840 ATCGCTTCTCGTCCTTTTGACTAAG 40816

 
                 
                
                 
                      Report:  
                     
                         
                             sequence start
                                   ?  
                                     
Start position of the estimated full-length sequence in genome.
Start index  
                                 :
                             
                             40653 
                         
                         
                             sequence end
                                   ?  
                                     
End position of the estimated full-length sequence in genome.
Start index  
                                 :
                             
                             40838 
                         
                         
                             bit score (CM)
                                   ?  
                                     
The score for aligning estimated full-length sequence to CM model
  (computed by RSEARCH -> default,
  infered from Rfam or provided by user) 
                                 :
                             
                             -13.2 
                         
                         
                             Homology estimate
                                   ?  
                                     
Quick homology estimate:
  Not homologous: bit score   20 and bit score > 0.5 * query length
  Uncertain otherwise 
                                 :
                             
                             Not homologous   
                         
                     
                    
                 
                
                
                 
                     
                          Estimated full-length sequence:  
                         
                          ? 
                             
Click checkbox to select multiple seuqences.
Fasta header format:
  UID|accession.versionSTRAND start-end 
                         
                     
                     >uid:38|NW_021127469.1rc 40653-40838
AUCGCUUCUCGUCCUUUUGACUAAGGCUGUUUUUUUUCCUUUGCUCUUGCAGGAAAGUGU
UUCAAAGUGCUGGCAAGUGAUGAGGAAACUAGGAUAGGAGUUCCAGCCAGACCAGAUGCG
AAUAUUGUGAUUACCGUUCCAUUGUCCGAAGAUCGUACCAACAAUGCAUCCAGUUAGGCG
GAAAUA
 
                 
                
             
            
             
                
                     
                         
                         
                             rnafold 
                             
                              ? 
                                 
Visualisation of predicted secondary structure.
To save the image:
  Right click on the image -> Save Image as. 
                             
                         
                     
                
                     
                         
                         
                             rfam-Rc 
                             
                              ? 
                                 
Visualisation of predicted secondary structure.
To save the image:
  Right click on the image -> Save Image as. 
                             
                         
                     
                
                     
                         
                         
                             TurboFold 
                             
                              ? 
                                 
Visualisation of predicted secondary structure.
To save the image:
  Right click on the image -> Save Image as. 
                             
                         
                     
                
             
            

        
             
                 Load Sequence viewer 
             
        
        
         
     
    

    
     
         
            Hit: NW_021127557.1
         
         
             
                 
                     NW_021127557.1 Acropora millepora isolate SF001 unplaced genomic scaffold, amil_sf_1.1 amil.xfSc0000187, whole genome shotgun sequence 
                     
                         
 ?  
This is BLAST alignment as read from the input file 
                         
 Score = 164.0 bits (149.2), Expect = 7.28E-34
 Identities = 138/174 (79%), Gaps = 1/174 (1%)
 Strand = Plus/Minus
Query     1 ATCGCTTCTCGGCCTTTTGGCTAAGATCAAGTGTAGTATCTGTTCTTATCAGTTTAATATCTGATA 66   
            |||||||||||||||||||||||||||||||||||||||||||||||||||| |||||||||||||      
Sbjct 39388 ATCGCTTCTCGGCCTTTTGGCTAAGATCAAGTGTAGTATCTGTTCTTATCAGCTTAATATCTGATA 39323

Query    67 CG-TCCTCTATCCGAGGACAATATATTAAATGGATTTTTGGAGCAGGGAGATGGAATAGGAGCTTG 131  
            || | |||  |  |  |   ||||||||||  |||||||||| | |||   ||||| ||  |||||      
Sbjct 39322 CGCTGCTCATTGAGCAGCTCATATATTAAACTGATTTTTGGAACCGGGCTGTGGAAAAGAGGCTTG 39257

Query   132 CTCTGTCCACTCCACGCATCGACCTGGTATTGCAGTACCTCC 173  
            |   ||||   |||||  | | |  ||||| ||| |||||||      
Sbjct 39256 CCTCGTCCCAGCCACGGGTTGCCTCGGTATAGCACTACCTCC 39215

 
                 
                
                 
                      Report:  
                     
                         
                             sequence start
                                   ?  
                                     
Start position of the estimated full-length sequence in genome.
Start index  
                                 :
                             
                             39200 
                         
                         
                             sequence end
                                   ?  
                                     
End position of the estimated full-length sequence in genome.
Start index  
                                 :
                             
                             39388 
                         
                         
                             bit score (CM)
                                   ?  
                                     
The score for aligning estimated full-length sequence to CM model
  (computed by RSEARCH -> default,
  infered from Rfam or provided by user) 
                                 :
                             
                             161.93 
                         
                         
                             Homology estimate
                                   ?  
                                     
Quick homology estimate:
  Not homologous: bit score   20 and bit score > 0.5 * query length
  Uncertain otherwise 
                                 :
                             
                             Homologous   
                         
                     
                    
                 
                
                
                 
                     
                          Estimated full-length sequence:  
                         
                          ? 
                             
Click checkbox to select multiple seuqences.
Fasta header format:
  UID|accession.versionSTRAND start-end 
                         
                     
                     >uid:39|NW_021127557.1rc 39200-39388
AUCGCUUCUCGGCCUUUUGGCUAAGAUCAAGUGUAGUAUCUGUUCUUAUCAGCUUAAUAU
CUGAUACGCUGCUCAUUGAGCAGCUCAUAUAUUAAACUGAUUUUUGGAACCGGGCUGUGG
AAAAGAGGCUUGCCUCGUCCCAGCCACGGGUUGCCUCGGUAUAGCACUACCUCCGAGCGC
GGCCCACUU
 
                 
                
             
            
             
                
                     
                         
                         
                             rnafold 
                             
                              ? 
                                 
Visualisation of predicted secondary structure.
To save the image:
  Right click on the image -> Save Image as. 
                             
                         
                     
                
                     
                         
                         
                             rfam-Rc 
                             
                              ? 
                                 
Visualisation of predicted secondary structure.
To save the image:
  Right click on the image -> Save Image as. 
                             
                         
                     
                
                     
                         
                         
                             TurboFold 
                             
                              ? 
                                 
Visualisation of predicted secondary structure.
To save the image:
  Right click on the image -> Save Image as. 
                             
                         
                     
                
             
            

        
             
                 Load Sequence viewer 
             
        
        
         
     
    

    
     
         
            Hit: NW_021127557.1
         
         
             
                 
                     NW_021127557.1 Acropora millepora isolate SF001 unplaced genomic scaffold, amil_sf_1.1 amil.xfSc0000187, whole genome shotgun sequence 
                     
                         
 ?  
This is BLAST alignment as read from the input file 
                         
 Score = 164.0 bits (149.2), Expect = 7.28E-34
 Identities = 138/174 (79%), Gaps = 1/174 (1%)
 Strand = Plus/Minus
Query     1 ATCGCTTCTCGGCCTTTTGGCTAAGATCAAGTGTAGTATCTGTTCTTATCAGTTTAATATCTGATA 66   
            |||||||||||||||||||||||||||||||||||||||||||||||||||| |||||||||||||      
Sbjct 48137 ATCGCTTCTCGGCCTTTTGGCTAAGATCAAGTGTAGTATCTGTTCTTATCAGCTTAATATCTGATA 48072

Query    67 CG-TCCTCTATCCGAGGACAATATATTAAATGGATTTTTGGAGCAGGGAGATGGAATAGGAGCTTG 131  
            || | |||  |  |  |   ||||||||||  |||||||||| | |||   ||||| ||  |||||      
Sbjct 48071 CGCTGCTCATTGAGCAGCTCATATATTAAACTGATTTTTGGAACCGGGCTGTGGAAAAGAGGCTTG 48006

Query   132 CTCTGTCCACTCCACGCATCGACCTGGTATTGCAGTACCTCC 173  
            |   ||||   |||||  | | |  ||||| ||| |||||||      
Sbjct 48005 CCTCGTCCCAGCCACGGGTTGCCTCGGTATAGCACTACCTCC 47964

 
                 
                
                 
                      Report:  
                     
                         
                             sequence start
                                   ?  
                                     
Start position of the estimated full-length sequence in genome.
Start index  
                                 :
                             
                             47949 
                         
                         
                             sequence end
                                   ?  
                                     
End position of the estimated full-length sequence in genome.
Start index  
                                 :
                             
                             48137 
                         
                         
                             bit score (CM)
                                   ?  
                                     
The score for aligning estimated full-length sequence to CM model
  (computed by RSEARCH -> default,
  infered from Rfam or provided by user) 
                                 :
                             
                             161.93 
                         
                         
                             Homology estimate
                                   ?  
                                     
Quick homology estimate:
  Not homologous: bit score   20 and bit score > 0.5 * query length
  Uncertain otherwise 
                                 :
                             
                             Homologous   
                         
                     
                    
                 
                
                
                 
                     
                          Estimated full-length sequence:  
                         
                          ? 
                             
Click checkbox to select multiple seuqences.
Fasta header format:
  UID|accession.versionSTRAND start-end 
                         
                     
                     >uid:40|NW_021127557.1rc 47949-48137
AUCGCUUCUCGGCCUUUUGGCUAAGAUCAAGUGUAGUAUCUGUUCUUAUCAGCUUAAUAU
CUGAUACGCUGCUCAUUGAGCAGCUCAUAUAUUAAACUGAUUUUUGGAACCGGGCUGUGG
AAAAGAGGCUUGCCUCGUCCCAGCCACGGGUUGCCUCGGUAUAGCACUACCUCCGAGCGC
GGCCCACUU
 
                 
                
             
            
             
                
                     
                         
                         
                             rnafold 
                             
                              ? 
                                 
Visualisation of predicted secondary structure.
To save the image:
  Right click on the image -> Save Image as. 
                             
                         
                     
                
                     
                         
                         
                             rfam-Rc 
                             
                              ? 
                                 
Visualisation of predicted secondary structure.
To save the image:
  Right click on the image -> Save Image as. 
                             
                         
                     
                
                     
                         
                         
                             TurboFold 
                             
                              ? 
                                 
Visualisation of predicted secondary structure.
To save the image:
  Right click on the image -> Save Image as. 
                             
                         
                     
                
             
            

        
             
                 Load Sequence viewer 
             
        
        
         
     
    

    
     
         
            Hit: NW_021127557.1
         
         
             
                 
                     NW_021127557.1 Acropora millepora isolate SF001 unplaced genomic scaffold, amil_sf_1.1 amil.xfSc0000187, whole genome shotgun sequence 
                     
                         
 ?  
This is BLAST alignment as read from the input file 
                         
 Score = 68.0 bits (62.6), Expect = 8.31E-08
 Identities = 98/138 (71%), Gaps = 2/138 (1%)
 Strand = Plus/Minus
Query   38 ATCTGTTCTTATCAGTTTAATATCTGATACG-TCCTCTATCCGAGGACAATATATTAAATGGATTTTT 104 
           ||||||||||||||| ||||||||||||| | | |||  |  |  |   ||||||||||  |||||||     
Sbjct 3227 ATCTGTTCTTATCAGCTTAATATCTGATATGTTGCTCATTGAGCAGCTCATATATTAAACTGATTTTT 3160

Query  105 GG-AGCAGGGAGATGGAATAGGAGCTTGCTCTGTCCACTCCACGCATCGACCTGGTATTGCAGTACCT 171 
           || | ||||  |    || | | ||||||   ||||   |||||  | | |  ||||| ||| |||||     
Sbjct 3159 GGAACCAGGCTGTGAAAAAAAGGGCTTGCCTCGTCCCAGCCACGGGTTGCCTCGGTATAGCACTACCT 3092

Query  172 CC 173 
           ||     
Sbjct 3091 CC 3090

 
                 
                
                 
                      Report:  
                     
                         
                             sequence start
                                   ?  
                                     
Start position of the estimated full-length sequence in genome.
Start index  
                                 :
                             
                             3071 
                         
                         
                             sequence end
                                   ?  
                                     
End position of the estimated full-length sequence in genome.
Start index  
                                 :
                             
                             3264 
                         
                         
                             bit score (CM)
                                   ?  
                                     
The score for aligning estimated full-length sequence to CM model
  (computed by RSEARCH -> default,
  infered from Rfam or provided by user) 
                                 :
                             
                             71.69 
                         
                         
                             Homology estimate
                                   ?  
                                     
Quick homology estimate:
  Not homologous: bit score   20 and bit score > 0.5 * query length
  Uncertain otherwise 
                                 :
                             
                             Uncertain  ↴  
                         
                     
                    
                     
                        Check the secondary structure and sequence viewer
                        for supporting information about possible homology.
                     
                    
                 
                
                
                 
                     
                          Estimated full-length sequence:  
                         
                          ? 
                             
Click checkbox to select multiple seuqences.
Fasta header format:
  UID|accession.versionSTRAND start-end 
                         
                     
                     >uid:41|NW_021127557.1rc 3071-3264
UUAAUUGAGAAUAUGUGAAUAAAGUUUCAAAAUAAAAAAAAAUCUGUUCUUAUCAGCUUA
AUAUCUGAUAUGUUGCUCAUUGAGCAGCUCAUAUAUUAAACUGAUUUUUGGAACCAGGCU
GUGAAAAAAAGGGCUUGCCUCGUCCCAGCCACGGGUUGCCUCGGUAUAGCACUACCUCCG
AGCACGGCCCACUU
 
                 
                
             
            
             
                
                     
                         
                         
                             rnafold 
                             
                              ? 
                                 
Visualisation of predicted secondary structure.
To save the image:
  Right click on the image -> Save Image as. 
                             
                         
                     
                
                     
                         
                         
                             rfam-Rc 
                             
                              ? 
                                 
Visualisation of predicted secondary structure.
To save the image:
  Right click on the image -> Save Image as. 
                             
                         
                     
                
                     
                         
                         
                             TurboFold 
                             
                              ? 
                                 
Visualisation of predicted secondary structure.
To save the image:
  Right click on the image -> Save Image as. 
                             
                         
                     
                
             
            

        
             
                 Load Sequence viewer 
             
        
        
         
     
    

    
     
         
            Hit: NW_021127557.1
         
         
             
                 
                     NW_021127557.1 Acropora millepora isolate SF001 unplaced genomic scaffold, amil_sf_1.1 amil.xfSc0000187, whole genome shotgun sequence 
                     
                         
 ?  
This is BLAST alignment as read from the input file 
                         
 Score = 45.0 bits (41.9), Expect = 2.72E-01
 Identities = 24/25 (96%), Gaps = 0/25 (0%)
 Strand = Plus/Minus
Query     1 ATCGCTTCTCGGCCTTTTGGCTAAG 25   
            ||||||||||||||||||||| |||      
Sbjct 87314 ATCGCTTCTCGGCCTTTTGGCCAAG 87290

 
                 
                
                 
                      Report:  
                     
                         
                             sequence start
                                   ?  
                                     
Start position of the estimated full-length sequence in genome.
Start index  
                                 :
                             
                             87127 
                         
                         
                             sequence end
                                   ?  
                                     
End position of the estimated full-length sequence in genome.
Start index  
                                 :
                             
                             87315 
                         
                         
                             bit score (CM)
                                   ?  
                                     
The score for aligning estimated full-length sequence to CM model
  (computed by RSEARCH -> default,
  infered from Rfam or provided by user) 
                                 :
                             
                             -4.0 
                         
                         
                             Homology estimate
                                   ?  
                                     
Quick homology estimate:
  Not homologous: bit score   20 and bit score > 0.5 * query length
  Uncertain otherwise 
                                 :
                             
                             Not homologous   
                         
                     
                    
                 
                
                
                 
                     
                          Estimated full-length sequence:  
                         
                          ? 
                             
Click checkbox to select multiple seuqences.
Fasta header format:
  UID|accession.versionSTRAND start-end 
                         
                     
                     >uid:42|NW_021127557.1rc 87127-87315
AUCGCUUCUCGGCCUUUUGGCCAAGUCUUUUUUCUUUCUUUUUUGAGAAGAGAGCUAGAA
GCGAUGGAAGGUGCUGACCAUGACCAAUGGAUGGAUGUCCCCGAACGUCCUAGUGAAGAU
CCGAGAAUUCAAGUUCCGGUUGAUGGCGAACACACCAGUGACGAUCCUAAUAGUUGGGCA
GAAGUUGUU
 
                 
                
             
            
             
                
                     
                         
                         
                             rnafold 
                             
                              ? 
                                 
Visualisation of predicted secondary structure.
To save the image:
  Right click on the image -> Save Image as. 
                             
                         
                     
                
                     
                         
                         
                             rfam-Rc 
                             
                              ? 
                                 
Visualisation of predicted secondary structure.
To save the image:
  Right click on the image -> Save Image as. 
                             
                         
                     
                
                     
                         
                         
                             TurboFold 
                             
                              ? 
                                 
Visualisation of predicted secondary structure.
To save the image:
  Right click on the image -> Save Image as. 
                             
                         
                     
                
             
            

        
             
                 Load Sequence viewer 
             
        
        
         
     
    

    
     
         
            Hit: NW_019218324.1
         
         
             
                 
                     NW_019218324.1 Stylophora pistillata isolate CSM Monaco unplaced genomic scaffold, Stylophora pistillata v1 Spis.scaffold541, whole genome shotgun sequence 
                     
                         
 ?  
This is BLAST alignment as read from the input file 
                         
 Score = 164.0 bits (149.2), Expect = 7.28E-34
 Identities = 138/174 (79%), Gaps = 1/174 (1%)
 Strand = Plus/Minus
Query    1 ATCGCTTCTCGGCCTTTTGGCTAAGATCAAGTGTAGTATCTGTTCTTATCAGTTTAATATCTGATACG 68  
           |||||||||||||||||||||||||||||||||||||||||||||||||||| |||||||||||||||     
Sbjct 6279 ATCGCTTCTCGGCCTTTTGGCTAAGATCAAGTGTAGTATCTGTTCTTATCAGCTTAATATCTGATACG 6212

Query   69 -TCCTCTATCCGAGGACAATATATTAAATGGATTTTTGGAGCAGGGAGATGGAATAGGAGCTTGCTCT 135 
            | |||  |  |  |   ||||||||||  |||||||||| | |||   ||||| ||  ||||||        
Sbjct 6211 CTGCTCATTGAGTAGCTCATATATTAAACTGATTTTTGGAACTGGGCTGTGGAAAAGAGGCTTGCCTC 6144

Query  136 GTCCACTCCACGCATCGACCTGGTATTGCAGTACCTCC 173 
           ||||   |||||  | | |  ||||| ||| |||||||     
Sbjct 6143 GTCCCAGCCACGGGTTGCCTCGGTATAGCACTACCTCC 6106

 
                 
                
                 
                      Report:  
                     
                         
                             sequence start
                                   ?  
                                     
Start position of the estimated full-length sequence in genome.
Start index  
                                 :
                             
                             6091 
                         
                         
                             sequence end
                                   ?  
                                     
End position of the estimated full-length sequence in genome.
Start index  
                                 :
                             
                             6279 
                         
                         
                             bit score (CM)
                                   ?  
                                     
The score for aligning estimated full-length sequence to CM model
  (computed by RSEARCH -> default,
  infered from Rfam or provided by user) 
                                 :
                             
                             157.09 
                         
                         
                             Homology estimate
                                   ?  
                                     
Quick homology estimate:
  Not homologous: bit score   20 and bit score > 0.5 * query length
  Uncertain otherwise 
                                 :
                             
                             Homologous   
                         
                     
                    
                 
                
                
                 
                     
                          Estimated full-length sequence:  
                         
                          ? 
                             
Click checkbox to select multiple seuqences.
Fasta header format:
  UID|accession.versionSTRAND start-end 
                         
                     
                     >uid:43|NW_019218324.1rc 6091-6279
AUCGCUUCUCGGCCUUUUGGCUAAGAUCAAGUGUAGUAUCUGUUCUUAUCAGCUUAAUAU
CUGAUACGCUGCUCAUUGAGUAGCUCAUAUAUUAAACUGAUUUUUGGAACUGGGCUGUGG
AAAAGAGGCUUGCCUCGUCCCAGCCACGGGUUGCCUCGGUAUAGCACUACCUCCGAGUGU
GGCCCACUU
 
                 
                
             
            
             
                
                     
                         
                         
                             rnafold 
                             
                              ? 
                                 
Visualisation of predicted secondary structure.
To save the image:
  Right click on the image -> Save Image as. 
                             
                         
                     
                
                     
                         
                         
                             rfam-Rc 
                             
                              ? 
                                 
Visualisation of predicted secondary structure.
To save the image:
  Right click on the image -> Save Image as. 
                             
                         
                     
                
                     
                         
                         
                             TurboFold 
                             
                              ? 
                                 
Visualisation of predicted secondary structure.
To save the image:
  Right click on the image -> Save Image as. 
                             
                         
                     
                
             
            

        
             
                 Load Sequence viewer 
             
        
        
         
     
    

    
     
         
            Hit: NW_019218324.1
         
         
             
                 
                     NW_019218324.1 Stylophora pistillata isolate CSM Monaco unplaced genomic scaffold, Stylophora pistillata v1 Spis.scaffold541, whole genome shotgun sequence 
                     
                         
 ?  
This is BLAST alignment as read from the input file 
                         
 Score = 85.0 bits (77.9), Expect = 3.77E-12
 Identities = 100/137 (73%), Gaps = 1/137 (1%)
 Strand = Plus/Minus
Query   38 ATCTGTTCTTATCAGTTTAATATCTGATACG-TCCTCTATCCGAGGACAATATATTAAATGGATTTTT 104 
           |||| |||||||||| ||||||||||||||| | |||  |  |  |   ||||||||||  |||||||     
Sbjct 6834 ATCTTTTCTTATCAGCTTAATATCTGATACGCTGCTCATTGAGTAGCTCATATATTAAACTGATTTTT 6767

Query  105 GGAGCAGGGAGATGGAATAGGAGCTTGCTCTGTCCACTCCACGCATCGACCTGGTATTGCAGTACCTC 172 
           ||| | |||   ||||| ||  ||||||   ||||   |||||  | | |  ||||| ||| ||||||     
Sbjct 6766 GGAACTGGGCTGTGGAAAAGAGGCTTGCCTCGTCCCAGCCACGGGTTGCCTCGGTATAGCACTACCTC 6699

Query  173 C 173 
           |     
Sbjct 6698 C 6698

 
                 
                
                 
                      Report:  
                     
                         
                             sequence start
                                   ?  
                                     
Start position of the estimated full-length sequence in genome.
Start index  
                                 :
                             
                             6679 
                         
                         
                             sequence end
                                   ?  
                                     
End position of the estimated full-length sequence in genome.
Start index  
                                 :
                             
                             6871 
                         
                         
                             bit score (CM)
                                   ?  
                                     
The score for aligning estimated full-length sequence to CM model
  (computed by RSEARCH -> default,
  infered from Rfam or provided by user) 
                                 :
                             
                             93.83 
                         
                         
                             Homology estimate
                                   ?  
                                     
Quick homology estimate:
  Not homologous: bit score   20 and bit score > 0.5 * query length
  Uncertain otherwise 
                                 :
                             
                             Uncertain  ↴  
                         
                     
                    
                     
                        Check the secondary structure and sequence viewer
                        for supporting information about possible homology.
                     
                    
                 
                
                
                 
                     
                          Estimated full-length sequence:  
                         
                          ? 
                             
Click checkbox to select multiple seuqences.
Fasta header format:
  UID|accession.versionSTRAND start-end 
                         
                     
                     >uid:44|NW_019218324.1rc 6679-6871
CUAGGUGAUUUAUUGUAAAUAAAUCCUUUUCAAAACUUAAAAUCUUUUCUUAUCAGCUUA
AUAUCUGAUACGCUGCUCAUUGAGUAGCUCAUAUAUUAAACUGAUUUUUGGAACUGGGCU
GUGGAAAAGAGGCUUGCCUCGUCCCAGCCACGGGUUGCCUCGGUAUAGCACUACCUCCGA
GUGUGGCCCACUU
 
                 
                
             
            
             
                
                     
                         
                         
                             rnafold 
                             
                              ? 
                                 
Visualisation of predicted secondary structure.
To save the image:
  Right click on the image -> Save Image as. 
                             
                         
                     
                
                     
                         
                         
                             rfam-Rc 
                             
                              ? 
                                 
Visualisation of predicted secondary structure.
To save the image:
  Right click on the image -> Save Image as. 
                             
                         
                     
                
                     
                         
                         
                             TurboFold 
                             
                              ? 
                                 
Visualisation of predicted secondary structure.
To save the image:
  Right click on the image -> Save Image as. 
                             
                         
                     
                
             
            

        
             
                 Load Sequence viewer 
             
        
        
         
     
    

    
     
         
            Hit: NW_019218324.1
         
         
             
                 
                     NW_019218324.1 Stylophora pistillata isolate CSM Monaco unplaced genomic scaffold, Stylophora pistillata v1 Spis.scaffold541, whole genome shotgun sequence 
                     
                         
 ?  
This is BLAST alignment as read from the input file 
                         
 Score = 66.0 bits (60.8), Expect = 2.90E-07
 Identities = 33/33 (100%), Gaps = 0/33 (0%)
 Strand = Plus/Minus
Query    1 ATCGCTTCTCGGCCTTTTGGCTAAGATCAAGTG 33  
           |||||||||||||||||||||||||||||||||     
Sbjct 4198 ATCGCTTCTCGGCCTTTTGGCTAAGATCAAGTG 4166

 
                 
                
                 
                      Report:  
                     
                         
                             sequence start
                                   ?  
                                     
Start position of the estimated full-length sequence in genome.
Start index  
                                 :
                             
                             4011 
                         
                         
                             sequence end
                                   ?  
                                     
End position of the estimated full-length sequence in genome.
Start index  
                                 :
                             
                             4184 
                         
                         
                             bit score (CM)
                                   ?  
                                     
The score for aligning estimated full-length sequence to CM model
  (computed by RSEARCH -> default,
  infered from Rfam or provided by user) 
                                 :
                             
                             21.86 
                         
                         
                             Homology estimate
                                   ?  
                                     
Quick homology estimate:
  Not homologous: bit score   20 and bit score > 0.5 * query length
  Uncertain otherwise 
                                 :
                             
                             Uncertain  ↴  
                         
                     
                    
                     
                        Check the secondary structure and sequence viewer
                        for supporting information about possible homology.
                     
                    
                 
                
                
                 
                     
                          Estimated full-length sequence:  
                         
                          ? 
                             
Click checkbox to select multiple seuqences.
Fasta header format:
  UID|accession.versionSTRAND start-end 
                         
                     
                     >uid:45|NW_019218324.1rc 4011-4184
AUCGCUUCUCGGCCUUUUGGCUAAGAUCAAGUGCUAAACUUUGAUUGGUCAAAGAGUCAU
AUUAUCCCCAGUUCGCCUCCGACUCUGGACGCGCGGAUCUGGUAAGUGAAAUAUAUAAAU
UUUCAACACCAUGAUAACCGCUGUAGUUGACGAUGUAGAGAAAGGAGAUUACGG
 
                 
                
             
            
             
                
                     
                         
                         
                             rnafold 
                             
                              ? 
                                 
Visualisation of predicted secondary structure.
To save the image:
  Right click on the image -> Save Image as. 
                             
                         
                     
                
                     
                         
                         
                             rfam-Rc 
                             
                              ? 
                                 
Visualisation of predicted secondary structure.
To save the image:
  Right click on the image -> Save Image as. 
                             
                         
                     
                
                     
                         
                         
                             TurboFold 
                             
                              ? 
                                 
Visualisation of predicted secondary structure.
To save the image:
  Right click on the image -> Save Image as. 
                             
                         
                     
                
             
            

        
             
                 Load Sequence viewer 
             
        
        
         
     
    

    
     
         
            Hit: NW_019218324.1
         
         
             
                 
                     NW_019218324.1 Stylophora pistillata isolate CSM Monaco unplaced genomic scaffold, Stylophora pistillata v1 Spis.scaffold541, whole genome shotgun sequence 
                     
                         
 ?  
This is BLAST alignment as read from the input file 
                         
 Score = 49.0 bits (45.5), Expect = 2.23E-02
 Identities = 28/29 (97%), Gaps = 1/29 (3%)
 Strand = Plus/Minus
Query     1 ATCGCTTCTCGGCCTTTTGGCTAAGATCA 29   
            |||||||||||||||||||||| ||||||      
Sbjct 20536 ATCGCTTCTCGGCCTTTTGGCT-AGATCA 20509

 
                 
                
                 
                      Report:  
                     
                         
                             sequence start
                                   ?  
                                     
Start position of the estimated full-length sequence in genome.
Start index  
                                 :
                             
                             20350 
                         
                         
                             sequence end
                                   ?  
                                     
End position of the estimated full-length sequence in genome.
Start index  
                                 :
                             
                             20534 
                         
                         
                             bit score (CM)
                                   ?  
                                     
The score for aligning estimated full-length sequence to CM model
  (computed by RSEARCH -> default,
  infered from Rfam or provided by user) 
                                 :
                             
                             2.14 
                         
                         
                             Homology estimate
                                   ?  
                                     
Quick homology estimate:
  Not homologous: bit score   20 and bit score > 0.5 * query length
  Uncertain otherwise 
                                 :
                             
                             Uncertain  ↴  
                         
                     
                    
                     
                        Check the secondary structure and sequence viewer
                        for supporting information about possible homology.
                     
                    
                 
                
                
                 
                     
                          Estimated full-length sequence:  
                         
                          ? 
                             
Click checkbox to select multiple seuqences.
Fasta header format:
  UID|accession.versionSTRAND start-end 
                         
                     
                     >uid:46|NW_019218324.1rc 20350-20534
AUCGCUUCUCGGCCUUUUGGCUAGAUCAGACGUACAUUUUUGUGCUCCGUCUAGCCACUG
UUUAAUUCGAAAUUUGAGAAAGCUUUUUGCCCCGUGGGGCUCUUCCUUUUUUAUUGGGAA
GCCUUUUGAUUAUGGAUCUUAACGAAACGACAGGAAUUAUUGAGGAUAUUUCGACUGAAA
GUGAU
 
                 
                
             
            
             
                
                     
                         
                         
                             rnafold 
                             
                              ? 
                                 
Visualisation of predicted secondary structure.
To save the image:
  Right click on the image -> Save Image as. 
                             
                         
                     
                
                     
                         
                         
                             rfam-Rc 
                             
                              ? 
                                 
Visualisation of predicted secondary structure.
To save the image:
  Right click on the image -> Save Image as. 
                             
                         
                     
                
                     
                         
                         
                             TurboFold 
                             
                              ? 
                                 
Visualisation of predicted secondary structure.
To save the image:
  Right click on the image -> Save Image as. 
                             
                         
                     
                
             
            

        
             
                 Load Sequence viewer 
             
        
        
         
     
    

    
     
         
            Hit: NW_019218324.1
         
         
             
                 
                     NW_019218324.1 Stylophora pistillata isolate CSM Monaco unplaced genomic scaffold, Stylophora pistillata v1 Spis.scaffold541, whole genome shotgun sequence 
                     
                         
 ?  
This is BLAST alignment as read from the input file 
                         
 Score = 42.0 bits (39.2), Expect = 9.49E-01
 Identities = 26/28 (93%), Gaps = 1/28 (4%)
 Strand = Plus/Minus
Query     1 ATCGCTTCTCGGCCTTTTGGCTAAGATC 28   
            ||||||||| |||||||||||| |||||      
Sbjct 14548 ATCGCTTCTTGGCCTTTTGGCT-AGATC 14522

 
                 
                
                 
                      Report:  
                     
                         
                             sequence start
                                   ?  
                                     
Start position of the estimated full-length sequence in genome.
Start index  
                                 :
                             
                             14362 
                         
                         
                             sequence end
                                   ?  
                                     
End position of the estimated full-length sequence in genome.
Start index  
                                 :
                             
                             14558 
                         
                         
                             bit score (CM)
                                   ?  
                                     
The score for aligning estimated full-length sequence to CM model
  (computed by RSEARCH -> default,
  infered from Rfam or provided by user) 
                                 :
                             
                             -0.26 
                         
                         
                             Homology estimate
                                   ?  
                                     
Quick homology estimate:
  Not homologous: bit score   20 and bit score > 0.5 * query length
  Uncertain otherwise 
                                 :
                             
                             Not homologous   
                         
                     
                    
                 
                
                
                 
                     
                          Estimated full-length sequence:  
                         
                          ? 
                             
Click checkbox to select multiple seuqences.
Fasta header format:
  UID|accession.versionSTRAND start-end 
                         
                     
                     >uid:47|NW_019218324.1rc 14362-14558
AUCGCUUCUUGGCCUUUUGGCUAGAUCGGACGUGUUUUGCAUCGCUCCCUUUUAGUCAAG
UCACCACACAAAGUUUACGGUUAAUUGAGGUCGGGUUGCCCUUUUGGGCCUUAUUUUCAU
UCUAUUUGAUUGCAUUAGAUUAUGGAUUUUACGAUGAAUGUGGAUGGUUUAGAAGAAAUG
GUCGCUCAAGAGAAUUU
 
                 
                
             
            
             
                
                     
                         
                         
                             rnafold 
                             
                              ? 
                                 
Visualisation of predicted secondary structure.
To save the image:
  Right click on the image -> Save Image as. 
                             
                         
                     
                
                     
                         
                         
                             rfam-Rc 
                             
                              ? 
                                 
Visualisation of predicted secondary structure.
To save the image:
  Right click on the image -> Save Image as. 
                             
                         
                     
                
                     
                         
                         
                             TurboFold 
                             
                              ? 
                                 
Visualisation of predicted secondary structure.
To save the image:
  Right click on the image -> Save Image as. 
                             
                         
                     
                
             
            

        
             
                 Load Sequence viewer 
             
        
        
         
     
    

    
     
         
            Hit: NW_015441080.1
         
         
             
                 
                     NW_015441080.1 Acropora digitifera unplaced genomic scaffold, Adig_1.1 DF970715.1, whole genome shotgun sequence 
                     
                         
 ?  
This is BLAST alignment as read from the input file 
                         
 Score = 164.0 bits (149.2), Expect = 7.28E-34
 Identities = 138/174 (79%), Gaps = 1/174 (1%)
 Strand = Plus/Minus
Query      1 ATCGCTTCTCGGCCTTTTGGCTAAGATCAAGTGTAGTATCTGTTCTTATCAGTTTAATATCTGA 64    
             |||||||||||||||||||||||||||||||||||||||||||||||||||| |||||||||||       
Sbjct 610775 ATCGCTTCTCGGCCTTTTGGCTAAGATCAAGTGTAGTATCTGTTCTTATCAGCTTAATATCTGA 610712

Query     65 TACG-TCCTCTATCCGAGGACAATATATTAAATGGATTTTTGGAGCAGGGAGATGGAATAGGAG 127   
             |||| | |||  |  |  |   ||||||||||  |||||||||| | |||   ||||| ||  |       
Sbjct 610711 TACGCTGCTCATTGAGCAGCTCATATATTAAACTGATTTTTGGAACCGGGCTGTGGAAAAGAGG 610648

Query    128 CTTGCTCTGTCCACTCCACGCATCGACCTGGTATTGCAGTACCTCC 173   
             |||||   ||||   |||||  | | |  ||||| ||| |||||||       
Sbjct 610647 CTTGCCTCGTCCCAGCCACGGGTTGCCTCGGTATAGCACTACCTCC 610602

 
                 
                
                 
                      Report:  
                     
                         
                             sequence start
                                   ?  
                                     
Start position of the estimated full-length sequence in genome.
Start index  
                                 :
                             
                             610587 
                         
                         
                             sequence end
                                   ?  
                                     
End position of the estimated full-length sequence in genome.
Start index  
                                 :
                             
                             610775 
                         
                         
                             bit score (CM)
                                   ?  
                                     
The score for aligning estimated full-length sequence to CM model
  (computed by RSEARCH -> default,
  infered from Rfam or provided by user) 
                                 :
                             
                             161.93 
                         
                         
                             Homology estimate
                                   ?  
                                     
Quick homology estimate:
  Not homologous: bit score   20 and bit score > 0.5 * query length
  Uncertain otherwise 
                                 :
                             
                             Homologous   
                         
                     
                    
                 
                
                
                 
                     
                          Estimated full-length sequence:  
                         
                          ? 
                             
Click checkbox to select multiple seuqences.
Fasta header format:
  UID|accession.versionSTRAND start-end 
                         
                     
                     >uid:48|NW_015441080.1rc 610587-610775
AUCGCUUCUCGGCCUUUUGGCUAAGAUCAAGUGUAGUAUCUGUUCUUAUCAGCUUAAUAU
CUGAUACGCUGCUCAUUGAGCAGCUCAUAUAUUAAACUGAUUUUUGGAACCGGGCUGUGG
AAAAGAGGCUUGCCUCGUCCCAGCCACGGGUUGCCUCGGUAUAGCACUACCUCCGAGCGC
GGCCCACUU
 
                 
                
             
            
             
                
                     
                         
                         
                             rnafold 
                             
                              ? 
                                 
Visualisation of predicted secondary structure.
To save the image:
  Right click on the image -> Save Image as. 
                             
                         
                     
                
                     
                         
                         
                             rfam-Rc 
                             
                              ? 
                                 
Visualisation of predicted secondary structure.
To save the image:
  Right click on the image -> Save Image as. 
                             
                         
                     
                
                     
                         
                         
                             TurboFold 
                             
                              ? 
                                 
Visualisation of predicted secondary structure.
To save the image:
  Right click on the image -> Save Image as. 
                             
                         
                     
                
             
            

        
             
                 Load Sequence viewer 
             
        
        
         
     
    

    
     
         
            Hit: NW_015441080.1
         
         
             
                 
                     NW_015441080.1 Acropora digitifera unplaced genomic scaffold, Adig_1.1 DF970715.1, whole genome shotgun sequence 
                     
                         
 ?  
This is BLAST alignment as read from the input file 
                         
 Score = 164.0 bits (149.2), Expect = 7.28E-34
 Identities = 138/174 (79%), Gaps = 1/174 (1%)
 Strand = Plus/Minus
Query      1 ATCGCTTCTCGGCCTTTTGGCTAAGATCAAGTGTAGTATCTGTTCTTATCAGTTTAATATCTGA 64    
             |||||||||||||||||||||||||||||||||||||||||||||||||||| |||||||||||       
Sbjct 612575 ATCGCTTCTCGGCCTTTTGGCTAAGATCAAGTGTAGTATCTGTTCTTATCAGCTTAATATCTGA 612512

Query     65 TACG-TCCTCTATCCGAGGACAATATATTAAATGGATTTTTGGAGCAGGGAGATGGAATAGGAG 127   
             |||| | |||  |  |  |   ||||||||||  |||||||||| | |||   ||||| ||  |       
Sbjct 612511 TACGCTGCTCATTGAGCAGCTCATATATTAAACTGATTTTTGGAACCGGGCTGTGGAAAAGAGG 612448

Query    128 CTTGCTCTGTCCACTCCACGCATCGACCTGGTATTGCAGTACCTCC 173   
             |||||   ||||   |||||  | | |  ||||| ||| |||||||       
Sbjct 612447 CTTGCCTCGTCCCAGCCACGGGTTGCCTCGGTATAGCACTACCTCC 612402

 
                 
                
                 
                      Report:  
                     
                         
                             sequence start
                                   ?  
                                     
Start position of the estimated full-length sequence in genome.
Start index  
                                 :
                             
                             612387 
                         
                         
                             sequence end
                                   ?  
                                     
End position of the estimated full-length sequence in genome.
Start index  
                                 :
                             
                             612575 
                         
                         
                             bit score (CM)
                                   ?  
                                     
The score for aligning estimated full-length sequence to CM model
  (computed by RSEARCH -> default,
  infered from Rfam or provided by user) 
                                 :
                             
                             161.93 
                         
                         
                             Homology estimate
                                   ?  
                                     
Quick homology estimate:
  Not homologous: bit score   20 and bit score > 0.5 * query length
  Uncertain otherwise 
                                 :
                             
                             Homologous   
                         
                     
                    
                 
                
                
                 
                     
                          Estimated full-length sequence:  
                         
                          ? 
                             
Click checkbox to select multiple seuqences.
Fasta header format:
  UID|accession.versionSTRAND start-end 
                         
                     
                     >uid:49|NW_015441080.1rc 612387-612575
AUCGCUUCUCGGCCUUUUGGCUAAGAUCAAGUGUAGUAUCUGUUCUUAUCAGCUUAAUAU
CUGAUACGCUGCUCAUUGAGCAGCUCAUAUAUUAAACUGAUUUUUGGAACCGGGCUGUGG
AAAAGAGGCUUGCCUCGUCCCAGCCACGGGUUGCCUCGGUAUAGCACUACCUCCGAGCGC
GGCCCACUU
 
                 
                
             
            
             
                
                     
                         
                         
                             rnafold 
                             
                              ? 
                                 
Visualisation of predicted secondary structure.
To save the image:
  Right click on the image -> Save Image as. 
                             
                         
                     
                
                     
                         
                         
                             rfam-Rc 
                             
                              ? 
                                 
Visualisation of predicted secondary structure.
To save the image:
  Right click on the image -> Save Image as. 
                             
                         
                     
                
                     
                         
                         
                             TurboFold 
                             
                              ? 
                                 
Visualisation of predicted secondary structure.
To save the image:
  Right click on the image -> Save Image as. 
                             
                         
                     
                
             
            

        
             
                 Load Sequence viewer 
             
        
        
         
     
    

    
     
         
            Hit: NW_015441080.1
         
         
             
                 
                     NW_015441080.1 Acropora digitifera unplaced genomic scaffold, Adig_1.1 DF970715.1, whole genome shotgun sequence 
                     
                         
 ?  
This is BLAST alignment as read from the input file 
                         
 Score = 164.0 bits (149.2), Expect = 7.28E-34
 Identities = 138/174 (79%), Gaps = 1/174 (1%)
 Strand = Plus/Minus
Query      1 ATCGCTTCTCGGCCTTTTGGCTAAGATCAAGTGTAGTATCTGTTCTTATCAGTTTAATATCTGA 64    
             |||||||||||||||||||||||||||||||||||||||||||||||||||| |||||||||||       
Sbjct 649451 ATCGCTTCTCGGCCTTTTGGCTAAGATCAAGTGTAGTATCTGTTCTTATCAGCTTAATATCTGA 649388

Query     65 TACG-TCCTCTATCCGAGGACAATATATTAAATGGATTTTTGGAGCAGGGAGATGGAATAGGAG 127   
             |||| | |||  |  |  |   ||||||||||  |||||||||| | |||   ||||| ||  |       
Sbjct 649387 TACGCTGCTCATTGAGCAGCTCATATATTAAACTGATTTTTGGAACCGGGCTGTGGAAAAGAGG 649324

Query    128 CTTGCTCTGTCCACTCCACGCATCGACCTGGTATTGCAGTACCTCC 173   
             |||||   ||||   |||||  | | |  ||||| ||| |||||||       
Sbjct 649323 CTTGCCTCGTCCCAGCCACGGGTTGCCTCGGTATAGCACTACCTCC 649278

 
                 
                
                 
                      Report:  
                     
                         
                             sequence start
                                   ?  
                                     
Start position of the estimated full-length sequence in genome.
Start index  
                                 :
                             
                             649263 
                         
                         
                             sequence end
                                   ?  
                                     
End position of the estimated full-length sequence in genome.
Start index  
                                 :
                             
                             649451 
                         
                         
                             bit score (CM)
                                   ?  
                                     
The score for aligning estimated full-length sequence to CM model
  (computed by RSEARCH -> default,
  infered from Rfam or provided by user) 
                                 :
                             
                             161.93 
                         
                         
                             Homology estimate
                                   ?  
                                     
Quick homology estimate:
  Not homologous: bit score   20 and bit score > 0.5 * query length
  Uncertain otherwise 
                                 :
                             
                             Homologous   
                         
                     
                    
                 
                
                
                 
                     
                          Estimated full-length sequence:  
                         
                          ? 
                             
Click checkbox to select multiple seuqences.
Fasta header format:
  UID|accession.versionSTRAND start-end 
                         
                     
                     >uid:50|NW_015441080.1rc 649263-649451
AUCGCUUCUCGGCCUUUUGGCUAAGAUCAAGUGUAGUAUCUGUUCUUAUCAGCUUAAUAU
CUGAUACGCUGCUCAUUGAGCAGCUCAUAUAUUAAACUGAUUUUUGGAACCGGGCUGUGG
AAAAGAGGCUUGCCUCGUCCCAGCCACGGGUUGCCUCGGUAUAGCACUACCUCCGAGCGC
GGCCCACUU
 
                 
                
             
            
             
                
                     
                         
                         
                             rnafold 
                             
                              ? 
                                 
Visualisation of predicted secondary structure.
To save the image:
  Right click on the image -> Save Image as. 
                             
                         
                     
                
                     
                         
                         
                             rfam-Rc 
                             
                              ? 
                                 
Visualisation of predicted secondary structure.
To save the image:
  Right click on the image -> Save Image as. 
                             
                         
                     
                
                     
                         
                         
                             TurboFold 
                             
                              ? 
                                 
Visualisation of predicted secondary structure.
To save the image:
  Right click on the image -> Save Image as. 
                             
                         
                     
                
             
            

        
             
                 Load Sequence viewer 
             
        
        
         
     
    

    
     
         
            Hit: NW_015441080.1
         
         
             
                 
                     NW_015441080.1 Acropora digitifera unplaced genomic scaffold, Adig_1.1 DF970715.1, whole genome shotgun sequence 
                     
                         
 ?  
This is BLAST alignment as read from the input file 
                         
 Score = 164.0 bits (149.2), Expect = 7.28E-34
 Identities = 138/174 (79%), Gaps = 1/174 (1%)
 Strand = Plus/Minus
Query      1 ATCGCTTCTCGGCCTTTTGGCTAAGATCAAGTGTAGTATCTGTTCTTATCAGTTTAATATCTGA 64    
             |||||||||||||||||||||||||||||||||||||||||||||||||||| |||||||||||       
Sbjct 681540 ATCGCTTCTCGGCCTTTTGGCTAAGATCAAGTGTAGTATCTGTTCTTATCAGCTTAATATCTGA 681477

Query     65 TACG-TCCTCTATCCGAGGACAATATATTAAATGGATTTTTGGAGCAGGGAGATGGAATAGGAG 127   
             |||| | |||  |  |  |   ||||||||||  |||||||||| | |||   ||||| ||  |       
Sbjct 681476 TACGCTGCTCATTGAGCAGCTCATATATTAAACTGATTTTTGGAACCGGGCTGTGGAAAAGAGG 681413

Query    128 CTTGCTCTGTCCACTCCACGCATCGACCTGGTATTGCAGTACCTCC 173   
             |||||   ||||   |||||  | | |  ||||| ||| |||||||       
Sbjct 681412 CTTGCCTCGTCCCAGCCACGGGTTGCCTCGGTATAGCACTACCTCC 681367

 
                 
                
                 
                      Report:  
                     
                         
                             sequence start
                                   ?  
                                     
Start position of the estimated full-length sequence in genome.
Start index  
                                 :
                             
                             681352 
                         
                         
                             sequence end
                                   ?  
                                     
End position of the estimated full-length sequence in genome.
Start index  
                                 :
                             
                             681540 
                         
                         
                             bit score (CM)
                                   ?  
                                     
The score for aligning estimated full-length sequence to CM model
  (computed by RSEARCH -> default,
  infered from Rfam or provided by user) 
                                 :
                             
                             161.93 
                         
                         
                             Homology estimate
                                   ?  
                                     
Quick homology estimate:
  Not homologous: bit score   20 and bit score > 0.5 * query length
  Uncertain otherwise 
                                 :
                             
                             Homologous   
                         
                     
                    
                 
                
                
                 
                     
                          Estimated full-length sequence:  
                         
                          ? 
                             
Click checkbox to select multiple seuqences.
Fasta header format:
  UID|accession.versionSTRAND start-end 
                         
                     
                     >uid:51|NW_015441080.1rc 681352-681540
AUCGCUUCUCGGCCUUUUGGCUAAGAUCAAGUGUAGUAUCUGUUCUUAUCAGCUUAAUAU
CUGAUACGCUGCUCAUUGAGCAGCUCAUAUAUUAAACUGAUUUUUGGAACCGGGCUGUGG
AAAAGAGGCUUGCCUCGUCCCAGCCACGGGUUGCCUCGGUAUAGCACUACCUCCGAGCGC
GGCCCACUU
 
                 
                
             
            
             
                
                     
                         
                         
                             rnafold 
                             
                              ? 
                                 
Visualisation of predicted secondary structure.
To save the image:
  Right click on the image -> Save Image as. 
                             
                         
                     
                
                     
                         
                         
                             rfam-Rc 
                             
                              ? 
                                 
Visualisation of predicted secondary structure.
To save the image:
  Right click on the image -> Save Image as. 
                             
                         
                     
                
                     
                         
                         
                             TurboFold 
                             
                              ? 
                                 
Visualisation of predicted secondary structure.
To save the image:
  Right click on the image -> Save Image as. 
                             
                         
                     
                
             
            

        
             
                 Load Sequence viewer 
             
        
        
         
     
    

    
     
         
            Hit: NW_015441080.1
         
         
             
                 
                     NW_015441080.1 Acropora digitifera unplaced genomic scaffold, Adig_1.1 DF970715.1, whole genome shotgun sequence 
                     
                         
 ?  
This is BLAST alignment as read from the input file 
                         
 Score = 164.0 bits (149.2), Expect = 7.28E-34
 Identities = 138/174 (79%), Gaps = 1/174 (1%)
 Strand = Plus/Minus
Query      1 ATCGCTTCTCGGCCTTTTGGCTAAGATCAAGTGTAGTATCTGTTCTTATCAGTTTAATATCTGA 64    
             |||||||||||||||||||||||||||||||||||||||||||||||||||| |||||||||||       
Sbjct 752278 ATCGCTTCTCGGCCTTTTGGCTAAGATCAAGTGTAGTATCTGTTCTTATCAGCTTAATATCTGA 752215

Query     65 TACG-TCCTCTATCCGAGGACAATATATTAAATGGATTTTTGGAGCAGGGAGATGGAATAGGAG 127   
             |||| | |||  |  |  |   ||||||||||  |||||||||| | |||   ||||| ||  |       
Sbjct 752214 TACGCTGCTCATTGAGCAGCTCATATATTAAACTGATTTTTGGAACCGGGCTGTGGAAAAGAGG 752151

Query    128 CTTGCTCTGTCCACTCCACGCATCGACCTGGTATTGCAGTACCTCC 173   
             |||||   ||||   |||||  | | |  ||||| ||| |||||||       
Sbjct 752150 CTTGCCTCGTCCCAGCCACGGGTTGCCTCGGTATAGCACTACCTCC 752105

 
                 
                
                 
                      Report:  
                     
                         
                             sequence start
                                   ?  
                                     
Start position of the estimated full-length sequence in genome.
Start index  
                                 :
                             
                             752090 
                         
                         
                             sequence end
                                   ?  
                                     
End position of the estimated full-length sequence in genome.
Start index  
                                 :
                             
                             752278 
                         
                         
                             bit score (CM)
                                   ?  
                                     
The score for aligning estimated full-length sequence to CM model
  (computed by RSEARCH -> default,
  infered from Rfam or provided by user) 
                                 :
                             
                             161.93 
                         
                         
                             Homology estimate
                                   ?  
                                     
Quick homology estimate:
  Not homologous: bit score   20 and bit score > 0.5 * query length
  Uncertain otherwise 
                                 :
                             
                             Homologous   
                         
                     
                    
                 
                
                
                 
                     
                          Estimated full-length sequence:  
                         
                          ? 
                             
Click checkbox to select multiple seuqences.
Fasta header format:
  UID|accession.versionSTRAND start-end 
                         
                     
                     >uid:52|NW_015441080.1rc 752090-752278
AUCGCUUCUCGGCCUUUUGGCUAAGAUCAAGUGUAGUAUCUGUUCUUAUCAGCUUAAUAU
CUGAUACGCUGCUCAUUGAGCAGCUCAUAUAUUAAACUGAUUUUUGGAACCGGGCUGUGG
AAAAGAGGCUUGCCUCGUCCCAGCCACGGGUUGCCUCGGUAUAGCACUACCUCCGAGCGC
GGCCCACUU
 
                 
                
             
            
             
                
                     
                         
                         
                             rnafold 
                             
                              ? 
                                 
Visualisation of predicted secondary structure.
To save the image:
  Right click on the image -> Save Image as. 
                             
                         
                     
                
                     
                         
                         
                             rfam-Rc 
                             
                              ? 
                                 
Visualisation of predicted secondary structure.
To save the image:
  Right click on the image -> Save Image as. 
                             
                         
                     
                
                     
                         
                         
                             TurboFold 
                             
                              ? 
                                 
Visualisation of predicted secondary structure.
To save the image:
  Right click on the image -> Save Image as. 
                             
                         
                     
                
             
            

        
             
                 Load Sequence viewer 
             
        
        
         
     
    

    
     
         
            Hit: NW_015441080.1
         
         
             
                 
                     NW_015441080.1 Acropora digitifera unplaced genomic scaffold, Adig_1.1 DF970715.1, whole genome shotgun sequence 
                     
                         
 ?  
This is BLAST alignment as read from the input file 
                         
 Score = 149.0 bits (135.6), Expect = 1.60E-29
 Identities = 135/174 (78%), Gaps = 1/174 (1%)
 Strand = Plus/Minus
Query      1 ATCGCTTCTCGGCCTTTTGGCTAAGATCAAGTGTAGTATCTGTTCTTATCAGTTTAATATCTGA 64    
             |||||||||||||||||||||||||||||||||||||||||||||||||||| |||||||||||       
Sbjct 633508 ATCGCTTCTCGGCCTTTTGGCTAAGATCAAGTGTAGTATCTGTTCTTATCAGCTTAATATCTGA 633445

Query     65 TACG-TCCTCTATCCGAGGACAATATATTAAATGGATTTTTGGAGCAGGGAGATGGAATAGGAG 127   
             |||| | |||  |  |  |   ||||||||||  |||||||||| | |||   ||||| |   |       
Sbjct 633444 TACGCTGCTCATTGAGCAGCTCATATATTAAACTGATTTTTGGAACCGGGCTGTGGAAAATAGG 633381

Query    128 CTTGCTCTGTCCACTCCACGCATCGACCTGGTATTGCAGTACCTCC 173   
             |||||   ||||    ||||  | | |  ||||| ||| ||| |||       
Sbjct 633380 CTTGCCTCGTCCCAGTCACGGGTTGCCTCGGTATAGCACTACTTCC 633335

 
                 
                
                 
                      Report:  
                     
                         
                             sequence start
                                   ?  
                                     
Start position of the estimated full-length sequence in genome.
Start index  
                                 :
                             
                             633320 
                         
                         
                             sequence end
                                   ?  
                                     
End position of the estimated full-length sequence in genome.
Start index  
                                 :
                             
                             633508 
                         
                         
                             bit score (CM)
                                   ?  
                                     
The score for aligning estimated full-length sequence to CM model
  (computed by RSEARCH -> default,
  infered from Rfam or provided by user) 
                                 :
                             
                             150.0 
                         
                         
                             Homology estimate
                                   ?  
                                     
Quick homology estimate:
  Not homologous: bit score   20 and bit score > 0.5 * query length
  Uncertain otherwise 
                                 :
                             
                             Homologous   
                         
                     
                    
                 
                
                
                 
                     
                          Estimated full-length sequence:  
                         
                          ? 
                             
Click checkbox to select multiple seuqences.
Fasta header format:
  UID|accession.versionSTRAND start-end 
                         
                     
                     >uid:53|NW_015441080.1rc 633320-633508
AUCGCUUCUCGGCCUUUUGGCUAAGAUCAAGUGUAGUAUCUGUUCUUAUCAGCUUAAUAU
CUGAUACGCUGCUCAUUGAGCAGCUCAUAUAUUAAACUGAUUUUUGGAACCGGGCUGUGG
AAAAUAGGCUUGCCUCGUCCCAGUCACGGGUUGCCUCGGUAUAGCACUACUUCCGAGCGC
GGCCCACUU
 
                 
                
             
            
             
                
                     
                         
                         
                             rnafold 
                             
                              ? 
                                 
Visualisation of predicted secondary structure.
To save the image:
  Right click on the image -> Save Image as. 
                             
                         
                     
                
                     
                         
                         
                             rfam-Rc 
                             
                              ? 
                                 
Visualisation of predicted secondary structure.
To save the image:
  Right click on the image -> Save Image as. 
                             
                         
                     
                
                     
                         
                         
                             TurboFold 
                             
                              ? 
                                 
Visualisation of predicted secondary structure.
To save the image:
  Right click on the image -> Save Image as. 
                             
                         
                     
                
             
            

        
             
                 Load Sequence viewer 
             
        
        
         
     
    

    
     
         
            Hit: NW_015441080.1
         
         
             
                 
                     NW_015441080.1 Acropora digitifera unplaced genomic scaffold, Adig_1.1 DF970715.1, whole genome shotgun sequence 
                     
                         
 ?  
This is BLAST alignment as read from the input file 
                         
 Score = 90.0 bits (82.4), Expect = 8.88E-14
 Identities = 101/137 (74%), Gaps = 1/137 (1%)
 Strand = Plus/Minus
Query     38 ATCTGTTCTTATCAGTTTAATATCTGATACG-TCCTCTATCCGAGGACAATATATTAAATGGAT 100   
             ||||||||||||||| ||||||||||||||| | |||  |  |  |   ||||||||||  |||       
Sbjct 655556 ATCTGTTCTTATCAGCTTAATATCTGATACGCTGCTCATTGAGCAGCTCATATATTAAACTGAT 655493

Query    101 TTTTGGAGCAGGGAGATGGAATAGGAGCTTGCTCTGTCCACTCCACGCATCGACCTGGTATTGC 164   
             ||||||| | |||   ||||| ||  ||||||   ||||   |||||  | | |  ||||| ||       
Sbjct 655492 TTTTGGAACCGGGCTGTGGAAAAGAGGCTTGCCTCGTCCCAGCCACGGGTTGCCTCGGTATAGC 655429

Query    165 AGTACCTCC 173   
             | |||||||       
Sbjct 655428 ACTACCTCC 655420

 
                 
                
                 
                      Report:  
                     
                         
                             sequence start
                                   ?  
                                     
Start position of the estimated full-length sequence in genome.
Start index  
                                 :
                             
                             655408 
                         
                         
                             sequence end
                                   ?  
                                     
End position of the estimated full-length sequence in genome.
Start index  
                                 :
                             
                             655593 
                         
                         
                             bit score (CM)
                                   ?  
                                     
The score for aligning estimated full-length sequence to CM model
  (computed by RSEARCH -> default,
  infered from Rfam or provided by user) 
                                 :
                             
                             113.0 
                         
                         
                             Homology estimate
                                   ?  
                                     
Quick homology estimate:
  Not homologous: bit score   20 and bit score > 0.5 * query length
  Uncertain otherwise 
                                 :
                             
                             Homologous   
                         
                     
                    
                 
                
                
                 
                     
                          Estimated full-length sequence:  
                         
                          ? 
                             
Click checkbox to select multiple seuqences.
Fasta header format:
  UID|accession.versionSTRAND start-end 
                         
                     
                     >uid:54|NW_015441080.1rc 655408-655593
AUUGUUUCGAAUAAAGUUUUUAAAAUACAAAAAAAUCUGUUCUUAUCAGCUUAAUAUCUG
AUACGCUGCUCAUUGAGCAGCUCAUAUAUUAAACUGAUUUUUGGAACCGGGCUGUGGAAA
AGAGGCUUGCCUCGUCCCAGCCACGGGUUGCCUCGGUAUAGCACUACCUCCGAGCGCGGC
CCACUU
 
                 
                
             
            
             
                
                     
                         
                         
                             rnafold 
                             
                              ? 
                                 
Visualisation of predicted secondary structure.
To save the image:
  Right click on the image -> Save Image as. 
                             
                         
                     
                
                     
                         
                         
                             rfam-Rc 
                             
                              ? 
                                 
Visualisation of predicted secondary structure.
To save the image:
  Right click on the image -> Save Image as. 
                             
                         
                     
                
                     
                         
                         
                             TurboFold 
                             
                              ? 
                                 
Visualisation of predicted secondary structure.
To save the image:
  Right click on the image -> Save Image as. 
                             
                         
                     
                
             
            

        
             
                 Load Sequence viewer 
             
        
        
         
     
    

    
     
         
            Hit: NW_015441080.1
         
         
             
                 
                     NW_015441080.1 Acropora digitifera unplaced genomic scaffold, Adig_1.1 DF970715.1, whole genome shotgun sequence 
                     
                         
 ?  
This is BLAST alignment as read from the input file 
                         
 Score = 83.0 bits (76.1), Expect = 1.32E-11
 Identities = 99/136 (73%), Gaps = 1/136 (1%)
 Strand = Plus/Minus
Query     38 ATCTGTTCTTATCAGTTTAATATCTGATACG-TCCTCTATCCGAGGACAATATATTAAATGGAT 100   
             ||||||||||||||| ||||||||||||| | | |||  |  |  |   |||||||| |  |||       
Sbjct 670437 ATCTGTTCTTATCAGCTTAATATCTGATATGCTGCTCATTGAGCAGCTCATATATTACACTGAT 670374

Query    101 TTTTGGAGCAGGGAGATGGAATAGGAGCTTGCTCTGTCCACTCCACGCATCGACCTGGTATTGC 164   
             ||||||| | |||   ||||| ||  ||||||   ||||   |||||  | | | |||||| ||       
Sbjct 670373 TTTTGGAACTGGGCTGTGGAAAAGAGGCTTGCCTCGTCCCAGCCACGGGTTGCCTTGGTATAGC 670310

Query    165 AGTACCTC 172   
             | ||||||       
Sbjct 670309 ACTACCTC 670302

 
                 
                
                 
                      Report:  
                     
                         
                             sequence start
                                   ?  
                                     
Start position of the estimated full-length sequence in genome.
Start index  
                                 :
                             
                             670286 
                         
                         
                             sequence end
                                   ?  
                                     
End position of the estimated full-length sequence in genome.
Start index  
                                 :
                             
                             670474 
                         
                         
                             bit score (CM)
                                   ?  
                                     
The score for aligning estimated full-length sequence to CM model
  (computed by RSEARCH -> default,
  infered from Rfam or provided by user) 
                                 :
                             
                             90.04 
                         
                         
                             Homology estimate
                                   ?  
                                     
Quick homology estimate:
  Not homologous: bit score   20 and bit score > 0.5 * query length
  Uncertain otherwise 
                                 :
                             
                             Uncertain  ↴  
                         
                     
                    
                     
                        Check the secondary structure and sequence viewer
                        for supporting information about possible homology.
                     
                    
                 
                
                
                 
                     
                          Estimated full-length sequence:  
                         
                          ? 
                             
Click checkbox to select multiple seuqences.
Fasta header format:
  UID|accession.versionSTRAND start-end 
                         
                     
                     >uid:55|NW_015441080.1rc 670286-670474
ACACUUUUAUUGUAAAUAAACGGUUUUUUCAAAAAAAAUCUGUUCUUAUCAGCUUAAUAU
CUGAUAUGCUGCUCAUUGAGCAGCUCAUAUAUUACACUGAUUUUUGGAACUGGGCUGUGG
AAAAGAGGCUUGCCUCGUCCCAGCCACGGGUUGCCUUGGUAUAGCACUACCUCUGAGCGC
GGCCCACUU
 
                 
                
             
            
             
                
                     
                         
                         
                             rnafold 
                             
                              ? 
                                 
Visualisation of predicted secondary structure.
To save the image:
  Right click on the image -> Save Image as. 
                             
                         
                     
                
                     
                         
                         
                             rfam-Rc 
                             
                              ? 
                                 
Visualisation of predicted secondary structure.
To save the image:
  Right click on the image -> Save Image as. 
                             
                         
                     
                
                     
                         
                         
                             TurboFold 
                             
                              ? 
                                 
Visualisation of predicted secondary structure.
To save the image:
  Right click on the image -> Save Image as. 
                             
                         
                     
                
             
            

        
             
                 Load Sequence viewer 
             
        
        
         
     
    

    
     
         
            Hit: NW_015441080.1
         
         
             
                 
                     NW_015441080.1 Acropora digitifera unplaced genomic scaffold, Adig_1.1 DF970715.1, whole genome shotgun sequence 
                     
                         
 ?  
This is BLAST alignment as read from the input file 
                         
 Score = 80.0 bits (73.4), Expect = 4.60E-11
 Identities = 99/137 (72%), Gaps = 1/137 (1%)
 Strand = Plus/Minus
Query     38 ATCTGTTCTTATCAGTTTAATATCTGATACG-TCCTCTATCCGAGGACAATATATTAAATGGAT 100   
             ||||||||||||||| ||||||||||||| | | |||  |  |  |   ||||||||||  |||       
Sbjct 739187 ATCTGTTCTTATCAGCTTAATATCTGATATGCTGCTCATTGAGCAGCTCATATATTAAACTGAT 739124

Query    101 TTTTGGAGCAGGGAGATGGAATAGGAGCTTGCTCTGTCCACTCCACGCATCGACCTGGTATTGC 164   
             ||||||| | |||   ||||| ||  ||||||   ||||    ||||  | | |  ||||| ||       
Sbjct 739123 TTTTGGAACCGGGCTGTGGAAAAGAGGCTTGCCTCGTCCCAGTCACGGGTTGCCTCGGTATAGC 739060

Query    165 AGTACCTCC 173   
             | |||||||       
Sbjct 739059 ACTACCTCC 739051

 
                 
                
                 
                      Report:  
                     
                         
                             sequence start
                                   ?  
                                     
Start position of the estimated full-length sequence in genome.
Start index  
                                 :
                             
                             739037 
                         
                         
                             sequence end
                                   ?  
                                     
End position of the estimated full-length sequence in genome.
Start index  
                                 :
                             
                             739224 
                         
                         
                             bit score (CM)
                                   ?  
                                     
The score for aligning estimated full-length sequence to CM model
  (computed by RSEARCH -> default,
  infered from Rfam or provided by user) 
                                 :
                             
                             95.45 
                         
                         
                             Homology estimate
                                   ?  
                                     
Quick homology estimate:
  Not homologous: bit score   20 and bit score > 0.5 * query length
  Uncertain otherwise 
                                 :
                             
                             Homologous   
                         
                     
                    
                 
                
                
                 
                     
                          Estimated full-length sequence:  
                         
                          ? 
                             
Click checkbox to select multiple seuqences.
Fasta header format:
  UID|accession.versionSTRAND start-end 
                         
                     
                     >uid:56|NW_015441080.1rc 739037-739224
UACAUUUGCUGAAUAAAAGUUUUAAAAAGGAAACAAAUCUGUUCUUAUCAGCUUAAUAUC
UGAUAUGCUGCUCAUUGAGCAGCUCAUAUAUUAAACUGAUUUUUGGAACCGGGCUGUGGA
AAAGAGGCUUGCCUCGUCCCAGUCACGGGUUGCCUCGGUAUAGCACUACCUCCGAGCGCG
GCCCACUU
 
                 
                
             
            
             
                
                     
                         
                         
                             rnafold 
                             
                              ? 
                                 
Visualisation of predicted secondary structure.
To save the image:
  Right click on the image -> Save Image as. 
                             
                         
                     
                
                     
                         
                         
                             rfam-Rc 
                             
                              ? 
                                 
Visualisation of predicted secondary structure.
To save the image:
  Right click on the image -> Save Image as. 
                             
                         
                     
                
                     
                         
                         
                             TurboFold 
                             
                              ? 
                                 
Visualisation of predicted secondary structure.
To save the image:
  Right click on the image -> Save Image as. 
                             
                         
                     
                
             
            

        
             
                 Load Sequence viewer 
             
        
        
         
     
    

    
     
         
            Hit: NW_015441080.1
         
         
             
                 
                     NW_015441080.1 Acropora digitifera unplaced genomic scaffold, Adig_1.1 DF970715.1, whole genome shotgun sequence 
                     
                         
 ?  
This is BLAST alignment as read from the input file 
                         
 Score = 80.0 bits (73.4), Expect = 4.60E-11
 Identities = 99/137 (72%), Gaps = 1/137 (1%)
 Strand = Plus/Minus
Query     38 ATCTGTTCTTATCAGTTTAATATCTGATACG-TCCTCTATCCGAGGACAATATATTAAATGGAT 100   
             ||||||||||||||| |||||||| |||| | | |||  |  |  |   ||||||||||  |||       
Sbjct 757694 ATCTGTTCTTATCAGCTTAATATCCGATATGCTGCTCATTAAGCAGCTCATATATTAAACTGAT 757631

Query    101 TTTTGGAGCAGGGAGATGGAATAGGAGCTTGCTCTGTCCACTCCACGCATCGACCTGGTATTGC 164   
             ||||||| | |||   ||||| ||  ||||||   ||||   |||||  | | |  ||||| ||       
Sbjct 757630 TTTTGGAACCGGGCTGTGGAAAAGAGGCTTGCCTCGTCCCAGCCACGAGTTGCCTCGGTATAGC 757567

Query    165 AGTACCTCC 173   
             | |||||||       
Sbjct 757566 ACTACCTCC 757558

 
                 
                
                 
                      Report:  
                     
                         
                             sequence start
                                   ?  
                                     
Start position of the estimated full-length sequence in genome.
Start index  
                                 :
                             
                             757544 
                         
                         
                             sequence end
                                   ?  
                                     
End position of the estimated full-length sequence in genome.
Start index  
                                 :
                             
                             757731 
                         
                         
                             bit score (CM)
                                   ?  
                                     
The score for aligning estimated full-length sequence to CM model
  (computed by RSEARCH -> default,
  infered from Rfam or provided by user) 
                                 :
                             
                             94.75 
                         
                         
                             Homology estimate
                                   ?  
                                     
Quick homology estimate:
  Not homologous: bit score   20 and bit score > 0.5 * query length
  Uncertain otherwise 
                                 :
                             
                             Homologous   
                         
                     
                    
                 
                
                
                 
                     
                          Estimated full-length sequence:  
                         
                          ? 
                             
Click checkbox to select multiple seuqences.
Fasta header format:
  UID|accession.versionSTRAND start-end 
                         
                     
                     >uid:57|NW_015441080.1rc 757544-757731
ACGGUUGCAAAUAAACUUUUAUCCUAAAUAAAAAAAAUCUGUUCUUAUCAGCUUAAUAUC
CGAUAUGCUGCUCAUUAAGCAGCUCAUAUAUUAAACUGAUUUUUGGAACCGGGCUGUGGA
AAAGAGGCUUGCCUCGUCCCAGCCACGAGUUGCCUCGGUAUAGCACUACCUCCGAGCGCA
ACCCACUU
 
                 
                
             
            
             
                
                     
                         
                         
                             rnafold 
                             
                              ? 
                                 
Visualisation of predicted secondary structure.
To save the image:
  Right click on the image -> Save Image as. 
                             
                         
                     
                
                     
                         
                         
                             rfam-Rc 
                             
                              ? 
                                 
Visualisation of predicted secondary structure.
To save the image:
  Right click on the image -> Save Image as. 
                             
                         
                     
                
                     
                         
                         
                             TurboFold 
                             
                              ? 
                                 
Visualisation of predicted secondary structure.
To save the image:
  Right click on the image -> Save Image as. 
                             
                         
                     
                
             
            

        
             
                 Load Sequence viewer 
             
        
        
         
     
    

    
     
         
            Hit: NW_015441080.1
         
         
             
                 
                     NW_015441080.1 Acropora digitifera unplaced genomic scaffold, Adig_1.1 DF970715.1, whole genome shotgun sequence 
                     
                         
 ?  
This is BLAST alignment as read from the input file 
                         
 Score = 75.0 bits (68.9), Expect = 1.96E-09
 Identities = 98/137 (72%), Gaps = 1/137 (1%)
 Strand = Plus/Minus
Query     38 ATCTGTTCTTATCAGTTTAATATCTGATACG-TCCTCTATCCGAGGACAATATATTAAATGGAT 100   
             ||| ||||||||||| ||||||||||||||| | |||  |  |  |   ||||||| ||  |||       
Sbjct 745053 ATCAGTTCTTATCAGCTTAATATCTGATACGCTGCTCATTGAGCAGCTCATATATTGAACTGAT 744990

Query    101 TTTTGGAGCAGGGAGATGGAATAGGAGCTTGCTCTGTCCACTCCACGCATCGACCTGGTATTGC 164   
             ||||||| | |||   ||||| ||  ||||||    |||   |||||  | | |  ||||| ||       
Sbjct 744989 TTTTGGAACCGGGCTGTGGAAAAGAGGCTTGCCTCATCCCAGCCACGGGTTGCCTCGGTATAGC 744926

Query    165 AGTACCTCC 173   
             | |||||||       
Sbjct 744925 ACTACCTCC 744917

 
                 
                
                 
                      Report:  
                     
                         
                             sequence start
                                   ?  
                                     
Start position of the estimated full-length sequence in genome.
Start index  
                                 :
                             
                             744907 
                         
                         
                             sequence end
                                   ?  
                                     
End position of the estimated full-length sequence in genome.
Start index  
                                 :
                             
                             745090 
                         
                         
                             bit score (CM)
                                   ?  
                                     
The score for aligning estimated full-length sequence to CM model
  (computed by RSEARCH -> default,
  infered from Rfam or provided by user) 
                                 :
                             
                             93.54 
                         
                         
                             Homology estimate
                                   ?  
                                     
Quick homology estimate:
  Not homologous: bit score   20 and bit score > 0.5 * query length
  Uncertain otherwise 
                                 :
                             
                             Uncertain  ↴  
                         
                     
                    
                     
                        Check the secondary structure and sequence viewer
                        for supporting information about possible homology.
                     
                    
                 
                
                
                 
                     
                          Estimated full-length sequence:  
                         
                          ? 
                             
Click checkbox to select multiple seuqences.
Fasta header format:
  UID|accession.versionSTRAND start-end 
                         
                     
                     >uid:58|NW_015441080.1rc 744907-745090
GUCACGGUUGUAAAUAAAUUUUUAUAAAAAAAAUCAGUUCUUAUCAGCUUAAUAUCUGAU
ACGCUGCUCAUUGAGCAGCUCAUAUAUUGAACUGAUUUUUGGAACCGGGCUGUGGAAAAG
AGGCUUGCCUCAUCCCAGCCACGGGUUGCCUCGGUAUAGCACUACCUCCGAGCGUGGCCC
ACUU
 
                 
                
             
            
             
                
                     
                         
                         
                             rnafold 
                             
                              ? 
                                 
Visualisation of predicted secondary structure.
To save the image:
  Right click on the image -> Save Image as. 
                             
                         
                     
                
                     
                         
                         
                             rfam-Rc 
                             
                              ? 
                                 
Visualisation of predicted secondary structure.
To save the image:
  Right click on the image -> Save Image as. 
                             
                         
                     
                
                     
                         
                         
                             TurboFold 
                             
                              ? 
                                 
Visualisation of predicted secondary structure.
To save the image:
  Right click on the image -> Save Image as. 
                             
                         
                     
                
             
            

        
             
                 Load Sequence viewer 
             
        
        
         
     
    

    
     
         
            Hit: NW_015441080.1
         
         
             
                 
                     NW_015441080.1 Acropora digitifera unplaced genomic scaffold, Adig_1.1 DF970715.1, whole genome shotgun sequence 
                     
                         
 ?  
This is BLAST alignment as read from the input file 
                         
 Score = 63.0 bits (58.1), Expect = 3.53E-06
 Identities = 33/34 (97%), Gaps = 0/34 (0%)
 Strand = Plus/Minus
Query      1 ATCGCTTCTCGGCCTTTTGGCTAAGATCAAGTGT 34    
             ||||||||||||||||||||||||||||||| ||       
Sbjct 731538 ATCGCTTCTCGGCCTTTTGGCTAAGATCAAGAGT 731505

 
                 
                
                 
                      Report:  
                     
                         
                             sequence start
                                   ?  
                                     
Start position of the estimated full-length sequence in genome.
Start index  
                                 :
                             
                             731351 
                         
                         
                             sequence end
                                   ?  
                                     
End position of the estimated full-length sequence in genome.
Start index  
                                 :
                             
                             731530 
                         
                         
                             bit score (CM)
                                   ?  
                                     
The score for aligning estimated full-length sequence to CM model
  (computed by RSEARCH -> default,
  infered from Rfam or provided by user) 
                                 :
                             
                             12.9 
                         
                         
                             Homology estimate
                                   ?  
                                     
Quick homology estimate:
  Not homologous: bit score   20 and bit score > 0.5 * query length
  Uncertain otherwise 
                                 :
                             
                             Uncertain  ↴  
                         
                     
                    
                     
                        Check the secondary structure and sequence viewer
                        for supporting information about possible homology.
                     
                    
                 
                
                
                 
                     
                          Estimated full-length sequence:  
                         
                          ? 
                             
Click checkbox to select multiple seuqences.
Fasta header format:
  UID|accession.versionSTRAND start-end 
                         
                     
                     >uid:59|NW_015441080.1rc 731351-731530
AUCGCUUCUCGGCCUUUUGGCUAAGAUCAAGAGUGGGUCUUGUAUCUGGCCAUAAGGAUU
GGUUUUUGUUUCUUGUGAUUACGUACGUCCUGUGCUAAGUACCCGCCGGGGGGGAACGCC
AUGGACCCCCUCCCCCAGAGAAGUGAUGUUUCCCGCCAAAACGAGGCAAAUGCUGACGUC
 
                 
                
             
            
             
                
                     
                         
                         
                             rnafold 
                             
                              ? 
                                 
Visualisation of predicted secondary structure.
To save the image:
  Right click on the image -> Save Image as. 
                             
                         
                     
                
                     
                         
                         
                             rfam-Rc 
                             
                              ? 
                                 
Visualisation of predicted secondary structure.
To save the image:
  Right click on the image -> Save Image as. 
                             
                         
                     
                
                     
                         
                         
                             TurboFold 
                             
                              ? 
                                 
Visualisation of predicted secondary structure.
To save the image:
  Right click on the image -> Save Image as. 
                             
                         
                     
                
             
            

        
             
                 Load Sequence viewer 
             
        
        
         
     
    

    
     
         
            Hit: NW_015441080.1
         
         
             
                 
                     NW_015441080.1 Acropora digitifera unplaced genomic scaffold, Adig_1.1 DF970715.1, whole genome shotgun sequence 
                     
                         
 ?  
This is BLAST alignment as read from the input file 
                         
 Score = 52.0 bits (48.2), Expect = 1.83E-03
 Identities = 26/26 (100%), Gaps = 0/26 (0%)
 Strand = Plus/Minus
Query      1 ATCGCTTCTCGGCCTTTTGGCTAAGA 26    
             ||||||||||||||||||||||||||       
Sbjct 657050 ATCGCTTCTCGGCCTTTTGGCTAAGA 657025

 
                 
                
                 
                      Report:  
                     
                         
                             sequence start
                                   ?  
                                     
Start position of the estimated full-length sequence in genome.
Start index  
                                 :
                             
                             656863 
                         
                         
                             sequence end
                                   ?  
                                     
End position of the estimated full-length sequence in genome.
Start index  
                                 :
                             
                             657050 
                         
                         
                             bit score (CM)
                                   ?  
                                     
The score for aligning estimated full-length sequence to CM model
  (computed by RSEARCH -> default,
  infered from Rfam or provided by user) 
                                 :
                             
                             6.85 
                         
                         
                             Homology estimate
                                   ?  
                                     
Quick homology estimate:
  Not homologous: bit score   20 and bit score > 0.5 * query length
  Uncertain otherwise 
                                 :
                             
                             Uncertain  ↴  
                         
                     
                    
                     
                        Check the secondary structure and sequence viewer
                        for supporting information about possible homology.
                     
                    
                 
                
                
                 
                     
                          Estimated full-length sequence:  
                         
                          ? 
                             
Click checkbox to select multiple seuqences.
Fasta header format:
  UID|accession.versionSTRAND start-end 
                         
                     
                     >uid:60|NW_015441080.1rc 656863-657050
AUCGCUUCUCGGCCUUUUGGCUAAGAGCGAUAGUUGACGUUCUCGUGUUAUUACUUUCAG
GUCAGGAUGUCAAUGCAAGACGACUUCCCGAAAAGUGUAGUGUUGCACUUCCCGCCAAGU
GUCUACCAAAAGAUUGGUGCCUCCGAGAUGUUACCAUAUUUGUUGGAGUCUUUGAAGAUU
GAAGACCU
 
                 
                
             
            
             
                
                     
                         
                         
                             rnafold 
                             
                              ? 
                                 
Visualisation of predicted secondary structure.
To save the image:
  Right click on the image -> Save Image as. 
                             
                         
                     
                
                     
                         
                         
                             rfam-Rc 
                             
                              ? 
                                 
Visualisation of predicted secondary structure.
To save the image:
  Right click on the image -> Save Image as. 
                             
                         
                     
                
                     
                         
                         
                             TurboFold 
                             
                              ? 
                                 
Visualisation of predicted secondary structure.
To save the image:
  Right click on the image -> Save Image as. 
                             
                         
                     
                
             
            

        
             
                 Load Sequence viewer 
             
        
        
         
     
    

    
     
         
            Hit: NW_015441080.1
         
         
             
                 
                     NW_015441080.1 Acropora digitifera unplaced genomic scaffold, Adig_1.1 DF970715.1, whole genome shotgun sequence 
                     
                         
 ?  
This is BLAST alignment as read from the input file 
                         
 Score = 51.0 bits (47.3), Expect = 6.39E-03
 Identities = 27/28 (96%), Gaps = 0/28 (0%)
 Strand = Plus/Minus
Query      1 ATCGCTTCTCGGCCTTTTGGCTAAGATC 28    
             ||||||||||||||||||||| ||||||       
Sbjct 653789 ATCGCTTCTCGGCCTTTTGGCCAAGATC 653762

 
                 
                
                 
                      Report:  
                     
                         
                             sequence start
                                   ?  
                                     
Start position of the estimated full-length sequence in genome.
Start index  
                                 :
                             
                             653602 
                         
                         
                             sequence end
                                   ?  
                                     
End position of the estimated full-length sequence in genome.
Start index  
                                 :
                             
                             653783 
                         
                         
                             bit score (CM)
                                   ?  
                                     
The score for aligning estimated full-length sequence to CM model
  (computed by RSEARCH -> default,
  infered from Rfam or provided by user) 
                                 :
                             
                             -3.18 
                         
                         
                             Homology estimate
                                   ?  
                                     
Quick homology estimate:
  Not homologous: bit score   20 and bit score > 0.5 * query length
  Uncertain otherwise 
                                 :
                             
                             Not homologous   
                         
                     
                    
                 
                
                
                 
                     
                          Estimated full-length sequence:  
                         
                          ? 
                             
Click checkbox to select multiple seuqences.
Fasta header format:
  UID|accession.versionSTRAND start-end 
                         
                     
                     >uid:61|NW_015441080.1rc 653602-653783
AUCGCUUCUCGGCCUUUUGGCCAAGAUCUCUUUUUGUCUUUUGCAGAAAGUGCUUUGAGA
AGGGAUGGCUGACGACGAUGAUUCAGAGAUGAGGUUUAUAAACGUGCCCGAAAGAUCAAG
CGAAGAUUUGGUAAUUACAGUACCGAGCGAGGAACGUACCGAGAACGCUUCCAGUUCCUG
GG
 
                 
                
             
            
             
                
                     
                         
                         
                             rnafold 
                             
                              ? 
                                 
Visualisation of predicted secondary structure.
To save the image:
  Right click on the image -> Save Image as. 
                             
                         
                     
                
                     
                         
                         
                             rfam-Rc 
                             
                              ? 
                                 
Visualisation of predicted secondary structure.
To save the image:
  Right click on the image -> Save Image as. 
                             
                         
                     
                
                     
                         
                         
                             TurboFold 
                             
                              ? 
                                 
Visualisation of predicted secondary structure.
To save the image:
  Right click on the image -> Save Image as. 
                             
                         
                     
                
             
            

        
             
                 Load Sequence viewer 
             
        
        
         
     
    

    
     
         
            Hit: NW_015441080.1
         
         
             
                 
                     NW_015441080.1 Acropora digitifera unplaced genomic scaffold, Adig_1.1 DF970715.1, whole genome shotgun sequence 
                     
                         
 ?  
This is BLAST alignment as read from the input file 
                         
 Score = 45.0 bits (41.9), Expect = 2.72E-01
 Identities = 24/25 (96%), Gaps = 0/25 (0%)
 Strand = Plus/Plus
Query      1 ATCGCTTCTCGGCCTTTTGGCTAAG 25    
             ||||||||||||||||||||| |||       
Sbjct 638222 ATCGCTTCTCGGCCTTTTGGCCAAG 638246

 
                 
                
                 
                      Report:  
                     
                         
                             sequence start
                                   ?  
                                     
Start position of the estimated full-length sequence in genome.
Start index  
                                 :
                             
                             638222 
                         
                         
                             sequence end
                                   ?  
                                     
End position of the estimated full-length sequence in genome.
Start index  
                                 :
                             
                             638408 
                         
                         
                             bit score (CM)
                                   ?  
                                     
The score for aligning estimated full-length sequence to CM model
  (computed by RSEARCH -> default,
  infered from Rfam or provided by user) 
                                 :
                             
                             -9.83 
                         
                         
                             Homology estimate
                                   ?  
                                     
Quick homology estimate:
  Not homologous: bit score   20 and bit score > 0.5 * query length
  Uncertain otherwise 
                                 :
                             
                             Not homologous   
                         
                     
                    
                 
                
                
                 
                     
                          Estimated full-length sequence:  
                         
                          ? 
                             
Click checkbox to select multiple seuqences.
Fasta header format:
  UID|accession.versionSTRAND start-end 
                         
                     
                     >uid:62|NW_015441080.1fw 638222-638408
AUCGCUUCUCGGCCUUUUGGCCAAGUUUUCUUUUUGUCUCGUUGCAGAAAGUGCUUCCAG
AAGUGAUGGAUACUUAUUCAGAGAAGAGGUUAAUAGAUGUACCGGAAAGAUCAACUGAAG
AUUUGGUGAUAAGAGUGGCCACUGAAGAACGUACCGAGAACGCAUCCCGUUCCUGGGCUG
UGAGAGC
 
                 
                
             
            
             
                
                     
                         
                         
                             rnafold 
                             
                              ? 
                                 
Visualisation of predicted secondary structure.
To save the image:
  Right click on the image -> Save Image as. 
                             
                         
                     
                
                     
                         
                         
                             rfam-Rc 
                             
                              ? 
                                 
Visualisation of predicted secondary structure.
To save the image:
  Right click on the image -> Save Image as. 
                             
                         
                     
                
                     
                         
                         
                             TurboFold 
                             
                              ? 
                                 
Visualisation of predicted secondary structure.
To save the image:
  Right click on the image -> Save Image as. 
                             
                         
                     
                
             
            

        
             
                 Load Sequence viewer 
             
        
        
         
     
    

    
     
         
            Hit: NW_015441080.1
         
         
             
                 
                     NW_015441080.1 Acropora digitifera unplaced genomic scaffold, Adig_1.1 DF970715.1, whole genome shotgun sequence 
                     
                         
 ?  
This is BLAST alignment as read from the input file 
                         
 Score = 45.0 bits (41.9), Expect = 2.72E-01
 Identities = 24/25 (96%), Gaps = 0/25 (0%)
 Strand = Plus/Minus
Query      1 ATCGCTTCTCGGCCTTTTGGCTAAG 25    
             ||||||||||||||||||||| |||       
Sbjct 642777 ATCGCTTCTCGGCCTTTTGGCCAAG 642753

 
                 
                
                 
                      Report:  
                     
                         
                             sequence start
                                   ?  
                                     
Start position of the estimated full-length sequence in genome.
Start index  
                                 :
                             
                             642590 
                         
                         
                             sequence end
                                   ?  
                                     
End position of the estimated full-length sequence in genome.
Start index  
                                 :
                             
                             642788 
                         
                         
                             bit score (CM)
                                   ?  
                                     
The score for aligning estimated full-length sequence to CM model
  (computed by RSEARCH -> default,
  infered from Rfam or provided by user) 
                                 :
                             
                             -9.19 
                         
                         
                             Homology estimate
                                   ?  
                                     
Quick homology estimate:
  Not homologous: bit score   20 and bit score > 0.5 * query length
  Uncertain otherwise 
                                 :
                             
                             Not homologous   
                         
                     
                    
                 
                
                
                 
                     
                          Estimated full-length sequence:  
                         
                          ? 
                             
Click checkbox to select multiple seuqences.
Fasta header format:
  UID|accession.versionSTRAND start-end 
                         
                     
                     >uid:63|NW_015441080.1rc 642590-642788
AUCGCUUCUCGGCCUUUUGGCCAAGUUUUCUUUUUUGCAAAGAGGGUCGUCAGAAGCGAU
GGCGGAUGGUGAUUCUGAGAUGAGGUUCAUAGAUGUUCCAGAGAGAACAGAAGAAAAUCU
GGUUAUUACAGUUCCUAGAGAGGAACGUAAGCAGAACGACUUCCAAUCUUGGGCCGAUCG
AGUGGAGAACUCUGAGCCC
 
                 
                
             
            
             
                
                     
                         
                         
                             rnafold 
                             
                              ? 
                                 
Visualisation of predicted secondary structure.
To save the image:
  Right click on the image -> Save Image as. 
                             
                         
                     
                
                     
                         
                         
                             rfam-Rc 
                             
                              ? 
                                 
Visualisation of predicted secondary structure.
To save the image:
  Right click on the image -> Save Image as. 
                             
                         
                     
                
                     
                         
                         
                             TurboFold 
                             
                              ? 
                                 
Visualisation of predicted secondary structure.
To save the image:
  Right click on the image -> Save Image as. 
                             
                         
                     
                
             
            

        
             
                 Load Sequence viewer 
             
        
        
         
     
    

    
     
         
            Hit: NW_015441080.1
         
         
             
                 
                     NW_015441080.1 Acropora digitifera unplaced genomic scaffold, Adig_1.1 DF970715.1, whole genome shotgun sequence 
                     
                         
 ?  
This is BLAST alignment as read from the input file 
                         
 Score = 45.0 bits (41.9), Expect = 2.72E-01
 Identities = 24/25 (96%), Gaps = 0/25 (0%)
 Strand = Plus/Minus
Query      1 ATCGCTTCTCGGCCTTTTGGCTAAG 25    
             |||||||||||||||||| ||||||       
Sbjct 675387 ATCGCTTCTCGGCCTTTTTGCTAAG 675363

 
                 
                
                 
                      Report:  
                     
                         
                             sequence start
                                   ?  
                                     
Start position of the estimated full-length sequence in genome.
Start index  
                                 :
                             
                             675200 
                         
                         
                             sequence end
                                   ?  
                                     
End position of the estimated full-length sequence in genome.
Start index  
                                 :
                             
                             675393 
                         
                         
                             bit score (CM)
                                   ?  
                                     
The score for aligning estimated full-length sequence to CM model
  (computed by RSEARCH -> default,
  infered from Rfam or provided by user) 
                                 :
                             
                             -14.57 
                         
                         
                             Homology estimate
                                   ?  
                                     
Quick homology estimate:
  Not homologous: bit score   20 and bit score > 0.5 * query length
  Uncertain otherwise 
                                 :
                             
                             Not homologous   
                         
                     
                    
                 
                
                
                 
                     
                          Estimated full-length sequence:  
                         
                          ? 
                             
Click checkbox to select multiple seuqences.
Fasta header format:
  UID|accession.versionSTRAND start-end 
                         
                     
                     >uid:64|NW_015441080.1rc 675200-675393
AUCGCUUCUCGGCCUUUUUGCUAAGCCAUUUUGCUGCGUUGUUUCACCCUCUUCCUCUUA
GGAGGUAUCUUCUAUGGAUAACGAUAUUGGUAAUGGUUCCCAGAAUUGGUGUGAUUCUUG
CGAAAAUGAUGUGAUGUCUGAAGUUCCUAAUCGUGCUUAUUCCGCUGUUUUAAUGUCUGG
UGUACAUGAUGGAU
 
                 
                
             
            
             
                
                     
                         
                         
                             rnafold 
                             
                              ? 
                                 
Visualisation of predicted secondary structure.
To save the image:
  Right click on the image -> Save Image as. 
                             
                         
                     
                
                     
                         
                         
                             rfam-Rc 
                             
                              ? 
                                 
Visualisation of predicted secondary structure.
To save the image:
  Right click on the image -> Save Image as. 
                             
                         
                     
                
                     
                         
                         
                             TurboFold 
                             
                              ? 
                                 
Visualisation of predicted secondary structure.
To save the image:
  Right click on the image -> Save Image as. 
                             
                         
                     
                
             
            

        
             
                 Load Sequence viewer 
             
        
        
         
     
    

    
     
         
            Hit: NW_015441080.1
         
         
             
                 
                     NW_015441080.1 Acropora digitifera unplaced genomic scaffold, Adig_1.1 DF970715.1, whole genome shotgun sequence 
                     
                         
 ?  
This is BLAST alignment as read from the input file 
                         
 Score = 44.0 bits (41.0), Expect = 2.72E-01
 Identities = 25/27 (93%), Gaps = 0/27 (0%)
 Strand = Plus/Minus
Query      1 ATCGCTTCTCGGCCTTTTGGCTAAGAT 27    
             |||||||||||||||| |||| |||||       
Sbjct 696570 ATCGCTTCTCGGCCTTCTGGCCAAGAT 696544

 
                 
                
                 
                      Report:  
                     
                         
                             sequence start
                                   ?  
                                     
Start position of the estimated full-length sequence in genome.
Start index  
                                 :
                             
                             696383 
                         
                         
                             sequence end
                                   ?  
                                     
End position of the estimated full-length sequence in genome.
Start index  
                                 :
                             
                             696568 
                         
                         
                             bit score (CM)
                                   ?  
                                     
The score for aligning estimated full-length sequence to CM model
  (computed by RSEARCH -> default,
  infered from Rfam or provided by user) 
                                 :
                             
                             -4.68 
                         
                         
                             Homology estimate
                                   ?  
                                     
Quick homology estimate:
  Not homologous: bit score   20 and bit score > 0.5 * query length
  Uncertain otherwise 
                                 :
                             
                             Not homologous   
                         
                     
                    
                 
                
                
                 
                     
                          Estimated full-length sequence:  
                         
                          ? 
                             
Click checkbox to select multiple seuqences.
Fasta header format:
  UID|accession.versionSTRAND start-end 
                         
                     
                     >uid:65|NW_015441080.1rc 696383-696568
AUCGCUUCUCGGCCUUCUGGCCAAGAUAUUUUCAUACUUUUCGCAGGUGAAGAUGCAAGG
AGCGGACGAUAUAAGAGAUGAGGUGAAUUUGACAUUUGUACCAGAAAGAAGUAAUGAAGA
CAUCGAAGUUGUAACAGAACGUACCAGUGACGACACCCGUAUUUGGGCAGAUGUUGUGCA
GGAGUC
 
                 
                
             
            
             
                
                     
                         
                         
                             rnafold 
                             
                              ? 
                                 
Visualisation of predicted secondary structure.
To save the image:
  Right click on the image -> Save Image as. 
                             
                         
                     
                
                     
                         
                         
                             rfam-Rc 
                             
                              ? 
                                 
Visualisation of predicted secondary structure.
To save the image:
  Right click on the image -> Save Image as. 
                             
                         
                     
                
                     
                         
                         
                             TurboFold 
                             
                              ? 
                                 
Visualisation of predicted secondary structure.
To save the image:
  Right click on the image -> Save Image as. 
                             
                         
                     
                
             
            

        
             
                 Load Sequence viewer 
             
        
        
         
     
    

    
     
         
            Hit: NW_015441080.1
         
         
             
                 
                     NW_015441080.1 Acropora digitifera unplaced genomic scaffold, Adig_1.1 DF970715.1, whole genome shotgun sequence 
                     
                         
 ?  
This is BLAST alignment as read from the input file 
                         
 Score = 44.0 bits (41.0), Expect = 2.72E-01
 Identities = 22/22 (100%), Gaps = 0/22 (0%)
 Strand = Plus/Minus
Query      1 ATCGCTTCTCGGCCTTTTGGCT 22    
             ||||||||||||||||||||||       
Sbjct 749897 ATCGCTTCTCGGCCTTTTGGCT 749876

 
                 
                
                 
                      Report:  
                     
                         
                             sequence start
                                   ?  
                                     
Start position of the estimated full-length sequence in genome.
Start index  
                                 :
                             
                             749710 
                         
                         
                             sequence end
                                   ?  
                                     
End position of the estimated full-length sequence in genome.
Start index  
                                 :
                             
                             749888 
                         
                         
                             bit score (CM)
                                   ?  
                                     
The score for aligning estimated full-length sequence to CM model
  (computed by RSEARCH -> default,
  infered from Rfam or provided by user) 
                                 :
                             
                             8.94 
                         
                         
                             Homology estimate
                                   ?  
                                     
Quick homology estimate:
  Not homologous: bit score   20 and bit score > 0.5 * query length
  Uncertain otherwise 
                                 :
                             
                             Uncertain  ↴  
                         
                     
                    
                     
                        Check the secondary structure and sequence viewer
                        for supporting information about possible homology.
                     
                    
                 
                
                
                 
                     
                          Estimated full-length sequence:  
                         
                          ? 
                             
Click checkbox to select multiple seuqences.
Fasta header format:
  UID|accession.versionSTRAND start-end 
                         
                     
                     >uid:66|NW_015441080.1rc 749710-749888
AUCGCUUCUCGGCCUUUUGGCUGGAUCCAGAAGGCUUUUUUGCUUUUAUCUCCAUAGAUU
GCCACGAUGUCUGACAGCAAUUUGAUGGAGAUUUUACAAGAUGUUGAAUCGCCUGUUUUG
AUUAGCGGAGAGAGUACUUUGGUUCCAGAAGAUGAACAUGCAAUUGCAGAUGAAGACCU
 
                 
                
             
            
             
                
                     
                         
                         
                             rnafold 
                             
                              ? 
                                 
Visualisation of predicted secondary structure.
To save the image:
  Right click on the image -> Save Image as. 
                             
                         
                     
                
                     
                         
                         
                             rfam-Rc 
                             
                              ? 
                                 
Visualisation of predicted secondary structure.
To save the image:
  Right click on the image -> Save Image as. 
                             
                         
                     
                
                     
                         
                         
                             TurboFold 
                             
                              ? 
                                 
Visualisation of predicted secondary structure.
To save the image:
  Right click on the image -> Save Image as. 
                             
                         
                     
                
             
            

        
             
                 Load Sequence viewer 
             
        
        
         
     
    

    
     
         
            Hit: NW_015441080.1
         
         
             
                 
                     NW_015441080.1 Acropora digitifera unplaced genomic scaffold, Adig_1.1 DF970715.1, whole genome shotgun sequence 
                     
                         
 ?  
This is BLAST alignment as read from the input file 
                         
 Score = 40.0 bits (37.4), Expect = 3.31E+00
 Identities = 23/25 (92%), Gaps = 0/25 (0%)
 Strand = Plus/Minus
Query      1 ATCGCTTCTCGGCCTTTTGGCTAAG 25    
             |||||||||||||||||| ||| ||       
Sbjct 763903 ATCGCTTCTCGGCCTTTTAGCTTAG 763879

 
                 
                
                 
                      Report:  
                     
                         
                             sequence start
                                   ?  
                                     
Start position of the estimated full-length sequence in genome.
Start index  
                                 :
                             
                             763716 
                         
                         
                             sequence end
                                   ?  
                                     
End position of the estimated full-length sequence in genome.
Start index  
                                 :
                             
                             763897 
                         
                         
                             bit score (CM)
                                   ?  
                                     
The score for aligning estimated full-length sequence to CM model
  (computed by RSEARCH -> default,
  infered from Rfam or provided by user) 
                                 :
                             
                             -2.16 
                         
                         
                             Homology estimate
                                   ?  
                                     
Quick homology estimate:
  Not homologous: bit score   20 and bit score > 0.5 * query length
  Uncertain otherwise 
                                 :
                             
                             Not homologous   
                         
                     
                    
                 
                
                
                 
                     
                          Estimated full-length sequence:  
                         
                          ? 
                             
Click checkbox to select multiple seuqences.
Fasta header format:
  UID|accession.versionSTRAND start-end 
                         
                     
                     >uid:67|NW_015441080.1rc 763716-763897
AUCGCUUCUCGGCCUUUUAGCUUAGGCCGUUUUCCUUGUUUUUGCAGAGGUGAGAUGACU
GAAGCGAUGGAUAGAGAUGAGGAGAAUACAAUGGUCGAUGUACCAGAGAGACAAACUGAA
GAUUUGGUGAUUACAGUAACCACGGAACCAGUGACGAAUCUCCAUGCUGGGCCGACCGAG
UC
 
                 
                
             
            
             
                
                     
                         
                         
                             rnafold 
                             
                              ? 
                                 
Visualisation of predicted secondary structure.
To save the image:
  Right click on the image -> Save Image as. 
                             
                         
                     
                
                     
                         
                         
                             rfam-Rc 
                             
                              ? 
                                 
Visualisation of predicted secondary structure.
To save the image:
  Right click on the image -> Save Image as. 
                             
                         
                     
                
                     
                         
                         
                             TurboFold 
                             
                              ? 
                                 
Visualisation of predicted secondary structure.
To save the image:
  Right click on the image -> Save Image as. 
                             
                         
                     
                
             
            

        
             
                 Load Sequence viewer 
             
        
        
         
     
    

    
     
         
            Hit: NW_015441095.1
         
         
             
                 
                     NW_015441095.1 Acropora digitifera unplaced genomic scaffold, Adig_1.1 DF970730.1, whole genome shotgun sequence 
                     
                         
 ?  
This is BLAST alignment as read from the input file 
                         
 Score = 164.0 bits (149.2), Expect = 7.28E-34
 Identities = 138/174 (79%), Gaps = 1/174 (1%)
 Strand = Plus/Minus
Query      1 ATCGCTTCTCGGCCTTTTGGCTAAGATCAAGTGTAGTATCTGTTCTTATCAGTTTAATATCTGA 64    
             |||||||||||||||||||||||||||||||||||||||||||||||||||| |||||||||||       
Sbjct 184113 ATCGCTTCTCGGCCTTTTGGCTAAGATCAAGTGTAGTATCTGTTCTTATCAGCTTAATATCTGA 184050

Query     65 TACG-TCCTCTATCCGAGGACAATATATTAAATGGATTTTTGGAGCAGGGAGATGGAATAGGAG 127   
             |||| | |||  |  |  |   ||||||||||  |||||||||| | |||   ||||| ||  |       
Sbjct 184049 TACGCTGCTCATTGAGCAGCTCATATATTAAACTGATTTTTGGAACCGGGCTGTGGAAAAGAGG 183986

Query    128 CTTGCTCTGTCCACTCCACGCATCGACCTGGTATTGCAGTACCTCC 173   
             |||||   ||||   |||||  | | |  ||||| ||| |||||||       
Sbjct 183985 CTTGCCTCGTCCCAGCCACGGGTTGCCTCGGTATAGCACTACCTCC 183940

 
                 
                
                 
                      Report:  
                     
                         
                             sequence start
                                   ?  
                                     
Start position of the estimated full-length sequence in genome.
Start index  
                                 :
                             
                             183925 
                         
                         
                             sequence end
                                   ?  
                                     
End position of the estimated full-length sequence in genome.
Start index  
                                 :
                             
                             184113 
                         
                         
                             bit score (CM)
                                   ?  
                                     
The score for aligning estimated full-length sequence to CM model
  (computed by RSEARCH -> default,
  infered from Rfam or provided by user) 
                                 :
                             
                             161.93 
                         
                         
                             Homology estimate
                                   ?  
                                     
Quick homology estimate:
  Not homologous: bit score   20 and bit score > 0.5 * query length
  Uncertain otherwise 
                                 :
                             
                             Homologous   
                         
                     
                    
                 
                
                
                 
                     
                          Estimated full-length sequence:  
                         
                          ? 
                             
Click checkbox to select multiple seuqences.
Fasta header format:
  UID|accession.versionSTRAND start-end 
                         
                     
                     >uid:68|NW_015441095.1rc 183925-184113
AUCGCUUCUCGGCCUUUUGGCUAAGAUCAAGUGUAGUAUCUGUUCUUAUCAGCUUAAUAU
CUGAUACGCUGCUCAUUGAGCAGCUCAUAUAUUAAACUGAUUUUUGGAACCGGGCUGUGG
AAAAGAGGCUUGCCUCGUCCCAGCCACGGGUUGCCUCGGUAUAGCACUACCUCCGAGCGC
GGCCCACUU
 
                 
                
             
            
             
                
                     
                         
                         
                             rnafold 
                             
                              ? 
                                 
Visualisation of predicted secondary structure.
To save the image:
  Right click on the image -> Save Image as. 
                             
                         
                     
                
                     
                         
                         
                             rfam-Rc 
                             
                              ? 
                                 
Visualisation of predicted secondary structure.
To save the image:
  Right click on the image -> Save Image as. 
                             
                         
                     
                
                     
                         
                         
                             TurboFold 
                             
                              ? 
                                 
Visualisation of predicted secondary structure.
To save the image:
  Right click on the image -> Save Image as. 
                             
                         
                     
                
             
            

        
             
                 Load Sequence viewer 
             
        
        
         
     
    

    
     
         
            Hit: NW_015441095.1
         
         
             
                 
                     NW_015441095.1 Acropora digitifera unplaced genomic scaffold, Adig_1.1 DF970730.1, whole genome shotgun sequence 
                     
                         
 ?  
This is BLAST alignment as read from the input file 
                         
 Score = 131.0 bits (119.4), Expect = 1.23E-24
 Identities = 67/68 (99%), Gaps = 0/68 (0%)
 Strand = Plus/Plus
Query      1 ATCGCTTCTCGGCCTTTTGGCTAAGATCAAGTGTAGTATCTGTTCTTATCAGTTTAATATCTGA 64    
             |||||||||||||||||||||||||||||||||||||||||||||||||||| |||||||||||       
Sbjct 226413 ATCGCTTCTCGGCCTTTTGGCTAAGATCAAGTGTAGTATCTGTTCTTATCAGCTTAATATCTGA 226476

Query     65 TACG 68    
             ||||       
Sbjct 226477 TACG 226480

 
                 
                
                 
                      Report:  
                     
                         
                             sequence start
                                   ?  
                                     
Start position of the estimated full-length sequence in genome.
Start index  
                                 :
                             
                             226413 
                         
                         
                             sequence end
                                   ?  
                                     
End position of the estimated full-length sequence in genome.
Start index  
                                 :
                             
                             226601 
                         
                         
                             bit score (CM)
                                   ?  
                                     
The score for aligning estimated full-length sequence to CM model
  (computed by RSEARCH -> default,
  infered from Rfam or provided by user) 
                                 :
                             
                             57.51 
                         
                         
                             Homology estimate
                                   ?  
                                     
Quick homology estimate:
  Not homologous: bit score   20 and bit score > 0.5 * query length
  Uncertain otherwise 
                                 :
                             
                             Uncertain  ↴  
                         
                     
                    
                     
                        Check the secondary structure and sequence viewer
                        for supporting information about possible homology.
                     
                    
                 
                
                
                 
                     
                          Estimated full-length sequence:  
                         
                          ? 
                             
Click checkbox to select multiple seuqences.
Fasta header format:
  UID|accession.versionSTRAND start-end 
                         
                     
                     >uid:69|NW_015441095.1fw 226413-226601
AUCGCUUCUCGGCCUUUUGGCUAAGAUCAAGUGUAGUAUCUGUUCUUAUCAGCUUAAUAU
CUGAUACGCUGCUCAUUGAGCAGCUCAUAUAUUAAACUGCACGGGGUAUUCCCAGGCAGU
CAACCAUCCAAGUACUAACCCCGCCCGACAGAGCUUAACUUCGGUGAUUGGACGAGAACC
GUUUUUUUU
 
                 
                
             
            
             
                
                     
                         
                         
                             rnafold 
                             
                              ? 
                                 
Visualisation of predicted secondary structure.
To save the image:
  Right click on the image -> Save Image as. 
                             
                         
                     
                
                     
                         
                         
                             rfam-Rc 
                             
                              ? 
                                 
Visualisation of predicted secondary structure.
To save the image:
  Right click on the image -> Save Image as. 
                             
                         
                     
                
                     
                         
                         
                             TurboFold 
                             
                              ? 
                                 
Visualisation of predicted secondary structure.
To save the image:
  Right click on the image -> Save Image as. 
                             
                         
                     
                
             
            

        
             
                 Load Sequence viewer 
             
        
        
         
     
    

    
     
         
            Hit: NW_015441095.1
         
         
             
                 
                     NW_015441095.1 Acropora digitifera unplaced genomic scaffold, Adig_1.1 DF970730.1, whole genome shotgun sequence 
                     
                         
 ?  
This is BLAST alignment as read from the input file 
                         
 Score = 83.0 bits (76.1), Expect = 1.32E-11
 Identities = 99/136 (73%), Gaps = 1/136 (1%)
 Strand = Plus/Minus
Query     38 ATCTGTTCTTATCAGTTTAATATCTGATACG-TCCTCTATCCGAGGACAATATATTAAATGGAT 100   
             ||||||||||||||| ||||||||||||||| | |||  |  |  |   ||||||||||  |||       
Sbjct 172323 ATCTGTTCTTATCAGCTTAATATCTGATACGCTGCTCATTGAGCAGCTCATATATTAAACTGAT 172260

Query    101 TTTTGGAGCAGGGAGATGGAATAGGAGCTTGCTCTGTCCACTCCACGCATCGACCTGGTATTGC 164   
             ||||||| |  ||   ||||| ||  ||||||   ||||   |||||  | | |  ||||| ||       
Sbjct 172259 TTTTGGAACCAGGCTGTGGAAAAGAGGCTTGCCTCGTCCCAGCCACGGGTTGCCTCGGTATAGC 172196

Query    165 AGTACCTC 172   
             | ||||||       
Sbjct 172195 ACTACCTC 172188

 
                 
                
                 
                      Report:  
                     
                         
                             sequence start
                                   ?  
                                     
Start position of the estimated full-length sequence in genome.
Start index  
                                 :
                             
                             172171 
                         
                         
                             sequence end
                                   ?  
                                     
End position of the estimated full-length sequence in genome.
Start index  
                                 :
                             
                             172360 
                         
                         
                             bit score (CM)
                                   ?  
                                     
The score for aligning estimated full-length sequence to CM model
  (computed by RSEARCH -> default,
  infered from Rfam or provided by user) 
                                 :
                             
                             94.9 
                         
                         
                             Homology estimate
                                   ?  
                                     
Quick homology estimate:
  Not homologous: bit score   20 and bit score > 0.5 * query length
  Uncertain otherwise 
                                 :
                             
                             Homologous   
                         
                     
                    
                 
                
                
                 
                     
                          Estimated full-length sequence:  
                         
                          ? 
                             
Click checkbox to select multiple seuqences.
Fasta header format:
  UID|accession.versionSTRAND start-end 
                         
                     
                     >uid:70|NW_015441095.1rc 172171-172360
UGUUUUAUAAUUAUUAUAUAAAGUUUUAAAAUACAAAAAUCUGUUCUUAUCAGCUUAAUA
UCUGAUACGCUGCUCAUUGAGCAGCUCAUAUAUUAAACUGAUUUUUGGAACCAGGCUGUG
GAAAAGAGGCUUGCCUCGUCCCAGCCACGGGUUGCCUCGGUAUAGCACUACCUCUGAGCG
CGGCCCACUU
 
                 
                
             
            
             
                
                     
                         
                         
                             rnafold 
                             
                              ? 
                                 
Visualisation of predicted secondary structure.
To save the image:
  Right click on the image -> Save Image as. 
                             
                         
                     
                
                     
                         
                         
                             rfam-Rc 
                             
                              ? 
                                 
Visualisation of predicted secondary structure.
To save the image:
  Right click on the image -> Save Image as. 
                             
                         
                     
                
                     
                         
                         
                             TurboFold 
                             
                              ? 
                                 
Visualisation of predicted secondary structure.
To save the image:
  Right click on the image -> Save Image as. 
                             
                         
                     
                
             
            

        
             
                 Load Sequence viewer 
             
        
        
         
     
    

    
     
         
            Hit: NW_015441095.1
         
         
             
                 
                     NW_015441095.1 Acropora digitifera unplaced genomic scaffold, Adig_1.1 DF970730.1, whole genome shotgun sequence 
                     
                         
 ?  
This is BLAST alignment as read from the input file 
                         
 Score = 79.0 bits (72.5), Expect = 1.60E-10
 Identities = 105/145 (72%), Gaps = 4/145 (3%)
 Strand = Plus/Plus
Query     38 ATCTGTTCTTATCAGTTTAATATCTGATACG-TCCTCTATCCGAGGACAATATATTAAATGGAT 100   
             ||||||||||||||| ||||||||||||||| |  ||  |  |  |   ||||||||||  |||       
Sbjct 224586 ATCTGTTCTTATCAGCTTAATATCTGATACGCTGTTCATTGAGCAGCTCATATATTAAACTGAT 224649

Query    101 TTTTGGAGCAGGGAGATGG-AATAGGAGCTTGCTCTGTCCACTCCACGCATCGACCTGGTATTG 163   
             ||||||| | |||   ||| || ||  ||||||   ||||   |||||  | | |  ||||| |       
Sbjct 224650 TTTTGGAACCGGGCTGTGGCAAAAGAGGCTTGCCTCGTCCCAGCCACGGGTTGCCTCGGTATAG 224713

Query    164 CAGTACCTCCAGGACCG 180   
             || |||||||  |||||       
Sbjct 224714 CACTACCTCC--GACCG 224728

 
                 
                
                 
                      Report:  
                     
                         
                             sequence start
                                   ?  
                                     
Start position of the estimated full-length sequence in genome.
Start index  
                                 :
                             
                             224549 
                         
                         
                             sequence end
                                   ?  
                                     
End position of the estimated full-length sequence in genome.
Start index  
                                 :
                             
                             224738 
                         
                         
                             bit score (CM)
                                   ?  
                                     
The score for aligning estimated full-length sequence to CM model
  (computed by RSEARCH -> default,
  infered from Rfam or provided by user) 
                                 :
                             
                             91.44 
                         
                         
                             Homology estimate
                                   ?  
                                     
Quick homology estimate:
  Not homologous: bit score   20 and bit score > 0.5 * query length
  Uncertain otherwise 
                                 :
                             
                             Uncertain  ↴  
                         
                     
                    
                     
                        Check the secondary structure and sequence viewer
                        for supporting information about possible homology.
                     
                    
                 
                
                
                 
                     
                          Estimated full-length sequence:  
                         
                          ? 
                             
Click checkbox to select multiple seuqences.
Fasta header format:
  UID|accession.versionSTRAND start-end 
                         
                     
                     >uid:71|NW_015441095.1fw 224549-224738
AUAUAUUUUCGAAUAAAAGUUUUAAAAUACAAAAAAAAUCUGUUCUUAUCAGCUUAAUAU
CUGAUACGCUGUUCAUUGAGCAGCUCAUAUAUUAAACUGAUUUUUGGAACCGGGCUGUGG
CAAAAGAGGCUUGCCUCGUCCCAGCCACGGGUUGCCUCGGUAUAGCACUACCUCCGACCG
CGGCCCAAUU
 
                 
                
             
            
             
                
                     
                         
                         
                             rnafold 
                             
                              ? 
                                 
Visualisation of predicted secondary structure.
To save the image:
  Right click on the image -> Save Image as. 
                             
                         
                     
                
                     
                         
                         
                             rfam-Rc 
                             
                              ? 
                                 
Visualisation of predicted secondary structure.
To save the image:
  Right click on the image -> Save Image as. 
                             
                         
                     
                
                     
                         
                         
                             TurboFold 
                             
                              ? 
                                 
Visualisation of predicted secondary structure.
To save the image:
  Right click on the image -> Save Image as. 
                             
                         
                     
                
             
            

        
             
                 Load Sequence viewer 
             
        
        
         
     
    

    
     
         
            Hit: NW_015441095.1
         
         
             
                 
                     NW_015441095.1 Acropora digitifera unplaced genomic scaffold, Adig_1.1 DF970730.1, whole genome shotgun sequence 
                     
                         
 ?  
This is BLAST alignment as read from the input file 
                         
 Score = 68.0 bits (62.6), Expect = 8.31E-08
 Identities = 34/34 (100%), Gaps = 0/34 (0%)
 Strand = Plus/Minus
Query      1 ATCGCTTCTCGGCCTTTTGGCTAAGATCAAGTGT 34    
             ||||||||||||||||||||||||||||||||||       
Sbjct 204118 ATCGCTTCTCGGCCTTTTGGCTAAGATCAAGTGT 204085

 
                 
                
                 
                      Report:  
                     
                         
                             sequence start
                                   ?  
                                     
Start position of the estimated full-length sequence in genome.
Start index  
                                 :
                             
                             203931 
                         
                         
                             sequence end
                                   ?  
                                     
End position of the estimated full-length sequence in genome.
Start index  
                                 :
                             
                             204130 
                         
                         
                             bit score (CM)
                                   ?  
                                     
The score for aligning estimated full-length sequence to CM model
  (computed by RSEARCH -> default,
  infered from Rfam or provided by user) 
                                 :
                             
                             20.13 
                         
                         
                             Homology estimate
                                   ?  
                                     
Quick homology estimate:
  Not homologous: bit score   20 and bit score > 0.5 * query length
  Uncertain otherwise 
                                 :
                             
                             Uncertain  ↴  
                         
                     
                    
                     
                        Check the secondary structure and sequence viewer
                        for supporting information about possible homology.
                     
                    
                 
                
                
                 
                     
                          Estimated full-length sequence:  
                         
                          ? 
                             
Click checkbox to select multiple seuqences.
Fasta header format:
  UID|accession.versionSTRAND start-end 
                         
                     
                     >uid:72|NW_015441095.1rc 203931-204130
AUCGCUUCUCGGCCUUUUGGCUAAGAUCAAGUGUUCUUGUAUCUUGCCAUUAGGAUUGUU
AGGUUAUUUUCUUCGCGUCCUGUGCUAAGUACCCGCCGGGGGGGAACGCCAUGGAGUCUC
CUCGUGUGAGUAGCGAUUCUUCACACGCUAUCGAGGCCAGUGCUGACGUCUUGGUGACGU
CAGCGGCACAUGGUUUGAGU
 
                 
                
             
            
             
                
                     
                         
                         
                             rnafold 
                             
                              ? 
                                 
Visualisation of predicted secondary structure.
To save the image:
  Right click on the image -> Save Image as. 
                             
                         
                     
                
                     
                         
                         
                             rfam-Rc 
                             
                              ? 
                                 
Visualisation of predicted secondary structure.
To save the image:
  Right click on the image -> Save Image as. 
                             
                         
                     
                
                     
                         
                         
                             TurboFold 
                             
                              ? 
                                 
Visualisation of predicted secondary structure.
To save the image:
  Right click on the image -> Save Image as. 
                             
                         
                     
                
             
            

        
             
                 Load Sequence viewer 
             
        
        
         
     
    

    
     
         
            Hit: NW_015441095.1
         
         
             
                 
                     NW_015441095.1 Acropora digitifera unplaced genomic scaffold, Adig_1.1 DF970730.1, whole genome shotgun sequence 
                     
                         
 ?  
This is BLAST alignment as read from the input file 
                         
 Score = 45.0 bits (41.9), Expect = 2.72E-01
 Identities = 24/25 (96%), Gaps = 0/25 (0%)
 Strand = Plus/Minus
Query      1 ATCGCTTCTCGGCCTTTTGGCTAAG 25    
             ||||||||||||||||||||| |||       
Sbjct 182313 ATCGCTTCTCGGCCTTTTGGCCAAG 182289

 
                 
                
                 
                      Report:  
                     
                         
                             sequence start
                                   ?  
                                     
Start position of the estimated full-length sequence in genome.
Start index  
                                 :
                             
                             182126 
                         
                         
                             sequence end
                                   ?  
                                     
End position of the estimated full-length sequence in genome.
Start index  
                                 :
                             
                             182302 
                         
                         
                             bit score (CM)
                                   ?  
                                     
The score for aligning estimated full-length sequence to CM model
  (computed by RSEARCH -> default,
  infered from Rfam or provided by user) 
                                 :
                             
                             -8.16 
                         
                         
                             Homology estimate
                                   ?  
                                     
Quick homology estimate:
  Not homologous: bit score   20 and bit score > 0.5 * query length
  Uncertain otherwise 
                                 :
                             
                             Not homologous   
                         
                     
                    
                 
                
                
                 
                     
                          Estimated full-length sequence:  
                         
                          ? 
                             
Click checkbox to select multiple seuqences.
Fasta header format:
  UID|accession.versionSTRAND start-end 
                         
                     
                     >uid:73|NW_015441095.1rc 182126-182302
AUCGCUUCUCGGCCUUUUGGCCAAGUUUUCUUUUUGUCUCGUUGCAGAAAGUGCUUCCAG
AUGUGAUGGAUACUGAUUCAGAGAUGAGGUUAAUAGAUGUACCGGAAAGAUCAACUGAAG
AUUUGGUGAUAACUGUGAAUACUGAAGAACGUACCAAGAACGCAUCCCGAUCCAGGG
 
                 
                
             
            
             
                
                     
                         
                         
                             rnafold 
                             
                              ? 
                                 
Visualisation of predicted secondary structure.
To save the image:
  Right click on the image -> Save Image as. 
                             
                         
                     
                
                     
                         
                         
                             rfam-Rc 
                             
                              ? 
                                 
Visualisation of predicted secondary structure.
To save the image:
  Right click on the image -> Save Image as. 
                             
                         
                     
                
                     
                         
                         
                             TurboFold 
                             
                              ? 
                                 
Visualisation of predicted secondary structure.
To save the image:
  Right click on the image -> Save Image as. 
                             
                         
                     
                
             
            

        
             
                 Load Sequence viewer 
             
        
        
         
     
    

    
     
         
            Hit: NW_015441095.1
         
         
             
                 
                     NW_015441095.1 Acropora digitifera unplaced genomic scaffold, Adig_1.1 DF970730.1, whole genome shotgun sequence 
                     
                         
 ?  
This is BLAST alignment as read from the input file 
                         
 Score = 41.0 bits (38.3), Expect = 3.31E+00
 Identities = 27/30 (90%), Gaps = 1/30 (3%)
 Strand = Plus/Plus
Query      6 TTCTCGGCCTTTTGGCTAAGAT-CAAGTGT 34    
             |||||||||||||||||||| | ||| |||       
Sbjct 222981 TTCTCGGCCTTTTGGCTAAGTTGCAATTGT 223010

 
                 
                
                 
                      Report:  
                     
                         
                             sequence start
                                   ?  
                                     
Start position of the estimated full-length sequence in genome.
Start index  
                                 :
                             
                             222976 
                         
                         
                             sequence end
                                   ?  
                                     
End position of the estimated full-length sequence in genome.
Start index  
                                 :
                             
                             223159 
                         
                         
                             bit score (CM)
                                   ?  
                                     
The score for aligning estimated full-length sequence to CM model
  (computed by RSEARCH -> default,
  infered from Rfam or provided by user) 
                                 :
                             
                             -1.83 
                         
                         
                             Homology estimate
                                   ?  
                                     
Quick homology estimate:
  Not homologous: bit score   20 and bit score > 0.5 * query length
  Uncertain otherwise 
                                 :
                             
                             Not homologous   
                         
                     
                    
                 
                
                
                 
                     
                          Estimated full-length sequence:  
                         
                          ? 
                             
Click checkbox to select multiple seuqences.
Fasta header format:
  UID|accession.versionSTRAND start-end 
                         
                     
                     >uid:74|NW_015441095.1fw 222976-223159
AUCUUUUCUCGGCCUUUUGGCUAAGUUGCAAUUGUUGACAGUUUACUGUUGAUUUUUCAG
AUUGUUCUGCCAUGAUGCCGCCGUCUGAGCUGUACCCGAAGAGCCUGCUGCUGCAUUUCC
CACCACAAGUGUACCAGAGAACCAGUCCAUCUGACCUGUUACCAAAGGUGUUAGGGAUUG
UCAA
 
                 
                
             
            
             
                
                     
                         
                         
                             rnafold 
                             
                              ? 
                                 
Visualisation of predicted secondary structure.
To save the image:
  Right click on the image -> Save Image as. 
                             
                         
                     
                
                     
                         
                         
                             rfam-Rc 
                             
                              ? 
                                 
Visualisation of predicted secondary structure.
To save the image:
  Right click on the image -> Save Image as. 
                             
                         
                     
                
                     
                         
                         
                             TurboFold 
                             
                              ? 
                                 
Visualisation of predicted secondary structure.
To save the image:
  Right click on the image -> Save Image as. 
                             
                         
                     
                
             
            

        
             
                 Load Sequence viewer 
             
        
        
         
     
    

    
     
         
            Hit: NW_015441189.1
         
         
             
                 
                     NW_015441189.1 Acropora digitifera unplaced genomic scaffold, Adig_1.1 DF970824.1, whole genome shotgun sequence 
                     
                         
 ?  
This is BLAST alignment as read from the input file 
                         
 Score = 164.0 bits (149.2), Expect = 7.28E-34
 Identities = 138/174 (79%), Gaps = 1/174 (1%)
 Strand = Plus/Minus
Query     1 ATCGCTTCTCGGCCTTTTGGCTAAGATCAAGTGTAGTATCTGTTCTTATCAGTTTAATATCTGATA 66   
            |||||||||||||||||||||||||||||||||||||||||||||||||||| |||||||||||||      
Sbjct 20729 ATCGCTTCTCGGCCTTTTGGCTAAGATCAAGTGTAGTATCTGTTCTTATCAGCTTAATATCTGATA 20664

Query    67 CG-TCCTCTATCCGAGGACAATATATTAAATGGATTTTTGGAGCAGGGAGATGGAATAGGAGCTTG 131  
            || | |||  |  |  |   ||||||||||  |||||||||| | |||   ||||| ||  |||||      
Sbjct 20663 CGCTGCTCATTGAGCAGCTCATATATTAAACTGATTTTTGGAACCGGGCTGTGGAAAAGAGGCTTG 20598

Query   132 CTCTGTCCACTCCACGCATCGACCTGGTATTGCAGTACCTCC 173  
            |   ||||   |||||  | | |  ||||| ||| |||||||      
Sbjct 20597 CCTCGTCCCAGCCACGGGTTGCCTCGGTATAGCACTACCTCC 20556

 
                 
                
                 
                      Report:  
                     
                         
                             sequence start
                                   ?  
                                     
Start position of the estimated full-length sequence in genome.
Start index  
                                 :
                             
                             20541 
                         
                         
                             sequence end
                                   ?  
                                     
End position of the estimated full-length sequence in genome.
Start index  
                                 :
                             
                             20729 
                         
                         
                             bit score (CM)
                                   ?  
                                     
The score for aligning estimated full-length sequence to CM model
  (computed by RSEARCH -> default,
  infered from Rfam or provided by user) 
                                 :
                             
                             161.93 
                         
                         
                             Homology estimate
                                   ?  
                                     
Quick homology estimate:
  Not homologous: bit score   20 and bit score > 0.5 * query length
  Uncertain otherwise 
                                 :
                             
                             Homologous   
                         
                     
                    
                 
                
                
                 
                     
                          Estimated full-length sequence:  
                         
                          ? 
                             
Click checkbox to select multiple seuqences.
Fasta header format:
  UID|accession.versionSTRAND start-end 
                         
                     
                     >uid:75|NW_015441189.1rc 20541-20729
AUCGCUUCUCGGCCUUUUGGCUAAGAUCAAGUGUAGUAUCUGUUCUUAUCAGCUUAAUAU
CUGAUACGCUGCUCAUUGAGCAGCUCAUAUAUUAAACUGAUUUUUGGAACCGGGCUGUGG
AAAAGAGGCUUGCCUCGUCCCAGCCACGGGUUGCCUCGGUAUAGCACUACCUCCGAGCGC
GGCCCACUU
 
                 
                
             
            
             
                
                     
                         
                         
                             rnafold 
                             
                              ? 
                                 
Visualisation of predicted secondary structure.
To save the image:
  Right click on the image -> Save Image as. 
                             
                         
                     
                
                     
                         
                         
                             rfam-Rc 
                             
                              ? 
                                 
Visualisation of predicted secondary structure.
To save the image:
  Right click on the image -> Save Image as. 
                             
                         
                     
                
                     
                         
                         
                             TurboFold 
                             
                              ? 
                                 
Visualisation of predicted secondary structure.
To save the image:
  Right click on the image -> Save Image as. 
                             
                         
                     
                
             
            

        
             
                 Load Sequence viewer 
             
        
        
         
     
    

    
     
         
            Hit: NW_015441189.1
         
         
             
                 
                     NW_015441189.1 Acropora digitifera unplaced genomic scaffold, Adig_1.1 DF970824.1, whole genome shotgun sequence 
                     
                         
 ?  
This is BLAST alignment as read from the input file 
                         
 Score = 164.0 bits (149.2), Expect = 7.28E-34
 Identities = 138/174 (79%), Gaps = 1/174 (1%)
 Strand = Plus/Minus
Query     1 ATCGCTTCTCGGCCTTTTGGCTAAGATCAAGTGTAGTATCTGTTCTTATCAGTTTAATATCTGATA 66   
            |||||||||||||||||||||||||||||||||||||||||||||||||||| |||||||||||||      
Sbjct 68374 ATCGCTTCTCGGCCTTTTGGCTAAGATCAAGTGTAGTATCTGTTCTTATCAGCTTAATATCTGATA 68309

Query    67 CG-TCCTCTATCCGAGGACAATATATTAAATGGATTTTTGGAGCAGGGAGATGGAATAGGAGCTTG 131  
            || | |||  |  |  |   ||||||||||  |||||||||| | |||   ||||| ||  |||||      
Sbjct 68308 CGCTGCTCATTGAGCAGCTCATATATTAAACTGATTTTTGGAACCGGGCTGTGGAAAAGAGGCTTG 68243

Query   132 CTCTGTCCACTCCACGCATCGACCTGGTATTGCAGTACCTCC 173  
            |   ||||   |||||  | | |  ||||| ||| |||||||      
Sbjct 68242 CCTCGTCCCAGCCACGGGTTGCCTCGGTATAGCACTACCTCC 68201

 
                 
                
                 
                      Report:  
                     
                         
                             sequence start
                                   ?  
                                     
Start position of the estimated full-length sequence in genome.
Start index  
                                 :
                             
                             68186 
                         
                         
                             sequence end
                                   ?  
                                     
End position of the estimated full-length sequence in genome.
Start index  
                                 :
                             
                             68374 
                         
                         
                             bit score (CM)
                                   ?  
                                     
The score for aligning estimated full-length sequence to CM model
  (computed by RSEARCH -> default,
  infered from Rfam or provided by user) 
                                 :
                             
                             161.93 
                         
                         
                             Homology estimate
                                   ?  
                                     
Quick homology estimate:
  Not homologous: bit score   20 and bit score > 0.5 * query length
  Uncertain otherwise 
                                 :
                             
                             Homologous   
                         
                     
                    
                 
                
                
                 
                     
                          Estimated full-length sequence:  
                         
                          ? 
                             
Click checkbox to select multiple seuqences.
Fasta header format:
  UID|accession.versionSTRAND start-end 
                         
                     
                     >uid:76|NW_015441189.1rc 68186-68374
AUCGCUUCUCGGCCUUUUGGCUAAGAUCAAGUGUAGUAUCUGUUCUUAUCAGCUUAAUAU
CUGAUACGCUGCUCAUUGAGCAGCUCAUAUAUUAAACUGAUUUUUGGAACCGGGCUGUGG
AAAAGAGGCUUGCCUCGUCCCAGCCACGGGUUGCCUCGGUAUAGCACUACCUCCGAGCGC
GGCCCACUU
 
                 
                
             
            
             
                
                     
                         
                         
                             rnafold 
                             
                              ? 
                                 
Visualisation of predicted secondary structure.
To save the image:
  Right click on the image -> Save Image as. 
                             
                         
                     
                
                     
                         
                         
                             rfam-Rc 
                             
                              ? 
                                 
Visualisation of predicted secondary structure.
To save the image:
  Right click on the image -> Save Image as. 
                             
                         
                     
                
                     
                         
                         
                             TurboFold 
                             
                              ? 
                                 
Visualisation of predicted secondary structure.
To save the image:
  Right click on the image -> Save Image as. 
                             
                         
                     
                
             
            

        
             
                 Load Sequence viewer 
             
        
        
         
     
    

    
     
         
            Hit: NW_015441189.1
         
         
             
                 
                     NW_015441189.1 Acropora digitifera unplaced genomic scaffold, Adig_1.1 DF970824.1, whole genome shotgun sequence 
                     
                         
 ?  
This is BLAST alignment as read from the input file 
                         
 Score = 162.0 bits (147.4), Expect = 2.54E-33
 Identities = 137/173 (79%), Gaps = 1/173 (1%)
 Strand = Plus/Minus
Query     1 ATCGCTTCTCGGCCTTTTGGCTAAGATCAAGTGTAGTATCTGTTCTTATCAGTTTAATATCTGATA 66   
            |||||||||||||||||||||||||||||||||||||||||||||||||||| |||||||||||||      
Sbjct 18925 ATCGCTTCTCGGCCTTTTGGCTAAGATCAAGTGTAGTATCTGTTCTTATCAGCTTAATATCTGATA 18860

Query    67 CG-TCCTCTATCCGAGGACAATATATTAAATGGATTTTTGGAGCAGGGAGATGGAATAGGAGCTTG 131  
            || | |||  |  |  |   ||||||||||  |||||||||| | |||   ||||| ||  |||||      
Sbjct 18859 CGCTGCTCATTGAGCAGCTCATATATTAAACTGATTTTTGGAACCGGGCTGTGGAAAAGAGGCTTG 18794

Query   132 CTCTGTCCACTCCACGCATCGACCTGGTATTGCAGTACCTC 172  
            |   ||||   |||||  | | |  ||||| ||| ||||||      
Sbjct 18793 CCTCGTCCCAGCCACGGGTTGCCTCGGTATAGCACTACCTC 18753

 
                 
                
                 
                      Report:  
                     
                         
                             sequence start
                                   ?  
                                     
Start position of the estimated full-length sequence in genome.
Start index  
                                 :
                             
                             18737 
                         
                         
                             sequence end
                                   ?  
                                     
End position of the estimated full-length sequence in genome.
Start index  
                                 :
                             
                             18925 
                         
                         
                             bit score (CM)
                                   ?  
                                     
The score for aligning estimated full-length sequence to CM model
  (computed by RSEARCH -> default,
  infered from Rfam or provided by user) 
                                 :
                             
                             158.07 
                         
                         
                             Homology estimate
                                   ?  
                                     
Quick homology estimate:
  Not homologous: bit score   20 and bit score > 0.5 * query length
  Uncertain otherwise 
                                 :
                             
                             Homologous   
                         
                     
                    
                 
                
                
                 
                     
                          Estimated full-length sequence:  
                         
                          ? 
                             
Click checkbox to select multiple seuqences.
Fasta header format:
  UID|accession.versionSTRAND start-end 
                         
                     
                     >uid:77|NW_015441189.1rc 18737-18925
AUCGCUUCUCGGCCUUUUGGCUAAGAUCAAGUGUAGUAUCUGUUCUUAUCAGCUUAAUAU
CUGAUACGCUGCUCAUUGAGCAGCUCAUAUAUUAAACUGAUUUUUGGAACCGGGCUGUGG
AAAAGAGGCUUGCCUCGUCCCAGCCACGGGUUGCCUCGGUAUAGCACUACCUCUGAGCGC
GGCCCACUU
 
                 
                
             
            
             
                
                     
                         
                         
                             rnafold 
                             
                              ? 
                                 
Visualisation of predicted secondary structure.
To save the image:
  Right click on the image -> Save Image as. 
                             
                         
                     
                
                     
                         
                         
                             rfam-Rc 
                             
                              ? 
                                 
Visualisation of predicted secondary structure.
To save the image:
  Right click on the image -> Save Image as. 
                             
                         
                     
                
                     
                         
                         
                             TurboFold 
                             
                              ? 
                                 
Visualisation of predicted secondary structure.
To save the image:
  Right click on the image -> Save Image as. 
                             
                         
                     
                
             
            

        
             
                 Load Sequence viewer 
             
        
        
         
     
    

    
     
         
            Hit: NW_015441189.1
         
         
             
                 
                     NW_015441189.1 Acropora digitifera unplaced genomic scaffold, Adig_1.1 DF970824.1, whole genome shotgun sequence 
                     
                         
 ?  
This is BLAST alignment as read from the input file 
                         
 Score = 80.0 bits (73.4), Expect = 4.60E-11
 Identities = 99/137 (72%), Gaps = 1/137 (1%)
 Strand = Plus/Minus
Query    38 ATCTGTTCTTATCAGTTTAATATCTGATACG-TCCTCTATCCGAGGACAATATATTAAATGGATTT 102  
            ||| ||||||||||| ||||||||||||||| | |||  |  |  |   ||||||| ||  |||||      
Sbjct 73719 ATCAGTTCTTATCAGCTTAATATCTGATACGCTGCTCATTGAGCAGCTCATATATTGAACTGATTT 73654

Query   103 TTGGAGCAGGGAGATGGAATAGGAGCTTGCTCTGTCCACTCCACGCATCGACCTGGTATTGCAGTA 168  
            ||||| | |||   ||||| ||  ||||||   ||||   |||||  | | |  ||||| ||| ||      
Sbjct 73653 TTGGAACCGGGCTGTGGAAAAGAGGCTTGCCTCGTCCCAGCCACGGGTTGCCTCGGTATAGCACTA 73588

Query   169 CCTCC 173  
            |||||      
Sbjct 73587 CCTCC 73583

 
                 
                
                 
                      Report:  
                     
                         
                             sequence start
                                   ?  
                                     
Start position of the estimated full-length sequence in genome.
Start index  
                                 :
                             
                             73568 
                         
                         
                             sequence end
                                   ?  
                                     
End position of the estimated full-length sequence in genome.
Start index  
                                 :
                             
                             73756 
                         
                         
                             bit score (CM)
                                   ?  
                                     
The score for aligning estimated full-length sequence to CM model
  (computed by RSEARCH -> default,
  infered from Rfam or provided by user) 
                                 :
                             
                             106.81 
                         
                         
                             Homology estimate
                                   ?  
                                     
Quick homology estimate:
  Not homologous: bit score   20 and bit score > 0.5 * query length
  Uncertain otherwise 
                                 :
                             
                             Homologous   
                         
                     
                    
                 
                
                
                 
                     
                          Estimated full-length sequence:  
                         
                          ? 
                             
Click checkbox to select multiple seuqences.
Fasta header format:
  UID|accession.versionSTRAND start-end 
                         
                     
                     >uid:78|NW_015441189.1rc 73568-73756
CCUUUGUCACGGUUGUAAAUAAACUUUUAUAAAAAAAAUCAGUUCUUAUCAGCUUAAUAU
CUGAUACGCUGCUCAUUGAGCAGCUCAUAUAUUGAACUGAUUUUUGGAACCGGGCUGUGG
AAAAGAGGCUUGCCUCGUCCCAGCCACGGGUUGCCUCGGUAUAGCACUACCUCCGAGCGC
GGCCCACUU
 
                 
                
             
            
             
                
                     
                         
                         
                             rnafold 
                             
                              ? 
                                 
Visualisation of predicted secondary structure.
To save the image:
  Right click on the image -> Save Image as. 
                             
                         
                     
                
                     
                         
                         
                             rfam-Rc 
                             
                              ? 
                                 
Visualisation of predicted secondary structure.
To save the image:
  Right click on the image -> Save Image as. 
                             
                         
                     
                
                     
                         
                         
                             TurboFold 
                             
                              ? 
                                 
Visualisation of predicted secondary structure.
To save the image:
  Right click on the image -> Save Image as. 
                             
                         
                     
                
             
            

        
             
                 Load Sequence viewer 
             
        
        
         
     
    

    
     
         
            Hit: NW_015441189.1
         
         
             
                 
                     NW_015441189.1 Acropora digitifera unplaced genomic scaffold, Adig_1.1 DF970824.1, whole genome shotgun sequence 
                     
                         
 ?  
This is BLAST alignment as read from the input file 
                         
 Score = 45.0 bits (41.9), Expect = 2.72E-01
 Identities = 24/25 (96%), Gaps = 0/25 (0%)
 Strand = Plus/Minus
Query     1 ATCGCTTCTCGGCCTTTTGGCTAAG 25   
            ||||||||||||||||||| |||||      
Sbjct 67133 ATCGCTTCTCGGCCTTTTGACTAAG 67109

 
                 
                
                 
                      Report:  
                     
                         
                             sequence start
                                   ?  
                                     
Start position of the estimated full-length sequence in genome.
Start index  
                                 :
                             
                             66946 
                         
                         
                             sequence end
                                   ?  
                                     
End position of the estimated full-length sequence in genome.
Start index  
                                 :
                             
                             67134 
                         
                         
                             bit score (CM)
                                   ?  
                                     
The score for aligning estimated full-length sequence to CM model
  (computed by RSEARCH -> default,
  infered from Rfam or provided by user) 
                                 :
                             
                             -12.78 
                         
                         
                             Homology estimate
                                   ?  
                                     
Quick homology estimate:
  Not homologous: bit score   20 and bit score > 0.5 * query length
  Uncertain otherwise 
                                 :
                             
                             Not homologous   
                         
                     
                    
                 
                
                
                 
                     
                          Estimated full-length sequence:  
                         
                          ? 
                             
Click checkbox to select multiple seuqences.
Fasta header format:
  UID|accession.versionSTRAND start-end 
                         
                     
                     >uid:79|NW_015441189.1rc 66946-67134
AUCGCUUCUCGGCCUUUUGACUAAGGCCUUCUUUUGCUUUUGCAGAGAGAGAUCCUUGCA
GCGAUGGAAGAUGAUGAGGAGAAUCGCUUUGUUAAUAUGCCUGAAAGACAGACCGAAGCU
GUGGAAGUUACUGUACCUGUGAUUCCAGAACGUACCGAUGACGUUUGCAGGUCCUGGGCU
GAACGAACU
 
                 
                
             
            
             
                
                     
                         
                         
                             rnafold 
                             
                              ? 
                                 
Visualisation of predicted secondary structure.
To save the image:
  Right click on the image -> Save Image as. 
                             
                         
                     
                
                     
                         
                         
                             rfam-Rc 
                             
                              ? 
                                 
Visualisation of predicted secondary structure.
To save the image:
  Right click on the image -> Save Image as. 
                             
                         
                     
                
                     
                         
                         
                             TurboFold 
                             
                              ? 
                                 
Visualisation of predicted secondary structure.
To save the image:
  Right click on the image -> Save Image as. 
                             
                         
                     
                
             
            

        
             
                 Load Sequence viewer 
             
        
        
         
     
    

    
     
         
            Hit: NW_015441189.1
         
         
             
                 
                     NW_015441189.1 Acropora digitifera unplaced genomic scaffold, Adig_1.1 DF970824.1, whole genome shotgun sequence 
                     
                         
 ?  
This is BLAST alignment as read from the input file 
                         
 Score = 44.0 bits (41.0), Expect = 2.72E-01
 Identities = 22/22 (100%), Gaps = 0/22 (0%)
 Strand = Plus/Minus
Query     1 ATCGCTTCTCGGCCTTTTGGCT 22   
            ||||||||||||||||||||||      
Sbjct 78458 ATCGCTTCTCGGCCTTTTGGCT 78437

 
                 
                
                 
                      Report:  
                     
                         
                             sequence start
                                   ?  
                                     
Start position of the estimated full-length sequence in genome.
Start index  
                                 :
                             
                             78271 
                         
                         
                             sequence end
                                   ?  
                                     
End position of the estimated full-length sequence in genome.
Start index  
                                 :
                             
                             78449 
                         
                         
                             bit score (CM)
                                   ?  
                                     
The score for aligning estimated full-length sequence to CM model
  (computed by RSEARCH -> default,
  infered from Rfam or provided by user) 
                                 :
                             
                             8.94 
                         
                         
                             Homology estimate
                                   ?  
                                     
Quick homology estimate:
  Not homologous: bit score   20 and bit score > 0.5 * query length
  Uncertain otherwise 
                                 :
                             
                             Uncertain  ↴  
                         
                     
                    
                     
                        Check the secondary structure and sequence viewer
                        for supporting information about possible homology.
                     
                    
                 
                
                
                 
                     
                          Estimated full-length sequence:  
                         
                          ? 
                             
Click checkbox to select multiple seuqences.
Fasta header format:
  UID|accession.versionSTRAND start-end 
                         
                     
                     >uid:80|NW_015441189.1rc 78271-78449
AUCGCUUCUCGGCCUUUUGGCUGGAUCCAGAAGGCUUUUUUGCUUUUAUCUCCAUAGAUU
GCCACGAUGUCUGACAGCAAUUUGAUGGAGAUUUUACAAGAUGUUGAAUCGCCUGUUUUG
AUUAGCGGAGAGAGUACUUUGGUUCCAGAAGAUGAACAUGCAAUUGCAGAUGAAGACCU
 
                 
                
             
            
             
                
                     
                         
                         
                             rnafold 
                             
                              ? 
                                 
Visualisation of predicted secondary structure.
To save the image:
  Right click on the image -> Save Image as. 
                             
                         
                     
                
                     
                         
                         
                             rfam-Rc 
                             
                              ? 
                                 
Visualisation of predicted secondary structure.
To save the image:
  Right click on the image -> Save Image as. 
                             
                         
                     
                
                     
                         
                         
                             TurboFold 
                             
                              ? 
                                 
Visualisation of predicted secondary structure.
To save the image:
  Right click on the image -> Save Image as. 
                             
                         
                     
                
             
            

        
             
                 Load Sequence viewer 
             
        
        
         
     
    

    
     
         
            Hit: NW_015441189.1
         
         
             
                 
                     NW_015441189.1 Acropora digitifera unplaced genomic scaffold, Adig_1.1 DF970824.1, whole genome shotgun sequence 
                     
                         
 ?  
This is BLAST alignment as read from the input file 
                         
 Score = 43.0 bits (40.1), Expect = 9.49E-01
 Identities = 23/24 (96%), Gaps = 0/24 (0%)
 Strand = Plus/Minus
Query     1 ATCGCTTCTCGGCCTTTTGGCTAA 24   
            ||||||||| ||||||||||||||      
Sbjct 15159 ATCGCTTCTTGGCCTTTTGGCTAA 15136

 
                 
                
                 
                      Report:  
                     
                         
                             sequence start
                                   ?  
                                     
Start position of the estimated full-length sequence in genome.
Start index  
                                 :
                             
                             14972 
                         
                         
                             sequence end
                                   ?  
                                     
End position of the estimated full-length sequence in genome.
Start index  
                                 :
                             
                             15165 
                         
                         
                             bit score (CM)
                                   ?  
                                     
The score for aligning estimated full-length sequence to CM model
  (computed by RSEARCH -> default,
  infered from Rfam or provided by user) 
                                 :
                             
                             -8.53 
                         
                         
                             Homology estimate
                                   ?  
                                     
Quick homology estimate:
  Not homologous: bit score   20 and bit score > 0.5 * query length
  Uncertain otherwise 
                                 :
                             
                             Not homologous   
                         
                     
                    
                 
                
                
                 
                     
                          Estimated full-length sequence:  
                         
                          ? 
                             
Click checkbox to select multiple seuqences.
Fasta header format:
  UID|accession.versionSTRAND start-end 
                         
                     
                     >uid:81|NW_015441189.1rc 14972-15165
AUCGCUUCUUGGCCUUUUGGCUAACUUCCGAAAGGUCCGCUUUGCUAUCCUUUUUAUCUC
CAUAGAUUAUCAUGGCAAGGACAGCUAUGGAGACGUCUGAAGACCUUCAAGUUCAAGAUG
CACAGGAAAAACAAAUUUGGAGUGAAUAAAUGGAACAACAAGUUCAGGAAAAUGCUAAUG
CUUGCGAACCACAG
 
                 
                
             
            
             
                
                     
                         
                         
                             rnafold 
                             
                              ? 
                                 
Visualisation of predicted secondary structure.
To save the image:
  Right click on the image -> Save Image as. 
                             
                         
                     
                
                     
                         
                         
                             rfam-Rc 
                             
                              ? 
                                 
Visualisation of predicted secondary structure.
To save the image:
  Right click on the image -> Save Image as. 
                             
                         
                     
                
                     
                         
                         
                             TurboFold 
                             
                              ? 
                                 
Visualisation of predicted secondary structure.
To save the image:
  Right click on the image -> Save Image as. 
                             
                         
                     
                
             
            

        
             
                 Load Sequence viewer 
             
        
        
         
     
    

    
     
         
            Hit: NW_015441189.1
         
         
             
                 
                     NW_015441189.1 Acropora digitifera unplaced genomic scaffold, Adig_1.1 DF970824.1, whole genome shotgun sequence 
                     
                         
 ?  
This is BLAST alignment as read from the input file 
                         
 Score = 40.0 bits (37.4), Expect = 3.31E+00
 Identities = 23/25 (92%), Gaps = 0/25 (0%)
 Strand = Plus/Minus
Query     4 GCTTCTCGGCCTTTTGGCTAAGATC 28   
            |||||||||||||||| ||||| ||      
Sbjct 49781 GCTTCTCGGCCTTTTGACTAAGGTC 49757

 
                 
                
                 
                      Report:  
                     
                         
                             sequence start
                                   ?  
                                     
Start position of the estimated full-length sequence in genome.
Start index  
                                 :
                             
                             49597 
                         
                         
                             sequence end
                                   ?  
                                     
End position of the estimated full-length sequence in genome.
Start index  
                                 :
                             
                             49774 
                         
                         
                             bit score (CM)
                                   ?  
                                     
The score for aligning estimated full-length sequence to CM model
  (computed by RSEARCH -> default,
  infered from Rfam or provided by user) 
                                 :
                             
                             -8.73 
                         
                         
                             Homology estimate
                                   ?  
                                     
Quick homology estimate:
  Not homologous: bit score   20 and bit score > 0.5 * query length
  Uncertain otherwise 
                                 :
                             
                             Not homologous   
                         
                     
                    
                 
                
                
                 
                     
                          Estimated full-length sequence:  
                         
                          ? 
                             
Click checkbox to select multiple seuqences.
Fasta header format:
  UID|accession.versionSTRAND start-end 
                         
                     
                     >uid:82|NW_015441189.1rc 49597-49774
GUAGCUUCUCGGCCUUUUGACUAAGGUCGGUGUUUUCUUGCUUUUGCAGAAAGAUGGCUG
AGGCGAUAGAUCCAGAUGAGGAAAAUACAAUGGUAGACGUGCCAGAAAGAGAAGAUUUGG
UGAUUACAGUAAACCGUGAACGUACCAGUGACGAAUCAGGAAGUUGGGCCGAGCGGGU
 
                 
                
             
            
             
                
                     
                         
                         
                             rnafold 
                             
                              ? 
                                 
Visualisation of predicted secondary structure.
To save the image:
  Right click on the image -> Save Image as. 
                             
                         
                     
                
                     
                         
                         
                             rfam-Rc 
                             
                              ? 
                                 
Visualisation of predicted secondary structure.
To save the image:
  Right click on the image -> Save Image as. 
                             
                         
                     
                
                     
                         
                         
                             TurboFold 
                             
                              ? 
                                 
Visualisation of predicted secondary structure.
To save the image:
  Right click on the image -> Save Image as. 
                             
                         
                     
                
             
            

        
             
                 Load Sequence viewer 
             
        
        
         
     
    

    
     
         
            Hit: NW_015441241.1
         
         
             
                 
                     NW_015441241.1 Acropora digitifera unplaced genomic scaffold, Adig_1.1 DF970876.1, whole genome shotgun sequence 
                     
                         
 ?  
This is BLAST alignment as read from the input file 
                         
 Score = 164.0 bits (149.2), Expect = 7.28E-34
 Identities = 138/174 (79%), Gaps = 1/174 (1%)
 Strand = Plus/Plus
Query      1 ATCGCTTCTCGGCCTTTTGGCTAAGATCAAGTGTAGTATCTGTTCTTATCAGTTTAATATCTGA 64    
             |||||||||||||||||||||||||||||||||||||||||||||||||||| |||||||||||       
Sbjct 198650 ATCGCTTCTCGGCCTTTTGGCTAAGATCAAGTGTAGTATCTGTTCTTATCAGCTTAATATCTGA 198713

Query     65 TACG-TCCTCTATCCGAGGACAATATATTAAATGGATTTTTGGAGCAGGGAGATGGAATAGGAG 127   
             |||| | |||  |  |  |   ||||||||||  |||||||||| | |||   ||||| ||  |       
Sbjct 198714 TACGCTGCTCATTGAGCAGCTCATATATTAAACTGATTTTTGGAACCGGGCTGTGGAAAAGAGG 198777

Query    128 CTTGCTCTGTCCACTCCACGCATCGACCTGGTATTGCAGTACCTCC 173   
             |||||   ||||   |||||  | | |  ||||| ||| |||||||       
Sbjct 198778 CTTGCCTCGTCCCAGCCACGGGTTGCCTCGGTATAGCACTACCTCC 198823

 
                 
                
                 
                      Report:  
                     
                         
                             sequence start
                                   ?  
                                     
Start position of the estimated full-length sequence in genome.
Start index  
                                 :
                             
                             198650 
                         
                         
                             sequence end
                                   ?  
                                     
End position of the estimated full-length sequence in genome.
Start index  
                                 :
                             
                             198838 
                         
                         
                             bit score (CM)
                                   ?  
                                     
The score for aligning estimated full-length sequence to CM model
  (computed by RSEARCH -> default,
  infered from Rfam or provided by user) 
                                 :
                             
                             161.93 
                         
                         
                             Homology estimate
                                   ?  
                                     
Quick homology estimate:
  Not homologous: bit score   20 and bit score > 0.5 * query length
  Uncertain otherwise 
                                 :
                             
                             Homologous
[truncated: 2,142,707 more chars]
