# Supplementary material for: rboAnalyzer: A Software to Improve Characterization of Non-coding RNAs From Sequence Database Search Output
Source: Front Genet. 2020 Jul 28;11:675. doi: 10.3389/fgene.2020.00675 (PMC7401326; doi:10.3389/fgene.2020.00675)
Supplement: Supplementary file 5 [file Data_Sheet_4.ZIP › WUXKWAHV014-Alignment_new.html]

rboAnalyzer


## Please wait while sequence viewer is loading...

# rboAnalyzer report

```
BLAST output file:   WUXKWAHV014-Alignment.xml
Query sequence file: MYB_Hs.fa
```

### Hit: NG\_012330.1

NG\_012330.1 Homo sapiens MYB proto-oncogene, transcription factor (MYB), RefSeqGene on chromosome 6

```
?

```
This is BLAST alignment as read from the input file
```

Score = 300.0 bits (271.8), Expect = 1.26E-70
 Identities = 150/150 (100%), Gaps = 0/150 (0%)
 Strand = Plus/Plus
Query    1 ATATCAACCTGTTTCCTCCTCCTCCTTCTCCTCCTCCTCCGTGACCTCCTCCTCCTCTTTCTCCTGAG 68  
           ||||||||||||||||||||||||||||||||||||||||||||||||||||||||||||||||||||     
Sbjct 5002 ATATCAACCTGTTTCCTCCTCCTCCTTCTCCTCCTCCTCCGTGACCTCCTCCTCCTCTTTCTCCTGAG 5069

Query   69 AAACTTCGCCCCAGCGGTGCGGAGCGCCGCTGCGCAGCCGGGGAGGGACGCAGGCAGGCGGCGGGCAG 136 
           ||||||||||||||||||||||||||||||||||||||||||||||||||||||||||||||||||||     
Sbjct 5070 AAACTTCGCCCCAGCGGTGCGGAGCGCCGCTGCGCAGCCGGGGAGGGACGCAGGCAGGCGGCGGGCAG 5137

Query  137 CGGGAGGCGGCAGC 150 
           ||||||||||||||     
Sbjct 5138 CGGGAGGCGGCAGC 5151
```

Report:

|  |  |
| --- | --- |
| sequence start ?  ``` Start position of the estimated full-length sequence in genome. Start index < end index. ``` : | 5002 |
| sequence end ?  ``` End position of the estimated full-length sequence in genome. Start index < end index. ``` : | 5151 |
| bit score (CM) ?  ``` The score for aligning estimated full-length sequence to CM model   (computed by RSEARCH -> default,   infered from Rfam or provided by user) ``` : | 196.1 |
| Homology estimate ?  ``` Quick homology estimate:   Not homologous: bit score < 0   Homologous: bit score > 20 and bit score > 0.5 * query length   Uncertain otherwise ``` : | Homologous |

Estimated full-length sequence:


?

```
Click checkbox to select multiple seuqences.
Fasta header format:
  UID|accession.versionSTRAND start-end
```

>uid:0|NG\_012330.1fw 5002-5151
AUAUCAACCUGUUUCCUCCUCCUCCUUCUCCUCCUCCUCCGUGACCUCCUCCUCCUCUUU
CUCCUGAGAAACUUCGCCCCAGCGGUGCGGAGCGCCGCUGCGCAGCCGGGGAGGGACGCA
GGCAGGCGGCGGGCAGCGGGAGGCGGCAGC

rnafold


?

```
Visualisation of predicted secondary structure.
To save the image:
  Right click on the image -> Save Image as.
```


Turbo-fast


?

```
Visualisation of predicted secondary structure.
To save the image:
  Right click on the image -> Save Image as.
```

Load Sequence viewer

Turbo-fast: Number of sequences is less then required. n=3 (4)

### Hit: NC\_000006.12

NC\_000006.12 Homo sapiens chromosome 6, GRCh38.p13 Primary Assembly

```
?

```
This is BLAST alignment as read from the input file
```

Score = 300.0 bits (271.8), Expect = 1.26E-70
 Identities = 150/150 (100%), Gaps = 0/150 (0%)
 Strand = Plus/Plus
Query         1 ATATCAACCTGTTTCCTCCTCCTCCTTCTCCTCCTCCTCCGTGACCTCCTCCTCCTCT 58       
                ||||||||||||||||||||||||||||||||||||||||||||||||||||||||||          
Sbjct 135181316 ATATCAACCTGTTTCCTCCTCCTCCTTCTCCTCCTCCTCCGTGACCTCCTCCTCCTCT 135181373

Query        59 TTCTCCTGAGAAACTTCGCCCCAGCGGTGCGGAGCGCCGCTGCGCAGCCGGGGAGGGA 116      
                ||||||||||||||||||||||||||||||||||||||||||||||||||||||||||          
Sbjct 135181374 TTCTCCTGAGAAACTTCGCCCCAGCGGTGCGGAGCGCCGCTGCGCAGCCGGGGAGGGA 135181431

Query       117 CGCAGGCAGGCGGCGGGCAGCGGGAGGCGGCAGC 150      
                ||||||||||||||||||||||||||||||||||          
Sbjct 135181432 CGCAGGCAGGCGGCGGGCAGCGGGAGGCGGCAGC 135181465
```

Report:

|  |  |
| --- | --- |
| sequence start ?  ``` Start position of the estimated full-length sequence in genome. Start index < end index. ``` : | 135181316 |
| sequence end ?  ``` End position of the estimated full-length sequence in genome. Start index < end index. ``` : | 135181465 |
| bit score (CM) ?  ``` The score for aligning estimated full-length sequence to CM model   (computed by RSEARCH -> default,   infered from Rfam or provided by user) ``` : | 196.1 |
| Homology estimate ?  ``` Quick homology estimate:   Not homologous: bit score < 0   Homologous: bit score > 20 and bit score > 0.5 * query length   Uncertain otherwise ``` : | Homologous |

Estimated full-length sequence:


?

```
Click checkbox to select multiple seuqences.
Fasta header format:
  UID|accession.versionSTRAND start-end
```

>uid:1|NC\_000006.12fw 135181316-135181465
AUAUCAACCUGUUUCCUCCUCCUCCUUCUCCUCCUCCUCCGUGACCUCCUCCUCCUCUUU
CUCCUGAGAAACUUCGCCCCAGCGGUGCGGAGCGCCGCUGCGCAGCCGGGGAGGGACGCA
GGCAGGCGGCGGGCAGCGGGAGGCGGCAGC

rnafold


?

```
Visualisation of predicted secondary structure.
To save the image:
  Right click on the image -> Save Image as.
```


Turbo-fast


?

```
Visualisation of predicted secondary structure.
To save the image:
  Right click on the image -> Save Image as.
```

Load Sequence viewer

Turbo-fast: Number of sequences is less then required. n=3 (4)

### Hit: NC\_000006.12

NC\_000006.12 Homo sapiens chromosome 6, GRCh38.p13 Primary Assembly

```
?

```
This is BLAST alignment as read from the input file
```

Score = 41.0 bits (38.3), Expect = 4.69E+00
 Identities = 39/49 (80%), Gaps = 3/49 (6%)
 Strand = Plus/Plus
Query       102 GCAGCCGGGGAGGGACGCAGGCAGGCGGCGGGCAGCGGGAGGC-GGCAG 149      
                |||| | || ||| | ||||||||||||| ||||  || |||| |||||          
Sbjct 161219891 GCAGGCAGGCAGGCAGGCAGGCAGGCGGCAGGCA--GGCAGGCAGGCAG 161219937
```

Report:

|  |  |
| --- | --- |
| sequence start ?  ``` Start position of the estimated full-length sequence in genome. Start index < end index. ``` : | 161219790 |
| sequence end ?  ``` End position of the estimated full-length sequence in genome. Start index < end index. ``` : | 161219938 |
| bit score (CM) ?  ``` The score for aligning estimated full-length sequence to CM model   (computed by RSEARCH -> default,   infered from Rfam or provided by user) ``` : | -23.2 |
| Homology estimate ?  ``` Quick homology estimate:   Not homologous: bit score < 0   Homologous: bit score > 20 and bit score > 0.5 * query length   Uncertain otherwise ``` : | Not homologous |

Estimated full-length sequence:


?

```
Click checkbox to select multiple seuqences.
Fasta header format:
  UID|accession.versionSTRAND start-end
```

>uid:2|NC\_000006.12fw 161219790-161219938
CAGGGACACAGAUUCACAGUAAAAAAGAUAGACAGAGAUAGAUAGAUAGAUAGAUAGAUA
GAUAGAUAGAUAGAUAGAUAGAUAGGCAGGCAGGCAGGCAGGCAGGCAGGCAGGCAGGCA
GGCAGGCGGCAGGCAGGCAGGCAGGCAGG

rnafold


?

```
Visualisation of predicted secondary structure.
To save the image:
  Right click on the image -> Save Image as.
```


Turbo-fast


?

```
Visualisation of predicted secondary structure.
To save the image:
  Right click on the image -> Save Image as.
```

Load Sequence viewer

### Hit: NT\_025741.16

NT\_025741.16 Homo sapiens chromosome 6 genomic scaffold, GRCh38.p13 Primary Assembly HSCHR6\_CTG5

```
?

```
This is BLAST alignment as read from the input file
```

Score = 300.0 bits (271.8), Expect = 1.26E-70
 Identities = 150/150 (100%), Gaps = 0/150 (0%)
 Strand = Plus/Plus
Query        1 ATATCAACCTGTTTCCTCCTCCTCCTTCTCCTCCTCCTCCGTGACCTCCTCCTCCTCTTT 60      
               ||||||||||||||||||||||||||||||||||||||||||||||||||||||||||||         
Sbjct 74951382 ATATCAACCTGTTTCCTCCTCCTCCTTCTCCTCCTCCTCCGTGACCTCCTCCTCCTCTTT 74951441

Query       61 CTCCTGAGAAACTTCGCCCCAGCGGTGCGGAGCGCCGCTGCGCAGCCGGGGAGGGACGCA 120     
               ||||||||||||||||||||||||||||||||||||||||||||||||||||||||||||         
Sbjct 74951442 CTCCTGAGAAACTTCGCCCCAGCGGTGCGGAGCGCCGCTGCGCAGCCGGGGAGGGACGCA 74951501

Query      121 GGCAGGCGGCGGGCAGCGGGAGGCGGCAGC 150     
               ||||||||||||||||||||||||||||||         
Sbjct 74951502 GGCAGGCGGCGGGCAGCGGGAGGCGGCAGC 74951531
```

Report:

|  |  |
| --- | --- |
| sequence start ?  ``` Start position of the estimated full-length sequence in genome. Start index < end index. ``` : | 74951382 |
| sequence end ?  ``` End position of the estimated full-length sequence in genome. Start index < end index. ``` : | 74951531 |
| bit score (CM) ?  ``` The score for aligning estimated full-length sequence to CM model   (computed by RSEARCH -> default,   infered from Rfam or provided by user) ``` : | 196.1 |
| Homology estimate ?  ``` Quick homology estimate:   Not homologous: bit score < 0   Homologous: bit score > 20 and bit score > 0.5 * query length   Uncertain otherwise ``` : | Homologous |

Estimated full-length sequence:


?

```
Click checkbox to select multiple seuqences.
Fasta header format:
  UID|accession.versionSTRAND start-end
```

>uid:3|NT\_025741.16fw 74951382-74951531
AUAUCAACCUGUUUCCUCCUCCUCCUUCUCCUCCUCCUCCGUGACCUCCUCCUCCUCUUU
CUCCUGAGAAACUUCGCCCCAGCGGUGCGGAGCGCCGCUGCGCAGCCGGGGAGGGACGCA
GGCAGGCGGCGGGCAGCGGGAGGCGGCAGC

rnafold


?

```
Visualisation of predicted secondary structure.
To save the image:
  Right click on the image -> Save Image as.
```


Turbo-fast


?

```
Visualisation of predicted secondary structure.
To save the image:
  Right click on the image -> Save Image as.
```

Load Sequence viewer

Turbo-fast: Number of sequences is less then required. n=3 (4)

### Hit: NT\_025741.16

NT\_025741.16 Homo sapiens chromosome 6 genomic scaffold, GRCh38.p13 Primary Assembly HSCHR6\_CTG5

```
?

```
This is BLAST alignment as read from the input file
```

Score = 41.0 bits (38.3), Expect = 4.69E+00
 Identities = 39/49 (80%), Gaps = 3/49 (6%)
 Strand = Plus/Plus
Query       102 GCAGCCGGGGAGGGACGCAGGCAGGCGGCGGGCAGCGGGAGGC-GGCAG 149      
                |||| | || ||| | ||||||||||||| ||||  || |||| |||||          
Sbjct 100989957 GCAGGCAGGCAGGCAGGCAGGCAGGCGGCAGGCA--GGCAGGCAGGCAG 100990003
```

Report:

|  |  |
| --- | --- |
| sequence start ?  ``` Start position of the estimated full-length sequence in genome. Start index < end index. ``` : | 100989856 |
| sequence end ?  ``` End position of the estimated full-length sequence in genome. Start index < end index. ``` : | 100990004 |
| bit score (CM) ?  ``` The score for aligning estimated full-length sequence to CM model   (computed by RSEARCH -> default,   infered from Rfam or provided by user) ``` : | -23.2 |
| Homology estimate ?  ``` Quick homology estimate:   Not homologous: bit score < 0   Homologous: bit score > 20 and bit score > 0.5 * query length   Uncertain otherwise ``` : | Not homologous |

Estimated full-length sequence:


?

```
Click checkbox to select multiple seuqences.
Fasta header format:
  UID|accession.versionSTRAND start-end
```

>uid:4|NT\_025741.16fw 100989856-100990004
CAGGGACACAGAUUCACAGUAAAAAAGAUAGACAGAGAUAGAUAGAUAGAUAGAUAGAUA
GAUAGAUAGAUAGAUAGAUAGAUAGGCAGGCAGGCAGGCAGGCAGGCAGGCAGGCAGGCA
GGCAGGCGGCAGGCAGGCAGGCAGGCAGG

rnafold


?

```
Visualisation of predicted secondary structure.
To save the image:
  Right click on the image -> Save Image as.
```


Turbo-fast


?

```
Visualisation of predicted secondary structure.
To save the image:
  Right click on the image -> Save Image as.
```

Load Sequence viewer

### Hit: NC\_000020.11

NC\_000020.11 Homo sapiens chromosome 20, GRCh38.p13 Primary Assembly

```
?

```
This is BLAST alignment as read from the input file
```

Score = 49.0 bits (45.5), Expect = 3.16E-02
 Identities = 51/65 (78%), Gaps = 4/65 (6%)
 Strand = Plus/Plus
Query       87 GCGGAGCGCCGCTGCGCAGC--CGGGGAGGGACGCAGGCAGGCGGCGGGCAGCGGGAGGC 144     
               |||| ||| ||| || || |  ||| ||||| || | || |||||||||| |||||||||         
Sbjct 62861672 GCGGCGCGGCGCGGCCCAACGGCGGCGAGGG-CG-ACGCGGGCGGCGGGCGGCGGGAGGC 62861729

Query      145 GGCAG 149     
               || ||         
Sbjct 62861730 GGGAG 62861734
```

Report:

|  |  |
| --- | --- |
| sequence start ?  ``` Start position of the estimated full-length sequence in genome. Start index < end index. ``` : | 62861571 |
| sequence end ?  ``` End position of the estimated full-length sequence in genome. Start index < end index. ``` : | 62861735 |
| bit score (CM) ?  ``` The score for aligning estimated full-length sequence to CM model   (computed by RSEARCH -> default,   infered from Rfam or provided by user) ``` : | 8.0 |
| Homology estimate ?  ``` Quick homology estimate:   Not homologous: bit score < 0   Homologous: bit score > 20 and bit score > 0.5 * query length   Uncertain otherwise ``` : | Uncertain **↴** |

Check the secondary structure and sequence viewer
for supporting information about possible homology.

Estimated full-length sequence:


?

```
Click checkbox to select multiple seuqences.
Fasta header format:
  UID|accession.versionSTRAND start-end
```

>uid:5|NC\_000020.11fw 62861571-62861735
CGCCCAGCGCCGCGUCCCCGCCGCCCGCGCCCUCGACGGCCGCCUCGCCGCCUGCCGCGC
CUGCCUCCGGCGGCGGCUCCCGCGGUCCGGGGCCCGACAUGGCGGCGCGGCGCGGCCCAA
CGGCGGCGAGGGCGACGCGGGCGGCGGGCGGCGGGAGGCGGGAGG

rnafold


?

```
Visualisation of predicted secondary structure.
To save the image:
  Right click on the image -> Save Image as.
```


Turbo-fast


?

```
Visualisation of predicted secondary structure.
To save the image:
  Right click on the image -> Save Image as.
```

Load Sequence viewer

### Hit: NC\_000020.11

NC\_000020.11 Homo sapiens chromosome 20, GRCh38.p13 Primary Assembly

```
?

```
This is BLAST alignment as read from the input file
```

Score = 41.0 bits (38.3), Expect = 4.69E+00
 Identities = 28/33 (85%), Gaps = 0/33 (0%)
 Strand = Plus/Minus
Query    113 GGGACGCAGGCAGGCGGCGGGCAGCGGGAGGCG 145   
             ||| ||||||| ||||||||||| ||| | |||       
Sbjct 664159 GGGGCGCAGGCGGGCGGCGGGCACCGGCACGCG 664127
```

Report:

|  |  |
| --- | --- |
| sequence start ?  ``` Start position of the estimated full-length sequence in genome. Start index < end index. ``` : | 664120 |
| sequence end ?  ``` End position of the estimated full-length sequence in genome. Start index < end index. ``` : | 664271 |
| bit score (CM) ?  ``` The score for aligning estimated full-length sequence to CM model   (computed by RSEARCH -> default,   infered from Rfam or provided by user) ``` : | -17.83 |
| Homology estimate ?  ``` Quick homology estimate:   Not homologous: bit score < 0   Homologous: bit score > 20 and bit score > 0.5 * query length   Uncertain otherwise ``` : | Not homologous |

Estimated full-length sequence:


?

```
Click checkbox to select multiple seuqences.
Fasta header format:
  UID|accession.versionSTRAND start-end
```

>uid:6|NC\_000020.11rc 664120-664271
AUGGACGCCUUCUUCAUCUCGGACGGGCGCUCGCGGCGGCGGCGGGGCGGGGGCGGCGGG
GACGCGGGGGGCUCGGGAGACGCGGGGGGCGCCGGGGGGCGCGCGGGGCGCGCGGGGGCG
CAGGCGGGCGGCGGGCACCGGCACGCGUGCGC

rnafold


?

```
Visualisation of predicted secondary structure.
To save the image:
  Right click on the image -> Save Image as.
```


Turbo-fast


?

```
Visualisation of predicted secondary structure.
To save the image:
  Right click on the image -> Save Image as.
```

Load Sequence viewer

### Hit: NT\_011362.11

NT\_011362.11 Homo sapiens chromosome 20 genomic scaffold, GRCh38.p13 Primary Assembly HSCHR20\_CTG2

```
?

```
This is BLAST alignment as read from the input file
```

Score = 49.0 bits (45.5), Expect = 3.16E-02
 Identities = 51/65 (78%), Gaps = 4/65 (6%)
 Strand = Plus/Plus
Query       87 GCGGAGCGCCGCTGCGCAGC--CGGGGAGGGACGCAGGCAGGCGGCGGGCAGCGGGAGGC 144     
               |||| ||| ||| || || |  ||| ||||| || | || |||||||||| |||||||||         
Sbjct 31810164 GCGGCGCGGCGCGGCCCAACGGCGGCGAGGG-CG-ACGCGGGCGGCGGGCGGCGGGAGGC 31810221

Query      145 GGCAG 149     
               || ||         
Sbjct 31810222 GGGAG 31810226
```

Report:

|  |  |
| --- | --- |
| sequence start ?  ``` Start position of the estimated full-length sequence in genome. Start index < end index. ``` : | 31810063 |
| sequence end ?  ``` End position of the estimated full-length sequence in genome. Start index < end index. ``` : | 31810227 |
| bit score (CM) ?  ``` The score for aligning estimated full-length sequence to CM model   (computed by RSEARCH -> default,   infered from Rfam or provided by user) ``` : | 8.0 |
| Homology estimate ?  ``` Quick homology estimate:   Not homologous: bit score < 0   Homologous: bit score > 20 and bit score > 0.5 * query length   Uncertain otherwise ``` : | Uncertain **↴** |

Check the secondary structure and sequence viewer
for supporting information about possible homology.

Estimated full-length sequence:


?

```
Click checkbox to select multiple seuqences.
Fasta header format:
  UID|accession.versionSTRAND start-end
```

>uid:7|NT\_011362.11fw 31810063-31810227
CGCCCAGCGCCGCGUCCCCGCCGCCCGCGCCCUCGACGGCCGCCUCGCCGCCUGCCGCGC
CUGCCUCCGGCGGCGGCUCCCGCGGUCCGGGGCCCGACAUGGCGGCGCGGCGCGGCCCAA
CGGCGGCGAGGGCGACGCGGGCGGCGGGCGGCGGGAGGCGGGAGG

rnafold


?

```
Visualisation of predicted secondary structure.
To save the image:
  Right click on the image -> Save Image as.
```


Turbo-fast


?

```
Visualisation of predicted secondary structure.
To save the image:
  Right click on the image -> Save Image as.
```

Load Sequence viewer

### Hit: NT\_086366.1

NT\_086366.1 Homo sapiens chromosome 7 sequence, ENCODE region ENm010

```
?

```
This is BLAST alignment as read from the input file
```

Score = 45.0 bits (41.9), Expect = 3.85E-01
 Identities = 35/43 (81%), Gaps = 4/43 (9%)
 Strand = Plus/Plus
Query     99 TGCGCAGCCGGGGAGGGACGCAG----GCAGGCGGCGGGCAGC 137   
             |||||||| ||| ||||||||||    | |||||| |||||||       
Sbjct 190386 TGCGCAGCTGGGCAGGGACGCAGGGTAGGAGGCGGGGGGCAGC 190428
```

Report:

|  |  |
| --- | --- |
| sequence start ?  ``` Start position of the estimated full-length sequence in genome. Start index < end index. ``` : | 190281 |
| sequence end ?  ``` End position of the estimated full-length sequence in genome. Start index < end index. ``` : | 190441 |
| bit score (CM) ?  ``` The score for aligning estimated full-length sequence to CM model   (computed by RSEARCH -> default,   infered from Rfam or provided by user) ``` : | -18.88 |
| Homology estimate ?  ``` Quick homology estimate:   Not homologous: bit score < 0   Homologous: bit score > 20 and bit score > 0.5 * query length   Uncertain otherwise ``` : | Not homologous |

Estimated full-length sequence:


?

```
Click checkbox to select multiple seuqences.
Fasta header format:
  UID|accession.versionSTRAND start-end
```

>uid:8|NT\_086366.1fw 190281-190441
AUAGCUGCCGUUGCCCUGCAGGCCAUGAGCGUGCGGGUCAUAGUCGGGGGUGCCCCCUGC
GCCCGCCCCUGCCGCCGUGUAGCGCUUCUGUGGGGGUGGCGGGGGUGCGCAGCUGGGCAG
GGACGCAGGGUAGGAGGCGGGGGGCAGCCCGUAGGUACCCU

rnafold


?

```
Visualisation of predicted secondary structure.
To save the image:
  Right click on the image -> Save Image as.
```


Turbo-fast


?

```
Visualisation of predicted secondary structure.
To save the image:
  Right click on the image -> Save Image as.
```

Load Sequence viewer

### Hit: NC\_000007.14

NC\_000007.14 Homo sapiens chromosome 7, GRCh38.p13 Primary Assembly

```
?

```
This is BLAST alignment as read from the input file
```

Score = 45.0 bits (41.9), Expect = 3.85E-01
 Identities = 35/43 (81%), Gaps = 4/43 (9%)
 Strand = Plus/Plus
Query       99 TGCGCAGCCGGGGAGGGACGCAG----GCAGGCGGCGGGCAGC 137     
               |||||||| ||| ||||||||||    | |||||| |||||||         
Sbjct 27108287 TGCGCAGCTGGGCAGGGACGCAGGGTAGGAGGCGGGGGGCAGC 27108329
```

Report:

|  |  |
| --- | --- |
| sequence start ?  ``` Start position of the estimated full-length sequence in genome. Start index < end index. ``` : | 27108182 |
| sequence end ?  ``` End position of the estimated full-length sequence in genome. Start index < end index. ``` : | 27108342 |
| bit score (CM) ?  ``` The score for aligning estimated full-length sequence to CM model   (computed by RSEARCH -> default,   infered from Rfam or provided by user) ``` : | -18.88 |
| Homology estimate ?  ``` Quick homology estimate:   Not homologous: bit score < 0   Homologous: bit score > 20 and bit score > 0.5 * query length   Uncertain otherwise ``` : | Not homologous |

Estimated full-length sequence:


?

```
Click checkbox to select multiple seuqences.
Fasta header format:
  UID|accession.versionSTRAND start-end
```

>uid:9|NC\_000007.14fw 27108182-27108342
AUAGCUGCCGUUGCCCUGCAGGCCAUGAGCGUGCGGGUCAUAGUCGGGGGUGCCCCCUGC
GCCCGCCCCUGCCGCCGUGUAGCGCUUCUGUGGGGGUGGCGGGGGUGCGCAGCUGGGCAG
GGACGCAGGGUAGGAGGCGGGGGGCAGCCCGUAGGUACCCU

rnafold


?

```
Visualisation of predicted secondary structure.
To save the image:
  Right click on the image -> Save Image as.
```


Turbo-fast


?

```
Visualisation of predicted secondary structure.
To save the image:
  Right click on the image -> Save Image as.
```

Load Sequence viewer

### Hit: NC\_000007.14

NC\_000007.14 Homo sapiens chromosome 7, GRCh38.p13 Primary Assembly

```
?

```
This is BLAST alignment as read from the input file
```

Score = 41.0 bits (38.3), Expect = 4.69E+00
 Identities = 28/33 (85%), Gaps = 0/33 (0%)
 Strand = Plus/Minus
Query      101 CGCAGCCGGGGAGGGACGCAGGCAGGCGGCGGG 133     
               || |||||| |||||||||||| || ||| |||         
Sbjct 30595411 CGGAGCCGGAGAGGGACGCAGGAAGTCGGAGGG 30595379
```

Report:

|  |  |
| --- | --- |
| sequence start ?  ``` Start position of the estimated full-length sequence in genome. Start index < end index. ``` : | 30595361 |
| sequence end ?  ``` End position of the estimated full-length sequence in genome. Start index < end index. ``` : | 30595510 |
| bit score (CM) ?  ``` The score for aligning estimated full-length sequence to CM model   (computed by RSEARCH -> default,   infered from Rfam or provided by user) ``` : | -19.71 |
| Homology estimate ?  ``` Quick homology estimate:   Not homologous: bit score < 0   Homologous: bit score > 20 and bit score > 0.5 * query length   Uncertain otherwise ``` : | Not homologous |

Estimated full-length sequence:


?

```
Click checkbox to select multiple seuqences.
Fasta header format:
  UID|accession.versionSTRAND start-end
```

>uid:10|NC\_000007.14rc 30595361-30595510
AGGUUAUUCAGUUAGGAGGCGAGGACAACCAAAGGGUCACAGAUCCCAGCCCCACUGGAA
AUAGAGGAGACGCGGGCUGUGACAGAAUGGGGAAGGGAGAACGGAGCCGGAGAGGGACGC
AGGAAGUCGGAGGGGUUCCAGGGAGUCAUC

rnafold


?

```
Visualisation of predicted secondary structure.
To save the image:
  Right click on the image -> Save Image as.
```


Turbo-fast


?

```
Visualisation of predicted secondary structure.
To save the image:
  Right click on the image -> Save Image as.
```

Load Sequence viewer

### Hit: NT\_007819.18

NT\_007819.18 Homo sapiens chromosome 7 genomic scaffold, GRCh38.p13 Primary Assembly HSCHR7\_CTG1

```
?

```
This is BLAST alignment as read from the input file
```

Score = 45.0 bits (41.9), Expect = 3.85E-01
 Identities = 35/43 (81%), Gaps = 4/43 (9%)
 Strand = Plus/Plus
Query       99 TGCGCAGCCGGGGAGGGACGCAG----GCAGGCGGCGGGCAGC 137     
               |||||||| ||| ||||||||||    | |||||| |||||||         
Sbjct 27098287 TGCGCAGCTGGGCAGGGACGCAGGGTAGGAGGCGGGGGGCAGC 27098329
```

Report:

|  |  |
| --- | --- |
| sequence start ?  ``` Start position of the estimated full-length sequence in genome. Start index < end index. ``` : | 27098182 |
| sequence end ?  ``` End position of the estimated full-length sequence in genome. Start index < end index. ``` : | 27098342 |
| bit score (CM) ?  ``` The score for aligning estimated full-length sequence to CM model   (computed by RSEARCH -> default,   infered from Rfam or provided by user) ``` : | -18.88 |
| Homology estimate ?  ``` Quick homology estimate:   Not homologous: bit score < 0   Homologous: bit score > 20 and bit score > 0.5 * query length   Uncertain otherwise ``` : | Not homologous |

Estimated full-length sequence:


?

```
Click checkbox to select multiple seuqences.
Fasta header format:
  UID|accession.versionSTRAND start-end
```

>uid:11|NT\_007819.18fw 27098182-27098342
AUAGCUGCCGUUGCCCUGCAGGCCAUGAGCGUGCGGGUCAUAGUCGGGGGUGCCCCCUGC
GCCCGCCCCUGCCGCCGUGUAGCGCUUCUGUGGGGGUGGCGGGGGUGCGCAGCUGGGCAG
GGACGCAGGGUAGGAGGCGGGGGGCAGCCCGUAGGUACCCU

rnafold


?

```
Visualisation of predicted secondary structure.
To save the image:
  Right click on the image -> Save Image as.
```


Turbo-fast


?

```
Visualisation of predicted secondary structure.
To save the image:
  Right click on the image -> Save Image as.
```

Load Sequence viewer

### Hit: NT\_007819.18

NT\_007819.18 Homo sapiens chromosome 7 genomic scaffold, GRCh38.p13 Primary Assembly HSCHR7\_CTG1

```
?

```
This is BLAST alignment as read from the input file
```

Score = 41.0 bits (38.3), Expect = 4.69E+00
 Identities = 28/33 (85%), Gaps = 0/33 (0%)
 Strand = Plus/Minus
Query      101 CGCAGCCGGGGAGGGACGCAGGCAGGCGGCGGG 133     
               || |||||| |||||||||||| || ||| |||         
Sbjct 30585411 CGGAGCCGGAGAGGGACGCAGGAAGTCGGAGGG 30585379
```

Report:

|  |  |
| --- | --- |
| sequence start ?  ``` Start position of the estimated full-length sequence in genome. Start index < end index. ``` : | 30585361 |
| sequence end ?  ``` End position of the estimated full-length sequence in genome. Start index < end index. ``` : | 30585510 |
| bit score (CM) ?  ``` The score for aligning estimated full-length sequence to CM model   (computed by RSEARCH -> default,   infered from Rfam or provided by user) ``` : | -19.71 |
| Homology estimate ?  ``` Quick homology estimate:   Not homologous: bit score < 0   Homologous: bit score > 20 and bit score > 0.5 * query length   Uncertain otherwise ``` : | Not homologous |

Estimated full-length sequence:


?

```
Click checkbox to select multiple seuqences.
Fasta header format:
  UID|accession.versionSTRAND start-end
```

>uid:12|NT\_007819.18rc 30585361-30585510
AGGUUAUUCAGUUAGGAGGCGAGGACAACCAAAGGGUCACAGAUCCCAGCCCCACUGGAA
AUAGAGGAGACGCGGGCUGUGACAGAAUGGGGAAGGGAGAACGGAGCCGGAGAGGGACGC
AGGAAGUCGGAGGGGUUCCAGGGAGUCAUC

rnafold


?

```
Visualisation of predicted secondary structure.
To save the image:
  Right click on the image -> Save Image as.
```


Turbo-fast


?

```
Visualisation of predicted secondary structure.
To save the image:
  Right click on the image -> Save Image as.
```

Load Sequence viewer

### Hit: NG\_030344.1

NG\_030344.1 Homo sapiens EBF transcription factor 2 (EBF2), RefSeqGene on chromosome 8

```
?

```
This is BLAST alignment as read from the input file
```

Score = 44.0 bits (41.0), Expect = 3.85E-01
 Identities = 41/50 (82%), Gaps = 4/50 (8%)
 Strand = Plus/Minus
Query   102 GCAGCCGGGGAGGGACGCAGGCAGGC-GGCGGGCAGCGGGAGGC-GGCAG 149  
            |||| |||| ||| | |||||||||| ||||||||  || |||| |||||      
Sbjct 47207 GCAGGCGGGCAGGCAGGCAGGCAGGCGGGCGGGCA--GGCAGGCAGGCAG 47160
```

Report:

|  |  |
| --- | --- |
| sequence start ?  ``` Start position of the estimated full-length sequence in genome. Start index < end index. ``` : | 47157 |
| sequence end ?  ``` End position of the estimated full-length sequence in genome. Start index < end index. ``` : | 47309 |
| bit score (CM) ?  ``` The score for aligning estimated full-length sequence to CM model   (computed by RSEARCH -> default,   infered from Rfam or provided by user) ``` : | -17.91 |
| Homology estimate ?  ``` Quick homology estimate:   Not homologous: bit score < 0   Homologous: bit score > 20 and bit score > 0.5 * query length   Uncertain otherwise ``` : | Not homologous |

Estimated full-length sequence:


?

```
Click checkbox to select multiple seuqences.
Fasta header format:
  UID|accession.versionSTRAND start-end
```

>uid:13|NG\_030344.1rc 47157-47309
AAGCCAACGGGACAGGCAGGCAGGCAGGCAGGCAGGCAGGCGGGCAGGCGGGCAGGCGGG
CAGGCGGGCAGGCGGGCAGGCAGGCGGGCAGGCGGGCAGGCGGGCAGGCGGGCAGGCAGG
CAGGCAGGCGGGCGGGCAGGCAGGCAGGCAGGC

rnafold


?

```
Visualisation of predicted secondary structure.
To save the image:
  Right click on the image -> Save Image as.
```


Turbo-fast


?

```
Visualisation of predicted secondary structure.
To save the image:
  Right click on the image -> Save Image as.
```

Load Sequence viewer

### Hit: NC\_000008.11

NC\_000008.11 Homo sapiens chromosome 8, GRCh38.p13 Primary Assembly

```
?

```
This is BLAST alignment as read from the input file
```

Score = 44.0 bits (41.0), Expect = 3.85E-01
 Identities = 41/50 (82%), Gaps = 4/50 (8%)
 Strand = Plus/Plus
Query      102 GCAGCCGGGGAGGGACGCAGGCAGGC-GGCGGGCAGCGGGAGGC-GGCAG 149     
               |||| |||| ||| | |||||||||| ||||||||  || |||| |||||         
Sbjct 26002918 GCAGGCGGGCAGGCAGGCAGGCAGGCGGGCGGGCA--GGCAGGCAGGCAG 26002965
```

Report:

|  |  |
| --- | --- |
| sequence start ?  ``` Start position of the estimated full-length sequence in genome. Start index < end index. ``` : | 26002815 |
| sequence end ?  ``` End position of the estimated full-length sequence in genome. Start index < end index. ``` : | 26002967 |
| bit score (CM) ?  ``` The score for aligning estimated full-length sequence to CM model   (computed by RSEARCH -> default,   infered from Rfam or provided by user) ``` : | -17.91 |
| Homology estimate ?  ``` Quick homology estimate:   Not homologous: bit score < 0   Homologous: bit score > 20 and bit score > 0.5 * query length   Uncertain otherwise ``` : | Not homologous |

Estimated full-length sequence:


?

```
Click checkbox to select multiple seuqences.
Fasta header format:
  UID|accession.versionSTRAND start-end
```

>uid:14|NC\_000008.11fw 26002815-26002967
AAGCCAACGGGACAGGCAGGCAGGCAGGCAGGCAGGCAGGCGGGCAGGCGGGCAGGCGGG
CAGGCGGGCAGGCGGGCAGGCAGGCGGGCAGGCGGGCAGGCGGGCAGGCGGGCAGGCAGG
CAGGCAGGCGGGCGGGCAGGCAGGCAGGCAGGC

rnafold


?

```
Visualisation of predicted secondary structure.
To save the image:
  Right click on the image -> Save Image as.
```


Turbo-fast


?

```
Visualisation of predicted secondary structure.
To save the image:
  Right click on the image -> Save Image as.
```

Load Sequence viewer

### Hit: NC\_000022.11

NC\_000022.11 Homo sapiens chromosome 22, GRCh38.p13 Primary Assembly

```
?

```
This is BLAST alignment as read from the input file
```

Score = 44.0 bits (41.0), Expect = 3.85E-01
 Identities = 25/27 (93%), Gaps = 0/27 (0%)
 Strand = Plus/Plus
Query      121 GGCAGGCGGCGGGCAGCGGGAGGCGGC 147     
               ||| |||||||||| ||||||||||||         
Sbjct 46738236 GGCGGGCGGCGGGCGGCGGGAGGCGGC 46738262
```

Report:

|  |  |
| --- | --- |
| sequence start ?  ``` Start position of the estimated full-length sequence in genome. Start index < end index. ``` : | 46738118 |
| sequence end ?  ``` End position of the estimated full-length sequence in genome. Start index < end index. ``` : | 46738265 |
| bit score (CM) ?  ``` The score for aligning estimated full-length sequence to CM model   (computed by RSEARCH -> default,   infered from Rfam or provided by user) ``` : | 19.45 |
| Homology estimate ?  ``` Quick homology estimate:   Not homologous: bit score < 0   Homologous: bit score > 20 and bit score > 0.5 * query length   Uncertain otherwise ``` : | Uncertain **↴** |

Check the secondary structure and sequence viewer
for supporting information about possible homology.

Estimated full-length sequence:


?

```
Click checkbox to select multiple seuqences.
Fasta header format:
  UID|accession.versionSTRAND start-end
```

>uid:15|NC\_000022.11fw 46738118-46738265
GCAGCGGCUCCGCCGCCCCCGUCGCCCCCAUCUCCGCCGCCGGGCUCGUCCGCCAGGCUG
GGGGCGCGCGGACGCCGAGGGGCGCCGGACCGUUAGCGGCCCCUGCAGUGGCCCGGGCGG
CGGGCGGCGGGCGGCGGGAGGCGGCGCU

rnafold


?

```
Visualisation of predicted secondary structure.
To save the image:
  Right click on the image -> Save Image as.
```


Turbo-fast


?

```
Visualisation of predicted secondary structure.
To save the image:
  Right click on the image -> Save Image as.
```

Load Sequence viewer

Turbo-fast: Number of sequences is less then required. n=3 (4)

### Hit: NT\_167187.2

NT\_167187.2 Homo sapiens chromosome 8 genomic scaffold, GRCh38.p13 Primary Assembly HSCHR8\_CTG3

```
?

```
This is BLAST alignment as read from the input file
```

Score = 44.0 bits (41.0), Expect = 3.85E-01
 Identities = 41/50 (82%), Gaps = 4/50 (8%)
 Strand = Plus/Plus
Query      102 GCAGCCGGGGAGGGACGCAGGCAGGC-GGCGGGCAGCGGGAGGC-GGCAG 149     
               |||| |||| ||| | |||||||||| ||||||||  || |||| |||||         
Sbjct 13718573 GCAGGCGGGCAGGCAGGCAGGCAGGCGGGCGGGCA--GGCAGGCAGGCAG 13718620
```

Report:

|  |  |
| --- | --- |
| sequence start ?  ``` Start position of the estimated full-length sequence in genome. Start index < end index. ``` : | 13718470 |
| sequence end ?  ``` End position of the estimated full-length sequence in genome. Start index < end index. ``` : | 13718622 |
| bit score (CM) ?  ``` The score for aligning estimated full-length sequence to CM model   (computed by RSEARCH -> default,   infered from Rfam or provided by user) ``` : | -17.91 |
| Homology estimate ?  ``` Quick homology estimate:   Not homologous: bit score < 0   Homologous: bit score > 20 and bit score > 0.5 * query length   Uncertain otherwise ``` : | Not homologous |

Estimated full-length sequence:


?

```
Click checkbox to select multiple seuqences.
Fasta header format:
  UID|accession.versionSTRAND start-end
```

>uid:16|NT\_167187.2fw 13718470-13718622
AAGCCAACGGGACAGGCAGGCAGGCAGGCAGGCAGGCAGGCGGGCAGGCGGGCAGGCGGG
CAGGCGGGCAGGCGGGCAGGCAGGCGGGCAGGCGGGCAGGCGGGCAGGCGGGCAGGCAGG
CAGGCAGGCGGGCGGGCAGGCAGGCAGGCAGGC

rnafold


?

```
Visualisation of predicted secondary structure.
To save the image:
  Right click on the image -> Save Image as.
```


Turbo-fast


?

```
Visualisation of predicted secondary structure.
To save the image:
  Right click on the image -> Save Image as.
```

Load Sequence viewer

### Hit: NT\_011520.13

NT\_011520.13 Homo sapiens chromosome 22 genomic scaffold, GRCh38.p13 Primary Assembly HSCHR22\_CTG3\_2

```
?

```
This is BLAST alignment as read from the input file
```

Score = 44.0 bits (41.0), Expect = 3.85E-01
 Identities = 25/27 (93%), Gaps = 0/27 (0%)
 Strand = Plus/Plus
Query      121 GGCAGGCGGCGGGCAGCGGGAGGCGGC 147     
               ||| |||||||||| ||||||||||||         
Sbjct 28028672 GGCGGGCGGCGGGCGGCGGGAGGCGGC 28028698
```

Report:

|  |  |
| --- | --- |
| sequence start ?  ``` Start position of the estimated full-length sequence in genome. Start index < end index. ``` : | 28028554 |
| sequence end ?  ``` End position of the estimated full-length sequence in genome. Start index < end index. ``` : | 28028701 |
| bit score (CM) ?  ``` The score for aligning estimated full-length sequence to CM model   (computed by RSEARCH -> default,   infered from Rfam or provided by user) ``` : | 19.45 |
| Homology estimate ?  ``` Quick homology estimate:   Not homologous: bit score < 0   Homologous: bit score > 20 and bit score > 0.5 * query length   Uncertain otherwise ``` : | Uncertain **↴** |

Check the secondary structure and sequence viewer
for supporting information about possible homology.

Estimated full-length sequence:


?

```
Click checkbox to select multiple seuqences.
Fasta header format:
  UID|accession.versionSTRAND start-end
```

>uid:17|NT\_011520.13fw 28028554-28028701
GCAGCGGCUCCGCCGCCCCCGUCGCCCCCAUCUCCGCCGCCGGGCUCGUCCGCCAGGCUG
GGGGCGCGCGGACGCCGAGGGGCGCCGGACCGUUAGCGGCCCCUGCAGUGGCCCGGGCGG
CGGGCGGCGGGCGGCGGGAGGCGGCGCU

rnafold


?

```
Visualisation of predicted secondary structure.
To save the image:
  Right click on the image -> Save Image as.
```


Turbo-fast


?

```
Visualisation of predicted secondary structure.
To save the image:
  Right click on the image -> Save Image as.
```

Load Sequence viewer

Turbo-fast: Number of sequences is less then required. n=3 (4)

### Hit: NG\_029771.1

NG\_029771.1 Homo sapiens microtubule affinity regulating kinase 2 (MARK2), RefSeqGene on chromosome 11

```
?

```
This is BLAST alignment as read from the input file
```

Score = 43.0 bits (40.1), Expect = 1.34E+00
 Identities = 46/59 (78%), Gaps = 5/59 (8%)
 Strand = Plus/Plus
Query   84 GGTGCGGAGC---GCCGCTGCGCAGCCGGGGAGGGACGCAGGCAGGCGGCGGGCAGCGG 139 
           || |||||||   ||||||| |  || |||||||  || ||||||||||| ||| ||||     
Sbjct 5241 GGCGCGGAGCAGTGCCGCTGAGG-GCAGGGGAGGAGCG-AGGCAGGCGGCCGGCTGCGG 5297
```

Report:

|  |  |
| --- | --- |
| sequence start ?  ``` Start position of the estimated full-length sequence in genome. Start index < end index. ``` : | 5161 |
| sequence end ?  ``` End position of the estimated full-length sequence in genome. Start index < end index. ``` : | 5308 |
| bit score (CM) ?  ``` The score for aligning estimated full-length sequence to CM model   (computed by RSEARCH -> default,   infered from Rfam or provided by user) ``` : | -22.38 |
| Homology estimate ?  ``` Quick homology estimate:   Not homologous: bit score < 0   Homologous: bit score > 20 and bit score > 0.5 * query length   Uncertain otherwise ``` : | Not homologous |

Estimated full-length sequence:


?

```
Click checkbox to select multiple seuqences.
Fasta header format:
  UID|accession.versionSTRAND start-end
```

>uid:18|NG\_029771.1fw 5161-5308
CCGGCGAAAAGAGCCGAGGGCCGGCGGUGGUGGCGGCCAUGUUGGGAGCAGCAGGUCCGG
CGGCGGCUGCCUGUGUGCCGGGCGCGGAGCAGUGCCGCUGAGGGCAGGGGAGGAGCGAGG
CAGGCGGCCGGCUGCGGCGGCAGAGAGU

rnafold


?

```
Visualisation of predicted secondary structure.
To save the image:
  Right click on the image -> Save Image as.
```


Turbo-fast


?

```
Visualisation of predicted secondary structure.
To save the image:
  Right click on the image -> Save Image as.
```

Load Sequence viewer

### Hit: NC\_000011.10

NC\_000011.10 Homo sapiens chromosome 11, GRCh38.p13 Primary Assembly

```
?

```
This is BLAST alignment as read from the input file
```

Score = 43.0 bits (40.1), Expect = 1.34E+00
 Identities = 46/59 (78%), Gaps = 5/59 (8%)
 Strand = Plus/Plus
Query       84 GGTGCGGAGC---GCCGCTGCGCAGCCGGGGAGGGACGCAGGCAGGCGGCGGGCAGCGG 139     
               || |||||||   ||||||| |  || |||||||  || ||||||||||| ||| ||||         
Sbjct 63839168 GGCGCGGAGCAGTGCCGCTGAGG-GCAGGGGAGGAGCG-AGGCAGGCGGCCGGCTGCGG 63839224
```

Report:

|  |  |
| --- | --- |
| sequence start ?  ``` Start position of the estimated full-length sequence in genome. Start index < end index. ``` : | 63839088 |
| sequence end ?  ``` End position of the estimated full-length sequence in genome. Start index < end index. ``` : | 63839235 |
| bit score (CM) ?  ``` The score for aligning estimated full-length sequence to CM model   (computed by RSEARCH -> default,   infered from Rfam or provided by user) ``` : | -22.38 |
| Homology estimate ?  ``` Quick homology estimate:   Not homologous: bit score < 0   Homologous: bit score > 20 and bit score > 0.5 * query length   Uncertain otherwise ``` : | Not homologous |

Estimated full-length sequence:


?

```
Click checkbox to select multiple seuqences.
Fasta header format:
  UID|accession.versionSTRAND start-end
```

>uid:19|NC\_000011.10fw 63839088-63839235
CCGGCGAAAAGAGCCGAGGGCCGGCGGUGGUGGCGGCCAUGUUGGGAGCAGCAGGUCCGG
CGGCGGCUGCCUGUGUGCCGGGCGCGGAGCAGUGCCGCUGAGGGCAGGGGAGGAGCGAGG
CAGGCGGCCGGCUGCGGCGGCAGAGAGU

rnafold


?

```
Visualisation of predicted secondary structure.
To save the image:
  Right click on the image -> Save Image as.
```


Turbo-fast


?

```
Visualisation of predicted secondary structure.
To save the image:
  Right click on the image -> Save Image as.
```

Load Sequence viewer

### Hit: NC\_000011.10

NC\_000011.10 Homo sapiens chromosome 11, GRCh38.p13 Primary Assembly

```
?

```
This is BLAST alignment as read from the input file
```

Score = 43.0 bits (40.1), Expect = 1.34E+00
 Identities = 28/31 (90%), Gaps = 1/31 (3%)
 Strand = Plus/Plus
Query       105 GCCGGGGAGGGACG-CAGGCAGGCGGCGGGC 134      
                |||||||||||||| || |||||| ||||||          
Sbjct 108498064 GCCGGGGAGGGACGCCACGCAGGCCGCGGGC 108498094
```

Report:

|  |  |
| --- | --- |
| sequence start ?  ``` Start position of the estimated full-length sequence in genome. Start index < end index. ``` : | 108497948 |
| sequence end ?  ``` End position of the estimated full-length sequence in genome. Start index < end index. ``` : | 108498115 |
| bit score (CM) ?  ``` The score for aligning estimated full-length sequence to CM model   (computed by RSEARCH -> default,   infered from Rfam or provided by user) ``` : | -17.55 |
| Homology estimate ?  ``` Quick homology estimate:   Not homologous: bit score < 0   Homologous: bit score > 20 and bit score > 0.5 * query length   Uncertain otherwise ``` : | Not homologous |

Estimated full-length sequence:


?

```
Click checkbox to select multiple seuqences.
Fasta header format:
  UID|accession.versionSTRAND start-end
```

>uid:20|NC\_000011.10fw 108497948-108498115
CCUUCAAGUUCAUUCAUUCAUUCAUUCCACAAACUUUCGUGGUGAUUUUUGCCCGCUUUG
AGUAUGGGCGGGAGGAGGGAAGAGGAGCUGACCAGACCCAAAAAGGGGCGACACACGCCG
GGGAGGGACGCCACGCAGGCCGCGGGCGGACUCCCGGGCGGCGGGGAC

rnafold


?

```
Visualisation of predicted secondary structure.
To save the image:
  Right click on the image -> Save Image as.
```


Turbo-fast


?

```
Visualisation of predicted secondary structure.
To save the image:
  Right click on the image -> Save Image as.
```

Load Sequence viewer

### Hit: NT\_167190.2

NT\_167190.2 Homo sapiens chromosome 11 genomic scaffold, GRCh38.p13 Primary Assembly HSCHR11\_CTG3\_1

```
?

```
This is BLAST alignment as read from the input file
```

Score = 43.0 bits (40.1), Expect = 1.34E+00
 Identities = 46/59 (78%), Gaps = 5/59 (8%)
 Strand = Plus/Plus
Query      84 GGTGCGGAGC---GCCGCTGCGCAGCCGGGGAGGGACGCAGGCAGGCGGCGGGCAGCGG 139    
              || |||||||   ||||||| |  || |||||||  || ||||||||||| ||| ||||        
Sbjct 9314094 GGCGCGGAGCAGTGCCGCTGAGG-GCAGGGGAGGAGCG-AGGCAGGCGGCCGGCTGCGG 9314150
```

Report:

|  |  |
| --- | --- |
| sequence start ?  ``` Start position of the estimated full-length sequence in genome. Start index < end index. ``` : | 9314014 |
| sequence end ?  ``` End position of the estimated full-length sequence in genome. Start index < end index. ``` : | 9314161 |
| bit score (CM) ?  ``` The score for aligning estimated full-length sequence to CM model   (computed by RSEARCH -> default,   infered from Rfam or provided by user) ``` : | -22.38 |
| Homology estimate ?  ``` Quick homology estimate:   Not homologous: bit score < 0   Homologous: bit score > 20 and bit score > 0.5 * query length   Uncertain otherwise ``` : | Not homologous |

Estimated full-length sequence:


?

```
Click checkbox to select multiple seuqences.
Fasta header format:
  UID|accession.versionSTRAND start-end
```

>uid:21|NT\_167190.2fw 9314014-9314161
CCGGCGAAAAGAGCCGAGGGCCGGCGGUGGUGGCGGCCAUGUUGGGAGCAGCAGGUCCGG
CGGCGGCUGCCUGUGUGCCGGGCGCGGAGCAGUGCCGCUGAGGGCAGGGGAGGAGCGAGG
CAGGCGGCCGGCUGCGGCGGCAGAGAGU

rnafold


?

```
Visualisation of predicted secondary structure.
To save the image:
  Right click on the image -> Save Image as.
```


Turbo-fast


?

```
Visualisation of predicted secondary structure.
To save the image:
  Right click on the image -> Save Image as.
```

Load Sequence viewer

### Hit: NT\_033899.9

NT\_033899.9 Homo sapiens chromosome 11 genomic scaffold, GRCh38.p13 Primary Assembly HSCHR11\_CTG8

```
?

```
This is BLAST alignment as read from the input file
```

Score = 43.0 bits (40.1), Expect = 1.34E+00
 Identities = 28/31 (90%), Gaps = 1/31 (3%)
 Strand = Plus/Plus
Query      105 GCCGGGGAGGGACG-CAGGCAGGCGGCGGGC 134     
               |||||||||||||| || |||||| ||||||         
Sbjct 20495168 GCCGGGGAGGGACGCCACGCAGGCCGCGGGC 20495198
```

Report:

|  |  |
| --- | --- |
| sequence start ?  ``` Start position of the estimated full-length sequence in genome. Start index < end index. ``` : | 20495052 |
| sequence end ?  ``` End position of the estimated full-length sequence in genome. Start index < end index. ``` : | 20495219 |
| bit score (CM) ?  ``` The score for aligning estimated full-length sequence to CM model   (computed by RSEARCH -> default,   infered from Rfam or provided by user) ``` : | -17.55 |
| Homology estimate ?  ``` Quick homology estimate:   Not homologous: bit score < 0   Homologous: bit score > 20 and bit score > 0.5 * query length   Uncertain otherwise ``` : | Not homologous |

Estimated full-length sequence:


?

```
Click checkbox to select multiple seuqences.
Fasta header format:
  UID|accession.versionSTRAND start-end
```

>uid:22|NT\_033899.9fw 20495052-20495219
CCUUCAAGUUCAUUCAUUCAUUCAUUCCACAAACUUUCGUGGUGAUUUUUGCCCGCUUUG
AGUAUGGGCGGGAGGAGGGAAGAGGAGCUGACCAGACCCAAAAAGGGGCGACACACGCCG
GGGAGGGACGCCACGCAGGCCGCGGGCGGACUCCCGGGCGGCGGGGAC

rnafold


?

```
Visualisation of predicted secondary structure.
To save the image:
  Right click on the image -> Save Image as.
```


Turbo-fast


?

```
Visualisation of predicted secondary structure.
To save the image:
  Right click on the image -> Save Image as.
```

Load Sequence viewer

### Hit: NG\_023249.1

NG\_023249.1 Homo sapiens hydroxyacylglutathione hydrolase (HAGH), RefSeqGene on chromosome 16

```
?

```
This is BLAST alignment as read from the input file
```

Score = 42.0 bits (39.2), Expect = 1.34E+00
 Identities = 50/65 (77%), Gaps = 7/65 (11%)
 Strand = Plus/Plus
Query   84 GGTGCGGAGCGCCGCTGCGCAGCCGGGGAGGGACGCAGGCA-GGCGG--CGGGCAGCGGGAGGCG 145 
           ||||||| | | || ||||  |||||||   ||||| |||  |||||  ||||||||||| ||||     
Sbjct 5487 GGTGCGGCGGGGCGGTGCGG-GCCGGGG---GACGCGGGCCTGGCGGGGCGGGCAGCGGGCGGCG 5547
```

Report:

|  |  |
| --- | --- |
| sequence start ?  ``` Start position of the estimated full-length sequence in genome. Start index < end index. ``` : | 5411 |
| sequence end ?  ``` End position of the estimated full-length sequence in genome. Start index < end index. ``` : | 5552 |
| bit score (CM) ?  ``` The score for aligning estimated full-length sequence to CM model   (computed by RSEARCH -> default,   infered from Rfam or provided by user) ``` : | -3.87 |
| Homology estimate ?  ``` Quick homology estimate:   Not homologous: bit score < 0   Homologous: bit score > 20 and bit score > 0.5 * query length   Uncertain otherwise ``` : | Not homologous |

Estimated full-length sequence:


?

```
Click checkbox to select multiple seuqences.
Fasta header format:
  UID|accession.versionSTRAND start-end
```

>uid:23|NG\_023249.1fw 5411-5552
GUGGUGGGCCGAGGGCUGCUCGGCCGCCGCAGCCUCGCCGCGCUGGGAGCCGCCUGCGCC
CGCCGAGGCCUCGGUGGGUGCGGCGGGGCGGUGCGGGCCGGGGGACGCGGGCCUGGCGGG
GCGGGCAGCGGGCGGCGCCGGG

rnafold


?

```
Visualisation of predicted secondary structure.
To save the image:
  Right click on the image -> Save Image as.
```


Turbo-fast


?

```
Visualisation of predicted secondary structure.
To save the image:
  Right click on the image -> Save Image as.
```

Load Sequence viewer

### Hit: NC\_000004.12

NC\_000004.12 Homo sapiens chromosome 4, GRCh38.p13 Primary Assembly

```
?

```
This is BLAST alignment as read from the input file
```

Score = 42.0 bits (39.2), Expect = 1.34E+00
 Identities = 40/49 (82%), Gaps = 4/49 (8%)
 Strand = Plus/Plus
Query     102 GCAGCCGGGGAGG-GACG--CAGGCAGGCGGCGGGCAGCG-GGAGGCGG 146    
              |||||| |||||| | ||  |||  ||| ||||||||||| ||||||||        
Sbjct 6761975 GCAGCCAGGGAGGAGGCGGGCAGCGAGGAGGCGGGCAGCGAGGAGGCGG 6762023
```

Report:

|  |  |
| --- | --- |
| sequence start ?  ``` Start position of the estimated full-length sequence in genome. Start index < end index. ``` : | 6761874 |
| sequence end ?  ``` End position of the estimated full-length sequence in genome. Start index < end index. ``` : | 6762028 |
| bit score (CM) ?  ``` The score for aligning estimated full-length sequence to CM model   (computed by RSEARCH -> default,   infered from Rfam or provided by user) ``` : | -13.21 |
| Homology estimate ?  ``` Quick homology estimate:   Not homologous: bit score < 0   Homologous: bit score > 20 and bit score > 0.5 * query length   Uncertain otherwise ``` : | Not homologous |

Estimated full-length sequence:


?

```
Click checkbox to select multiple seuqences.
Fasta header format:
  UID|accession.versionSTRAND start-end
```

>uid:24|NC\_000004.12fw 6761874-6762028
GGGCUGACGCUCACAUUUCCGCACUGGCCCCAUUUCUUGCUGCAGAAACGCACUGACUGC
CUUCACAGGCUUCCUGCAGCUGAGCUGGGGGCCUCCGCCUGGCAGCCAGGGAGGAGGCGG
GCAGCGAGGAGGCGGGCAGCGAGGAGGCGGGCAGG

rnafold


?

```
Visualisation of predicted secondary structure.
To save the image:
  Right click on the image -> Save Image as.
```


Turbo-fast


?

```
Visualisation of predicted secondary structure.
To save the image:
  Right click on the image -> Save Image as.
```

Load Sequence viewer

### Hit: NC\_000004.12

NC\_000004.12 Homo sapiens chromosome 4, GRCh38.p13 Primary Assembly

```
?

```
This is BLAST alignment as read from the input file
```

Score = 41.0 bits (38.3), Expect = 4.69E+00
 Identities = 53/71 (75%), Gaps = 4/71 (6%)
 Strand = Plus/Plus
Query      75 CGCCCCAGCGGTGCGGAGCGCCGCTGCGCAGCCGGGG-AGGGAC-GCAGGCAGGCGGCGGGC 134    
              |||||| || |  ||  |||||||   ||||||  || ||| || || |||||||||||||         
Sbjct 6782802 CGCCCCCGCCGGCCGCCGCGCCGCCCGGCAGCCTCGGCAGGTACTGCGGGCAGGCGGCGGG- 6782862

Query     135 AGCGGGAGG 143    
               | ||||||        
Sbjct 6782863 -GAGGGAGG 6782870
```

Report:

|  |  |
| --- | --- |
| sequence start ?  ``` Start position of the estimated full-length sequence in genome. Start index < end index. ``` : | 6782729 |
| sequence end ?  ``` End position of the estimated full-length sequence in genome. Start index < end index. ``` : | 6782879 |
| bit score (CM) ?  ``` The score for aligning estimated full-length sequence to CM model   (computed by RSEARCH -> default,   infered from Rfam or provided by user) ``` : | -21.46 |
| Homology estimate ?  ``` Quick homology estimate:   Not homologous: bit score < 0   Homologous: bit score > 20 and bit score > 0.5 * query length   Uncertain otherwise ``` : | Not homologous |

Estimated full-length sequence:


?

```
Click checkbox to select multiple seuqences.
Fasta header format:
  UID|accession.versionSTRAND start-end
```

>uid:25|NC\_000004.12fw 6782729-6782879
UUUACGUAAGGCGCAGGCCAGGGCCGCCCGGCGCUCGGCAGCCGCCCGCAGCCCCUCGGA
GCCAGAGGAGAGGCGCCCCCGCCGGCCGCCGCGCCGCCCGGCAGCCUCGGCAGGUACUGC
GGGCAGGCGGCGGGGAGGGAGGGCGCGGAGC

rnafold


?

```
Visualisation of predicted secondary structure.
To save the image:
  Right click on the image -> Save Image as.
```


Turbo-fast


?

```
Visualisation of predicted secondary structure.
To save the image:
  Right click on the image -> Save Image as.
```

Load Sequence viewer

### Hit: NC\_000004.12

NC\_000004.12 Homo sapiens chromosome 4, GRCh38.p13 Primary Assembly

```
?

```
This is BLAST alignment as read from the input file
```

Score = 40.0 bits (37.4), Expect = 4.69E+00
 Identities = 30/34 (88%), Gaps = 2/34 (6%)
 Strand = Plus/Plus
Query     119 CAGGCAGGCGGCGGGCAGCG-GGAGGC-GGCAGC 150    
              |||| ||| ||||||||||| |||||| ||||||        
Sbjct 6761980 CAGGGAGGAGGCGGGCAGCGAGGAGGCGGGCAGC 6762013
```

Report:

|  |  |
| --- | --- |
| sequence start ?  ``` Start position of the estimated full-length sequence in genome. Start index < end index. ``` : | 6761862 |
| sequence end ?  ``` End position of the estimated full-length sequence in genome. Start index < end index. ``` : | 6762013 |
| bit score (CM) ?  ``` The score for aligning estimated full-length sequence to CM model   (computed by RSEARCH -> default,   infered from Rfam or provided by user) ``` : | -22.0 |
| Homology estimate ?  ``` Quick homology estimate:   Not homologous: bit score < 0   Homologous: bit score > 20 and bit score > 0.5 * query length   Uncertain otherwise ``` : | Not homologous |

Estimated full-length sequence:


?

```
Click checkbox to select multiple seuqences.
Fasta header format:
  UID|accession.versionSTRAND start-end
```

>uid:26|NC\_000004.12fw 6761862-6762013
GGCGCUGAGGGUGGGCUGACGCUCACAUUUCCGCACUGGCCCCAUUUCUUGCUGCAGAAA
CGCACUGACUGCCUUCACAGGCUUCCUGCAGCUGAGCUGGGGGCCUCCGCCUGGCAGCCA
GGGAGGAGGCGGGCAGCGAGGAGGCGGGCAGC

rnafold


?

```
Visualisation of predicted secondary structure.
To save the image:
  Right click on the image -> Save Image as.
```


Turbo-fast


?

```
Visualisation of predicted secondary structure.
To save the image:
  Right click on the image -> Save Image as.
```

Load Sequence viewer

### Hit: NC\_000016.10

NC\_000016.10 Homo sapiens chromosome 16, GRCh38.p13 Primary Assembly

```
?

```
This is BLAST alignment as read from the input file
```

Score = 42.0 bits (39.2), Expect = 1.34E+00
 Identities = 50/65 (77%), Gaps = 7/65 (11%)
 Strand = Plus/Minus
Query      84 GGTGCGGAGCGCCGCTGCGCAGCCGGGGAGGGACGCAGGCA-GGCGG--CGGGCAGCGGGAG 142    
              ||||||| | | || ||||  |||||||   ||||| |||  |||||  ||||||||||| |        
Sbjct 1826708 GGTGCGGCGGGGCGGTGCGG-GCCGGGG---GACGCGGGCCTGGCGGGGCGGGCAGCGGGCG 1826651

Query     143 GCG 145    
              |||        
Sbjct 1826650 GCG 1826648
```

Report:

|  |  |
| --- | --- |
| sequence start ?  ``` Start position of the estimated full-length sequence in genome. Start index < end index. ``` : | 1826650 |
| sequence end ?  ``` End position of the estimated full-length sequence in genome. Start index < end index. ``` : | 1826791 |
| bit score (CM) ?  ``` The score for aligning estimated full-length sequence to CM model   (computed by RSEARCH -> default,   infered from Rfam or provided by user) ``` : | -3.87 |
| Homology estimate ?  ``` Quick homology estimate:   Not homologous: bit score < 0   Homologous: bit score > 20 and bit score > 0.5 * query length   Uncertain otherwise ``` : | Not homologous |

Estimated full-length sequence:


?

```
Click checkbox to select multiple seuqences.
Fasta header format:
  UID|accession.versionSTRAND start-end
```

>uid:27|NC\_000016.10rc 1826650-1826791
GUGGUGGGCCGAGGGCUGCUCGGCCGCCGCAGCCUCGCCGCGCUGGGAGCCGCCUGCGCC
CGCCGAGGCCUCGGUGGGUGCGGCGGGGCGGUGCGGGCCGGGGGACGCGGGCCUGGCGGG
GCGGGCAGCGGGCGGCGCCGGG

rnafold


?

```
Visualisation of predicted secondary structure.
To save the image:
  Right click on the image -> Save Image as.
```


Turbo-fast


?

```
Visualisation of predicted secondary structure.
To save the image:
  Right click on the image -> Save Image as.
```

Load Sequence viewer

### Hit: NT\_006051.19

NT\_006051.19 Homo sapiens chromosome 4 genomic scaffold, GRCh38.p13 Primary Assembly HSCHR4\_CTG1

```
?

```
This is BLAST alignment as read from the input file
```

Score = 42.0 bits (39.2), Expect = 1.34E+00
 Identities = 40/49 (82%), Gaps = 4/49 (8%)
 Strand = Plus/Plus
Query     102 GCAGCCGGGGAGG-GACG--CAGGCAGGCGGCGGGCAGCG-GGAGGCGG 146    
              |||||| |||||| | ||  |||  ||| ||||||||||| ||||||||        
Sbjct 6751975 GCAGCCAGGGAGGAGGCGGGCAGCGAGGAGGCGGGCAGCGAGGAGGCGG 6752023
```

Report:

|  |  |
| --- | --- |
| sequence start ?  ``` Start position of the estimated full-length sequence in genome. Start index < end index. ``` : | 6751874 |
| sequence end ?  ``` End position of the estimated full-length sequence in genome. Start index < end index. ``` : | 6752028 |
| bit score (CM) ?  ``` The score for aligning estimated full-length sequence to CM model   (computed by RSEARCH -> default,   infered from Rfam or provided by user) ``` : | -13.21 |
| Homology estimate ?  ``` Quick homology estimate:   Not homologous: bit score < 0   Homologous: bit score > 20 and bit score > 0.5 * query length   Uncertain otherwise ``` : | Not homologous |

Estimated full-length sequence:


?

```
Click checkbox to select multiple seuqences.
Fasta header format:
  UID|accession.versionSTRAND start-end
```

>uid:28|NT\_006051.19fw 6751874-6752028
GGGCUGACGCUCACAUUUCCGCACUGGCCCCAUUUCUUGCUGCAGAAACGCACUGACUGC
CUUCACAGGCUUCCUGCAGCUGAGCUGGGGGCCUCCGCCUGGCAGCCAGGGAGGAGGCGG
GCAGCGAGGAGGCGGGCAGCGAGGAGGCGGGCAGG

rnafold


?

```
Visualisation of predicted secondary structure.
To save the image:
  Right click on the image -> Save Image as.
```


Turbo-fast


?

```
Visualisation of predicted secondary structure.
To save the image:
  Right click on the image -> Save Image as.
```

Load Sequence viewer

### Hit: NT\_006051.19

NT\_006051.19 Homo sapiens chromosome 4 genomic scaffold, GRCh38.p13 Primary Assembly HSCHR4\_CTG1

```
?

```
This is BLAST alignment as read from the input file
```

Score = 41.0 bits (38.3), Expect = 4.69E+00
 Identities = 53/71 (75%), Gaps = 4/71 (6%)
 Strand = Plus/Plus
Query      75 CGCCCCAGCGGTGCGGAGCGCCGCTGCGCAGCCGGGG-AGGGAC-GCAGGCAGGCGGCGGGC 134    
              |||||| || |  ||  |||||||   ||||||  || ||| || || |||||||||||||         
Sbjct 6772802 CGCCCCCGCCGGCCGCCGCGCCGCCCGGCAGCCTCGGCAGGTACTGCGGGCAGGCGGCGGG- 6772862

Query     135 AGCGGGAGG 143    
               | ||||||        
Sbjct 6772863 -GAGGGAGG 6772870
```

Report:

|  |  |
| --- | --- |
| sequence start ?  ``` Start position of the estimated full-length sequence in genome. Start index < end index. ``` : | 6772729 |
| sequence end ?  ``` End position of the estimated full-length sequence in genome. Start index < end index. ``` : | 6772879 |
| bit score (CM) ?  ``` The score for aligning estimated full-length sequence to CM model   (computed by RSEARCH -> default,   infered from Rfam or provided by user) ``` : | -21.46 |
| Homology estimate ?  ``` Quick homology estimate:   Not homologous: bit score < 0   Homologous: bit score > 20 and bit score > 0.5 * query length   Uncertain otherwise ``` : | Not homologous |

Estimated full-length sequence:


?

```
Click checkbox to select multiple seuqences.
Fasta header format:
  UID|accession.versionSTRAND start-end
```

>uid:29|NT\_006051.19fw 6772729-6772879
UUUACGUAAGGCGCAGGCCAGGGCCGCCCGGCGCUCGGCAGCCGCCCGCAGCCCCUCGGA
GCCAGAGGAGAGGCGCCCCCGCCGGCCGCCGCGCCGCCCGGCAGCCUCGGCAGGUACUGC
GGGCAGGCGGCGGGGAGGGAGGGCGCGGAGC

rnafold


?

```
Visualisation of predicted secondary structure.
To save the image:
  Right click on the image -> Save Image as.
```


Turbo-fast


?

```
Visualisation of predicted secondary structure.
To save the image:
  Right click on the image -> Save Image as.
```

Load Sequence viewer

### Hit: NT\_006051.19

NT\_006051.19 Homo sapiens chromosome 4 genomic scaffold, GRCh38.p13 Primary Assembly HSCHR4\_CTG1

```
?

```
This is BLAST alignment as read from the input file
```

Score = 40.0 bits (37.4), Expect = 4.69E+00
 Identities = 30/34 (88%), Gaps = 2/34 (6%)
 Strand = Plus/Plus
Query     119 CAGGCAGGCGGCGGGCAGCG-GGAGGC-GGCAGC 150    
              |||| ||| ||||||||||| |||||| ||||||        
Sbjct 6751980 CAGGGAGGAGGCGGGCAGCGAGGAGGCGGGCAGC 6752013
```

Report:

|  |  |
| --- | --- |
| sequence start ?  ``` Start position of the estimated full-length sequence in genome. Start index < end index. ``` : | 6751862 |
| sequence end ?  ``` End position of the estimated full-length sequence in genome. Start index < end index. ``` : | 6752013 |
| bit score (CM) ?  ``` The score for aligning estimated full-length sequence to CM model   (computed by RSEARCH -> default,   infered from Rfam or provided by user) ``` : | -22.0 |
| Homology estimate ?  ``` Quick homology estimate:   Not homologous: bit score < 0   Homologous: bit score > 20 and bit score > 0.5 * query length   Uncertain otherwise ``` : | Not homologous |

Estimated full-length sequence:


?

```
Click checkbox to select multiple seuqences.
Fasta header format:
  UID|accession.versionSTRAND start-end
```

>uid:30|NT\_006051.19fw 6751862-6752013
GGCGCUGAGGGUGGGCUGACGCUCACAUUUCCGCACUGGCCCCAUUUCUUGCUGCAGAAA
CGCACUGACUGCCUUCACAGGCUUCCUGCAGCUGAGCUGGGGGCCUCCGCCUGGCAGCCA
GGGAGGAGGCGGGCAGCGAGGAGGCGGGCAGC

rnafold


?

```
Visualisation of predicted secondary structure.
To save the image:
  Right click on the image -> Save Image as.
```


Turbo-fast


?

```
Visualisation of predicted secondary structure.
To save the image:
  Right click on the image -> Save Image as.
```

Load Sequence viewer

### Hit: NT\_010393.17

NT\_010393.17 Homo sapiens chromosome 16 genomic scaffold, GRCh38.p13 Primary Assembly HSCHR16\_CTG1

```
?

```
This is BLAST alignment as read from the input file
```

Score = 42.0 bits (39.2), Expect = 1.34E+00
 Identities = 50/65 (77%), Gaps = 7/65 (11%)
 Strand = Plus/Minus
Query      84 GGTGCGGAGCGCCGCTGCGCAGCCGGGGAGGGACGCAGGCA-GGCGG--CGGGCAGCGGGAG 142    
              ||||||| | | || ||||  |||||||   ||||| |||  |||||  ||||||||||| |        
Sbjct 1816708 GGTGCGGCGGGGCGGTGCGG-GCCGGGG---GACGCGGGCCTGGCGGGGCGGGCAGCGGGCG 1816651

Query     143 GCG 145    
              |||        
Sbjct 1816650 GCG 1816648
```

Report:

|  |  |
| --- | --- |
| sequence start ?  ``` Start position of the estimated full-length sequence in genome. Start index < end index. ``` : | 1816650 |
| sequence end ?  ``` End position of the estimated full-length sequence in genome. Start index < end index. ``` : | 1816791 |
| bit score (CM) ?  ``` The score for aligning estimated full-length sequence to CM model   (computed by RSEARCH -> default,   infered from Rfam or provided by user) ``` : | -3.87 |
| Homology estimate ?  ``` Quick homology estimate:   Not homologous: bit score < 0   Homologous: bit score > 20 and bit score > 0.5 * query length   Uncertain otherwise ``` : | Not homologous |

Estimated full-length sequence:


?

```
Click checkbox to select multiple seuqences.
Fasta header format:
  UID|accession.versionSTRAND start-end
```

>uid:31|NT\_010393.17rc 1816650-1816791
GUGGUGGGCCGAGGGCUGCUCGGCCGCCGCAGCCUCGCCGCGCUGGGAGCCGCCUGCGCC
CGCCGAGGCCUCGGUGGGUGCGGCGGGGCGGUGCGGGCCGGGGGACGCGGGCCUGGCGGG
GCGGGCAGCGGGCGGCGCCGGG

rnafold


?

```
Visualisation of predicted secondary structure.
To save the image:
  Right click on the image -> Save Image as.
```


Turbo-fast


?

```
Visualisation of predicted secondary structure.
To save the image:
  Right click on the image -> Save Image as.
```

Load Sequence viewer

### Hit: NG\_011776.1

NG\_011776.1 Homo sapiens euchromatic histone lysine methyltransferase 1 (EHMT1), RefSeqGene on chromosome 9

```
?

```
This is BLAST alignment as read from the input file
```

Score = 41.0 bits (38.3), Expect = 4.69E+00
 Identities = 43/56 (77%), Gaps = 4/56 (7%)
 Strand = Plus/Plus
Query   94 GCCGCTGCGCAGCCGGGGAGGGACGCAGGCAGGCGGCGGGCAGCG--GGAGGCGGC 147 
           || || | || | ||||| | | ||| ||  ||||||||||||||  |||||||||     
Sbjct 5063 GCAGCGGGGCCGGCGGGGGGCGGCGCGGG--GGCGGCGGGCAGCGGCGGAGGCGGC 5116
```

Report:

|  |  |
| --- | --- |
| sequence start ?  ``` Start position of the estimated full-length sequence in genome. Start index < end index. ``` : | 4969 |
| sequence end ?  ``` End position of the estimated full-length sequence in genome. Start index < end index. ``` : | 5119 |
| bit score (CM) ?  ``` The score for aligning estimated full-length sequence to CM model   (computed by RSEARCH -> default,   infered from Rfam or provided by user) ``` : | -21.76 |
| Homology estimate ?  ``` Quick homology estimate:   Not homologous: bit score < 0   Homologous: bit score > 20 and bit score > 0.5 * query length   Uncertain otherwise ``` : | Not homologous |

Estimated full-length sequence:


?

```
Click checkbox to select multiple seuqences.
Fasta header format:
  UID|accession.versionSTRAND start-end
```

>uid:32|NG\_011776.1fw 4969-5119
UCUGGGCCGCGAGGCGCGGGCGGGGCGAUGGCGCGCGGGAGGGGCGGGGCCACGCUGCGG
GCCCGGGCCAUGGCCGCCGCCGAUGCCGAGGUGAGCAGCGGGGCCGGCGGGGGGCGGCGC
GGGGGCGGCGGGCAGCGGCGGAGGCGGCGCG

rnafold


?

```
Visualisation of predicted secondary structure.
To save the image:
  Right click on the image -> Save Image as.
```


Turbo-fast


?

```
Visualisation of predicted secondary structure.
To save the image:
  Right click on the image -> Save Image as.
```

Load Sequence viewer

### Hit: NG\_009081.1

NG\_009081.1 Homo sapiens actinin alpha 2 (ACTN2), RefSeqGene on chromosome 1

```
?

```
This is BLAST alignment as read from the input file
```

Score = 41.0 bits (38.3), Expect = 4.69E+00
 Identities = 28/33 (85%), Gaps = 0/33 (0%)
 Strand = Plus/Minus
Query  114 GGACGCAGGCAGGCGGCGGGCAGCGGGAGGCGG 146 
           |||| || | ||||||||||| ||||| |||||     
Sbjct 5138 GGACCCACGGAGGCGGCGGGCGGCGGGCGGCGG 5106
```

Report:

|  |  |
| --- | --- |
| sequence start ?  ``` Start position of the estimated full-length sequence in genome. Start index < end index. ``` : | 5101 |
| sequence end ?  ``` End position of the estimated full-length sequence in genome. Start index < end index. ``` : | 5251 |
| bit score (CM) ?  ``` The score for aligning estimated full-length sequence to CM model   (computed by RSEARCH -> default,   infered from Rfam or provided by user) ``` : | -19.63 |
| Homology estimate ?  ``` Quick homology estimate:   Not homologous: bit score < 0   Homologous: bit score > 20 and bit score > 0.5 * query length   Uncertain otherwise ``` : | Not homologous |

Estimated full-length sequence:


?

```
Click checkbox to select multiple seuqences.
Fasta header format:
  UID|accession.versionSTRAND start-end
```

>uid:33|NG\_009081.1rc 5101-5251
CUCGUCGUACACGUAGUUGUACUGCACGCCGGGCUCUAUCUGGUUCAUGGCGCUCGGUUG
GCCGGGGCUGCGGCGGGGCGCGAGGGGCUCGGACGCACGGGCUGACUGGCAAACGGACCC
ACGGAGGCGGCGGGCGGCGGGCGGCGGGCGC

rnafold


?

```
Visualisation of predicted secondary structure.
To save the image:
  Right click on the image -> Save Image as.
```


Turbo-fast


?

```
Visualisation of predicted secondary structure.
To save the image:
  Right click on the image -> Save Image as.
```

Load Sequence viewer

### Hit: NG\_007942.1

NG\_007942.1 Homo sapiens glycyl-tRNA synthetase 1 (GARS1), RefSeqGene (LRG\_243) on chromosome 7

```
?

```
This is BLAST alignment as read from the input file
```

Score = 41.0 bits (38.3), Expect = 4.69E+00
 Identities = 28/33 (85%), Gaps = 0/33 (0%)
 Strand = Plus/Minus
Query  101 CGCAGCCGGGGAGGGACGCAGGCAGGCGGCGGG 133 
           || |||||| |||||||||||| || ||| |||     
Sbjct 5847 CGGAGCCGGAGAGGGACGCAGGAAGTCGGAGGG 5815
```

Report:

|  |  |
| --- | --- |
| sequence start ?  ``` Start position of the estimated full-length sequence in genome. Start index < end index. ``` : | 5797 |
| sequence end ?  ``` End position of the estimated full-length sequence in genome. Start index < end index. ``` : | 5946 |
| bit score (CM) ?  ``` The score for aligning estimated full-length sequence to CM model   (computed by RSEARCH -> default,   infered from Rfam or provided by user) ``` : | -19.71 |
| Homology estimate ?  ``` Quick homology estimate:   Not homologous: bit score < 0   Homologous: bit score > 20 and bit score > 0.5 * query length   Uncertain otherwise ``` : | Not homologous |

Estimated full-length sequence:


?

```
Click checkbox to select multiple seuqences.
Fasta header format:
  UID|accession.versionSTRAND start-end
```

>uid:34|NG\_007942.1rc 5797-5946
AGGUUAUUCAGUUAGGAGGCGAGGACAACCAAAGGGUCACAGAUCCCAGCCCCACUGGAA
AUAGAGGAGACGCGGGCUGUGACAGAAUGGGGAAGGGAGAACGGAGCCGGAGAGGGACGC
AGGAAGUCGGAGGGGUUCCAGGGAGUCAUC

rnafold


?

```
Visualisation of predicted secondary structure.
To save the image:
  Right click on the image -> Save Image as.
```


Turbo-fast


?

```
Visualisation of predicted secondary structure.
To save the image:
  Right click on the image -> Save Image as.
```

Load Sequence viewer

### Hit: NC\_000001.11

NC\_000001.11 Homo sapiens chromosome 1, GRCh38.p13 Primary Assembly

```
?

```
This is BLAST alignment as read from the input file
```

Score = 41.0 bits (38.3), Expect = 4.69E+00
 Identities = 28/33 (85%), Gaps = 0/33 (0%)
 Strand = Plus/Minus
Query       114 GGACGCAGGCAGGCGGCGGGCAGCGGGAGGCGG 146      
                |||| || | ||||||||||| ||||| |||||          
Sbjct 236686607 GGACCCACGGAGGCGGCGGGCGGCGGGCGGCGG 236686575
```

Report:

|  |  |
| --- | --- |
| sequence start ?  ``` Start position of the estimated full-length sequence in genome. Start index < end index. ``` : | 236686570 |
| sequence end ?  ``` End position of the estimated full-length sequence in genome. Start index < end index. ``` : | 236686720 |
| bit score (CM) ?  ``` The score for aligning estimated full-length sequence to CM model   (computed by RSEARCH -> default,   infered from Rfam or provided by user) ``` : | -19.63 |
| Homology estimate ?  ``` Quick homology estimate:   Not homologous: bit score < 0   Homologous: bit score > 20 and bit score > 0.5 * query length   Uncertain otherwise ``` : | Not homologous |

Estimated full-length sequence:


?

```
Click checkbox to select multiple seuqences.
Fasta header format:
  UID|accession.versionSTRAND start-end
```

>uid:35|NC\_000001.11rc 236686570-236686720
CUCGUCGUACACGUAGUUGUACUGCACGCCGGGCUCUAUCUGGUUCAUGGCGCUCGGUUG
GCCGGGGCUGCGGCGGGGCGCGAGGGGCUCGGACGCACGGGCUGACUGGCAAACGGACCC
ACGGAGGCGGCGGGCGGCGGGCGGCGGGCGC

rnafold


?

```
Visualisation of predicted secondary structure.
To save the image:
  Right click on the image -> Save Image as.
```


Turbo-fast


?

```
Visualisation of predicted secondary structure.
To save the image:
  Right click on the image -> Save Image as.
```

Load Sequence viewer

### Hit: NC\_000001.11

NC\_000001.11 Homo sapiens chromosome 1, GRCh38.p13 Primary Assembly

```
?

```
This is BLAST alignment as read from the input file
```

Score = 40.0 bits (37.4), Expect = 4.69E+00
 Identities = 31/37 (84%), Gaps = 1/37 (3%)
 Strand = Plus/Minus
Query       94 GCCGCTGC-GCAGCCGGGGAGGGACGCAGGCAGGCGG 129     
               ||||| || |||| |||| ||| | ||||||||||||         
Sbjct 44645755 GCCGCCGCAGCAGGCGGGCAGGCAGGCAGGCAGGCGG 44645719
```

Report:

|  |  |
| --- | --- |
| sequence start ?  ``` Start position of the estimated full-length sequence in genome. Start index < end index. ``` : | 44645695 |
| sequence end ?  ``` End position of the estimated full-length sequence in genome. Start index < end index. ``` : | 44645848 |
| bit score (CM) ?  ``` The score for aligning estimated full-length sequence to CM model   (computed by RSEARCH -> default,   infered from Rfam or provided by user) ``` : | -16.59 |
| Homology estimate ?  ``` Quick homology estimate:   Not homologous: bit score < 0   Homologous: bit score > 20 and bit score > 0.5 * query length   Uncertain otherwise ``` : | Not homologous |

Estimated full-length sequence:


?

```
Click checkbox to select multiple seuqences.
Fasta header format:
  UID|accession.versionSTRAND start-end
```

>uid:36|NC\_000001.11rc 44645695-44645848
AAACCAAUUUAGCUCCCGAUUAAGAAACAGAAACUGGAGAUGAAUCAAGUGUGCCGGCUG
GGCUGGGCGGCCAUACAUCACAUUCCCCGGGCGAAGGCCGCCGCAGCAGGCGGGCAGGCA
GGCAGGCAGGCGGGUGGACUGCUCCUCCCACCGC

rnafold


?

```
Visualisation of predicted secondary structure.
To save the image:
  Right click on the image -> Save Image as.
```


Turbo-fast


?

```
Visualisation of predicted secondary structure.
To save the image:
  Right click on the image -> Save Image as.
```

Load Sequence viewer

### Hit: NC\_000009.12

NC\_000009.12 Homo sapiens chromosome 9, GRCh38.p13 Primary Assembly

```
?

```
This is BLAST alignment as read from the input file
```

Score = 41.0 bits (38.3), Expect = 4.69E+00
 Identities = 43/56 (77%), Gaps = 4/56 (7%)
 Strand = Plus/Plus
Query        94 GCCGCTGCGCAGCCGGGGAGGGACGCAGGCAGGCGGCGGGCAGCG--GGAGGCGGC 147      
                || || | || | ||||| | | ||| ||  ||||||||||||||  |||||||||          
Sbjct 137619054 GCAGCGGGGCCGGCGGGGGGCGGCGCGGG--GGCGGCGGGCAGCGGCGGAGGCGGC 137619107
```

Report:

|  |  |
| --- | --- |
| sequence start ?  ``` Start position of the estimated full-length sequence in genome. Start index < end index. ``` : | 137618960 |
| sequence end ?  ``` End position of the estimated full-length sequence in genome. Start index < end index. ``` : | 137619110 |
| bit score (CM) ?  ``` The score for aligning estimated full-length sequence to CM model   (computed by RSEARCH -> default,   infered from Rfam or provided by user) ``` : | -21.76 |
| Homology estimate ?  ``` Quick homology estimate:   Not homologous: bit score < 0   Homologous: bit score > 20 and bit score > 0.5 * query length   Uncertain otherwise ``` : | Not homologous |

Estimated full-length sequence:


?

```
Click checkbox to select multiple seuqences.
Fasta header format:
  UID|accession.versionSTRAND start-end
```

>uid:37|NC\_000009.12fw 137618960-137619110
UCUGGGCCGCGAGGCGCGGGCGGGGCGAUGGCGCGCGGGAGGGGCGGGGCCACGCUGCGG
GCCCGGGCCAUGGCCGCCGCCGAUGCCGAGGUGAGCAGCGGGGCCGGCGGGGGGCGGCGC
GGGGGCGGCGGGCAGCGGCGGAGGCGGCGCG

rnafold


?

```
Visualisation of predicted secondary structure.
To save the image:
  Right click on the image -> Save Image as.
```


Turbo-fast


?

```
Visualisation of predicted secondary structure.
To save the image:
  Right click on the image -> Save Image as.
```

Load Sequence viewer

### Hit: NC\_000009.12

NC\_000009.12 Homo sapiens chromosome 9, GRCh38.p13 Primary Assembly

```
?

```
This is BLAST alignment as read from the input file
```

Score = 40.0 bits (37.4), Expect = 4.69E+00
 Identities = 23/25 (92%), Gaps = 0/25 (0%)
 Strand = Plus/Plus
Query       121 GGCAGGCGGCGGGCAGCGGGAGGCG 145      
                ||| |||||||||||||||| ||||          
Sbjct 104991746 GGCGGGCGGCGGGCAGCGGGCGGCG 104991770
```

Report:

|  |  |
| --- | --- |
| sequence start ?  ``` Start position of the estimated full-length sequence in genome. Start index < end index. ``` : | 104991627 |
| sequence end ?  ``` End position of the estimated full-length sequence in genome. Start index < end index. ``` : | 104991774 |
| bit score (CM) ?  ``` The score for aligning estimated full-length sequence to CM model   (computed by RSEARCH -> default,   infered from Rfam or provided by user) ``` : | -16.51 |
| Homology estimate ?  ``` Quick homology estimate:   Not homologous: bit score < 0   Homologous: bit score > 20 and bit score > 0.5 * query length   Uncertain otherwise ``` : | Not homologous |

Estimated full-length sequence:


?

```
Click checkbox to select multiple seuqences.
Fasta header format:
  UID|accession.versionSTRAND start-end
```

>uid:38|NC\_000009.12fw 104991627-104991774
AGAGGGCCCAAUGUCGCCCCGCCAGACCCAGAAUUAGGGCACGUCUGCAAAGCUCACACC
CUCCAUUUGAGAGCCGAACCGAAACCAUCACAAGAUGAAACCUCCGGACUCCCAGCCGCG
GCGGGCGGCGGGCAGCGGGCGGCGCGCA

rnafold


?

```
Visualisation of predicted secondary structure.
To save the image:
  Right click on the image -> Save Image as.
```


Turbo-fast


?

```
Visualisation of predicted secondary structure.
To save the image:
  Right click on the image -> Save Image as.
```

Load Sequence viewer

### Hit: NC\_000009.12

NC\_000009.12 Homo sapiens chromosome 9, GRCh38.p13 Primary Assembly

```
?

```
This is BLAST alignment as read from the input file
```

Score = 40.0 bits (37.4), Expect = 4.69E+00
 Identities = 25/27 (93%), Gaps = 1/27 (4%)
 Strand = Plus/Minus
Query       121 GGCAGGCGGCGGGCAGCGGGAGGCGGC 147      
                ||| |||||||||||||||| ||||||          
Sbjct 135736605 GGCGGGCGGCGGGCAGCGGG-GGCGGC 135736580
```

Report:

|  |  |
| --- | --- |
| sequence start ?  ``` Start position of the estimated full-length sequence in genome. Start index < end index. ``` : | 135736571 |
| sequence end ?  ``` End position of the estimated full-length sequence in genome. Start index < end index. ``` : | 135736725 |
| bit score (CM) ?  ``` The score for aligning estimated full-length sequence to CM model   (computed by RSEARCH -> default,   infered from Rfam or provided by user) ``` : | -10.21 |
| Homology estimate ?  ``` Quick homology estimate:   Not homologous: bit score < 0   Homologous: bit score > 20 and bit score > 0.5 * query length   Uncertain otherwise ``` : | Not homologous |

Estimated full-length sequence:


?

```
Click checkbox to select multiple seuqences.
Fasta header format:
  UID|accession.versionSTRAND start-end
```

>uid:39|NC\_000009.12rc 135736571-135736725
GAGCCCGUGAGGCUCCUCGGGGGCUGCGGCGCCGGCUGGGGCGCCCCCCGGGCCCGCCUU
GCCCUCGGACGGCGAGCGCGGCAGCUUGGCCCGCGCCAUCCUGCGGGAGCGCUCGGCGCG
GCGGGCGGCGGGCGGCGGGCAGCGGGGGCGGCGCC

rnafold


?

```
Visualisation of predicted secondary structure.
To save the image:
  Right click on the image -> Save Image as.
```


Turbo-fast


?

```
Visualisation of predicted secondary structure.
To save the image:
  Right click on the image -> Save Image as.
```

Load Sequence viewer

### Hit: NC\_000017.11

NC\_000017.11 Homo sapiens chromosome 17, GRCh38.p13 Primary Assembly

```
?

```
This is BLAST alignment as read from the input file
```

Score = 41.0 bits (38.3), Expect = 4.69E+00
 Identities = 28/33 (85%), Gaps = 0/33 (0%)
 Strand = Plus/Minus
Query      118 GCAGGCAGGCGGCGGGCAGCGGGAGGCGGCAGC 150     
               |||||| || ||||||  |||||||||||| ||         
Sbjct 44968346 GCAGGCGGGAGGCGGGAGGCGGGAGGCGGCGGC 44968314
```

Report:

|  |  |
| --- | --- |
| sequence start ?  ``` Start position of the estimated full-length sequence in genome. Start index < end index. ``` : | 44968313 |
| sequence end ?  ``` End position of the estimated full-length sequence in genome. Start index < end index. ``` : | 44968463 |
| bit score (CM) ?  ``` The score for aligning estimated full-length sequence to CM model   (computed by RSEARCH -> default,   infered from Rfam or provided by user) ``` : | -5.08 |
| Homology estimate ?  ``` Quick homology estimate:   Not homologous: bit score < 0   Homologous: bit score > 20 and bit score > 0.5 * query length   Uncertain otherwise ``` : | Not homologous |

Estimated full-length sequence:


?

```
Click checkbox to select multiple seuqences.
Fasta header format:
  UID|accession.versionSTRAND start-end
```

>uid:40|NC\_000017.11rc 44968313-44968463
GCCCCGCCCGCAGCCCCACCCCUAGAGAGCCCAGAGCCGCGCGGAGCUGGCGGGGCGGGG
CCCGGCCGAGGGGGCGGUGGCCCGGCUCAAGGAGGCGGGAGGAGUCAGGCGGGAGGCUGC
AGGCGGGAGGCGGGAGGCGGGAGGCGGCGGC

rnafold


?

```
Visualisation of predicted secondary structure.
To save the image:
  Right click on the image -> Save Image as.
```


Turbo-fast


?

```
Visualisation of predicted secondary structure.
To save the image:
  Right click on the image -> Save Image as.
```

Load Sequence viewer

### Hit: NT\_167186.2

NT\_167186.2 Homo sapiens chromosome 1 genomic scaffold, GRCh38.p13 Primary Assembly HSCHR1\_CTG32\_1

```
?

```
This is BLAST alignment as read from the input file
```

Score = 41.0 bits (38.3), Expect = 4.69E+00
 Identities = 28/33 (85%), Gaps = 0/33 (0%)
 Strand = Plus/Minus
Query      114 GGACGCAGGCAGGCGGCGGGCAGCGGGAGGCGG 146     
               |||| || | ||||||||||| ||||| |||||         
Sbjct 13077672 GGACCCACGGAGGCGGCGGGCGGCGGGCGGCGG 13077640
```

Report:

|  |  |
| --- | --- |
| sequence start ?  ``` Start position of the estimated full-length sequence in genome. Start index < end index. ``` : | 13077635 |
| sequence end ?  ``` End position of the estimated full-length sequence in genome. Start index < end index. ``` : | 13077785 |
| bit score (CM) ?  ``` The score for aligning estimated full-length sequence to CM model   (computed by RSEARCH -> default,   infered from Rfam or provided by user) ``` : | -19.63 |
| Homology estimate ?  ``` Quick homology estimate:   Not homologous: bit score < 0   Homologous: bit score > 20 and bit score > 0.5 * query length   Uncertain otherwise ``` : | Not homologous |

Estimated full-length sequence:


?

```
Click checkbox to select multiple seuqences.
Fasta header format:
  UID|accession.versionSTRAND start-end
```

>uid:41|NT\_167186.2rc 13077635-13077785
CUCGUCGUACACGUAGUUGUACUGCACGCCGGGCUCUAUCUGGUUCAUGGCGCUCGGUUG
GCCGGGGCUGCGGCGGGGCGCGAGGGGCUCGGACGCACGGGCUGACUGGCAAACGGACCC
ACGGAGGCGGCGGGCGGCGGGCGGCGGGCGC

rnafold


?

```
Visualisation of predicted secondary structure.
To save the image:
  Right click on the image -> Save Image as.
```


Turbo-fast


?

```
Visualisation of predicted secondary structure.
To save the image:
  Right click on the image -> Save Image as.
```

Load Sequence viewer

### Hit: NT\_008470.20

NT\_008470.20 Homo sapiens chromosome 9 genomic scaffold, GRCh38.p13 Primary Assembly HSCHR9\_CTG35

```
?

```
This is BLAST alignment as read from the input file
```

Score = 41.0 bits (38.3), Expect = 4.69E+00
 Identities = 43/56 (77%), Gaps = 4/56 (7%)
 Strand = Plus/Plus
Query       94 GCCGCTGCGCAGCCGGGGAGGGACGCAGGCAGGCGGCGGGCAGCG--GGAGGCGGC 147     
               || || | || | ||||| | | ||| ||  ||||||||||||||  |||||||||         
Sbjct 69398502 GCAGCGGGGCCGGCGGGGGGCGGCGCGGG--GGCGGCGGGCAGCGGCGGAGGCGGC 69398555
```

Report:

|  |  |
| --- | --- |
| sequence start ?  ``` Start position of the estimated full-length sequence in genome. Start index < end index. ``` : | 69398408 |
| sequence end ?  ``` End position of the estimated full-length sequence in genome. Start index < end index. ``` : | 69398558 |
| bit score (CM) ?  ``` The score for aligning estimated full-length sequence to CM model   (computed by RSEARCH -> default,   infered from Rfam or provided by user) ``` : | -21.76 |
| Homology estimate ?  ``` Quick homology estimate:   Not homologous: bit score < 0   Homologous: bit score > 20 and bit score > 0.5 * query length   Uncertain otherwise ``` : | Not homologous |

Estimated full-length sequence:


?

```
Click checkbox to select multiple seuqences.
Fasta header format:
  UID|accession.versionSTRAND start-end
```

>uid:42|NT\_008470.20fw 69398408-69398558
UCUGGGCCGCGAGGCGCGGGCGGGGCGAUGGCGCGCGGGAGGGGCGGGGCCACGCUGCGG
GCCCGGGCCAUGGCCGCCGCCGAUGCCGAGGUGAGCAGCGGGGCCGGCGGGGGGCGGCGC
GGGGGCGGCGGGCAGCGGCGGAGGCGGCGCG

rnafold


?

```
Visualisation of predicted secondary structure.
To save the image:
  Right click on the image -> Save Image as.
```


Turbo-fast


?

```
Visualisation of predicted secondary structure.
To save the image:
  Right click on the image -> Save Image as.
```

Load Sequence viewer

### Hit: NT\_008470.20

NT\_008470.20 Homo sapiens chromosome 9 genomic scaffold, GRCh38.p13 Primary Assembly HSCHR9\_CTG35

```
?

```
This is BLAST alignment as read from the input file
```

Score = 40.0 bits (37.4), Expect = 4.69E+00
 Identities = 23/25 (92%), Gaps = 0/25 (0%)
 Strand = Plus/Plus
Query      121 GGCAGGCGGCGGGCAGCGGGAGGCG 145     
               ||| |||||||||||||||| ||||         
Sbjct 36771194 GGCGGGCGGCGGGCAGCGGGCGGCG 36771218
```

Report:

|  |  |
| --- | --- |
| sequence start ?  ``` Start position of the estimated full-length sequence in genome. Start index < end index. ``` : | 36771075 |
| sequence end ?  ``` End position of the estimated full-length sequence in genome. Start index < end index. ``` : | 36771222 |
| bit score (CM) ?  ``` The score for aligning estimated full-length sequence to CM model   (computed by RSEARCH -> default,   infered from Rfam or provided by user) ``` : | -16.51 |
| Homology estimate ?  ``` Quick homology estimate:   Not homologous: bit score < 0   Homologous: bit score > 20 and bit score > 0.5 * query length   Uncertain otherwise ``` : | Not homologous |

Estimated full-length sequence:


?

```
Click checkbox to select multiple seuqences.
Fasta header format:
  UID|accession.versionSTRAND start-end
```

>uid:43|NT\_008470.20fw 36771075-36771222
AGAGGGCCCAAUGUCGCCCCGCCAGACCCAGAAUUAGGGCACGUCUGCAAAGCUCACACC
CUCCAUUUGAGAGCCGAACCGAAACCAUCACAAGAUGAAACCUCCGGACUCCCAGCCGCG
GCGGGCGGCGGGCAGCGGGCGGCGCGCA

rnafold


?

```
Visualisation of predicted secondary structure.
To save the image:
  Right click on the image -> Save Image as.
```


Turbo-fast


?

```
Visualisation of predicted secondary structure.
To save the image:
  Right click on the image -> Save Image as.
```

Load Sequence viewer

### Hit: NT\_008470.20

NT\_008470.20 Homo sapiens chromosome 9 genomic scaffold, GRCh38.p13 Primary Assembly HSCHR9\_CTG35

```
?

```
This is BLAST alignment as read from the input file
```

Score = 40.0 bits (37.4), Expect = 4.69E+00
 Identities = 25/27 (93%), Gaps = 1/27 (4%)
 Strand = Plus/Minus
Query      121 GGCAGGCGGCGGGCAGCGGGAGGCGGC 147     
               ||| |||||||||||||||| ||||||         
Sbjct 67516053 GGCGGGCGGCGGGCAGCGGG-GGCGGC 67516028
```

Report:

|  |  |
| --- | --- |
| sequence start ?  ``` Start position of the estimated full-length sequence in genome. Start index < end index. ``` : | 67516019 |
| sequence end ?  ``` End position of the estimated full-length sequence in genome. Start index < end index. ``` : | 67516173 |
| bit score (CM) ?  ``` The score for aligning estimated full-length sequence to CM model   (computed by RSEARCH -> default,   infered from Rfam or provided by user) ``` : | -10.21 |
| Homology estimate ?  ``` Quick homology estimate:   Not homologous: bit score < 0   Homologous: bit score > 20 and bit score > 0.5 * query length   Uncertain otherwise ``` : | Not homologous |

Estimated full-length sequence:


?

```
Click checkbox to select multiple seuqences.
Fasta header format:
  UID|accession.versionSTRAND start-end
```

>uid:44|NT\_008470.20rc 67516019-67516173
GAGCCCGUGAGGCUCCUCGGGGGCUGCGGCGCCGGCUGGGGCGCCCCCCGGGCCCGCCUU
GCCCUCGGACGGCGAGCGCGGCAGCUUGGCCCGCGCCAUCCUGCGGGAGCGCUCGGCGCG
GCGGGCGGCGGGCGGCGGGCAGCGGGGGCGGCGCC

rnafold


?

```
Visualisation of predicted secondary structure.
To save the image:
  Right click on the image -> Save Image as.
```


Turbo-fast


?

```
Visualisation of predicted secondary structure.
To save the image:
  Right click on the image -> Save Image as.
```

Load Sequence viewer

### Hit: NT\_010783.16

NT\_010783.16 Homo sapiens chromosome 17 genomic scaffold, GRCh38.p13 Primary Assembly HSCHR17\_CTG4

```
?

```
This is BLAST alignment as read from the input file
```

Score = 41.0 bits (38.3), Expect = 4.69E+00
 Identities = 28/33 (85%), Gaps = 0/33 (0%)
 Strand = Plus/Minus
Query      118 GCAGGCAGGCGGCGGGCAGCGGGAGGCGGCAGC 150     
               |||||| || ||||||  |||||||||||| ||         
Sbjct 18032366 GCAGGCGGGAGGCGGGAGGCGGGAGGCGGCGGC 18032334
```

Report:

|  |  |
| --- | --- |
| sequence start ?  ``` Start position of the estimated full-length sequence in genome. Start index < end index. ``` : | 18032333 |
| sequence end ?  ``` End position of the estimated full-length sequence in genome. Start index < end index. ``` : | 18032483 |
| bit score (CM) ?  ``` The score for aligning estimated full-length sequence to CM model   (computed by RSEARCH -> default,   infered from Rfam or provided by user) ``` : | -5.08 |
| Homology estimate ?  ``` Quick homology estimate:   Not homologous: bit score < 0   Homologous: bit score > 20 and bit score > 0.5 * query length   Uncertain otherwise ``` : | Not homologous |

Estimated full-length sequence:


?

```
Click checkbox to select multiple seuqences.
Fasta header format:
  UID|accession.versionSTRAND start-end
```

>uid:45|NT\_010783.16rc 18032333-18032483
GCCCCGCCCGCAGCCCCACCCCUAGAGAGCCCAGAGCCGCGCGGAGCUGGCGGGGCGGGG
CCCGGCCGAGGGGGCGGUGGCCCGGCUCAAGGAGGCGGGAGGAGUCAGGCGGGAGGCUGC
AGGCGGGAGGCGGGAGGCGGGAGGCGGCGGC

rnafold


?

```
Visualisation of predicted secondary structure.
To save the image:
  Right click on the image -> Save Image as.
```


Turbo-fast


?

```
Visualisation of predicted secondary structure.
To save the image:
  Right click on the image -> Save Image as.
```

Load Sequence viewer

### Hit: NT\_011387.9

NT\_011387.9 Homo sapiens chromosome 20 genomic scaffold, GRCh38.p13 Primary Assembly HSCHR20\_CTG1

```
?

```
This is BLAST alignment as read from the input file
```

Score = 41.0 bits (38.3), Expect = 4.69E+00
 Identities = 28/33 (85%), Gaps = 0/33 (0%)
 Strand = Plus/Minus
Query    113 GGGACGCAGGCAGGCGGCGGGCAGCGGGAGGCG 145   
             ||| ||||||| ||||||||||| ||| | |||       
Sbjct 604159 GGGGCGCAGGCGGGCGGCGGGCACCGGCACGCG 604127
```

Report:

|  |  |
| --- | --- |
| sequence start ?  ``` Start position of the estimated full-length sequence in genome. Start index < end index. ``` : | 604120 |
| sequence end ?  ``` End position of the estimated full-length sequence in genome. Start index < end index. ``` : | 604271 |
| bit score (CM) ?  ``` The score for aligning estimated full-length sequence to CM model   (computed by RSEARCH -> default,   infered from Rfam or provided by user) ``` : | -17.83 |
| Homology estimate ?  ``` Quick homology estimate:   Not homologous: bit score < 0   Homologous: bit score > 20 and bit score > 0.5 * query length   Uncertain otherwise ``` : | Not homologous |

Estimated full-length sequence:


?

```
Click checkbox to select multiple seuqences.
Fasta header format:
  UID|accession.versionSTRAND start-end
```

>uid:46|NT\_011387.9rc 604120-604271
AUGGACGCCUUCUUCAUCUCGGACGGGCGCUCGCGGCGGCGGCGGGGCGGGGGCGGCGGG
GACGCGGGGGGCUCGGGAGACGCGGGGGGCGCCGGGGGGCGCGCGGGGCGCGCGGGGGCG
CAGGCGGGCGGCGGGCACCGGCACGCGUGCGC

rnafold


?

```
Visualisation of predicted secondary structure.
To save the image:
  Right click on the image -> Save Image as.
```


Turbo-fast


?

```
Visualisation of predicted secondary structure.
To save the image:
  Right click on the image -> Save Image as.
```

Load Sequence viewer

### Hit: NG\_029017.2

NG\_029017.2 Homo sapiens ATPase family AAA domain containing 2B (ATAD2B), RefSeqGene on chromosome 2

```
?

```
This is BLAST alignment as read from the input file
```

Score = 40.0 bits (37.4), Expect = 4.69E+00
 Identities = 49/65 (75%), Gaps = 5/65 (8%)
 Strand = Plus/Plus
Query   90 GAGCGCCGCTGCGCAGCCGGGGAGGG---ACGCAG-GCAGGCGGCGGG-CAGCGGGAGGCGGCAG 149 
           ||||| |||| | | ||||  ||| |   |||| | || || |||||| |||| |||||||||||     
Sbjct 4807 GAGCGTCGCTCCCCGGCCGCCGAGCGCTGACGCCGCGCCGGGGGCGGGGCAGCTGGAGGCGGCAG 4871
```

Report:

|  |  |
| --- | --- |
| sequence start ?  ``` Start position of the estimated full-length sequence in genome. Start index < end index. ``` : | 4733 |
| sequence end ?  ``` End position of the estimated full-length sequence in genome. Start index < end index. ``` : | 4872 |
| bit score (CM) ?  ``` The score for aligning estimated full-length sequence to CM model   (computed by RSEARCH -> default,   infered from Rfam or provided by user) ``` : | 1.62 |
| Homology estimate ?  ``` Quick homology estimate:   Not homologous: bit score < 0   Homologous: bit score > 20 and bit score > 0.5 * query length   Uncertain otherwise ``` : | Uncertain **↴** |

Check the secondary structure and sequence viewer
for supporting information about possible homology.

Estimated full-length sequence:


?

```
Click checkbox to select multiple seuqences.
Fasta header format:
  UID|accession.versionSTRAND start-end
```

>uid:47|NG\_029017.2fw 4733-4872
UGAUUAGUCUGGCUUUUCUGGUCGCCCCAGCCCUGCGGUCGGGGAGAGCUGGAAACCACC
GAGCUGACAGCCUAGAGCGUCGCUCCCCGGCCGCCGAGCGCUGACGCCGCGCCGGGGGCG
GGGCAGCUGGAGGCGGCAGG

rnafold


?

```
Visualisation of predicted secondary structure.
To save the image:
  Right click on the image -> Save Image as.
```


Turbo-fast


?

```
Visualisation of predicted secondary structure.
To save the image:
  Right click on the image -> Save Image as.
```

Load Sequence viewer

### Hit: NG\_033070.1

NG\_033070.1 Homo sapiens potassium sodium-activated channel subfamily T member 1 (KCNT1), RefSeqGene on chromosome 9

```
?

```
This is BLAST alignment as read from the input file
```

Score = 40.0 bits (37.4), Expect = 4.69E+00
 Identities = 25/27 (93%), Gaps = 1/27 (4%)
 Strand = Plus/Minus
Query   121 GGCAGGCGGCGGGCAGCGGGAGGCGGC 147  
            ||| |||||||||||||||| ||||||      
Sbjct 39421 GGCGGGCGGCGGGCAGCGGG-GGCGGC 39396
```

Report:

|  |  |
| --- | --- |
| sequence start ?  ``` Start position of the estimated full-length sequence in genome. Start index < end index. ``` : | 39387 |
| sequence end ?  ``` End position of the estimated full-length sequence in genome. Start index < end index. ``` : | 39541 |
| bit score (CM) ?  ``` The score for aligning estimated full-length sequence to CM model   (computed by RSEARCH -> default,   infered from Rfam or provided by user) ``` : | -10.21 |
| Homology estimate ?  ``` Quick homology estimate:   Not homologous: bit score < 0   Homologous: bit score > 20 and bit score > 0.5 * query length   Uncertain otherwise ``` : | Not homologous |

Estimated full-length sequence:


?

```
Click checkbox to select multiple seuqences.
Fasta header format:
  UID|accession.versionSTRAND start-end
```

>uid:48|NG\_033070.1rc 39387-39541
GAGCCCGUGAGGCUCCUCGGGGGCUGCGGCGCCGGCUGGGGCGCCCCCCGGGCCCGCCUU
GCCCUCGGACGGCGAGCGCGGCAGCUUGGCCCGCGCCAUCCUGCGGGAGCGCUCGGCGCG
GCGGGCGGCGGGCGGCGGGCAGCGGGGGCGGCGCC

rnafold


?

```
Visualisation of predicted secondary structure.
To save the image:
  Right click on the image -> Save Image as.
```


Turbo-fast


?

```
Visualisation of predicted secondary structure.
To save the image:
  Right click on the image -> Save Image as.
```

Load Sequence viewer

### Hit: NC\_000002.12

NC\_000002.12 Homo sapiens chromosome 2, GRCh38.p13 Primary Assembly

```
?

```
This is BLAST alignment as read from the input file
```

Score = 40.0 bits (37.4), Expect = 4.69E+00
 Identities = 49/65 (75%), Gaps = 5/65 (8%)
 Strand = Plus/Minus
Query       90 GAGCGCCGCTGCGCAGCCGGGGAGGG---ACGCAG-GCAGGCGGCGGG-CAGCGGGAGGC 144     
               ||||| |||| | | ||||  ||| |   |||| | || || |||||| |||| ||||||         
Sbjct 23927260 GAGCGTCGCTCCCCGGCCGCCGAGCGCTGACGCCGCGCCGGGGGCGGGGCAGCTGGAGGC 23927201

Query      145 GGCAG 149     
               |||||         
Sbjct 23927200 GGCAG 23927196
```

Report:

|  |  |
| --- | --- |
| sequence start ?  ``` Start position of the estimated full-length sequence in genome. Start index < end index. ``` : | 23927210 |
| sequence end ?  ``` End position of the estimated full-length sequence in genome. Start index < end index. ``` : | 23927349 |
| bit score (CM) ?  ``` The score for aligning estimated full-length sequence to CM model   (computed by RSEARCH -> default,   infered from Rfam or provided by user) ``` : | 1.62 |
| Homology estimate ?  ``` Quick homology estimate:   Not homologous: bit score < 0   Homologous: bit score > 20 and bit score > 0.5 * query length   Uncertain otherwise ``` : | Uncertain **↴** |

Check the secondary structure and sequence viewer
for supporting information about possible homology.

Estimated full-length sequence:


?

```
Click checkbox to select multiple seuqences.
Fasta header format:
  UID|accession.versionSTRAND start-end
```

>uid:49|NC\_000002.12rc 23927210-23927349
UGAUUAGUCUGGCUUUUCUGGUCGCCCCAGCCCUGCGGUCGGGGAGAGCUGGAAACCACC
GAGCUGACAGCCUAGAGCGUCGCUCCCCGGCCGCCGAGCGCUGACGCCGCGCCGGGGGCG
GGGCAGCUGGAGGCGGCAGG

rnafold


?

```
Visualisation of predicted secondary structure.
To save the image:
  Right click on the image -> Save Image as.
```


Turbo-fast


?

```
Visualisation of predicted secondary structure.
To save the image:
  Right click on the image -> Save Image as.
```

Load Sequence viewer

### Hit: NC\_000003.12

NC\_000003.12 Homo sapiens chromosome 3, GRCh38.p13 Primary Assembly

```
?

```
This is BLAST alignment as read from the input file
```

Score = 40.0 bits (37.4), Expect = 4.69E+00
 Identities = 38/49 (78%), Gaps = 2/49 (4%)
 Strand = Plus/Plus
Query       102 GCAGCCGGGGAGGGACGCAGGCAGGCGGCGGGCAGCGGGAGGCGGCAGC 150      
                ||| || ||||||  |  ||||||||||| || ||  |||||| |||||          
Sbjct 185004105 GCACCCCGGGAGG--CCAAGGCAGGCGGCCGGGAGGTGGAGGCTGCAGC 185004151
```

Report:

|  |  |
| --- | --- |
| sequence start ?  ``` Start position of the estimated full-length sequence in genome. Start index < end index. ``` : | 185004008 |
| sequence end ?  ``` End position of the estimated full-length sequence in genome. Start index < end index. ``` : | 185004151 |
| bit score (CM) ?  ``` The score for aligning estimated full-length sequence to CM model   (computed by RSEARCH -> default,   infered from Rfam or provided by user) ``` : | -18.9 |
| Homology estimate ?  ``` Quick homology estimate:   Not homologous: bit score < 0   Homologous: bit score > 20 and bit score > 0.5 * query length   Uncertain otherwise ``` : | Not homologous |

Estimated full-length sequence:


?

```
Click checkbox to select multiple seuqences.
Fasta header format:
  UID|accession.versionSTRAND start-end
```

>uid:50|NC\_000003.12fw 185004008-185004151
GGGCAGAGACGCUCCUCACUUUCCAGACUGGGCAGCCAGGCAGAGAUGCUCCCCACCUCC
CAGAUGGGGUGGCGGCCAGGCAGAGGCUGCAAUCUCGGCACCCCGGGAGGCCAAGGCAGG
CGGCCGGGAGGUGGAGGCUGCAGC

rnafold


?

```
Visualisation of predicted secondary structure.
To save the image:
  Right click on the image -> Save Image as.
```


Turbo-fast


?

```
Visualisation of predicted secondary structure.
To save the image:
  Right click on the image -> Save Image as.
```

Load Sequence viewer

### Hit: NC\_000014.9

NC\_000014.9 Homo sapiens chromosome 14, GRCh38.p13 Primary Assembly

```
?

```
This is BLAST alignment as read from the input file
```

Score = 40.0 bits (37.4), Expect = 4.69E+00
 Identities = 29/34 (85%), Gaps = 2/34 (6%)
 Strand = Plus/Minus
Query       109 GGGAGGGACGCAGGCAGGCG--GCGGGCAGCGGG 140      
                |||||| | |||||||||||  || |||||||||          
Sbjct 104172089 GGGAGGTAAGCAGGCAGGCGCCGCAGGCAGCGGG 104172056
```

Report:

|  |  |
| --- | --- |
| sequence start ?  ``` Start position of the estimated full-length sequence in genome. Start index < end index. ``` : | 104172047 |
| sequence end ?  ``` End position of the estimated full-length sequence in genome. Start index < end index. ``` : | 104172197 |
| bit score (CM) ?  ``` The score for aligning estimated full-length sequence to CM model   (computed by RSEARCH -> default,   infered from Rfam or provided by user) ``` : | -19.47 |
| Homology estimate ?  ``` Quick homology estimate:   Not homologous: bit score < 0   Homologous: bit score > 20 and bit score > 0.5 * query length   Uncertain otherwise ``` : | Not homologous |

Estimated full-length sequence:


?

```
Click checkbox to select multiple seuqences.
Fasta header format:
  UID|accession.versionSTRAND start-end
```

>uid:51|NC\_000014.9rc 104172047-104172197
UUCCCGGGAGGGCGGGCUCCGGAAAACCAGGCCUUUCCCUGUGGACUUGGCCCAGAGCCU
CGGCAGGGGCGGAUGGAGACACCCAAGGACGCUGGCCAGGACGAUGAGGGAGGUAAGCAG
GCAGGCGCCGCAGGCAGCGGGCCCCUCGCUC

rnafold


?

```
Visualisation of predicted secondary structure.
To save the image:
  Right click on the image -> Save Image as.
```


Turbo-fast


?

```
Visualisation of predicted secondary structure.
To save the image:
  Right click on the image -> Save Image as.
```

Load Sequence viewer

### Hit: NT\_032977.10

NT\_032977.10 Homo sapiens chromosome 1 genomic scaffold, GRCh38.p13 Primary Assembly HSCHR1\_CTG3

```
?

```
This is BLAST alignment as read from the input file
```

Score = 40.0 bits (37.4), Expect = 4.69E+00
 Identities = 31/37 (84%), Gaps = 1/37 (3%)
 Strand = Plus/Minus
Query       94 GCCGCTGC-GCAGCCGGGGAGGGACGCAGGCAGGCGG 129     
               ||||| || |||| |||| ||| | ||||||||||||         
Sbjct 44059767 GCCGCCGCAGCAGGCGGGCAGGCAGGCAGGCAGGCGG 44059731
```

Report:

|  |  |
| --- | --- |
| sequence start ?  ``` Start position of the estimated full-length sequence in genome. Start index < end index. ``` : | 44059707 |
| sequence end ?  ``` End position of the estimated full-length sequence in genome. Start index < end index. ``` : | 44059860 |
| bit score (CM) ?  ``` The score for aligning estimated full-length sequence to CM model   (computed by RSEARCH -> default,   infered from Rfam or provided by user) ``` : | -16.59 |
| Homology estimate ?  ``` Quick homology estimate:   Not homologous: bit score < 0   Homologous: bit score > 20 and bit score > 0.5 * query length   Uncertain otherwise ``` : | Not homologous |

Estimated full-length sequence:


?

```
Click checkbox to select multiple seuqences.
Fasta header format:
  UID|accession.versionSTRAND start-end
```

>uid:52|NT\_032977.10rc 44059707-44059860
AAACCAAUUUAGCUCCCGAUUAAGAAACAGAAACUGGAGAUGAAUCAAGUGUGCCGGCUG
GGCUGGGCGGCCAUACAUCACAUUCCCCGGGCGAAGGCCGCCGCAGCAGGCGGGCAGGCA
GGCAGGCAGGCGGGUGGACUGCUCCUCCCACCGC

rnafold


?

```
Visualisation of predicted secondary structure.
To save the image:
  Right click on the image -> Save Image as.
```


Turbo-fast


?

```
Visualisation of predicted secondary structure.
To save the image:
  Right click on the image -> Save Image as.
```

Load Sequence viewer

### Hit: NT\_022184.16

NT\_022184.16 Homo sapiens chromosome 2 genomic scaffold, GRCh38.p13 Primary Assembly HSCHR2\_CTG5

```
?

```
This is BLAST alignment as read from the input file
```

Score = 40.0 bits (37.4), Expect = 4.69E+00
 Identities = 49/65 (75%), Gaps = 5/65 (8%)
 Strand = Plus/Minus
Query      90 GAGCGCCGCTGCGCAGCCGGGGAGGG---ACGCAG-GCAGGCGGCGGG-CAGCGGGAGGCGG 146    
              ||||| |||| | | ||||  ||| |   |||| | || || |||||| |||| ||||||||        
Sbjct 7781141 GAGCGTCGCTCCCCGGCCGCCGAGCGCTGACGCCGCGCCGGGGGCGGGGCAGCTGGAGGCGG 7781080

Query     147 CAG 149    
              |||        
Sbjct 7781079 CAG 7781077
```

Report:

|  |  |
| --- | --- |
| sequence start ?  ``` Start position of the estimated full-length sequence in genome. Start index < end index. ``` : | 7781091 |
| sequence end ?  ``` End position of the estimated full-length sequence in genome. Start index < end index. ``` : | 7781230 |
| bit score (CM) ?  ``` The score for aligning estimated full-length sequence to CM model   (computed by RSEARCH -> default,   infered from Rfam or provided by user) ``` : | 1.62 |
| Homology estimate ?  ``` Quick homology estimate:   Not homologous: bit score < 0   Homologous: bit score > 20 and bit score > 0.5 * query length   Uncertain otherwise ``` : | Uncertain **↴** |

Check the secondary structure and sequence viewer
for supporting information about possible homology.

Estimated full-length sequence:


?

```
Click checkbox to select multiple seuqences.
Fasta header format:
  UID|accession.versionSTRAND start-end
```

>uid:53|NT\_022184.16rc 7781091-7781230
UGAUUAGUCUGGCUUUUCUGGUCGCCCCAGCCCUGCGGUCGGGGAGAGCUGGAAACCACC
GAGCUGACAGCCUAGAGCGUCGCUCCCCGGCCGCCGAGCGCUGACGCCGCGCCGGGGGCG
GGGCAGCUGGAGGCGGCAGG

rnafold


?

```
Visualisation of predicted secondary structure.
To save the image:
  Right click on the image -> Save Image as.
```


Turbo-fast


?

```
Visualisation of predicted secondary structure.
To save the image:
  Right click on the image -> Save Image as.
```

Load Sequence viewer

### Hit: NT\_005612.17

NT\_005612.17 Homo sapiens chromosome 3 genomic scaffold, GRCh38.p13 Primary Assembly HSCHR3\_CTG2\_1

```
?

```
This is BLAST alignment as read from the input file
```

Score = 40.0 bits (37.4), Expect = 4.69E+00
 Identities = 38/49 (78%), Gaps = 2/49 (4%)
 Strand = Plus/Plus
Query      102 GCAGCCGGGGAGGGACGCAGGCAGGCGGCGGGCAGCGGGAGGCGGCAGC 150     
               ||| || ||||||  |  ||||||||||| || ||  |||||| |||||         
Sbjct 91298531 GCACCCCGGGAGG--CCAAGGCAGGCGGCCGGGAGGTGGAGGCTGCAGC 91298577
```

Report:

|  |  |
| --- | --- |
| sequence start ?  ``` Start position of the estimated full-length sequence in genome. Start index < end index. ``` : | 91298434 |
| sequence end ?  ``` End position of the estimated full-length sequence in genome. Start index < end index. ``` : | 91298577 |
| bit score (CM) ?  ``` The score for aligning estimated full-length sequence to CM model   (computed by RSEARCH -> default,   infered from Rfam or provided by user) ``` : | -18.9 |
| Homology estimate ?  ``` Quick homology estimate:   Not homologous: bit score < 0   Homologous: bit score > 20 and bit score > 0.5 * query length   Uncertain otherwise ``` : | Not homologous |

Estimated full-length sequence:


?

```
Click checkbox to select multiple seuqences.
Fasta header format:
  UID|accession.versionSTRAND start-end
```

>uid:54|NT\_005612.17fw 91298434-91298577
GGGCAGAGACGCUCCUCACUUUCCAGACUGGGCAGCCAGGCAGAGAUGCUCCCCACCUCC
CAGAUGGGGUGGCGGCCAGGCAGAGGCUGCAAUCUCGGCACCCCGGGAGGCCAAGGCAGG
CGGCCGGGAGGUGGAGGCUGCAGC

rnafold


?

```
Visualisation of predicted secondary structure.
To save the image:
  Right click on the image -> Save Image as.
```


Turbo-fast


?

```
Visualisation of predicted secondary structure.
To save the image:
  Right click on the image -> Save Image as.
```

Load Sequence viewer

### Hit: NT\_026437.13

NT\_026437.13 Homo sapiens chromosome 14 genomic scaffold, GRCh38.p13 Primary Assembly HSCHR14\_CTG1

```
?

```
This is BLAST alignment as read from the input file
```

Score = 40.0 bits (37.4), Expect = 4.69E+00
 Identities = 29/34 (85%), Gaps = 2/34 (6%)
 Strand = Plus/Minus
Query      109 GGGAGGGACGCAGGCAGGCG--GCGGGCAGCGGG 140     
               |||||| | |||||||||||  || |||||||||         
Sbjct 85948566 GGGAGGTAAGCAGGCAGGCGCCGCAGGCAGCGGG 85948533
```

Report:

|  |  |
| --- | --- |
| sequence start ?  ``` Start position of the estimated full-length sequence in genome. Start index < end index. ``` : | 85948524 |
| sequence end ?  ``` End position of the estimated full-length sequence in genome. Start index < end index. ``` : | 85948674 |
| bit score (CM) ?  ``` The score for aligning estimated full-length sequence to CM model   (computed by RSEARCH -> default,   infered from Rfam or provided by user) ``` : | -19.47 |
| Homology estimate ?  ``` Quick homology estimate:   Not homologous: bit score < 0   Homologous: bit score > 20 and bit score > 0.5 * query length   Uncertain otherwise ``` : | Not homologous |

Estimated full-length sequence:


?

```
Click checkbox to select multiple seuqences.
Fasta header format:
  UID|accession.versionSTRAND start-end
```

>uid:55|NT\_026437.13rc 85948524-85948674
UUCCCGGGAGGGCGGGCUCCGGAAAACCAGGCCUUUCCCUGUGGACUUGGCCCAGAGCCU
CGGCAGGGGCGGAUGGAGACACCCAAGGACGCUGGCCAGGACGAUGAGGGAGGUAAGCAG
GCAGGCGCCGCAGGCAGCGGGCCCCUCGCUC

rnafold


?

```
Visualisation of predicted secondary structure.
To save the image:
  Right click on the image -> Save Image as.
```


Turbo-fast


?

```
Visualisation of predicted secondary structure.
To save the image:
  Right click on the image -> Save Image as.
```

Load Sequence viewer

### Command, parameters and program log

executed command:

/home/pepik/miniconda3/bin/rboAnalyzer -db genomes.bdb -q MYB\_Hs.fa -pm Turbo-fast rnafold rfam-Rc -in WUXKWAHV014-Alignment.xml --html hWUXKWAHV014-Alignment\_new.html

date:
15:16:37 21. 11. 2019

program log:

rfam-Rc: No CM model. Can't use rfam-Rc.

```
parameters:

    b_type: guess

    blast_db: genomes.bdb

    blast_in: WUXKWAHV014-Alignment.xml

    blast_query: MYB_Hs.fa

    blast_regexp: [A-Z][0-9]{5}\.[0-9]+|[A-Z]{2}[0-9]{6}\.[0-9]+|[A-Z]{2}[0-9]{8}\.[0-9]+|[A-Z]{4}[0-9]{8,}\.[0-9]+|[A-Z]{6}[0-9]{9,}\.[0-9]+|AC_[0-9A-Z]+\.[0-9]+|NC_[0-9A-Z]+\.[0-9]+|NG_[0-9A-Z]+\.[0-9]+|NT_[0-9A-Z]+\.[0-9]+|NW_[0-9A-Z]+\.[0-9]+|NZ_[0-9A-Z]+\.[0-9]+|NM_[0-9A-Z]+\.[0-9]+|NR_[0-9A-Z]+\.[0-9]+|XM_[0-9A-Z]+\.[0-9]+|XR_[0-9A-Z]+\.[0-9]+|AP_[0-9A-Z]+\.[0-9]+|NP_[0-9A-Z]+\.[0-9]+|YP_[0-9A-Z]+\.[0-9]+|XP_[0-9A-Z]+\.[0-9]+|WP_[0-9A-Z]+\.[0-9]+|[A-Z]{5}[0-9]{7}\.[0-9]+|[A-Z]{3}[0-9]{5}\.[0-9]+|[A-Z]{3}[0-9]{7}\.[0-9]+|[0-9A-Z]{4}[_|][0-9A-Z]{1,2}|[A-Z]{4}[0-9]{2}S?[0-9]{6,}\.[0-9]+|[A-Z]{6}[0-9]{2}S?[0-9]{7,}\.[0-9]+|1KPD|GPS_[0-9]{9}\.[0-9]+|ZP_[0-9]{8}\.[0-9]+|NS_[0-9]{6}\.[0-9]+

    centroid_fast_preset: False

    cm_file: None

    command: ['/home/pepik/miniconda3/bin/rboAnalyzer', '-db', 'genomes.bdb', '-q', 'MYB_Hs.fa', '-pm', 'Turbo-fast', 'rnafold', 'rfam-Rc', '-in', 'WUXKWAHV014-Alignment.xml', '--html', 'hWUXKWAHV014-Alignment_new.html']

    config_file: None

    csv: None

    db_type: blastdb

    dev_pred: False

    download_rfam: False

    dump: None

    enable_overwrite: False

    entrez: None

    filter_by_bitscore: None

    filter_by_eval: None

    html: hWUXKWAHV014-Alignment_new.html

    json: None

    locarna_anchor_length: 7

    locarna_params: --struct-local=0 --sequ-local=0 --free-endgaps=++++

    logfile: None

    mode: locarna

    pandas_dump: None

    pm_param_file: None

    pred_params: 
    "Turbo-fast": {
        "max_seqs_in_prediction": 4,
        "query_max_len_diff": 0.05
    },
    "rfam-Rc": {},
    "rnafold": {}


    prediction_method: ['Turbo-fast', 'rnafold', 'rfam-Rc']

    repredict_file: None

    sha1: 92c3513a6304b8c4951292730ed2f8db9d6c900f

    show_HSP: False

    show_gene_browser: True

    skip_missing: False

    subseq_window_locarna: 30

    threads: None

    turbo_fast_preset: False

    use_rfam: False

    verbose: 0

    zip_json: False
```

Select all Seqs.
Select all Structs.
Export Sequences
Export Structures
Sort Eval desc.
View all Regions
